# Complementary transcriptomic and proteomic analyses reveal regulatory mechanisms of milk protein production in dairy cows consuming different forages

**DOI:** 10.1038/srep44234

**Published:** 2017-03-14

**Authors:** Wenting Dai, Qiong Chen, Quanjuan Wang, Robin R. White, Jianxin Liu, Hongyun Liu

**Affiliations:** 1 Institute of Dairy Science, College of Animal Sciences, Zhejiang University, Hangzhou 310058, P.R. China; 2 Department of Animal and Poultry Science, Virginia Tech, Blacksburg 24061, United States

## Abstract

Forage plays a critical role in the milk production of dairy cows; however, the mechanisms regulating bovine milk synthesis in dairy cows fed high forage rations with different basal forage types are not well-understood. In the study, rice straw (RS, low-quality) and alfalfa hay (AH, high-quality) diets were fed to lactating cows to explore how forage quality affected the molecular mechanisms regulating milk production using RNA-seq transcriptomic method with iTRAQ proteomic technique. A total of 554 transcripts (423 increased and 131 decreased) and 517 proteins (231 up-regulated and 286 down-regulated) were differentially expressed in the mammary glands of the two groups. The correlation analysis demonstrated seven proteins (six up-regulated and one down-regulated) had consistent mRNA expression. Functional analysis of the differentially expressed transcripts/proteins suggested that enhanced capacity for energy and fatty acid metabolism, increased protein degradation, reduced protein synthesis, decreased amino acid metabolism and depressed cell growth were related to RS consumption. The results indicated cows consuming RS diets may have had depressed milk protein synthesis because these animals had decreased capacity for protein synthesis, enhanced proteolysis, inefficient energy generation and reduced cell growth. Additional work evaluating RS- and AH-based rations may help better isolate molecular adaptations to low nutrient availability during lactation.

Bovine milk is a complex fluid produced by the mammary gland and is an essential source of nutrients for humans^1^. Due to its large population, China must expand its dairy industry to provide sufficient milk to meet domestic demand. Forage is essential in the dairy industry, accounting for at least half of a cow’s daily diet^2^. The efficacy of using forages in dairy rations is largely dependent on forage quality, which can affect energy availability, productivity, and milk quality. For example, alfalfa hay (AH) is a high-quality forage that is commonly used because it helps to improve productivity^3^; however, the availability of AH is limited in China. Importing foreign AH substantially increases production costs; therefore, use of this high-quality forage is often economically infeasible. Consequently, crop residues have received considerable attention as an alternative forage source. In tropical areas such as southern China, rice straw (RS) is the main crop-residue usually stored by farmers as ruminant feed^4^. However, compared with AH, RS is low in digestible energy and crude protein, which limits its application in modern dairy farming^5^. Understanding the molecular mechanisms regulating how various-quality forages differentially affect milk production may help to design alternative feeding or management strategies that make better use of low-quality forages.

Some studies have been conducted to decipher the impacts of roughage type on dairy cattle performance^6^,^7^,^8^. Dairy cows fed AH-based diets had higher milk yield and milk protein content than cows fed corn stover (CS)-based diets^6^,^9^,^10^ and RS-based diets^6^,^11^. In addition, low milk protein production on low-quality forages (CS and RS) was found to be associated with low feed- and nitrogen-use efficiency in mammary glands in a microRNA transcriptomic study^7^. However, further studies are still required to more completely define the differences in mammary gland responses to alternative dietary energy and protein profiles provided by rations with differing forage sources.

Next-generation RNA sequencing technology allows whole transcriptome characterization of gene expression under a given condition, thereby providing deeper knowledge of transcriptomic regulation. Although this approach has been successfully applied to study the whole transcriptome of bovine mammary glands under different conditions^12^,^13^,^14^,^15^, the effect of forage type on mammary gland metabolisms has not been extensively evaluated. It is well acknowledged that the expression of some genes is regulated both at the translational and post-transcriptional levels, hence proteome analysis is essential to fully understand the molecular mechanisms. To date, iTRAQ-based quantitative proteomics has improved protein identification coverage, thus providing more comprehensive linking of proteins to their metabolic function^16^. Integrating transcriptomic and quantitative proteomic analyses will promote a more complete understanding of molecular mechanisms underlying mammary gland adaptation to the alternative nutrient supplies provided by rations with different forage sources. To move towards this goal, we evaluate how the mammary transcriptome and proteome of dairy cattle consuming RS- and AH-based diets differed using coupled RNA-seq transcriptomics and iTRAQ proteomics.

## Results

### Summary of RNA-seq transcriptomic and quantitative proteomic analyses

A total of 19,656 transcripts were assembled from the clean data, and 554 genes (423 up-regulated and 131 down-regulated) were identified as significantly altered (1.5-fold change and *P* < 0.05) between RS and AH groups (Table S1). After removal of low-scoring spectra, 62,367 unique spectra were totally matched to 3,744 unique proteins, and 517 differentially changed proteins (231 increased and 286 decreased) were detected between RS- and AH-fed cows with 99% confidence (*P* < 0.05) and a 1.2-fold change cut-off (Table S1). Summaries of the differentially expressed genes and proteins are presented in Tables S2 and S3, respectively. Comparison between the transcriptome and proteome identified 17 significantly expressed genes in common at the mRNA and protein levels. Seven of the 17 proteins shifted in the same direction as their corresponding mRNAs (Table 1). The correlation between the significant transcript-protein pairs was the highest for the same expression patterns (r = 0.14; Fig. S1). Although the limited overlap of protein and mRNA was expected given that RNA-based analyses often fail to fully represent protein dynamics^17^, the results suggest that more targeted studies evaluating the overlap between mammary protein and mRNA dynamics are needed to evaluate the reliability of mRNA-based analyses of bovine mammary metabolism.

### Gene ontology analysis of differentially expressed transcripts and proteins

Comparison of Gene ontology (GO) enrichment at the mRNA and protein levels using GO annotation analysis found similarities in several GO terms (Table S4). The most abundant molecular function was binding activity at the mRNA level (79.9%) and protein level (46.6%). Another major functional category was catalytic activity, which was related to 32.5% and 30.6% of differentially expressed mRNA and protein, respectively. Notably, the GO term “structure activity” represented 3.5% of the transcriptome but none of the proteome (0%). The patterns of GO terms in cellular components between transcriptome and proteome were rather similar. This similarity is important because although the genes and proteins changing did not necessarily agree in the analyses, the conclusions drawn at the functional between RNA and protein-based evaluations would have minimal practical differences. The most frequently encountered biological processes in the transcriptome and proteome were cellular process (71.3% and 70.9%), metabolic process (53.4% and 50.5%), biological regulation (43.4% and 39.2%) and pigmentation (40.1% and 36.5%).

**Figure 1:**
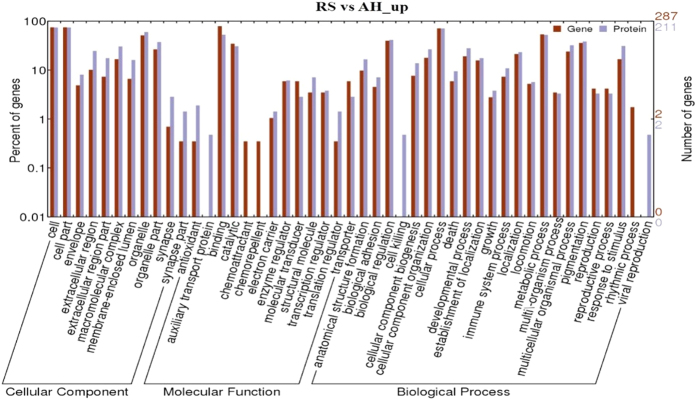
GO categories assigned to the differentially up-regulated transcripts and proteins in mammary glands between RS- and AH-fed dairy cows. The differentially expressed genes were classified into biological process, cellular component, and molecular function by WEGO according to the go terms. The right panel shows the gene number mapped to the go terms. The left panel shows the proportion of up-regulated genes in accordance with go terms.

**Figure 2:**
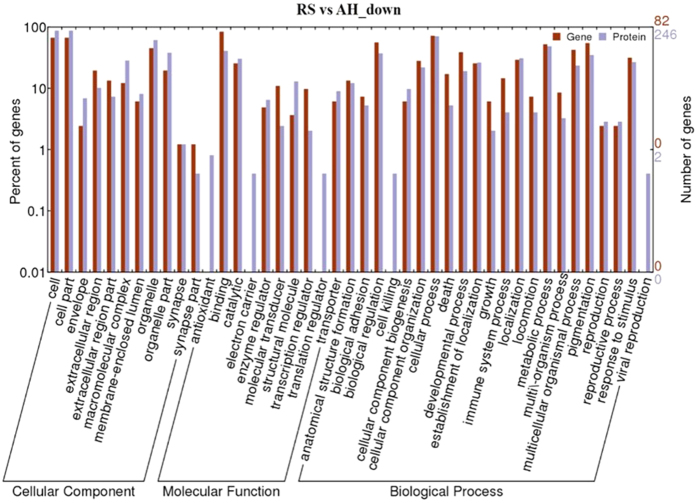
GO categories assigned to the differentially down-regulated transcripts and proteins in mammary glands between RS- and AH-fed dairy cows. The differentially expressed genes were classified into biological process, cellular component, and molecular function by WEGO according to the go terms. The right panel shows the gene number mapped to the go terms. The left panel shows the proportion of down-regulated genes in accordance with go terms.

Of those transcripts that were differentially upregulated, 287 out of 423 transcripts were annotated with 1,369 GO terms; 22.50% belonged to molecular functions, 14.17% belonged to cellular components and 63.33% belonged to biological processes (Table S5). Additionally, 82 down-regulated transcripts were assigned to 603 GO terms classified by molecular functions (21.23%), cellular components (10.28%) and biological processes (68.49%) (Table S6). The increased and decreased proteins were assigned to 2,845 (Table S7) and 2,698 GO terms (Table S8), respectively. A single gene could be annotated with anywhere from 1 to 17 terms. Among these GO terms, molecular functions accounted for 14.27% and 15.42% of the up- and down-regulated proteins, respectively. Cellular components accounted for 11.60% and 13.20% of the up- and down- proteins, respectively, and biological processes reflected the vast majority of the up- and down-regulated proteins (74.06% and 71.39%, respectively). The up-regulated (Fig. 1) and down-regulated (Fig. 2) transcripts and proteins assigned to each of the three main GO terms and secondary categories were presented in parallel, except for some minor differences, to better highlight the similarities in conclusions drawn from the two approaches.

### Function correlation analysis by the KEGG system

**Figure 3:**
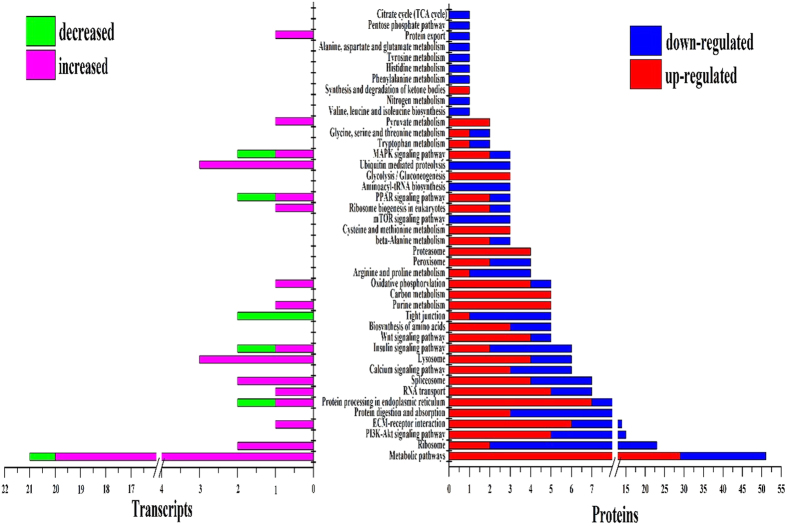
KEGG enriched pathways of differentially altered transcripts and proteins of the mammary glands between RS- and AH- fed dairy cows. The bottom panel shows the number of transcripts and proteins mapped to the pathway. The pink bars represent the enriched pathway of the increased transcripts, the green bars represent the enriched pathway of the decreased transcripts; the red bars represent the enriched pathway of the up-regulated proteins, and the blue bars represent the enriched pathway of the down-regulated transcripts.

In the Kyoto Encyclopedia of Genes and Genomes (KEGG) pathway analysis, 80 out of 423 increased transcripts and 30 out of 131 decreased transcripts were assigned to 61 pathways (Table S9). In the proteome data, 146 up-regulated and 192 down-regulated proteins were annotated to 203 pathways (Table S10). In all, 42 KEGG pathways were associated with milk production at the mRNA and protein levels (Fig. 3 and Table S11). Notably, although only 7 enriched proteins had corresponding changes in their mRNA expression levels, nearly 50% of the pathways mapped by enriched transcripts and proteins overlapped.

### Correlation between mRNA expression and protein abundance

**Figure 4:**
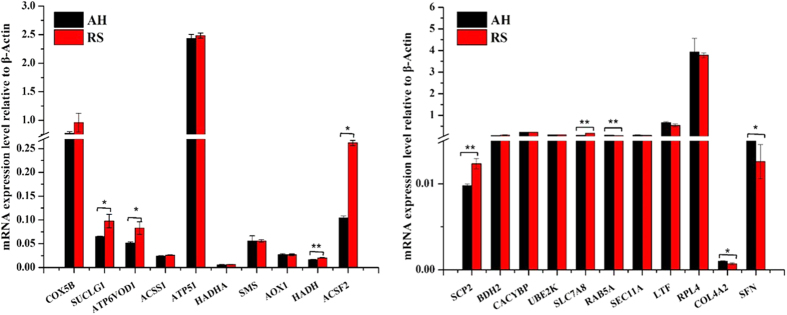
The qRT-PCR qualification of differentially changed transcripts related to milk metabolisms in mammary glands between RS- and AH-fed dairy cows. Relative mRNA expression levels were calculated according to the 2^−ΔΔCT^ method with β-actin as an internal reference gene. Error bars represent the standard deviation. **indicates that the difference in gene expression between RS and AH groups reached the extremely significant level, *P* < 0.01, and *indicates that the difference in gene expression between RS and AH groups reached the significant level, 0.01 < *P* < 0.05.

**Figure 5:**
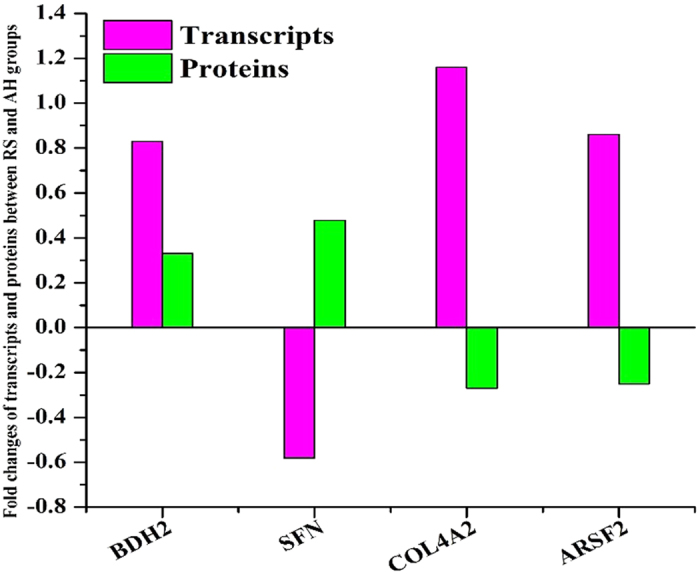
Correlation of the regulation patterns between transcripts and proteins in mammary glands between RS- and AH-fed dairy cows. The y axis positive direction represents the increased/up-regulated transcripts/proteins, and the y axis negative direction represents the decreased/down-regulated transcripts/proteins.

To examine the correlations between the mRNA and protein expression trends within the RS and AH groups, qRT-PCR was utilized. The 21 genes analysed were related to energy metabolism, amino acid/protein transport, protein degradation/synthesis, fatty acid/amino acid metabolism and mammary gland growth and development (Fig. S2 and Table S12). Among these genes, the expression levels of nine genes were significantly altered in the RS group compared to those in the AH group (Fig. 4 and Table S12). Of these nine genes, three genes acyl-CoA synthetase family member 2 (ACSF2), collagen, type IV, alpha 2 (COL4A2) and 14-3-3 protein sigma (SFN) had differentially expressed transcripts and proteins, but their expression patterns were opposite at the mRNA and protein levels (Fig. 5 and Table S12).

### Confirmation of protein abundance

**Figure 6:**
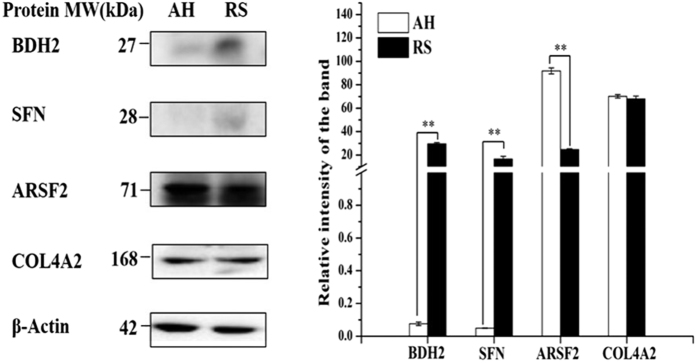
Protein abundance of significantly changed transcripts and proteins. Proteins were extracted from the mammary glands of RS and AH groups and separated by SDS-polyacrylamide gel electrophoresis and transferred onto PVDF membrane. β-Actin was used as internal control protein. The dilution of primary antibodies against BDH2, SFN, ARSF2 and COL4A2 and β-Actin were 1:500, 1:1000, 1:1000, 1:500 and 1:1000, separately. The relative intensities of bands were calculated using ImagePro Plus software. **indicates that the difference in gene expression between RS and AH reached the very significant level, *P* < 0.01, and *indicates that the difference in gene expression between RS and AH reached a significant level, 0.01 < *P* < 0.05.

For Western blot analysis (Fig. 6 and Table S12), four proteins that were up- or down-regulated at the transcript level were selected, including 3-hydroxybutyrate dehydrogenase type 2 (BDH2), SFN, ARSF2 and COL4A2. Compared with the RS group, protein abundances of ARSF2 and COL4A2 were lower than those in the AH group. In contrast, BDH2 and SFN protein levels were higher in the RS group than those in the AH group. These changes were also identified in the proteomic analysis; therefore, the Western blot analysis confirmed that the proteomic analysis was reliable.

### Relationship between the differentially changed transcripts/proteins and milk metabolism

**Figure 7:**
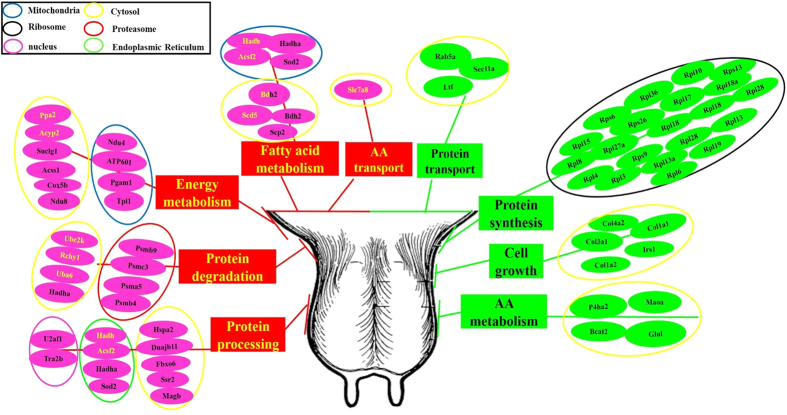
The whole view of the regulated units associated with molecular mechanisms of low milk production underlying rice straw forage. Color coding is as follows for items from the present study: black letters and pink background represents the up-regulated proteins, yellow letters and pink background represents increased transcripts, black letters and green background represents the down-regulated proteins. The half yellow/half black background represents genes that were both up-regulated at mRNA and protein levels.

A comprehensive view of the molecular mechanism underlying milk production was summarized based on the proteomic and transcriptomic data collected (Fig. 7 and Table S13). The regulatory subsections proposed include: energy metabolism, AA/fatty acid metabolism, protein degradation, protein synthesis, protein processing, AA/protein transport, and cell growth and development. Each of these regulatory functions is involved in regulation of milk metabolism, and the collective analysis shows how the mammary gland adapts to the low nutrient availability on the RS ration compared with the higher nutrient availability on the AH ration. The inconsistency in transcript- and protein-level responses identified here contrasts with the high agreement between analyses at the functional level, suggesting that transcriptomic analysis of the mammary gland might be sufficient to characterize tissue functional responses to altered states but might not always be reflective of shifts in specific proteins. Future work in this area is needed to better understand the relative utility of transcriptomic and proteomic analysis for understanding mammary gland metabolism.

## Discussion

Rice straw is one of the main roughages produced for ruminants in southern China, but its application is rather limited because this feed is poorly digested and low in protein content^4^. In a study by Wang *et al*.^6^, replacing AH with RS in the diet of lactating dairy cows as the forage source reduced nutrient digestibility (total-tract apparent digestibilities of DM (dry matter), OM (organic matter), NDF (neutral detergent fiber) and ADF (acid detergent fiber); *P* < 0.01). As a result of this depressed digestibility, rumen fermentation parameters (the ruminal total VFA concentration, including acetate (*P* = 0.02) and propionate (*P* = 0.04)) were also decreased when RS, rather than AH, was the primary forage source. With the limited digestibility and decreased fermentation associated with RS, it was not unexpected that lactation performance (yield of milk, ECM (energy-corrected milk), milk fat, milk protein, lactose and N conversion; *P* < 0.01) were decressed on the RS-based diet. To better characterize how the mammary gland responds to the limited nutrient availability of RS-based rations, this analysis integrated transcriptomic and proteomic patterns of mammary gland metabolism from dairy cows fed RS and AH rations. A total of 554 transcripts and 517 proteins were differentially expressed in mammary glands between the RS- and AH-fed animals. Of the 17 differentially expressed genes shared in the transcriptome and proteome, only seven genes displayed consistent expression at the mRNA and protein levels; the remaining ten genes demonstrated opposite expression (Table 1), which indicated a relatively modest concordance between transcriptome and proteome. The low global correlation between transcriptome and proteome (r = 0.05; Fig. S1A) further confirmed that the transcriptome was not well-corresponded to the proteome in this study. A series of studies have found it is common that transcriptome data are not always consistent with the corresponding proteome data due to potential post-modifications^18^,^19^,^20^. Although it is not uncommon to have poor correlation between mRNA and protein-based analyses, the differences here are important to consider because if the analysis had been done solely on mRNA or solely on protein, it is plausible that we might have reached different conclusions about how the mammary gland responds to RS. In this case, our objective was to understand the coordinated functional shifts in the mammary gland associated with the low nutrient availability of RS-based diets. Functional shifts were categorized primarily by mapping to GO terms and KEGG pathways. Although the differential expression of mRNA and proteins was not always in agreement, analysis of the GO terms overlapped by 80% suggesting that the conclusions at the functional level were similar between the analyses.

Among the detected genes and proteins in mammary gland, only a small proportion of them were overlapped between our current study and previous studies^13^,^15^,^21^,^22^,^23^,^24^. This could be possibly resulted from different species of cows used (American Holstein vs. China Holstein), different physiological stages (early lactation or dry period in other studies vs. mid-lactation in this study), different diets fed to cows (diet supplemented with varying fatty acid vs. diet supplemented with varying forages) or different methods for sampling tissues (biopsy in other studies vs. slaughter in this study). It is unlikely that these methodological differences compromised the results of our study because many genes previously reported to regulate lactation performance in cattle were detected in our tissue samples. For example, several ribosomal proteins (RPL23A, RPL4 and RPS6) have been associated with milk protein synthesis in mammary gland of lactating cows^13^,^24^ and the expression of SLC7A8 has been highly correlated to the leucine uptake into bovine mammary glands during lactation^24^. Additionally, Stearoyl-CoA desaturase (SCD) and 3-hydroxybutyrate dehydrogenase (BDH2) were involved in fatty acid synthesis and ketone body utilization, respectively in bovine lactating mammary gland^15^,^25^. Also, in our study the detected collagen proteins (COL1A1, COL4A2 and COL1A2) play major roles in the maintenance of organ morphology and function in mammary gland of healthy lactating cows^23^.

The interpretation of the findings from the present study has several limitations. For instance, only 60% of the mRNAs and approximately 80% of the proteins found in the bovine genome have functional annotations, and the significant level of KEGG enrichment analysis was set as *P*-value less than 0.1. In addition, despite the fact that the significant fold change of protein level were set lower at 1.2 with *P*-value < 0.05 (up-regulated) and 0.83 with *P*-value < 0.05 (down-regulated), the number of annotated genes shared at mRNA level and protein level was still only seven. Because of the better functional agreement between the mRNA and protein-based analyses we have selected to discuss the results based on functional conclusion.

Results appeared to support altered mammary gland ATP production associated with RS versus AH diets. Two up-regulated transcripts and a total of 8 out of 231 increased proteins (Fig. 7 and Table S13) and were related to ATP synthesis. Cytochrome c oxidase subunit 5b (COX5B), NADH dehydrogenase 8 (NDUFB8), NADH dehydrogenase 4 (NDUFB4) and V-type proton ATPase (ATP6V0D1) are essential enzymes involved in mitochondrial oxidative phosphorylation to provide ATP^26^. Cytochrome c oxidase (COX) is very important in determining the rate of oxygen consumption and thereby the rate of ATP synthesis; COX is also responsible for the reduction of molecular oxygen to water using reducing equivalents donated by cytochrome c and for site 3 energy coupling to the respiratory chain^27^,^28^. Additionally, acetyl-CoA synthetase 1 (ACSS1) and ARSF2 are involved in pyruvate metabolism which can promote ATP generation via the tricarboxylic acid cycle in mitochondria^29^. The upregulation of these factors related to ATP production suggests that dairy cows fed the RS-based diet appear to have enhanced ATP synthesis capacity. This is somewhat paradoxical because RS-based diets would be expected to have substantially reduced metabolizable energy compared with an AH diet^30^. As such, the reduced availability of energy substrate would be expected to limit the capacity for ATP generation. It is possible that the up-regulation of machinery associated with ATP generation in cows fed the RS diet represents some adaptation by the mammary gland to this low energy ration. This adaptation may involve up-regulating glucose uptake to procure more energy substrate for the mammary gland or increasing the activity and affinity of enzymes associated with ATP production to improve energy use efficiency within the mammary gland. Further research is needed to confirm this finding and better understand how and why ATP synthesis proteins in the mammary gland increase on rations with low energy availability.

Seven genes with increased abundance were involved in fatty acid metabolism, synthesis of ketone bodies and PPAR signalling (Fig. 7 and Table S13). This is logical given the different dietary conditions because breakdown of body stores for energy would be expected on the RS diet due to its low energy availability. Hydroxyacyl-CoA dehydrogenase (HADH) catalyses the third step of mitochondrial fatty acid β-oxidation, which converts the hydroxyl group into a keto group^31^. Cytosolic type BDH2 is involved in the cytosolic utilization of ketone bodies, which can subsequently enter mitochondria and the tricarboxylic acid cycle^32^. Notably, BDH2 displayed consistently up-regulated patterns at the mRNA and protein levels, suggesting that, as expected, ketone bodies were likely a primary energy source for the mammary gland on the RS diet. (SCD) plays a critical role in bovine mammary lipid metabolism and can maintain the fluidity of cell membranes and milk fat^33^. Increased SCD5 transcript also suggests enhanced fatty acid metabolism in mammary glands of the RS group. Sterol carrier protein-2 (SCP2), also called nonspecific lipid-transfer protein, is thought to be important in intracellular lipid trafficking and metabolism^34^, such as transporting cholesterol from intracellular sites (lipid droplets) to the mitochondria^35^. The protein SCP2 increased in abundance, indicating a higher level of fatty acid transport in the RS group. Overall, the up-regulated transcripts and proteins suggest that the mammary gland of RS-fed cows had potentially enhanced capacity to transport and use fatty acids as an energy source. The results collectively suggest enhanced mobilization of body fat to provide energy for milk production on the RS ration compared with the AH ration. This finding corresponds well to anticipated changes in energy availability of the RS versus AH rations.

Although the shifts in energy-related metabolism (ATP synthesis and fatty acid metabolism) involved only a small number of proteins and transcripts, ribosomal assembly and protein synthesis processes were associated with 21 of the 286 decreased proteins (Tables S11 and S13). Ribosomes consist of a small 40S subunit and a large 60S subunit, and ribosomal proteins are essential for ribosome assembly and optimal function^36^. Four ribosomal proteins (RPS6, RPS9, RPS13 and RPS26) are essential components of the 40S ribosomal subunit. RPS13 has been shown to bind to the 5.8S rRNA, and this complex affects the decoding activity of ribosomes^37^. RPS9 can interact with nucleolar protein and facilitate ribosome biogenesis^38^. RPS26 interacts with the 5′ untranslated region of mRNA and might play an important role in translation initiation^39^. Notably, the function of RPS6 is linked to mTOR, which exerts signalling effects associated with cell size, cell proliferation, and glucose homeostasis^40^. The remaining 16 ribosomal proteins are all indispensable components of the 60S ribosomal subunit. Ribosomal protein L10 (RPL10) is required in ribosome biogenesis and translational fidelity and is also a central controller of ribosome transit between non-rotated and rotated conformational states^41^. RPL13, RPL15 and RPL17 are important for maintaining ribosome viability^42^,^43^,^44^, and RL13A may have evolved from an essential ribosomal protein in lower eukaryotes to perform a dispensable ribosomal function in higher eukaryotes^45^. RPL18 functions as a molecular conveyor, allowing efficient uptake of cytosolic 5S rRNA molecules and maintenance of normal ribosome function^46^. The ribosomal proteins RPL19, RPL18A, RPL27A, RPL28 and RPL36 are indispensable in maintaining the integrity of the 80S ribosome^36^,^47^. Together, the lower abundance of all the aforementioned ribosomal proteins in the RS group likely indicates depressed capacity for protein translation. Although this should be corroborated with future and more targeted work, decreased protein translation would help explain why RS rations result in reduced milk protein production compared with AH rations^6^. Given the low protein content and poor digestibility of RS-based rations, it is likely that limited substrate availability is the primary cause for the decreased protein translation and poor milk protein production on RS-based rations. If this is the case, future work evaluating protein or key amino acid supplementation of rice straw based rations may help define feeding strategies to enhance efficiency of cattle consuming RS-based rations.

Altered expression of genes and abundance of proteins also suggested that shifts in protein degradation may be a part of the mammary responses to the low nutrient availability of RS-based rations. Three transcripts of increased abundance were involved in ubiquitin-proteasome-dependent proteolysis (Fig. 7; Tables S11 and S13). Additionally, three 20S core particles (PSMB4, PSMA5 and PSMB9) and one 26S regulatory subunit, PSMC3 (Fig. 7; Tables S11 and S13), had increased expression. In eukaryotes, the ubiquitin proteasome system (UPS) precisely regulates the cell cycle at key checkpoints by targeting cell cycle regulators for proteasome-mediated degradation^48^,^49^. The UPS requires the ubiquitin-activating enzyme (E1), the ubiquitin-conjugating enzyme (E2) and ubiquitin ligases (E3) to work together to facilitate ubiquitination of target proteins. Of the ubiquitin enzymes, E2 donates the ubiquitin from its Cys to the Lys of the target protein through E3-mediated specificity^50^. Ubiquitin-conjugating enzyme E2K (E2 enzyme) and ubiquitin-like modifier activating enzyme 6 (E1 enzyme) are integral components of the ubiquitin proteasome system. Overall, the abundance levels of the above proteolytic proteins coupled with the above identified changes in protein synthesis suggest that mammary capacity for milk protein output might be depressed in response to the low nutrient availability on the RS-based diet because protein synthesis machinery is downregulated and proteolysis responses appear to be enhanced.

This hypothesized adaptation to the RS-based ration is further supported by the observed shifts in proteins associated with amino acid metabolism (Fig. 7; Tables S11 and S13). Notably, amine oxidase (MOAO) was decreased on the RS-based ration. This protein plays an essential role in the metabolism of various amino acids (including phenylalanine, histidine, tyrosine, tryptophan, arginine, proline, glycine, serine and threonine metabolism) and catalyses the oxidative cleavage of alkyl-amines into aldehydes and ammonia^51^. Glutamine synthetase (GLUL), which catalyses ATP-dependent ligation of ammonia and glutamate to glutamine^52^, and BCAA aminotransferase 2 (BCAT2), which promotes branch chain amino acid degradation and increases the capacity of the mammary gland to transaminate BCAA, were also both downregulated^53^. When coupled with the indicators of depressed protein synthesis and enhanced protein degradation, the apparent shifts in protein responsible for AA metabolism further support the idea that the bovine mammary gland adapts to the low nutrient availability of RS-based diets by reducing capacity for milk protein synthesis.

The RS-based ration was also associated with changes in protein processing and secretion. In total, 10 proteins and three transcripts of increased abundance were involved in protein processing (protein folding, translocation and secretion) (Table S11; Fig. 7; Table S13). An additional three proteins with decreased abundance were involved in protein transport (Fig. 7 and Table S13). Protein secretion is the process of proteins moving across the endoplasmic reticulum (ER) membrane (site of post-translational modification)^54^ to the Golgi complex and eventually migrating to their final biological target. The first step of ER-to-Golgi transport is mediated by the coat protein II (COPII) complex, which consists of five core proteins: Sar1, Sec23, Sec24, Sec13 and Sec31^55^. Among these proteins, Sec24 is involved in cargo binding during vesicle budding and fusion^56^. Increased levels of Sec24D transcript identified in this study would be expected to promote translocation of proteins from the ER to the Golgi complex. This was not expected because the shifts in proteins regulating protein synthesis appear to suggest depressed protein generation and one would expect comparatively reduced protein translocation. Another apparently inconsistent finding was the upregulation of system L amino acid transporter 2 solute carrier family 7, SLC7A8. The protein is critical for the uptake of neutral amino acids such as leucine, and thereby activates mTOR signalling^57^. Up-regulating SLC7A8 would lead to increased leucine uptake into mammary gland in the RS group. Given the other shifts in protein suggest impaired protein synthesis and enhanced degradation, the upregulation of leucine uptake into the mammary is somewhat inconsistent. It is possible, given the expected low availability of dietary protein and the importance of leucine in milk protein synthesis^58^,^59^, that this upregulated Leucine uptake is an adaptation of the mammary gland to the low nutrient availability of the RS-based diet. Additional work evaluating the mammary gland responses to limited nutrient availability is needed to better understand what role protein translocation plays in the adaptation to poor quality diets.

Other shifts in proteins related to translocation and secretion agree better with the changes in protein modulating synthesis and degradation. As an ER molecular chaperone of the Hsp40 family, DnaJ homolog subfamily B member 11 (DNAJB11; also known as ERj3) binds to BiP, which is a major molecular chaperone involved in ER-associated degradation (ERAD), and then aggregates Hsp70 ATPases to stimulate ERAD. The up-regulated coexpression of DNAJB11 and the Hsp70 protein HSPA1 on the RS-based ration suggests that ERAD activity was enhanced in the RS group, which would further impair milk protein synthesis. Ras-related protein (Rab-5A) is primarily involved in the regulation of early endosome fusion and drives the maturation of endosomes by transporting vacuolar (H+)-ATPases (V-ATPases) from the trans-Golgi network to endocytic vesicles^60^. Most secretory proteins contain amino terminal or internal signal peptides that direct their sorting to the ER^61^. As one of the subunits of the signal peptidase complex, SEC11A is critical for secretory protein transport from the ER to either the extracellular space or the plasma membrane through the ER-Golgi secretory pathway^62^. Here, the decrease in Rab-5A and SEC11A proteins appears to suggest reduced protein transport and secretion to the outer membrane in mammary gland cells. This is in line with expected shifts in these biological processes given the apparent changes in protein synthesis and degradation.

A final category of shift associated with the RS-based ration is supported by the decreased abundance of four types of collagen proteins (COL4A2, COL1A1, COL3A2 and COL1A2) in the RS group (Fig. 7 and Table S13). Collagens are the main structural proteins in extracellular matrix, acting as an important regulator of the differentiated phenotype of mammary epithelial cells^63^. Furthermore, decreased expression of the above collagens suggests reduced cell growth in the mammary of cattle consuming RS-based rations. This suggests a lower metabolic activity of mammary tissue on RS-based rations and is likely related to the observed decreases in milk protein production^64^. In addition to depressed cellular growth, downregulation of COL4A2 identified in the RS-based ratio suggests a loss of basement membrane integrity, accompanied by an alteration of alveolar morphology characterized by decreased size and shrunken lumen containing little β-casein^63^. Although the data available in the present study do not enable us to determine whether these shifts are in response to the limited dietary substrate directly or a secondary response linked to reduced protein synthesis, further evaluation of the role of mammary structural proteins in adapting to poor quality diets may provide an alternative look at strategies for improving productivity of nutrient-restricted cattle^6^.

Gene Ontology is a useful means of understanding how genes are involved in cellular, tissue, or organismal processes^65^. The analysis of GO terms agreed with the above findings with some exceptions. Eight decreased transcripts of 82 annotated genes (Table S6) were involved in signal transduction and support the general impairment of normal mammary metabolism on the RS diet. An additional 59 up-regulated proteins (Table S7) were involved in response to stimulus, suggesting that RS feeding may generate some stress for dairy cows^6^. This is logical given the shifts in energy metabolism (increased expression of machinery involved in ATP production and enhanced fatty acid transport activity) because the molecular profiles suggest that RS cattle were in a negative energy balance and needed to mobilize body fat to provide sufficient energy for milk. Additionally, 174 of 246 annotated proteins with decreased abundance (Table S8) were implicated in molecular functions, demonstrating a substantial reduction in various biological reactions (protein synthesis) as a result of RS feeding. The high representation of catalytic activity and binding activity (protein binding, nucleotide binding, ATP/GTP binding and DNA/RNA binding) in the molecular function categories (Fig. 1 and Table S5) suggest that the RS group had strong metabolic activity and enhanced biological reactions in some aspects, which is not necessarily in agreement with the previously identified decreases in energy metabolism and protein synthesis. The GO terms related to protein transport were also inconsistent with the above findings because these terms were abundantly enriched (Tables S5 and S7), suggesting enhanced protein transport in RS-fed cows. Future work on a larger dataset of cattle and different types of nutrient restriction may help understand the biological drivers for some of the apparent inconsistencies in this analysis. Another critical factor here is that most transcripts and protein species were newly identified, suggesting the need to extend the proteome and transcriptome of the bovine mammary gland. Enhanced mapping of these resources will help to better understand the biological adaptions of cattle to varying environmental states.

KEGG is an integrated database resource for biological interpretation of genome sequences and other high-throughput data in cells or organisms^66^. The wide range of pathways enriched in the present study indicated that the normal function of the bovine mammary gland requires a wide repertoire of metabolic pathways working in concert (Fig. 3, Tables S9 and S10). Most of the transcript-mapped pathways overlapped with the protein-mapped pathways, suggesting that although expression is not always consistent between mRNA and protein-based analyses, mapping those analyses to larger pathways can result in consistent conclusions. Some of the pathways mapped by the increased proteins were involved in oxidative phosphorylation, pyruvate metabolism, and glycolysis/gluconeogenesis, suggesting enhanced energy demand in the RS-fed cow. The increased abundance of these proteins fuelled the upregulation of these important biological pathways associated with energy metabolism. In contrast, other proteins that increased in abundance were involved in the synthesis of ketone bodies and the PPAR signalling pathway. This suggests enhanced fatty acid metabolism, especially fatty acid β-oxidation, provided additional energy for the mammary gland when cattle consumed the RS diet which is in agreement with the differential expression discussed above.

Down-regulated proteins were enriched in various amino acid metabolic pathways, including that of phenylalanine, histidine, tyrosine, alanine, aspartate, and glutamate, indicating that the RS diet may have impaired amino acid metabolism (Table S10). However, functionally increased cysteine and methionine metabolism pathways within the RS group appear inconsistent with other findings and may denote that the biological reaction of cysteine and methionine was not negatively impacted by the RS diet. Proteomic data suggested that ribosomal proteins were substantially reduced on the RS treatment (Table S10). A large number of ribosomal proteins in the nucleus that were linked to the BIN decreased their abundance levels, which likely would disturb ribosome assembly and attenuate the ribosome activity^67^. Proteomic data also suggested that proteins responsible for protein processing in the ER were abundant (Fig. 3 and Table S10), suggesting an enhanced ERAD process. Another pathway, ubiquitin mediated proteolysis (Fig. 3), which directly induced protein degradation, was mapped to transcripts of decreased abundances. Moreover, four proteins of increased expression enriched in the pathway of proteasome, which functions with ubiquitin system to form a major protein degradative pathway in cell cycle^68^, providing further evidence for a high degree of proteolysis in the mammary on the RS treatment. Upregulated protein degradation and downregulated amino acid metabolism would reduce the protein reserve and further impair milk protein synthesis. The KEGG pathway-based analysis agrees with the above discussion of differential expression suggesting that a major component of mammary adaptation to the low nutrient availability of RS-based rations is reducing protein synthesis/processing and enhancing protein degradation.

The transcripts and proteins from the present study are summarized in Fig. 7, which gives a comprehensive view of the regulatory mechanisms underlying mammary responses to low nutrient availability on RS-based rations identified in this analysis. Four primary regulatory functions were apparent in mammary glands of RS-fed cattle compared with AH-fed cattle: energy/fatty acid metabolism; protein synthesis; protein processing/transport and degradation; and cell growth/development. Although only an initial snapshot of mammary responses to RS-based rations, the results here collectively suggest that inefficient utilization of amino acids for milk protein synthesis may contribute to the reduced milk production in the RS group. At the same time, the reduced energy concentration of this ration may have also affected the efficiency of milk and milk protein synthesis. Supplying amino acids is only the first step in protein synthesis; the subsequent post-translational modifications of proteins, protein processing, protein transport and extracellular secretion are all essential to protein production^69^, and portions of the process are energy-mediated^70^. This analysis revealed that all steps of protein synthesis appear to be affected by RS-based rations and further analysis is needed to better understand which of these changes are energy-mediated, which are due to limited AA availability, and which, if any, are associated with other driving factors.

## Conclusions

Milk is one of the most important products generated from the dairy industry, and forage is required during milk production. Through integrated transcriptomic and proteomic analyses, the results of this study identified four crucial biological processes that may contribute to the reduced milk synthesis observed in cows on RS diets. These factors included: (i) inefficient utilization of energy and enhanced fatty-acid metabolism, which were likely caused by an insufficient energy supply limiting normal protein production in dairy cows; (ii) reduced ribosomal activity and enhanced protein degradation, which likely increased the rate of proteolysis and impaired milk protein synthesis; (iii) increased protein processing in the ER and ER-associated protein degradation, which likely decreased protein transport and depressed the translocation of secretory proteins; and (iv) decreased abundance of transcripts/proteins related to cell growth/development, which impaired growth of the bovine mammary gland. These mechanisms involved in milk protein production of dairy cows on RS-forage-based diets can direct future work to better understand how the mammary gland adapts to low nutrient availability and eventually, how feeding strategies should be adapted to more efficiently utilize low-quality forage.

## Materials and Methods

### Animals, diets, experimental design and sample collection

All the experimental protocols were approved by the Animal Care Committee, Zhejiang University, Hangzhou, P. R. China, and all procedures, including animal handling prior to and after mammary gland biopsy in this study were in compliance with the Guidelines of China for Animal Care and conducted in accordance with the approved protocols. Twelve multiparous Holstein dairy cows (mean ± SD; body weight = 607 ± 55.6 kg, days in milk = 164 ± 24.8 day, and milk yield = 29.7 ± 4.7 kg/d) were assigned to 2 blocks balanced for milk yield and body weight, and then randomly assigned the RS and AH diets. The chemical composition of the individual forages and the experiment procedures were described by Wang *et al*.^6^. At the end of the experimental period (d 98), mammary tissue samples were collected immediately after slaughter according to previously described methods^71^. Approximately 50 g of tissue from each cow were snap-frozen in liquid nitrogen and stored at −80 °C for further analysis.

### RNA preparation, cDNA synthesis and RNA sequencing

Total RNA was extracted from samples with TRIzol (Invitrogen, CA, USA) and quantified using an Agilent Bioanalyzer 2100 and purified by a RNA 6000 Nano LabChip Kit (Agilent, CA, USA). With an RIN cut-off of 7.0, 5 μg of RNA was used for cDNA library construction using the Illumina RNA ligation-based method^72^. The average insert size for paired-end libraries was 400 bp (±50 bp), and paired-end sequencing was performed on an Illumina 2500 platform at LC Biotech (Hangzhou, China). Bovine reference genome sequences UMD3.1 (ftp://ftp.ensembl.org/pub/release-79/fasta/bos_taurus/dna/) were downloaded. After removing low-quality reads and adapter sequences, clean reads were aligned to the bovine reference genome using TopHat 2.0.9 (http://ccb.jhu.edu/software/tophat/index.shtml), ensuring reads were shorter than 20 bp and only 2 mismatches were allowed. Gene expression of the aligned reads was estimated using Cufflinks 2.1.1 (http://cole-trapnell-lab.github.io/cufflinks/)^73^, normalized by calculating the reads per kilobase per million mapped reads (FPKM) for each gene and annotated with NCBI genome assembly^74^. Genes with 1.5-fold changes and a *P*-value < 0.05 were considered significant.

### The qRT-PCR analysis

Extracted RNA was reverse transcribed for cDNA synthesis using a PrimeScript RT Reagent Kit (Takara, Tokyo, Japan), and qRT-PCR was performed on a 7500 Real-Time PCR instrument (Applied Biosystems, Foster City, CA) in a 20 μL reaction system as described by Li *et al*.^75^. The gene expression level was calculated relative to the β-actin gene according to the R = 2^−ΔΔCt^ method. The gene-specific primers were designed using Primer 5 (Premier Biosoft International, CA, USA) (Table S14). Three independent biological replicates were analysed for RS and AH groups.

### Protein preparation, digestion and iTRAQ labelling

To reduce sample variability, six replicates from each group were equally pooled into two samples as in previous studies^76^,^77^. Mammary tissues were ground into a fine powder in liquid nitrogen, and 150 mg from each sample were extracted with lysis buffer (7 M urea, 2 M thiourea, 4% CHAPS, 40 mM Tris-HCl, pH 8.5) supplemented with protease inhibitor (Roche Applied Science, Mannheim, Germany). After homogenization on ice for 15 min and centrifugation at 12,000× g at 4 °C for 15 min, the supernatants were kept at −80 °C until iTRAQ and Western blotting analysis.

Supernatants containing 150 μg of proteins were digested with Trypsin Gold (Promega, Madison, USA) at a 30:1 protein: trypsin ratio at 37 °C for 16 h. The peptides were dried using vacuum-centrifugation, reconstituted in 0.5 M TEAB (Applied Biosystems, Milan, Italy) and processed according to the manufacturer’s protocol for 6-plex iTRAQ Reagent kit. In the labelling reaction, samples were incubated for 2 h at room temperature, pooled and dried using vacuum-centrifugation.

### Strong cation-exchange and LC-ESI-MS/MS analysis

Strong cation-exchange (SCX) chromatography was performed with a LC-20AB HPLC pump system (Shimadzu, Kyoto, Japan). The iTRAQ-labelled peptide mixtures were reconstituted with 4 mL of buffer A (25 mM NaH_2_PO_4_ in 25% ACN, pH 2.7) and loaded onto a 4.6 × 250-mm Ultremex SCX column containing 5-μm particles (Phenomenex, Torrance, USA). Buffer B consisted of 25 mM NaH_2_PO_4_ and 1 M KCl in 25% ACN (pH 2.7). Peptide elution procedures were conducted following the procedure of Yan *et al*.

Each fraction was resuspended in buffer C (2% (v/v) ACN and 0.1% (v/v) FA in MilliQ water) and centrifuged at 20,000× g for 10 min. Next, 10 μL of peptide (0.5 μg/μL) were loaded onto a C18 trap column (2 cm × 100 μm, 5 μm) in a LC-20AD nano-HPLC system (Shimadzu, Kyoto, Japan) by auto-sampler. The samples were loaded at 8 μL/min for 4 min, then run on a 44 min gradient increasing from 2% to 35% buffer D (98% ACN (v/v) and 0.1% FA (v/v)) at 300 nL/min, followed by a 2 min linear gradient to 80% buffer D, maintained at 80% buffer D for 4 min, and finally returned to 5% buffer D for 1 min. Peptide analysis was performed on a Q-Exactive mass spectrometer (Thermo Fisher Scientific, San Jose, CA) in positive ion mode with a selected mass range of 350–2000 mass/charge (m/z) for a full MS scan and 100–1800 m/z for MS_2_ scans. Intact peptides were detected in the Orbitrap at a resolution of 70,000. Peptides were selected for MS/MS using high-energy collision dissociation (HCD) with a normalized collision energy setting of 27.0; ion fragments were detected in the Orbitrap at a resolution of 17,500. Resolving power for the Q-Exactive was set as 70,000 for the MS scan and 17,500 for the MS/MS scan at m/z 200. The electrospray voltage was set to 1.6 KV and MS/MS data were acquired using the 15 most abundant precursor ions above a threshold ion count of 20,000 in the MS survey scan with a dynamic exclusion duration of 15 s. The automatic gain control target values for full MS were 3^e^6 and 1^e^5 for MS_2_. The underfill ratio was defined as 0.1% on the Q-Exactive.

### Protein identification and quantification

Raw files were merged and transformed to a MGF file by Proteome Discoverer (ver. 1.2; Thermo Fisher Scientific, San Jose, CA) and then probed using the Mascot search engine (ver. 2.3.02; Matrix Science, London, UK). The species database was an in-house UniProt bovine (*Bos taurus*) database with 31,661 entries. For protein identification, the parameters were as follows: monoisotopic mass, trypsin digestion allowing up to two missed cleavages, MS/MS ion search, fragment mass tolerance at 0.02 Da, and peptide mass tolerance at ±10 ppm. Fixed modifications were carbamidomethylation of cysteine, iTRAQ 6-plex (N-term), and iTRAQ 6-plex (K). Potential variable modifications were Gln- > pyro-Glu (N-term Q), oxidation (M), or deamidation (NQ). The decoy database pattern was the reverse of the target database. The probability of false peptide identification was reduced by only counting peptides at the 99% confidence level. Protein identification required at least one unique peptide identification and at least two unique spectra. Relative quantification of identified proteins was estimated according to the weighted and normalized ratios of uniquely identified peptides using the median ratio in Mascot. Differential expression of proteins was determined using Fisher’s test. Proteins with a 1.2-fold change and a *P*-value < 0.05 were defined as differentially expressed.

### Bioinformatics analysis

Functional annotation of the identified transcripts and proteins was conducted using the Blast2GO program with the non-redundant gene/protein database (NR; NCBI). Analyses of the annotated functions of identified transcripts/proteins were performed using GO annotation software (ftp://ftp.ncbi.nih.gov/gene/DATA/gene2go.gz). Metabolic pathway analysis of the identified proteins was conducted using the KEGG Pathway Database (http://www.genome.jp/kegg). Pathway enrichment statistical analyses were performed using Fisher’s exact test. A corrected *P*-value of 0.05 defined significance.

### Western blot analysis

The mammary tissues from each cow were homogenized in an ice-cold RIPA buffer (radio immunoprecipitation assay; Beyotime, Shanghai, China) containing 1 mmol/L PMSF (for 30 min at room tempeture), 1% (v/v) protease, and a phosphatase inhibitor cocktail (Thermo Fisher Scientific, San Jose, CA, USA). The mammary cell lysates were centrifuged at 2000× g for 5 min and the supernants were transferred to new tubes. The protein concentrations of the samples were determined by BCA assay kits (bicinchoninic acid; Beyotime, shanghai, China). Approximately 40 μg of extracted protein per sample was separated using 12% SDS-PAGE. Proteins were subsequently transferred onto 0.45-μm PVDF membranes (IPVH00010, Millipore) and blocked with blocking buffer (Beyotime, Jiangsu, China) for 2 h at room temperature. The membranes were incubated with primary antibodies (diluted 1:1000 in PBS (phosphate-buffered saline)) against BDH2 (ab87208; Abcam, Cambridge, MA, USA), anti-SFN (ab87209, Abcam), anti-ARSF2 (ab86178, Abcam) and anti-COL4A2 (sc-70243; Santa Cruz biotechnology, Cambridge, MA, USA) and anti-β-actin (AF0003; Beyotime, Jiangsu, China) overnight at 4 °C. After washing with TBST (Tris-buffered saline containing 0.02% (v/v) Tween-20) for three times, the membranes were then incubated with secondary antibodies of goat anti-rabbit IgG or goat anti-mouse IgG conjugated with horseradish peroxidase diluted at 1:5000 in PBS (Beyotime, Jiangsu, China) for 2 h at room temperature, incubated with ECL Western Blotting Substrate Kits (Beyotime, Jiangsu, China). The protein bands were visualized using a chemiluminescence system (CLiNX Science 158 Instrument, Shanghai, China) and analyzed with ImagePro Plus 6.0 software (Media Cybernetics, Washington, MD, USA) using β-actin as the reference protein. Differences among groups were compared using one-way ANOVA, and *P*-value < 0.05 was considered significant.

## Additional Information

**How to cite this article**: Dai, W. *et al*. Complementary transcriptomic and proteomic analyses reveal regulatory mechanisms of milk protein production in dairy cows consuming different forages. *Sci. Rep.* **7**, 44234; doi: 10.1038/srep44234 (2017).

**Publisher's note:** Springer Nature remains neutral with regard to jurisdictional claims in published maps and institutional affiliations.

## Supplementary Material

Supplementary Information

## Figures and Tables

**Table 1:** Differentially expressed transcripts and their corresponding proteins in mammary glands between RS- and AH-fed dairy cows.

Gene ID	Gene annotation	Fold RS/AH in transcripts	P-value (transcripts)	Regulation in transcriptome	Protein ID	Fold RS/AH in proteins	P-value (proteins)	Regulation in proteome	Protein annotation
XLOC_005548	CALB1	14.01	5.00E-05	up	IPI00703547	1.47	0.0009	up	Calbindin
XLOC_023621	SLC30A9	4.00	0.0003	up	IPI00714898	0.75	0.0175	down	Solute carrier family 30 (zinc transporter), member 9-like
XLOC_023813	MFSD10	3.62	0.0203	up	IPI00697349	0.82	0.0090	down	Major facilitator superfamily domain-containing protein 10
XLOC_011437	KRT15	2.88	0.0010	up	IPI00692588	2.68	6.70E-12	up	Keratin 15
XLOC_023975	HERC6	2.87	0.0059	up	IPI00825680	0.81	2.39E-06	down	Hect domain and RLD 6
XLOC_023465	CAMK2D	2.58	0.0091	up	IPI00688651	1.48	0.0397	up	Calcium/calmodulin-dependent protein kinase type II subunit delta
XLOC_024273	CTBP1	2.41	0.0088	up	IPI00690446	1.28	0.0158	up	C-terminal binding protein 1
XLOC_003206	DYSF	2.19	0.0152	up	IPI00843305	0.76	2.39E-14	down	Dysferlin
XLOC_004006	COL4A2	2.16	0.0136	up	IPI00712524	0.78	0.032584	down	Collagen, type IV, alpha 2, partial
XLOC_024070	OCIAD2	1.99	0.0233	up	IPI00694581	0.82	0.0025	down	OCIA domain-containing protein 2
XLOC_012858	ALPL	1.98	0.0162	up	IPI00702768	0.79	0.0053	down	Alkaline phosphatase, tissue-nonspecific isozyme
XLOC_023745	SEPTIN 11	1.87	0.0325	up	IPI00707718	1.33	0.0037	up	Septin-11
XLOC_011326	ARSF2	1.86	0.0254	up	IPI00717758	0.75	0.0001	down	Acyl-CoA synthetase family member 2, mitochondrial
XLOC_023501	BDH2	1.83	0.0480	up	IPI00694312	1.33	0.0012	up	3-hydroxybutyrate dehydrogenase type 2
XLOC_025920	PALLD	0.53	0.0464	down	IPI00689005	1.57	0.0026	up	Palladin, cytoskeletal associated protein
XLOC_012793	SFN	0.42	0.0445	down	IPI00715354	1.478	0.0028	up	14-3-3 protein sigma
XLOC_004450	AKR1C4	0.07	0.0002	down	IPI00704078	0.774	0.0002	down	Dihydrodiol dehydrogenase 3
