# Supplementary material for: Complementary transcriptomic and proteomic analyses reveal regulatory mechanisms of milk protein production in dairy cows consuming different forages
Source: Sci Rep. 2017 Mar 14;7:44234. doi: 10.1038/srep44234 (PMC5349593; doi:10.1038/srep44234)
Supplement: Supplementary Information [file srep44234-s1.pdf]

Complementary transcriptomic and proteomic analyses reveal regulatory mechanisms of milk protein production in dairy cows consuming different forages

Wenting Dai[1], Qiong Chen[1], Quanjuan Wang[1], Robin Rosemarie White[2], Jianxin Liu[1], Hongyun Liu[1*]

[1] Institute of Dairy Science, College of Animal Sciences, Zhejiang University, Hangzhou 310058, P.R. China

[2] Department of Dairy Science, Virginia Tech, Blacksburg 24060, United States

[*] Correspondence author:

Dr. Hongyun Liu: Tel 0571-88982965； Fax 0571-88982930； E-mail: hyliu@zju.edu.cn

Table S1 Summary of transcripts and proteins detected in mammary glands between RS- and AH-fed dairy cows

Items	Transcripts	Proteins
Unique genes/proteins detected	19,656	3,744
Significantly changed genes/proteins	554	517
Up-regulated genes/proteins	423	231
Down-regulated genes/proteins	131	286
Shared genes	17	

Table S2 The differentially expressed transcripts in mammary glands between RS- and AH-fed dairy cows

**Gene id**	**Gene**	**Gene annotation**	**RS /AH**	***P*** **-value**	**Regulation**
XLOC_000144	FAM55C	Neurexophilin and PC-esterase domain family, member 3 (NXPE3), with a synonym of FAM55C	2.21	0.0158	up
XLOC_000212	-		NA	5.00E-05	up
XLOC_000596	UBL5	Ubiquitin-like protein 5	3.30	0.0264	up
XLOC_000924	-		NA	0.0001	up
XLOC_001060	BT.52938	Serotransferrin-like	3.23	0.0219	up
XLOC_001311	SLC7A8	Solute carrier family 7 (amino acid transporter light chain, L system), member 8 (SLC7A8)	2.22	0.0067	up
XLOC_001362	RNASE1	Ribonuclease, RNase A family, 1 (pancreatic)	5.22	5.00E-05	up
XLOC_001369	-		NA	0.0003	up
XLOC_001463	SNAP23	Synaptosomal-associated protein, 23kDa	1.89	0.0323	up
XLOC_001756	-		NA	0.0002	up
XLOC_001806	BT.90608	Uncharacterized protein	2.12	0.0211	up
XLOC_002163	-		NA	5.00E-05	up
XLOC_002246	-		NA	0.0004	up
XLOC_002356	-		NA	5.00E-05	up
XLOC_002497	UNC50	Unc-50 homolog (C. elegans)	1.98	0.0404	up
XLOC_002509	BT.55456	Uncharacterized protein	2.44	0.0126	up
XLOC_002550	BOLA3	BolA homolog 3 (E. coli)	2.03	0.0424	up
XLOC_002589	BT.89253	Uncharacterized protein	2.08	0.0235	up
XLOC_002593	CRIM1	Cysteine rich transmembrane BMP regulator 1 (chordin-like)	1.90	0.0416	up
XLOC_002602	FAM82A1	Regulator of microtubule dynamics 2 (RMDN2), synonyms of FAM82A1	2.29	0.0492	up
XLOC_002615	RPL23A	60S ribosomal protein L23a	5.00	0.0173	up
XLOC_002658	ACYP2	Acylphosphatase 2, muscle type	2.49	0.0464	up
XLOC_002711	KRCC1	Lysine-rich coiled-coil 1	1.94	0.0192	up
XLOC_002772	ARHGAP25	Rho GTPase activating protein 25	2.23	0.0470	up
XLOC_002834	-		NA	5.00E-05	up
XLOC_002847	HS1BP3	HCLS1 binding protein 3	1.90	0.0485	up
XLOC_002857	RDH14	Retinol dehydrogenase 14 (all-trans/9-cis/11-cis)	1.80	0.0472	up
XLOC_002935	RABEPK	Rab9 effector protein with kelch motifs	3.66	0.0256	up
XLOC_002956	ENG	Endoglin	2.09	0.0250	up
XLOC_002962	COQ4	Coenzyme Q4	2.11	0.0355	up
XLOC_002973	PKN3	Protein kinase N3	2.56	0.0301	up
XLOC_003001	NUP214	Nucleoporin 214kDa	1.91	0.0269	up
XLOC_003064	-		NA	0.0003	up
XLOC_003068	TMEM203	Transmembrane protein 203	2.47	0.0361	up
XLOC_003080	ABCA2	ATP-binding cassette, sub-family A (ABC1), member 2	2.35	0.0291	up
XLOC_003103	ZC3H6	Znc finger CCCH-type containing 6	2.15	0.0467	up
XLOC_003107	MERTK	C-mer proto-oncogene tyrosine kinase	2.72	0.0157	up
XLOC_003148	AFF3	AF4/FMR2 family, member 3	2.44	0.0341	up
XLOC_003157	BT.52969	Uncharacterized protein	5.16	0.0005	up
XLOC_003163	-		NA	0.0003	up
XLOC_003180	INO80B	INO80 complex subunit B	2.02	0.0230	up
XLOC_003206	DYSF	Dysferlin	2.19	0.0152	up
XLOC_003207	ZNF638	Zinc finger protein 638	2.09	0.0133	up
XLOC_003268	SRBD1	S1 RNA binding domain 1	2.23	0.0303	up
XLOC_003272	MCFD2	Multiple coagulation factor deficiency 2	1.75	0.0477	up
XLOC_003316	MRPL19	Mitochondrial ribosomal protein L19	2.22	0.0197	up
XLOC_003322	GCC2	GRIP and coiled-coil domain containing 2	1.78	0.0374	up
XLOC_003350	KDM3A	Lysine (K)-specific demethylase 3A	1.79	0.0458	up

XLOC_003464	BT.29369	Uncharacterized protein	2.16	0.0085	up
XLOC_003605	ENG	Endoglin	2.38	0.0060	up
XLOC_003625	ZER1	Zyg-11 related, cell cycle regulator	1.98	0.0308	up
XLOC_003664	-		NA	5.00E-05	up
XLOC_003674	DNLZ	DNL-type zinc finger	2.10	0.0264	up
XLOC_003678	INPP5E	Inositol polyphosphate-5-phosphatase, 72 kDa	3.60	0.0480	up
XLOC_003697	SARDH	Sarcosine dehydrogenase	2.70	0.0252	up
XLOC_003706	MRPL41	Mitochondrial ribosomal protein L41	2.10	0.0160	up
XLOC_003747	PPP1R26	Protein phosphatase 1, regulatory subunit 26	2.14	0.0186	up
XLOC_003749	COL5A1	Collagen, type V, alpha 1	1.91	0.0364	up
XLOC_004364	-		NA	0.0002	up
XLOC_004385	SLC39A12	Solute carrier family 39 (zinc transporter), member 12	3.23	0.0080	up
XLOC_004705	-		NA	5.00E-05	up
XLOC_004756	SNORD12	Small nucleolar	3.32	0.0111	up
XLOC_004967	-		NA	5.00E-05	up
XLOC_005485	-		3.72	0.0172	up
XLOC_005548	CALB1	Calbindin 1, 28kDa	14.01	5.00E-05	up
XLOC_006678	ENSBTAG00000009359	Uncharacterized protein	2.00	0.0263	up
XLOC_006775	-		NA	5.00E-05	up
XLOC_006776	-		NA	0.0003	up
XLOC_006959	-		NA	0.0002	up
XLOC_007550	SLC45A3	Solute carrier family 45, member 3	1.91	0.0347	up
XLOC_007791	-		NA	0.0001	up
XLOC_007927	-		NA	0.0002	up
XLOC_008015	-		NA	5.00E-05	up
XLOC_008078	-		NA	5.00E-05	up
XLOC_008198	-		NA	5.00E-05	up
XLOC_008544	ENSBTAG00000032433	Uncharacterized protein	13.92	5.00E-05	up
XLOC_008732	-		NA	5.00E-05	up
XLOC_008893	ADCY7	Adenylate cyclase 7	2.04	0.0230	up
XLOC_009270	GLTSCR1	Glioma tumor suppressor candidate region gene 1	1.90	0.0489	up
XLOC_009283	-		NA	0.0003	up
XLOC_009333	BT.12647	Uncharacterized protein	2.19	0.0078	up
XLOC_009715	-		NA	0.0001	up
XLOC_009716	-		NA	0.0008	up
XLOC_009917	ZNF285	Uncharacterized protein	2.73	0.0179	up
XLOC_010523	NME1	Non-metastatic cells 1, protein (NM23A) expressed in (NME1), transcript variant 2	1.84	0.0457	up
XLOC_004006	COL4A2	Collagen, type IV, alpha 2	2.16	0.0136	up
XLOC_010746	SECTM1	Secreted and transmembrane 1	5.93	5.00E-05	up
XLOC_011222	PER1	Period circadian clock 1	2.06	0.0152	up
XLOC_011326	ACSF2	Acyl-CoA synthetase family member 2	1.86	0.0254	up
XLOC_011437	KRT15	Uncharacterized protein	2.88	0.0010	up
XLOC_011982	AOX1	Aldehyde oxidase 1	1.95	0.0285	up
XLOC_012182	SNRNP40	Small nuclear ribonucleoprotein 40kDa (U5)	1.94	0.0308	up
XLOC_012264	ID3	Inhibitor of DNA binding 3, dominant negative helix-loop-helix protein	2.36	0.0134	up
XLOC_012279	HSPG2	Heparan sulfate proteoglycan 2	1.87	0.0361	up
XLOC_012403	ENSBTAG00000002009	Uncharacterized protein	NA	0.0003	up
XLOC_012452	BT.17865	Nitric oxide synthase trafficker	2.02	0.0193	up
XLOC_012654	-		NA	5.00E-05	up
XLOC_012821	-		NA	0.0002	up
XLOC_012858	ALPL	Alkaline phosphatase, liver/bone/kidney	1.98	0.0162	up
XLOC_013208	-		5.15	0.0278	up
XLOC_013257	FST	Follistatin	2.06	0.0484	up
XLOC_013393	-		NA	0.0025	up

XLOC_013487	-		NA	5.00E-05	up
XLOC_013557	FBXO22	F-box protein 22	15.45	5.00E-05	up
XLOC_013602	NPAS3	Neuronal PAS domain protein 3	3.74	0.0110	up
XLOC_014862	-		NA	0.0002	up
XLOC_014863	-		NA	0.0002	up
XLOC_014944	-		NA	0.0003	up
XLOC_015121	-		NA	5.00E-05	up
XLOC_015126	NOTCH4	Notch 4	1.82	0.0460	up
XLOC_015255	BT.88561	Uncharacterized protein	4.23	0.0111	up
XLOC_015261	H4	Histone 4	NA	0.0008	up
XLOC_015335	BT.38575	Uncharacterized protein	2.56	0.0260	up
XLOC_015530	GTPBP2	GTP binding protein 2	1.83	0.0329	up
XLOC_015580	BOLA-DQB	Major histocompatibility complex, class II, DQ beta	2.01	0.0261	up
XLOC_015912	-		NA	0.0001	up
XLOC_016324	EME2	Uncharacterized protein	2.49	0.0431	up
XLOC_016594	BT.45558	Uncharacterized protein	2.60	0.0225	up
XLOC_016622	RASA4	RAS p21 protein activator 4B	2.41	0.0082	up
XLOC_016634	-		NA	0.0001	up
XLOC_016819	-		NA	0.0001	up
XLOC_016820	BT.45760	Uncharacterized protein	4.57	0.0003	up
XLOC_016821	BT.106170	Uncharacterized protein	5.47	5.00E-05	up
XLOC_017003	-		NA	5.00E-05	up
XLOC_017018	PSPH	Phosphoserine phosphatase	2.28	0.0074	up
XLOC_017228	ENSBTAG00000046100	Uncharacterized protein	7.30	0.0031	up
XLOC_017321	-		NA	0.0028	up
XLOC_017359	TECTB	Tectorin beta	1.95	0.0410	up
XLOC_017920	BT.45696	Uncharacterized protein	1.90	0.0398	up
XLOC_018228	EGLN1	Egl-9 family hypoxia-inducible factor 1	2.08	0.0253	up
XLOC_018858	-		NA	5.00E-05	up
XLOC_018924	ROBO4	Roundabout homolog 4, magic roundabout (Drosophila)	1.97	0.0229	up
XLOC_019107	-		NA	5.00E-05	up
XLOC_019109	-		NA	5.00E-05	up
XLOC_019300	ENSBTAG00000011511	Uncharacterized protein	2.12	0.0319	up
XLOC_019503	-		NA	5.00E-05	up
XLOC_019504	-		NA	5.00E-05	up
XLOC_019671	-		NA	0.0003	up
XLOC_020095	PPOX	Protoporphyrinogen oxidase	1.90	0.0460	up
XLOC_020155	-		NA	0.0001	up
XLOC_020166	-		NA	0.0003	up
XLOC_020201	-		NA	5.00E-05	up
XLOC_020267	ECM1	Extracellular matrix protein 1	2.12	0.0126	up
XLOC_020616	-		2.43	0.0099	up
XLOC_020670	C1ORF84	Chromosome 1 open reading frame 84	2.07	0.0374	up
XLOC_020698	CTPS	CTP synthase	2.00	0.0138	up
XLOC_020726	ZC3H12A	Zinc finger CCCH-type containing 12A	2.41	0.0220	up
XLOC_020890	BT.99652	Uncharacterized protein	2.19	0.0433	up
XLOC_021122	ENSBTAG00000047361	Uncharacterized protein	2.10	0.0155	up
XLOC_021577	RAMP3	Receptor (G protein-coupled) activity modifying protein 3	2.82	0.0413	up
XLOC_021743	-		NA	0.0002	up
XLOC_021864	METAP2	Methionyl aminopeptidase 2	1.80	0.0339	up
XLOC_021996	ASB8	Ankyrin repeat and SOCS box containing 8	2.00	0.0239	up
XLOC_022209	-		NA	5.00E-05	up
XLOC_022367	-		NA	0.0003	up
XLOC_022472	CECR2	Cat eye syndrome chromosome region, candidate 2	2.37	0.0191	up
XLOC_022513	-		NA	5.00E-05	up
XLOC_022630	ENSBTAG00000038351	Uncharacterized protein	3.47	0.0087	up

XLOC_022707	-		NA	5.00E-05	up
XLOC_022708	-		NA	5.00E-05	up
XLOC_022789	-		NA	0.0002	up
XLOC_022807	ENSBTAG00000032428	Olfactory receptor 10AD1-like	2.08	0.0484	up
XLOC_022812	C5H12orf68	Chromosome 5 open reading frame 6	5.15	5.00E-05	up
XLOC_022857	MDM2	MDM2 proto-oncogene, E3 ubiquitin protein ligase	2.02	0.0181	up
XLOC_023217	PTMS	Parathymosin	2.02	0.0201	up
XLOC_023297	PDGFB	Platelet-derived growth factor beta polypeptide	2.29	0.0111	up
XLOC_023396	IL17REL	Interleukin 17 receptor E-like	2.06	0.0366	up
XLOC_023423	-		7.70	0.0361	up
XLOC_023428	NAF1	Nuclear assembly factor 1 ribonucleoprotein	2.19	0.0175	up
XLOC_023432	ANXA5	Annexin A5	2.51	0.0197	up
XLOC_023435	BT.61452	Uncharacterized protein	2.99	0.0191	up
XLOC_023439	BT.56334	Uncharacterized protein	3.46	0.0267	up
XLOC_023444	PDE5A	Phosphodiesterase 5A, cGMP-specific	2.31	0.0228	up
XLOC_023446	C4ORF3	Chromosome 4 open reading frame 3	2.88	0.0468	up
XLOC_023448	SEC24D	SEC24 family member D	2.03	0.0256	up
XLOC_023465	CAMK2D	Calcium/calmodulin-dependent protein kinase II delta	2.58	0.0091	up
XLOC_023472	TIFA	TRAF-interacting protein with forkhead-associated domain	2.01	0.0324	up
XLOC_023480	PLA2G12A	Phospholipase A2, group XIIA	2.53	0.0019	up
XLOC_023481	CASP6	Caspase 6, apoptosis-related cysteine peptidase	3.08	0.0003	up
XLOC_023491	TBCK	TBC1 domain containing kinase	2.26	0.0315	up
XLOC_023494	INTS12	Integrator complex subunit 12	3.08	0.0031	up
XLOC_023495	PPA2	Pyrophosphatase (inorganic) 2	2.04	0.0368	up
XLOC_023501	BDH2	3-hydroxybutyrate dehydrogenase, type 2	1.83	0.0480	up
XLOC_023509	MANBA	Mannosidase, beta A, lysosomal	2.07	0.0388	up
XLOC_023510	SLC39A8	Solute carrier family 39 (zinc transporter), member 8	2.66	0.0195	up
XLOC_023512	PPP3CA	Protein phosphatase 3, catalytic subunit, alpha isozyme	2.68	0.0040	up
XLOC_023513	EMCN	Endomucin	2.19	0.0153	up
XLOC_023518	H2AFZ	H2A histone family, member Z	2.13	0.0107	up
XLOC_023519	DNAJB14	DnaJ (Hsp40) homolog, subfamily B, member 14	2.68	0.0163	up
XLOC_023520	LAMTOR3	Late endosomal/lysosomal adaptor, MAPK and MTOR activator 3	2.44	0.0024	up
XLOC_023522	RG9MTD2	tRNA methyltransferase 10 homolog A (S. cerevisiae)	3.47	0.0057	up
XLOC_023529	ADH5	Alcohol dehydrogenase 5 (class III), chi polypeptide	2.33	0.0063	up
XLOC_023531	EIF4E	Eukaryotic translation initiation factor 4E	2.41	0.0034	up
XLOC_023532	TSPAN5	Tetraspanin 5	3.07	0.0436	up
XLOC_023550	FAM13A	Family with sequence similarity 13, member A	2.49	0.0225	up
XLOC_023552	BT.67700	Uncharacterized protein	2.03	0.0141	up
XLOC_023553	PPM1K	Protein phosphatase, Mg2+/Mn2+ dependent, 1K	3.87	0.0001	up
XLOC_023555	LAP3	Leucine aminopeptidase 3	2.95	0.0010	up
XLOC_023558	SLIT2	Slit homolog 2 (Drosophila)	3.44	0.0056	up
XLOC_023564	ECSOD	Superoxide dismutase 3, extracellular	2.75	0.0040	up
XLOC_023565	-		NA	5.00E-05	up
XLOC_023566	BT.25341	Phosphatidylinositol 4-kinase type 2 beta	2.21	0.0069	up
XLOC_023568	ANAPC4	Anaphase promoting complex subunit 4	2.83	0.0083	up
XLOC_023572	-		2.38	0.0058	up
XLOC_023579	C6H4orf52	Chromosome 6 open reading frame 52	2.07	0.0146	up

XLOC_023581	RBPJ	Recombination signal binding protein for immunoglobulin kappa J region	2.85	0.0120	up
XLOC_023583	STIM2	Stromal interaction molecule 2	1.92	0.0375	up
XLOC_023596	PGM2	Uncharacterized protein	2.91	0.0015	up
XLOC_023598	TBC1D1	TBC1 (tre-2/USP6, BUB2, cdc16) domain family, member 1	2.47	0.0042	up
XLOC_023599	KLF3	Kruppel-like factor 3 (basic)	2.79	0.0266	up
XLOC_023601	FAM114A1	family with sequence similarity 114, member A1	3.06	0.0033	up
XLOC_023602	KLHL5	Kelch-like family member 5	2.40	0.0059	up
XLOC_023603	WDR19	WD repeat domain 19	2.26	0.0189	up
XLOC_023606	LIAS	Lipoic acid synthetase	3.00	0.0245	up
XLOC_023609	UBE2K	Ubiquitin-conjugating enzyme E2K	2.61	0.0211	up
XLOC_023611	N4BP2	NEDD4 binding protein 2	2.48	0.0036	up
XLOC_023618	LIMCH1	LIM and calponin homology domains 1	2.99	0.0026	up
XLOC_023620	TMEM33	Transmembrane protein 33	2.73	0.0348	up
XLOC_023621	SLC30A9	Solute carrier family 30 (zinc transporter), member 9	4.00	0.0003	up
XLOC_023626	GUF1	GUF1 GTPase homolog (S. cerevisiae)	2.58	0.0146	up
XLOC_023632	SLAIN2	SLAIN motif family, member 2	2.12	0.0260	up
XLOC_023637	OCIAD1	OCIA domain containing 1	2.51	0.0031	up
XLOC_023648	FIP1L1	Factor interacting with PAPOLA and CPSF1	3.02	0.0032	up
XLOC_023651	BT.67124	Uncharacterized protein	2.40	0.0039	up
XLOC_023653	KIT	v-kit Hardy-Zuckerman 4 feline sarcoma viral oncogene homolog	4.37	5.00E-05	up
XLOC_023654	-		NA	0.0001	up
XLOC_023661	EXOC1	Exocyst complex component 1	2.59	0.0031	up
XLOC_023664	BT.31332	Uncharacterized protein	5.62	0.0064	up
XLOC_023666	SRP72	Signal recognition particle 72kDa	2.48	0.0027	up
XLOC_023669	ENSBTAG00000019359	Uncharacterized protein	3.06	0.0011	up
XLOC_023677	YTHDC1	YTH domain containing 1	2.66	0.0013	up
XLOC_023699	RUFY3	RUN and FYVE domain containing 3	2.37	0.0190	up
XLOC_023701	-		NA	0.0001	up
XLOC_023702	MOB1B	MOB kinase activator 1B	3.28	0.0268	up
XLOC_023703	DCK	Deoxycytidine kinase	3.52	0.0159	up
XLOC_023720	CXCL2	Chemokine (C-X-C motif) ligand 2	2.53	0.0059	up
XLOC_023726	DKFZP564O0823	Prostate androgen-regulated mucin-like protein 1	2.08	0.0470	up
XLOC_023730	USO1	USO1 vesicle docking protein homolog (yeast)	2.59	0.0013	up
XLOC_023731	U1	U1 spliceosomal RNA	NA	0.0256	up
XLOC_023745	Septin 11	Septin 11	1.87	0.0325	up
XLOC_023747	BT.87531	Uncharacterized protein	2.74	0.0402	up
XLOC_023756	ANXA3	Annexin A3	2.25	0.0146	up
XLOC_023758	BMP2K	Uncharacterized protein	2.89	0.0204	up
XLOC_023762	BMP3	Bone morphogenetic protein 3	6.80	0.0194	up
XLOC_023775	THAP9	THAP domain containing 9	2.87	0.0311	up
XLOC_023777	COPS4	COP9 constitutive photomorphogenic homolog subunit 4 (Arabidopsis)	2.50	0.0023	up
XLOC_023786	BT.64781	Uncharacterized protein	3.34	0.0006	up
XLOC_023787	AFF1	AF4/FMR2 family, member 1	2.90	0.0057	up
XLOC_023788	NUDT9	Nudix (nucleoside diphosphate linked moiety X)-type motif 9	3.36	0.0002	up
XLOC_023801	STX18	Syntaxin 18	1.99	0.0229	up
XLOC_023807	LYAR	Ly1 antibody reactive homolog (mouse)	1.99	0.0388	up
XLOC_023808	TMEM128	Transmembrane protein 128	2.51	0.0020	up
XLOC_023812	NOP14	NOP14 nucleolar protein homolog (yeast)	1.96	0.0275	up
XLOC_023813	MFSD10	Major facilitator superfamily domain containing 10	3.62	0.0203	up
XLOC_023822	MXD4	MAX dimerization protein 4	3.28	0.0082	up
XLOC_023825	BT.105430	Uncharacterized protein	2.10	0.0207	up
XLOC_023829	BT.102972	Uncharacterized protein	2.74	0.0343	up

XLOC_023830	IDUA	Iduronidase, alpha-L-	3.03	0.0020	up
XLOC_023834	BT.36159	Uncharacterized protein	2.04	0.0297	up
XLOC_023835	UVSSA	UV-stimulated scaffold protein A	1.98	0.0354	up
XLOC_023841	-		NA	5.00E-05	up
XLOC_023848	C1QTNF7	C1q and tumor necrosis factor related protein 7	3.10	0.0260	up
XLOC_023853	CD38	CD38 molecule	3.70	0.0161	up
XLOC_023861	CNO	Cappuccino homolog (mouse)	3.99	0.0018	up
XLOC_023863	TBC1D14	TBC1 domain family, member 14	2.59	0.0043	up
XLOC_023876	ENSBTAG00000032764	Uncharacterized protein	4.64	0.0020	up
XLOC_023882	-		NA	0.0002	up
XLOC_023883	-		NA	0.0003	up
XLOC_023888	BT.95206	Uncharacterized protein	3.21	0.0062	up
XLOC_023891	SYNPO2	Synaptopodin 2	2.06	0.0230	up
XLOC_023892	BT.67542	Uncharacterized protein	2.93	0.0031	up
XLOC_023893	SNORA24	Small nucleolar RNA	2.73	0.0147	up
XLOC_023903	LARP7	Uncharacterized protein	3.29	0.0004	up
XLOC_023907	ALPK1	Alpha-kinase 1	4.20	5.00E-05	up
XLOC_023908	MGC155012	Adaptor-related protein complex 1 associated regulatory protein (AP1AR)	2.01	0.0496	up
XLOC_023909	C6H4orf32	Chromosome 6 open reading frame 32	2.91	0.0003	up
XLOC_023915	GAR1	GAR1 ribonucleoprotein	2.44	0.0163	up
XLOC_023917	SEC24B	SEC24 family member B	2.45	0.0032	up
XLOC_023918	OSTC	Oligosaccharyltransferase complex subunit	2.55	0.0016	up
XLOC_023920	HADH	Hydroxyacyl-CoA dehydrogenase	1.94	0.0256	up
XLOC_023921	CYP2U1	Cytochrome P450, family 2, subfamily U, polypeptide 1	3.79	0.0115	up
XLOC_023922	SGMS2	Sphingomyelin synthase 2	2.97	0.0369	up
XLOC_023923	SCYE1	Aminoacyl tRNA synthetase complex-interacting multifunctional protein 1	2.67	0.0010	up
XLOC_023928	BT.87504	Uncharacterized protein	2.97	0.0222	up
XLOC_023933	TET2	Tet methylcytosine dioxygenase 2	2.65	0.0008	up
XLOC_023939	-		2.34	0.0170	up
XLOC_023941	NFKB1	Nuclear factor of kappa light polypeptide gene enhancer in B-cells 1	2.36	0.0049	up
XLOC_023943	-		NA	5.00E-05	up
XLOC_023948	-		NA	0.0001	up
XLOC_023949	-		NA	0.0001	up
XLOC_023953	METAP1	Methionyl aminopeptidase 1	2.60	0.0066	up
XLOC_023956	BT.65168	Uncharacterized protein	2.46	0.0063	up
XLOC_023962	BT.67505	Uncharacterized protein	3.01	0.0062	up
XLOC_023963	SMARCAD1	SWI/SNF-related matrix-associated actin-dependent regulator of chromatin subfamily A containing DEAD/H box 1	2.92	0.0019	up
XLOC_023969	CCSER1	Family with sequence similarity 190, member A	2.80	0.0190	up
XLOC_023970	MMRN1	Multimerin 1	2.53	0.0082	up
XLOC_023971	TIGD2	Tigger transposable element derived 2	2.52	0.0187	up
XLOC_023974	HERC5	Hect domain and RLD 5	3.26	0.0204	up
XLOC_023975	HERC6	Hect domain and RLD 6; Uncharacterized protein	2.87	0.0059	up
XLOC_023978	PKD2	Polycystic kidney disease 2 (autosomal dominant)	2.38	0.0050	up
XLOC_023980	-		3.40	0.0068	up
XLOC_023981	-		2.71	0.0149	up
XLOC_023988	LCORL	Ligand dependent nuclear receptor corepressor-like	3.37	0.0276	up
XLOC_023990	GPR125	G protein-coupled receptor 125	2.35	0.0106	up
XLOC_023992	PPARGC1A	Peroxisome proliferator-activated receptor gamma, coactivator 1 alpha	2.24	0.0094	up

XLOC_023994	DHX15	DEAH (Asp-Glu-Ala-His) box polypeptide 15	2.20	0.0072	up
XLOC_023997	CCDC149	Coiled-coil domain containing 149	2.31	0.0104	up
XLOC_023999	SEPSECS	Sep (O-phosphoserine) tRNA:Sec (selenocysteine) tRNA synthase	3.42	0.0082	up
XLOC_024015	RELL1	RELT-like 1	2.54	0.0255	up
XLOC_024021	BT.45021	Uncharacterized protein	3.21	0.0083	up
XLOC_024024	RFC1	Replication factor C (activator 1) 1, 145kDa	3.10	0.0007	up
XLOC_024025	-		2.84	0.0131	up
XLOC_024028	C6H4orf34	Chromosome 6 open reading frame 34	2.41	0.0104	up
XLOC_024034	PDS5A	PDS5, regulator of cohesion maintenance, homolog A (S. cerevisiae)	2.46	0.0081	up
XLOC_024037	BT.22438	Uncharacterized protein	2.63	0.0124	up
XLOC_024038	APBB2	Amyloid beta (A4) precursor protein-binding, family B, member 2	1.99	0.0402	up
XLOC_024050	-		7.56	0.0478	up
XLOC_024053	GNPDA2	Glucosamine-6-phosphate deaminase 2	2.41	0.0474	up
XLOC_024061	NFXL1	Nuclear transcription factor, X-box binding-like 1	2.44	0.0378	up
XLOC_024070	OCIAD2	OCIA domain containing 2	1.99	0.0233	up
XLOC_024072	SGCB	Sarcoglycan, beta (43kDa dystrophin-associated glycoprotein)	3.08	0.0018	up
XLOC_024073	USP46	Ubiquitin specific peptidase 46	3.14	0.0002	up
XLOC_024074	SCFD2	Sec1 family domain containing 2	2.36	0.0109	up
XLOC_024075	LNX1	Ligand of numb-protein X 1	2.26	0.0099	up
XLOC_024081	KDR	Kinase insert domain receptor (a type III receptor tyrosine kinase)	3.43	0.0004	up
XLOC_024088	AASDH	Aminoadipate-semialdehyde dehydrogenase	3.74	0.0015	up
XLOC_024098	NOA1	Nitric oxide associated 1	2.15	0.0094	up
XLOC_024099	IGFBP7	Insulin-like growth factor binding protein 7	3.03	0.0148	up
XLOC_024104	CENPC	Centromere protein C 1	2.97	0.0023	up
XLOC_024105	UBA6	Ubiquitin-like modifier activating enzyme 6	2.57	0.0040	up
XLOC_024114	ENSBTAG00000035726	Uncharacterized protein	5.43	0.0074	up
XLOC_024127	SULT1B1	Sulfotransferase family, cytosolic, 1B, member 1	2.84	0.0195	up
XLOC_024130	-		3.41	0.0481	up
XLOC_024141	GRSF1	G-rich sequence factor 1	2.79	0.0006	up
XLOC_024147	COX18	Cytochrome c oxidase assembly homolog (S. cerevisiae)	2.43	0.0418	up
XLOC_024148	ANKRD17	Ankyrin repeat domain 17	2.36	0.0062	up
XLOC_024150	RASSF6	Ras association (RalGDS/AF-6) domain family member 6	5.78	0.0004	up
XLOC_024154	-		NA	0.0002	up
XLOC_024155	RCHY1	Ring finger and CHY zinc finger domain containing 1	2.04	0.0288	up
XLOC_024157	G3BP2	GTPase activating protein (SH3 domain) binding protein 2	1.82	0.0407	up
XLOC_024160	SDAD1	SDA1 domain containing 1	3.57	0.0003	up
XLOC_024161	CXCL9	Chemokine (C-X-C motif) ligand 9	13.53	0.0006	up
XLOC_024164	BT.52474	Uncharacterized protein	2.90	0.0068	up
XLOC_024165	SCARB2	Scavenger receptor class B, member 2	2.43	0.0059	up
XLOC_024170	ANKRD56	Sosondowah ankyrin repeat domain family member B	2.71	0.0071	up
XLOC_024171	CCNI	Cyclin I	2.12	0.0207	up
XLOC_024173	CNOT6L	CCR4-NOT transcription complex, subunit 6-like	3.32	0.0091	up
XLOC_024180	ANTXR2	Anthrax toxin receptor 2	2.74	0.0037	up
XLOC_024181	-		NA	5.00E-05	up
XLOC_024182	-		NA	0.0001	up

XLOC_024183	PRKG2	Protein kinase, cGMP-dependent, type II	2.43	0.0100	up
XLOC_024185	RASGEF1B	RasGEF domain family, member 1B	2.09	0.0268	up
XLOC_024188	TMEM150C	Transmembrane protein 150C	2.68	0.0084	up
XLOC_024190	SCD5	Stearoyl-CoA desaturase 5	2.36	0.0120	up
XLOC_024191	SEC31A	SEC31 homolog A (S. cerevisiae)	2.02	0.0279	up
XLOC_024194	PLAC8	Placenta-specific 8	3.23	0.0265	up
XLOC_024196	COQ2	Coenzyme Q2 homolog, prenyltransferase (yeast)	2.43	0.0038	up
XLOC_024201	HELQ	Helicase, POLQ-like	6.57	0.0163	up
XLOC_024203	FAM175A	Family with sequence similarity 175, member A	2.97	0.0086	up
XLOC_024206	WDFY3	WD repeat and FYVE domain containing 3	3.47	0.0002	up
XLOC_024214	KLHL8	Kelch-like family member 8	2.09	0.0478	up
XLOC_024219	HSD17B11	Hydroxysteroid (17-beta) dehydrogenase 11	2.26	0.0193	up
XLOC_024220	SPARCL1	SPARC-like 1 (hevin)	2.42	0.0042	up
XLOC_024235	ZBTB49	Zinc finger and BTB domain containing 49	3.04	0.0191	up
XLOC_024239	HTT	Huntingtin	2.96	0.0009	up
XLOC_024247	GRK4	G protein-coupled receptor kinase 4	2.98	0.0022	up
XLOC_024251	ADD1	adducin 1 (alpha)	3.28	0.0182	up
XLOC_024253	FAM193A	Family with sequence similarity 193, member A	2.31	0.0086	up
XLOC_024254	RNF4	Ring finger protein 4	2.14	0.0114	up
XLOC_024256	-		NA	0.0001	up
XLOC_024260	ATP5I	ATP synthase, H+ transporting, mitochondrial Fo complex, subunit E	3.02	0.0009	up
XLOC_024261	MFSD7	Major facilitator superfamily domain containing 7	2.34	0.0117	up
XLOC_024264	GAK	Cyclin G associated kinase	3.06	0.0018	up
XLOC_024265	DGKQ	Diacylglycerol kinase, theta 110kDa	3.55	0.0155	up
XLOC_024272	SPON2	Spondin 2, extracellular matrix protein	2.65	0.0093	up
XLOC_024273	CTBP1	Uncharacterized protein	2.41	0.0088	up
XLOC_024278	LETM1	Leucine zipper-EF-hand containing transmembrane protein 1	2.02	0.0279	up
XLOC_024279	BT.97056	Uncharacterized protein	3.09	0.0080	up
XLOC_024280	C4orf48	Chromosome 4 open reading frame 48	9.37	0.0386	up
XLOC_024282	BT.75467	Uncharacterized protein	2.44	0.0098	up
XLOC_024284	ZNF518B	Zinc finger protein 518B	3.08	0.0211	up
XLOC_024287	RAB28	RAB28, member RAS oncogene family	2.47	0.0050	up
XLOC_024289	BOD1L1	Biorientation of chromosomes in cell division 1-like	2.96	0.0006	up
XLOC_024292	BT.17790	Uncharacterized protein	2.01	0.0176	up
XLOC_024296	PROM1	Prominin 1	3.53	0.0002	up
XLOC_024298	TAPT1	Transmembrane anterior posterior transformation 1	2.06	0.0266	up
XLOC_024302	QDPR	Quinoid dihydropteridine reductase	1.96	0.0300	up
XLOC_024304	MRFAP1	Morf4 family associated protein 1	2.01	0.0270	up
XLOC_024311	AFAP1	Actin filament associated protein 1	2.06	0.0471	up
XLOC_024425	ILVBL	IlvB (bacterial acetolactate synthase)-like	2.15	0.0375	up
XLOC_024632	-		NA	0.0003	up
XLOC_024802	TPGS1	Tubulin polyglutamylase complex subunit 1	2.79	0.0413	up
XLOC_024806	HCN2	Hyperpolarization activated cyclic nucleotide-gated potassium channel 2	1.95	0.0333	up
XLOC_024845	-		NA	5.00E-05	up
XLOC_025005	-		NA	5.00E-05	up
XLOC_025260	-		NA	0.0001	up
XLOC_025290	-		NA	5.00E-05	up
XLOC_025400	BT.105935	Uncharacterized protein	1.94	0.0475	up
XLOC_025492	-		NA	5.00E-05	up
XLOC_025493	-		NA	5.00E-05	up

XLOC_025523	BT.68694	Uncharacterized protein	2.20	0.0072	up
XLOC_025531	CSNK1G3	Casein kinase 1, gamma 3	1.84	0.0499	up
XLOC_025657	UQCR11	Ubiquinol-cytochrome c reductase, complex III subunit XI	1.97	0.0473	up
XLOC_025765	-		NA	0.0002	up
XLOC_025902	NUDT12	Nudix (nucleoside diphosphate linked moiety X)-type motif 12	1.88	0.0426	up
XLOC_025906	-		NA	0.0003	up
XLOC_026123	RUSC2	RUN and SH3 domain containing 2	1.95	0.0308	up
XLOC_026259	-		NA	5.00E-05	up
XLOC_026807	TMEM245	Transmembrane protein 245	2.67	0.0363	up
XLOC_026829	-		NA	5.00E-05	up
XLOC_026896	-		4.22	0.0239	up
XLOC_026899	KCNQ5	Potassium voltage-gated channel, KQT-like subfamily, member 5	12.42	0.0165	up
XLOC_026905	-		NA	5.00E-05	up
XLOC_027337	-		NA	0.0003	up
XLOC_027546	-		NA	5.00E-05	up
XLOC_027665	-		NA	0.0001	up
XLOC_028013	RPS23	Ribosomal protein S23	3.80	0.0118	up
XLOC_028154	GPR64	G protein-coupled receptor 64	2.54	0.0126	up
XLOC_028161	-		NA	5.00E-05	up
XLOC_028248	-		NA	0.0040	up
XLOC_028500	-		NA	5.00E-05	up
XLOC_028519	-		NA	5.00E-05	up
XLOC_028702	-		NA	0.0005	up
XLOC_000002	RCAN1	Regulator of calcineurin 1	0.35	0.0015	down
XLOC_000091	-		0.00	0.0122	down
XLOC_000557	-		0.43	0.0084	down
XLOC_000599	ENSBTAG00000038697	Uncharacterized protein	0.00	0.0005	down
XLOC_000629	BT.3923	Uncharacterized protein	0.29	0.0469	down
XLOC_000961	BT.85209	Uncharacterized protein	0.43	0.0379	down
XLOC_001118	SIK1	Serine/threonine-protein kinase	0.55	0.0336	down
XLOC_001733	FOS	FBJ murine osteosarcoma viral oncogene homolog	0.30	0.0017	down
XLOC_001849	-		0.00	0.0096	down
XLOC_001898	GRAMD2	GRAM domain containing 2	0.44	0.0375	down
XLOC_002260	SEMA6D	Sema domain, transmembrane domain (TM), and cytoplasmic domain, (semaphorin) 6D	0.45	0.0409	down
XLOC_002436	STON2	Stonin 2	0.45	0.0074	down
XLOC_003522	-		0.00	0.0001	down
XLOC_003686	BT.29579	Uncharacterized protein	0.51	0.0474	down
XLOC_003872	HSPH1	Heat shock 105kDa/110kDa protein 1	0.45	0.0120	down
XLOC_003967	BT.61919	Uncharacterized protein	0.37	0.0130	down
XLOC_004339	ENSBTAG00000003937	Uncharacterized protein	0.31	0.0499	down
XLOC_004450	BT.63212	Uncharacterized protein	0.07	0.0002	down
XLOC_004817	ITIH2	Inter-alpha-trypsin inhibitor heavy chain 2	0.39	0.0032	down
XLOC_005027	-		0.00	0.0108	down
XLOC_005145	-		0.30	0.0195	down
XLOC_005147	-		0.00	5.00E-05	down
XLOC_005156	ADA	Adenosine deaminase	0.07	0.0286	down
XLOC_005214	B4GALT5	UDP-Gal:betaGlcNAc beta 1,4-galactosyltransferase, polypeptide 5	0.48	0.0401	down
XLOC_005319	ST3GAL1	ST3 beta-galactoside alpha-2,3-sialyltransferase 1	0.53	0.0418	down
XLOC_005533	GEM	GTP binding protein overexpressed in skeletal muscle , transcript variant 1	0.47	0.0363	down
XLOC_005665	MYC	V-myc myelocytomatosis viral oncogene homolog (avian)	0.51	0.0163	down
XLOC_007126	-		0.48	0.0407	down
XLOC_007166	TGFB2	Transforming growth factor, beta 2	0.54	0.0474	down

XLOC_007447	ELF3	E74-like factor 3 (ets domain transcription factor, epithelial-specific)	0.50	0.0454	down
XLOC_007525	CHI3L1	Chitinase 3-like 1 (cartilage glycoprotein-39)	0.48	0.0216	down
XLOC_007556	RAB7B	Member RAS oncogene family	0.44	0.0304	down
XLOC_007560	-		0.30	0.0444	down
XLOC_007583	RGS1	Regulator of G-protein signaling 1	0.30	0.0054	down
XLOC_007669	SELP	Selectin P (granule membrane protein 140kDa, antigen CD62)	0.31	0.0150	down
XLOC_007981	NR3C2	Nuclear receptor subfamily 3, group C, member 2	0.34	0.0029	down
XLOC_007997	-		0.43	0.0046	down
XLOC_008191	BT.53553	Uncharacterized protein	0.30	0.0103	down
XLOC_008837	IRF8	Interferon regulatory factor 8	0.51	0.0254	down
XLOC_009047	-		0.00	0.0047	down
XLOC_009183	-		0.10	0.0279	down
XLOC_009240	FOSB	FBJ murine osteosarcoma viral oncogene homolog B	0.21	0.0005	down
XLOC_009648	MT1A	Metallothionein-1A	0.17	0.0017	down
XLOC_009649	MT1E	Metallothionein 1E	0.17	0.0245	down
XLOC_009651	MT2A	Metallothionein 2A	0.35	0.0039	down
XLOC_009681	-		0.46	0.0347	down
XLOC_009705	TPPP3	Tubulin polymerization-promoting protein family member 3	0.49	0.0218	down
XLOC_009913	KCNN4	Potassium intermediate/small conductance calcium-activated channel, subfamily N, member 4	0.39	0.0376	down
XLOC_010383	SPNS2	Spinster homolog 2 (Drosophila)	0.36	0.0015	down
XLOC_010661	ARL4D	ADP-ribosylation factor-like 4D	0.51	0.0213	down
XLOC_010800	SOCS3	Suppressor of cytokine signaling 3	0.38	0.0276	down
XLOC_011014	CCL2	Chemokine (C-C motif) ligand 2	0.19	0.0151	down
XLOC_011157	CAMKK1	Calcium/calmodulin-dependent protein kinase kinase 1, alpha	0.32	0.0313	down
XLOC_011269	PMP22	Peripheral myelin protein 22	0.50	0.0116	down
XLOC_011272	-		0.00	0.0014	down
XLOC_011718	RGS9	Regulator of G-protein signaling 9	0.36	0.0090	down
XLOC_011852	ITGB6	Integrin, beta 6	0.43	0.0233	down
XLOC_012311	-		0.00	0.0030	down
XLOC_012793	SFN	Stratifin	0.42	0.0445	down
XLOC_012880	-		0.33	0.0005	down
XLOC_013166	BT.85182	Uncharacterized protein	0.57	0.0399	down
XLOC_013217	FAM159B	Family with sequence similarity 159, member B	0.50	0.0207	down
XLOC_013547	BT.18516	Uncharacterized protein	0.23	0.0018	down
XLOC_014139	SERPINA11	Serpin peptidase inhibitor, clade A (alpha-1 antiproteinase, antitrypsin), member 11	0.54	0.0469	down
XLOC_014157	-		0.17	0.0429	down
XLOC_014158	-		0.21	0.0310	down
XLOC_014159	C14ORF49	Chromosome 14 open reading frame 49	0.40	0.0294	down
XLOC_014280	-		0.00	0.0022	down
XLOC_015404	-		0.00	0.0034	down
XLOC_015450	-		0.18	0.0160	down
XLOC_015468	-		0.31	0.0258	down
XLOC_015548	GPR110	G protein-coupled receptor 110	0.37	0.0009	down
XLOC_015601	HSPA1A	Heat shock 70kDa protein 1A	0.29	0.0003	down
XLOC_015603	-		0.19	0.0003	down
XLOC_015816	-		0.00	0.0061	down
XLOC_016019	-		0.37	0.0271	down
XLOC_016188	EPB41L3	Erythrocyte membrane protein band 4.1-like 3	0.43	0.0072	down
XLOC_016728	ZFAND2A	Zinc finger, AN1-type domain 2A	0.18	0.0046	down
XLOC_017082	SERPINE1	Serpin peptidase inhibitor, clade E (nexin, plasminogen activator inhibitor type 1), member 1	0.22	5.00E-05	down

XLOC_017390	BAG3	BCL2-associated athanogene 3	0.37	0.0011	down
XLOC_017407	SPADH1	Spermadhesin 1	0.37	0.0032	down
XLOC_017919	BT.85406	Uncharacterized protein	0.15	0.0495	down
XLOC_018104	BT.97928	Uncharacterized protein	0.53	0.0375	down
XLOC_018702	-		0.29	0.0014	down
XLOC_018883	-		0.00	0.0010	down
XLOC_019252	ADAMTS4	ADAM metallopeptidase with thrombospondin type 1 motif 4	0.26	0.0092	down
XLOC_019428	TMOD4	Tropomodulin 4 (muscle)	0.51	0.0330	down
XLOC_019507	-		0.00	0.0038	down
XLOC_019583	-		0.33	0.0337	down
XLOC_019618	F3	Coagulation factor III	0.36	0.0137	down
XLOC_020088	HSPA6	Heat shock 70kDa protein 6	0.21	0.0403	down
XLOC_020191	-		0.00	0.0003	down
XLOC_020249	CGN	Cingulin	0.43	0.0139	down
XLOC_020541	PDE4B	Phosphodiesterase 4B, cAMP-specific	0.39	0.0396	down
XLOC_020881	IL6	Interleukin 6 (interferon, beta 2)	0.00	5.00E-05	down
XLOC_021085	BT.91596	Uncharacterized protein	0.34	0.0279	down
XLOC_021372	BT.102008	Uncharacterized protein	0.46	0.0141	down
XLOC_021530	ADCYAP1R1	Pituitary adenylate cyclase-activating polypeptide type I receptor	0.46	0.0175	down
XLOC_021897	BT.87815	Uncharacterized protein	0.41	0.0025	down
XLOC_021929	BIN2	Bridging integrator 2	0.48	0.0200	down
XLOC_021978	-		0.03	0.0153	down
XLOC_021984	-		0.00	0.0038	down
XLOC_022018	NELL2	NEL-like 2 (chicken)	0.52	0.0169	down
XLOC_022114	SDR9C7	Short chain dehydrogenase/reductase family 9C, member 7	0.28	0.0457	down
XLOC_022599	-		0.45	0.0233	down
XLOC_022600	-		0.54	0.0482	down
XLOC_022742	KRT18	Keratin 18	0.40	0.0082	down
XLOC_022786	ENSBTAG00000037775	Uncharacterized protein	0.54	0.0468	down
XLOC_023018	-		0.00	0.0002	down
XLOC_023047	-		0.00	0.0013	down
XLOC_023147	APOLD1	Apolipoprotein L domain containing 1	0.39	0.0043	down
XLOC_023425	-		0.00	0.0096	down
XLOC_024317	GFPT2	Glutamine-fructose-6-phosphate transaminase 2	0.34	0.0302	down
XLOC_024579	ANGPTL4	Angiopoietin 4	0.30	0.0031	down
XLOC_024894	EGR1	Early growth response 1	0.45	0.0092	down
XLOC_024907	CXXC5	CXXC finger protein 5	0.38	0.0210	down
XLOC_025135	-		0.18	0.0090	down
XLOC_025136	-		0.22	0.0061	down
XLOC_025554	-		0.00	5.00E-05	down
XLOC_025920	PALLD	Palladin, cytoskeletal associated protein	0.53	0.0464	down
XLOC_026660	STC1	Stanniocalcin 1	0.25	0.0001	down
XLOC_026730	FBP1	Fructose-1,6-bisphosphatase 1	0.40	0.0045	down
XLOC_026769	GADD45G	Growth arrest and DNA-damage-inducible, gamma	0.49	0.0140	down
XLOC_026812	EPB41L4B	Erythrocyte membrane protein band 4.1 like 4B	0.55	0.0419	down
XLOC_027299	-		0.41	0.0241	down
XLOC_027571	THBS2	Thrombospondin 2	0.36	0.0173	down
XLOC_027931	-		0.33	0.0268	down
XLOC_028093	-		0.15	5.00E-05	down
XLOC_028128	ENSBTAG00000006383	40S ribosomal protein S3a	0.10	5.00E-05	down
XLOC_028876	MGC151921	Odorant-binding protein-like	0.30	0.0003	down
XLOC_028877	BDA20	Major allergen	0.16	0.0048	down

NA indicated that the expression abundance of these transcripts were zero in AH group, but higher than zero in RS group.

Table S3 The differentially expressed proteins in mammary glands between RS- and AH-fed dairy cows

Protein accession	Protein	Protein description	RS/AH	*P*-value	Regulation
IPI00694204	KRT19	Keratin, type I cytoskeletal 19	1.72	0.0000	up
IPI00689750	LMNA	Uncharacterized protein	1.32	0.0000	up
IPI00685418	KRT8	Keratin, type II cytoskeletal 8	1.88	3.25E-12	up
IPI00694214	KRT7	Keratin, type II cytoskeletal 7	1.70	3.25E-12	up
IPI00689228	VIM	Vimentin	1.39	3.25E-12	up
IPI00698900	ACTB	Actin, cytoplasmic 1	1.23	4.27E-12	up
IPI00713573	COL6A1	Collagen, type VI, alpha 1	1.29	4.96E-12	up
IPI00692588	KRT13	Uncharacterized protein	2.68	6.70E-12	up
IPI00837992	COL6A2	Collagen, type VI, alpha 2	1.24	6.86E-11	up
IPI00696729	NUCB2	Uncharacterized protein	1.47	1.38E-10	up
IPI00717116	－	Uncharacterized protein	1.81	1.77E-10	up
IPI00688608	DCN	Decorin	1.24	2.28E-10	up
IPI00691963	CALR	Calreticulin	1.27	1.86E-09	up
IPI00716158	LUM	Lumican	1.44	5.16E-09	up
IPI00904104	TKT	Transketolase	1.25	1.12E-08	up
IPI00712775	EEF1A1	Elongation factor 1-alpha 1	1.35	2.75E-08	up
IPI00905865	DES	Desmin	1.40	4.53E-08	up
IPI00716493	CALU	Calumenin	1.40	7.28E-08	up
IPI00689325	PDIA3	Protein disulfide-isomerase A3	1.26	8.89E-08	up
IPI00691212	ORM1	Alpha-1-acid glycoprotein	1.91	1.41E-07	up
IPI00867179	ST13	Suppression of tumorigenicity 13 (colon carcinoma) (Hsp70 interacting protein)	1.30	7.72E-07	up
IPI00708921	KRT18	Keratin, type I cytoskeletal 18	1.65	9.37E-07	up
IPI00716123	OGN	Mimecan	1.46	9.47E-07	up
IPI00955263	－	Periostin	2.27	1.27E-06	up
IPI00702028	TAGLN2	Transgelin-2	1.27	1.50E-06	up
IPI01002277	CRKL	V-crk sarcoma virus CT10 oncogene homolog (avian)-like	1.50	2.77E-06	up
IPI00871133	A2M	Alpha-2-macroglobulin	1.20	3.33E-06	up
IPI00715281	OLFML1	Olfactomedin-like 1	1.94	4.52E-06	up
IPI00686601	SCP2	Isoform SCPx of Non-specific lipid-transfer protein	1.25	1.29E-05	up
IPI00713536	RPS12	40S ribosomal protein S12	1.54	1.39E-05	up
IPI00695506	LCP1	Lymphocyte cytosolic protein 1	1.20	1.46E-05	up
IPI00686183	GPX1	Glutathione peroxidase 1	1.30	1.69E-05	up
IPI00692627	ALDH1A1	Retinal dehydrogenase 1	1.23	2.00E-05	up
IPI00730144	PAPSS2	3-phosphoadenosine 5-phosphosulfate synthase 2	1.29	2.15E-05	up
IPI00698589	PGAM1	Phosphoglycerate mutase 1	1.21	2.69E-05	up
IPI00698039	H4	Histone 4	1.29	3.30E-05	up
IPI00868597	CLINT1	Clathrin interactor 1	1.22	3.97E-05	up
IPI00695965	APOA4	Apolipoprotein A-IV	1.37	4.01E-05	up
IPI00700295	C5H22orf28	tRNA-splicing ligase RtcB homolog	1.21	4.28E-05	up
IPI00714673	FN1	Embryo-specific fibronectin 1 transcript variant	1.30	4.97E-05	up
IPI00695489	SERPINA1	Alpha-1-antiproteinase	1.28	5.35E-05	up
IPI00706458	SRI	Sorcin	1.51	5.95E-05	up
IPI00906033	LOC788816	Tropomyosin 2, beta-like isoform 1	1.48	7.30E-05	up
IPI00696912	ACSS1	Acetyl-CoA synthetase 2	1.23	8.68E-05	up
IPI00702891	ERP29	Endoplasmic reticulum resident protein 29	1.22	0.0001	up
IPI00717119	ASPN	Asporin	1.24	0.0001	up
IPI00688921	CAPZB	Isoform Beta-3 of F-actin-capping protein subunit beta	1.22	0.0001	up
IPI00704449	KCTD12	Potassium channel tetramerisation domain containing 12-like	1.39	0.0001	up
IPI00718757	COTL1	Coactosin-like protein	1.41	0.0002	up
IPI00689323	PMM2	Phosphomannomutase 2	1.26	0.0002	

IPI00843257	EFHD2	EF-hand domain-containing protein D2	1.28	0.0002	up
IPI00694580	SH3BGRL3	SH3 domain-binding glutamic acid-rich-like protein 3	1.42	0.0002	up
IPI00685792	S100A10	Protein S100-A10	1.29	0.0002	up
IPI00904732	MAPRE1	Microtubule-associated protein, RP/EB family, member 1	1.26	0.0003	up
IPI00697891	HNRNPL	Heterogeneous nuclear ribonucleoprotein L	1.36	0.0003	up
IPI00840588	TGFBI	LOC539596 protein	1.44	0.0003	up
IPI00713642	PDCD4	Programmed cell death 4 (neoplastic transformation inhibitor)	1.37	0.0003	up
IPI00703157	FHL1	Four and a half LIM domains 1 isoform 2	1.32	0.0003	up
IPI00694751	S100A9	Protein S100-A9	1.76	0.0003	up
IPI00697196	MANF	Mesencephalic astrocyte-derived neurotrophic factor	1.29	0.0004	up
IPI00704735	PEBP1	Phosphatidylethanolamine-binding protein 1	1.34	0.0004	up
IPI00703268	TPM2	Isoform 2 of Tropomyosin beta chain	1.44	0.0004	up
IPI00727017	TPM4	Tropomyosin 4	1.44	0.0004	up
IPI00968658	SERPINA3-1	Serpin A3-6	1.38	0.0005	up
IPI00704729	SEP 15	15 kDa selenoprotein	1.28	0.0005	up
IPI00701223	BUB3	Mitotic checkpoint protein BUB3	1.35	0.0006	up
IPI00707359	DAG1	Dystroglycan	1.43	0.0007	up
IPI00711573	ERH	Enhancer of rudimentary homolog	1.82	0.0008	up
IPI00883375	PRDX3	PRDX3 protein	1.24	0.0008	up
IPI00705660	TPT1	Translationally-controlled tumor protein	1.31	0.0008	up
IPI00704877	PLAC9	Placenta-specific protein 9	1.53	0.0008	up
IPI00715339	FBLN5	Fibulin-5	1.53	0.0008	up
IPI00717085	CATHL1	Cathelicidin 1-like	1.31	0.0008	up
IPI00703547	CALB1	Calbindin	1.47	0.0009	up
IPI00707587	DPT	Dermatopontin	1.26	0.0010	up
IPI00718193	CSRP1	Cysteine and glycine-rich protein 1	1.24	0.0010	up
IPI00718381	SH3GL1	Endophilin-A2	1.24	0.0010	up
IPI00700920	AKR1B1	Aldo-keto reductase family 1, member B1	1.40	0.0010	up
IPI00688489	ACTC1	Actin, alpha cardiac muscle 1	1.52	0.0010	up
IPI00702700	ITIH3	Inter-alpha-trypsin inhibitor heavy chain H3	1.50	0.0011	up
IPI00694312	BDH2	3-hydroxybutyrate dehydrogenase type 2	1.33	0.0012	up
IPI00944429	IDI1	Isoform 1 of Isopentenyl-diphosphate Delta-isomerase 1	1.24	0.0012	up
IPI00728768	SET	SET translocation	1.33	0.0013	up
IPI00698993	AZGP1	Zinc-alpha-2-glycoprotein	1.45	0.0013	up
IPI00689362	TTR	Transthyretin	1.25	0.0014	up
IPI00713141	MYL9	Myosin, light chain 9, regulatory	1.24	0.0015	up
IPI00907801	－	Uncharacterized protein (Fragment)	1.23	0.0015	up
IPI00705000	LOC522960	Histone H2B	1.83	0.0015	up
IPI00700789	PHPT1	14 kDa phosphohistidine phosphatase	1.29	0.0016	up
IPI00716555	GDA	Guanine deaminase	1.72	0.0016	up
IPI00695508	CALM	Calmodulin	1.40	0.0017	up
IPI00692911	ANP32A	Acidic leucine-rich nuclear phosphoprotein 32 family member A	1.30	0.0018	up
IPI00742596	DHFR	Dihydrofolate reductase	1.39	0.0019	up
IPI00690439	－	Uncharacterized protein	1.61	0.0019	up
IPI00718311	PSAP	Isoform 1 of Proactivator polypeptide	1.24	0.0019	up
IPI00689760	VCAN	Isoform V2 of Versican core protein	1.62	0.0019	up
IPI00699798	PPP1CB	Serine/threonine-protein phosphatase PP1-beta catalytic subunit	1.22	0.0021	up
IPI00867339	HSPA2	Heat shock 70kDa protein 1A	1.38	0.0022	up
IPI00706624	PCOLCE	Procollagen C-endopeptidase enhancer	1.55	0.0022	up
IPI00707101	AHSG	Alpha-2-HS-glycoprotein	1.25	0.0022	up
IPI00705463	FUS	RNA-binding protein FUS	1.33	0.0023	up
IPI00691669	CATHL2	Cathelicidin-2	1.51	0.0024	up
IPI00715287	ITIH2	ITIH2 protein	1.25	0.0025	up

IPI00689005	PALLD	Palladin, cytoskeletal associated protein	1.57	0.0026	up
IPI00689035	MFGE8	Isoform Long of Lactadherin	1.23	0.0026	up
IPI00694849	COX5B	Cytochrome c oxidase subunit 5B, mitochondrial	1.24	0.0027	up
IPI00715354	SFN	14-3-3 protein sigma	1.48	0.0028	up
IPI00711750	ARPC3	Actin-related protein 2/3 complex subunit 3	1.58	0.0028	up
IPI00923985	–	Uncharacterized protein (Fragment)	1.84	0.0028	up
IPI00711759	SMS	Spermine synthase	1.47	0.0031	up
IPI00690160	CORO1A	Coronin-1A	1.40	0.0033	up
IPI00701166	KNG1	Isoform HMW of Kininogen-1	1.62	0.0033	up
IPI00716121	SERPINF1	Pigment epithelium-derived factor	1.53	0.0034	up
IPI00708018	REXO2	Oligoribonuclease, mitochondrial (Fragment)	1.35	0.0036	up
IPI00694739	APIP	Probable methylthioribulose-1-phosphate dehydratase	1.28	0.0036	up
IPI00707718	SEPT 11	Septin-11	1.33	0.0037	up
IPI00687601	MAP1B	Microtubule-associated protein 1B	1.33	0.0037	up
IPI00838607	TMED7	Transmembrane emp24 protein transport domain containing 7	1.24	0.0038	up
IPI00690298	LOC515150	SUB1 homolog (S. cerevisiae)	1.41	0.0040	up
IPI00700542	AEBP1	Peroxisome proliferator-activated receptor delta), with a synonym of PPAR-beta	1.23	0.0040	up
IPI00693755	S100A11	Drebrin 1	1.39	0.0044	up
IPI00701698	C1QBP	Tenascin C	1.24	0.0045	up
IPI00695331	SUB1	SUB1 homolog (S. cerevisiae)	1.33	0.0046	up
IPI00703129	VDAC1	Voltage-dependent anion-selective channel protein 1	1.22	0.0047	up
IPI00699355	PPARD	Peroxisome proliferator-activated receptor delta), with a synonym of PPAR-beta	1.35	0.0049	up
IPI00714405	TPM3	Isoform 2 of Tropomyosin alpha-3 chain	1.26	0.0049	up
IPI00711419	DTD1	D-tyrosyl-tRNA(Tyr) deacylase 1	1.25	0.0049	up
IPI00699803	SEPT10	Septin-10	1.35	0.0052	up
IPI00695666	DBN1	Drebrin 1	1.25	0.0052	up
IPI00694504	TNC	Tenascin C	2.15	0.0052	up
IPI00694851	THRAP3	Thyroid hormone receptor associated protein 3	1.32	0.0053	up
IPI00713757	CFH	Complement factor H	1.24	0.0053	up
IPI00714481	MESDC2	LDLR chaperone MESD	1.30	0.0053	up
IPI00709922	FBLN1	Fibulin 1	1.40	0.0055	up
IPI00695142	GP2	Glycoprotein 2	1.26	0.0056	up
IPI00690094	LGALS1	Galectin-1	1.21	0.0059	up
IPI00725325	C28H10orf116	Adipogenesis regulatory factor(ADIRF), a synonym of ADIRF	1.22	0.0059	up
IPI00699107	DNAJB11	DnaJ homolog subfamily B member 11	1.40	0.0062	up
IPI00699002	ANP32B	Acidic leucine-rich nuclear phosphoprotein 32 family member B	1.26	0.0063	up
IPI00703776	ARRB1	Isoform 1A of Beta-arrestin-1	1.26	0.0064	up
IPI00703731	PPID	Peptidyl-prolyl cis-trans isomerase D	1.20	0.0066	up
IPI00692819	IMPA1	Inositol monophosphatase 1	1.35	0.0068	up
IPI00702650	HADHA	FGF-2 binding protein	1.55	0.0068	up
IPI00705941	RAN	GTP-binding nuclear protein Ran	1.60	0.0071	up
IPI00710408	CLTB	Isoform Non-brain of Clathrin light chain B	1.29	0.0071	up
IPI00687539	DPYSL3	DPYSL3 protein	1.29	0.0080	up
IPI00706942	TPI1	Triosephosphate isomerase	1.30	0.0080	up
IPI00712671	UGDH	UDP-glucose 6-dehydrogenase	1.85	0.0083	up
IPI00716580	LOC785758	Splicing factor, arginine/serine-rich 9	1.65	0.0084	up
IPI00847093	RCN1	RCN1 protein	1.23	0.0085	up
IPI00714302	GIMAP7	GTPase, IMAP family member 7)	1.24	0.0086	up

IPI00845215	CPPED1	Isoform 1 of Calcineurin-like phosphoesterase domain-containing protein 1	1.37	0.0087	up
IPI00690001	GCHFR	GTP cyclohydrolase 1 feedback regulatory protein	1.23	0.0098	up
IPI00715091	RPS20	40S ribosomal protein S20	1.42	0.0101	up
IPI00707559	ETFA	Electron transfer flavoprotein subunit alpha, mitochondrial	1.25	0.0104	up
IPI01018577	TRIM28	Transcription intermediary factor 1-beta	1.22	0.0104	up
IPI00712366	FMOD	Fibromodulin	1.76	0.0104	up
IPI00971595	LOC784932	Serpin A3-7-like	1.58	0.0107	up
IPI01028487	GBAS	Glioblastoma amplified sequence	1.21	0.0111	up
IPI00697081	BGN	Biglycan	1.23	0.0111	up
IPI00710385	PRELP	Prolargin	1.60	0.0117	up
IPI00839224	RALY	RALY heterogeneous nuclear ribonucleoprotein	1.20	0.0118	up
IPI00697070	SARNP	SAP domain containing ribonucleoprotein	1.41	0.0123	up
IPI00708311	CACYBP	Calcyclin-binding protein	1.22	0.0125	up
IPI00716195	ATP6V0D1	V-type proton ATPase subunit d 1	1.36	0.0125	up
IPI00707875	PSMA5	Proteasome subunit alpha type-5	1.25	0.0132	up
IPI00829551	PDLIM7	PDZ and LIM domain 7 (enigma)	1.28	0.0135	up
IPI00720259	HAVCR1	Hepatitis A virus cellular receptor 1	1.49	0.0136	up
IPI00697757	PSMB4	Proteasome subunit beta type-4	1.22	0.0139	up
IPI00716881	BPHL	Biphenyl hydrolase-like	1.21	0.0149	up
IPI00690785	FKBP7	FK506 binding protein 7	1.85	0.0153	up
IPI00704835	DFFA	DNA fragmentation factor, 45kDa, alpha polypeptide	1.27	0.0156	up
IPI00690446	CTBP1	C-terminal binding protein 1	1.28	0.0158	up
IPI00697184	RBP4	Retinol-binding protein 4	1.57	0.0161	up
IPI00687625	HNRNPA0	Uncharacterized protein (Fragment)	1.28	0.0162	up
IPI00704728	EIF5A	Eukaryotic translation initiation factor 5A-1	1.27	0.0175	up
IPI00695600	U2AF1	Splicing factor U2AF 35 kDa subunit	1.29	0.0184	up
IPI00695597	C3H1orf123	UPF0587 protein C1orf123 homolog	1.23	0.0194	up
IPI00692468	SOD2	Superoxide dismutase [Mn], mitochondrial	1.45	0.0195	up
IPI00703380	SEP6	Septin-6	1.36	0.0203	up
IPI00715486	LAMC2	Laminin, gamma 2	1.32	0.0214	up
IPI00696616	SSR2	Translocon-associated protein subunit beta	1.20	0.0220	up
IPI00726650	ADK	Adenosine kinase	1.23	0.0227	up
IPI00697355	SNX9	Sorting nexin 9-like	1.21	0.0228	up
IPI00842934	LPIN1	Lipin 1, with a synonym of KIAA0188	1.28	0.0235	up
IPI00687116	PDLIM4	PDZ and LIM domain protein 4	1.30	0.0239	up
IPI00701790	PSMC3	26S protease regulatory subunit 6A	1.27	0.0240	up
IPI00708438	SUCLG1	Succinyl-CoA ligase [GDP-forming] subunit alpha, mitochondrial	1.38	0.0242	up
IPI00706141	SCIN	Adseverin	1.54	0.0244	up
IPI00693073	－	Uncharacterized protein	1.93	0.0253	up
IPI00717759	TRA2B	Transformer-2 protein homolog beta	1.28	0.0254	up
IPI00692676	FHL2	Four and a half LIM domains protein 2	1.24	0.0256	up
IPI00707452	DNAJC8	DnaJ (Hsp40) homolog, subfamily C, member 8-like	1.54	0.0260	up
IPI00685278	HNRNPF	Heterogeneous nuclear ribonucleoprotein F	1.31	0.0265	up
IPI00704977	－	Uncharacterized protein	1.49	0.0266	up
IPI00686966	HTRA2	Serine protease HTRA2, mitochondrial	1.24	0.0272	up
IPI00686841	SAMHD1	SAM domain and HD domain-containing protein 1	1.38	0.0289	up
IPI00696930	EFEMP1	EGF containing fibulin-like extracellular matrix protein 1	1.34	0.0290	up
IPI00700547	FBXO6	F-box only protein 6	1.38	0.0290	up

IPI00711233	NDUFB8	NADH dehydrogenase [ubiquinone] 1 beta subcomplex subunit 8, mitochondrial	1.22	0.0298	up
IPI00693324	EIF3J	Eukaryotic translation initiation factor 3 subunit J	1.26	0.0304	up
IPI00715967	THYN1	Thymocyte nuclear protein 1	1.24	0.0306	up
IPI00839134	NIPSNAP1	Nipsnap homolog 1 (C. elegans)	1.33	0.0312	up
IPI00693628	S100A2	Protein S100-A2	1.92	0.0315	up
IPI00695176	PSMB9	Proteasome subunit beta type-9	1.47	0.0323	up
IPI00695890	SSBP1	Single-stranded DNA-binding protein, mitochondrial	1.23	0.0331	up
IPI00690232	MAGOHB	Mago nashi homolog 2	1.31	0.0331	up
IPI00690308	PTRHD1	Putative peptidyl-tRNA hydrolase PTRHD1	1.26	0.0341	up
IPI00687372	ALCAM	CD166 antigen	1.30	0.0342	up
IPI00686225	HPRT1	Hypoxanthine-guanine phosphoribosyltransferase	1.51	0.0343	up
IPI00716497	CCDC93	LOC616839 protein	1.25	0.0346	up
IPI00706203	HEXB	Beta-hexosaminidase subunit beta preproprotein	1.31	0.0346	up
IPI00702620	SUMO1	Small ubiquitin-related modifier 1	1.44	0.0359	up
IPI00732368	UBQLN4	Ubiquilin 4	1.26	0.0370	up
IPI00715527	TSTD1	KAT protein-like	1.39	0.0377	up
IPI00708234	RCN3	Reticulocalbin-3	1.58	0.0378	up
IPI00687842	FTH1	Ferritin heavy chain	1.55	0.0384	up
IPI00716843	GNAO1	Guanine nucleotide-binding protein G(o) subunit alpha	1.22	0.0392	up
IPI00703753	ENPP1	Ectonucleotide pyrophosphatase/phosphodiesterase 1	1.49	0.0393	up
IPI00688651	CAMK2D	Calcium/calmodulin-dependent protein kinase type II subunit delta	1.48	0.0397	up
IPI00903569	OLFML3	Olfactomedin-like 3	1.62	0.0398	up
IPI00694126	NSUN2	NOP2/Sun domain family, member 2	1.39	0.0406	up
IPI00703854	NASP	Nuclear autoantigenic sperm protein	1.22	0.0410	up
IPI00718698	LTBP2	Latent transforming growth factor beta binding protein 2	1.30	0.0411	up
IPI00693338	PTPRC	Receptor-type tyrosine-protein phosphatase C	1.21	0.0422	up
IPI00686803	HRSP12	Ribonuclease UK114	1.30	0.0426	up
IPI00906505	LOC100299426	Uncharacterized protein	1.49	0.0444	up
IPI00721270	KRT14	Keratin, type I cytoskeletal 14	2.44	0.0453	up
IPI00912603	PAWR	PRKC apoptosis WT1 regulator protein-like	1.38	0.0457	up
IPI00687657	EVL	Enah/Vasp-like	1.26	0.0462	up
IPI00712677	NDUFB4	NADH dehydrogenase [ubiquinone] 1 beta subcomplex subunit 4	1.21	0.0494	up
IPI00710204	CD36	Platelet glycoprotein 4	0.75	0.0000	down
IPI00710664	LTF	Lactotransferrin	0.66	1.11E-16	down
IPI00843305	DYSF	Dysferlin	0.76	2.39E-14	down
IPI00708244	COL1A2	Collagen alpha-2(I) chain	0.65	3.25E-12	down
IPI00685653	SAA1	Serum amyloid A protein	0.18	3.25E-12	down
IPI00698916	FKBP1A	Peptidyl-prolyl cis-trans isomerase FKBP1A	0.73	3.26E-12	down
IPI00717930	ITIH4	Inter-alpha-trypsin inhibitor heavy chain H4	0.74	1.08E-11	down
IPI00709876	CAV1	Caveolin-1	0.77	6.79E-11	down
IPI00731432	COL3A1	Collagen, type III, alpha 1	0.72	5.67E-10	down
IPI00705441	CRP	C-reactive protein precursor	0.46	1.25E-08	down
IPI00707857	COL1A1	Collagen alpha-1(I) chain	0.73	1.67E-08	down
IPI00716157	LPO	Lactoperoxidase	0.71	2.24E-08	down
IPI01028182	GSTM3	Glutathione S-transferase mu 3	0.73	3.05E-08	down
IPI00708210	RPL10	60S ribosomal protein L10	0.78	6.95E-08	down
IPI00707670	ATP2C2	Calcium-transporting ATPase type 2C member 2-like	0.82	7.53E-08	down
IPI00713474	ARFGEF2	Uncharacterized protein	0.83	1.26E-07	down

IPI00699827	RPL4	60S ribosomal protein L4	0.64	2.14E-07	down
IPI00717048	RPL6	60S ribosomal protein L6	0.54	2.74E-07	down
IPI00694565	RPS9	40S ribosomal protein S9	0.66	3.13E-07	down
IPI00706037	HIP1R	KIAA0655 protein-like	0.82	7.02E-07	down
IPI00691086	SLC29A1	Equilibrative nucleoside transporter 1	0.75	8.19E-07	down
IPI00867237	QSOX1	QSOX1 protein	0.79	1.03E-06	down
IPI00715414	LOC100337199	ADP-ribosylation factor guanine nucleotide-exchange factor 2 (brefeldin A-inhibited)	0.79	1.06E-06	down
IPI00708597	ARL6IP5	PRA1 family protein 3	0.77	1.07E-06	down
IPI00827121	ABO	ABO blood group (Transferase A, alpha 1-3-N-acetylgalactosaminyltransferase	0.62	1.16E-06	down
IPI00692186	SLC4A1	Solute carrier family 4 (anion exchanger), member 1 (Diego blood group)	0.71	1.59E-06	down
IPI00705159	ATP1A1	Sodium/potassium-transporting ATPase subunit alpha-1	0.82	2.25E-06	down
IPI00825680	HERC6	HECT and RLD domain containing E3 ubiquitin protein ligase family member 6	0.81	2.39E-06	down
IPI00702948	RPS6	40S ribosomal protein S6	0.78	3.31E-06	down
IPI00708246	QARS	Glutaminyl-tRNA synthetase	0.82	5.28E-06	down
IPI00696488	LOC784931	HCG1994130-like	0.68	5.55E-06	down
IPI00829520	RAB1A	RAB1A, member RAS oncogene family	0.81	9.03E-06	down
IPI00717764	CHI3L1	Chitinase-3-like protein 1	0.56	1.04E-05	down
IPI00694831	－	Uncharacterized protein	0.68	1.18E-05	down
IPI00707386	RPL18A	60S ribosomal protein L18a	0.69	1.18E-05	down
IPI00718379	RPL18	60S ribosomal protein L18	0.67	1.25E-05	down
IPI00714325	LOC508439	Uncharacterized protein	0.78	1.45E-05	down
IPI00715868	RPL21	Uncharacterized protein (Fragment)	0.58	1.50E-05	down
IPI00689629	FKBP11	Peptidyl-prolyl cis-trans isomerase FKBP11	0.80	1.71E-05	down
IPI00691826	RAB18	Ras-related protein Rab-18	0.78	2.55E-05	down
IPI00708535	BTN1A1	Butyrophilin subfamily 1 member A1	0.80	3.21E-05	down
IPI00695037	CDK5RAP3	CDK5 regulatory subunit associated protein 3	0.77	3.44E-05	down
IPI00699226	ATP2B4	ATPase, Ca++ transporting, plasma membrane 4	0.75	4.64E-05	down
IPI00705739	RPL13	60S ribosomal protein L13	0.60	4.77E-05	down
IPI00716725	PACSIN3	Protein kinase C and casein kinase substrate in neurons 3	0.60	6.61E-05	down
IPI00703084	GPAM	Glycerol-3-phosphate acyltransferase 1, mitochondrial	0.70	7.18E-05	down
IPI01000395	INADL	InaD-like	0.76	7.44E-05	down
IPI00694688	RPS26	40S ribosomal protein S26	0.74	7.49E-05	down
IPI00688878	DHRS7B	Dehydrogenase/reductase SDR family member 7B	0.75	7.88E-05	down
IPI00706142	CAND1	Cullin-associated NEDD8-dissociated protein 1	0.82	8.45E-05	down
IPI00710834	STOM	STOM protein	0.83	9.63E-05	down
IPI00718152	RAB11B	Ras-related protein Rab-11B	0.82	0.0001	down
IPI00712737	EPM2AIP1	EPM2A (laforin) interacting protein 1	0.78	0.0001	down
IPI00690620	ATAD3A	ATPase family AAA domain-containing protein 3	0.83	0.0001	down
IPI00717758	ACSF2	Acyl-CoA synthetase family member 2, mitochondrial	0.75	0.0001	down
IPI00708070	PKP2	PKP2 protein	0.76	0.0001	down
IPI00695042	RRAS	Related RAS viral (r-ras) oncogene homolog	0.80	0.0001	down
IPI00712384	RPL13A	60S ribosomal protein L13a	0.62	0.0002	down
IPI00867017	DARS2	Aspartyl-tRNA synthetase, mitochondrial	0.66	0.0002	down
IPI00691093	－	Uncharacterized protein	0.73	0.0002	down
IPI00708085	TSPAN13	Tetraspanin-13	0.81	0.0002	down
IPI00685769	FLOT2	Flotillin-2	0.77	0.0002	down
IPI00704078	AKR1C4	Dihydrodiol dehydrogenase 3	0.77	0.0002	down

IPI00871114	AASS	Alpha-aminoadipic semialdehyde synthase, mitochondrial	0.83	0.0003	down
IPI00712263	PTBP1	Polypyrimidine tract-binding protein 1	0.57	0.0003	down
IPI00713979	CDH5	Cadherin-5	0.80	0.0003	down
IPI00688977	PDHX	Pyruvate dehydrogenase protein X component	0.83	0.0003	down
IPI00686872	ATP5G2	ATP synthase lipid-binding protein, mitochondrial	0.60	0.0003	down
IPI00713049	RASIP1	Ras interacting protein 1	0.76	0.0004	down
IPI00697798	HIST1H1A	Histone cluster 1, H1a-like	0.50	0.0004	down
IPI00710859	RPL19	60S ribosomal protein L19	0.52	0.0004	down
IPI00713313	F11R	Junctional adhesion molecule A	0.82	0.0004	down
IPI00699470	VPS53	Vacuolar protein sorting 53 homolog (S. cerevisiae)	0.73	0.0004	down
IPI00716220	MUC15 I	Soform 1 of Mucin-15	0.75	0.0004	down
IPI00706314	SLC25A4	ADP/ATP translocase 1	0.83	0.0005	down
IPI00688337	SNRPG	Small nuclear ribonucleoprotein G	0.81	0.0005	down
IPI00706634	ADCK3	Chaperone activity of bc1 complex-like, mitochondrial	0.79	0.0005	down
IPI00697917	MID1	Midline 1	0.82	0.0006	down
IPI00705565	GGT1	Gamma-glutamyltranspeptidase 1	0.80	0.0006	down
IPI00730219	SLC25A12	Solute carrier family 25 (aspartate/glutamate carrier), member 12	0.73	0.0006	down
IPI00712858	SLC25A5	ADP/ATP translocase 2	0.77	0.0006	down
IPI00716860	RPL36	60S ribosomal protein L36	0.78	0.0006	down
IPI00718678	HUWE1	HECT, UBA and WWE domain containing 1, E3 ubiquitin protein ligase	0.82	0.0006	down
IPI00841549	CROCC	Ciliary rootlet coiled-coil, rootletin	0.81	0.0007	down
IPI01028041	BTN1A1	38 kDa protein	0.77	0.0007	down
IPI00826022	－	Uncharacterized protein	0.50	0.0008	down
IPI00712596	EPHX1	Epoxide hydrolase 1, microsomal (xenobiotic)	0.73	0.0008	down
IPI00692739	TMED2	Transmembrane emp24 domain trafficking protein 2	0.62	0.0009	down
IPI00705378	SLC25A6	ADP/ATP translocase 3	0.74	0.0010	down
IPI00713460	GOSR1	Golgi SNAP receptor complex member 1	0.75	0.0010	down
IPI00709888	ATP2C1	Calcium-transporting ATPase type 2C member 1	0.80	0.0010	down
IPI00690355	CLEC14A	C-type lectin domain family 14, member A	0.76	0.0011	down
IPI00707537	RPL28	60S ribosomal protein L28	0.64	0.0012	down
IPI00698339	H2AFY	Histone H2A	0.79	0.0012	down
IPI00689226	NUP93	Nuclear pore complex protein Nup93	0.81	0.0013	down
IPI01003286	CUX1	Cut-like homeobox 1	0.80	0.0013	down
IPI00698213	MYBBP1A	MYB binding protein (P160) 1a	0.79	0.0014	down
IPI00935131	PHF1	PHD finger protein 1	0.67	0.0015	down
IPI00707455	TRAP1	Heat shock protein 75 kDa, mitochondrial	0.78	0.0016	down
IPI00698077	RPL8	60S ribosomal protein L8	0.67	0.0016	down
IPI00694444	RPL27A	60S ribosomal protein L27a	0.72	0.0017	down
IPI00693452	ORAI1	ORAI calcium release-activated calcium modulator 1	0.79	0.0017	down
IPI00714098	NEK9	Serine/threonine-protein kinase Nek9	0.82	0.0017	down
IPI00687634	RPL3	60S ribosomal protein L3	0.56	0.0018	down
IPI00692668	RAB3GAP1	RAB3 GTPase activating protein subunit 1 (catalytic)	0.81	0.0019	down
IPI00689641	H1FX	H1 histone family, member X-like	0.69	0.0019	down
IPI00727050	RAB1B	Ras-related protein Rab-1B	0.80	0.0020	down
IPI01000483	GNA13	Guanine nucleotide binding protein, alpha 12-like	0.81	0.0020	down
IPI00715640	HM13	Minor histocompatibility antigen 13	0.74	0.0020	down
IPI00687395	PRPF8	pre-mRNA processing factor 8	0.81	0.0021	down
IPI00689899	LOC100297610	CG10465-like	0.82	0.0022	down
IPI00692963	SEC23A	Protein transport protein Sec23A	0.78	0.0023	down

IPI00712399	H2AFX	Histone H2A	0.70	0.0024	down
IPI00694581	OCIAD2	OCIA domain-containing protein 2	0.82	0.0025	down
IPI00876781	C8G	Complement component 8, gamma polypeptide	0.71	0.0029	down
IPI00689365	AARS2	Probable alanyl-tRNA synthetase, mitochondrial	0.71	0.0029	down
IPI00876805	DMD	DMD protein	0.77	0.0030	down
IPI00687478	GLRX	Glutaredoxin-1	0.77	0.0030	down
IPI00714577	TCEAL4	Transcription elongation factor A (SII)-like 4 isoform 1	0.79	0.0030	down
IPI00694071	NBAS	Neuroblastoma amplified sequence	0.75	0.0033	down
IPI00688727	RPS13	40S ribosomal protein S13	0.73	0.0033	down
IPI01017508	DIS3	DIS3 protein	0.81	0.0034	down
IPI00699372	ATP6V0A1	Isoform 1 of V-type proton ATPase 116 kDa subunit a isoform 1	0.74	0.0036	down
IPI00703925	ZFYVE1	Zinc finger, FYVE domain containing 1	0.74	0.0036	down
IPI00694758	PAK4	p21 protein (Cdc42/Rac)-activated kinase 4	0.31	0.0037	down
IPI00727591	HLCS	Hypothetical protein	0.58	0.0038	down
IPI01000984	LOC789637	Multidrug resistance-associated protein 4 splice-like, partial	0.53	0.0038	down
IPI00690581	SELS	Selenoprotein S	0.75	0.0040	down
IPI00689556	CD300LG	CD300 molecule-like family member g isoform 2	0.79	0.0046	down
IPI00715174	APCS	Serum amyloid P-component	0.74	0.0047	down
IPI00708037	STON2	Stonin-2	0.67	0.0049	down
IPI00716830	VAPA	Vesicle-associated membrane protein-associated protein A	0.63	0.0049	down
IPI00704332	COASY	CoA synthase	0.78	0.0049	down
IPI00708162	GOLGA5	Golgin A5	0.83	0.0050	down
IPI00854492	CD177	CD177 protein	0.56	0.0050	down
IPI00760457	GLUL	Glutamine synthetase	0.78	0.0053	down
IPI00702768	ALPL	Alkaline phosphatase, tissue-nonspecific isozyme	0.79	0.0053	down
IPI00699039	HS2ST1	Uncharacterized protein	0.83	0.0054	down
IPI00687662	LOC784384	Uncharacterized protein	0.47	0.0055	down
IPI00839150	PNPLA6	Neuropathy target esterase	0.73	0.0060	down
IPI00707776	CARM1	Uncharacterized protein (Fragment)	0.76	0.0060	down
IPI00714056	ELMO1	Engulfment and cell motility 1 isoform 1	0.81	0.0062	down
IPI00714269	KPNA1 I	Mportin subunit alpha-1	0.67	0.0062	down
IPI00695852	ERGIC1	Endoplasmic reticulum-golgi intermediate compartment 1	0.83	0.0063	down
IPI00694094	FOXRED1	Isoform 1 of FAD-dependent oxidoreductase domain-containing protein 1	0.56	0.0064	down
IPI00715601	NAA25	N(alpha)-acetyltransferase 25, NatB auxiliary subunit	0.67	0.0066	down
IPI01028455	ALB	Serum albumin	0.28	0.0069	down
IPI00866975	PICALM	PICALM protein	0.67	0.0070	down
IPI00685147	LRRC8C	Leucine-rich repeat-containing protein 8C	0.81	0.0070	down
IPI00697164	OVCA2	Ovarian cancer-associated gene 2 protein homolog	0.49	0.0071	down
IPI01000962	LOC100336729	CG8379-like	0.82	0.0071	down
IPI00707685	–	Serpin A3-8	0.69	0.0072	down
IPI00708767	ARPC4	Actin-related protein 2/3 complex subunit 4	0.70	0.0073	down
IPI00690390	LOC789375	Uncharacterized protein	0.71	0.0074	down
IPI00704655	LOC531049	Uncharacterized protein	0.75	0.0076	down
IPI00711463	VPS52	Vacuolar protein sorting 52 homolog (S. cerevisiae)	0.83	0.0077	down
IPI00854399	SLC44A3	Solute carrier family 44, member 3	0.71	0.0078	down
IPI00713520	BTNL9	Butyrophilin-like 9	0.66	0.0081	down

IPI00714660	STX4	Syntaxin-4	0.83	0.0086	down
IPI01000210	FARP1	FERM, RhoGEF and pleckstrin domain-containing protein 1	0.71	0.0088	down
IPI00697349	MFSD10	Major facilitator superfamily domain-containing protein 10	0.82	0.0090	down
IPI00689165	ST14	Suppressor of tumorigenicity 14 protein homolog	0.80	0.0092	down
IPI00694597	LOC510193	Uncharacterized protein	0.78	0.0092	down
IPI00710920	SLC12A2	Solute carrier family 12 (sodium/potassium/chloride transporter), member 2	0.80	0.0094	down
IPI00704226	TTC39A	Tetratricopeptide repeat domain 39A-like	0.82	0.0095	down
IPI00687375	TIMP3	Metalloproteinase inhibitor 3	0.63	0.0095	down
IPI00845178	MCC	MCC protein	0.74	0.0095	down
IPI00700228	DDX39A	DEAD (Asp-Glu-Ala-Asp) box polypeptide 39A	0.73	0.0095	down
IPI00866955	TMEM120B	Transmembrane protein 120B	0.77	0.0097	down
IPI00904814	MRPL38	Mitochondrial ribosomal protein L38	0.54	0.0099	down
IPI00715066	BCAT2	Branched-chain-amino-acid aminotransferase, mitochondrial	0.79	0.0100	down
IPI00702151	GSTM1	Glutathione S-transferase Mu 1	0.83	0.0100	down
IPI00708654	LGALS7B	Lectin, galactoside-binding, soluble, 7B	0.80	0.0103	down
IPI00696456	CASKIN2	CASK interacting protein 2	0.60	0.0104	down
IPI00705583	LGALS8	Lectin, galactoside-binding, soluble, 8	0.55	0.0107	down
IPI00867311	SCAMP2	Secretory carrier membrane protein 2	0.82	0.0108	down
IPI00840437	LOC533324	Uncharacterized protein	0.72	0.0110	down
IPI00691649	L2HGDH	L-2-hydroxyglutarate dehydrogenase, mitochondrial	0.74	0.0111	down
IPI00714621	C16H1orf93	Prostamide/prostaglandin F synthase	0.59	0.0113	down
IPI00696116	VAMP2	Vesicle-associated membrane protein 2	0.41	0.0114	down
IPI00710274	NF2	Neurofibromin 2 (merlin)	0.82	0.0115	down
IPI00709124	ENDOG	Endonuclease G, mitochondrial	0.54	0.0122	down
IPI00847122	RAP2B	Member of RAS oncogene family	0.74	0.0134	down
IPI00700622	SERPINA3-1	Serpin A3-1	0.69	0.0135	down
IPI00908033	RAB24	Member of RAS oncogene family	0.77	0.0136	down
IPI00687839	FKBP8	FK506 binding protein 8, 38kDa	0.67	0.0141	down
IPI00701747	LOC100140923	Uncharacterized protein	0.75	0.0141	down
IPI00694390	GNE	Bifunctional UDP-N-acetylglucosamine 2-epimerase/N-acetylmannosamine kinase	0.78	0.0142	down
IPI00708824	CD47	Leukocyte surface antigen CD47	0.79	0.0145	down
IPI00707815	AIP	AH receptor-interacting protein	0.71	0.0150	down
IPI00852474	NAT10	NAT10 protein	0.69	0.0150	down
IPI00688763	LOC530677	Histone cluster 1, H2bd-like	0.64	0.0154	down
IPI00903861	TMEM111	ER membrane protein complex subunit 3), a synonym of EMC3	0.42	0.0155	down
IPI00693824	RAB5A	Ras-related protein Rab-5A	0.73	0.0157	down
IPI00704902	CNOT7	CCR4-NOT transcription complex subunit 7	0.74	0.0157	down
IPI00711457	MCU	Coiled-coil domain-containing protein 109A	0.79	0.0159	down
IPI00685695	HSPA13	Heat shock 70 kDa protein 13	0.76	0.0160	down
IPI00734257	STAG1	Stromal antigen 1 isoform 1	0.71	0.0160	down
IPI00711074	MYADM	Myeloid-associated differentiation marker	0.61	0.0161	down
IPI00710377	ATP13A4	Cation-transporting P5-ATPase-like	0.61	0.0165	down
IPI00698823	NPC1	Niemann-Pick type C1 disease protein	0.74	0.0169	down
IPI00709542	LOC100335281	CD82 Uncharacterized protein	0.79	0.0170	down
IPI00695952	COQ5	Coenzyme Q5 homolog, methyltransferase (S. cerevisiae)	0.77	0.0174	down

IPI00714898	LOC100335825	Solute carrier family 30 (zinc transporter), member 9-like	0.75	0.0175	down
IPI00688980	ARL15	ADP-ribosylation factor-like protein 15	0.66	0.0179	down
IPI00695548	DIP2B	Disco-interacting protein 2 homolog B	0.82	0.0186	down
IPI00883285	TRAF3IP3	TRAF3 interacting protein 3 isoform 1	0.83	0.0187	down
IPI00713597	CSPG4	Chondroitin sulfate proteoglycan 4	0.61	0.0187	down
IPI00704041	CUL5	Cullin 5	0.77	0.0196	down
IPI00715606	EHBP1L1	EH domain binding protein 1-like 1	0.81	0.0199	down
IPI00705933	CHMP6	Charged multivesicular body protein 6	0.66	0.0201	down
IPI00867230	CSTB	Cystatin B (stefin B)	0.66	0.0204	down
IPI00838432	USE1	Unconventional SNARE in the ER 1 homolog	0.65	0.0209	down
IPI00838420	P4HA2	Prolyl 4-hydroxylase subunit alpha-2 isoform 2	0.80	0.0210	down
IPI00688704	NLE1	Notchless protein homolog 1	0.33	0.0213	down
IPI00710277	SPINT2	Serine peptidase inhibitor, Kunitz type, 2	0.79	0.0213	down
IPI00692893	TM4SF18	Transmembrane 4 L6 family member 18	0.78	0.0216	down
IPI00687578	PCNT	Pericentrin	0.81	0.0218	down
IPI00694038	C2CD2	C2 domain-containing protein 2	0.81	0.0224	down
IPI00700471	KRT75	Keratin, type II cytoskeletal 75	0.73	0.0227	down
IPI00688474	TTC19	Tetratricopeptide repeat domain 19	0.65	0.0235	down
IPI00693579	DIS3L2	DIS3-like exonuclease 2	0.80	0.0238	down
IPI00710996	EPS8L2	EPS8-like 2	0.78	0.0249	down
IPI00734394	LOC617150	ATP-binding cassette protein C4-like	0.79	0.0251	down
IPI00906031	PPP1CC	Serine/threonine-protein phosphatase PP1-gamma catalytic subunit	0.81	0.0252	down
IPI00705237	PKP4	Plakophilin 4	0.77	0.0252	down
IPI00703954	ARL6IP1	ADP-ribosylation factor-like 6 interacting protein 1	0.72	0.0255	down
IPI01028468	XPO4	Exportin-4	0.70	0.0258	down
IPI00697595	ITGA1	Integrin, alpha 1	0.83	0.0258	down
IPI00686595	SF3B1	Splicing factor 3B subunit 1	0.83	0.0261	down
IPI00712288	DAAM1	Dishevelled associated activator of morphogenesis 1	0.81	0.0263	down
IPI00708946	BDA20	Allergen Bos d 2	0.42	0.0263	down
IPI00717110	PGRMC1	Membrane-associated progesterone receptor component 1	0.70	0.0267	down
IPI00725840	DOCK11	Dedicator of cytokinesis 11	0.67	0.0273	down
IPI00703022	FTSJD2	Cap-specific mRNA (nucleoside-2~-O-)-methyltransferase 1	0.55	0.0274	down
IPI00924039	ANTXR1	Anthrax toxin receptor 1	0.76	0.0277	down
IPI01017588	PPP2R5D	Serine/threonine-protein phosphatase 2A 56 kDa regulatory subunit delta isoform	0.67	0.0282	down
IPI01003645	LAMB2	Laminin, beta 2	0.80	0.0282	down
IPI00698059	MAOA	Amine oxidase [flavin-containing] A	0.82	0.0282	down
IPI00706780	CISD3	CDGSH iron sulfur domain 3-like	0.71	0.0284	down
IPI00693842	YLPM1	YLP motif containing 1	0.73	0.0287	down
IPI00841259	RAB3GAP2	Rab3 GTPase-activating protein non-catalytic subunit	0.83	0.0288	down
IPI00702251	AMACR	Alpha-methylacyl-CoA racemase	0.81	0.0292	down
IPI00706879	AGPAT1	1-acyl-sn-glycerol-3-phosphate acyltransferase alpha	0.81	0.0295	down
IPI00714656	SLC2A1	Solute carrier family 2, facilitated glucose transporter member 1	0.76	0.0303	down
IPI00687661	CDIPT	CDP-diacylglycerol--inositol 3-phosphatidyltransferase	0.78	0.0307	down
IPI00711069	LOC790189	Uncharacterized protein (Fragment)	0.65	0.0308	down
IPI00689044	LRRC57	Leucine rich repeat containing 57	0.81	0.0308	down
IPI00685678	NQO1	NAD(P)H dehydrogenase, quinone 1	0.75	0.0310	down
IPI00854393	SLC4A7	Solute carrier family 4, sodium bicarbonate cotransporter, member 7	0.59	0.0311	down

IPI00712524	COL4A2	Collagen, type IV, alpha 2, partial	0.78	0.0326	down
IPI00709409	RPL15	60S ribosomal protein L15	0.81	0.0331	down
IPI00697194	FDXR	Isoform Short of NADPH:adrenodoxin oxidoreductase, mitochondrial	0.77	0.0338	down
IPI00689143	METTL9	Methyltransferase-like protein 9	0.66	0.0340	down
IPI00703813	SLC34A2	Sodium-dependent phosphate transport protein 2B	0.81	0.0347	down
IPI00696748	BCCIP	BRCA2 and CDKN1A-interacting protein	0.75	0.0351	down
IPI00903807	MFF	Uncharacterized protein	0.65	0.0351	down
IPI00685617	CD81	CD81 antigen	0.79	0.0356	down
IPI00707673	NUP205	Nucleoporin 205kDa	0.77	0.0358	down
IPI00852533	SLC44A2	Choline transporter-like protein 2	0.68	0.0359	down
IPI00815564	FAM65A	Hypothetical protein	0.78	0.0368	down
IPI00699817	EXTL3	EXTL3 protein	0.74	0.0370	down
IPI00687906	FUNDC2	FUN14 domain-containing protein 2	0.83	0.0374	down
IPI00689565	RPL17	60S ribosomal protein L17	0.73	0.0376	down
IPI00715059	IRS1	Insulin receptor substrate 1	0.62	0.0387	down
IPI00694430	MLYCD	Malonyl-CoA decarboxylase	0.77	0.0390	down
IPI00690789	MVD	Diphosphomevalonate decarboxylase	0.69	0.0396	down
IPI00690309	MTOR	Mechanistic target of rapamycin (serine/threonine kinase	0.83	0.0403	down
IPI00692417	EXOC3	Exocyst complex component 3	0.78	0.0403	down
IPI01000079	LOC100336406	GMP reductase 1-like	0.74	0.0414	down
IPI00689194	EXOC2	Exocyst complex component 2	0.79	0.0417	down
IPI00705104	H1F0	Histone H1.0	0.28	0.0419	down
IPI00694198	RAB5C	Ras-related protein Rab-5C	0.79	0.0446	down
IPI00700673	SAA4	Serum amyloid A-4 protein	0.81	0.0448	down
IPI01002995	LOC100337431	Solute carrier family 12, member 7-like	0.71	0.0449	down
IPI00689379	CAPN6	Calpain 6	0.76	0.0454	down
IPI00688400	ANXA9	Annexin A9	0.62	0.0463	down
IPI00697648	ACTA1	ACTC1 Actin, alpha skeletal muscle	0.24	0.0465	down
IPI00688446	CPSF2	Cleavage and polyadenylation specificity factor subunit 2	0.67	0.0469	down
IPI00713084	LOC512120	KIAA1467-like	0.75	0.0474	down
IPI00685510	APP	Amyloid beta (A4) protein	0.74	0.0477	down
IPI00685804	SEC11A	Signal peptidase complex catalytic subunit SEC11A	0.79	0.0486	down
IPI00708601	ARHGEF7	Rho guanine nucleotide exchange factor (GEF) 7	0.83	0.0486	down
IPI00697413	KRTCAP3	Keratinocyte-associated protein 3	0.80	0.0491	down
IPI00866858	ARHGEF16	Rho guanine nucleotide exchange factor (GEF) 16	0.78	0.0496	down
IPI00688451	ASAP3	ArfGAP with SH3 domain, ankyrin repeat and PH domain 3	0.80	0.0499	down

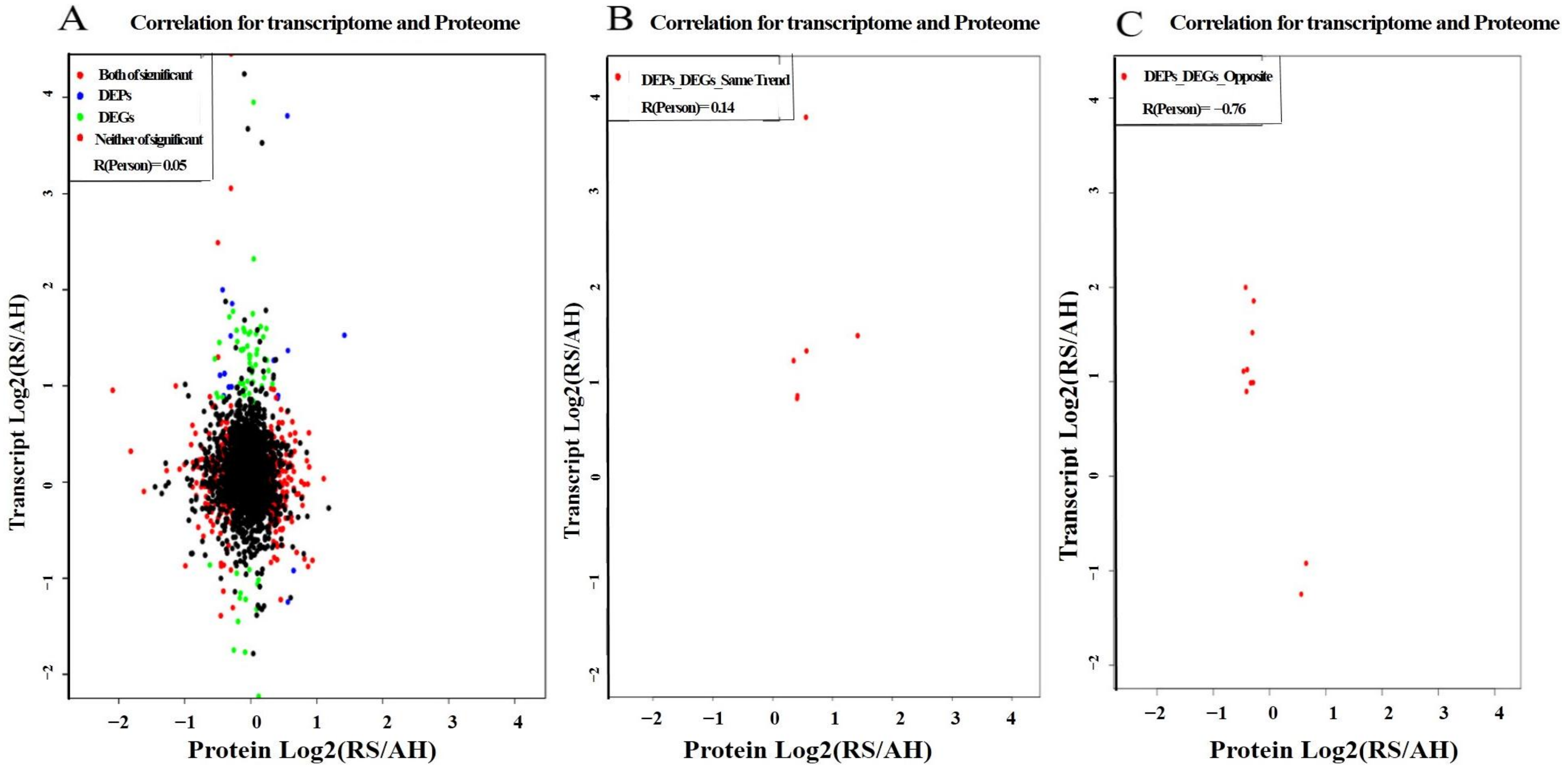

A
Correlation for transcriptome and Proteome
Both of significant
DEPs
DEGs
Neither of significant
R(Person)= 0.05
Transcript Log2(RS/AH)
Protein Log2(RS/AH)
B
Correlation for transcriptome and Proteome
DEPs_DEGs_Same Trend
R(Person)= 0.14
Transcript Log2(RS/AH)
Protein Log2(RS/AH)
C
Correlation for transcriptome and Proteome
DEPs_DEGs_Opposite
R(Person)= −0.76
Transcript Log2(RS/AH)
Protein Log2(RS/AH)

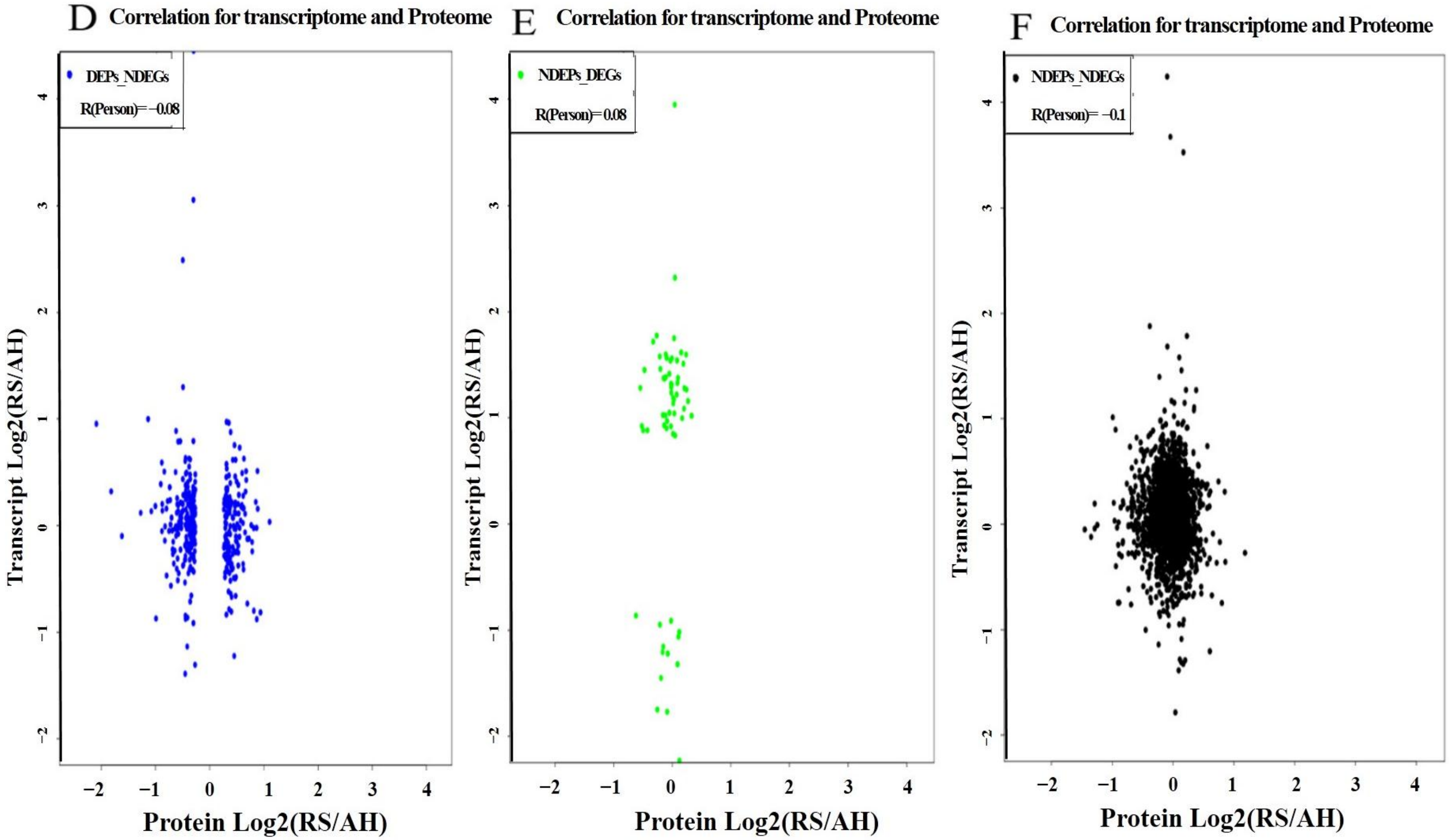

Figure S1. Relationship between the transcripts and proteins in mammary glands between RS- and AH-fed dairy cows. The correlation of the whole transcriptome and the corresponding proteome was showed (Fig. S1A), the red points are those which were significant for both of the two datasets of the same expression patterns (Fig. S1B) and the opposite expression patterns (Fig. S1C), the green points are those which were significant only for transcripts (Fig.S1D), the blue points are those which were significant only for proteins (Fig. S1E) and the black points are those which were not significant in either of the two datasets (Fig. S1F).

Table S4 Comparison of GO enrichment of the differentially expressed transcripts and proteins in mammary glands between RS- and AH-fed dairy cows

**GO id**	**GO description**	**GO class**	**NO.of mapping transcripts**	**Transcripts (%)**	**NO.of mapping proteins**	**Proteins (%)**
GO:0000003	Reproduction	Biological Process	14	3.8	14	3.1
GO:0032502	Developmental process	Biological Process	87	23.6	106	23.2
GO:0044085	Cellular component biogenesis	Biological Process	27	7.3	53	11.6
GO:0016043	Cellular component organization	Biological Process	74	20.1	110	24.1
GO:0016265	Death	Biological Process	31	8.4	33	7.2
GO:0022414	Reproductive process	Biological Process	14	3.8	14	3.1
GO:0002376	Immune system process	Biological Process	33	8.9	33	7.2
GO:0050896	Response to stimulus	Biological Process	74	20.1	132	28.9
GO:0032501	Multicellular organismal process	Biological Process	104	28.2	126	27.6
GO:0010926	Anatomical structure formation	Biological Process	39	10.6	65	14.2
GO:0051704	Multi-organism process	Biological Process	17	4.6	15	3.3
GO:0051234	Establishment of localization	Biological Process	66	17.9	102	22.3
GO:0022610	Biological adhesion	Biological Process	19	5.1	28	6.1
GO:0008152	Metabolic process	Biological Process	197	53.4	231	50.5
GO:0016032	Viral reproduction	Biological Process	0	0	2	0.4
GO:0048511	Rhythmic process	Biological Process	5	1.4	0	0
GO:0043473	Pigmentation	Biological Process	148	40.1	167	36.5
GO:0040011	Locomotion	Biological Process	21	5.7	22	4.8
GO:0051179	Localization	Biological Process	85	23	125	27.4
GO:0040007	Growth	Biological Process	13	3.5	13	2.8
GO:0009987	Cellular process	Biological Process	263	71.3	324	70.9
GO:0001906	Cell killing	Biological Process	0	0	2	0.4
GO:0065007	Biological regulation	Biological Process	160	43.4	179	39.2
GO:0005576	Extracellular region	Cellular Component	45	12.2	77	16.8
GO:0044421	Extracellular region part	Cellular Component	32	8.7	55	12
GO:0005623	Cell	Cellular Component	270	73.2	373	81.6
GO:0044464	Cell part	Cellular Component	270	73.2	373	81.6
GO:0031974	Membrane-enclosed lumen	Cellular Component	24	6.5	54	11.8
GO:0031975	Envelope	Cellular Component	16	4.3	34	7.4
GO:0032991	Macromolecular complex	Cellular Component	58	15.7	134	29.3
GO:0043226	Organelle	Cellular Component	184	49.9	278	60.8
GO:0044422	Organelle part	Cellular Component	92	24.9	173	37.9
GO:0045202	Synapse	Cellular Component	3	0.8	9	2
GO:0044456	Synapse part	Cellular Component	2	0.5	4	0.9
GO:0009055	Electron carrier activity	Molecular Function	3	0.8	4	0.9
GO:0060089	Molecular transducer activity	Molecular Function	26	7	12	2.6
GO:0030528	Transcription regulator activity	Molecular Function	18	4.9	13	2.8

GO:0030234	Enzyme regulator activity	Molecular Function	21	5.7	29	6.3
GO:0003824	Catalytic activity	Molecular Function	120	32.5	140	30.6
GO:0005488	Binding	Molecular Function	295	79.9	213	46.6
GO:0016209	Antioxidant activity	Molecular Function	1	0.3	6	1.3
GO:0015457	Auxiliary transport protein activity	Molecular Function	0	0	1	0.2
GO:0045499	Chemorepellent activity	Molecular Function	1	0.3	0	0
GO:0045182	Translation regulator activity	Molecular Function	1	0.3	4	0.9
GO:0005198	Structural molecule activity	Molecular Function	13	3.5	47	0
GO:0042056	Chemoattractant activity	Molecular Function	1	0.3	0	0
GO:0005215	Transporter activity	Molecular Function	22	6	28	6.1

Table S5 GO categories assigned to the differentially up-regulated transcripts in mammary glands between RS and AH dairy cows

GO id	Gene list	GO term	GO description	GO Class	S gene number	TS gene number	P-value of Fisher's Exact Test
GO:0045749	XLOC_024038	Biological Process	Negative regulation of S phase of mitotic cell cycle	1	1	287	0.1511
GO:0051925	XLOC_023978	Biological Process	Regulation of calcium ion transport via voltage-gated calcium channel activity	1	1	287	0.3496
GO:0032851	XLOC_009333;XLOC_023491;XLOC_023598;XLOC_023863	Biological Process	Positive regulation of Rab GTPase activity	17	4	287	0.0138
GO:0090267	XLOC_023439	Biological Process	Positive regulation of mitotic cell cycle spindle assembly checkpoint	17	1	287	0.1156
GO:0032313	XLOC_009333;XLOC_023491;XLOC_023598;XLOC_023863	Biological Process	Regulation of Rab GTPase activity	16	4	287	0.0149
GO:0030513	XLOC_002956;XLOC_003605;XLOC_023581	Biological Process	Positive regulation of BMP signaling pathway	15	3	287	0.0136
GO:0072277	XLOC_023651	Biological Process	Metanephric glomerular capillary formation	15	1	287	0.0596
GO:0060564	XLOC_023439	Biological Process	Negative regulation of mitotic anaphase-promoting complex activity	15	1	287	0.0786
GO:0043547	XLOC_002772;XLOC_016622;XLOC_023598;XLOC_023863	Biological Process	Positive regulation of GTPase activity	15	4	287	0.0939
GO:0006977	XLOC_022857	Biological Process	DNA damage response, signal transduction by p53 class mediator resulting in cell cycle arrest	15	1	287	0.2017
GO:0072264	XLOC_023297	Biological Process	Metanephric glomerular endothelium development	14	1	287	0.0401
GO:0003256	XLOC_023581	Biological Process	Regulation of transcription from RNA polymerase II promoter involved in myocardial precursor cell differentiation	14	1	287	0.0973
GO:0007221	XLOC_023581	Biological Process	Positive regulation of transcription of Notch receptor target	14	1	287	0.0973
GO:0031659	XLOC_023978	Biological Process	Positive regulation of cyclin-dependent protein serine/threonine kinase activity involved in G1/S transition of mitotic cell cycle	14	1	287	0.1511
GO:0006098	XLOC_024273	Biological Process	Pentose-phosphate shunt	14	1	287	0.2645
GO:0030819	XLOC_008893	Biological Process	Positive regulation of cAMP biosynthetic process	14	1	287	0.3224
GO:0051209	XLOC_023978	Biological Process	Release of sequestered calcium ion into cytosol	14	1	287	0.3628
GO:0043507	XLOC_024021	Biological Process	Positive regulation of JUN kinase activity	14	1	287	0.4366

GO:0032088	XLOC_020726	Biological Process	Negative regulation of NF-kappaB transcription factor activity	14	1	287	0.6106
GO:0043406	XLOC_023297;XLOC_023444;XLOC_023653	Biological Process	Positive regulation of MAP kinase activity	13	3	287	0.0223
GO:0003104	XLOC_023297	Biological Process	Positive regulation of glomerular filtration	13	1	287	0.0596
GO:0035793	XLOC_023297	Biological Process	Positive regulation of metanephric mesenchymal cell migration by platelet-derived growth factor receptor-beta signaling pathway	13	1	287	0.0596
GO:2001171	XLOC_023992	Biological Process	Positive regulation of ATP biosynthetic process	13	1	287	0.0596
GO:0051091	XLOC_023653;XLOC_023756;XLOC_023992	Biological Process	Positive regulation of sequence-specific DNA binding transcription factor activity	13	3	287	0.0768
GO:0072284	XLOC_023978	Biological Process	Metanephric S-shaped body morphogenesis	13	1	287	0.0786
GO:0030814	XLOC_023978	Biological Process	Regulation of cAMP metabolic process	13	1	287	0.1156
GO:0045944	XLOC_002509;XLOC_002956;XLOC_003350;XLOC_003605;XLOC_009333;XLOC_023512;XLOC_023581;XLOC_023621;XLOC_023762;XLOC_023933;XLOC_023941;XLOC_023978;XLOC_023992;XLOC_024194;XLOC_024254	Biological Process	Positive regulation of transcription from RNA polymerase II promoter	13	15	287	0.1309
GO:0030071	XLOC_023568	Biological Process	Regulation of mitotic metaphase/anaphase transition	13	1	287	0.1511
GO:0010842	XLOC_005548	Biological Process	Retina layer formation	13	1	287	0.1683
GO:0003222	XLOC_023581	Biological Process	Ventricular trabecula myocardium morphogenesis	13	1	287	0.1852
GO:0021756	XLOC_024239	Biological Process	Striatum development	13	1	287	0.1852
GO:0045737	XLOC_023297	Biological Process	Positive regulation of cyclin-dependent protein serine/threonine kinase activity	13	1	287	0.1852
GO:0032148	XLOC_023297	Biological Process	Activation of protein kinase B activity	13	1	287	0.2179
GO:0043433	XLOC_012264;XLOC_018228	Biological Process	Negative regulation of sequence-specific DNA binding transcription factor activity	13	2	287	0.2314
GO:0021772	XLOC_023558	Biological Process	Olfactory bulb development	13	1	287	0.2337
GO:0043536	XLOC_023297	Biological Process	Positive regulation of blood vessel endothelial cell migration	13	1	287	0.3083
GO:0030509	XLOC_013257	Biological Process	BMP signaling pathway	13	1	287	0.5504
GO:0000187	XLOC_023653	Biological Process	Activation of MAPK activity	13	1	287	0.6486

GO:0032436	XLOC_013557;XLOC_022857;XLOC_024155	Biological Process	Positive regulation of proteasomal ubiquitin-dependent protein catabolic process	12	3	287	0.0310
GO:0034401	XLOC_024273	Biological Process	Chromatin organization involved in regulation of transcription	12	1	287	0.0401
GO:0046709	XLOC_023788	Biological Process	IDP catabolic process	12	1	287	0.0401
GO:0061441	XLOC_023978	Biological Process	Renal artery morphogenesis	12	1	287	0.0401
GO:0072262	XLOC_023297	Biological Process	Metanephric glomerular mesangial cell proliferation involved in metanephros development	12	1	287	0.0401
GO:0090241	XLOC_024273	Biological Process	Negative regulation of histone H4 acetylation	12	1	287	0.0401
GO:2000591	XLOC_023297	Biological Process	Positive regulation of metanephric mesenchymal cell migration	12	1	287	0.0401
GO:0015936	XLOC_023603	Biological Process	Coenzyme A metabolic process	12	1	287	0.0596
GO:0021836	XLOC_023558	Biological Process	Chemorepulsion involved in postnatal olfactory bulb interneuron migration	12	1	287	0.0596
GO:0021988	XLOC_024239	Biological Process	Olfactory lobe development	12	1	287	0.0596
GO:0060219	XLOC_024296	Biological Process	Camera-type eye photoreceptor cell differentiation	12	1	287	0.0596
GO:1901186	XLOC_023581	Biological Process	Positive regulation of ERBB signaling pathway	12	1	287	0.0596
GO:1901189	XLOC_023581	Biological Process	Positive regulation of ephrin receptor signaling pathway	12	1	287	0.0596
GO:0046069	XLOC_023444	Biological Process	cGMP catabolic process	12	1	287	0.0786
GO:0009912	XLOC_023581	Biological Process	Auditory receptor cell fate commitment	12	1	287	0.0973
GO:0034244	XLOC_024279	Biological Process	Negative regulation of transcription elongation from RNA polymerase II promoter	12	1	287	0.0973
GO:0038091	XLOC_023651	Biological Process	Positive regulation of cell proliferation by VEGF-activated platelet derived growth factor receptor signaling pathway	12	1	287	0.0973
GO:0045743	XLOC_023297	Biological Process	Positive regulation of fibroblast growth factor receptor signaling pathway	12	1	287	0.0973
GO:0046068	XLOC_023444	Biological Process	cGMP metabolic process	12	1	287	0.0973
GO:0051438	XLOC_003625	Biological Process	Regulation of ubiquitin-protein transferase activity	12	1	287	0.0973
GO:0010595	XLOC_023756;XLOC_024081	Biological Process	Positive regulation of endothelial cell migration	12	2	287	0.1032
GO:0070374	XLOC_023297;XLOC_023651;XLOC_024081	Biological Process	Positive regulation of ERK1 and ERK2 cascade	12	3	287	0.1130
GO:0035912	XLOC_023581	Biological Process	Dorsal aorta morphogenesis	12	1	287	0.1156
GO:0042523	XLOC_023653	Biological Process	Positive regulation of tyrosine phosphorylation of Stat5 protein	12	1	287	0.1156
GO:0042511	XLOC_023653	Biological Process	Positive regulation of tyrosine phosphorylation of Stat1 protein	12	1	287	0.1335
GO:0051798	XLOC_013257	Biological Process	Positive regulation of hair follicle development	12	1	287	0.1335

GO:0060042	XLOC_024296	Biological Process	Retina morphogenesis in camera-type eye	12	1	287	0.1335
GO:0033169	XLOC_003350	Biological Process	Histone H3-K9 demethylation	12	1	287	0.1511
GO:0014068	XLOC_023297;XLOC_024081	Biological Process	Positive regulation of phosphatidylinositol 3-kinase signaling	12	2	287	0.1578
GO:0045892	XLOC_011222;XLOC_012264;XLOC_012452;XLOC_021577;XLOC_022857;XLOC_023297;XLOC_023581;XLOC_023853;XLOC_023941; XLOC_024273	Biological Process	Negative regulation of transcription, DNA-templated	12	10	287	0.1639
GO:0006183	XLOC_010523	Biological Process	GTP biosynthetic process	12	1	287	0.1683
GO:0032926	XLOC_013257	Biological Process	Negative regulation of activin receptor signaling pathway	12	1	287	0.1683
GO:2000060	XLOC_013557	Biological Process	Positive regulation of protein ubiquitination involved in ubiquitin-dependent protein catabolic process	12	1	287	0.1683
GO:0003198	XLOC_023581	Biological Process	Epithelial to mesenchymal transition involved in endocardial cushion formation	12	1	287	0.1852
GO:0003214	XLOC_023581	Biological Process	Cardiac left ventricle morphogenesis	12	1	287	0.1852
GO:0006171	XLOC_008893	Biological Process	cAMP biosynthetic process	12	1	287	0.2017
GO:0002042	XLOC_023558	Biological Process	Cell migration involved in sprouting angiogenesis	12	1	287	0.2179
GO:0006334	XLOC_015255;XLOC_015261;XLOC_023518	Biological Process	Nucleosome assembly	12	3	287	0.2207
GO:0010596	XLOC_023558	Biological Process	Negative regulation of endothelial cell migration	12	1	287	0.2337
GO:0021983	XLOC_023581	Biological Process	Pituitary gland development	12	1	287	0.2493
GO:0042517	XLOC_023653	Biological Process	Positive regulation of tyrosine phosphorylation of Stat3 protein	12	1	287	0.2493
GO:0015986	XLOC_024260	Biological Process	ATP synthesis coupled proton transport	12	1	287	0.2794
GO:0032147	XLOC_023297	Biological Process	Activation of protein kinase activity	12	1	287	0.2940
GO:0045766	XLOC_023756;XLOC_024081	Biological Process	Positive regulation of angiogenesis	12	2	287	0.3217
GO:0010718	XLOC_010535	Biological Process	Positive regulation of epithelial to mesenchymal transition	12	1	287	0.3361
GO:0070979	XLOC_023568	Biological Process	Protein K11-linked ubiquitination	12	1	287	0.3884

GO:0000122	XLOC_002956;XLOC_003605;XLOC_009333;XLOC_011222;XLOC_012264;XLOC_013257;XLOC_022857;XLOC_023581;XLOC_024273	Biological Process	Negative regulation of transcription from RNA polymerase II promoter	12	9	287	0.3905
GO:0007204	XLOC_023651	Biological Process	Positive regulation of cytosolic calcium ion concentration	12	1	287	0.4249
GO:0070936	XLOC_023609	Biological Process	Protein K48-linked ubiquitination	12	1	287	0.5504
GO:0031398	XLOC_024155	Biological Process	Positive regulation of protein ubiquitination	12	1	287	0.5685
GO:0006357	XLOC_020267;XLOC_023581;XLOC_023988	Biological Process	Regulation of transcription from RNA polymerase II promoter	12	3	287	0.6114
GO:0001843	XLOC_023606	Biological Process	Neural tube closure	12	1	287	0.6263
GO:0045893	XLOC_003350;XLOC_010535;XLOC_013602;XLOC_023297;XLOC_023787;XLOC_023853;XLOC_023992	Biological Process	Positive regulation of transcription, DNA-templated	12	7	287	0.6879
GO:0006184	XLOC_015530;XLOC_023626;XLOC_024287	Biological Process	GTP catabolic process	12	3	287	0.8771
GO:0031120	XLOC_023428;XLOC_023915	Biological Process	snRNA pseudouridine synthesis	11	2	287	0.0039
GO:0060351	XLOC_010535;XLOC_012279	Biological Process	Cartilage development involved in endochondral bone morphogenesis	11	2	287	0.0039
GO:0055072	XLOC_023501;XLOC_024239;XLOC_024292	Biological Process	Iron ion homeostasis	11	3	287	0.0095
GO:0010613	XLOC_023444;XLOC_023465	Biological Process	Positive regulation of cardiac muscle hypertrophy	11	2	287	0.0106
GO:0060394	XLOC_002956;XLOC_003605	Biological Process	Negative regulation of pathway-restricted SMAD protein phosphorylation	11	2	287	0.0134
GO:0006241	XLOC_010523;XLOC_020698	Biological Process	CTP biosynthetic process	11	2	287	0.0199
GO:0017015	XLOC_002956;XLOC_003605	Biological Process	Regulation of transforming growth factor beta receptor signaling pathway	11	2	287	0.0236
GO:0001947	XLOC_002956;XLOC_003605;XLOC_023978	Biological Process	Heart looping	11	3	287	0.0243
GO:0031953	XLOC_002956;XLOC_003605	Biological Process	Negative regulation of protein autophosphorylation	11	2	287	0.0275
GO:0006742	XLOC_025902	Biological Process	NADP catabolic process	11	1	287	0.0401
GO:0007290	XLOC_003350	Biological Process	Spermatid nucleus elongation	11	1	287	0.0401
GO:0019677	XLOC_025902	Biological Process	NAD catabolic process	11	1	287	0.0401

GO:0021990	XLOC_024239	Biological Process	Neural plate formation	11	1	287	0.0401
GO:0034047	XLOC_024239	Biological Process	Regulation of protein phosphatase type 2A activity	11	1	287	0.0401
GO:0035790	XLOC_023651	Biological Process	Platelet-derived growth factor receptor-alpha signaling pathway	11	1	287	0.0401
GO:0046032	XLOC_023788	Biological Process	ADP catabolic process	11	1	287	0.0401
GO:0048641	XLOC_025523	Biological Process	Regulation of skeletal muscle tissue development	11	1	287	0.0401
GO:0055118	XLOC_023444	Biological Process	Negative regulation of cardiac muscle contraction	11	1	287	0.0401
GO:0072214	XLOC_023978	Biological Process	Metanephric cortex development	11	1	287	0.0401
GO:0072255	XLOC_023297	Biological Process	Metanephric glomerular mesangial cell development	11	1	287	0.0401
GO:1900127	XLOC_023297	Biological Process	Positive regulation of hyaluronan biosynthetic process	11	1	287	0.0401
GO:1901860	XLOC_023992	Biological Process	Positive regulation of mitochondrial DNA metabolic process	11	1	287	0.0401
GO:0014883	XLOC_023512	Biological Process	Transition between fast and slow fiber	11	1	287	0.0596
GO:0060282	XLOC_023444	Biological Process	Positive regulation of oocyte development	11	1	287	0.0596
GO:0060844	XLOC_023581	Biological Process	Arterial endothelial cell fate commitment	11	1	287	0.0596
GO:0072208	XLOC_023978	Biological Process	Metanephric smooth muscle tissue development	11	1	287	0.0596
GO:0072221	XLOC_005548	Biological Process	Metanephric distal convoluted tubule development	11	1	287	0.0596
GO:2000768	XLOC_024296	Biological Process	Positive regulation of nephron tubule epithelial cell differentiation	11	1	287	0.0596
GO:0045740	XLOC_023297;XLOC_023651	Biological Process	Positive regulation of DNA replication	11	2	287	0.0724
GO:0048701	XLOC_023603;XLOC_023651	Biological Process	Embryonic cranial skeleton morphogenesis	11	2	287	0.0783
GO:0007089	XLOC_022857	Biological Process	Traversing start control point of mitotic cell cycle	11	1	287	0.0786
GO:0048742	XLOC_013557	Biological Process	Regulation of skeletal muscle fiber development	11	1	287	0.0786
GO:0048820	XLOC_023581	Biological Process	Hair follicle maturation	11	1	287	0.0786
GO:0072075	XLOC_023978	Biological Process	Metanephric mesenchyme development	11	1	287	0.0786
GO:0043410	XLOC_023297;XLOC_023653;XLOC_024081	Biological Process	Positive regulation of MAPK cascade	11	3	287	0.0882
GO:0048341	XLOC_024239	Biological Process	Paraxial mesoderm formation	11	1	287	0.0973
GO:0070100	XLOC_023558	Biological Process	Negative regulation of chemokine-mediated signaling pathway	11	1	287	0.0973
GO:1900025	XLOC_023908	Biological Process	Negative regulation of substrate adhesion-dependent cell spreading	11	1	287	0.0973
GO:0014911	XLOC_023297	Biological Process	Positive regulation of smooth muscle cell migration	11	1	287	0.1156
GO:0051573	XLOC_003350	Biological Process	Negative regulation of histone H3-K9 methylation	11	1	287	0.1156
GO:0051938	XLOC_024239	Biological Process	L-glutamate import	11	1	287	0.1156

GO:0014912	XLOC_023558	Biological Process	Negative regulation of smooth muscle cell migration	11	1	287	0.1335
GO:0033033	XLOC_023834	Biological Process	Negative regulation of myeloid cell apoptotic process	11	1	287	0.1335
GO:0060315	XLOC_023978	Biological Process	Negative regulation of ryanodine-sensitive calcium-release channel activity	11	1	287	0.1335
GO:0010388	XLOC_023777	Biological Process	Cullin deneddylation	11	1	287	0.1511
GO:0030837	XLOC_023558	Biological Process	Negative regulation of actin filament polymerization	11	1	287	0.1511
GO:0031145	XLOC_023568	Biological Process	Anaphase-promoting complex-dependent proteasomal ubiquitin-dependent protein catabolic process	11	1	287	0.1511
GO:0032434	XLOC_023609	Biological Process	Regulation of proteasomal ubiquitin-dependent protein catabolic process	11	1	287	0.1511
GO:0006228	XLOC_010523	Biological Process	UTP biosynthetic process	11	1	287	0.1683
GO:0035067	XLOC_024273	Biological Process	Negative regulation of histone acetylation	11	1	287	0.1683
GO:0060041	XLOC_003107;XLOC_005548	Biological Process	Retina development in camera-type eye	11	2	287	0.1795
GO:0006283	XLOC_023835	Biological Process	Transcription-coupled nucleotide-excision repair	11	1	287	0.1852
GO:0031954	XLOC_023297	Biological Process	Positive regulation of protein autophosphorylation	11	1	287	0.1852
GO:0051894	XLOC_024081	Biological Process	Positive regulation of focal adhesion assembly	11	1	287	0.1852
GO:0043524	XLOC_023435;XLOC_023992;XLOC_024239	Biological Process	Negative regulation of neuron apoptotic process	11	3	287	0.1893
GO:0031065	XLOC_024273	Biological Process	Positive regulation of histone deacetylation	11	1	287	0.2179
GO:0043518	XLOC_022857	Biological Process	Negative regulation of DNA damage response, signal transduction by p53 class mediator	11	1	287	0.2179
GO:0048741	XLOC_023512	Biological Process	Skeletal muscle fiber development	11	1	287	0.2179
GO:0016180	XLOC_023494	Biological Process	SnRNA processing	11	1	287	0.2337
GO:2000352	XLOC_024081	Biological Process	Negative regulation of endothelial cell apoptotic process	11	1	287	0.2337
GO:0008156	XLOC_024034	Biological Process	Negative regulation of DNA replication	11	1	287	0.2493
GO:0061098	XLOC_023297	Biological Process	Positive regulation of protein tyrosine kinase activity	11	1	287	0.2645
GO:0031146	XLOC_024292	Biological Process	SCF-dependent proteasomal ubiquitin-dependent protein catabolic process	11	1	287	0.2794
GO:0045739	XLOC_024203	Biological Process	Positive regulation of DNA repair	11	1	287	0.3224
GO:0031069	XLOC_013257	Biological Process	Hair follicle morphogenesis	11	1	287	0.3496
GO:0006879	XLOC_001060	Biological Process	Cellular iron ion homeostasis	11	1	287	0.4130
GO:0015991	XLOC_003625	Biological Process	ATP hydrolysis coupled proton transport	11	1	287	0.4366
GO:0006754	XLOC_024260	Biological Process	ATP biosynthetic process	11	1	287	0.4809
GO:0051865	XLOC_024155	Biological Process	Protein autoubiquitination	11	1	287	0.4809
GO:0090263	XLOC_010535	Biological Process	Positive regulation of canonical Wnt signaling pathway	11	1	287	0.5504

GO:0038032	XLOC_024247	Biological Process	Termination of G-protein coupled receptor signaling pathway	11	1	287	0.5858
GO:0006364	XLOC_023928	Biological Process	rRNA processing	11	1	287	0.6893
GO:0000209	XLOC_013557	Biological Process	Protein polyubiquitination	11	1	287	0.7079
GO:0006366	XLOC_023988	Biological Process	Transcription from RNA polymerase II promoter	11	1	287	0.8024
GO:0006200	XLOC_003080	Biological Process	ATP catabolic process	11	1	287	0.8890
GO:0006355	XLOC_002509;XLOC_002956;XLOC_003605;XLOC_003674;XLOC_009917;XLOC_011222;XLOC_013602;XLOC_023581;XLOC_023941;XLOC_023992;XLOC_024038;XLOC_024061	Biological Process	Regulation of transcription, DNA-templated	11	12	287	0.9941
GO:0042787	XLOC_022857;XLOC_023974;XLOC_024075;XLOC_024155	Biological Process	Protein ubiquitination involved in ubiquitin-dependent protein catabolic process	10	4	287	0.0047
GO:0003084	XLOC_002956;XLOC_003605	Biological Process	Positive regulation of systemic arterial blood pressure	10	2	287	0.0080
GO:0048845	XLOC_002956;XLOC_003605	Biological Process	Venous blood vessel morphogenesis	10	2	287	0.0106
GO:0003127	XLOC_023978	Biological Process	Detection of nodal flow	10	1	287	0.0401
GO:0019244	XLOC_024239	Biological Process	Lactate biosynthetic process from pyruvate	10	1	287	0.0401
GO:0019290	XLOC_023501	Biological Process	Siderophore biosynthetic process	10	1	287	0.0401
GO:0031587	XLOC_023978	Biological Process	Positive regulation of inositol 1,4,5-trisphosphate-sensitive calcium-release channel activity	10	1	287	0.0401
GO:0034505	XLOC_010535	Biological Process	Tooth mineralization	10	1	287	0.0401
GO:0035904	XLOC_023978	Biological Process	Aorta development	10	1	287	0.0401
GO:0036123	XLOC_003350	Biological Process	Histone H3-K9 dimethylation	10	1	287	0.0401
GO:0051058	XLOC_023558	Biological Process	Negative regulation of small GTPase mediated signal transduction	10	1	287	0.0401
GO:0072235	XLOC_023978	Biological Process	Metanephric distal tubule development	10	1	287	0.0401
GO:0072286	XLOC_005548	Biological Process	Metanephric connecting tubule development	10	1	287	0.0401
GO:0097324	XLOC_023653	Biological Process	Melanocyte migration	10	1	287	0.0401
GO:0048844	XLOC_002956;XLOC_003605	Biological Process	Artery morphogenesis	10	2	287	0.0455
GO:0001956	XLOC_015335	Biological Process	Positive regulation of neurotransmitter secretion	10	1	287	0.0596
GO:0002327	XLOC_023653	Biological Process	Immature B cell differentiation	10	1	287	0.0596
GO:0006220	XLOC_023703	Biological Process	Pyrimidine nucleotide metabolic process	10	1	287	0.0596
GO:0009107	XLOC_023606	Biological Process	Lipoate biosynthetic process	10	1	287	0.0596
GO:0010512	XLOC_023297	Biological Process	Negative regulation of phosphatidylinositol biosynthetic process	10	1	287	0.0596

GO:0033173	XLOC_023512	Biological Process	Calcineurin-NFAT signaling cascade	10	1	287	0.0596
GO:0038109	XLOC_023653	Biological Process	Kit signaling pathway	10	1	287	0.0596
GO:0042496	XLOC_024021	Biological Process	Detection of diacyl bacterial lipopeptide	10	1	287	0.0596
GO:0072139	XLOC_024296	Biological Process	Glomerular parietal epithelial cell differentiation	10	1	287	0.0596
GO:0072218	XLOC_023978	Biological Process	Metanephric ascending thin limb development	10	1	287	0.0596
GO:0010862	XLOC_002956;XLOC_003605	Biological Process	Positive regulation of pathway-restricted SMAD protein phosphorylation	10	2	287	0.0612
GO:0048010	XLOC_023651;XLOC_024081	Biological Process	Vascular endothelial growth factor receptor signaling pathway	10	2	287	0.0612
GO:0003160	XLOC_023581	Biological Process	Endocardium morphogenesis	10	1	287	0.0786
GO:0006313	XLOC_023775	Biological Process	Transposition, DNA-mediated	10	1	287	0.0786
GO:0006564	XLOC_017018	Biological Process	L-serine biosynthetic process	10	1	287	0.0786
GO:0018095	XLOC_024802	Biological Process	Protein polyglutamylation	10	1	287	0.0786
GO:0072554	XLOC_023581	Biological Process	Blood vessel lumenization	10	1	287	0.0786
GO:0043552	XLOC_023297;XLOC_023651	Biological Process	Positive regulation of phosphatidylinositol 3-kinase activity	10	2	287	0.0843
GO:0048008	XLOC_023297;XLOC_023651	Biological Process	Platelet-derived growth factor receptor signaling pathway	10	2	287	0.0843
GO:0016567	XLOC_003625;XLOC_021996;XLOC_022857;XLOC_023609;XLOC_023835;XLOC_024105;XLOC_024155;XLOC_024292	Biological Process	Protein ubiquitination	10	8	287	0.0912
GO:0001525	XLOC_002956;XLOC_003605;XLOC_018924;XLOC_020726;XLOC_023581	Biological Process	Angiogenesis	10	5	287	0.0969
GO:0001514	XLOC_023999	Biological Process	Selenocysteine incorporation	10	1	287	0.0973
GO:0006221	XLOC_020698	Biological Process	Pyrimidine nucleotide biosynthetic process	10	1	287	0.0973
GO:0007288	XLOC_024802	Biological Process	Sperm axoneme assembly	10	1	287	0.0973
GO:0060763	XLOC_023558	Biological Process	Mammary duct terminal end bud growth	10	1	287	0.0973
GO:0061371	XLOC_023978	Biological Process	Determination of heart left/right asymmetry	10	1	287	0.0973
GO:0071910	XLOC_023978	Biological Process	Determination of liver left/right asymmetry	10	1	287	0.0973
GO:0072112	XLOC_024296	Biological Process	Glomerular visceral epithelial cell differentiation	10	1	287	0.0973
GO:0032228	XLOC_024073	Biological Process	Regulation of synaptic transmission, GABAergic	10	1	287	0.1156
GO:0038084	XLOC_024081	Biological Process	Vascular endothelial growth factor signaling pathway	10	1	287	0.1156
GO:0048592	XLOC_003749	Biological Process	Eye morphogenesis	10	1	287	0.1156
GO:0051151	XLOC_024148	Biological Process	Negative regulation of smooth muscle cell differentiation	10	1	287	0.1156

GO:0060664	XLOC_023297	Biological Process	Epithelial cell proliferation involved in salivary gland morphogenesis	10	1	287	0.1156
GO:0070933	XLOC_023963	Biological Process	Histone H4 deacetylation	10	1	287	0.1156
GO:0001656	XLOC_012264;XLOC_023558	Biological Process	Metanephros development	10	2	287	0.1298
GO:0001960	XLOC_020267	Biological Process	Negative regulation of cytokine-mediated signaling pathway	10	1	287	0.1335
GO:0010830	XLOC_013557	Biological Process	Regulation of myotube differentiation	10	1	287	0.1335
GO:0015807	XLOC_001311	Biological Process	L-amino acid transport	10	1	287	0.1335
GO:0019800	XLOC_023435	Biological Process	Peptide cross-linking via chondroitin 4-sulfate glycosaminoglycan	10	1	287	0.1335
GO:0070932	XLOC_023963	Biological Process	Histone H3 deacetylation	10	1	287	0.1335
GO:2000573	XLOC_023297	Biological Process	Positive regulation of DNA biosynthetic process	10	1	287	0.1335
GO:0006546	XLOC_003697	Biological Process	Glycine catabolic process	10	1	287	0.1511
GO:0031116	XLOC_023632	Biological Process	Positive regulation of microtubule polymerization	10	1	287	0.1511
GO:0035584	XLOC_024081	Biological Process	Calcium-mediated signaling using intracellular calcium source	10	1	287	0.1511
GO:0006165	XLOC_010523	Biological Process	Nucleoside diphosphate phosphorylation	10	1	287	0.1683
GO:0080182	XLOC_023933	Biological Process	Histone H3-K4 trimethylation	10	1	287	0.1683
GO:0046427	XLOC_023653	Biological Process	Positive regulation of JAK-STAT cascade	10	1	287	0.1852
GO:0070208	XLOC_010535	Biological Process	Protein heterotrimerization	10	1	287	0.1852
GO:0042475	XLOC_013257;XLOC_023651	Biological Process	Odontogenesis of dentin-containing tooth	10	2	287	0.1941
GO:0050910	XLOC_023653	Biological Process	Detection of mechanical stimulus involved in sensory perception of sound	10	1	287	0.2017
GO:2000134	XLOC_023978	Biological Process	Negative regulation of G1/S transition of mitotic cell cycle	10	1	287	0.2017
GO:0019933	XLOC_024265	Biological Process	cAMP-mediated signaling	10	1	287	0.2337
GO:0046855	XLOC_003678	Biological Process	Inositol phosphate dephosphorylation	10	1	287	0.2337
GO:0001934	XLOC_002956;XLOC_003605;XLOC_024081	Biological Process	Positive regulation of protein phosphorylation	10	3	287	0.2476
GO:0032008	XLOC_023520	Biological Process	Positive regulation of TOR signaling	10	1	287	0.2493
GO:0045653	XLOC_015261	Biological Process	Negative regulation of megakaryocyte differentiation	10	1	287	0.2493
GO:0007179	XLOC_002956;XLOC_003605	Biological Process	Transforming growth factor beta receptor signaling pathway	10	2	287	0.2540
GO:0046856	XLOC_003678	Biological Process	Phosphatidylinositol dephosphorylation	10	1	287	0.2794
GO:2001237	XLOC_024239	Biological Process	Negative regulation of extrinsic apoptotic signaling pathway	10	1	287	0.2794
GO:0007093	XLOC_023439	Biological Process	Mitotic cell cycle checkpoint	10	1	287	0.2940
GO:0030073	XLOC_024239	Biological Process	Insulin secretion	10	1	287	0.2940
GO:0009190	XLOC_008893	Biological Process	Cyclic nucleotide biosynthetic process	10	1	287	0.3224
GO:0030318	XLOC_023653	Biological Process	Melanocyte differentiation	10	1	287	0.3224

GO:0031648	XLOC_022857	Biological Process	Protein destabilization	10	1	287	0.3224
GO:0055074	XLOC_024081	Biological Process	Calcium ion homeostasis	10	1	287	0.3224
GO:0001837	XLOC_023581	Biological Process	Epithelial to mesenchymal transition	10	1	287	0.3884
GO:0048666	XLOC_024239	Biological Process	Neuron development	10	1	287	0.3884
GO:0006635	XLOC_023501	Biological Process	Fatty acid beta-oxidation	10	1	287	0.4130
GO:0031572	XLOC_024203	Biological Process	G2 DNA damage checkpoint	10	1	287	0.4130
GO:0016579	XLOC_024073	Biological Process	Protein deubiquitination	10	1	287	0.4366
GO:0035019	XLOC_023581	Biological Process	Somatic stem cell maintenance	10	1	287	0.4480
GO:0001933	XLOC_023558	Biological Process	Negative regulation of protein phosphorylation	10	1	287	0.4809
GO:0003151	XLOC_023581	Biological Process	Outflow tract morphogenesis	10	1	287	0.4915
GO:0048839	XLOC_024099	Biological Process	Inner ear development	10	1	287	0.4915
GO:0010923	XLOC_003747	Biological Process	Negative regulation of phosphatase activity	10	1	287	0.5595
GO:0046854	XLOC_003678	Biological Process	Phosphatidylinositol phosphorylation	10	1	287	0.5685
GO:0048704	XLOC_012279	Biological Process	Embryonic skeletal system morphogenesis	10	1	287	0.5685
GO:0006302	XLOC_024203	Biological Process	Double-strand break repair	10	1	287	0.5943
GO:0006874	XLOC_023978	Biological Process	Cellular calcium ion homeostasis	10	1	287	0.6486
GO:0050731	XLOC_023297	Biological Process	Positive regulation of peptidyl-tyrosine phosphorylation	10	1	287	0.6696
GO:0043161	XLOC_024254	Biological Process	Proteasome-mediated ubiquitin-dependent protein catabolic process	10	1	287	0.6957
GO:0043123	XLOC_024021	Biological Process	Positive regulation of I-kappaB kinase/NF-kappaB signaling	10	1	287	0.8935
GO:0006351	XLOC_002509;XLOC_012264;XLOC_023581;XLOC_023992	Biological Process	Transcription, DNA-templated	10	4	287	0.9954
GO:0003273	XLOC_002956;XLOC_003605	Biological Process	Cell migration involved in endocardial cushion formation	9	2	287	0.0080
GO:0018108	XLOC_023297;XLOC_023651;XLOC_023653;XLOC_024081	Biological Process	Peptidyl-tyrosine phosphorylation	9	4	287	0.0236
GO:0048490	XLOC_015335;XLOC_023861	Biological Process	Anterograde synaptic vesicle transport	9	2	287	0.0361
GO:0006069	XLOC_023529	Biological Process	Ethanol oxidation	9	1	287	0.0401
GO:0010593	XLOC_023558	Biological Process	Negative regulation of lamellipodium assembly	9	1	287	0.0401
GO:0021972	XLOC_023558	Biological Process	Corticospinal neuron axon guidance through spinal cord	9	1	287	0.0401
GO:0045083	XLOC_023941	Biological Process	Negative regulation of interleukin-12 biosynthetic process	9	1	287	0.0401
GO:0072219	XLOC_023978	Biological Process	Metanephric cortical collecting duct development	9	1	287	0.0401
GO:0090288	XLOC_023558	Biological Process	Negative regulation of cellular response to growth factor stimulus	9	1	287	0.0401
GO:1901857	XLOC_023992	Biological Process	Positive regulation of cellular respiration	9	1	287	0.0401
GO:2000739	XLOC_023651	Biological Process	Regulation of mesenchymal stem cell differentiation	9	1	287	0.0401

GO:0071158	XLOC_009333;XLOC_023978	Biological Process	Positive regulation of cell cycle arrest	9	2	287	0.0407
GO:0008089	XLOC_015335;XLOC_023861	Biological Process	Anterograde axon cargo transport	9	2	287	0.0455
GO:0006829	XLOC_004385;XLOC_023510	Biological Process	Zinc ion transport	9	2	287	0.0558
GO:0001523	XLOC_023529	Biological Process	Retinoid metabolic process	9	1	287	0.0596
GO:0018205	XLOC_022857	Biological Process	Peptidyl-lysine modification	9	1	287	0.0596
GO:0031113	XLOC_023632	Biological Process	Regulation of microtubule polymerization	9	1	287	0.0596
GO:0038162	XLOC_023653	Biological Process	Erythropoietin-mediated signaling pathway	9	1	287	0.0596
GO:0046439	XLOC_001806	Biological Process	L-cysteine metabolic process	9	1	287	0.0596
GO:0051414	XLOC_023558	Biological Process	Response to cortisol	9	1	287	0.0596
GO:0060374	XLOC_023653	Biological Process	Mast cell differentiation	9	1	287	0.0596
GO:0060830	XLOC_023603	Biological Process	Ciliary receptor clustering involved in smoothened signaling pathway	9	1	287	0.0596
GO:0071726	XLOC_024021	Biological Process	Cellular response to diacyl bacterial lipopeptide	9	1	287	0.0596
GO:0090024	XLOC_023558	Biological Process	Negative regulation of neutrophil chemotaxis	9	1	287	0.0596
GO:0090169	XLOC_024254	Biological Process	Regulation of spindle assembly	9	1	287	0.0596
GO:2000785	XLOC_023863	Biological Process	Regulation of autophagic vacuole assembly	9	1	287	0.0596
GO:0018119	XLOC_023529	Biological Process	Peptidyl-cysteine S-nitrosylation	9	1	287	0.0786
GO:0022029	XLOC_023558	Biological Process	Telencephalon cell migration	9	1	287	0.0786
GO:0042471	XLOC_023603	Biological Process	Ear morphogenesis	9	1	287	0.0786
GO:0045777	XLOC_023529	Biological Process	Positive regulation of blood pressure	9	1	287	0.0786
GO:0090280	XLOC_023297	Biological Process	Positive regulation of calcium ion import	9	1	287	0.0786
GO:0006506	XLOC_023552;XLOC_023825	Biological Process	GPI anchor biosynthetic process	9	2	287	0.0968
GO:0010631	XLOC_002973	Biological Process	Epithelial cell migration	9	1	287	0.0973
GO:0018206	XLOC_021864	Biological Process	Peptidyl-methionine modification	9	1	287	0.0973
GO:0030072	XLOC_024239	Biological Process	Peptide hormone secretion	9	1	287	0.0973
GO:0043312	XLOC_023756	Biological Process	Neutrophil degranulation	9	1	287	0.0973
GO:0047496	XLOC_024239	Biological Process	Vesicle transport along microtubule	9	1	287	0.0973
GO:0060831	XLOC_023603	Biological Process	Smoothened signaling pathway involved in dorsal/ventral neural tube patterning	9	1	287	0.0973
GO:0007169	XLOC_023651;XLOC_023653;XLOC_024081	Biological Process	Transmembrane receptor protein tyrosine kinase signaling pathway	9	3	287	0.1044
GO:0003157	XLOC_023581	Biological Process	Endocardium development	9	1	287	0.1156
GO:0010863	XLOC_023651	Biological Process	Positive regulation of phospholipase C activity	9	1	287	0.1156
GO:0018401	XLOC_018228	Biological Process	Peptidyl-proline hydroxylation to 4-hydroxy-L-proline	9	1	287	0.1156
GO:0030213	XLOC_023297	Biological Process	Hyaluronan biosynthetic process	9	1	287	0.1156

GO:0036302	XLOC_023581	Biological Process	Atrioventricular canal development	9	1	287	0.1156
GO:0043303	XLOC_023653	Biological Process	Mast cell degranulation	9	1	287	0.1156
GO:0055003	XLOC_023651	Biological Process	Cardiac myofibril assembly	9	1	287	0.1156
GO:0030336	XLOC_018924;XLOC_023297;XLOC_023558	Biological Process	Negative regulation of cell migration	9	3	287	0.1218
GO:0048706	XLOC_010535;XLOC_024298	Biological Process	Embryonic skeletal system development	9	2	287	0.1230
GO:0006563	XLOC_017018	Biological Process	L-serine metabolic process	9	1	287	0.1335
GO:0008535	XLOC_024147	Biological Process	Respiratory chain complex IV assembly	9	1	287	0.1335
GO:0032006	XLOC_023520	Biological Process	Regulation of TOR signaling	9	1	287	0.1335
GO:0033280	XLOC_012858	Biological Process	Response to vitamin D	9	1	287	0.1335
GO:0043589	XLOC_010535	Biological Process	Skin morphogenesis	9	1	287	0.1335
GO:0048733	XLOC_023581	Biological Process	Sebaceous gland development	9	1	287	0.1335
GO:0060603	XLOC_023558	Biological Process	Mammary gland duct morphogenesis	9	1	287	0.1335
GO:0070493	XLOC_024265	Biological Process	Thrombin receptor signaling pathway	9	1	287	0.1335
GO:0030335	XLOC_010535;XLOC_023297;XLOC_023651;XLOC_024081	Biological Process	Positive regulation of cell migration	9	4	287	0.1341
GO:0001955	XLOC_024148	Biological Process	Blood vessel maturation	9	1	287	0.1511
GO:0021510	XLOC_023978	Biological Process	Spinal cord development	9	1	287	0.1511
GO:0051291	XLOC_023745	Biological Process	Protein heterooligomerization	9	1	287	0.1511
GO:0001921	XLOC_021577	Biological Process	Positive regulation of receptor recycling	9	1	287	0.1683
GO:0006614	XLOC_023666	Biological Process	SRP-dependent cotranslational protein targeting to membrane	9	1	287	0.1683
GO:0015074	XLOC_023775	Biological Process	DNA integration	9	1	287	0.1683
GO:0035313	XLOC_003749	Biological Process	Wound healing, spreading of epidermal cells	9	1	287	0.1683
GO:0035855	XLOC_023653	Biological Process	Megakaryocyte development	9	1	287	0.1683
GO:0051770	XLOC_024081	Biological Process	Positive regulation of nitric-oxide synthase biosynthetic process	9	1	287	0.1683
GO:0007212	XLOC_024239	Biological Process	Dopamine receptor signaling pathway	9	1	287	0.1852
GO:0060346	XLOC_010535	Biological Process	Bone trabecula formation	9	1	287	0.1852
GO:0007218	XLOC_023990;XLOC_028154	Biological Process	Neuropeptide signaling pathway	9	2	287	0.2015
GO:0006541	XLOC_020698	Biological Process	Glutamine metabolic process	9	1	287	0.2017
GO:0071157	XLOC_022857	Biological Process	Negative regulation of cell cycle arrest	9	1	287	0.2017
GO:0048738	XLOC_012279	Biological Process	Cardiac muscle tissue development	9	1	287	0.2179
GO:0050730	XLOC_023297	Biological Process	Regulation of peptidyl-tyrosine phosphorylation	9	1	287	0.2179
GO:0006631	XLOC_011326;XLOC_023920	Biological Process	Fatty acid metabolic process	9	2	287	0.2239
GO:0006865	XLOC_001311	Biological Process	Amino acid transport	9	1	287	0.2337
GO:0043116	XLOC_023558	Biological Process	Negative regulation of vascular permeability	9	1	287	0.2337
GO:0048557	XLOC_023651	Biological Process	Embryonic digestive tract morphogenesis	9	1	287	0.2337

GO:0006816	XLOC_021577;XLOC_023512;XLOC_023978	Biological Process	Calcium ion transport	9	3	287	0.2368
GO:0001779	XLOC_003107	Biological Process	Natural killer cell differentiation	9	1	287	0.2493
GO:0006783	XLOC_020095	Biological Process	Heme biosynthetic process	9	1	287	0.2493
GO:0006826	XLOC_001060	Biological Process	Iron ion transport	9	1	287	0.2493
GO:0009165	XLOC_023703	Biological Process	Nucleotide biosynthetic process	9	1	287	0.2493
GO:0046777	XLOC_023651;XLOC_023653;XLOC_023702;XLOC_024081	Biological Process	Protein autophosphorylation	9	4	287	0.2523
GO:0006511	XLOC_023888;XLOC_024073;XLOC_024075;XLOC_024105	Biological Process	Ubiquitin-dependent protein catabolic process	9	4	287	0.2612
GO:0000132	XLOC_024239	Biological Process	Establishment of mitotic spindle orientation	9	1	287	0.2645
GO:0006493	XLOC_023933	Biological Process	Protein O-linked glycosylation	9	1	287	0.2645
GO:0042177	XLOC_023439	Biological Process	Negative regulation of protein catabolic process	9	1	287	0.2645
GO:0045840	XLOC_023297	Biological Process	Positive regulation of mitosis	9	1	287	0.2645
GO:0008277	XLOC_021577	Biological Process	Regulation of G-protein coupled receptor protein signaling pathway	9	1	287	0.2794
GO:0060079	XLOC_023512	Biological Process	Regulation of excitatory postsynaptic membrane potential	9	1	287	0.2794
GO:0051056	XLOC_016622;XLOC_024185	Biological Process	Regulation of small GTPase mediated signal transduction	9	2	287	0.2842
GO:0042476	XLOC_012264	Biological Process	Odontogenesis	9	1	287	0.3083
GO:0045429	XLOC_023978	Biological Process	Positive regulation of nitric oxide biosynthetic process	9	1	287	0.3083
GO:0048167	XLOC_024239	Biological Process	Regulation of synaptic plasticity	9	1	287	0.3083
GO:0007205	XLOC_024265	Biological Process	Protein kinase C-activating G-protein coupled receptor signaling pathway	9	1	287	0.3361
GO:0042130	XLOC_023444	Biological Process	Negative regulation of T cell proliferation	9	1	287	0.3361
GO:0042327	XLOC_023702	Biological Process	Positive regulation of phosphorylation	9	1	287	0.3496
GO:0046488	XLOC_003678	Biological Process	Phosphatidylinositol metabolic process	9	1	287	0.3496
GO:0019722	XLOC_023512	Biological Process	Calcium-mediated signaling	9	1	287	0.3628
GO:0021915	XLOC_023978	Biological Process	Neural tube development	9	1	287	0.3628
GO:0003007	XLOC_003749	Biological Process	Heart morphogenesis	9	1	287	0.3757
GO:0030521	XLOC_003350	Biological Process	Androgen receptor signaling pathway	9	1	287	0.3757
GO:0045600	XLOC_020726	Biological Process	Positive regulation of fat cell differentiation	9	1	287	0.3757
GO:0051402	XLOC_024239	Biological Process	Neuron apoptotic process	9	1	287	0.3757
GO:0001822	XLOC_023933;XLOC_023978	Biological Process	Kidney development	9	2	287	0.3806

GO:0001701	XLOC_023558;XLOC_023603;XLOC_023651;XLOC_024251;XLOC_024298	Biological Process	In utero embryonic development	9	5	287	0.3815
GO:0048015	XLOC_023651	Biological Process	Phosphatidylinositol-mediated signaling	9	1	287	0.3884
GO:0032720	XLOC_020726	Biological Process	Negative regulation of tumor necrosis factor production	9	1	287	0.4008
GO:0034446	XLOC_003107	Biological Process	Substrate adhesion-dependent cell spreading	9	1	287	0.4249
GO:0030879	XLOC_015126	Biological Process	Mammary gland development	9	1	287	0.4366
GO:0030217	XLOC_023653	Biological Process	T cell differentiation	9	1	287	0.4480
GO:0030890	XLOC_023853	Biological Process	Positive regulation of B cell proliferation	9	1	287	0.4480
GO:0045668	XLOC_012264	Biological Process	Negative regulation of osteoblast differentiation	9	1	287	0.4480
GO:0048286	XLOC_024081	Biological Process	Lung alveolus development	9	1	287	0.4480
GO:0030183	XLOC_023581	Biological Process	B cell differentiation	9	1	287	0.4592
GO:0008360	XLOC_023653;XLOC_024081	Biological Process	Regulation of cell shape	9	2	287	0.4645
GO:0030216	XLOC_023581	Biological Process	Keratinocyte differentiation	9	1	287	0.4702
GO:0007368	XLOC_023978	Biological Process	Determination of left/right symmetry	9	1	287	0.4809
GO:0006006	XLOC_023596	Biological Process	Glucose metabolic process	9	1	287	0.5119
GO:0007286	XLOC_023653	Biological Process	Spermatid development	9	1	287	0.5119
GO:0007420	XLOC_012279;XLOC_024239	Biological Process	Brain development	9	2	287	0.5166
GO:0006352	XLOC_015261	Biological Process	DNA-templated transcription, initiation	9	1	287	0.5504
GO:0006633	XLOC_024190	Biological Process	Fatty acid biosynthetic process	9	1	287	0.5772
GO:0007601	XLOC_010535	Biological Process	Visual perception	9	1	287	0.5772
GO:0030326	XLOC_023603	Biological Process	Embryonic limb morphogenesis	9	1	287	0.5943
GO:0006396	XLOC_023903	Biological Process	RNA processing	9	1	287	0.6106
GO:0030182	XLOC_023581	Biological Process	Neuron differentiation	9	1	287	0.6106
GO:0051260	XLOC_023848;XLOC_024075	Biological Process	Protein homooligomerization	9	2	287	0.6614
GO:0007605	XLOC_010535	Biological Process	Sensory perception of sound	9	1	287	0.6763
GO:0018105	XLOC_023297	Biological Process	Peptidyl-serine phosphorylation	9	1	287	0.7019
GO:0006310	XLOC_023775	Biological Process	DNA recombination	9	1	287	0.7254
GO:0043066	XLOC_022857;XLOC_023439;XLOC_023853;XLOC_024081;XLOC_024194	Biological Process	Negative regulation of apoptotic process	9	5	287	0.7256
GO:0043065	XLOC_023558;XLOC_024038	Biological Process	Positive regulation of apoptotic process	9	2	287	0.8141
GO:0035335	XLOC_023786	Biological Process	Peptidyl-tyrosine dephosphorylation	9	1	287	0.8457
GO:0006260	XLOC_024024	Biological Process	DNA replication	9	1	287	0.8608
GO:0006281	XLOC_023835;XLOC_024203	Biological Process	DNA repair	9	2	287	0.8772

GO:0060326	XLOC_002956;XLOC_003605;XLOC_023297;XLOC_023651;XLOC_023653	Biological Process	Cell chemotaxis	8	5	287	0.0002
GO:0035502	XLOC_005548;XLOC_023978	Biological Process	Metanephric part of ureteric bud development	8	2	287	0.0039
GO:0022009	XLOC_002956;XLOC_003605	Biological Process	Central nervous system vasculogenesis	8	2	287	0.0058
GO:0010544	XLOC_023297;XLOC_023651	Biological Process	Negative regulation of platelet activation	8	2	287	0.0080
GO:0051001	XLOC_002956;XLOC_003605	Biological Process	Negative regulation of nitric-oxide synthase activity	8	2	287	0.0106
GO:0006622	XLOC_003322;XLOC_024165	Biological Process	Protein targeting to lysosome	8	2	287	0.0165
GO:0006744	XLOC_002962;XLOC_024196	Biological Process	Ubiquinone biosynthetic process	8	2	287	0.0199
GO:0007417	XLOC_012264;XLOC_020670;XLOC_024239	Biological Process	Central nervous system development	8	3	287	0.0310
GO:0048705	XLOC_010535;XLOC_023651;XLOC_023830	Biological Process	Skeletal system morphogenesis	8	3	287	0.0310
GO:0008585	XLOC_013257;XLOC_023651	Biological Process	Female gonad development	8	2	287	0.0317
GO:0038093	XLOC_023653	Biological Process	Fc receptor signaling pathway	8	1	287	0.0401
GO:0061055	XLOC_023603	Biological Process	Myotome development	8	1	287	0.0401
GO:0070861	XLOC_003322	Biological Process	Regulation of protein exit from endoplasmic reticulum	8	1	287	0.0401
GO:0071955	XLOC_003322	Biological Process	Recycling endosome to Golgi transport	8	1	287	0.0401
GO:0097326	XLOC_023653	Biological Process	Melanocyte adhesion	8	1	287	0.0401
GO:1901863	XLOC_023992	Biological Process	Positive regulation of muscle tissue development	8	1	287	0.0401
GO:0000050	XLOC_024239	Biological Process	Urea cycle	8	1	287	0.0596
GO:0019805	XLOC_024239	Biological Process	Quinolinate biosynthetic process	8	1	287	0.0596
GO:0033563	XLOC_023558	Biological Process	Dorsal/ventral axon guidance	8	1	287	0.0596
GO:0034499	XLOC_003322	Biological Process	Late endosome to Golgi transport	8	1	287	0.0596
GO:0045446	XLOC_024081	Biological Process	Endothelial cell differentiation	8	1	287	0.0596
GO:0051147	XLOC_025523	Biological Process	Regulation of muscle cell differentiation	8	1	287	0.0596
GO:0090234	XLOC_024254	Biological Process	Regulation of kinetochore assembly	8	1	287	0.0596
GO:0097101	XLOC_023581	Biological Process	Blood vessel endothelial cell fate specification	8	1	287	0.0596
GO:2000507	XLOC_023992	Biological Process	Positive regulation of energy homeostasis	8	1	287	0.0596
GO:2001178	XLOC_009333	Biological Process	Positive regulation of mediator complex assembly	8	1	287	0.0596
GO:0034504	XLOC_010535;XLOC_022857	Biological Process	Protein localization to nucleus	8	2	287	0.0667

GO:0001541	XLOC_023653;XLOC_024081	Biological Process	Ovarian follicle development	8	2	287	0.0724
GO:0090305	XLOC_001362;XLOC_016324;XLOC_020726	Biological Process	Nucleic acid phosphodiester bond hydrolysis	8	3	287	0.0768
GO:0000052	XLOC_024239	Biological Process	Citrulline metabolic process	8	1	287	0.0786
GO:0006929	XLOC_023297	Biological Process	Substrate-dependent cell migration	8	1	287	0.0786
GO:0010884	XLOC_020726	Biological Process	Positive regulation of lipid storage	8	1	287	0.0786
GO:0014816	XLOC_025523	Biological Process	Skeletal muscle satellite cell differentiation	8	1	287	0.0786
GO:0031365	XLOC_021864	Biological Process	N-terminal protein amino acid modification	8	1	287	0.0786
GO:0048822	XLOC_023834	Biological Process	Enucleate erythrocyte development	8	1	287	0.0786
GO:0048846	XLOC_023558	Biological Process	Axon extension involved in axon guidance	8	1	287	0.0786
GO:0055119	XLOC_023444	Biological Process	Relaxation of cardiac muscle	8	1	287	0.0786
GO:0061333	XLOC_023978	Biological Process	Renal tubule morphogenesis	8	1	287	0.0786
GO:0071504	XLOC_023558	Biological Process	Cellular response to heparin	8	1	287	0.0786
GO:0072177	XLOC_023978	Biological Process	Mesonephric duct development	8	1	287	0.0786
GO:0007507	XLOC_002956;XLOC_003605;XLOC_012264;XLOC_023297;XLOC_023581;XLOC_023978	Biological Process	Heart development	8	6	287	0.0868
GO:0001568	XLOC_003749;XLOC_010535;XLOC_023297	Biological Process	Blood vessel development	8	3	287	0.0962
GO:0045596	XLOC_013257;XLOC_023581	Biological Process	Negative regulation of cell differentiation	8	2	287	0.0968
GO:0048505	XLOC_023581	Biological Process	Regulation of timing of cell differentiation	8	1	287	0.0973
GO:0055001	XLOC_025523	Biological Process	Muscle cell development	8	1	287	0.0973
GO:0071361	XLOC_008893	Biological Process	Cellular response to ethanol	8	1	287	0.0973
GO:0072205	XLOC_005548	Biological Process	Metanephric collecting duct development	8	1	287	0.0973
GO:0001569	XLOC_002956;XLOC_003605	Biological Process	Patterning of blood vessels	8	2	287	0.1032
GO:0010628	XLOC_015335;XLOC_020726;XLOC_023581;XLOC_023653;XLOC_023978	Biological Process	Positive regulation of gene expression	8	5	287	0.1130
GO:0002573	XLOC_023653	Biological Process	Myeloid leukocyte differentiation	8	1	287	0.1156
GO:0006686	XLOC_023922	Biological Process	Sphingomyelin biosynthetic process	8	1	287	0.1156
GO:0006729	XLOC_024302	Biological Process	Tetrahydrobiopterin biosynthetic process	8	1	287	0.1156
GO:0014059	XLOC_015335	Biological Process	Regulation of dopamine secretion	8	1	287	0.1156
GO:0032364	XLOC_018228	Biological Process	Oxygen homeostasis	8	1	287	0.1156
GO:0032543	XLOC_024098	Biological Process	Mitochondrial translation	8	1	287	0.1156
GO:0034067	XLOC_003322	Biological Process	Protein localization to Golgi apparatus	8	1	287	0.1156

GO:0042711	XLOC_013602	Biological Process	Maternal behavior	8	1	287	0.1156
GO:0043069	XLOC_023653	Biological Process	Negative regulation of programmed cell death	8	1	287	0.1156
GO:0043457	XLOC_024098	Biological Process	Regulation of cellular respiration	8	1	287	0.1156
GO:0046825	XLOC_024239	Biological Process	Regulation of protein export from nucleus	8	1	287	0.1156
GO:0070528	XLOC_024265	Biological Process	Protein kinase C signaling	8	1	287	0.1156
GO:0072602	XLOC_023581	Biological Process	Interleukin-4 secretion	8	1	287	0.1156
GO:0090279	XLOC_023978	Biological Process	Regulation of calcium ion import	8	1	287	0.1156
GO:0002318	XLOC_023653	Biological Process	Myeloid progenitor cell differentiation	8	1	287	0.1335
GO:0010606	XLOC_024173	Biological Process	Positive regulation of cytoplasmic mRNA processing body assembly	8	1	287	0.1335
GO:0030903	XLOC_012264	Biological Process	Notochord development	8	1	287	0.1335
GO:0032020	XLOC_023974	Biological Process	ISG15-protein conjugation	8	1	287	0.1335
GO:0045019	XLOC_020726	Biological Process	Negative regulation of nitric oxide biosynthetic process	8	1	287	0.1335
GO:0002320	XLOC_023653	Biological Process	Lymphoid progenitor cell differentiation	8	1	287	0.1511
GO:0006044	XLOC_024053	Biological Process	N-acetylglucosamine metabolic process	8	1	287	0.1511
GO:0008088	XLOC_024239	Biological Process	Axon cargo transport	8	1	287	0.1511
GO:0023019	XLOC_023651	Biological Process	Signal transduction involved in regulation of gene expression	8	1	287	0.1511
GO:0042407	XLOC_024278	Biological Process	Cristae formation	8	1	287	0.1511
GO:0042994	XLOC_023978	Biological Process	Cytoplasmic sequestering of transcription factor	8	1	287	0.1511
GO:0043129	XLOC_024081	Biological Process	Surfactant homeostasis	8	1	287	0.1511
GO:0050872	XLOC_024273	Biological Process	White fat cell differentiation	8	1	287	0.1511
GO:0061298	XLOC_023651	Biological Process	Retina vasculature development in camera-type eye	8	1	287	0.1511
GO:0001553	XLOC_023651	Biological Process	Luteinization	8	1	287	0.1683
GO:0031076	XLOC_023603	Biological Process	Embryonic camera-type eye development	8	1	287	0.1683
GO:0001945	XLOC_024081	Biological Process	Lymph vessel development	8	1	287	0.2017
GO:0010507	XLOC_023863	Biological Process	Negative regulation of autophagy	8	1	287	0.2017
GO:0016331	XLOC_024141	Biological Process	Morphogenesis of embryonic epithelium	8	1	287	0.2017
GO:0032922	XLOC_011222	Biological Process	Circadian regulation of gene expression	8	1	287	0.2017
GO:0050804	XLOC_023512	Biological Process	Regulation of synaptic transmission	8	1	287	0.2017
GO:0097067	XLOC_023653	Biological Process	Cellular response to thyroid hormone stimulus	8	1	287	0.2017
GO:0043588	XLOC_003749;XLOC_010535	Biological Process	Skin development	8	2	287	0.2090

GO:0006468	XLOC_002973;XLOC_003107;XLOC_017920;XLOC_023297;XLOC_023465;XLOC_023491;XLOC_023651;XLOC_023653;XLOC_023758;XLOC_023907;XLOC_024081;XLOC_024183;XLOC_024247;XLOC_024264;XLOC_025531	Biological Process	Protein phosphorylation	8	15	287	0.2106
GO:0022904	XLOC_023992	Biological Process	Respiratory electron transport chain	8	1	287	0.2179
GO:0060068	XLOC_003107	Biological Process	Vagina development	8	1	287	0.2179
GO:0072001	XLOC_023978	Biological Process	Renal system development	8	1	287	0.2179
GO:0007259	XLOC_023978	Biological Process	JAK-STAT cascade	8	1	287	0.2337
GO:0031290	XLOC_023558	Biological Process	Retinal ganglion cell axon guidance	8	1	287	0.2337
GO:0042176	XLOC_022857	Biological Process	Regulation of protein catabolic process	8	1	287	0.2337
GO:0007219	XLOC_015126;XLOC_023581	Biological Process	Notch signaling pathway	8	2	287	0.2389
GO:0006779	XLOC_020095	Biological Process	Porphyrin-containing compound biosynthetic process	8	1	287	0.2493
GO:0030325	XLOC_023651	Biological Process	Adrenal gland development	8	1	287	0.2493
GO:0008045	XLOC_023558	Biological Process	Motor neuron axon guidance	8	1	287	0.2645
GO:0032715	XLOC_020726	Biological Process	Negative regulation of interleukin-6 production	8	1	287	0.2645
GO:0007249	XLOC_023472	Biological Process	I-kappaB kinase/NF-kappaB signaling	8	1	287	0.2794
GO:0010508	XLOC_020726	Biological Process	Positive regulation of autophagy	8	1	287	0.2940
GO:0007369	XLOC_024239	Biological Process	Gastrulation	8	1	287	0.3224
GO:0009267	XLOC_013557	Biological Process	Cellular response to starvation	8	1	287	0.3224
GO:0035329	XLOC_023702	Biological Process	Hippo signaling	8	1	287	0.3224
GO:0035924	XLOC_024081	Biological Process	Cellular response to vascular endothelial growth factor stimulus	8	1	287	0.3224
GO:0050766	XLOC_003107	Biological Process	Positive regulation of phagocytosis	8	1	287	0.3224
GO:0010629	XLOC_020726;XLOC_023558	Biological Process	Negative regulation of gene expression	8	2	287	0.3291
GO:0001764	XLOC_023435;XLOC_024038	Biological Process	Neuron migration	8	2	287	0.3440
GO:0043491	XLOC_003107	Biological Process	Protein kinase B signaling	8	1	287	0.3496
GO:0035108	XLOC_023830	Biological Process	Limb morphogenesis	8	1	287	0.3628
GO:0006259	XLOC_016324	Biological Process	DNA metabolic process	8	1	287	0.3884
GO:0006813	XLOC_024806;XLOC_026899	Biological Process	Potassium ion transport	8	2	287	0.3950
GO:0050873	XLOC_024194	Biological Process	Brown fat cell differentiation	8	1	287	0.4008
GO:0008542	XLOC_024239	Biological Process	Visual learning	8	1	287	0.4130
GO:0045931	XLOC_022857	Biological Process	Positive regulation of mitotic cell cycle	8	1	287	0.4130

GO:0030324	XLOC_023651;XLOC_024081	Biological Process	Lung development	8	2	287	0.4233
GO:0050853	XLOC_023853	Biological Process	B cell receptor signaling pathway	8	1	287	0.4366
GO:0010976	XLOC_023435	Biological Process	Positive regulation of neuron projection development	8	1	287	0.4480
GO:0001658	XLOC_023978	Biological Process	Branching involved in ureteric bud morphogenesis	8	1	287	0.4592
GO:0009952	XLOC_024141;XLOC_024239	Biological Process	Anterior/posterior pattern specification	8	2	287	0.4645
GO:0006470	XLOC_023512;XLOC_023553;XLOC_023786	Biological Process	Protein dephosphorylation	8	3	287	0.4975
GO:0048469	XLOC_024081	Biological Process	Cell maturation	8	1	287	0.5018
GO:0000082	XLOC_023512	Biological Process	G1/S transition of mitotic cell cycle	8	1	287	0.5218
GO:0006417	XLOC_023531	Biological Process	Regulation of translation	8	1	287	0.5218
GO:0015992	XLOC_024260	Biological Process	Proton transport	8	1	287	0.5316
GO:0050885	XLOC_023861	Biological Process	Neuromuscular process controlling balance	8	1	287	0.5316
GO:0007224	XLOC_023603	Biological Process	Smoothened signaling pathway	8	1	287	0.5411
GO:0051216	XLOC_023762	Biological Process	Cartilage development	8	1	287	0.5504
GO:0006913	XLOC_013557;XLOC_024287	Biological Process	Nucleocytoplasmic transport	8	2	287	0.5652
GO:0050728	XLOC_023941	Biological Process	Negative regulation of inflammatory response	8	1	287	0.5685
GO:0071222	XLOC_020726	Biological Process	Cellular response to lipopolysaccharide	8	1	287	0.5685
GO:0001889	XLOC_023978	Biological Process	Liver development	8	1	287	0.6025
GO:0008584	XLOC_023653	Biological Process	Male gonad development	8	1	287	0.6106
GO:0006413	XLOC_023531	Biological Process	Translational initiation	8	1	287	0.6263
GO:0006814	XLOC_003157	Biological Process	Sodium ion transport	8	1	287	0.6486
GO:0007409	XLOC_023558	Biological Process	Axonogenesis	8	1	287	0.6829
GO:0019221	XLOC_023653	Biological Process	Cytokine-mediated signaling pathway	8	1	287	0.7079
GO:0009887	XLOC_023651	Biological Process	Organ morphogenesis	8	1	287	0.7310
GO:0016055	XLOC_025531	Biological Process	Wnt signaling pathway	8	1	287	0.7983
GO:0007264	XLOC_023626;XLOC_024185;XLOC_024287	Biological Process	Small GTPase mediated signal transduction	8	3	287	0.8644
GO:0007186	XLOC_021577;XLOC_023990;XLOC_024265;XLOC_028154	Biological Process	G-protein coupled receptor signaling pathway	8	4	287	0.8908
GO:0042981	XLOC_024150	Biological Process	Regulation of apoptotic process	8	1	287	0.9235
GO:0070527	XLOC_023651;XLOC_023861	Biological Process	Platelet aggregation	7	2	287	0.0134
GO:0001937	XLOC_002956;XLOC_003605;XLOC_023923	Biological Process	Negative regulation of endothelial cell proliferation	7	3	287	0.0152
GO:0006888	XLOC_023448;XLOC_023917;XLOC_024239	Biological Process	ER to Golgi vesicle-mediated transport	7	3	287	0.0185

GO:0051592	XLOC_023512;XLOC_024191;XLOC_024239	Biological Process	Response to calcium ion	7	3	287	0.0185
GO:0071230	XLOC_010535;XLOC_023520;XLOC_023651	Biological Process	Cellular response to amino acid stimulus	7	3	287	0.0243
GO:2000379	XLOC_020726;XLOC_023297	Biological Process	Positive regulation of reactive oxygen species metabolic process	7	2	287	0.0275
GO:0048745	XLOC_002956;XLOC_003605	Biological Process	Smooth muscle tissue development	7	2	287	0.0361
GO:0002689	XLOC_023558	Biological Process	Negative regulation of leukocyte chemotaxis	7	1	287	0.0401
GO:0032762	XLOC_023653	Biological Process	Mast cell cytokine production	7	1	287	0.0401
GO:0046293	XLOC_003350	Biological Process	Formaldehyde biosynthetic process	7	1	287	0.0401
GO:0048280	XLOC_023730	Biological Process	Vesicle fusion with Golgi apparatus	7	1	287	0.0401
GO:0050929	XLOC_023558	Biological Process	Induction of negative chemotaxis	7	1	287	0.0401
GO:0051204	XLOC_024147	Biological Process	Protein insertion into mitochondrial membrane	7	1	287	0.0401
GO:0051304	XLOC_023963	Biological Process	Chromosome separation	7	1	287	0.0401
GO:0071676	XLOC_023558	Biological Process	Negative regulation of mononuclear cell migration	7	1	287	0.0401
GO:0072126	XLOC_023297	Biological Process	Positive regulation of glomerular mesangial cell proliferation	7	1	287	0.0401
GO:0090260	XLOC_023558	Biological Process	Negative regulation of retinal ganglion cell axon guidance	7	1	287	0.0401
GO:2000404	XLOC_020267	Biological Process	Regulation of T cell migration	7	1	287	0.0401
GO:0016477	XLOC_002956;XLOC_003605;XLOC_003749;XLOC_023651;XLOC_024081	Biological Process	Cell migration	7	5	287	0.0405
GO:0001957	XLOC_010535	Biological Process	Intramembranous ossification	7	1	287	0.0596
GO:0009957	XLOC_023581	Biological Process	Epidermal cell fate specification	7	1	287	0.0596
GO:0010822	XLOC_023992	Biological Process	Positive regulation of mitochondrion organization	7	1	287	0.0596
GO:0035563	XLOC_009333	Biological Process	Positive regulation of chromatin binding	7	1	287	0.0596
GO:0038001	XLOC_023297	Biological Process	Paracrine signaling	7	1	287	0.0596
GO:0045112	XLOC_003749	Biological Process	Integrin biosynthetic process	7	1	287	0.0596
GO:0046294	XLOC_023529	Biological Process	Formaldehyde catabolic process	7	1	287	0.0596
GO:0071731	XLOC_018228	Biological Process	Response to nitric oxide	7	1	287	0.0596
GO:0001958	XLOC_010535;XLOC_012279	Biological Process	Endochondral ossification	7	2	287	0.0667

GO:0016310	XLOC_002973;XLOC_003107;XLOC_010523;XLOC_023465;XLOC_023651;XLOC_023653;XLOC_023703;XLOC_023758;XLOC_024081;XLOC_024183;XLOC_024247;XLOC_025531	Biological Process	Phosphorylation	7	12	287	0.0743
GO:0030001	XLOC_004385;XLOC_023510	Biological Process	Metal ion transport	7	2	287	0.0783
GO:0001818	XLOC_023941	Biological Process	Negative regulation of cytokine production	7	1	287	0.0786
GO:0050819	XLOC_023432	Biological Process	Negative regulation of coagulation	7	1	287	0.0786
GO:0051205	XLOC_024147	Biological Process	Protein insertion into membrane	7	1	287	0.0786
GO:0051250	XLOC_003107	Biological Process	Negative regulation of lymphocyte activation	7	1	287	0.0786
GO:0060155	XLOC_015335	Biological Process	Platelet dense granule organization	7	1	287	0.0786
GO:0001570	XLOC_002956;XLOC_003605;XLOC_024081	Biological Process	Vasculogenesis	7	3	287	0.0805
GO:0042742	XLOC_023581;XLOC_023756;XLOC_024194	Biological Process	Defense response to bacterium	7	3	287	0.0805
GO:0031623	XLOC_021577;XLOC_024247	Biological Process	Rceptor internalization	7	2	287	0.0968
GO:0048812	XLOC_015335;XLOC_023558	Biological Process	Neuron projection morphogenesis	7	2	287	0.0968
GO:0015695	XLOC_001311	Biological Process	Organic cation transport	7	1	287	0.0973
GO:0035989	XLOC_003749	Biological Process	Tendon development	7	1	287	0.0973
GO:0043654	XLOC_025523	Biological Process	Recognition of apoptotic cell	7	1	287	0.0973
GO:0072164	XLOC_023978	Biological Process	Mesonephric tubule development	7	1	287	0.0973
GO:0048754	XLOC_023558;XLOC_024081	Biological Process	Branching morphogenesis of an epithelial tube	7	2	287	0.1097
GO:0031023	XLOC_003322	Biological Process	Microtubule organizing center organization	7	1	287	0.1156
GO:0043206	XLOC_003749	Biological Process	Extracellular fibril organization	7	1	287	0.1156
GO:0046168	XLOC_023920	Biological Process	Glycerol-3-phosphate catabolic process	7	1	287	0.1156
GO:0051382	XLOC_024104	Biological Process	Kinetochore assembly	7	1	287	0.1156
GO:0055123	XLOC_023603	Biological Process	Digestive system development	7	1	287	0.1156
GO:0030199	XLOC_003749;XLOC_010535	Biological Process	Collagen fibril organization	7	2	287	0.1163
GO:0060325	XLOC_010535;XLOC_023651	Biological Process	Face morphogenesis	7	2	287	0.1230

GO:0000902	XLOC_023603;XLOC_023830;XLOC_024251	Biological Process	Cell morphogenesis	7	3	287	0.1308
GO:0006687	XLOC_023653	Biological Process	Glycosphingolipid metabolic process	7	1	287	0.1335
GO:0007020	XLOC_023632	Biological Process	Microtubule nucleation	7	1	287	0.1335
GO:0033327	XLOC_023651	Biological Process	Leydig cell differentiation	7	1	287	0.1335
GO:0035051	XLOC_024072	Biological Process	Cardiocyte differentiation	7	1	287	0.1335
GO:0045165	XLOC_023581;XLOC_024081	Biological Process	Cell fate commitment	7	2	287	0.1367
GO:0001938	XLOC_023297;XLOC_024081	Biological Process	Positive regulation of endothelial cell proliferation	7	2	287	0.1436
GO:0031532	XLOC_015335;XLOC_023653	Biological Process	Actin cytoskeleton reorganization	7	2	287	0.1436
GO:0000042	XLOC_003322	Biological Process	Protein targeting to Golgi	7	1	287	0.1511
GO:0006516	XLOC_023509	Biological Process	Glycoprotein catabolic process	7	1	287	0.1511
GO:0009755	XLOC_003350	Biological Process	Hormone-mediated signaling pathway	7	1	287	0.1683
GO:0015711	XLOC_003157	Biological Process	Organic anion transport	7	1	287	0.1683
GO:0034109	XLOC_025523	Biological Process	Homotypic cell-cell adhesion	7	1	287	0.1683
GO:0031122	XLOC_023632	Biological Process	Cytoplasmic microtubule organization	7	1	287	0.1852
GO:0034453	XLOC_003322	Biological Process	Microtubule anchoring	7	1	287	0.1852
GO:0060445	XLOC_023297	Biological Process	Branching involved in salivary gland morphogenesis	7	1	287	0.1852
GO:0060674	XLOC_023978	Biological Process	Placenta blood vessel development	7	1	287	0.1852
GO:0006464	XLOC_023974;XLOC_023975;XLOC_024105	Biological Process	Cellular protein modification process	7	3	287	0.1997
GO:0006071	XLOC_024196	Biological Process	Glycerol metabolic process	7	1	287	0.2017
GO:0008340	XLOC_024239	Biological Process	Determination of adult lifespan	7	1	287	0.2017
GO:0030500	XLOC_023758	Biological Process	Regulation of bone mineralization	7	1	287	0.2017
GO:0030539	XLOC_023651	Biological Process	Male genitalia development	7	1	287	0.2017
GO:0032438	XLOC_023861	Biological Process	Melanosome organization	7	1	287	0.2017
GO:0032870	XLOC_023558	Biological Process	Cellular response to hormone stimulus	7	1	287	0.2017
GO:0048821	XLOC_023834	Biological Process	Erythrocyte development	7	1	287	0.2017
GO:0033198	XLOC_024265	Biological Process	Response to ATP	7	1	287	0.2179
GO:0040015	XLOC_024194	Biological Process	Negative regulation of multicellular organism growth	7	1	287	0.2179
GO:0031032	XLOC_023618	Biological Process	Actomyosin structure organization	7	1	287	0.2337
GO:0048565	XLOC_023653	Biological Process	Digestive tract development	7	1	287	0.2337
GO:0071805	XLOC_023978;XLOC_026899	Biological Process	Potassium ion transmembrane transport	7	2	287	0.2389
GO:0006890	XLOC_024239	Biological Process	Retrograde vesicle-mediated transport, Golgi to ER	7	1	287	0.2493
GO:0030099	XLOC_023933	Biological Process	Myeloid cell differentiation	7	1	287	0.2493
GO:0046902	XLOC_024239	Biological Process	Regulation of mitochondrial membrane permeability	7	1	287	0.2493
GO:0002244	XLOC_013257	Biological Process	Hematopoietic progenitor cell differentiation	7	1	287	0.2645

GO:0007283	XLOC_003107;XLOC_003350;XLOC_023653;XLOC_024239	Biological Process	Spermatogenesis	7	4	287	0.2747
GO:0006904	XLOC_024074	Biological Process	Vesicle docking involved in exocytosis	7	1	287	0.2794
GO:0019430	XLOC_023564	Biological Process	Removal of superoxide radicals	7	1	287	0.2794
GO:0035162	XLOC_023653	Biological Process	Embryonic hemopoiesis	7	1	287	0.2794
GO:0030279	XLOC_023581	Biological Process	Negative regulation of ossification	7	1	287	0.2940
GO:0003333	XLOC_001311	Biological Process	Amino acid transmembrane transport	7	1	287	0.3083
GO:0006644	XLOC_023480	Biological Process	Phospholipid metabolic process	7	1	287	0.3083
GO:0043388	XLOC_010523	Biological Process	Positive regulation of DNA binding	7	1	287	0.3083
GO:0060716	XLOC_023581	Biological Process	Labyrinthine layer blood vessel development	7	1	287	0.3083
GO:0008306	XLOC_024239	Biological Process	Associative learning	7	1	287	0.3224
GO:0008652	XLOC_017018	Biological Process	Cellular amino acid biosynthetic process	7	1	287	0.3224
GO:0048863	XLOC_023653	Biological Process	Stem cell differentiation	7	1	287	0.3224
GO:0048873	XLOC_024251	Biological Process	Homeostasis of number of cells within a tissue	7	1	287	0.3224
GO:0006406	XLOC_003001	Biological Process	mRNA export from nucleus	7	1	287	0.3361
GO:0035725	XLOC_023978	Biological Process	Sodium ion transmembrane transport	7	1	287	0.3496
GO:0002062	XLOC_012279	Biological Process	Chondrocyte differentiation	7	1	287	0.3628
GO:0071407	XLOC_012858	Biological Process	Cellular response to organic cyclic compound	7	1	287	0.3628
GO:0016568	XLOC_023963;XLOC_024203	Biological Process	Chromatin modification	7	2	287	0.3660
GO:0071363	XLOC_023297	Biological Process	Cellular response to growth factor stimulus	7	1	287	0.3757
GO:0006412	XLOC_002615;XLOC_003316;XLOC_003706;XLOC_023531;XLOC_023626;XLOC_028013	Biological Process	Translation	7	6	287	0.3812
GO:0007050	XLOC_023978;XLOC_024038	Biological Process	Cell cycle arrest	7	2	287	0.3878
GO:0030855	XLOC_012264	Biological Process	Epithelial cell differentiation	7	1	287	0.4008
GO:0051781	XLOC_023297	Biological Process	Positive regulation of cell division	7	1	287	0.4008
GO:0032496	XLOC_023529;XLOC_023606	Biological Process	Response to lipopolysaccharide	7	2	287	0.4092
GO:0002053	XLOC_024081	Biological Process	Positive regulation of mesenchymal cell proliferation	7	1	287	0.4130
GO:0022008	XLOC_024239	Biological Process	Neurogenesis	7	1	287	0.4130
GO:0030032	XLOC_023653	Biological Process	Lamellipodium assembly	7	1	287	0.4130
GO:0042147	XLOC_003322	Biological Process	Retrograde transport, endosome to Golgi	7	1	287	0.4130
GO:0007411	XLOC_023558;XLOC_024038	Biological Process	Axon guidance	7	2	287	0.4163
GO:0030163	XLOC_022857	Biological Process	Protein catabolic process	7	1	287	0.4249

GO:0016311	XLOC_012858;XLOC_017018;XLOC_023512	Biological Process	Dephosphorylation	7	3	287	0.4507
GO:0001657	XLOC_023558	Biological Process	Ureteric bud development	7	1	287	0.4809
GO:0007268	XLOC_024802	Biological Process	Synaptic transmission	7	1	287	0.4915
GO:0016485	XLOC_021864	Biological Process	Protein processing	7	1	287	0.4915
GO:0006886	XLOC_021577;XLOC_023448;XLOC_023730;XLOC_023801;XLOC_023917;XLOC_024287	Biological Process	Intracellular protein transport	7	6	287	0.5097
GO:0006461	XLOC_022857	Biological Process	Protein complex assembly	7	1	287	0.5119
GO:0060271	XLOC_023603	Biological Process	Cilium morphogenesis	7	1	287	0.5218
GO:0030308	XLOC_023558;XLOC_024038	Biological Process	Negative regulation of cell growth	7	2	287	0.5228
GO:0006915	XLOC_020726;XLOC_022472;XLOC_023481;XLOC_023813;XLOC_024239	Biological Process	Apoptotic process	7	5	287	0.5380
GO:0009411	XLOC_023835	Biological Process	Response to UV	7	1	287	0.5411
GO:0007166	XLOC_023990;XLOC_028154	Biological Process	Cell surface receptor signaling pathway	7	2	287	0.5593
GO:0001649	XLOC_010535	Biological Process	Osteoblast differentiation	7	1	287	0.5595
GO:0070588	XLOC_023978	Biological Process	Calcium ion transmembrane transport	7	1	287	0.6263
GO:0035556	XLOC_008893;XLOC_016622;XLOC_021996;XLOC_024265	Biological Process	Intracellular signal transduction	7	4	287	0.6654
GO:0001501	XLOC_010535	Biological Process	Skeletal system development	7	1	287	0.7418
GO:0010468	XLOC_023581	Biological Process	Regulation of gene expression	7	1	287	0.8142
GO:0006457	XLOC_023519	Biological Process	Protein folding	7	1	287	0.9116
GO:0001300	XLOC_002956;XLOC_003605	Biological Process	Chronological cell aging	6	2	287	0.0058
GO:0010941	XLOC_023992;XLOC_024098	Biological Process	Regulation of cell death	6	2	287	0.0058
GO:0070483	XLOC_002956;XLOC_003605	Biological Process	Detection of hypoxia	6	2	287	0.0058
GO:0022617	XLOC_002956;XLOC_003605	Biological Process	Extracellular matrix disassembly	6	2	287	0.0106
GO:0032964	XLOC_003749;XLOC_010535	Biological Process	Collagen biosynthetic process	6	2	287	0.0106
GO:0006954	XLOC_020267;XLOC_023606;XLOC_023653;XLOC_023720;XLOC_024021;XLOC_024161	Biological Process	Inflammatory response	6	6	287	0.0216

GO:0006606	XLOC_003001;XLOC_023512;XLOC_024239	Biological Process	Protein import into nucleus	6	3	287	0.0287
GO:0042060	XLOC_002956;XLOC_003605;XLOC_023651	Biological Process	Wound healing	6	3	287	0.0359
GO:0002371	XLOC_023653	Biological Process	Dendritic cell cytokine production	6	1	287	0.0401
GO:0002551	XLOC_023653	Biological Process	Mast cell chemotaxis	6	1	287	0.0401
GO:0002828	XLOC_020267	Biological Process	Regulation of type 2 immune response	6	1	287	0.0401
GO:0006710	XLOC_024219	Biological Process	Androgen catabolic process	6	1	287	0.0401
GO:0014719	XLOC_025523	Biological Process	Skeletal muscle satellite cell activation	6	1	287	0.0401
GO:0018958	XLOC_024127	Biological Process	Phenol-containing compound metabolic process	6	1	287	0.0401
GO:0042745	XLOC_002509	Biological Process	Circadian sleep/wake cycle	6	1	287	0.0401
GO:0051923	XLOC_024127	Biological Process	Sulfation	6	1	287	0.0401
GO:0071464	XLOC_023978	Biological Process	Cellular response to hydrostatic pressure	6	1	287	0.0401
GO:0030097	XLOC_023581;XLOC_023653;XLOC_023933;XLOC_024081	Biological Process	Hemopoiesis	6	4	287	0.0447
GO:0006139	XLOC_003268;XLOC_023703;XLOC_023892	Biological Process	Nucleobase-containing compound metabolic process	6	3	287	0.0499
GO:0048870	XLOC_002956;XLOC_003605	Biological Process	Cell motility	6	2	287	0.0506
GO:0030198	XLOC_012279;XLOC_023435;XLOC_023651;XLOC_024038	Biological Process	Extracellular matrix organization	6	4	287	0.0558
GO:0016192	XLOC_001463;XLOC_003322;XLOC_023730;XLOC_023801;XLOC_023923;XLOC_023970;XLOC_024074	Biological Process	Vesicle-mediated transport	6	7	287	0.0595
GO:0051648	XLOC_024802	Biological Process	Vesicle localization	6	1	287	0.0596
GO:0061364	XLOC_023558	Biological Process	Apoptotic process involved in luteolysis	6	1	287	0.0596
GO:0071672	XLOC_023558	Biological Process	Negative regulation of smooth muscle cell chemotaxis	6	1	287	0.0596
GO:0046677	XLOC_012858;XLOC_022857	Biological Process	Response to antibiotic	6	2	287	0.0612
GO:0016052	XLOC_023596	Biological Process	Carbohydrate catabolic process	6	1	287	0.0786
GO:0045475	XLOC_002509	Biological Process	Locomotor rhythm	6	1	287	0.0786
GO:0048878	XLOC_023830	Biological Process	Chemical homeostasis	6	1	287	0.0786
GO:0050982	XLOC_023978	Biological Process	Detection of mechanical stimulus	6	1	287	0.0786
GO:0071470	XLOC_023978	Biological Process	Cellular response to osmotic stress	6	1	287	0.0786
GO:0090161	XLOC_003322	Biological Process	Golgi ribbon formation	6	1	287	0.0786
GO:1901998	XLOC_001311	Biological Process	Toxin transport	6	1	287	0.0786

GO:2000138	XLOC_023581	Biological Process	Positive regulation of cell proliferation involved in heart morphogenesis	6	1	287	0.0786
GO:0020027	XLOC_024251	Biological Process	Hemoglobin metabolic process	6	1	287	0.0973
GO:0035234	XLOC_023653	Biological Process	Ectopic germ cell programmed cell death	6	1	287	0.0973
GO:0050927	XLOC_024081	Biological Process	Positive regulation of positive chemotaxis	6	1	287	0.0973
GO:0070997	XLOC_023992	Biological Process	Neuron death	6	1	287	0.0973
GO:0071498	XLOC_023978	Biological Process	Cellular response to fluid shear stress	6	1	287	0.0973
GO:0030218	XLOC_023653;XLOC_024251	Biological Process	Erythrocyte differentiation	6	2	287	0.1298
GO:0001778	XLOC_003206	Biological Process	Plasma membrane repair	6	1	287	0.1335
GO:0006796	XLOC_023495	Biological Process	Phosphate-containing compound metabolic process	6	1	287	0.1335
GO:0006884	XLOC_024251	Biological Process	Cell volume homeostasis	6	1	287	0.1335
GO:0008210	XLOC_023651	Biological Process	Estrogen metabolic process	6	1	287	0.1335
GO:0048070	XLOC_023653	Biological Process	Regulation of developmental pigmentation	6	1	287	0.1335
GO:0050688	XLOC_023974	Biological Process	Regulation of defense response to virus	6	1	287	0.1335
GO:0001558	XLOC_002593;XLOC_024099	Biological Process	Regulation of cell growth	6	2	287	0.1367
GO:0002548	XLOC_023297	Biological Process	Monocyte chemotaxis	6	1	287	0.1511
GO:0003016	XLOC_023529	Biological Process	Respiratory system process	6	1	287	0.1511
GO:0006906	XLOC_003206	Biological Process	Vesicle fusion	6	1	287	0.1511
GO:0010812	XLOC_010535	Biological Process	Negative regulation of cell-substrate adhesion	6	1	287	0.1511
GO:0006839	XLOC_024239	Biological Process	Mitochondrial transport	6	1	287	0.1683
GO:0009416	XLOC_011222	Biological Process	Response to light stimulus	6	1	287	0.1683
GO:0009791	XLOC_020670;XLOC_023933;XLOC_024298	Biological Process	Post-embryonic development	6	3	287	0.1691
GO:0071456	XLOC_022857;XLOC_023297	Biological Process	Cellular response to hypoxia	6	2	287	0.1722
GO:0048146	XLOC_023297;XLOC_023651	Biological Process	Positive regulation of fibroblast proliferation	6	2	287	0.1795
GO:0030031	XLOC_023297	Biological Process	Cell projection assembly	6	1	287	0.1852
GO:0043616	XLOC_013257	Biological Process	Keratinocyte proliferation	6	1	287	0.1852
GO:0048193	XLOC_023730	Biological Process	Golgi vesicle transport	6	1	287	0.1852
GO:0050679	XLOC_010523;XLOC_024081	Biological Process	Positive regulation of epithelial cell proliferation	6	2	287	0.1941
GO:0051726	XLOC_003001;XLOC_023747;XLOC_024273	Biological Process	Regulation of cell cycle	6	3	287	0.1945
GO:0045184	XLOC_022857	Biological Process	Establishment of protein localization	6	1	287	0.2017
GO:0050877	XLOC_023603	Biological Process	Neurological system process	6	1	287	0.2017
GO:0042254	XLOC_023428;XLOC_023915	Biological Process	Ribosome biogenesis	6	2	287	0.2239
GO:0008202	XLOC_024127	Biological Process	Steroid metabolic process	6	1	287	0.2337
GO:0043277	XLOC_003107	Biological Process	Apoptotic cell clearance	6	1	287	0.2337
GO:0050919	XLOC_023558	Biological Process	Negative chemotaxis	6	1	287	0.2337
GO:0006801	XLOC_023564	Biological Process	Superoxide metabolic process	6	1	287	0.2493

GO:0060045	XLOC_023581	Biological Process	Positive regulation of cardiac muscle cell proliferation	6	1	287	0.2493
GO:0000278	XLOC_024104	Biological Process	Mitotic cell cycle	6	1	287	0.2645
GO:0035690	XLOC_023512	Biological Process	Cellular response to drug	6	1	287	0.3083
GO:0006611	XLOC_003001	Biological Process	Protein export from nucleus	6	1	287	0.3224
GO:0048661	XLOC_023297	Biological Process	Positive regulation of smooth muscle cell proliferation	6	1	287	0.3224
GO:0051881	XLOC_024239	Biological Process	Regulation of mitochondrial membrane potential	6	1	287	0.3224
GO:0030168	XLOC_003107	Biological Process	Platelet activation	6	1	287	0.3496
GO:0007165	XLOC_002509;XLOC_002772;XLOC_002973;XLOC_003749;XLOC_010535;XLOC_011222;XLOC_012452;XLOC_013602;XLOC_016622;XLOC_023444;XLOC_023941;XLOC_023990;XLOC_024021;XLOC_024150;XLOC_024220;XLOC_024247;XLOC_024265;XLOC_024287;XLOC_024802	Biological Process	Signal transduction	6	19	287	0.3507
GO:0045787	XLOC_022857	Biological Process	Positive regulation of cell cycle	6	1	287	0.3628
GO:0016197	XLOC_024239	Biological Process	Endosomal transport	6	1	287	0.3757
GO:0034599	XLOC_023992	Biological Process	Cellular response to oxidative stress	6	1	287	0.3757
GO:0050918	XLOC_023297	Biological Process	Positive chemotaxis	6	1	287	0.3757
GO:0071310	XLOC_005548	Biological Process	Cellular response to organic substance	6	1	287	0.3757
GO:0048147	XLOC_009333	Biological Process	Negative regulation of fibroblast proliferation	6	1	287	0.3884
GO:0001974	XLOC_023581	Biological Process	Blood vessel remodeling	6	1	287	0.4008
GO:0072659	XLOC_021577	Biological Process	Protein localization to plasma membrane	6	1	287	0.4008
GO:0007612	XLOC_024239	Biological Process	Learning	6	1	287	0.4130
GO:0007040	XLOC_023830	Biological Process	Lysosome organization	6	1	287	0.4249
GO:0010811	XLOC_023435	Biological Process	Positive regulation of cell-substrate adhesion	6	1	287	0.4249
GO:0007267	XLOC_023923	Biological Process	Cell-cell signaling	6	1	287	0.4480
GO:0006887	XLOC_001463	Biological Process	Exocytosis	6	1	287	0.4702
GO:0010212	XLOC_024203	Biological Process	Response to ionizing radiation	6	1	287	0.4915
GO:0015031	XLOC_002497;XLOC_010535;XLOC_021577;XLOC_023448;XLOC_023730;XLOC_023917;XLOC_024147;XLOC_024287	Biological Process	Protein transport	6	8	287	0.5007
GO:0034613	XLOC_023520	Biological Process	Cellular protein localization	6	1	287	0.5018
GO:0007389	XLOC_013257	Biological Process	Pattern specification process	6	1	287	0.5119
GO:0016042	XLOC_023480	Biological Process	Lipid catabolic process	6	1	287	0.5119

GO:0007596	XLOC_015335	Biological Process	Blood coagulation	6	1	287	0.5218
GO:0071260	XLOC_010535	Biological Process	Cellular response to mechanical stimulus	6	1	287	0.5772
GO:0030154	XLOC_015126;XLOC_020726;XLOC_023762	Biological Process	Cell differentiation	6	3	287	0.5800
GO:0008610	XLOC_024190	Biological Process	Lipid biosynthetic process	6	1	287	0.6339
GO:0042384	XLOC_023603	Biological Process	Cilium assembly	6	1	287	0.6829
GO:0031175	XLOC_015335	Biological Process	Neuron projection development	6	1	287	0.6893
GO:0016337	XLOC_023558	Biological Process	Single organismal cell-cell adhesion	6	1	287	0.7365
GO:0009790	XLOC_024239	Biological Process	Embryo development	6	1	287	0.7671
GO:0022900	XLOC_025657	Biological Process	Electron transport chain	6	1	287	0.7898
GO:0006812	XLOC_023621	Biological Process	Cation transport	6	1	287	0.7941
GO:0030036	XLOC_023297	Biological Process	Actin cytoskeleton organization	6	1	287	0.8392
GO:0006508	XLOC_021864;XLOC_023481;XLOC_023555;XLOC_023953;XLOC_024073	Biological Process	Proteolysis	6	5	287	0.8889
GO:0006974	XLOC_023835;XLOC_024203	Biological Process	Cellular response to DNA damage stimulus	6	2	287	0.9116
GO:0009812	XLOC_024127	Biological Process	Flavonoid metabolic process	5	1	287	0.0401
GO:0014841	XLOC_025523	Biological Process	Skeletal muscle satellite cell proliferation	5	1	287	0.0401
GO:0060341	XLOC_023465	Biological Process	Regulation of cellular localization	5	1	287	0.0401
GO:0001666	XLOC_002956;XLOC_003605;XLOC_018228;XLOC_023564	Biological Process	Response to hypoxia	5	4	287	0.0558
GO:0051409	XLOC_023529	Biological Process	Response to nitrosative stress	5	1	287	0.0596
GO:0043086	XLOC_001060;XLOC_002593	Biological Process	Negative regulation of catalytic activity	5	2	287	0.0724
GO:0035176	XLOC_013602;XLOC_024239	Biological Process	Social behavior	5	2	287	0.0783
GO:0051128	XLOC_003749	Biological Process	Regulation of cellular component organization	5	1	287	0.0786
GO:0001892	XLOC_023297;XLOC_023978	Biological Process	Embryonic placenta development	5	2	287	0.0843
GO:0006909	XLOC_003107;XLOC_023756	Biological Process	Phagocytosis	5	2	287	0.0843
GO:0014850	XLOC_023992	Biological Process	Response to muscle activity	5	1	287	0.0973
GO:0042403	XLOC_024127	Biological Process	Thyroid hormone metabolic process	5	1	287	0.0973
GO:0050920	XLOC_023651	Biological Process	Regulation of chemotaxis	5	1	287	0.0973

GO:0055114	XLOC_001806;XLOC_002857;XLOC_003697;XLOC_011982;XLOC_020095;XLOC_023529;XLOC_023564;XLOC_023603;XLOC_023920;XLOC_023921;XLOC_023999;XLOC_024190;XLOC_024219;XLOC_024273;XLOC_024302	Biological Process	Oxidation-reduction process	5	15	287	0.1093
GO:0050921	XLOC_023297	Biological Process	Positive regulation of chemotaxis	5	1	287	0.1156
GO:0055085	XLOC_001311;XLOC_003464;XLOC_004385;XLOC_007550;XLOC_023510;XLOC_023621;XLOC_023813;XLOC_023978;XLOC_024261;XLOC_024806;XLOC_026899	Biological Process	Transmembrane transport	5	11	287	0.1290
GO:0032940	XLOC_003107	Biological Process	Secretion by cell	5	1	287	0.1511
GO:0002437	XLOC_023581	Biological Process	Inflammatory response to antigenic stimulus	5	1	287	0.1683
GO:0006805	XLOC_024127	Biological Process	Xenobiotic metabolic process	5	1	287	0.1683
GO:0001662	XLOC_024073	Biological Process	Behavioral fear response	5	1	287	0.1852
GO:0002504	XLOC_015580	Biological Process	Antigen processing and presentation of peptide or polysaccharide antigen via MHC class II	5	1	287	0.2017
GO:0030534	XLOC_024802	Biological Process	Adult behavior	5	1	287	0.2017
GO:0007029	XLOC_024239	Biological Process	Endoplasmic reticulum organization	5	1	287	0.2337
GO:0019538	XLOC_023555	Biological Process	Protein metabolic process	5	1	287	0.2337
GO:0007276	XLOC_013257	Biological Process	Gamete generation	5	1	287	0.2493
GO:0009314	XLOC_023653	Biological Process	Response to radiation	5	1	287	0.2493
GO:0048066	XLOC_023653	Biological Process	Developmental pigmentation	5	1	287	0.2493
GO:0009409	XLOC_024194	Biological Process	Response to cold	5	1	287	0.2645
GO:0010033	XLOC_023432	Biological Process	Response to organic substance	5	1	287	0.2794
GO:0045494	XLOC_024296	Biological Process	Photoreceptor cell maintenance	5	1	287	0.2940
GO:0006959	XLOC_023581	Biological Process	Humoral immune response	5	1	287	0.3083
GO:0005975	XLOC_023509;XLOC_023596;XLOC_023830;XLOC_024053	Biological Process	Carbohydrate metabolic process	5	4	287	0.3156
GO:0008284	XLOC_023297;XLOC_023581;XLOC_023651;XLOC_023653;XLOC_024081;XLOC_024194	Biological Process	Positive regulation of cell proliferation	5	6	287	0.3271

GO:0007611	XLOC_024239	Biological Process	Learning or memory	5	1	287	0.3361
GO:0009611	XLOC_023297	Biological Process	Response to wounding	5	1	287	0.3361
GO:0072593	XLOC_023297	Biological Process	Reactive oxygen species metabolic process	5	1	287	0.3496
GO:0007569	XLOC_024239	Biological Process	Cell aging	5	1	287	0.3628
GO:0042493	XLOC_023666;XLOC_023853	Biological Process	Response to drug	5	2	287	0.4441
GO:0006811	XLOC_003157;XLOC_004385;XLOC_023978;XLOC_024260;XLOC_024806;XLOC_026899	Biological Process	Ion transport	5	6	287	0.4605
GO:0006629	XLOC_011326;XLOC_024127;XLOC_024190	Biological Process	Lipid metabolic process	5	3	287	0.4665
GO:0016049	XLOC_023435	Biological Process	Cell growth	5	1	287	0.4702
GO:0007010	XLOC_023834;XLOC_024072	Biological Process	Cytoskeleton organization	5	2	287	0.4974
GO:0007005	XLOC_024239	Biological Process	Mitochondrion organization	5	1	287	0.5018
GO:0007030	XLOC_024239	Biological Process	Golgi organization	5	1	287	0.5119
GO:0001503	XLOC_023762	Biological Process	Ossification	5	1	287	0.5411
GO:0042391	XLOC_024806	Biological Process	Regulation of membrane potential	5	1	287	0.5504
GO:0007059	XLOC_024104	Biological Process	Chromosome segregation	5	1	287	0.5858
GO:0035264	XLOC_024251	Biological Process	Multicellular organism growth	5	1	287	0.6263
GO:0008104	XLOC_012279	Biological Process	Protein localization	5	1	287	0.6413
GO:0051607	XLOC_024161	Biological Process	Defense response to virus	5	1	287	0.6627
GO:0060021	XLOC_023651	Biological Process	Palate development	5	1	287	0.6696
GO:0045087	XLOC_024021	Biological Process	Innate immune response	5	1	287	0.7139
GO:0007154	XLOC_002847	Biological Process	Cell communication	5	1	287	0.7310
GO:0006979	XLOC_023606	Biological Process	Response to oxidative stress	5	1	287	0.7573
GO:0006810	XLOC_001311;XLOC_002497;XLOC_003157;XLOC_003464;XLOC_004385;XLOC_023448;XLOC_023730;XLOC_023813;XLOC_023917;XLOC_023978;XLOC_024157;XLOC_024260;XLOC_024806;XLOC_026899	Biological Process	Transport	5	14	287	0.8177
GO:0042127	XLOC_023978	Biological Process	Regulation of cell proliferation	5	1	287	0.8636
GO:0007275	XLOC_015126;XLOC_020726;XLOC_023762	Biological Process	Multicellular organismal development	5	3	287	0.8663
GO:0051301	XLOC_023745	Biological Process	Cell division	5	1	287	0.8890
GO:0008285	XLOC_023558;XLOC_023581	Biological Process	Negative regulation of cell proliferation	5	2	287	0.9429
GO:0007049	XLOC_023745	Biological Process	Cell cycle	5	1	287	0.9711
GO:0009653	XLOC_023651;XLOC_024239	Biological Process	Anatomical structure morphogenesis	4	2	287	0.0106

GO:0009893	XLOC_023552	Biological Process	Positive regulation of metabolic process	4	1	287	0.0401
GO:0003006	XLOC_012858	Biological Process	Developmental process involved in reproduction	4	1	287	0.0596
GO:0051775	XLOC_023529	Biological Process	Response to redox state	4	1	287	0.0786
GO:0007626	XLOC_005548;XLOC_013602;XLOC_024239	Biological Process	Locomotory behavior	4	3	287	0.0805
GO:0050793	XLOC_015126	Biological Process	Regulation of developmental process	4	1	287	0.0973
GO:0043473	XLOC_020670;XLOC_023653	Biological Process	Pigmentation	4	2	287	0.1436
GO:0006996	XLOC_015335	Biological Process	Organelle organization	4	1	287	0.1852
GO:0042445	XLOC_024239	Biological Process	Hormone metabolic process	4	1	287	0.2017
GO:0007623	XLOC_011222	Biological Process	Circadian rhythm	4	1	287	0.3083
GO:0050900	XLOC_023923	Biological Process	Leukocyte migration	4	1	287	0.3496
GO:0006955	XLOC_015580;XLOC_023720;XLOC_024021;XLOC_024161	Biological Process	Immune response	4	4	287	0.3893
GO:0019882	XLOC_015580	Biological Process	Antigen processing and presentation	4	1	287	0.5411
GO:0044237	XLOC_002857	Biological Process	Cellular metabolic process	4	1	287	0.5595
GO:0007155	XLOC_003749;XLOC_020890;XLOC_023834;XLOC_024099;XLOC_024165	Biological Process	Cell adhesion	4	5	287	0.5850
GO:0032259	XLOC_023522;XLOC_023892	Biological Process	Methylation	4	2	287	0.6209
GO:0009058	XLOC_024196	Biological Process	Biosynthetic process	4	1	287	0.6957
GO:0006950	XLOC_023512	Biological Process	Response to stress	4	1	287	0.8180
GO:0008283	XLOC_002615	Biological Process	Cell proliferation	4	1	287	0.9151
GO:0007610	XLOC_013602;XLOC_024073;XLOC_024239	Biological Process	Behavior	3	3	287	0.0073
GO:0022414	XLOC_024180	Biological Process	Reproductive process	3	1	287	0.0786
GO:0009987	XLOC_021864;XLOC_023953	Biological Process	Cellular process	3	2	287	0.1032
GO:0008152	XLOC_002857;XLOC_011326;XLOC_012858;XLOC_017018;XLOC_023444;XLOC_023501;XLOC_023509;XLOC_023825;XLOC_023853;XLOC_024088;XLOC_024219;XLOC_024273;XLOC_024302;XLOC_024425	Biological Process	Metabolic process	3	14	287	0.2528
GO:0040007	XLOC_023762	Biological Process	Growth	3	1	287	0.4130
GO:0008150	XLOC_002615	Biological Process	Biological_process	2	1	287	0.3361
GO:0001950	XLOC_001463	Cellular Component	Plasma membrane enriched fraction	NA	1	287	0.1511

GO:0019717	XLOC_001463;XLOC_015335	Cellular Component	Synaptosome	NA	2	287	0.2090
GO:0005792	XLOC_001463;XLOC_023512;XLOC_023730	Cellular Component	Microsome	NA	3	287	0.2531
GO:0005626	XLOC_022857	Cellular Component	Insoluble fraction	NA	1	287	0.2940
GO:0005624	XLOC_002956;XLOC_003605;XLOC_023834;XLOC_023853;XLOC_024239	Cellular Component	Membrane fraction	NA	5	287	0.3093
GO:0005625	XLOC_024239	Cellular Component	Soluble fraction	NA	1	287	0.8104
GO:0000276	XLOC_024260	Cellular Component	Mitochondrial proton-transporting ATP synthase complex, coupling factor F(o)	15	1	287	0.1156
GO:0031305	XLOC_024147	Cellular Component	Integral component of mitochondrial inner membrane	15	1	287	0.1852
GO:0031304	XLOC_020095	Cellular Component	Intrinsic component of mitochondrial inner membrane	14	1	287	0.0401
GO:0031314	XLOC_024098	Cellular Component	Extrinsic component of mitochondrial inner membrane	14	1	287	0.0786
GO:0071458	XLOC_023978	Cellular Component	Integral component of cytoplasmic side of endoplasmic reticulum membrane	14	1	287	0.0786
GO:0071556	XLOC_023978	Cellular Component	Integral component of lumenal side of endoplasmic reticulum membrane	14	1	287	0.0786
GO:0005847	XLOC_023648	Cellular Component	mRNA cleavage and polyadenylation specificity factor complex	14	1	287	0.1683
GO:0005719	XLOC_022472	Cellular Component	Nuclear euchromatin	14	1	287	0.2794
GO:0005753	XLOC_024260	Cellular Component	Mitochondrial proton-transporting ATP synthase complex	14	1	287	0.2940
GO:0016607	XLOC_024265	Cellular Component	Nuclear speck	14	1	287	0.7764
GO:0030670	XLOC_023756	Cellular Component	Phagocytic vesicle membrane	13	1	287	0.0973
GO:0042582	XLOC_001463	Cellular Component	Azurophil granule	13	1	287	0.1156
GO:0000780	XLOC_024104	Cellular Component	Condensed nuclear chromosome, centromeric region	13	1	287	0.1511
GO:0005721	XLOC_024104	Cellular Component	Pericentric heterochromatin	13	1	287	0.1511
GO:0005743	XLOC_002962;XLOC_023626;XLOC_023920;XLOC_024070;XLOC_024098;XLOC_024260;XLOC_024278;XLOC_025657	Cellular Component	Mitochondrial inner membrane	13	8	287	0.1519
GO:0005762	XLOC_003706	Cellular Component	Mitochondrial large ribosomal subunit	13	1	287	0.2179
GO:0030018	XLOC_023512;XLOC_023962	Cellular Component	Z disc	13	2	287	0.2540
GO:0030173	XLOC_023922	Cellular Component	Integral component of Golgi membrane	13	1	287	0.4480

GO:0016604	XLOC_022857	Cellular Component	Nuclear body	13	1	287	0.5119
GO:0042581	XLOC_001463;XLOC_023756	Cellular Component	Specific granule	12	2	287	0.0236
GO:0031083	XLOC_015335;XLOC_023861	Cellular Component	BLOC-1 complex	12	2	287	0.0275
GO:0030134	XLOC_024191	Cellular Component	ER to Golgi transport vesicle	12	1	287	0.0973
GO:0043202	XLOC_024165	Cellular Component	Lysosomal lumen	12	1	287	0.0973
GO:0071986	XLOC_023520	Cellular Component	Ragulator complex	12	1	287	0.0973
GO:0000786	XLOC_015255;XLOC_015261;XLOC_023518	Cellular Component	Nucleosome	12	3	287	0.1308
GO:0005663	XLOC_024024	Cellular Component	DNA replication factor C complex	12	1	287	0.1335
GO:0035371	XLOC_023632	Cellular Component	Microtubule plus-end	12	1	287	0.1683
GO:0008250	XLOC_023918	Cellular Component	Oligosaccharyltransferase complex	12	1	287	0.2017
GO:0031941	XLOC_023978	Cellular Component	Filamentous actin	12	1	287	0.3224
GO:0030672	XLOC_015335	Cellular Component	Synaptic vesicle membrane	12	1	287	0.3361
GO:0000792	XLOC_023963	Cellular Component	Heterochromatin	12	1	287	0.3628
GO:0005643	XLOC_023439	Cellular Component	Nuclear pore	12	1	287	0.4702
GO:0022627	XLOC_028013	Cellular Component	Cytosolic small ribosomal subunit	12	1	287	0.5119
GO:0001725	XLOC_023745	Cellular Component	Stress fiber	12	1	287	0.5595
GO:0042470	XLOC_023620	Cellular Component	Melanosome	12	1	287	0.5858
GO:0031966	XLOC_020095	Cellular Component	Mitochondrial membrane	12	1	287	0.6413
GO:0005765	XLOC_024165	Cellular Component	Lysosomal membrane	12	1	287	0.6829
GO:0031965	XLOC_003316	Cellular Component	Nuclear membrane	12	1	287	0.8289
GO:0030127	XLOC_023448;XLOC_023917	Cellular Component	COPII vesicle coat	11	2	287	0.0134
GO:0045180	XLOC_023978	Cellular Component	Basal cortex	11	1	287	0.0596
GO:0000221	XLOC_003625	Cellular Component	Vacuolar proton-transporting V-type ATPase, V1 domain	11	1	287	0.0786
GO:0000506	XLOC_023552	Cellular Component	Glycosylphosphatidylinositol-N-acetylglucosaminyltransferase (GPI-GnT) complex	11	1	287	0.0973
GO:0005826	XLOC_023834	Cellular Component	Actomyosin contractile ring	11	1	287	0.1335
GO:0070531	XLOC_024203	Cellular Component	BRCA1-A complex	11	1	287	0.1511
GO:0031105	XLOC_023745	Cellular Component	Septin complex	11	1	287	0.2645
GO:0032039	XLOC_023494	Cellular Component	Integrator complex	11	1	287	0.2645
GO:0031902	XLOC_023520	Cellular Component	Late endosome membrane	11	1	287	0.3496
GO:0015935	XLOC_028013	Cellular Component	Small ribosomal subunit	11	1	287	0.3757
GO:0030133	XLOC_023908	Cellular Component	Transport vesicle	11	1	287	0.3884
GO:0008180	XLOC_023777	Cellular Component	COP9 signalosome	11	1	287	0.4480
GO:0000139	XLOC_002497;XLOC_003678;XLOC_023730	Cellular Component	Golgi membrane	11	3	287	0.4665
GO:0030659	XLOC_024239	Cellular Component	Cytoplasmic vesicle membrane	11	1	287	0.5018
GO:0005882	XLOC_011437	Cellular Component	Intermediate filament	11	1	287	0.5504
GO:0022625	XLOC_002615	Cellular Component	Cytosolic large ribosomal subunit	11	1	287	0.5685
GO:0010008	XLOC_002935	Cellular Component	Endosome membrane	11	1	287	0.5943
GO:0016363	XLOC_023834	Cellular Component	Nuclear matrix	11	1	287	0.6185
GO:0000785	XLOC_024034	Cellular Component	Chromatin	11	1	287	0.6557
GO:0005789	XLOC_015335;XLOC_023552;XLOC_023978;XLOC_024190	Cellular Component	Endoplasmic reticulum membrane	11	4	287	0.7017

GO:0005730	XLOC_015261;XLOC_022857;XLOC_023581;XLOC_023666;XLOC_023807;XLOC_023908;XLOC_024160;XLOC_024201	Cellular Component	Nucleolus	11	8	287	0.7145
GO:0005764	XLOC_021577;XLOC_024165	Cellular Component	Lysosome	11	2	287	0.7475
GO:0005654	XLOC_015261;XLOC_022857	Cellular Component	Nucleoplasm	11	2	287	0.7729
GO:0005635	XLOC_024206	Cellular Component	Nuclear envelope	11	1	287	0.7855
GO:0005759	XLOC_023553	Cellular Component	Mitochondrial matrix	11	1	287	0.7983
GO:0005874	XLOC_002602	Cellular Component	Microtubule	11	1	287	0.9571
GO:0005776	XLOC_023863;XLOC_024206;XLOC_024239	Cellular Component	Autophagic vacuole	10	3	287	0.0243
GO:0031429	XLOC_023915	Cellular Component	Box H/ACA snoRNP complex	10	1	287	0.0401
GO:0033391	XLOC_023531	Cellular Component	Chromatoid body	10	1	287	0.0596
GO:0044427	XLOC_023835	Cellular Component	Chromosomal part	10	1	287	0.0596
GO:0005786	XLOC_023666	Cellular Component	Signal recognition particle, endoplasmic reticulum targeting	10	1	287	0.1156
GO:0033017	XLOC_023465	Cellular Component	Sarcoplasmic reticulum membrane	10	1	287	0.2179
GO:0005770	XLOC_023726;XLOC_024239	Cellular Component	Late endosome	10	2	287	0.3142
GO:0072686	XLOC_023978	Cellular Component	Mitotic spindle	10	1	287	0.3361
GO:0016023	XLOC_020890;XLOC_024239	Cellular Component	Cytoplasmic membrane-bounded vesicle	10	2	287	0.3878
GO:0005802	XLOC_003322;XLOC_023564	Cellular Component	Trans-Golgi network	10	2	287	0.4233
GO:0000932	XLOC_023531	Cellular Component	Cytoplasmic mRNA processing body	10	1	287	0.5411
GO:0015630	XLOC_023632;XLOC_024015	Cellular Component	Microtubule cytoskeleton	10	2	287	0.5938
GO:0000922	XLOC_002602	Cellular Component	Spindle pole	10	1	287	0.6763
GO:0005769	XLOC_023726	Cellular Component	Early endosome	10	1	287	0.7718
GO:0015629	XLOC_015261;XLOC_023834	Cellular Component	Actin cytoskeleton	10	2	287	0.7797
GO:0005777	XLOC_025902	Cellular Component	Peroxisome	10	1	287	0.7941
GO:0016012	XLOC_024072	Cellular Component	Sarcoglycan complex	9	1	287	0.0973
GO:0031462	XLOC_003625	Cellular Component	Cul2-RING ubiquitin ligase complex	9	1	287	0.1156
GO:0048500	XLOC_023666	Cellular Component	Signal recognition particle	9	1	287	0.1335
GO:0042622	XLOC_024296	Cellular Component	Photoreceptor outer segment membrane	9	1	287	0.1511

GO:0005856	XLOC_002602;XLOC_023621;XLOC_023632;XLOC_023745;XLOC_023786;XLOC_023834;XLOC_024072;XLOC_024251;XLOC_024265	Cellular Component	Cytoskeleton	9	9	287	0.1882
GO:0005768	XLOC_002935;XLOC_023520;XLOC_023637;XLOC_024070;XLOC_024081	Cellular Component	Endosome	9	5	287	0.1958
GO:0032391	XLOC_023603	Cellular Component	Photoreceptor connecting cilium	9	1	287	0.2645
GO:0005694	XLOC_015255;XLOC_015261;XLOC_023518;XLOC_023835	Cellular Component	Chromosome	9	4	287	0.3019
GO:0005680	XLOC_023568	Cellular Component	Anaphase-promoting complex	9	1	287	0.3361
GO:0005634	XLOC_001463;XLOC_002497;XLOC_002509;XLOC_002615;XLOC_002956;XLOC_003180;XLOC_003207;XLOC_003316;XLOC_003322;XLOC_003350;XLOC_003605;XLOC_003747;XLOC_005548;XLOC_011222;XLOC_012182;XLOC_012264;XLOC_012452;XLOC_013257;XLOC_013557;XLOC_013602;XLOC_015255;XLOC_015261;XLOC_015335;XLOC_020726;XLOC_021996;XLOC_022472;XLOC_022857;XLOC_023439;XLOC_023481;XLOC_023494;XLOC_023512;XLOC_023518;XLOC_023555;XLOC_023564;XLOC_023568;XLOC_023581;XLOC_023598;XLOC_023621;XLOC_023648;XLOC_023651;XLOC_023653;XLOC_023702;XLOC_023	Cellular Component	Nucleus	9	76	287	0.3741
GO:0005689	XLOC_023994	Cellular Component	U12-type spliceosomal complex	9	1	287	0.3884
GO:0016529	XLOC_023465	Cellular Component	Sarcoplasmic reticulum	9	1	287	0.3884

GO:0005739	XLOC_002658;XLOC_002857;XLOC_002962;XLOC_003316;XLOC_003674;XLOC_003697;XLOC_003706;XLOC_011326;XLOC_020095;XLOC_023495;XLOC_023512;XLOC_023529;XLOC_023552;XLOC_023553;XLOC_023555;XLOC_023606;XLOC_023626;XLOC_023637;XLOC_023788;XLOC_023920;XLOC_024070;XLOC_024098;XLOC_024141;XLOC_024260;XLOC_024278;XLOC_024302;XLOC_025657	Cellular Component	Mitochondrion	9	27	287	0.3992
GO:0019005	XLOC_024292	Cellular Component	SCF ubiquitin ligase complex	9	1	287	0.4008
GO:0005819	XLOC_002602;XLOC_023834	Cellular Component	Spindle	9	2	287	0.4163
GO:0005811	XLOC_024219	Cellular Component	Lipid particle	9	1	287	0.4366
GO:0000776	XLOC_023439;XLOC_024104	Cellular Component	Kinetochore	9	2	287	0.4441
GO:0048471	XLOC_023439;XLOC_023730;XLOC_024191;XLOC_024292;XLOC_024304	Cellular Component	Perinuclear region of cytoplasm	9	5	287	0.6093
GO:0005794	XLOC_001463;XLOC_002497;XLOC_003322;XLOC_023480;XLOC_023726;XLOC_023730;XLOC_023908;XLOC_023922;XLOC_024081;XLOC_024239;XLOC_024264	Cellular Component	Golgi apparatus	9	11	287	0.6161
GO:0005840	XLOC_002615;XLOC_003316;XLOC_028013	Cellular Component	Ribosome	9	3	287	0.7503
GO:0071013	XLOC_012182	Cellular Component	Catalytic step 2 spliceosome	9	1	287	0.7855

GO:0005829	XLOC_005548;XLOC_023439;XLOC_023481;XLOC_023512;XLOC_023596;XLOC_023730;XLOC_023941;XLOC_024239;XLOC_024265	Cellular Component	Cytosol	9	9	287	0.7937
GO:0031410	XLOC_001463;XLOC_003206	Cellular Component	Cytoplasmic vesicle	9	2	287	0.8022
GO:0005783	XLOC_002857;XLOC_023480;XLOC_023666;XLOC_023825;XLOC_023978;XLOC_024190;XLOC_024191;XLOC_024239	Cellular Component	Endoplasmic reticulum	9	8	287	0.8709
GO:0005813	XLOC_023428;XLOC_023632;XLOC_024802	Cellular Component	Centrosome	9	3	287	0.9389
GO:0031513	XLOC_023603;XLOC_023978	Cellular Component	Nonmotile primary cilium	8	2	287	0.0106
GO:0043197	XLOC_015335;XLOC_023745	Cellular Component	Dendritic spine	8	2	287	0.0667
GO:0005732	XLOC_023428	Cellular Component	Small nucleolar ribonucleoprotein complex	8	1	287	0.0786
GO:0016528	XLOC_015335	Cellular Component	Sarcoplasm	8	1	287	0.0786
GO:0033267	XLOC_015335	Cellular Component	Axon part	8	1	287	0.0973
GO:0031932	XLOC_002615	Cellular Component	TORC2 complex	8	1	287	0.1335
GO:0031512	XLOC_023978	Cellular Component	Motile primary cilium	8	1	287	0.1683
GO:0045263	XLOC_024260	Cellular Component	Proton-transporting ATP synthase complex, coupling factor F(o)	8	1	287	0.2017
GO:0031011	XLOC_003180	Cellular Component	Ino80 complex	8	1	287	0.2179
GO:0042613	XLOC_015580	Cellular Component	MHC class II protein complex	8	1	287	0.2337
GO:0005637	XLOC_002497	Cellular Component	Nuclear inner membrane	8	1	287	0.2940
GO:0044444	XLOC_024206	Cellular Component	Cytoplasmic part	8	1	287	0.3083
GO:0008076	XLOC_026899	Cellular Component	Voltage-gated potassium channel complex	8	1	287	0.4249
GO:0043231	XLOC_002935;XLOC_003207;XLOC_023726;XLOC_024191	Cellular Component	Intracellular membrane-bounded organelle	8	4	287	0.7958
GO:0072563	XLOC_002956;XLOC_003605;XLOC_023432	Cellular Component	Endothelial microparticle	7	3	287	0.0003
GO:0017101	XLOC_023923	Cellular Component	Aminoacyl-tRNA synthetase multienzyme complex	7	1	287	0.0401

GO:0005737	XLOC_002509;XLOC_002602;XLOC_002935;XLOC_002956;XLOC_003107;XLOC_003180;XLOC_003322;XLOC_003350;XLOC_003605;XLOC_003697;XLOC_005548;XLOC_010535;XLOC_011982;XLOC_012182;XLOC_012264;XLOC_013257;XLOC_013557;XLOC_013602;XLOC_015335;XLOC_017018;XLOC_018228;XLOC_020726;XLOC_021864;XLOC_021996;XLOC_022812;XLOC_022857;XLOC_023428;XLOC_023481;XLOC_023495;XLOC_023501;XLOC_023512;XLOC_023529;XLOC_023531;XLOC_023555;XLOC_023558;XLOC_023566;XLOC_023581;XLOC_023609;XLOC_023632;XLOC_023653;XLOC_023666;XLOC_023702;XLOC_023	Cellular Component	Cytoplasm	7	81	287	0.0454
GO:0005887	XLOC_020890;XLOC_023651;XLOC_023834;XLOC_023922;XLOC_023978;XLOC_024081;XLOC_024296	Cellular Component	Integral component of plasma membrane	7	7	287	0.0537
GO:0005584	XLOC_010535	Cellular Component	Collagen type I trimer	7	1	287	0.0596
GO:0035355	XLOC_024021	Cellular Component	Toll-like receptor 2-Toll-like receptor 6 protein complex	7	1	287	0.0596
GO:0044441	XLOC_023978	Cellular Component	Ciliary part	7	1	287	0.0596
GO:0005588	XLOC_003749	Cellular Component	Collagen type V trimer	7	1	287	0.0786
GO:0005955	XLOC_023512	Cellular Component	Calcineurin complex	7	1	287	0.0786
GO:0031226	XLOC_023651	Cellular Component	Intrinsic component of plasma membrane	7	1	287	0.1156
GO:0030424	XLOC_005548;XLOC_015335;XLOC_023745;XLOC_024239	Cellular Component	Axon	7	4	287	0.1270
GO:0016010	XLOC_024072	Cellular Component	Dystrophin-associated glycoprotein complex	7	1	287	0.1511
GO:0016234	XLOC_024239	Cellular Component	Inclusion body	7	1	287	0.1683
GO:0005845	XLOC_023531	Cellular Component	mRNA cap binding complex	7	1	287	0.1852
GO:0030014	XLOC_024173	Cellular Component	CCR4-NOT complex	7	1	287	0.2337
GO:0001891	XLOC_025523	Cellular Component	Phagocytic cup	7	1	287	0.2493
GO:0030315	XLOC_003206	Cellular Component	T-tubule	7	1	287	0.2493
GO:0005605	XLOC_012279	Cellular Component	Basal lamina	7	1	287	0.2794
GO:0032420	XLOC_024296	Cellular Component	Stereocilium	7	1	287	0.2794

GO:0070062	XLOC_012279;XLOC_023432	Cellular Component	Extracellular vesicular exosome	7	2	287	0.3366
GO:0009925	XLOC_023978	Cellular Component	Basal plasma membrane	7	1	287	0.3757
GO:0031514	XLOC_023603	Cellular Component	Motile cilium	7	1	287	0.3757
GO:0030425	XLOC_005548;XLOC_024239	Cellular Component	Dendrite	7	2	287	0.4441
GO:0000151	XLOC_024155	Cellular Component	Ubiquitin ligase complex	7	1	287	0.5411
GO:0005667	XLOC_002509;XLOC_023581;XLOC_024273	Cellular Component	Transcription factor complex	7	3	287	0.5891
GO:0030529	XLOC_002615;XLOC_003706;XLOC_023903;XLOC_028013	Cellular Component	Ribonucleoprotein complex	7	4	287	0.7381
GO:0009897	XLOC_002956;XLOC_003605;XLOC_018924;XLOC_023432;XLOC_023651;XLOC_023653;XLOC_023990;XLOC_024081;XLOC_024161	Cellular Component	External side of plasma membrane	6	9	287	0.0045
GO:0042383	XLOC_003206;XLOC_015335;XLOC_023465;XLOC_024072	Cellular Component	Sarcolemma	6	4	287	0.0160
GO:0016028	XLOC_003107	Cellular Component	Rhabdomere	6	1	287	0.0401
GO:0030991	XLOC_023603	Cellular Component	Intraciliary transport particle A	6	1	287	0.0786
GO:0031225	XLOC_012858	Cellular Component	Anchored component of membrane	6	1	287	0.2645
GO:0005903	XLOC_024296	Cellular Component	Brush border	6	1	287	0.3083
GO:0043005	XLOC_005548;XLOC_015335	Cellular Component	Neuron projection	6	2	287	0.3587
GO:0005902	XLOC_024296	Cellular Component	Microvillus	6	1	287	0.4008
GO:0030426	XLOC_015335	Cellular Component	Growth cone	6	1	287	0.4130
GO:0005604	XLOC_003749;XLOC_012279	Cellular Component	Basement membrane	6	2	287	0.4163

GO:0016021	XLOC_001311;XLOC_002497;XLOC_002602;XLOC_003107;XLOC_003157;XLOC_003206;XLOC_003464;XLOC_004385;XLOC_007550;XLOC_008893;XLOC_015126;XLOC_015580;XLOC_021577;XLOC_023519;XLOC_023532;XLOC_023620;XLOC_023621;XLOC_023651;XLOC_023653;XLOC_023808;XLOC_023813;XLOC_023909;XLOC_023918;XLOC_023978;XLOC_023990;XLOC_023997;XLOC_024015;XLOC_024021;XLOC_024028;XLOC_024072;XLOC_024081;XLOC_024147;XLOC_024165;XLOC_024180;XLOC_024188;XLOC_024190;XLOC_024196;XLOC_024261;XLOC_024296;XLOC_024425;XLOC_024806;XLOC_025657;XLOC_026	Cellular Component	Integral component of membrane	6	45	287	0.4509
GO:0005929	XLOC_023603;XLOC_023978	Cellular Component	Cilium	6	2	287	0.5228
GO:0030027	XLOC_023978	Cellular Component	Lamellipodium	6	1	287	0.8142
GO:0043025	XLOC_005548	Cellular Component	Neuronal cell body	6	1	287	0.8425
GO:0016324	XLOC_024296	Cellular Component	Apical plasma membrane	6	1	287	0.8744
GO:0005615	XLOC_002956;XLOC_003107;XLOC_003605;XLOC_010535;XLOC_012279;XLOC_020267;XLOC_023558;XLOC_023564;XLOC_023653;XLOC_023720;XLOC_023762;XLOC_023848;XLOC_024099;XLOC_024161;XLOC_024220;XLOC_024296	Cellular Component	Extracellular space	5	16	287	0.0037

GO:0009986	XLOC_002956;XLOC_003605;XLOC_015126;XLOC_021577;XLOC_023297;XLOC_023558;XLOC_023651;XLOC_023653;XLOC_023853;XLOC_023923;XLOC_024296	Cellular Component	Cell surface	5	11	287	0.0195
GO:0002133	XLOC_023978	Cellular Component	Polycystin complex	5	1	287	0.0596
GO:0005578	XLOC_002589;XLOC_010535;XLOC_012279;XLOC_017359;XLOC_024220	Cellular Component	Proteinaceous extracellular matrix	5	5	287	0.0869
GO:0002193	XLOC_023581	Cellular Component	MAML1-RBP-Jkappa- ICN1 complex	5	1	287	0.1156
GO:0031224	XLOC_024021	Cellular Component	Intrinsic component of membrane	5	1	287	0.2337
GO:0044297	XLOC_005548	Cellular Component	Cell body	5	1	287	0.2493
GO:0008287	XLOC_023553	Cellular Component	Protein serine/threonine phosphatase complex	5	1	287	0.2645
GO:0043235	XLOC_021577	Cellular Component	Receptor complex	5	1	287	0.2940
GO:0019898	XLOC_024206	Cellular Component	Extrinsic component of membrane	5	1	287	0.3884
GO:0071944	XLOC_024081	Cellular Component	Cell periphery	5	1	287	0.4809
GO:0017053	XLOC_024273	Cellular Component	Transcriptional repressor complex	5	1	287	0.5772
GO:0070469	XLOC_025657	Cellular Component	Respiratory chain	5	1	287	0.6339
GO:0042995	XLOC_023745;XLOC_024296	Cellular Component	Cell projection	5	2	287	0.6565
GO:0005886	XLOC_001311;XLOC_001463;XLOC_002935;XLOC_003180;XLOC_008893;XLOC_012858;XLOC_015335;XLOC_020726;XLOC_021577;XLOC_023465;XLOC_023653;XLOC_023666;XLOC_023726;XLOC_023756;XLOC_023978;XLOC_023990;XLOC_024015;XLOC_024034;XLOC_024072;XLOC_024253;XLOC_024265;XLOC_024287;XLOC_024296;XLOC_028154	Cellular Component	Plasma membrane	5	24	287	0.7728
GO:0045121	XLOC_024081	Cellular Component	Membrane raft	5	1	287	0.7898

GO:0005622	XLOC_002615;XLOC_002772;XLOC_002973;XLOC_003207;XLOC_003316;XLOC_005548;XLOC_008893;XLOC_009333;XLOC_009917;XLOC_012452;XLOC_015255;XLOC_016622;XLOC_022857;XLOC_023432;XLOC_023480;XLOC_023491;XLOC_023555;XLOC_023598;XLOC_023599;XLOC_023801;XLOC_023863;XLOC_023974;XLOC_023975;XLOC_024021;XLOC_024157;XLOC_024185;XLOC_024235;XLOC_024284;XLOC_024287;XLOC_028013	Cellular Component	Intracellular	5	30	287	0.9103
GO:0031090	XLOC_023510	Cellular Component	Organelle membrane	4	1	287	0.0973
GO:0014069	XLOC_015335	Cellular Component	Postsynaptic density	4	1	287	0.4809
GO:0005911	XLOC_023978	Cellular Component	Cell-cell junction	4	1	287	0.8254
GO:0043234	XLOC_022857;XLOC_024239	Cellular Component	Protein complex	4	2	287	0.8281
GO:0005576	XLOC_000144;XLOC_001060;XLOC_001362;XLOC_002550;XLOC_002593;XLOC_010535;XLOC_013257;XLOC_017359;XLOC_020267;XLOC_023435;XLOC_023480;XLOC_023558;XLOC_023720;XLOC_023762;XLOC_023848;XLOC_024099;XLOC_024161;XLOC_024280	Cellular Component	Extracellular region	3	18	287	0.0290
GO:0031012	XLOC_002589;XLOC_003749;XLOC_010535;XLOC_012279;XLOC_023435;XLOC_023564;XLOC_024099	Cellular Component	Extracellular matrix	3	7	287	0.0483

GO:0005581	XLOC_003749;XLOC_010535;XLOC_023848	Cellular Component	Collagen trimer	3	3	287	0.0882
GO:0016020	XLOC_001311;XLOC_001463;XLOC_002497;XLOC_002602;XLOC_002935;XLOC_002962;XLOC_003107;XLOC_003157;XLOC_003322;XLOC_003464;XLOC_004385;XLOC_008893;XLOC_012858;XLOC_015126;XLOC_015580;XLOC_023297;XLOC_023465;XLOC_023510;XLOC_023519;XLOC_023520;XLOC_023532;XLOC_023626;XLOC_023651;XLOC_023653;XLOC_023730;XLOC_023756;XLOC_023801;XLOC_023808;XLOC_023813;XLOC_023853;XLOC_023909;XLOC_023918;XLOC_023922;XLOC_023923;XLOC_023970;XLOC_023978;XLOC_023990;XLOC_023997;XLOC_024015;XLOC_024021;XLOC_024028;XLOC_024072;XLOC_024	Cellular Component	Membrane	3	56	287	0.5901
GO:0030054	XLOC_023428;XLOC_023745	Cellular Component	Cell junction	3	2	287	0.8557
GO:0045202	XLOC_023745	Cellular Component	Synapse	3	1	287	0.9281
GO:0005575	XLOC_024302	Cellular Component	Cellular_component	2	1	287	0.3757
GO:0043498	XLOC_023297;XLOC_023923	Molecular Function	Cell surface binding	NA	2	287	0.0134
GO:0043499	XLOC_023297;XLOC_023432	Molecular Function	Eukaryotic cell surface binding	NA	2	287	0.0558
GO:0046961	XLOC_003625	Molecular Function	Proton-transporting ATPase activity, rotational mechanism	16	1	287	0.2940
GO:0003689	XLOC_024024	Molecular Function	DNA clamp loader activity	13	1	287	0.0401
GO:0048763	XLOC_023978	Molecular Function	Calcium-induced calcium release activity	12	1	287	0.0596
GO:0005018	XLOC_023651	Molecular Function	Platelet-derived growth factor alpha-receptor activity	11	1	287	0.0401
GO:0000978	XLOC_011222;XLOC_023581	Molecular Function	RNA polymerase II core promoter proximal region sequence-specific DNA binding	11	2	287	0.0843

GO:0033192	XLOC_023512	Molecular Function	Calmodulin-dependent protein phosphatase activity	11	1	287	0.0973
GO:0005249	XLOC_024806;XLOC_026899	Molecular Function	Voltage-gated potassium channel activity	11	2	287	0.1507
GO:0005248	XLOC_023978	Molecular Function	Voltage-gated sodium channel activity	11	1	287	0.1852
GO:0005245	XLOC_023978	Molecular Function	Voltage-gated calcium channel activity	11	1	287	0.3361
GO:0000979	XLOC_023581	Molecular Function	RNA polymerase II core promoter sequence-specific DNA binding	11	1	287	0.3757
GO:0008026	XLOC_023994	Molecular Function	ATP-dependent helicase activity	11	1	287	0.8425
GO:0005021	XLOC_023651;XLOC_024081	Molecular Function	Vascular endothelial growth factor-activated receptor activity	10	2	287	0.0106
GO:0004137	XLOC_023703	Molecular Function	Deoxycytidine kinase activity	10	1	287	0.0401
GO:0005020	XLOC_023653	Molecular Function	Stem cell factor receptor activity	10	1	287	0.0401
GO:0022843	XLOC_023978	Molecular Function	Voltage-gated cation channel activity	10	1	287	0.0401
GO:0005024	XLOC_002956;XLOC_003605	Molecular Function	Transforming growth factor beta-activated receptor activity	10	2	287	0.0558
GO:0004692	XLOC_024183	Molecular Function	cGMP-dependent protein kinase activity	10	1	287	0.0786
GO:0046404	XLOC_023611	Molecular Function	ATP-dependent polydeoxyribonucleotide 5'-hydroxyl-kinase activity	10	1	287	0.0786
GO:0047555	XLOC_023444	Molecular Function	3',5'-cyclic-GMP phosphodiesterase activity	10	1	287	0.0786
GO:0008508	XLOC_003157	Molecular Function	Bile acid:sodium symporter activity	10	1	287	0.0973
GO:0004439	XLOC_003678	Molecular Function	Phosphatidylinositol-4,5-bisphosphate 5-phosphatase activity	10	1	287	0.1335
GO:0004522	XLOC_001362	Molecular Function	Pancreatic ribonuclease activity	10	1	287	0.1683
GO:0005267	XLOC_023978;XLOC_026899	Molecular Function	Potassium channel activity	10	2	287	0.2090
GO:0015078	XLOC_024260	Molecular Function	Hydrogen ion transmembrane transporter activity	10	1	287	0.3361
GO:0005262	XLOC_023978	Molecular Function	Calcium channel activity	10	1	287	0.5685
GO:0004714	XLOC_023651;XLOC_023653;XLOC_024081	Molecular Function	Transmembrane receptor protein tyrosine kinase activity	9	3	287	0.0310
GO:0046873	XLOC_004385;XLOC_023510	Molecular Function	Metal ion transmembrane transporter activity	9	2	287	0.0361
GO:0000210	XLOC_025902	Molecular Function	NAD+ diphosphatase activity	9	1	287	0.0401
GO:0035529	XLOC_025902	Molecular Function	NADH pyrophosphatase activity	9	1	287	0.0401
GO:0043262	XLOC_023788	Molecular Function	Adenosine-diphosphatase activity	9	1	287	0.0596
GO:0004683	XLOC_023465	Molecular Function	Calmodulin-dependent protein kinase activity	9	1	287	0.1156
GO:0004703	XLOC_024247	Molecular Function	G-protein coupled receptor kinase activity	9	1	287	0.1335
GO:0008253	XLOC_017018	Molecular Function	5'-nucleotidase activity	9	1	287	0.1335

GO:0015179	XLOC_001311	Molecular Function	L-amino acid transmembrane transporter activity	9	1	287	0.1335
GO:0030553	XLOC_023444	Molecular Function	cGMP binding	9	1	287	0.1335
GO:0008199	XLOC_001060	Molecular Function	ferric iron binding	9	1	287	0.1852
GO:0046965	XLOC_009333	Molecular Function	Retinoid X receptor binding	9	1	287	0.2337
GO:0001047	XLOC_003350	Molecular Function	Core promoter binding	9	1	287	0.3361
GO:0050681	XLOC_003350	Molecular Function	Androgen receptor binding	9	1	287	0.3361
GO:0004114	XLOC_023444	Molecular Function	3',5'-cyclic-nucleotide phosphodiesterase activity	9	1	287	0.3496
GO:0070888	XLOC_011222	Molecular Function	E-box binding	9	1	287	0.3496
GO:0000976	XLOC_003350	Molecular Function	Transcription regulatory region sequence-specific DNA binding	9	1	287	0.4008
GO:0004722	XLOC_023553	Molecular Function	Protein serine/threonine phosphatase activity	9	1	287	0.4592
GO:0019003	XLOC_024287	Molecular Function	GDP binding	9	1	287	0.5504
GO:0004843	XLOC_024073	Molecular Function	Ubiquitin-specific protease activity	9	1	287	0.5595
GO:0005524	XLOC_002973;XLOC_003080;XLOC_003107;XLOC_008893;XLOC_010523;XLOC_011326;XLOC_017920;XLOC_023465;XLOC_023491;XLOC_023609;XLOC_023611;XLOC_023651;XLOC_023653;XLOC_023703;XLOC_023907;XLOC_023963;XLOC_023994;XLOC_024024;XLOC_024081;XLOC_024105;XLOC_024183;XLOC_024247;XLOC_024264;XLOC_025531	Molecular Function	ATP binding	9	24	287	0.6133
GO:0005244	XLOC_023978	Molecular Function	Voltage-gated ion channel activity	9	1	287	0.6486
GO:0004386	XLOC_023963;XLOC_023994	Molecular Function	Helicase activity	9	2	287	0.6894
GO:0005525	XLOC_010523;XLOC_015530;XLOC_023626;XLOC_023745;XLOC_024098;XLOC_024287	Molecular Function	GTP binding	9	6	287	0.7486
GO:0004725	XLOC_023786	Molecular Function	Protein tyrosine phosphatase activity	9	1	287	0.8519
GO:0003924	XLOC_015530;XLOC_023626;XLOC_024287	Molecular Function	GTPase activity	9	3	287	0.8963
GO:0016887	XLOC_003080	Molecular Function	ATPase activity	9	1	287	0.9169

GO:0008235	XLOC_021864;XLOC_023555;XLOC_023953	Molecular Function	Metalloexopeptidase activity	8	3	287	0.0006
GO:0004177	XLOC_021864;XLOC_023555;XLOC_023953	Molecular Function	Aminopeptidase activity	8	3	287	0.0095
GO:0005114	XLOC_002956;XLOC_003605	Molecular Function	Type II transforming growth factor beta receptor binding	8	2	287	0.0134
GO:0034713	XLOC_002956;XLOC_003605	Molecular Function	Type I transforming growth factor beta receptor binding	8	2	287	0.0134
GO:0004035	XLOC_012858	Molecular Function	Alkaline phosphatase activity	8	1	287	0.0401
GO:0004567	XLOC_023509	Molecular Function	Beta-mannosidase activity	8	1	287	0.0401
GO:0018467	XLOC_023529	Molecular Function	Formaldehyde dehydrogenase activity	8	1	287	0.0401
GO:0051267	XLOC_023825	Molecular Function	CP2 mannose-ethanolamine phosphotransferase activity	8	1	287	0.0401
GO:0070740	XLOC_024802	Molecular Function	Tubulin-glutamic acid ligase activity	8	1	287	0.0401
GO:0004112	XLOC_023444	Molecular Function	Cyclic-nucleotide phosphodiesterase activity	8	1	287	0.0596
GO:0004647	XLOC_017018	Molecular Function	Phosphoserine phosphatase activity	8	1	287	0.0596
GO:0005506	XLOC_001806;XLOC_003350;XLOC_011982;XLOC_023921;XLOC_024190;XLOC_024292	Molecular Function	Iron ion binding	8	6	287	0.0636
GO:0003831	XLOC_024206	Molecular Function	Beta-N-acetylglucosaminylglycopeptide beta-1,4-galactosyltransferase activity	8	1	287	0.0786
GO:0031545	XLOC_018228	Molecular Function	Peptidyl-proline 4-dioxygenase activity	8	1	287	0.0786
GO:0047631	XLOC_023788	Molecular Function	ADP-ribose diphosphatase activity	8	1	287	0.0786
GO:0004427	XLOC_023495	Molecular Function	Inorganic diphosphatase activity	8	1	287	0.0973
GO:0004768	XLOC_024190	Molecular Function	Stearoyl-CoA 9-desaturase activity	8	1	287	0.0973
GO:0015101	XLOC_001311	Molecular Function	Organic cation transmembrane transporter activity	8	1	287	0.0973
GO:0008649	XLOC_023928	Molecular Function	rRNA methyltransferase activity	8	1	287	0.1156
GO:0070700	XLOC_023762	Molecular Function	BMP receptor binding	8	1	287	0.1156
GO:0019206	XLOC_023703	Molecular Function	Nucleoside kinase activity	8	1	287	0.1335
GO:0008009	XLOC_023720;XLOC_024161	Molecular Function	Chemokine activity	8	2	287	0.1578
GO:0004550	XLOC_010523	Molecular Function	Nucleoside diphosphate kinase activity	8	1	287	0.1683
GO:0034450	XLOC_023609	Molecular Function	Ubiquitin-ubiquitin ligase activity	8	1	287	0.1683
GO:0042974	XLOC_009333	Molecular Function	Retinoic acid receptor binding	8	1	287	0.2017
GO:0004623	XLOC_023480	Molecular Function	Phospholipase A2 activity	8	1	287	0.2179

GO:0004713	XLOC_002973;XLOC_003107;XLOC_017920;XLOC_023465;XLOC_023491;XLOC_023651;XLOC_023653;XLOC_024081;XLOC_024183;XLOC_024247;XLOC_024264;XLOC_025531	Molecular Function	Protein tyrosine kinase activity	8	12	287	0.2351
GO:0015171	XLOC_001311	Molecular Function	Amino acid transmembrane transporter activity	8	1	287	0.3083
GO:0030145	XLOC_023555	Molecular Function	Manganese ion binding	8	1	287	0.4915
GO:0004197	XLOC_023481	Molecular Function	Cysteine-type endopeptidase activity	8	1	287	0.5119
GO:0004721	XLOC_023512;XLOC_023553	Molecular Function	Phosphoprotein phosphatase activity	8	2	287	0.5710
GO:0005507	XLOC_023564	Molecular Function	Copper ion binding	8	1	287	0.5858
GO:0004674	XLOC_002973;XLOC_023465;XLOC_023907;XLOC_024183;XLOC_024247;XLOC_024264;XLOC_025531	Molecular Function	Protein serine/threonine kinase activity	8	7	287	0.6090
GO:0003743	XLOC_023531	Molecular Function	Translation initiation factor activity	8	1	287	0.6106
GO:0005216	XLOC_023978;XLOC_024806;XLOC_026899	Molecular Function	Ion channel activity	8	3	287	0.6574
GO:0017111	XLOC_003080;XLOC_023994	Molecular Function	Nucleoside-triphosphatase activity	8	2	287	0.7763
GO:0008270	XLOC_003103;XLOC_003207;XLOC_003674;XLOC_009917;XLOC_018228;XLOC_020726;XLOC_022857;XLOC_023448;XLOC_023494;XLOC_023529;XLOC_023564;XLOC_023599;XLOC_023618;XLOC_023917;XLOC_023962;XLOC_024061;XLOC_024075;XLOC_024155;XLOC_024235;XLOC_024254;XLOC_024284	Molecular Function	Zinc ion binding	8	21	287	0.9784
GO:0005097	XLOC_009333;XLOC_023491;XLOC_023598;XLOC_023863	Molecular Function	Rab GTPase activator activity	7	4	287	0.0171

GO:0030515	XLOC_023428;XLOC_023915	Molecular Function	snoRNA binding	7	2	287	0.0236
GO:0005161	XLOC_023297;XLOC_023651	Molecular Function	Platelet-derived growth factor receptor binding	7	2	287	0.0317
GO:0003953	XLOC_023853	Molecular Function	NAD+ nucleosidase activity	7	1	287	0.0401
GO:0004031	XLOC_011982	Molecular Function	Aldehyde oxidase activity	7	1	287	0.0401
GO:0004155	XLOC_024302	Molecular Function	6,7-dihydropteridine reductase activity	7	1	287	0.0401
GO:0004729	XLOC_020095	Molecular Function	Oxygen-dependent protoporphyrinogen oxidase activity	7	1	287	0.0401
GO:0008973	XLOC_023596	Molecular Function	Phosphopentomutase activity	7	1	287	0.0401
GO:0016992	XLOC_023606	Molecular Function	Lipoate synthase activity	7	1	287	0.0401
GO:0019780	XLOC_024105	Molecular Function	FAT10 activating enzyme activity	7	1	287	0.0401
GO:0051903	XLOC_023529	Molecular Function	S-(hydroxymethyl)glutathione dehydrogenase activity	7	1	287	0.0401
GO:0004519	XLOC_001362;XLOC_020726;XLOC_023611	Molecular Function	Endonuclease activity	7	3	287	0.0593
GO:0003858	XLOC_023501	Molecular Function	3-hydroxybutyrate dehydrogenase activity	7	1	287	0.0596
GO:0003998	XLOC_002658	Molecular Function	Acylphosphatase activity	7	1	287	0.0596
GO:0004022	XLOC_023529	Molecular Function	Alcohol dehydrogenase (NAD) activity	7	1	287	0.0596
GO:0004342	XLOC_024053	Molecular Function	Glucosamine-6-phosphate deaminase activity	7	1	287	0.0596
GO:0017172	XLOC_001806	Molecular Function	Cysteine dioxygenase activity	7	1	287	0.0596
GO:0047800	XLOC_001806	Molecular Function	Cysteamine dioxygenase activity	7	1	287	0.0596
GO:0004062	XLOC_024127	Molecular Function	Aryl sulfotransferase activity	7	1	287	0.0786
GO:0047493	XLOC_023922	Molecular Function	Ceramide cholinephosphotransferase activity	7	1	287	0.0786
GO:0000049	XLOC_023923;XLOC_023999	Molecular Function	tRNA binding	7	2	287	0.0843
GO:0004047	XLOC_003697	Molecular Function	Aminomethyltransferase activity	7	1	287	0.0973
GO:0004614	XLOC_023596	Molecular Function	Phosphoglucomutase activity	7	1	287	0.0973
GO:0005134	XLOC_020267	Molecular Function	Interleukin-2 receptor binding	7	1	287	0.0973
GO:0004536	XLOC_010523	Molecular Function	Deoxyribonuclease activity	7	1	287	0.1156
GO:0070579	XLOC_023933	Molecular Function	Methylcytosine dioxygenase activity	7	1	287	0.1156
GO:0030374	XLOC_023621;XLOC_023992	Molecular Function	Ligand-dependent nuclear receptor transcription coactivator activity	7	2	287	0.1230
GO:0003857	XLOC_023920	Molecular Function	3-hydroxyacyl-CoA dehydrogenase activity	7	1	287	0.1335
GO:0008121	XLOC_025657	Molecular Function	Ubiquinol-cytochrome-c reductase activity	7	1	287	0.1335
GO:0008312	XLOC_023666	Molecular Function	7S RNA binding	7	1	287	0.1335

GO:0005509	XLOC_002589;XLOC_003272;XLOC_005548;XLOC_012279;XLOC_015126;XLOC_017018;XLOC_023432;XLOC_023480;XLOC_023558;XLOC_023756;XLOC_023970;XLOC_023978;XLOC_024220;XLOC_024278	Molecular Function	Calcium ion binding	7	14	287	0.1500
GO:0004616	XLOC_024273	Molecular Function	Phosphogluconate dehydrogenase (decarboxylating) activity	7	1	287	0.1511
GO:0000993	XLOC_023835	Molecular Function	RNA polymerase II core binding	7	1	287	0.1852
GO:0008762	XLOC_011982	Molecular Function	UDP-N-acetylmuramate dehydrogenase activity	7	1	287	0.2017
GO:0000287	XLOC_010523;XLOC_017018;XLOC_023495;XLOC_023596;XLOC_024425	Molecular Function	Magnesium ion binding	7	5	287	0.2137
GO:0070403	XLOC_023920	Molecular Function	NAD+ binding	7	1	287	0.2179
GO:0001103	XLOC_023581	Molecular Function	RNA polymerase II repressing transcription factor binding	7	1	287	0.2645
GO:0001106	XLOC_024273	Molecular Function	RNA polymerase II transcription corepressor activity	7	1	287	0.2645
GO:0004143	XLOC_024265	Molecular Function	Diacylglycerol kinase activity	7	1	287	0.2645
GO:0004672	XLOC_002973;XLOC_003107;XLOC_017920;XLOC_023465;XLOC_023491;XLOC_023651;XLOC_023653;XLOC_023758;XLOC_024081;XLOC_024183;XLOC_024247;XLOC_024264;XLOC_025531	Molecular Function	Protein kinase activity	7	13	287	0.2702
GO:0005545	XLOC_024206	Molecular Function	1-phosphatidylinositol binding	7	1	287	0.2940
GO:0031593	XLOC_024203	Molecular Function	Polyubiquitin binding	7	1	287	0.3224
GO:0008324	XLOC_023621	Molecular Function	Cation transmembrane transporter activity	7	1	287	0.3361
GO:0048487	XLOC_024239	Molecular Function	Beta-tubulin binding	7	1	287	0.3496
GO:0004221	XLOC_023888;XLOC_024073	Molecular Function	Ubiquitin thiolesterase activity	7	2	287	0.3513
GO:0019843	XLOC_002615	Molecular Function	rRNA binding	7	1	287	0.3884
GO:0015293	XLOC_003157	Molecular Function	Symporter activity	7	1	287	0.4592

GO:0001077	XLOC_023581	Molecular Function	RNA polymerase II core promoter proximal region sequence-specific DNA binding transcription factor activity involved in positive regulation of transcription	7	1	287	0.5316
GO:0008234	XLOC_023481;XLOC_024073	Molecular Function	Cysteine-type peptidase activity	7	2	287	0.5710
GO:0008081	XLOC_023444	Molecular Function	Phosphoric diester hydrolase activity	7	1	287	0.6263
GO:0019903	XLOC_023568	Molecular Function	Protein phosphatase binding	7	1	287	0.6557
GO:0004867	XLOC_002593	Molecular Function	Serine-type endopeptidase inhibitor activity	7	1	287	0.6627
GO:0016791	XLOC_012858;XLOC_017018	Molecular Function	Phosphatase activity	7	2	287	0.6939
GO:0008017	XLOC_024802	Molecular Function	Microtubule binding	7	1	287	0.8324
GO:0031625	XLOC_023609	Molecular Function	Ubiquitin protein ligase binding	7	1	287	0.9281
GO:0043565	XLOC_003674;XLOC_015255;XLOC_023581;XLOC_023775;XLOC_023941	Molecular Function	Sequence-specific DNA binding	7	5	287	0.9437
GO:0019901	XLOC_023863	Molecular Function	Protein kinase binding	7	1	287	0.9934
GO:0048407	XLOC_003749;XLOC_010535;XLOC_023297;XLOC_023651	Molecular Function	Platelet-derived growth factor binding	6	4	287	0.0002
GO:0005072	XLOC_002956;XLOC_003605	Molecular Function	Transforming growth factor beta receptor, cytoplasmic mediator activity	6	2	287	0.0039
GO:0042805	XLOC_023978;XLOC_024296	Molecular Function	Actinin binding	6	2	287	0.0039
GO:0005534	XLOC_002956;XLOC_003605	Molecular Function	Galactose binding	6	2	287	0.0058
GO:0038085	XLOC_023651;XLOC_024081	Molecular Function	Vascular endothelial growth factor binding	6	2	287	0.0058
GO:0043394	XLOC_003749;XLOC_023558	Molecular Function	Proteoglycan binding	6	2	287	0.0058
GO:0051287	XLOC_011982;XLOC_023501;XLOC_023920;XLOC_024273	Molecular Function	NAD binding	6	4	287	0.0110

GO:0042803	XLOC_002956;XLOC_003605;XLOC_017018;XLOC_023297;XLOC_023439;XLOC_023529;XLOC_023558;XLOC_023651;XLOC_023653;XLOC_023703;XLOC_023923;XLOC_023941;XLOC_023978;XLOC_024155	Molecular Function	Protein homodimerization activity	6	14	287	0.0260
GO:0050431	XLOC_002956;XLOC_003605	Molecular Function	Transforming growth factor beta binding	6	2	287	0.0317
GO:0005499	XLOC_005548	Molecular Function	Vitamin D binding	6	1	287	0.0401
GO:0045505	XLOC_024239	Molecular Function	Dynein intermediate chain binding	6	1	287	0.0401
GO:0005544	XLOC_023432;XLOC_023756	Molecular Function	Calcium-dependent phospholipid binding	6	2	287	0.0455
GO:0016922	XLOC_023621;XLOC_023992	Molecular Function	Ligand-dependent nuclear receptor binding	6	2	287	0.0506
GO:0008201	XLOC_003749;XLOC_020890;XLOC_023435;XLOC_023558	Molecular Function	Heparin binding	6	4	287	0.0558
GO:0003883	XLOC_020698	Molecular Function	CTP synthase activity	6	1	287	0.0596
GO:0042498	XLOC_024021	Molecular Function	Diacyl lipopeptide binding	6	1	287	0.0596
GO:0004859	XLOC_023756	Molecular Function	Phospholipase inhibitor activity	6	1	287	0.0786
GO:0042296	XLOC_023974	Molecular Function	ISG15 transferase activity	6	1	287	0.0786
GO:0043398	XLOC_023978	Molecular Function	HLH domain binding	6	1	287	0.0786
GO:0005125	XLOC_023720;XLOC_023762;XLOC_023923;XLOC_024161	Molecular Function	Cytokine activity	6	4	287	0.0820
GO:0016301	XLOC_002973;XLOC_003107;XLOC_010523;XLOC_023465;XLOC_023651;XLOC_023653;XLOC_023703;XLOC_023758;XLOC_024081;XLOC_024183;XLOC_024247;XLOC_025531	Molecular Function	Kinase activity	6	12	287	0.0864
GO:0005520	XLOC_002593;XLOC_024099	Molecular Function	Insulin-like growth factor binding	6	2	287	0.0905
GO:0004518	XLOC_001362;XLOC_016324;XLOC_020726	Molecular Function	Nuclease activity	6	3	287	0.0921
GO:0030976	XLOC_024425	Molecular Function	Thiamine pyrophosphate binding	6	1	287	0.0973
GO:0043237	XLOC_023558	Molecular Function	Laminin-1 binding	6	1	287	0.0973

GO:0048495	XLOC_023558	Molecular Function	Roundabout binding	6	1	287	0.0973
GO:0016616	XLOC_023603;XLOC_023920;XLOC_024273	Molecular Function	Oxidoreductase activity, acting on the CH-OH group of donors, NAD or NADP as acceptor	6	3	287	0.1003
GO:0004659	XLOC_024196	Molecular Function	Prenyltransferase activity	6	1	287	0.1156
GO:0034452	XLOC_024239	Molecular Function	Dynactin binding	6	1	287	0.1156
GO:0043546	XLOC_011982	Molecular Function	Molybdopterin cofactor binding	6	1	287	0.1156
GO:0004784	XLOC_023564	Molecular Function	Superoxide dismutase activity	6	1	287	0.1335
GO:0008641	XLOC_024105	Molecular Function	Small protein activating enzyme activity	6	1	287	0.1335
GO:0004553	XLOC_023509;XLOC_023830	Molecular Function	Hydrolase activity, hydrolyzing O-glycosyl compounds	6	2	287	0.1436
GO:0019900	XLOC_023702;XLOC_024265	Molecular Function	Kinase binding	6	2	287	0.1507
GO:0000982	XLOC_023581	Molecular Function	RNA polymerase II core promoter proximal region sequence-specific DNA binding transcription factor activity	6	1	287	0.1511
GO:0001228	XLOC_023581	Molecular Function	RNA polymerase II transcription regulatory region sequence-specific DNA binding transcription factor activity involved in positive regulation of transcription	6	1	287	0.1511
GO:0016868	XLOC_023596	Molecular Function	Intramolecular transferase activity, phosphotransferases	6	1	287	0.1511
GO:0015267	XLOC_023978	Molecular Function	Channel activity	6	1	287	0.1683
GO:0016628	XLOC_023501	Molecular Function	Oxidoreductase activity, acting on the CH-CH group of donors, NAD or NADP as acceptor	6	1	287	0.1683
GO:0016717	XLOC_024190	Molecular Function	Oxidoreductase activity, acting on paired donors, with oxidation of a pair of donors resulting in the reduction of molecular oxygen to two molecules of water	6	1	287	0.1683
GO:0043274	XLOC_024265	Molecular Function	Phospholipase binding	6	1	287	0.1852
GO:0016881	XLOC_023609;XLOC_023974;XLOC_023975	Molecular Function	Amino acid ligase activity	6	3	287	0.2049
GO:0016712	XLOC_023921	Molecular Function	Oxidoreductase activity, acting on paired donors, with incorporation or reduction of molecular oxygen, reduced flavin or flavoprotein as one donor, and incorporation of one atom of oxygen	6	1	287	0.2179
GO:0048185	XLOC_013257	Molecular Function	Activin binding	6	1	287	0.2179
GO:0008083	XLOC_023297;XLOC_023720;XLOC_023762	Molecular Function	Growth factor activity	6	3	287	0.2207
GO:0005178	XLOC_020890;XLOC_024081	Molecular Function	Integrin binding	6	2	287	0.2314

GO:0051117	XLOC_023978	Molecular Function	ATPase binding	6	1	287	0.2337
GO:0004866	XLOC_025523	Molecular Function	Endopeptidase inhibitor activity	6	1	287	0.2645
GO:0051879	XLOC_002509	Molecular Function	Hsp90 protein binding	6	1	287	0.2645
GO:0004842	XLOC_003625;XLOC_022857;XLOC_023609;XLOC_023974;XLOC_024075;XLOC_024155	Molecular Function	Ubiquitin-protein transferase activity	6	6	287	0.2707
GO:0008603	XLOC_024802	Molecular Function	cAMP-dependent protein kinase regulator activity	6	1	287	0.2794
GO:0033613	XLOC_024265	Molecular Function	Activating transcription factor binding	6	1	287	0.2794
GO:0051537	XLOC_011982	Molecular Function	2 iron, 2 sulfur cluster binding	6	1	287	0.3083
GO:0009982	XLOC_023915	Molecular Function	Pseudouridine synthase activity	6	1	287	0.3224
GO:0046872	XLOC_008893;XLOC_009917;XLOC_011982;XLOC_012858;XLOC_017018;XLOC_018228;XLOC_020726;XLOC_021864;XLOC_022857;XLOC_023444;XLOC_023494;XLOC_023512;XLOC_023529;XLOC_023553;XLOC_023555;XLOC_023564;XLOC_023606;XLOC_023618;XLOC_023653;XLOC_023699;XLOC_023921;XLOC_023953;XLOC_023962;XLOC_024061;XLOC_024075;XLOC_024155;XLOC_024190;XLOC_024206;XLOC_024235;XLOC_024251;XLOC_024254;XLOC_024425;XLOC_025902	Molecular Function	Metal ion binding	6	33	287	0.3225
GO:0045296	XLOC_024296	Molecular Function	Cadherin binding	6	1	287	0.3361
GO:0051539	XLOC_023606	Molecular Function	4 iron, 4 sulfur cluster binding	6	1	287	0.3757
GO:0050660	XLOC_011982;XLOC_023920	Molecular Function	Flavin adenine dinucleotide binding	6	2	287	0.3878
GO:0004601	XLOC_023999	Molecular Function	Peroxidase activity	6	1	287	0.3884
GO:0015631	XLOC_024802	Molecular Function	Tubulin binding	6	1	287	0.4008
GO:0070491	XLOC_024273	Molecular Function	Repressing transcription factor binding	6	1	287	0.4130
GO:0005518	XLOC_023297	Molecular Function	Collagen binding	6	1	287	0.4249
GO:0008146	XLOC_024127	Molecular Function	Sulfotransferase activity	6	1	287	0.4702

GO:0019902	XLOC_003747	Molecular Function	Phosphatase binding	6	1	287	0.4915
GO:0016747	XLOC_023529	Molecular Function	Transferase activity, transferring acyl groups other than amino-acyl groups	6	1	287	0.5411
GO:0016773	XLOC_023566;XLOC_023703	Molecular Function	Phosphotransferase activity, alcohol group as acceptor	6	2	287	0.5593
GO:0003723	XLOC_002497;XLOC_002615;XLOC_003268;XLOC_023428;XLOC_023531;XLOC_023903;XLOC_023992;XLOC_024148	Molecular Function	RNA binding	6	8	287	0.5850
GO:0002020	XLOC_023653	Molecular Function	Protease binding	6	1	287	0.6025
GO:0030165	XLOC_024075	Molecular Function	PDZ domain binding	6	1	287	0.6106
GO:0008168	XLOC_023522;XLOC_023892	Molecular Function	Methyltransferase activity	6	2	287	0.6757
GO:0030170	XLOC_023999	Molecular Function	Pyridoxal phosphate binding	6	1	287	0.6763
GO:0035091	XLOC_002847	Molecular Function	Phosphatidylinositol binding	6	1	287	0.7365
GO:0046982	XLOC_023297;XLOC_024021	Molecular Function	Protein heterodimerization activity	6	2	287	0.8752
GO:0020037	XLOC_023921	Molecular Function	Heme binding	6	1	287	0.8820
GO:0003713	XLOC_023992	Molecular Function	Transcription coactivator activity	6	1	287	0.9059
GO:0003779	XLOC_023618;XLOC_023834	Molecular Function	Actin binding	6	2	287	0.9144
GO:0004930	XLOC_023990;XLOC_028154	Molecular Function	G-protein coupled receptor activity	6	2	287	0.9144
GO:0003677	XLOC_002509;XLOC_012452;XLOC_015255;XLOC_015261;XLOC_016324;XLOC_023518;XLOC_023581;XLOC_023822;XLOC_023941;XLOC_023963;XLOC_023971;XLOC_023988;XLOC_023992;XLOC_024024;XLOC_024289	Molecular Function	DNA binding	6	15	287	0.9731
GO:0005539	XLOC_002956;XLOC_003605;XLOC_023435	Molecular Function	Glycosaminoglycan binding	5	3	287	0.0054
GO:0097110	XLOC_011437;XLOC_022857	Molecular Function	Scaffold protein binding	5	2	287	0.0317

GO:0008134	XLOC_009333;XLOC_012264;XLOC_023581;XLOC_023992;XLOC_024038;XLOC_024239;XLOC_024254;XLOC_024273	Molecular Function	Transcription factor binding	5	8	287	0.0331
GO:0016229	XLOC_024219	Molecular Function	Steroid dehydrogenase activity	5	1	287	0.0401
GO:0016785	XLOC_023999	Molecular Function	Transferase activity, transferring selenium-containing groups	5	1	287	0.0401
GO:0050809	XLOC_024239	Molecular Function	Diazepam binding	5	1	287	0.0401
GO:0071723	XLOC_024021	Molecular Function	Lipopeptide binding	5	1	287	0.0401
GO:0016176	XLOC_023297	Molecular Function	Superoxide-generating NADPH oxidase activator activity	5	1	287	0.0596
GO:0002039	XLOC_022857;XLOC_024155;XLOC_024239	Molecular Function	p53 binding	5	3	287	0.0626
GO:0050662	XLOC_002857;XLOC_023603;XLOC_023920	Molecular Function	Coenzyme binding	5	3	287	0.0660
GO:0016849	XLOC_008893;XLOC_023853	Molecular Function	Phosphorus-oxygen lyase activity	5	2	287	0.0667
GO:0005047	XLOC_023666	Molecular Function	Signal recognition particle binding	5	1	287	0.0786

GO:0000166	XLOC_002615;XLOC_002857;XLOC_002973;XLOC_003080;XLOC_003107;XLOC_003207;XLOC_003322;XLOC_008893;XLOC_010523;XLOC_011326;XLOC_015530;XLOC_020095;XLOC_023444;XLOC_023465;XLOC_023501;XLOC_023529;XLOC_023609;XLOC_023621;XLOC_023626;XLOC_023651;XLOC_023653;XLOC_023703;XLOC_023745;XLOC_023853;XLOC_023903;XLOC_023920;XLOC_023963;XLOC_023992;XLOC_023994;XLOC_024037;XLOC_024081;XLOC_024105;XLOC_024141;XLOC_024157;XLOC_024183;XLOC_024219;XLOC_024247;XLOC_024273;XLOC_024287;XLOC_024302;XLOC_025531	Molecular Function	Nucleotide binding	5	41	287	0.0878
GO:0005095	XLOC_023558	Molecular Function	GTPase inhibitor activity	5	1	287	0.0973
GO:0005504	XLOC_023529	Molecular Function	Fatty acid binding	5	1	287	0.0973
GO:0005096	XLOC_002772;XLOC_016622;XLOC_023598;XLOC_023863	Molecular Function	GTPase activator activity	5	4	287	0.1066
GO:0016798	XLOC_023509;XLOC_023853	Molecular Function	Hydrolase activity, acting on glycosyl bonds	5	2	287	0.1230
GO:0015026	XLOC_021577	Molecular Function	Coreceptor activity	5	1	287	0.1335
GO:0019209	XLOC_023702	Molecular Function	Kinase activator activity	5	1	287	0.1335
GO:0043169	XLOC_023509;XLOC_023830	Molecular Function	Cation binding	5	2	287	0.1578
GO:0008565	XLOC_021577;XLOC_023730;XLOC_024147	Molecular Function	Protein transporter activity	5	3	287	0.1592
GO:0004016	XLOC_008893	Molecular Function	Adenylate cyclase activity	5	1	287	0.1683
GO:0043024	XLOC_010523	Molecular Function	Ribosomal small subunit binding	5	1	287	0.1852
GO:0019955	XLOC_023653	Molecular Function	Cytokine binding	5	1	287	0.2017

GO:0016772	XLOC_002973;XLOC_003107;XLOC_017920;XLOC_023465;XLOC_023491;XLOC_023651;XLOC_023653;XLOC_023758;XLOC_023907;XLOC_024081;XLOC_024183;XLOC_024247;XLOC_024264;XLOC_025531	Molecular Function	Transferase activity, transferring phosphorus-containing groups	5	14	287	0.2057
GO:0051536	XLOC_011982;XLOC_023606	Molecular Function	Iron-sulfur cluster binding	5	2	287	0.2314
GO:0031490	XLOC_023992	Molecular Function	Chromatin DNA binding	5	1	287	0.2337
GO:0004888	XLOC_023990;XLOC_024021;XLOC_028154	Molecular Function	Transmembrane signaling receptor activity	5	3	287	0.2750
GO:0016614	XLOC_011982	Molecular Function	Oxidoreductase activity, acting on CH-OH group of donors	5	1	287	0.2794
GO:0005484	XLOC_023801	Molecular Function	SNAP receptor activity	5	1	287	0.3496
GO:0031072	XLOC_023519;XLOC_024264	Molecular Function	Heat shock protein binding	5	2	287	0.3513
GO:0048306	XLOC_024191	Molecular Function	Calcium-dependent protein binding	5	1	287	0.3757
GO:0005516	XLOC_023465;XLOC_023512	Molecular Function	Calmodulin binding	5	2	287	0.3950
GO:0046983	XLOC_002509;XLOC_012264;XLOC_023512;XLOC_023822	Molecular Function	Protein dimerization activity	5	4	287	0.4122
GO:0019838	XLOC_024081	Molecular Function	Growth factor binding	5	1	287	0.4249
GO:0016788	XLOC_003268	Molecular Function	Hydrolase activity, acting on ester bonds	5	1	287	0.5018
GO:0044325	XLOC_023978	Molecular Function	Ion channel binding	5	1	287	0.5595
GO:0019904	XLOC_012264;XLOC_024273	Molecular Function	Protein domain specific binding	5	2	287	0.5993
GO:0008092	XLOC_023978	Molecular Function	Cytoskeletal protein binding	5	1	287	0.6263
GO:0008233	XLOC_021864;XLOC_023481;XLOC_023555;XLOC_023953;XLOC_024073	Molecular Function	Peptidase activity	5	5	287	0.6457
GO:0019899	XLOC_022857;XLOC_023512;XLOC_024165	Molecular Function	Enzyme binding	5	3	287	0.6732
GO:0004497	XLOC_023921	Molecular Function	Monooxygenase activity	5	1	287	0.6829
GO:0016705	XLOC_023921	Molecular Function	Oxidoreductase activity, acting on paired donors, with incorporation or reduction of molecular oxygen	5	1	287	0.6957
GO:0047485	XLOC_023581	Molecular Function	Protein N-terminus binding	5	1	287	0.6957

GO:0042802	XLOC_003322;XLOC_010523;XLOC_010535;XLOC_022857;XLOC_023978	Molecular Function	Identical protein binding	5	5	287	0.7062
GO:0005102	XLOC_003749;XLOC_010535;XLOC_023978	Molecular Function	Receptor binding	5	3	287	0.7137
GO:0051082	XLOC_023519	Molecular Function	Unfolded protein binding	5	1	287	0.8104
GO:0003676	XLOC_001362;XLOC_003103;XLOC_003207;XLOC_009917;XLOC_020726;XLOC_023599;XLOC_023775;XLOC_023903;XLOC_023963;XLOC_023971;XLOC_023992;XLOC_023994;XLOC_024037;XLOC_024141;XLOC_024157;XLOC_024235;XLOC_024284	Molecular Function	Nucleic acid binding	5	17	287	0.8245
GO:0008022	XLOC_012279	Molecular Function	Protein C-terminus binding	5	1	287	0.8488
GO:0005543	XLOC_002772;XLOC_016622;XLOC_024311	Molecular Function	Phospholipid binding	5	3	287	0.9474
GO:0005201	XLOC_003749;XLOC_010535;XLOC_017359	Molecular Function	Extracellular matrix structural constituent	4	3	287	0.0204
GO:0004857	XLOC_001060;XLOC_002593	Molecular Function	Enzyme inhibitor activity	4	2	287	0.0506
GO:0004803	XLOC_023775	Molecular Function	Transposase activity	4	1	287	0.0786
GO:0019208	XLOC_023758	Molecular Function	Phosphatase regulator activity	4	1	287	0.0786
GO:0019534	XLOC_001311	Molecular Function	Toxin transporter activity	4	1	287	0.0786
GO:0008144	XLOC_023512;XLOC_023703	Molecular Function	Drug binding	4	2	287	0.1163
GO:0005088	XLOC_024185	Molecular Function	Ras guanyl-nucleotide exchange factor activity	4	1	287	0.1683
GO:0005487	XLOC_003001	Molecular Function	Nucleocytoplasmic transporter activity	4	1	287	0.1683

GO:0016787	XLOC_001362;XLOC_002658;XLOC_012858;XLOC_017018;XLOC_020698;XLOC_020726;XLOC_021864;XLOC_023444;XLOC_023481;XLOC_023509;XLOC_023512;XLOC_023553;XLOC_023555;XLOC_023626;XLOC_023788;XLOC_023853;XLOC_023953;XLOC_023963;XLOC_023994;XLOC_024053;XLOC_024073;XLOC_025902	Molecular Function	Hydrolase activity	4	22	287	0.1683
GO:0016491	XLOC_002857;XLOC_003697;XLOC_011982;XLOC_020095;XLOC_023501;XLOC_023529;XLOC_023564;XLOC_023920;XLOC_023921;XLOC_024190;XLOC_024219;XLOC_024302	Molecular Function	Oxidoreductase activity	4	12	287	0.1778
GO:0032947	XLOC_023520	Molecular Function	Protein complex scaffold	4	1	287	0.2794
GO:0016740	XLOC_002973;XLOC_003107;XLOC_010523;XLOC_023465;XLOC_023522;XLOC_023606;XLOC_023651;XLOC_023653;XLOC_023703;XLOC_023999;XLOC_024081;XLOC_024127;XLOC_024183;XLOC_024247;XLOC_024425;XLOC_025531	Molecular Function	Transferase activity	4	16	287	0.3127
GO:0048037	XLOC_024273	Molecular Function	Cofactor binding	4	1	287	0.3628
GO:0003735	XLOC_002615;XLOC_003316;XLOC_003706;XLOC_028013	Molecular Function	Structural constituent of ribosome	4	4	287	0.4662
GO:0022857	XLOC_023813	Molecular Function	Transmembrane transporter activity	4	1	287	0.5218

GO:0005515	XLOC_000596;XLOC_001311;XLOC_001362;XLOC_001463;XLOC_002509;XLOC_002589;XLOC_002593;XLOC_002615;XLOC_002772;XLOC_002847;XLOC_002935;XLOC_002973;XLOC_003001;XLOC_003107;XLOC_003180;XLOC_003206;XLOC_003322;XLOC_003350;XLOC_003625;XLOC_003747;XLOC_003749;XLOC_005548;XLOC_009270;XLOC_009333;XLOC_010523;XLOC_010535;XLOC_011222;XLOC_011437;XLOC_012182;XLOC_012264;XLOC_012279;XLOC_012452;XLOC_013257;XLOC_013557;XLOC_013602;XLOC_015126;XLOC_015261;XLOC_015335;XLOC_015580;XLOC_016622;XLOC_018228;XLOC_018924;XLOC_020	Molecular Function	Protein binding	4	147	287	0.5735
GO:0003682	XLOC_023581;XLOC_023621;XLOC_024194;XLOC_024279	Molecular Function	Chromatin binding	4	4	287	0.6224
GO:0016874	XLOC_011326;XLOC_023609	Molecular Function	Ligase activity	4	2	287	0.7359
GO:0016829	XLOC_008893	Molecular Function	Lyase activity	4	1	287	0.7671
GO:0003700	XLOC_002509;XLOC_003350;XLOC_003674;XLOC_012264;XLOC_023581;XLOC_023941;XLOC_024061;XLOC_024273	Molecular Function	Sequence-specific DNA binding transcription factor activity	4	8	287	0.9108
GO:0004871	XLOC_002509;XLOC_011222;XLOC_013602;XLOC_023990	Molecular Function	Signal transducer activity	4	4	287	0.9254
GO:0045499	XLOC_023558	Molecular Function	Chemorepellent activity	3	1	287	0.1335
GO:0000988	XLOC_011222	Molecular Function	Protein binding transcription factor activity	3	1	287	0.2337

GO:0003824	XLOC_002857;XLOC_011326;XLOC_011982;XLOC_012858;XLOC_017018;XLOC_023444;XLOC_023509;XLOC_023553;XLOC_023606;XLOC_023825;XLOC_023830;XLOC_023922;XLOC_023999;XLOC_024088;XLOC_024105;XLOC_024425	Molecular Function	Catalytic activity	3	16	287	0.2347
GO:0009055	XLOC_011982;XLOC_023921;XLOC_025657	Molecular Function	Electron carrier activity	3	3	287	0.3027
GO:0042056	XLOC_023297	Molecular Function	Chemoattractant activity	3	1	287	0.3083
GO:0005085	XLOC_023520;XLOC_024185	Molecular Function	Guanyl-nucleotide exchange factor activity	3	2	287	0.5652
GO:0004872	XLOC_003107;XLOC_018924;XLOC_021577;XLOC_023651;XLOC_023653;XLOC_023990;XLOC_023992;XLOC_024021;XLOC_024081;XLOC_024165;XLOC_024180	Molecular Function	Receptor activity	3	11	287	0.5806
GO:0005488	XLOC_003625;XLOC_023730;XLOC_023956;XLOC_024034;XLOC_024160;XLOC_024206;XLOC_024239	Molecular Function	Binding	3	7	287	0.6290
GO:0005198	XLOC_011437;XLOC_024251	Molecular Function	Structural molecule activity	3	2	287	0.6662
GO:0005215	XLOC_003464;XLOC_023813	Molecular Function	Transporter activity	3	2	287	0.8227
GO:0003674	XLOC_002615	Molecular Function	Molecular_function	2	1	287	0.4480

S gene number indicated the number of significantly up-regulated transcripts annotated to one certain GO term by GO analysis; TS gene number indicated the total number of significantly up-regulated transcripts annotated by GO analysis.

Table S6 GO categories assigned to the differentially down-regulated transcripts in mammary glands between RS- and AH-fed dairy cows

GO id	Gene list	GO term	GO description	GO class	S gene number	TS gene number	P-value of Fisher's Exact Test
GO:0072333	XLOC_001118	Biological Process	Anoikis by p53 class mediator	1	1	82	0.0117
GO:2001220	XLOC_005665	Biological Process	Negative regulation of G2 phase of mitotic cell cycle	1	1	82	0.0117
GO:0008633	XLOC_005665	Biological Process	Activation of pro-apoptotic gene products	1	1	82	0.0290
GO:0008634	XLOC_005665	Biological Process	Negative regulation of survival gene product expression	1	1	82	0.0460
GO:0045768	XLOC_020881	Biological Process	Positive regulation of anti-apoptosis	1	1	82	0.0792
GO:0045630	XLOC_020881	Biological Process	Positive regulation of T-helper 2 cell differentiation	15	1	82	0.0290
GO:0061044	XLOC_017082	Biological Process	Negative regulation of vascular wound healing	14	1	82	0.0117
GO:0070244	XLOC_005156	Biological Process	Negative regulation of thymocyte apoptotic process	14	1	82	0.0175
GO:0032792	XLOC_001118	Biological Process	Negative regulation of CREB transcription factor activity	14	1	82	0.0233
GO:0006919	XLOC_005665; XLOC_019618	Biological Process	Activation of cysteine-type endopeptidase activity involved in apoptotic process	14	2	82	0.0442
GO:0051092	XLOC_007556	Biological Process	Positive regulation of NF-kappaB transcription factor activity	14	1	82	0.3503
GO:0007171	XLOC_024579	Biological Process	Activation of transmembrane receptor protein tyrosine kinase activity	13	1	82	0.0404

GO:0007250	XLOC_007525	Biological Process	Activation of NF-kappaB-inducing kinase activity	13	1	82	0.0572
GO:0043537	XLOC_024579	Biological Process	Negative regulation of blood vessel endothelial cell migration	13	1	82	0.0628
GO:0043536	XLOC_024579	Biological Process	Positive regulation of blood vessel endothelial cell migration	13	1	82	0.1007
GO:0000186	XLOC_026769	Biological Process	Activation of MAPKK activity	13	1	82	0.1268
GO:0043280	XLOC_005665	Biological Process	Positive regulation of cysteine-type endopeptidase activity involved in apoptotic process	13	1	82	0.1371
GO:0045944	XLOC_001733; XLOC_005665; XLOC_007447; XLOC_020881; XLOC_024894	Biological Process	Positive regulation of transcription from RNA polymerase II promoter	13	5	82	0.1975
GO:0030509	XLOC_024894	Biological Process	BMP signaling pathway	13	1	82	0.2055
GO:0051091	XLOC_020881	Biological Process	Positive regulation of sequence-specific DNA binding transcription factor activity	13	1	82	0.2422
GO:0043154	XLOC_012793	Biological Process	Negative regulation of cysteine-type endopeptidase activity involved in apoptotic process	13	1	82	0.2555
GO:0045766	XLOC_007525; XLOC_017082; XLOC_019618; XLOC_024579	Biological Process	Positive regulation of angiogenesis	12	4	82	0.0004
GO:0010641	XLOC_019618	Biological Process	Positive regulation of platelet-derived growth factor receptor signaling pathway	12	1	82	0.0117
GO:0010664	XLOC_017390	Biological Process	Negative regulation of striated muscle cell apoptotic process	12	1	82	0.0117
GO:0046061	XLOC_005156	Biological Process	dATP catabolic process	12	1	82	0.0117

GO:0060169	XLOC_005156	Biological Process	Negative regulation of adenosine receptor signaling pathway	12	1	82	0.0117
GO:0032873	XLOC_005665	Biological Process	Negative regulation of stress-activated MAPK cascade	12	1	82	0.0233
GO:0045893	XLOC_001733; XLOC_005665; XLOC_007447; XLOC_008837; XLOC_020881; XLOC_024894	Biological Process	Positive regulation of transcription, DNA-templated	12	6	82	0.0277
GO:0060261	XLOC_008837	Biological Process	Positive regulation of transcription initiation from RNA polymerase II promoter	12	1	82	0.0290
GO:0033089	XLOC_005156	Biological Process	Positive regulation of T cell differentiation in thymus	12	1	82	0.0347
GO:0046638	XLOC_005156	Biological Process	Positive regulation of alpha-beta T cell differentiation	12	1	82	0.0404
GO:0030913	XLOC_016188	Biological Process	Paranodal junction assembly	12	1	82	0.0460
GO:0042517	XLOC_020881	Biological Process	Positive regulation of tyrosine phosphorylation of Stat3 protein	12	1	82	0.0792
GO:0046627	XLOC_010800	Biological Process	Negative regulation of insulin receptor signaling pathway	12	1	82	0.1007
GO:0010595	XLOC_024579	Biological Process	Positive regulation of endothelial cell migration	12	1	82	0.1421
GO:0000079	XLOC_012793	Biological Process	Regulation of cyclin-dependent protein serine/threonine kinase activity	12	1	82	0.1865
GO:0070374	XLOC_007525	Biological Process	Positive regulation of ERK1 and ERK2 cascade	12	1	82	0.2815
GO:0006184	XLOC_005533; XLOC_007556	Biological Process	GTP catabolic process	12	2	82	0.4213

GO:0000122	XLOC_005665; XLOC_024894	Biological Process	Negative regulation of transcription from RNA polymerase II promoter	12	2	82	0.6610
GO:0045892	XLOC_007447	Biological Process	Negative regulation of transcription, DNA-templated	12	1	82	0.8652
GO:0016525	XLOC_011014; XLOC_024579; XLOC_027571	Biological Process	Negative regulation of angiogenesis	11	3	82	0.0023
GO:0006157	XLOC_005156	Biological Process	Deoxyadenosine catabolic process	11	1	82	0.0117
GO:0033233	XLOC_024894	Biological Process	Regulation of protein sumoylation	11	1	82	0.0117
GO:0033382	XLOC_018104	Biological Process	Maintenance of granzyme B location in T cell secretory granule	11	1	82	0.0117
GO:0034164	XLOC_007556	Biological Process	Negative regulation of toll-like receptor 9 signaling pathway	11	1	82	0.0117
GO:0046103	XLOC_005156	Biological Process	Inosine biosynthetic process	11	1	82	0.0117
GO:0046111	XLOC_005156	Biological Process	Xanthine biosynthetic process	11	1	82	0.0117
GO:0046320	XLOC_021372	Biological Process	Regulation of fatty acid oxidation	11	1	82	0.0117
GO:0010951	XLOC_014139; XLOC_017082	Biological Process	Negative regulation of endopeptidase activity	11	2	82	0.0125
GO:0006048	XLOC_024317	Biological Process	UDP-N-acetylglucosamine biosynthetic process	11	1	82	0.0175
GO:0006154	XLOC_005156	Biological Process	Adenosine catabolic process	11	1	82	0.0175

GO:0032288	XLOC_011269	Biological Process	Myelin assembly	11	1	82	0.0175
GO:0043217	XLOC_016188	Biological Process	Myelin maintenance	11	1	82	0.0175
GO:0045656	XLOC_005665	Biological Process	Negative regulation of monocyte differentiation	11	1	82	0.0175
GO:0046101	XLOC_005156	Biological Process	Hypoxanthine biosynthetic process	11	1	82	0.0175
GO:0034144	XLOC_007556	Biological Process	Negative regulation of toll-like receptor 4 signaling pathway	11	1	82	0.0233
GO:0038032	XLOC_007583; XLOC_011718	Biological Process	Termination of G-protein coupled receptor signaling pathway	11	2	82	0.0275
GO:0010757	XLOC_017082	Biological Process	Negative regulation of plasminogen activation	11	1	82	0.0290
GO:0010460	XLOC_005156	Biological Process	Positive regulation of heart rate	11	1	82	0.0347
GO:0060056	XLOC_007447	Biological Process	Mammary gland involution	11	1	82	0.0347
GO:1902042	XLOC_017082	Biological Process	Negative regulation of extrinsic apoptotic signaling pathway via death domain receptors	11	1	82	0.0347
GO:0009168	XLOC_005156	Biological Process	Purine ribonucleoside monophosphate biosynthetic process	11	1	82	0.0404
GO:0014912	XLOC_017082	Biological Process	Negative regulation of smooth muscle cell migration	11	1	82	0.0404
GO:0034351	XLOC_011014	Biological Process	Negative regulation of glial cell apoptotic process	11	1	82	0.0460

GO:0006882	XLOC_009651	Biological Process	Cellular zinc ion homeostasis	11	1	82	0.0517
GO:0045582	XLOC_005156	Biological Process	Positive regulation of T cell differentiation	11	1	82	0.0572
GO:0046580	XLOC_026730	Biological Process	Negative regulation of Ras protein signal transduction	11	1	82	0.0628
GO:0048741	XLOC_000002	Biological Process	Skeletal muscle fiber development	11	1	82	0.0683
GO:2000352	XLOC_017082	Biological Process	Negative regulation of endothelial cell apoptotic process	11	1	82	0.0738
GO:0045765	XLOC_017082	Biological Process	Regulation of angiogenesis	11	1	82	0.0792
GO:0050850	XLOC_005156	Biological Process	Positive regulation of calcium-mediated signaling	11	1	82	0.0846
GO:0031069	XLOC_007166	Biological Process	Hair follicle morphogenesis	11	1	82	0.1164
GO:0006879	XLOC_005665	Biological Process	Cellular iron ion homeostasis	11	1	82	0.1421
GO:0043410	XLOC_020881	Biological Process	Positive regulation of MAPK cascade	11	1	82	0.2555
GO:0006469	XLOC_026769	Biological Process	Negative regulation of protein kinase activity	11	1	82	0.2643
GO:0090090	XLOC_024894	Biological Process	Negative regulation of canonical Wnt signaling pathway	11	1	82	0.3268
GO:0043524	XLOC_011014	Biological Process	Negative regulation of neuron apoptotic process	11	1	82	0.3464

GO:0006366	XLOC_024894	Biological Process	Transcription from RNA polymerase II promoter	11	1	82	0.3729
GO:0006355	XLOC_001733; XLOC_005665; XLOC_007447; XLOC_007981; XLOC_008837; XLOC_009240; XLOC_024894	Biological Process	Regulation of transcription, DNA-templated	11	7	82	0.4481
GO:0006200	XLOC_003967	Biological Process	ATP catabolic process	11	1	82	0.4689
GO:0050862	XLOC_005156; XLOC_009913	Biological Process	Positive regulation of T cell receptor signaling pathway	10	2	82	0.0009
GO:0002314	XLOC_005156	Biological Process	Germinal center B cell differentiation	10	1	82	0.0117
GO:0006883	XLOC_007981	Biological Process	Cellular sodium ion homeostasis	10	1	82	0.0117
GO:0010868	XLOC_001118	Biological Process	Negative regulation of triglyceride biosynthetic process	10	1	82	0.0117
GO:0033373	XLOC_018104	Biological Process	Maintenance of protease location in mast cell secretory granule	10	1	82	0.0117
GO:0043000	XLOC_022742	Biological Process	Golgi to plasma membrane CFTR protein transport	10	1	82	0.0117
GO:0070091	XLOC_020881	Biological Process	Glucagon secretion	10	1	82	0.0117
GO:0032204	XLOC_005665	Biological Process	Regulation of telomere maintenance	10	1	82	0.0175
GO:0042304	XLOC_021372	Biological Process	Regulation of fatty acid biosynthetic process	10	1	82	0.0175

GO:2000210	XLOC_001118	Biological Process	Positive regulation of anoikis	10	1	82	0.0233
GO:0010510	XLOC_021372	Biological Process	Regulation of acetyl-CoA biosynthetic process from pyruvate	10	1	82	0.0290
GO:0051897	XLOC_007525; XLOC_019618	Biological Process	Positive regulation of protein kinase B signaling	10	2	82	0.0335
GO:0045580	XLOC_005156	Biological Process	Regulation of T cell differentiation	10	1	82	0.0347
GO:0046888	XLOC_020881	Biological Process	Negative regulation of hormone secretion	10	1	82	0.0347
GO:0060664	XLOC_020881	Biological Process	Epithelial cell proliferation involved in salivary gland morphogenesis	10	1	82	0.0347
GO:0010830	XLOC_001118	Biological Process	Regulation of myotube differentiation	10	1	82	0.0404
GO:0043666	XLOC_000002	Biological Process	Regulation of phosphoprotein phosphatase activity	10	1	82	0.0404
GO:0045654	XLOC_007556	Biological Process	Positive regulation of megakaryocyte differentiation	10	1	82	0.0404
GO:2000573	XLOC_005665	Biological Process	Positive regulation of DNA biosynthetic process	10	1	82	0.0404
GO:0050731	XLOC_020881; XLOC_024579	Biological Process	Positive regulation of peptidyl-tyrosine phosphorylation	10	2	82	0.0414
GO:0045987	XLOC_005156	Biological Process	Positive regulation of smooth muscle contraction	10	1	82	0.0517
GO:0046427	XLOC_020881	Biological Process	Positive regulation of JAK-STAT cascade	10	1	82	0.0572

GO:0010800	XLOC_007525	Biological Process	Positive regulation of peptidyl-threonine phosphorylation	10	1	82	0.0683
GO:0051085	XLOC_003872	Biological Process	Chaperone mediated protein folding requiring cofactor	10	1	82	0.0683
GO:0048663	XLOC_007166	Biological Process	Neuron fate commitment	10	1	82	0.0792
GO:2000811	XLOC_021372	Biological Process	Negative regulation of anoikis	10	1	82	0.0792
GO:0060395	XLOC_001733	Biological Process	SMAD protein signal transduction	10	1	82	0.0900
GO:0006309	XLOC_005665	Biological Process	Apoptotic DNA fragmentation	10	1	82	0.0954
GO:0006885	XLOC_021372	Biological Process	Regulation of pH	10	1	82	0.0954
GO:0001824	XLOC_007447	Biological Process	Blastocyst development	10	1	82	0.1112
GO:0048666	XLOC_007166	Biological Process	Neuron development	10	1	82	0.1320
GO:0010466	XLOC_017082	Biological Process	Negative regulation of peptidase activity	10	1	82	0.1472
GO:0001525	XLOC_017082; XLOC_024579	Biological Process	Angiogenesis	10	2	82	0.1506
GO:0008286	XLOC_021372	Biological Process	Insulin receptor signaling pathway	10	1	82	0.1769
GO:0051289	XLOC_026730	Biological Process	Protein homotetramerization	10	1	82	0.1817

GO:0001942	XLOC_007166	Biological Process	Hair follicle development	10	1	82	0.1865
GO:0046854	XLOC_026730	Biological Process	Phosphatidylinositol phosphorylation	10	1	82	0.2149
GO:0033138	XLOC_020881	Biological Process	Positive regulation of peptidyl-serine phosphorylation	10	1	82	0.2377
GO:0007179	XLOC_001733	Biological Process	Transforming growth factor beta receptor signaling pathway	10	1	82	0.2422
GO:0043123	XLOC_024907	Biological Process	Positive regulation of I-kappaB kinase/NF-kappaB signaling	10	1	82	0.4752
GO:0016567	XLOC_010800	Biological Process	Protein ubiquitination	10	1	82	0.7292
GO:0006351	XLOC_005665; XLOC_007981	Biological Process	Transcription, DNA-templated	10	2	82	0.8260
GO:0043066	XLOC_005156; XLOC_005665; XLOC_017082; XLOC_017390; XLOC_020881; XLOC_022742; XLOC_024579	Biological Process	Negative regulation of apoptotic process	9	7	82	0.0019
GO:0008360	XLOC_011014; XLOC_014159; XLOC_016188	Biological Process	Regulation of cell shape	9	3	82	0.0108
GO:0002636	XLOC_005156	Biological Process	Positive regulation of germinal center formation	9	1	82	0.0117
GO:0032261	XLOC_005156	Biological Process	Purine nucleotide salvage	9	1	82	0.0117
GO:0035491	XLOC_017082	Biological Process	Positive regulation of leukotriene production involved in inflammatory response	9	1	82	0.0117

GO:0060407	XLOC_005156	Biological Process	Negative regulation of penile erection	9	1	82	0.0117
GO:2000502	XLOC_011014	Biological Process	Negative regulation of natural killer cell chemotaxis	9	1	82	0.0117
GO:0002675	XLOC_020881	Biological Process	Positive regulation of acute inflammatory response	9	1	82	0.0175
GO:0045079	XLOC_020881	Biological Process	Negative regulation of chemokine biosynthetic process	9	1	82	0.0175
GO:2000427	XLOC_011014	Biological Process	Positive regulation of apoptotic cell clearance	9	1	82	0.0175
GO:0007263	XLOC_009651	Biological Process	Nitric oxide mediated signal transduction	9	1	82	0.0233
GO:0045332	XLOC_009913	Biological Process	Phospholipid translocation	9	1	82	0.0233
GO:0002906	XLOC_005156	Biological Process	Negative regulation of mature B cell apoptotic process	9	1	82	0.0290
GO:0043615	XLOC_011014	Biological Process	Astrocyte cell migration	9	1	82	0.0290
GO:0045820	XLOC_026730	Biological Process	Negative regulation of glycolytic process	9	1	82	0.0290
GO:0051024	XLOC_020881	Biological Process	Positive regulation of immunoglobulin secretion	9	1	82	0.0290
GO:0051918	XLOC_017082	Biological Process	Negative regulation of fibrinolysis	9	1	82	0.0290
GO:0018106	XLOC_021372	Biological Process	Peptidyl-histidine phosphorylation	9	1	82	0.0347

GO:0030212	XLOC_004817	Biological Process	Hyaluronan metabolic process	9	1	82	0.0347
GO:0046827	XLOC_012793	Biological Process	Positive regulation of protein export from nucleus	9	1	82	0.0404
GO:0000165	XLOC_005665; XLOC_011014	Biological Process	MAPK cascade	9	2	82	0.0442
GO:0050710	XLOC_018104	Biological Process	Negative regulation of cytokine secretion	9	1	82	0.0460
GO:0070328	XLOC_024579	Biological Process	Triglyceride homeostasis	9	1	82	0.0460
GO:0051770	XLOC_011014	Biological Process	Positive regulation of nitric-oxide synthase biosynthetic process	9	1	82	0.0517
GO:0070102	XLOC_020881	Biological Process	Interleukin-6-mediated signaling pathway	9	1	82	0.0572
GO:0006541	XLOC_024317	Biological Process	Glutamine metabolic process	9	1	82	0.0628
GO:0060389	XLOC_007166	Biological Process	Pathway-restricted SMAD protein phosphorylation	9	1	82	0.0683
GO:0001502	XLOC_007166	Biological Process	Cartilage condensation	9	1	82	0.0792
GO:0070498	XLOC_024894	Biological Process	Interleukin-1-mediated signaling pathway	9	1	82	0.0792
GO:0006094	XLOC_026730	Biological Process	Gluconeogenesis	9	1	82	0.0846
GO:0009749	XLOC_024894	Biological Process	Response to glucose	9	1	82	0.0954

GO:0045429	XLOC_000961	Biological Process	Positive regulation of nitric oxide biosynthetic process	9	1	82	0.1007
GO:0033209	XLOC_022742	Biological Process	Tumor necrosis factor-mediated signaling pathway	9	1	82	0.1060
GO:0008625	XLOC_017390	Biological Process	Extrinsic apoptotic signaling pathway via death domain receptors	9	1	82	0.1112
GO:0008630	XLOC_012793	Biological Process	Intrinsic apoptotic signaling pathway in response to DNA damage	9	1	82	0.1112
GO:0019722	XLOC_000002	Biological Process	Calcium-mediated signaling	9	1	82	0.1216
GO:0045727	XLOC_020881	Biological Process	Positive regulation of translation	9	1	82	0.1472
GO:0001932	XLOC_010800	Biological Process	Regulation of protein phosphorylation	9	1	82	0.1572
GO:0030217	XLOC_024894	Biological Process	T cell differentiation	9	1	82	0.1572
GO:0030890	XLOC_005156	Biological Process	Positive regulation of B cell proliferation	9	1	82	0.1572
GO:0048286	XLOC_005156	Biological Process	Lung alveolus development	9	1	82	0.1572
GO:0030216	XLOC_012793	Biological Process	Keratinocyte differentiation	9	1	82	0.1671
GO:0007218	XLOC_015548	Biological Process	Neuropeptide signaling pathway	9	1	82	0.2102
GO:0060070	XLOC_005665	Biological Process	Canonical Wnt signaling pathway	9	1	82	0.2772

GO:0030336	XLOC_017082	Biological Process	Negative regulation of cell migration	9	1	82	0.2899
GO:0006816	XLOC_009913	Biological Process	Calcium ion transport	9	1	82	0.3803
GO:0035335	XLOC_013166	Biological Process	Peptidyl-tyrosine dephosphorylation	9	1	82	0.4160
GO:0046777	XLOC_001118	Biological Process	Protein autophosphorylation	9	1	82	0.5170
GO:0001701	XLOC_005156	Biological Process	In utero embryonic development	9	1	82	0.6874
GO:0071294	XLOC_009648; XLOC_009651	Biological Process	Cellular response to zinc ion	8	2	82	0.0002
GO:0071222	XLOC_008837; XLOC_011014; XLOC_017082	Biological Process	Cellular response to lipopolysaccharide	8	3	82	0.0020
GO:0050829	XLOC_017082; XLOC_020881	Biological Process	Defense response to Gram-negative bacterium	8	2	82	0.0034
GO:0050870	XLOC_005156; XLOC_011014	Biological Process	Positive regulation of T cell activation	8	2	82	0.0049
GO:0071277	XLOC_001733; XLOC_009240	Biological Process	Cellular response to calcium ion	8	2	82	0.0080
GO:0002541	XLOC_019618	Biological Process	Activation of plasma proteins involved in acute inflammatory response	8	1	82	0.0117
GO:0071286	XLOC_026730	Biological Process	Cellular response to magnesium ion	8	1	82	0.0117
GO:0072606	XLOC_007525	Biological Process	Interleukin-8 secretion	8	1	82	0.0117

GO:0032755	XLOC_007556; XLOC_020881	Biological Process	Positive regulation of interleukin-6 production	8	2	82	0.0142
GO:0032966	XLOC_020881	Biological Process	Negative regulation of collagen biosynthetic process	8	1	82	0.0175
GO:0034499	XLOC_007556	Biological Process	Late endosome to Golgi transport	8	1	82	0.0175
GO:0070256	XLOC_005156	Biological Process	Negative regulation of mucus secretion	8	1	82	0.0175
GO:0090265	XLOC_011014	Biological Process	Positive regulation of immune complex clearance by monocytes and macrophages	8	1	82	0.0175
GO:2000098	XLOC_017082	Biological Process	Negative regulation of smooth muscle cell-matrix adhesion	8	1	82	0.0175
GO:0010572	XLOC_007669	Biological Process	Positive regulation of platelet activation	8	1	82	0.0233
GO:0045124	XLOC_021372	Biological Process	Regulation of bone resorption	8	1	82	0.0233
GO:0060708	XLOC_010800	Biological Process	Spongiotrophoblast differentiation	8	1	82	0.0233
GO:0006032	XLOC_007525	Biological Process	Chitin catabolic process	8	1	82	0.0290
GO:0008626	XLOC_018104	Biological Process	Granzyme-mediated apoptotic signaling pathway	8	1	82	0.0290
GO:0010469	XLOC_017082	Biological Process	Regulation of receptor activity	8	1	82	0.0290
GO:0043276	XLOC_001118	Biological Process	Anoikis	8	1	82	0.0290

GO:0045721	XLOC_001118	Biological Process	Negative regulation of gluconeogenesis	8	1	82	0.0290
GO:0051005	XLOC_024579	Biological Process	Negative regulation of lipoprotein lipase activity	8	1	82	0.0290
GO:0010759	XLOC_011014	Biological Process	Positive regulation of macrophage chemotaxis	8	1	82	0.0347
GO:0050871	XLOC_020881	Biological Process	Positive regulation of B cell activation	8	1	82	0.0347
GO:0042981	XLOC_005665; XLOC_007166; XLOC_020881	Biological Process	Regulation of apoptotic process	8	3	82	0.0387
GO:0048260	XLOC_017082	Biological Process	Positive regulation of receptor-mediated endocytosis	8	1	82	0.0404
GO:0071480	XLOC_024894	Biological Process	Cellular response to gamma radiation	8	1	82	0.0404
GO:0031018	XLOC_020881	Biological Process	Endocrine pancreas development	8	1	82	0.0460
GO:2001022	XLOC_005665	Biological Process	Positive regulation of response to DNA damage stimulus	8	1	82	0.0460
GO:0019221	XLOC_011014; XLOC_020881	Biological Process	Cytokine-mediated signaling pathway	8	2	82	0.0499
GO:0001878	XLOC_000961	Biological Process	Response to yeast	8	1	82	0.0517
GO:0030194	XLOC_017082	Biological Process	Positive regulation of blood coagulation	8	1	82	0.0517
GO:0030502	XLOC_018104	Biological Process	Negative regulation of bone mineralization	8	1	82	0.0517

GO:0050714	XLOC_009913	Biological Process	Positive regulation of protein secretion	8	1	82	0.0517
GO:0060670	XLOC_010800	Biological Process	Branching involved in labyrinthine layer morphogenesis	8	1	82	0.0517
GO:0060707	XLOC_010800	Biological Process	Trophoblast giant cell differentiation	8	1	82	0.0517
GO:0090026	XLOC_017082	Biological Process	Positive regulation of monocyte chemotaxis	8	1	82	0.0517
GO:0006953	XLOC_020881	Biological Process	Acute-phase response	8	1	82	0.0572
GO:0030324	XLOC_005156; XLOC_007525	Biological Process	Lung development	8	2	82	0.0653
GO:0032722	XLOC_020881	Biological Process	Positive regulation of chemokine production	8	1	82	0.0683
GO:0044344	XLOC_011014	Biological Process	Cellular response to fibroblast growth factor stimulus	8	1	82	0.0683
GO:0048566	XLOC_005156	Biological Process	Embryonic digestive tract development	8	1	82	0.0683
GO:0032735	XLOC_008837	Biological Process	Positive regulation of interleukin-12 production	8	1	82	0.0792
GO:0051384	XLOC_020881	Biological Process	Response to glucocorticoid	8	1	82	0.0792
GO:0000160	XLOC_021372	Biological Process	Phosphorelay signal transduction system	8	1	82	0.0846
GO:0044130	XLOC_008837	Biological Process	Negative regulation of growth of symbiont in host	8	1	82	0.0846

GO:0070301	XLOC_020881	Biological Process	Cellular response to hydrogen peroxide	8	1	82	0.0846
GO:0032757	XLOC_017082	Biological Process	Positive regulation of interleukin-8 production	8	1	82	0.0900
GO:0055007	XLOC_001118	Biological Process	Cardiac muscle cell differentiation	8	1	82	0.0954
GO:0009267	XLOC_021372	Biological Process	Cellular response to starvation	8	1	82	0.1060
GO:0050766	XLOC_000961	Biological Process	Positive regulation of phagocytosis	8	1	82	0.1060
GO:0071347	XLOC_011014	Biological Process	Cellular response to interleukin-1	8	1	82	0.1112
GO:0043491	XLOC_011014	Biological Process	Protein kinase B signaling	8	1	82	0.1164
GO:0045597	XLOC_010800	Biological Process	Positive regulation of cell differentiation	8	1	82	0.1164
GO:0023014	XLOC_021372	Biological Process	Signal transduction by phosphorylation	8	1	82	0.1216
GO:0060326	XLOC_021929	Biological Process	Cell chemotaxis	8	1	82	0.1216
GO:0009968	XLOC_010800	Biological Process	Negative regulation of signal transduction	8	1	82	0.1268
GO:0050729	XLOC_017082	Biological Process	Positive regulation of inflammatory response	8	1	82	0.1371
GO:0060348	XLOC_010383	Biological Process	Bone development	8	1	82	0.1371

GO:0071356	XLOC_011014	Biological Process	Cellular response to tumor necrosis factor	8	1	82	0.1371
GO:0031663	XLOC_011014	Biological Process	Lipopolysaccharide-mediated signaling pathway	8	1	82	0.1421
GO:0034644	XLOC_005665	Biological Process	Cellular response to UV	8	1	82	0.1421
GO:0042102	XLOC_020881	Biological Process	Positive regulation of T cell proliferation	8	1	82	0.1472
GO:0032729	XLOC_008837	Biological Process	Positive regulation of interferon-gamma production	8	1	82	0.1572
GO:0050830	XLOC_020881	Biological Process	Defense response to Gram-positive bacterium	8	1	82	0.1572
GO:0007264	XLOC_005533; XLOC_007556; XLOC_010661	Biological Process	Small GTPase mediated signal transduction	8	3	82	0.1643
GO:0006338	XLOC_005665	Biological Process	Chromatin remodeling	8	1	82	0.2102
GO:0043588	XLOC_012793	Biological Process	Skin development	8	1	82	0.2149
GO:0050728	XLOC_005156	Biological Process	Negative regulation of inflammatory response	8	1	82	0.2149
GO:0001889	XLOC_005156	Biological Process	Liver development	8	1	82	0.2332
GO:0042593	XLOC_021372	Biological Process	Glucose homeostasis	8	1	82	0.2422
GO:0043401	XLOC_007981	Biological Process	Steroid hormone mediated signaling pathway	8	1	82	0.2599

GO:0001568	XLOC_007166	Biological Process	Blood vessel development	8	1	82	0.2643
GO:0007229	XLOC_011852	Biological Process	Integrin-mediated signaling pathway	8	1	82	0.2729
GO:0007186	XLOC_011718; XLOC_015548; XLOC_021530	Biological Process	G-protein coupled receptor signaling pathway	8	3	82	0.2871
GO:0006486	XLOC_005319	Biological Process	Protein glycosylation	8	1	82	0.2983
GO:0006813	XLOC_009913	Biological Process	Potassium ion transport	8	1	82	0.3228
GO:0006913	XLOC_007556	Biological Process	Nucleocytoplasmic transport	8	1	82	0.4194
GO:0006468	XLOC_001118; XLOC_005319; XLOC_011157; XLOC_021372	Biological Process	Protein phosphorylation	8	4	82	0.4387
GO:0010628	XLOC_007166	Biological Process	Positive regulation of gene expression	8	1	82	0.5142
GO:0006470	XLOC_013166	Biological Process	Protein dephosphorylation	8	1	82	0.5340
GO:0007507	XLOC_007166	Biological Process	Heart development	8	1	82	0.5788
GO:0010574	XLOC_011014; XLOC_020881	Biological Process	Regulation of vascular endothelial growth factor production	7	2	82	0.0005
GO:0042832	XLOC_008837; XLOC_020881	Biological Process	Defense response to protozoan	7	2	82	0.0022
GO:0030162	XLOC_014139; XLOC_017082	Biological Process	Regulation of proteolysis	7	2	82	0.0049

GO:0009617	XLOC_008837; XLOC_011014	Biological Process	Response to bacterium	7	2	82	0.0055
GO:0000902	XLOC_003872; XLOC_015601; XLOC_020088	Biological Process	Cell morphogenesis	7	3	82	0.0058
GO:0045595	XLOC_001118; XLOC_010800	Biological Process	Regulation of cell differentiation	7	2	82	0.0060
GO:0001836	XLOC_005665; XLOC_012793	Biological Process	Release of cytochrome c from mitochondria	7	2	82	0.0067
GO:0033371	XLOC_018104	Biological Process	T cell secretory granule organization	7	1	82	0.0117
GO:0035684	XLOC_011014	Biological Process	Helper T cell extravasation	7	1	82	0.0117
GO:0048073	XLOC_010383	Biological Process	Regulation of eye pigmentation	7	1	82	0.0117
GO:0033364	XLOC_018104	Biological Process	Mast cell secretory granule organization	7	1	82	0.0175
GO:0070741	XLOC_007525	Biological Process	Response to interleukin-6	7	1	82	0.0175
GO:0035914	XLOC_001733; XLOC_024894	Biological Process	Skeletal muscle cell differentiation	7	2	82	0.0199
GO:0006002	XLOC_026730	Biological Process	Fructose 6-phosphate metabolic process	7	1	82	0.0233
GO:0051782	XLOC_005665	Biological Process	Negative regulation of cell division	7	1	82	0.0233
GO:0071205	XLOC_016188	Biological Process	Protein localization to juxtaparanode region of axon	7	1	82	0.0233

GO:0071398	XLOC_021372	Biological Process	Cellular response to fatty acid	7	1	82	0.0233
GO:0002175	XLOC_016188	Biological Process	Protein localization to paranode region of axon	7	1	82	0.0290
GO:0046541	XLOC_009913	Biological Process	Saliva secretion	7	1	82	0.0290
GO:0033632	XLOC_005156	Biological Process	Regulation of cell-cell adhesion mediated by integrin	7	1	82	0.0347
GO:0006924	XLOC_007166	Biological Process	Activation-induced cell death of T cells	7	1	82	0.0404
GO:0006111	XLOC_026730	Biological Process	Regulation of gluconeogenesis	7	1	82	0.0460
GO:0032494	XLOC_020881	Biological Process	Response to peptidoglycan	7	1	82	0.0460
GO:0042416	XLOC_007166	Biological Process	Dopamine biosynthetic process	7	1	82	0.0460
GO:0048103	XLOC_007166	Biological Process	Somatic stem cell division	7	1	82	0.0460
GO:0048541	XLOC_005156	Biological Process	Peyer's patch development	7	1	82	0.0517
GO:0060445	XLOC_020881	Biological Process	Branching involved in salivary gland morphogenesis	7	1	82	0.0572
GO:0060674	XLOC_010800	Biological Process	Placenta blood vessel development	7	1	82	0.0572
GO:0007050	XLOC_005665; XLOC_011269	Biological Process	Cell cycle arrest	7	2	82	0.0574

GO:0010906	XLOC_021372	Biological Process	Regulation of glucose metabolic process	7	1	82	0.0628
GO:0031032	XLOC_026812	Biological Process	Actomyosin structure organization	7	1	82	0.0738
GO:0030099	XLOC_008837	Biological Process	Myeloid cell differentiation	7	1	82	0.0792
GO:0034614	XLOC_001733	Biological Process	Cellular response to reactive oxygen species	7	1	82	0.0792
GO:0071346	XLOC_011014	Biological Process	Cellular response to interferon-gamma	7	1	82	0.0846
GO:0001829	XLOC_005156	Biological Process	Trophectodermal cell differentiation	7	1	82	0.0954
GO:0034612	XLOC_007525	Biological Process	Response to tumor necrosis factor	7	1	82	0.0954
GO:0007346	XLOC_001118	Biological Process	Regulation of mitotic cell cycle	7	1	82	0.1007
GO:0030866	XLOC_016188	Biological Process	Cortical actin cytoskeleton organization	7	1	82	0.1007
GO:0007166	XLOC_015548; XLOC_021530	Biological Process	Cell surface receptor signaling pathway	7	2	82	0.1018
GO:0001578	XLOC_009705	Biological Process	Microtubule bundle formation	7	1	82	0.1060
GO:0007159	XLOC_007669	Biological Process	Leukocyte cell-cell adhesion	7	1	82	0.1112
GO:0032868	XLOC_024894	Biological Process	Response to insulin	7	1	82	0.1112

GO:0070555	XLOC_007525	Biological Process	Response to interleukin-1	7	1	82	0.1112
GO:0007157	XLOC_007669	Biological Process	Heterophilic cell-cell adhesion via plasma membrane cell adhesion molecules	7	1	82	0.1164
GO:0010332	XLOC_005665	Biological Process	Response to gamma radiation	7	1	82	0.1164
GO:0071407	XLOC_011014	Biological Process	Cellular response to organic cyclic compound	7	1	82	0.1216
GO:0030855	XLOC_007447	Biological Process	Epithelial cell differentiation	7	1	82	0.1371
GO:0048812	XLOC_016188	Biological Process	Neuron projection morphogenesis	7	1	82	0.1371
GO:0051781	XLOC_007166	Biological Process	Positive regulation of cell division	7	1	82	0.1371
GO:0060325	XLOC_007166	Biological Process	Face morphogenesis	7	1	82	0.1572
GO:0016311	XLOC_013166; XLOC_026730	Biological Process	Dephosphorylation	7	2	82	0.1588
GO:0006915	XLOC_012793; XLOC_022742; XLOC_026769	Biological Process	Apoptotic process	7	3	82	0.1643
GO:0001938	XLOC_019618	Biological Process	Positive regulation of endothelial cell proliferation	7	1	82	0.1720
GO:0016485	XLOC_018104	Biological Process	Protein processing	7	1	82	0.1769
GO:0030307	XLOC_012793	Biological Process	Positive regulation of cell growth	7	1	82	0.2102

GO:0006886	XLOC_002436; XLOC_007556; XLOC_010661	Biological Process	Intracellular protein transport	7	3	82	0.2255
GO:0071805	XLOC_009913	Biological Process	Potassium ion transmembrane transport	7	1	82	0.2332
GO:0042742	XLOC_008837	Biological Process	Defense response to bacterium	7	1	82	0.2467
GO:0006897	XLOC_002436	Biological Process	Endocytosis	7	1	82	0.2555
GO:0001501	XLOC_007166	Biological Process	Skeletal system development	7	1	82	0.3228
GO:0007411	XLOC_007166	Biological Process	Axon guidance	7	1	82	0.3347
GO:0007399	XLOC_001733	Biological Process	Nervous system development	7	1	82	0.3692
GO:0035556	XLOC_010800; XLOC_011718	Biological Process	Intracellular signal transduction	7	2	82	0.3737
GO:0030308	XLOC_026730	Biological Process	Negative regulation of cell growth	7	1	82	0.3948
GO:0006457	XLOC_013547	Biological Process	Protein folding	7	1	82	0.5025
GO:0016310	XLOC_011157	Biological Process	Phosphorylation	7	1	82	0.8827
GO:0006954	XLOC_007447; XLOC_007525; XLOC_007669; XLOC_011014; XLOC_011852; XLOC_020881	Biological Process	Inflammatory response	6	6	82	0.0000
GO:0035690	XLOC_005665; XLOC_026730	Biological Process	Cellular response to drug	6	2	82	0.0055

GO:0042110	XLOC_005156; XLOC_020881	Biological Process	T cell activation	6	2	82	0.0109
GO:0001781	XLOC_020881	Biological Process	Neutrophil apoptotic process	6	1	82	0.0117
GO:0002384	XLOC_020881	Biological Process	Hepatic immune response	6	1	82	0.0117
GO:0030322	XLOC_009913	Biological Process	Stabilization of membrane potential	6	1	82	0.0117
GO:0002920	XLOC_010383	Biological Process	Regulation of humoral immune response	6	1	82	0.0175
GO:0010565	XLOC_021372	Biological Process	Regulation of cellular ketone metabolic process	6	1	82	0.0175
GO:0033629	XLOC_017082	Biological Process	Negative regulation of cell adhesion mediated by integrin	6	1	82	0.0175
GO:0050679	XLOC_005665; XLOC_020881	Biological Process	Positive regulation of epithelial cell proliferation	6	2	82	0.0230
GO:0090286	XLOC_014159	Biological Process	Cytoskeletal anchoring at nuclear membrane	6	1	82	0.0233
GO:0001300	XLOC_017082	Biological Process	Chronological cell aging	6	1	82	0.0290
GO:0071800	XLOC_021929	Biological Process	Podosome assembly	6	1	82	0.0290
GO:0097320	XLOC_021929	Biological Process	Membrane tubulation	6	1	82	0.0290
GO:0002686	XLOC_005156	Biological Process	Negative regulation of leukocyte migration	6	1	82	0.0347

GO:0006884	XLOC_009913	Biological Process	Cell volume homeostasis	6	1	82	0.0404
GO:0002548	XLOC_011014	Biological Process	Monocyte chemotaxis	6	1	82	0.0460
GO:0002687	XLOC_007669	Biological Process	Positive regulation of leukocyte migration	6	1	82	0.0460
GO:0050901	XLOC_007669	Biological Process	Leukocyte tethering or rolling	6	1	82	0.0460
GO:0007588	XLOC_007981	Biological Process	Excretion	6	1	82	0.0517
GO:0048246	XLOC_011014	Biological Process	Macrophage chemotaxis	6	1	82	0.0517
GO:0007338	XLOC_017407	Biological Process	Single fertilization	6	1	82	0.0572
GO:0016051	XLOC_024317	Biological Process	Carbohydrate biosynthetic process	6	1	82	0.0572
GO:0043616	XLOC_012793	Biological Process	Keratinocyte proliferation	6	1	82	0.0572
GO:0030198	XLOC_007166; XLOC_007447	Biological Process	Extracellular matrix organization	6	2	82	0.0621
GO:0030100	XLOC_002436	Biological Process	Regulation of endocytosis	6	1	82	0.0628
GO:0006911	XLOC_021929	Biological Process	Phagocytosis, engulfment	6	1	82	0.0683
GO:0051726	XLOC_012793; XLOC_026769	Biological Process	Regulation of cell cycle	6	2	82	0.0702

GO:0007599	XLOC_019618	Biological Process	Hemostasis	6	1	82	0.0738
GO:0043277	XLOC_011014	Biological Process	Apoptotic cell clearance	6	1	82	0.0738
GO:0042157	XLOC_023147	Biological Process	Lipoprotein metabolic process	6	1	82	0.0846
GO:0001782	XLOC_010383	Biological Process	B cell homeostasis	6	1	82	0.0900
GO:0046716	XLOC_020881	Biological Process	Muscle cell cellular homeostasis	6	1	82	0.0900
GO:0031668	XLOC_001733	Biological Process	Cellular response to extracellular stimulus	6	1	82	0.1007
GO:0048661	XLOC_020881	Biological Process	Positive regulation of smooth muscle cell proliferation	6	1	82	0.1060
GO:0007165	XLOC_005533; XLOC_007556; XLOC_011718; XLOC_020541; XLOC_021372; XLOC_021530; XLOC_024579; XLOC_024907	Biological Process	Signal transduction	6	8	82	0.1153
GO:0043029	XLOC_010383	Biological Process	T cell homeostasis	6	1	82	0.1164
GO:0045787	XLOC_007166	Biological Process	Positive regulation of cell cycle	6	1	82	0.1216
GO:0071310	XLOC_024894	Biological Process	Cellular response to organic substance	6	1	82	0.1268
GO:0048147	XLOC_005665	Biological Process	Negative regulation of fibroblast proliferation	6	1	82	0.1320

GO:0001890	XLOC_005156	Biological Process	Placenta development	6	1	82	0.1371
GO:0001974	XLOC_007166	Biological Process	Blood vessel remodeling	6	1	82	0.1371
GO:0072659	XLOC_016188	Biological Process	Protein localization to plasma membrane	6	1	82	0.1371
GO:0007596	XLOC_019618	Biological Process	Blood coagulation	6	1	82	0.1913
GO:0071456	XLOC_024579	Biological Process	Cellular response to hypoxia	6	1	82	0.1913
GO:0048146	XLOC_005665	Biological Process	Positive regulation of fibroblast proliferation	6	1	82	0.1961
GO:0030154	XLOC_024579; XLOC_026769	Biological Process	Cell differentiation	6	2	82	0.2138
GO:0071260	XLOC_017390	Biological Process	Cellular response to mechanical stimulus	6	1	82	0.2195
GO:0007160	XLOC_011852	Biological Process	Cell-matrix adhesion	6	1	82	0.2555
GO:0031175	XLOC_020881	Biological Process	Neuron projection development	6	1	82	0.2857
GO:0030097	XLOC_007166	Biological Process	Hemopoiesis	6	1	82	0.3106
GO:0009790	XLOC_007447	Biological Process	Embryo development	6	1	82	0.3425
GO:0015031	XLOC_005533; XLOC_007556; XLOC_010661	Biological Process	Protein transport	6	3	82	0.3757

GO:0016192	XLOC_002436	Biological Process	Vesicle-mediated transport	6	1	82	0.6239
GO:0006974	XLOC_005665	Biological Process	Cellular response to DNA damage stimulus	6	1	82	0.6874
GO:0006508	XLOC_019252	Biological Process	Proteolysis	6	1	82	0.8948
GO:0005975	XLOC_003686; XLOC_005214; XLOC_007525; XLOC_024317; XLOC_026730	Biological Process	Carbohydrate metabolic process	5	5	82	0.0014
GO:0045926	XLOC_009648; XLOC_009651	Biological Process	Negative regulation of growth	5	2	82	0.0022
GO:0009611	XLOC_011014;XLOC_020881	Biological Process	Response to wounding	5	2	82	0.0067
GO:0010273	XLOC_009651	Biological Process	Detoxification of copper ion	5	1	82	0.0117
GO:0070887	XLOC_017082	Biological Process	Cellular response to chemical stimulus	5	1	82	0.0117
GO:0008228	XLOC_000961	Biological Process	Opsonization	5	1	82	0.0175
GO:0009612	XLOC_007525	Biological Process	Response to mechanical stimulus	5	1	82	0.0290
GO:0042594	XLOC_021372	Biological Process	Response to starvation	5	1	82	0.0404
GO:0007275	XLOC_002260; XLOC_011852; XLOC_024579; XLOC_026769	Biological Process	Multicellular organismal development	5	4	82	0.0527
GO:0042493	XLOC_001733; XLOC_005665	Biological Process	Response to drug	5	2	82	0.0702

GO:0009314	XLOC_005665	Biological Process	Response to radiation	5	1	82	0.0792
GO:0007010	XLOC_011014; XLOC_014159	Biological Process	Cytoskeleton organization	5	2	82	0.0838
GO:0042127	XLOC_017082; XLOC_020881	Biological Process	Regulation of cell proliferation	5	2	82	0.1130
GO:0072593	XLOC_021372	Biological Process	Reactive oxygen species metabolic process	5	1	82	0.1164
GO:0006909	XLOC_008837	Biological Process	Phagocytosis	5	1	82	0.1268
GO:0051276	XLOC_005665	Biological Process	Chromosome organization	5	1	82	0.1522
GO:0016049	XLOC_007166	Biological Process	Cell growth	5	1	82	0.1671
GO:0006869	XLOC_023147	Biological Process	Lipid transport	5	1	82	0.2008
GO:0006935	XLOC_011014	Biological Process	Chemotaxis	5	1	82	0.2287
GO:0051607	XLOC_020881	Biological Process	Defense response to virus	5	1	82	0.2686
GO:0001666	XLOC_005156	Biological Process	Response to hypoxia	5	1	82	0.3307
GO:0008284	XLOC_005665; XLOC_020881	Biological Process	Positive regulation of cell proliferation	5	2	82	0.3862
GO:0055085	XLOC_003967; XLOC_010383	Biological Process	Transmembrane transport	5	2	82	0.6271

GO:0007049	XLOC_011269	Biological Process	Cell cycle	5	1	82	0.6393
GO:0006810	XLOC_003967; XLOC_007556; XLOC_028876; XLOC_028877	Biological Process	Transport	5	4	82	0.7307
GO:0008285	XLOC_012793	Biological Process	Negative regulation of cell proliferation	5	1	82	0.7324
GO:0055114	XLOC_004450; XLOC_022114	Biological Process	Oxidation-reduction process	5	2	82	0.8048
GO:0006950	XLOC_003872; XLOC_015601; XLOC_026769	Biological Process	Response to stress	4	3	82	0.0137
GO:0009653	XLOC_007447	Biological Process	Anatomical structure morphogenesis	4	1	82	0.0404
GO:0044419	XLOC_011852	Biological Process	Interspecies interaction between organisms	4	1	82	0.0628
GO:0006955	XLOC_008837; XLOC_011014; XLOC_020881	Biological Process	Immune response	4	3	82	0.0630
GO:0040008	XLOC_010800	Biological Process	Regulation of growth	4	1	82	0.1164
GO:0050900	XLOC_007669	Biological Process	Leukocyte migration	4	1	82	0.1164
GO:0007155	XLOC_007669; XLOC_011852; XLOC_027571	Biological Process	Cell adhesion	4	3	82	0.1831
GO:0032259	XLOC_017919	Biological Process	Methylation	4	1	82	0.4529
GO:0040007	XLOC_007166	Biological Process	Growth	3	1	82	0.1421

GO:0008152	XLOC_017919; XLOC_020541; XLOC_022114; XLOC_024317; XLOC_026730	Biological Process	Metabolic process	3	5	82	0.2259
GO:0034993	XLOC_014159	Cellular Component	SUN-KASH complex	13	1	82	0.0290
GO:0030173	XLOC_005319	Cellular Component	Integral component of Golgi membrane	13	1	82	0.1572
GO:0016604	XLOC_005665	Cellular Component	Nuclear body	13	1	82	0.1865
GO:0030018	XLOC_017390	Cellular Component	Z disc	13	1	82	0.2422
GO:0005743	XLOC_021372	Cellular Component	Mitochondrial inner membrane	13	1	82	0.7738
GO:0045095	XLOC_021897; XLOC_022742	Cellular Component	Keratin filament	12	2	82	0.0030
GO:0042629	XLOC_018104	Cellular Component	Mast cell granule	12	1	82	0.0117
GO:0030136	XLOC_002436	Cellular Component	Clathrin-coated vesicle	12	1	82	0.1421
GO:0005882	XLOC_021897; XLOC_022742	Cellular Component	Intermediate filament	11	2	82	0.0230
GO:0030131	XLOC_002436	Cellular Component	Clathrin adaptor complex	11	1	82	0.1268
GO:0008180	XLOC_020088	Cellular Component	COP9 signalosome	11	1	82	0.1572
GO:0005764	XLOC_005156; XLOC_007556	Cellular Component	Lysosome	11	2	82	0.1796

GO:0016459	XLOC_020249	Cellular Component	Myosin complex	11	1	82	0.2102
GO:0005874	XLOC_003872; XLOC_009705	Cellular Component	Microtubule	11	2	82	0.2289
GO:0008021	XLOC_002436	Cellular Component	Synaptic vesicle	11	1	82	0.2643
GO:0005730	XLOC_002436; XLOC_005665; XLOC_010661; XLOC_022114	Cellular Component	Nucleolus	11	4	82	0.2771
GO:0005815	XLOC_022742	Cellular Component	Microtubule organizing center	11	1	82	0.3065
GO:0005635	XLOC_014159	Cellular Component	Nuclear envelope	11	1	82	0.3579
GO:0005654	XLOC_005665	Cellular Component	Nucleoplasm	11	1	82	0.5556
GO:0034451	XLOC_022742	Cellular Component	Centriolar satellite	10	1	82	0.0460
GO:0005770	XLOC_007556	Cellular Component	Late endosome	10	1	82	0.2772
GO:0005802	XLOC_007556	Cellular Component	Trans-Golgi network	10	1	82	0.3386
GO:0033270	XLOC_016188	Cellular Component	Paranode region of axon	9	1	82	0.0347
GO:0030673	XLOC_016188	Cellular Component	Axolemma	9	1	82	0.0404
GO:0044224	XLOC_016188	Cellular Component	Juxtaparanode region of axon	9	1	82	0.0404

GO:0002102	XLOC_021929	Cellular Component	Podosome	9	1	82	0.0683
GO:0005856	XLOC_009705; XLOC_016188; XLOC_019428; XLOC_026812	Cellular Component	Cytoskeleton	9	4	82	0.1084
GO:0005794	XLOC_002260; XLOC_005319; XLOC_007447; XLOC_007556; XLOC_018104	Cellular Component	Golgi apparatus	9	5	82	0.2400
GO:0005819	XLOC_005665	Cellular Component	Spindle	9	1	82	0.3347
GO:0048471	XLOC_009648; XLOC_009651	Cellular Component	Perinuclear region of cytoplasm	9	2	82	0.4474
GO:0005768	XLOC_007556	Cellular Component	Endosome	9	1	82	0.5838
GO:0005634	XLOC_000002; XLOC_001118; XLOC_001733; XLOC_003872; XLOC_005533; XLOC_005665; XLOC_007447; XLOC_007556; XLOC_007981; XLOC_008837; XLOC_009240; XLOC_009648; XLOC_009651; XLOC_010661; XLOC_012793; XLOC_015548; XLOC_024894; XLOC_024907; XLOC_026769	Cellular Component	Nucleus	9	19	82	0.7240
GO:0005829	XLOC_017390; XLOC_026730	Cellular Component	Cytosol	9	2	82	0.8362
GO:0005783	XLOC_007525	Cellular Component	Endoplasmic reticulum	9	1	82	0.9613
GO:0005739	XLOC_021372	Cellular Component	Mitochondrion	9	1	82	0.9995

GO:0005896	XLOC_020881	Cellular Component	Interleukin-6 receptor complex	8	1	82	0.0233
GO:0005640	XLOC_014159	Cellular Component	Nuclear outer membrane	8	1	82	0.0572
GO:0008305	XLOC_011852	Cellular Component	Integrin complex	8	1	82	0.1572
GO:0001891	XLOC_021929	Cellular Component	Phagocytic cup	7	1	82	0.0792
GO:0005737	XLOC_000002; XLOC_001118; XLOC_002436; XLOC_003872; XLOC_004450; XLOC_005156; XLOC_007447; XLOC_007525; XLOC_007556; XLOC_009648; XLOC_009651; XLOC_009705; XLOC_010661; XLOC_012793; XLOC_016188; XLOC_017390; XLOC_019428; XLOC_021929; XLOC_022114; XLOC_022742; XLOC_024317; XLOC_024894; XLOC_024907; XLOC_026730; XLOC_026812	Cellular Component	Cytoplasm	7	25	82	0.0973
GO:0005834	XLOC_011718	Cellular Component	Heterotrimeric G-protein complex	7	1	82	0.1720
GO:0030424	XLOC_007166	Cellular Component	Axon	7	1	82	0.4194
GO:0005667	XLOC_001733	Cellular Component	Transcription factor complex	7	1	82	0.5838

GO:0009897	XLOC_005156; XLOC_007669; XLOC_020881	Cellular Component	External side of plasma membrane	6	3	82	0.0583
GO:0009898	XLOC_005533	Cellular Component	Cytoplasmic side of plasma membrane	6	1	82	0.1720
GO:0005604	XLOC_027571	Cellular Component	Basement membrane	6	1	82	0.3347
GO:0043025	XLOC_007166	Cellular Component	Neuronal cell body	6	1	82	0.4125
GO:0016021	XLOC_002260; XLOC_003967; XLOC_005319; XLOC_009913; XLOC_010383; XLOC_011269; XLOC_011852; XLOC_013217; XLOC_014159; XLOC_015548; XLOC_019618; XLOC_021530	Cellular Component	Integral component of membrane	6	12	82	0.6111
GO:0005615	XLOC_007166; XLOC_007525; XLOC_011014; XLOC_017082; XLOC_018104; XLOC_019252; XLOC_019618; XLOC_020881; XLOC_024579; XLOC_026660	Cellular Component	Extracellular space	5	10	82	0.0001
GO:0019898	XLOC_016188; XLOC_026812	Cellular Component	Extrinsic component of membrane	5	2	82	0.0094
GO:0043296	XLOC_020249	Cellular Component	Apical junction complex	5	1	82	0.0792
GO:0005578	XLOC_019252; XLOC_024579	Cellular Component	Proteinaceous extracellular matrix	5	2	82	0.1425
GO:0045177	XLOC_026812	Cellular Component	Apical part of cell	5	1	82	0.3024

GO:0009986	XLOC_005156; XLOC_019618	Cellular Component	Cell surface	5	2	82	0.4455
GO:0005886	XLOC_002260; XLOC_005156; XLOC_010661; XLOC_011269; XLOC_015548; XLOC_017390; XLOC_021530	Cellular Component	Plasma membrane	5	7	82	0.6731
GO:0005622	XLOC_005533; XLOC_007556; XLOC_010661 ;XLOC_010800; XLOC_024894	Cellular Component	Intracellular	5	5	82	0.9854
GO:0005911	XLOC_016188	Cellular Component	Cell-cell junction	4	1	82	0.3948
GO:0043234	XLOC_005665	Cellular Component	Protein complex	4	1	82	0.6008
GO:0005576	XLOC_007166; XLOC_011014; XLOC_012793; XLOC_017082; XLOC_019252; XLOC_020881; XLOC_022018; XLOC_023147; XLOC_024579; XLOC_026660; XLOC_027571; XLOC_028876; XLOC_028877	Cellular Component	Extracellular region	3	13	82	0.0000
GO:0031012	XLOC_017082; XLOC_019252; XLOC_019618; XLOC_027571	Cellular Component	Extracellular matrix	3	4	82	0.0146
GO:0030054	XLOC_015548	Cellular Component	Cell junction	3	1	82	0.6261

GO:0016020	XLOC_002260; XLOC_003686; XLOC_005156; XLOC_005319; XLOC_005533; XLOC_007556; XLOC_007669; XLOC_010661; XLOC_011269; XLOC_011718; XLOC_011852; XLOC_013217; XLOC_015548; XLOC_019618; XLOC_021530	Cellular Component	Membrane	3	15	82	0.6854
GO:0043498	XLOC_019618	Molecular Function	Cell surface binding	NA	1	82	0.0460
GO:0042626	XLOC_003967	Molecular Function	ATPase activity, coupled to transmembrane movement of substances	12	1	82	0.1720
GO:0016286	XLOC_009913	Molecular Function	Small conductance calcium-activated potassium channel activity	11	1	82	0.0233
GO:0042132	XLOC_026730	Molecular Function	Fructose 1,6-bisphosphate 1-phosphatase activity	10	1	82	0.0175
GO:0017017	XLOC_013166	Molecular Function	MAP kinase tyrosine/serine/threonine phosphatase activity	10	1	82	0.0683
GO:0015269	XLOC_009913	Molecular Function	Calcium-activated potassium channel activity	10	1	82	0.0738
GO:0005267	XLOC_009913	Molecular Function	Potassium channel activity	10	1	82	0.2149
GO:0031727	XLOC_011014	Molecular Function	CCR2 chemokine receptor binding	9	1	82	0.0175
GO:0016208	XLOC_026730	Molecular Function	AMP binding	9	1	82	0.0290
GO:0000155	XLOC_021372	Molecular Function	Phosphorelay sensor kinase activity	9	1	82	0.0628

GO:0004114	XLOC_020541	Molecular Function	3',5'-cyclic-nucleotide phosphodiesterase activity	9	1	82	0.1164
GO:0070888	XLOC_005665	Molecular Function	E-box binding	9	1	82	0.1164
GO:0000976	XLOC_024894	Molecular Function	Transcription regulatory region sequence-specific DNA binding	9	1	82	0.1371
GO:0003924	XLOC_005533; XLOC_007556; XLOC_010661	Molecular Function	GTPase activity	9	3	82	0.1920
GO:0019003	XLOC_005533	Molecular Function	GDP binding	9	1	82	0.2055
GO:0008138	XLOC_013166	Molecular Function	Protein tyrosine/serine/threonine phosphatase activity	9	1	82	0.2241
GO:0003774	XLOC_020249	Molecular Function	Motor activity	9	1	82	0.2377
GO:0005525	XLOC_005533; XLOC_007556; XLOC_010661	Molecular Function	GTP binding	9	3	82	0.3548
GO:0004725	XLOC_013166	Molecular Function	Protein tyrosine phosphatase activity	9	1	82	0.4229
GO:0016887	XLOC_003967	Molecular Function	ATPase activity	9	1	82	0.5113
GO:0005524	XLOC_001118; XLOC_003872; XLOC_003967; XLOC_011157; XLOC_015601; XLOC_020088; XLOC_021372	Molecular Function	ATP binding	9	7	82	0.5794
GO:0004380	XLOC_003686	Molecular Function	Glycoprotein-fucosylgalactoside alpha-N acetylgalacto-saminyltransferase activity	8	1	82	0.0117
GO:0004381	XLOC_003686	Molecular Function	Fucosylgalactoside 3-alpha-galactosyltransferase activity	8	1	82	0.0117

GO:0004360	XLOC_024317	Molecular Function	Glutamine-fructose-6-phosphate transaminase (isomerizing) activity	8	1	82	0.0175
GO:0030297	XLOC_024579	Molecular Function	Transmembrane receptor protein tyrosine kinase activator activity	8	1	82	0.0175
GO:0004673	XLOC_021372	Molecular Function	Protein histidine kinase activity	8	1	82	0.0404
GO:0008009	XLOC_011014	Molecular Function	Chemokine activity	8	1	82	0.1817
GO:0044212	XLOC_001733; XLOC_024894	Molecular Function	Transcription regulatory region DNA binding	8	2	82	0.2009
GO:0003690	XLOC_001733	Molecular Function	Double-stranded DNA binding	8	1	82	0.2102
GO:0004222	XLOC_019252	Molecular Function	Metalloendopeptidase activity	8	1	82	0.3729
GO:0004721	XLOC_013166	Molecular Function	Phosphoprotein phosphatase activity	8	1	82	0.4229
GO:0017111	XLOC_003967	Molecular Function	Nucleoside-triphosphatase activity	8	1	82	0.5583
GO:0004674	XLOC_001118; XLOC_011157	Molecular Function	Protein serine/threonine kinase activity	8	2	82	0.6271
GO:0008270	XLOC_005156; XLOC_007981; XLOC_009648; XLOC_009651; XLOC_016728; XLOC_019252; XLOC_024894; XLOC_024907	Molecular Function	Zinc ion binding	8	8	82	0.6623
GO:0004713	XLOC_001118; XLOC_011157	Molecular Function	Protein tyrosine kinase activity	8	2	82	0.7523
GO:0004867	XLOC_004817; XLOC_014139; XLOC_017082	Molecular Function	Serine-type endopeptidase inhibitor activity	7	3	82	0.0041

GO:0004999	XLOC_021530	Molecular Function	Vasoactive intestinal polypeptide receptor activity	7	1	82	0.0233
GO:0003836	XLOC_005319	Molecular Function	Beta-galactoside (CMP) alpha-2,3-sialyltransferase activity	7	1	82	0.0290
GO:0004568	XLOC_007525	Molecular Function	Chitinase activity	7	1	82	0.0290
GO:0005138	XLOC_020881	Molecular Function	Interleukin-6 receptor binding	7	1	82	0.0290
GO:0033691	XLOC_007669	Molecular Function	Sialic acid binding	7	1	82	0.0290
GO:0008140	XLOC_001118	Molecular Function	cAMP response element binding protein binding	7	1	82	0.0347
GO:0004745	XLOC_022114	Molecular Function	Retinol dehydrogenase activity	7	1	82	0.0404
GO:0004000	XLOC_005156	Molecular Function	Adenosine deaminase activity	7	1	82	0.0460
GO:0005160	XLOC_007166	Molecular Function	Transforming growth factor beta receptor binding	7	1	82	0.0517
GO:0000975	XLOC_008837	Molecular Function	Regulatory region DNA binding	7	1	82	0.0683
GO:0043014	XLOC_003872	Molecular Function	Alpha-tubulin binding	7	1	82	0.0900
GO:0043565	XLOC_001733; XLOC_007447; XLOC_007981; XLOC_009240; XLOC_024894	Molecular Function	Sequence-specific DNA binding	7	5	82	0.1133
GO:0000287	XLOC_001118; XLOC_005533	Molecular Function	Magnesium ion binding	7	2	82	0.2289

GO:0008081	XLOC_020541	Molecular Function	Phosphoric diester hydrolase activity	7	1	82	0.2467
GO:0019903	XLOC_009913	Molecular Function	Protein phosphatase binding	7	1	82	0.2643
GO:0008237	XLOC_019252	Molecular Function	Metallopeptidase activity	7	1	82	0.4160
GO:0019901	XLOC_001118; XLOC_012793	Molecular Function	Protein kinase binding	7	2	82	0.4213
GO:0016791	XLOC_013166	Molecular Function	Phosphatase activity	7	1	82	0.4995
GO:0004672	XLOC_001118; XLOC_011157; XLOC_021372	Molecular Function	Protein kinase activity	7	3	82	0.5887
GO:0005509	XLOC_022018; XLOC_027571	Molecular Function	Calcium ion binding	7	2	82	0.7929
GO:0002020	XLOC_017082; XLOC_019252; XLOC_019618	Molecular Function	Protease binding	6	3	82	0.0026
GO:0001872	XLOC_000961	Molecular Function	Beta-D-glucan binding	6	1	82	0.0175
GO:0030215	XLOC_002260	Molecular Function	Semaphorin receptor binding	6	1	82	0.0404
GO:0042578	XLOC_026730	Molecular Function	Phosphoric ester hydrolase activity	6	1	82	0.0404
GO:0000982	XLOC_024894	Molecular Function	RNA polymerase II core promoter proximal region sequence-specific DNA binding transcription factor activity	6	1	82	0.0460
GO:0005523	XLOC_019428	Molecular Function	Tropomyosin binding	6	1	82	0.0517

GO:0001664	XLOC_011014	Molecular Function	G-protein coupled receptor binding	6	1	82	0.0738
GO:0005125	XLOC_011014; XLOC_020881	Molecular Function	Cytokine activity	6	2	82	0.0786
GO:0008083	XLOC_007166; XLOC_020881	Molecular Function	Growth factor activity	6	2	82	0.0786
GO:0008373	XLOC_005319	Molecular Function	Sialyltransferase activity	6	1	82	0.0846
GO:0008483	XLOC_024317	Molecular Function	Transaminase activity	6	1	82	0.0846
GO:0035035	XLOC_024894	Molecular Function	Histone acetyltransferase binding	6	1	82	0.0846
GO:0070412	XLOC_001733	Molecular Function	R-SMAD binding	6	1	82	0.0900
GO:0003779	XLOC_014159; XLOC_016188; XLOC_019428	Molecular Function	Actin binding	6	3	82	0.1133
GO:0005179	XLOC_026660	Molecular Function	Hormone activity	6	1	82	0.1371
GO:0015631	XLOC_009705	Molecular Function	Tubulin binding	6	1	82	0.1371
GO:0030971	XLOC_024579	Molecular Function	Receptor tyrosine kinase binding	6	1	82	0.1421
GO:0070491	XLOC_005665	Molecular Function	Repressing transcription factor binding	6	1	82	0.1421
GO:0004553	XLOC_007525	Molecular Function	Hydrolase activity, hydrolyzing O-glycosyl compounds	6	1	82	0.1720

GO:0016758	XLOC_003686	Molecular Function	Transferase activity, transferring hexosyl groups	6	1	82	0.1865
GO:0005178	XLOC_011852	Molecular Function	Integrin binding	6	1	82	0.2287
GO:0042826	XLOC_001118	Molecular Function	Histone deacetylase binding	6	1	82	0.2857
GO:0004930	XLOC_015548; XLOC_021530	Molecular Function	G-protein coupled receptor activity	6	2	82	0.3267
GO:0003677	XLOC_001733; XLOC_005665; XLOC_007447; XLOC_007981; XLOC_008837; XLOC_009240; XLOC_024894; XLOC_024907	Molecular Function	DNA binding	6	8	82	0.3367
GO:0046872	XLOC_007981; XLOC_009648; XLOC_009649; XLOC_009651; XLOC_013547; XLOC_016728; XLOC_019252; XLOC_020541; XLOC_024907; XLOC_026730	Molecular Function	Metal ion binding	6	10	82	0.3607
GO:0008168	XLOC_017919	Molecular Function	Methyltransferase activity	6	1	82	0.4875
GO:0003713	XLOC_007447	Molecular Function	Transcription coactivator activity	6	1	82	0.4935
GO:0046982	XLOC_001733	Molecular Function	Protein heterodimerization activity	6	1	82	0.6456
GO:0016301	XLOC_011157	Molecular Function	Kinase activity	6	1	82	0.8895

GO:0008061	XLOC_007525	Molecular Function	Chitin binding	5	1	82	0.0233
GO:0048029	XLOC_026730	Molecular Function	Monosaccharide binding	5	1	82	0.0290
GO:0008092	XLOC_016188; XLOC_026812	Molecular Function	Cytoskeletal protein binding	5	2	82	0.0335
GO:0046983	XLOC_001733; XLOC_005665; XLOC_009240	Molecular Function	Protein dimerization activity	5	3	82	0.0680
GO:0071889	XLOC_001118	Molecular Function	14-3-3 protein binding	5	1	82	0.0683
GO:0097110	XLOC_022742	Molecular Function	Scaffold protein binding	5	1	82	0.0738
GO:0005102	XLOC_011852; XLOC_017082; XLOC_024579	Molecular Function	Receptor binding	5	3	82	0.0905
GO:0004888	XLOC_015548; XLOC_021530	Molecular Function	Transmembrane signaling receptor activity	5	2	82	0.0963
GO:0051219	XLOC_012793	Molecular Function	Phosphoprotein binding	5	1	82	0.1216
GO:0048306	XLOC_007669	Molecular Function	Calcium-dependent protein binding	5	1	82	0.1268
GO:0001948	XLOC_007669	Molecular Function	Glycoprotein binding	5	1	82	0.1421
GO:0016757	XLOC_005214;XLOC_005319	Molecular Function	Transferase activity, transferring glycosyl groups	5	2	82	0.1486
GO:0030414	XLOC_017082	Molecular Function	Peptidase inhibitor activity	5	1	82	0.1572

GO:0051087	XLOC_017390	Molecular Function	Chaperone binding	5	1	82	0.1720
GO:0043169	XLOC_007525	Molecular Function	Cation binding	5	1	82	0.1817
GO:0032403	XLOC_005665	Molecular Function	Protein complex binding	5	1	82	0.1913
GO:0003707	XLOC_007981	Molecular Function	Steroid hormone receptor activity	5	1	82	0.2511
GO:0031072	XLOC_013547	Molecular Function	Heat shock protein binding	5	1	82	0.2983
GO:0005516	XLOC_009913	Molecular Function	Calmodulin binding	5	1	82	0.3228
GO:0051082	XLOC_013547	Molecular Function	Unfolded protein binding	5	1	82	0.3803
GO:0019904	XLOC_012793	Molecular Function	Protein domain specific binding	5	1	82	0.4397
GO:0000166	XLOC_001118; XLOC_003872; XLOC_003967; XLOC_005533; XLOC_007556; XLOC_010661; XLOC_011157; XLOC_015601; XLOC_020088; XLOC_022114	Molecular Function	Nucleotide binding	5	10	82	0.4722
GO:0005543	XLOC_019618; XLOC_021929	Molecular Function	Phospholipid binding	5	2	82	0.5326
GO:0008134	XLOC_005665	Molecular Function	Transcription factor binding	5	1	82	0.6478
GO:0008233	XLOC_019252	Molecular Function	Peptidase activity	5	1	82	0.7957

GO:0016772	XLOC_001118; XLOC_011157	Molecular Function	Transferase activity, transferring phosphorus-containing groups	5	2	82	0.8211
GO:0003676	XLOC_024894	Molecular Function	Nucleic acid binding	5	1	82	0.9978
GO:0005549	XLOC_028876; XLOC_028877	Molecular Function	Odorant binding	4	2	82	0.0003
GO:0030246	XLOC_000629; XLOC_007669; XLOC_024317	Molecular Function	Carbohydrate binding	4	3	82	0.0336
GO:0019239	XLOC_005156	Molecular Function	Deaminase activity	4	1	82	0.0404
GO:0003700	XLOC_001733;XLOC_005665;XLOC_007447;XLOC_007981;XLOC_008837;XLOC_009240;XLOC_024894	Molecular Function	Sequence-specific DNA binding transcription factor activity	4	7	82	0.0555
GO:0004857	XLOC_024579	Molecular Function	Enzyme inhibitor activity	4	1	82	0.0954
GO:0022857	XLOC_010383	Molecular Function	Transmembrane transporter activity	4	1	82	0.1913
GO:0004871	XLOC_011718;XLOC_021530;XLOC_024907	Molecular Function	Signal transducer activity	4	3	82	0.3338
GO:0008289	XLOC_023147	Molecular Function	Lipid binding	4	1	82	0.3803
GO:0016740	XLOC_005319; XLOC_011157; XLOC_017919; XLOC_024317	Molecular Function	Transferase activity	4	4	82	0.5613

GO:0005515	XLOC_000002; XLOC_001118; XLOC_001733; XLOC_002260; XLOC_002436; XLOC_004817; XLOC_005156; XLOC_005533; XLOC_005665; XLOC_007166; XLOC_007447; XLOC_007669; XLOC_008837; XLOC_009913; XLOC_010661; XLOC_010800; XLOC_011014; XLOC_011269; XLOC_012793; XLOC_013166; XLOC_014159; XLOC_016188; XLOC_017082; XLOC_017390; XLOC_018104; XLOC_019252; XLOC_019618; XLOC_020249; XLOC_020881; XLOC_021085; XLOC_021530; XLOC_021929; XLOC_022018;	Molecular Function	Protein binding	4	41	82	0.6551
GO:0016491	XLOC_004450; XLOC_022114	Molecular Function	Oxidoreductase activity	4	2	82	0.7200
GO:0016787	XLOC_013166; XLOC_019252; XLOC_020541; XLOC_026730	Molecular Function	Hydrolase activity	4	4	82	0.7461
GO:0005198	XLOC_016188; XLOC_021897; XLOC_022742	Molecular Function	Structural molecule activity	3	3	82	0.0288
GO:0005215	XLOC_028876; XLOC_028877	Molecular Function	Transporter activity	3	2	82	0.2289
GO:0004872	XLOC_002260; XLOC_007981; XLOC_011852; XLOC_021530	Molecular Function	Receptor activity	3	4	82	0.4068
GO:0003824	XLOC_007525; XLOC_020541; XLOC_026730	Molecular Function	Catalytic activity	3	3	82	0.7231

S gene number indicated the number of significantly down-regulated transcripts annotated to one certain GO term by GO analysis; TS gene number indicated the total number of significantly down-regulated transcripts annotated by GO analysis.

Table S7 GO categories assigned to the differentially up-regulated proteins in mammary glands between RS- and AH-fed dairy cows

GO id	Gene list	GO term	GO description	GO class	S gene number	TS gene number	P-value of Fisher's Exact Test
GO:0007010	IPI00687539;IPI00687601;IPI00687657;IPI00688489;IPI00688921;IPI00689228;IPI00689750;IPI00690160;IPI00691963;IPI00694204;IPI00695506;IPI00695666;IPI00698900; IPI00701223;IPI00703776;IPI00706141;IPI00707359;IPI00721270;IPI00829551;IPI00842934	Biological Process	Cytoskeleton organization	5	20	211	0.0030
GO:0007015	IPI00687539;IPI00687657;IPI00688489;IPI00690160;IPI00695506;IPI00695666;IPI00703776;IPI00706141	Biological Process	Actin filament organization	7	8	211	0.0049
GO:0030036	IPI00687539;IPI00687657;IPI00688489;IPI00688921;IPI00690160;IPI00691963;IPI00694204;IPI00695506;IPI00695666;IPI00698900;IPI00703776;IPI00706141;IPI00829551; IPI00842934	Biological Process	Actin cytoskeleton organization	6	14	211	0.0055
GO:0006166	IPI00686225;IPI00726650;IPI00883375	Biological Process	Purine ribonucleoside salvage	11	3	211	0.0064
GO:0043174	IPI00686225;IPI00726650;IPI00883375	Biological Process	Nucleoside salvage	9	3	211	0.0064

GO:0071822	IPI00686225;IPI00687539;IPI00687657;IPI00688489;IPI00689228;IPI00690160;IPI00691963;IPI00692468;IPI00695506;IPI00695666;IPI00698039;IPI00703731;IPI00703776;IPI00703854;IPI00705000;IPI00706141;IPI00707718;IPI00713573;IPI00721270;IPI00728768;IPI00837992;IPI01018577	Biological Process	Protein complex subunit organization	6	22	211	0.0065
GO:0030029	IPI00687539;IPI00687657;IPI00688489;IPI00688921;IPI00690160;IPI00691963;IPI00694204;IPI00695506;IPI00695666;IPI00698900;IPI00703776;IPI00706141;IPI00829551;IPI00842934	Biological Process	Actin filament-based process	5	14	211	0.0076
GO:0055002	IPI00688489;IPI00689750;IPI00692676;IPI00694204;IPI00694504;IPI00698900	Biological Process	Striated muscle cell development	9	6	211	0.0080
GO:0030203	IPI00688608;IPI00697081;IPI00702700;IPI00715287	Biological Process	Glycosaminoglycan metabolic process	7	4	211	0.0081
GO:0045598	IPI00692468;IPI00699355;IPI00703753;IPI00842934	Biological Process	Regulation of fat cell differentiation	8	4	211	0.0081

GO:0006790	IPI00688608;IPI00692468;IPI00694739;IPI00697081;IPI00703753;IPI00730144;IPI00883375	Biological Process	Sulfur compound metabolic process	5	7	211	0.0091
GO:0055001	IPI00688489;IPI00689750;IPI00692676;IPI00694204;IPI00694504;IPI00698900	Biological Process	Muscle cell development	8	6	211	0.0095
GO:0043101	IPI00686225;IPI00726650;IPI00883375	Biological Process	Purine-containing compound salvage	8	3	211	0.0098
GO:0043933	IPI00686225;IPI00687539;IPI00687657;IPI00688489;IPI00689228;IPI00690160;IPI00691963;IPI00692468;IPI00695506;IPI00695666;IPI00695965;IPI00698039;IPI00701698;IPI00703731;IPI00703776;IPI00703854;IPI00705000;IPI00706141;IPI00707718;IPI00713573;IPI00721270;IPI00728768;IPI00837992;IPI01018577	Biological Process	Macromolecular complex subunit organization	5	24	211	0.0100
GO:0043094	IPI00686225;IPI00694739;IPI00726650;IPI00883375	Biological Process	Cellular metabolic compound salvage	6	4	211	0.0106
GO:0006952	IPI00686841;IPI00687625;IPI00691212;IPI00691669;IPI00693338;IPI00694751;IPI00695965;IPI00701166;IPI00701698;IPI00707101;IPI00713757;IPI00718757	Biological Process	Defense response	5	12	211	0.0122
GO:0002683	IPI00693338;IPI00697757;IPI00700789;IPI00701698;IPI00871133;IPI00912603	Biological Process	Negative regulation of immune system process	5	6	211	0.0129

GO:0006022	IPI00688608;IPI00697081;IPI00702700;IPI00715287	Biological Process	Aminoglycan metabolic process	6	4	211	0.0134
GO:0048678	IPI00687539;IPI00692468;IPI00694504	Biological Process	Response to axon injury	6	3	211	0.0141
GO:0090066	IPI00687601;IPI00688921;IPI00690160;IPI00692468;IPI00697196;IPI00701166;IPI00706141;IPI00711750	Biological Process	Regulation of anatomical structure size	5	8	211	0.0183
GO:0009611	IPI00687539;IPI00687625;IPI00691212;IPI00692468;IPI00694504;IPI00694751;IPI00699355;IPI00701166;IPI00707101;IPI00714673;IPI00730144	Biological Process	Response to wounding	5	11	211	0.0188
GO:0034754	IPI00686601;IPI00692627;IPI00697184	Biological Process	Cellular hormone metabolic process	5	3	211	0.0192
GO:0002251	IPI00695142;IPI00695965	Biological Process	Organ or tissue specific immune response	5	2	211	0.0202
GO:0002385	IPI00695142;IPI00695965	Biological Process	Mucosal immune response	6	2	211	0.0202
GO:0019800	IPI00688608;IPI00697081	Biological Process	Peptide cross-linking via chondroitin 4-sulfate glycosaminoglycan	10	2	211	0.0202
GO:0030204	IPI00688608;IPI00697081	Biological Process	Chondroitin sulfate metabolic process	9	2	211	0.0202
GO:0046098	IPI00686225;IPI00716555	Biological Process	Guanine metabolic process	10	2	211	0.0202
GO:0050654	IPI00688608;IPI00697081	Biological Process	Chondroitin sulfate proteoglycan metabolic process	8	2	211	0.0202
GO:0090239	IPI00690446;IPI00703776	Biological Process	Regulation of histone H4 acetylation	11	2	211	0.0202
GO:2000983	IPI00700789;IPI01028487	Biological Process	Regulation of ATP citrate synthase activity	7	2	211	0.0202
GO:2000984	IPI00700789;IPI01028487	Biological Process	Negative regulation of ATP citrate synthase activity	7	2	211	0.0202

GO:0042692	IPI00688489;IPI00689750;IPI00690094;IPI00692676;IPI00694204;IPI00694504;IPI00698900	Biological Process	Muscle cell differentiation	7	7	211	0.0215
GO:0050854	IPI00693338;IPI00700789;IPI00912603	Biological Process	Regulation of antigen receptor-mediated signaling pathway	8	3	211	0.0252
GO:0051054	IPI00691963;IPI00693338;IPI01018577	Biological Process	Positive regulation of DNA metabolic process	10	3	211	0.0252
GO:0055006	IPI00688489;IPI00689750;IPI00692676	Biological Process	Cardiac cell development	8	3	211	0.0252
GO:0055013	IPI00688489;IPI00689750;IPI00692676	Biological Process	Cardiac muscle cell development	9	3	211	0.0252
GO:0050793	IPI00687539;IPI00687601;IPI00688921;IPI00689228;IPI00689750;IPI00690160;IPI00691963;IPI00692468;IPI00693338;IPI00696930;IPI00697184;IPI00698039;IPI00699355; IPI00701698;IPI00703753;IPI00706141;IPI00707101;IPI00716121;IPI00717119;IPI00842934	Biological Process	Regulation of developmental process	4	20	211	0.0259
GO:0051146	IPI00688489;IPI00689750;IPI00692676;IPI00694204;IPI00694504;IPI00698900	Biological Process	Striated muscle cell differentiation	8	6	211	0.0281

GO:0006996	IPI00686601;IPI00686966;IPI00687539;IPI00687601;IPI00687657;IPI00688489;IPI00688921;IPI00689228;IPI00689750;IPI00690160;IPI00690446;IPI00691963;IPI00692468;IPI00694204;IPI00695506;IPI00695666;IPI00695890;IPI00698039;IPI00698900;IPI00701223;IPI00701698;IPI00703731;IPI00703776;IPI00703854;IPI00705000;IPI00705941;IPI00706141;IPI00707359;IPI00715354;IPI00721270;IPI00728768;IPI00829551;IPI00842934;IPI00904732	Biological Process	Organelle organization	4	34	211	0.0284
GO:0006357	IPI00689750;IPI00691963;IPI00692468;IPI00692676;IPI00694851;IPI00695331;IPI00699355;IPI00701698;IPI00703731;IPI00703776;IPI00705463;IPI00716158;IPI00842934;IPI01018577	Biological Process	Regulation of transcription from RNA polymerase II promoter	12	14	211	0.0288
GO:0009062	IPI00694312;IPI00699355;IPI00702650;IPI00842934	Biological Process	Fatty acid catabolic process	9	4	211	0.0295

GO:0051093	IPI00691963;IPI00692468;IPI00696930;IPI00697184;IPI00698039;IPI00703753;IPI00707101;IPI00716121;IPI00717119	Biological Process	Negative regulation of developmental process	5	9	211	0.0306
GO:0006955	IPI00686841;IPI00687842;IPI00690094;IPI00694751;IPI00695142;IPI00695506;IPI00695965;IPI00698993;IPI00701698;IPI00703753;IPI00713757	Biological Process	Immune response	4	11	211	0.0311
GO:0002698	IPI00693338;IPI00701698;IPI00871133	Biological Process	Negative regulation of immune effector process	6	3	211	0.0322
GO:0002920	IPI00693338;IPI00701698;IPI00871133	Biological Process	Regulation of humoral immune response	6	3	211	0.0322
GO:0030239	IPI00688489;IPI00694204;IPI00698900	Biological Process	Myofibril assembly	8	3	211	0.0322
GO:0051291	IPI00707718;IPI00713573;IPI00837992	Biological Process	Protein heterooligomerization	9	3	211	0.0322
GO:0001976	IPI00692468;IPI00697196	Biological Process	Neurological system process involved in regulation of systemic arterial blood pressure	10	2	211	0.0324
GO:0009583	IPI00697184;IPI00703776	Biological Process	Detection of light stimulus	6	2	211	0.0324
GO:0031063	IPI00690446;IPI00842934	Biological Process	Regulation of histone deacetylation	10	2	211	0.0324
GO:0031065	IPI00690446;IPI00842934	Biological Process	Positive regulation of histone deacetylation	11	2	211	0.0324
GO:0035634	IPI00695965;IPI00944429	Biological Process	Response to stilbenoid	8	2	211	0.0324
GO:0043616	IPI00699355;IPI00715354	Biological Process	Keratinocyte proliferation	6	2	211	0.0324
GO:0055012	IPI00689750;IPI00692676	Biological Process	Ventricular cardiac muscle cell differentiation	9	2	211	0.0324
GO:0055015	IPI00689750;IPI00692676	Biological Process	Ventricular cardiac muscle cell development	10	2	211	0.0324

GO:0060347	IPI00692676;IPI00697184	Biological Process	Heart trabecula formation	11	2	211	0.0324
GO:0080184	IPI00695965;IPI00944429	Biological Process	Response to phenylpropanoid	7	2	211	0.0324
GO:0090312	IPI00690446;IPI00842934	Biological Process	Positive regulation of protein deacetylation	11	2	211	0.0324
GO:0022607	IPI00686225;IPI00687539;IPI00687657;IPI00688489;IPI00688921;IPI00690160;IPI00691963;IPI00692468;IPI00694204;IPI00695506;IPI00695965;IPI00698039;IPI00698900;IPI00701698;IPI00703731;IPI00703776;IPI00705000;IPI00707718;IPI00713573;IPI00714673;IPI00715339;IPI00721270;IPI00728768;IPI00837992;IPI01018577	Biological Process	Cellular component assembly	5	25	211	0.0330
GO:0007155	IPI00687372;IPI00689035;IPI00689760;IPI00690160;IPI00693338;IPI00694751;IPI00695965;IPI00699355;IPI00700542;IPI00707587;IPI00714673;IPI00715339;IPI00840588	Biological Process	Cell adhesion	4	13	211	0.0335

GO:0022610	IPI00687372;IPI00689035;IPI00689760;IPI00690160;IPI00693338;IPI00694751;IPI00695965;IPI00699355;IPI00700542;IPI00707587;IPI00714673;IPI00715339;IPI00840588	Biological Process	Biological adhesion	3	13	211	0.0335
GO:0061061	IPI00688489;IPI00689750;IPI00690094;IPI00692676;IPI00694204;IPI00694504;IPI00698900;IPI00702028	Biological Process	Muscle structure development	5	8	211	0.0347
GO:0002682	IPI00686841;IPI00690094;IPI00690160;IPI00691212;IPI00693338;IPI00697184;IPI00697757;IPI00698039;IPI00700789;IPI00701698;IPI00706141;IPI00713757;IPI00871133;IPI00912603	Biological Process	Regulation of immune system process	4	14	211	0.0359
GO:0045595	IPI00687539;IPI00687601;IPI00689228;IPI00691963;IPI00692468;IPI00693338;IPI00696930;IPI00698039;IPI00699355;IPI00701698;IPI00703753;IPI00706141;IPI00716121;IPI00842934	Biological Process	Regulation of cell differentiation	7	14	211	0.0359
GO:0051235	IPI00687842;IPI00695666;IPI00701223;IPI00703753;IPI00707359	Biological Process	Maintenance of location	4	5	211	0.0365

GO:0000122	IPI00691963;IPI00692676;IPI00699355;IPI00701698;IPI00703731;IPI00842934;IPI01018577	Biological Process	Negative regulation of transcription from RNA polymerase II promoter	12	7	211	0.0389
GO:0018149	IPI00688608;IPI00697081;IPI00714673	Biological Process	Peptide cross-linking	8	3	211	0.0400
GO:0035051	IPI00688489;IPI00689750;IPI00692676	Biological Process	Cardiocyte differentiation	7	3	211	0.0400
GO:0050777	IPI00693338;IPI00697757;IPI00871133	Biological Process	Negative regulation of immune response	6	3	211	0.0400
GO:0055007	IPI00688489;IPI00689750;IPI00692676	Biological Process	Cardiac muscle cell differentiation	8	3	211	0.0400
GO:0006816	IPI00688651;IPI00690160;IPI00693338;IPI00706458	Biological Process	Calcium ion transport	9	4	211	0.0406
GO:0032868	IPI00697184;IPI00702650;IPI00703753;IPI00842934	Biological Process	Response to insulin	7	4	211	0.0406
GO:0048589	IPI00687601;IPI00699355;IPI00718311;IPI01018577	Biological Process	Developmental growth	5	4	211	0.0406
GO:0070838	IPI00688651;IPI00690160;IPI00693338;IPI00706458	Biological Process	Divalent metal ion transport	8	4	211	0.0406
GO:0072329	IPI00694312;IPI00699355;IPI00702650;IPI00842934	Biological Process	Monocarboxylic acid catabolic process	8	4	211	0.0406
GO:0072511	IPI00688651;IPI00690160;IPI00693338;IPI00706458	Biological Process	Divalent inorganic cation transport	7	4	211	0.0406
GO:0050727	IPI00694751;IPI00697757;IPI00701698;IPI00707101;IPI00871133	Biological Process	Regulation of inflammatory response	7	5	211	0.0463

GO:0051241	IPI00697184;IPI00701166;IPI00701698;IPI00703753;IPI00703776;IPI00707101;IPI00717119	Biological Process	Negative regulation of multicellular organismal process	5	7	211	0.0463
GO:0019509	IPI00694739;IPI00883375	Biological Process	L-methionine biosynthetic process from methylthioadenosine	12	2	211	0.0467
GO:0030279	IPI00703753;IPI00707101	Biological Process	Negative regulation of ossification	7	2	211	0.0467
GO:0031953	IPI00693338;IPI00703753	Biological Process	Negative regulation of protein autophosphorylation	11	2	211	0.0467
GO:0032330	IPI00696930;IPI00706141	Biological Process	Regulation of chondrocyte differentiation	8	2	211	0.0467
GO:0035065	IPI00690446;IPI00703776	Biological Process	Regulation of histone acetylation	10	2	211	0.0467
GO:0042572	IPI00692627;IPI00697184	Biological Process	Retinol metabolic process	6	2	211	0.0467
GO:0043102	IPI00694739;IPI00883375	Biological Process	Amino acid salvage	8	2	211	0.0467
GO:0045599	IPI00692468;IPI00703753	Biological Process	Negative regulation of fat cell differentiation	9	2	211	0.0467
GO:0045652	IPI00698039;IPI00706141	Biological Process	Regulation of megakaryocyte differentiation	9	2	211	0.0467
GO:0050766	IPI00691963;IPI00707101	Biological Process	Positive regulation of phagocytosis	8	2	211	0.0467
GO:0060042	IPI00697184;IPI00703547	Biological Process	Retina morphogenesis in camera-type eye	12	2	211	0.0467
GO:0061384	IPI00692676;IPI00697184	Biological Process	Heart trabecula morphogenesis	6	2	211	0.0467
GO:0070168	IPI00707101;IPI00717119	Biological Process	Negative regulation of biomineral tissue development	10	2	211	0.0467
GO:0071265	IPI00694739;IPI00883375	Biological Process	L-methionine biosynthetic process	10	2	211	0.0467
GO:0071267	IPI00694739;IPI00883375	Biological Process	L-methionine salvage	11	2	211	0.0467
GO:0071695	IPI00699355;IPI00707359	Biological Process	Anatomical structure maturation	6	2	211	0.0467
GO:0090311	IPI00690446;IPI00842934	Biological Process	Regulation of protein deacetylation	10	2	211	0.0467
GO:1901983	IPI00690446;IPI00703776	Biological Process	Regulation of protein acetylation	10	2	211	0.0467
GO:2000756	IPI00690446;IPI00703776	Biological Process	Regulation of peptidyl-lysine acetylation	9	2	211	0.0467

GO:0048738	IPI00688489;IPI00689750;IPI00692676;IPI00697184	Biological Process	Cardiac muscle tissue development	9	4	211	0.0469
GO:0051494	IPI00688921;IPI00690160;IPI00706141;IPI00904732	Biological Process	Ngative regulation of cytoskeleton organization	7	4	211	0.0469
GO:0048519	IPI00687539;IPI00687601;IPI00687842;IPI00688921;IPI00689228;IPI00690001;IPI00690160;IPI00690446;IPI00691963;IPI00692468;IPI00692676;IPI00693338;IPI00694739;IPI00695508;IPI00695965;IPI00696930;IPI00697184;IPI00697757;IPI00698039;IPI00699355;IPI00700789;IPI00701166;IPI00701698;IPI00703731;IPI00703753;IPI00703776;IPI00705660;IPI00706141;IPI00707101;IPI00707359;IPI00707587;IPI00708921;IPI00715354;IPI00716121;IPI00717119;IPI00842934;IPI00871133;IPI00904732;IPI00912603;IPI01018577	Biological Process	Negative regulation of biological process	5	40	211	0.0470

GO:0032502	IPI00686966;IPI00687372;IPI00687539;IPI00687601;IPI00688489;IPI00689035;IPI00689228;IPI00689750;IPI00690094;IPI00690446;IPI00691963;IPI00692468;IPI00692676;IPI00693338;IPI00694204;IPI00694504;IPI00695666;IPI00695890;IPI00697184;IPI00698900;IPI00699355;IPI00700295;IPI00701790;IPI00702028;IPI00702620;IPI00703547;IPI00703854;IPI00706942;IPI00707359;IPI00710385;IPI00712671;IPI00713642;IPI00714673;IPI00715354;IPI00716121;IPI00717119;IPI00718311;IPI00721270;IPI00730144;IPI00840588;IPI00847093;IP	Biological Process	Developmental process	3	44	211	0.0485
GO:0007159	IPI00693338;IPI00694751;IPI00695965	Biological Process	Leukocyte cell-cell adhesion	7	3	211	0.0487
GO:0007569	IPI00691963;IPI00710385;IPI00713642	Biological Process	Cell aging	5	3	211	0.0487
GO:0019319	IPI00689323;IPI00697184;IPI00706942	Biological Process	Hexose biosynthetic process	8	3	211	0.0487
GO:0035150	IPI00692468;IPI00697196;IPI00701166	Biological Process	Regulation of tube size	6	3	211	0.0487
GO:0042445	IPI00686601;IPI00692627;IPI00697184	Biological Process	Hormone metabolic process	4	3	211	0.0487
GO:0050880	IPI00692468;IPI00697196;IPI00701166	Biological Process	Regulation of blood vessel size	8	3	211	0.0487
GO:0051606	IPI00692468;IPI00697184;IPI00703776	Biological Process	Detection of stimulus	4	3	211	0.0487

GO:0010639	IPI00688921;IPI00690160;IPI00690446;IPI00706141;IPI00904732	Biological Process	Negative regulation of organelle organization	5	5	211	0.0517
GO:0007568	IPI00691963;IPI00692468;IPI00710385;IPI00713642	Biological Process	Aging	5	4	211	0.0537
GO:0043010	IPI00689228;IPI00697184;IPI00703547;IPI00847093	Biological Process	Camera-type eye development	10	4	211	0.0537
GO:0010033	IPI00686966;IPI00687539;IPI00687625;IPI00690160;IPI00692676;IPI00694204;IPI00695965;IPI00697184;IPI00697196;IPI00700547;IPI00702650;IPI00703547;IPI00703753;IPI00708921;IPI00713573;IPI00718311;IPI00742596;IPI00842934;IPI00944429	Biological Process	Response to organic substance	5	19	211	0.0568
GO:0032879	IPI00686601;IPI00687539;IPI00687601;IPI00688651;IPI00689750;IPI00690160;IPI00691963;IPI00693338;IPI00695506;IPI00695508;IPI00695965;IPI00697184;IPI00700789;IPI00701698;IPI00703753;IPI00703776;IPI00704977;IPI00706141;IPI00707101;IPI00707359;IPI00715354	Biological Process	Regulation of localization	4	21	211	0.0571

GO:0031058	IPI00690446;IPI00703776;IPI00842934	Biological Process	Positive regulation of histone modification	10	3	211	0.0582
GO:2001252	IPI00690446;IPI00703776;IPI00842934	Biological Process	Positive regulation of chromosome organization	7	3	211	0.0582
GO:0007626	IPI00686966;IPI00692468;IPI00703547;IPI00716843	Biological Process	Locomotory behavior	4	4	211	0.0610
GO:0034728	IPI00698039;IPI00703854;IPI00705000;IPI00728768	Biological Process	Nucleosome organization	7	4	211	0.0610
GO:0043434	IPI00697184;IPI00702650;IPI00703753;IPI00842934	Biological Process	Response to peptide hormone	6	4	211	0.0610
GO:0071824	IPI00698039;IPI00703854;IPI00705000;IPI00728768	Biological Process	Protein-DNA complex subunit organization	6	4	211	0.0610
GO:0002637	IPI00693338;IPI00697184	Biological Process	Regulation of immunoglobulin production	7	2	211	0.0628
GO:0002793	IPI00697184;IPI00703776	Biological Process	Positive regulation of peptide secretion	9	2	211	0.0628
GO:0010977	IPI00687539;IPI00689228	Biological Process	Negative regulation of neuron projection development	8	2	211	0.0628
GO:0030212	IPI00702700;IPI00715287	Biological Process	Hyaluronan metabolic process	9	2	211	0.0628
GO:0032024	IPI00697184;IPI00703776	Biological Process	Positive regulation of insulin secretion	11	2	211	0.0628
GO:0042311	IPI00692468;IPI00701166	Biological Process	Vasodilation	9	2	211	0.0628
GO:0043647	IPI00686601;IPI00692819	Biological Process	Inositol phosphate metabolic process	7	2	211	0.0628
GO:0045214	IPI00694204;IPI00698900	Biological Process	Sarcomere organization	9	2	211	0.0628
GO:0046113	IPI00687539;IPI00716555	Biological Process	Nucleobase catabolic process	9	2	211	0.0628
GO:0048638	IPI00687601;IPI00697184	Biological Process	Regulation of developmental growth	6	2	211	0.0628
GO:0050858	IPI00700789;IPI00912603	Biological Process	Negative regulation of antigen receptor-mediated signaling pathway	9	2	211	0.0628
GO:0050860	IPI00700789;IPI00912603	Biological Process	Negative regulation of T cell receptor signaling pathway	10	2	211	0.0628
GO:0060343	IPI00692676;IPI00697184	Biological Process	Trabecula formation	9	2	211	0.0628

GO:0061035	IPI00696930;IPI00706141	Biological Process	Regulation of cartilage development	9	2	211	0.0628
GO:0070167	IPI00707101;IPI00717119	Biological Process	Regulation of biomineral tissue development	9	2	211	0.0628
GO:0070208	IPI00713573;IPI00837992	Biological Process	Protein heterotrimerization	10	2	211	0.0628
GO:0090277	IPI00697184;IPI00703776	Biological Process	Positive regulation of peptide hormone secretion	11	2	211	0.0628
GO:2000241	IPI00701698;IPI00716121	Biological Process	Regulation of reproductive process	4	2	211	0.0628
GO:0051259	IPI00686225;IPI00687539;IPI00687657;IPI00692468;IPI00707718;IPI00713573;IPI00837992;IPI01018577	Biological Process	Protein oligomerization	8	8	211	0.0632
GO:0003013	IPI00688489;IPI00688651;IPI00692468;IPI00697196;IPI00701166	Biological Process	Circulatory system process	6	5	211	0.0636
GO:0008015	IPI00688489;IPI00688651;IPI00692468;IPI00697196;IPI00701166	Biological Process	Blood circulation	7	5	211	0.0636
GO:0051130	IPI00687539;IPI00687601;IPI00690446;IPI00691963;IPI00701698;IPI00703776;IPI00704728;IPI00706141;IPI00707101;IPI00842934	Biological Process	Positive regulation of cellular component organization	6	10	211	0.0669
GO:0033043	IPI00688921;IPI00690160;IPI00690446;IPI00700789;IPI00701698;IPI00703776;IPI00704977;IPI00706141;IPI00711750;IPI00842934;IPI00904732	Biological Process	Regulation of organelle organization	5	11	211	0.0672
GO:0003018	IPI00692468;IPI00697196;IPI00701166	Biological Process	Vascular process in circulatory system	7	3	211	0.0686

GO:0010817	IPI00686601;IPI00692627;IPI00697184	Biological Process	Regulation of hormone levels	5	3	211	0.0686
GO:0031348	IPI00697757;IPI00701698;IPI00871133	Biological Process	Negative regulation of defense response	7	3	211	0.0686
GO:0046364	IPI00689323;IPI00697184;IPI00706942	Biological Process	Monosaccharide biosynthetic process	7	3	211	0.0686
GO:0001890	IPI00694204;IPI00699355;IPI00700295;IPI01018577	Biological Process	Placenta development	6	4	211	0.0688
GO:0016311	IPI00690308;IPI00692819;IPI00693338;IPI00699798;IPI00700789	Biological Process	Dephosphorylation	7	5	211	0.0700
GO:0031328	IPI00689750;IPI00691963;IPI00692468;IPI00694851;IPI00695965;IPI00699355;IPI00701698;IPI00703776;IPI00704728;IPI00705463;IPI00716158;IPI00842934;IPI00912603;IPI01018577	Biological Process	Positive regulation of cellular biosynthetic process	7	14	211	0.0710

GO:0016043	IPI00686225;IPI00686601;IPI00686966;IPI00687372;IPI00687539;IPI00687601;IPI00687657;IPI00688489;IPI00688921;IPI00689228;IPI00689750;IPI00690160;IPI00690446;IPI00691963;IPI00692468;IPI00694204;IPI00694504;IPI00695506;IPI00695666;IPI00695890;IPI00695965;IPI00698039;IPI00698900;IPI00701223;IPI00701698;IPI00703731;IPI00703776;IPI00703854;IPI00705000;IPI00705941;IPI00706141;IPI00707359;IPI00707587;IPI00707718;IPI00708921;IPI00709922;IPI00711750;IPI00713573;IPI00714673;IPI00715339;IPI00715354;IP	Biological Process	Cellular component organization	4	49	211	0.0736
GO:0006954	IPI00687625;IPI00691212;IPI00694751;IPI00701166;IPI00707101	Biological Process	Inflammatory response	6	5	211	0.0768
GO:0051651	IPI00687842;IPI00695666;IPI00701223;IPI00707359	Biological Process	Maintenance of location in cell	5	4	211	0.0771
GO:1901652	IPI00697184;IPI00702650;IPI00703753;IPI00842934	Biological Process	Response to peptide	6	4	211	0.0771
GO:0001503	IPI00692676;IPI00707101;IPI00717119	Biological Process	Ossification	5	3	211	0.0797
GO:0006635	IPI00694312;IPI00699355;IPI00702650	Biological Process	Fatty acid beta-oxidation	10	3	211	0.0797
GO:0018209	IPI00688608;IPI00688651;IPI00697081	Biological Process	Peptidyl-serine modification	9	3	211	0.0797

GO:0042129	IPI00690160;IPI00693338;IPI00912603	Biological Process	Regulation of T cell proliferation	8	3	211	0.0797
GO:0051017	IPI00687539;IPI00695506;IPI00703776	Biological Process	Actin filament bundle assembly	9	3	211	0.0797
GO:0061572	IPI00687539;IPI00695506;IPI00703776	Biological Process	Actin filament bundle organization	8	3	211	0.0797
GO:0006950	IPI00686183;IPI00686841;IPI00687539;IPI00687625;IPI00689750;IPI00691212;IPI00691669;IPI00691963;IPI00692468;IPI00693338;IPI00694504;IPI00694751;IPI00695965;IPI00697196;IPI00699355;IPI00700547;IPI00701166;IPI00701698;IPI00707101;IPI00712677;IPI00713757;IPI00714673;IPI00715354;IPI00718757;IPI00727017;IPI00730144;IPI00867179;IPI00867339;IPI01018577	Biological Process	Response to stress	4	29	211	0.0799
GO:0006029	IPI00688608;IPI00697081	Biological Process	Proteoglycan metabolic process	7	2	211	0.0804
GO:0006555	IPI00694739;IPI00883375	Biological Process	Methionine metabolic process	8	2	211	0.0804
GO:0007586	IPI00695965;IPI00697184	Biological Process	Digestion	5	2	211	0.0804
GO:0009086	IPI00694739;IPI00883375	Biological Process	Methionine biosynthetic process	9	2	211	0.0804
GO:0030449	IPI00701698;IPI00871133	Biological Process	Regulation of complement activation	6	2	211	0.0804
GO:0034394	IPI00714481;IPI00715339	Biological Process	Protein localization to cell surface	7	2	211	0.0804

GO:0034453	IPI00701223;IPI00707359	Biological Process	Microtubule anchoring	7	2	211	0.0804
GO:0045103	IPI00689228;IPI00721270	Biological Process	Intermediate filament-based process	5	2	211	0.0804
GO:0045104	IPI00689228;IPI00721270	Biological Process	Intermediate filament cytoskeleton organization	6	2	211	0.0804
GO:0045109	IPI00689228;IPI00721270	Biological Process	Intermediate filament organization	7	2	211	0.0804
GO:0045940	IPI00686601;IPI00695965	Biological Process	Positive regulation of steroid metabolic process	8	2	211	0.0804
GO:0048147	IPI00687842;IPI00692468	Biological Process	Negative regulation of fibroblast proliferation	6	2	211	0.0804
GO:0050856	IPI00700789;IPI00912603	Biological Process	Regulation of T cell receptor signaling pathway	9	2	211	0.0804
GO:0061383	IPI00692676;IPI00697184	Biological Process	Trabecula morphogenesis	5	2	211	0.0804
GO:2000257	IPI00701698;IPI00871133	Biological Process	Regulation of protein activation cascade	7	2	211	0.0804
GO:0048468	IPI00686966;IPI00687372;IPI00687601;IPI00688489;IPI00689228;IPI00689750;IPI00692468;IPI00692676;IPI00693338;IPI00694204;IPI00694504;IPI00698900;IPI00707359;IPI00714673;IPI01018577	Biological Process	Cell development	7	15	211	0.0817

GO:0048856	IPI00686966;IPI00687372;IPI00687539;IPI00687601;IPI00688489;IPI00689035;IPI00689228;IPI00689750;IPI00690094;IPI00691963;IPI00692468;IPI00692676;IPI00693338;IPI00694204;IPI00694504;IPI00695666;IPI00695890;IPI00697184;IPI00698900;IPI00699355;IPI00700295;IPI00701790;IPI00702028;IPI00702620;IPI00703547;IPI00703854;IPI00706942;IPI00707359;IPI00712671;IPI00714673;IPI00715354;IPI00716121;IPI00717119;IPI00718311;IPI00721270;IPI00730144;IPI00840588;IPI00847093;IPI01018577	Biological Process	Anatomical structure development	4	39	211	0.0830
GO:0044085	IPI00686225;IPI00687539;IPI00687657;IPI00688489;IPI00688921;IPI00690160;IPI00691963;IPI00692468;IPI00694204;IPI00695506;IPI00695965;IPI00698039;IPI00698900;IPI00701698;IPI00703731;IPI00703776;IPI00705000;IPI00707718;IPI00713573;IPI00714673;IPI00715339;IPI00721270;IPI00728768;IPI00837992;IPI01018577	Biological Process	Cellular component biogenesis	4	25	211	0.0836

GO:0032271	IPI00688921;IPI00690160;IPI00706141;IPI00711750;IPI00904732	Biological Process	Regulation of protein polymerization	7	5	211	0.0840
GO:0009891	IPI00689750;IPI00691963;IPI00692468;IPI00694851;IPI00695965;IPI00699355;IPI00701698;IPI00703776;IPI00704728;IPI00705463;IPI00716158;IPI00842934;IPI00912603;IPI01018577	Biological Process	Positive regulation of biosynthetic process	6	14	211	0.0842
GO:0030154	IPI00686966;IPI00687372;IPI00687539;IPI00687601;IPI00688489;IPI00689228;IPI00689750;IPI00690094;IPI00690446;IPI00691963;IPI00692468;IPI00692676;IPI00693338;IPI00694204;IPI00694504;IPI00695666;IPI00698900;IPI00699355;IPI00707359;IPI00714673;IPI00715354;IPI00716121;IPI00718311;IPI00721270;IPI00871133;IPI01018577	Biological Process	Cell differentiation	6	26	211	0.0846
GO:0009725	IPI00692676;IPI00694204;IPI00697184;IPI00702650;IPI00703753;IPI00842934	Biological Process	Response to hormone	5	6	211	0.0856
GO:0001654	IPI00689228;IPI00697184;IPI00703547;IPI00847093	Biological Process	Eye development	9	4	211	0.0859

GO:0044707	IPI00686966;IPI00687372;IPI00687539;IPI00687601;IPI00688489;IPI00688651;IPI00689035;IPI00689228;IPI00689750;IPI00690094;IPI00690160;IPI00691963;IPI00692468;IPI00692676;IPI00693338;IPI00693628;IPI00694204;IPI00694504;IPI00695666;IPI00695965;IPI00697184;IPI00697196;IPI00699355;IPI00700295;IPI00701166;IPI00701790;IPI00702028;IPI00703547;IPI00703753;IPI00703854;IPI00706942;IPI00707101;IPI00707359;IPI00712671;IPI00715354;IPI00716121;IPI00717119;IPI00718311;IPI00730144;IPI00839134;IPI00840588;IP	Biological Process	Single-multicellular organism process	4	45	211	0.0861

GO:0050896	IPI00685792;IPI00686183;IPI00686225;IPI00686841;IPI00686966;IPI00687372;IPI00687539;IPI00687625;IPI00687842;IPI00689750;IPI00690094;IPI00690160;IPI00691212;IPI00691669;IPI00691963;IPI00692468;IPI00692676;IPI00692819;IPI00693338;IPI00694204;IPI00694504;IPI00694751;IPI00694851;IPI00695142;IPI00695506;IPI00695508;IPI00695965;IPI00697184;IPI00697196;IPI00698993;IPI00699355;IPI00700547;IPI00701166;IPI00701698;IPI00702650;IPI00703547;IPI00703731;IPI00703753;IPI00703776;IPI00705941;IPI00707101;IP	Biological Process	Response to stimulus	3	59	211	0.0897
GO:0051129	IPI00687539;IPI00688921;IPI00689228;IPI00690160;IPI00690446;IPI00706141;IPI00904732	Biological Process	Negative regulation of cellular component organization	6	7	211	0.0901
GO:0006334	IPI00698039;IPI00705000;IPI00728768	Biological Process	Nucleosome assembly	12	3	211	0.0915
GO:0006720	IPI00692627;IPI00697184;IPI00944429	Biological Process	Isoprenoid metabolic process	6	3	211	0.0915
GO:0009112	IPI00686225;IPI00687539;IPI00716555	Biological Process	Nucleobase metabolic process	8	3	211	0.0915
GO:0031032	IPI00688489;IPI00694204;IPI00698900	Biological Process	Actomyosin structure organization	7	3	211	0.0915
GO:0031056	IPI00690446;IPI00703776;IPI00842934	Biological Process	Regulation of histone modification	9	3	211	0.0915

GO:0032272	IPI00688921;IPI00706141;IPI00904732	Biological Process	Negative regulation of protein polymerization	8	3	211	0.0915
GO:0065004	IPI00698039;IPI00705000;IPI00728768	Biological Process	Protein-DNA complex assembly	8	3	211	0.0915
GO:1902275	IPI00690446;IPI00703776;IPI00842934	Biological Process	Regulation of chromatin organization	7	3	211	0.0915
GO:0070925	IPI00688489;IPI00690160;IPI00694204;IPI00698900;IPI00701698	Biological Process	Organelle assembly	5	5	211	0.0915
GO:0006470	IPI00690308;IPI00693338;IPI00699798;IPI00700789	Biological Process	Protein dephosphorylation	8	4	211	0.0951
GO:0051173	IPI00686225;IPI00689750;IPI00691963;IPI00692468;IPI00693338;IPI00694851;IPI00699355;IPI00703776;IPI00705463;IPI00716158;IPI00717759;IPI00842934;IPI01018577	Biological Process	Positive regulation of nitrogen compound metabolic process	6	13	211	0.0982
GO:0006913	IPI00689750;IPI00691963;IPI00692911;IPI00699002;IPI00705941;IPI00707359	Biological Process	Nucleocytoplasmic transport	8	6	211	0.0989
GO:0051169	IPI00689750;IPI00691963;IPI00692911;IPI00699002;IPI00705941;IPI00707359	Biological Process	Nuclear transport	7	6	211	0.0989
GO:0030001	IPI00687842;IPI00688651;IPI00690160;IPI00693338;IPI00706458	Biological Process	Metal ion transport	7	5	211	0.0994
GO:0000097	IPI00694739;IPI00883375	Biological Process	Sulfur amino acid biosynthetic process	8	2	211	0.0994
GO:0000303	IPI00692468;IPI00695965	Biological Process	Response to superoxide	7	2	211	0.0994

GO:0001523	IPI00692627;IPI00697184	Biological Process	Retinoid metabolic process	9	2	211	0.0994
GO:0002673	IPI00701698;IPI00871133	Biological Process	Regulation of acute inflammatory response	8	2	211	0.0994
GO:0009226	IPI00689323;IPI00712671	Biological Process	Nucleotide-sugar biosynthetic process	10	2	211	0.0994
GO:0009582	IPI00697184;IPI00703776	Biological Process	Detection of abiotic stimulus	5	2	211	0.0994
GO:0016101	IPI00692627;IPI00697184	Biological Process	Diterpenoid metabolic process	8	2	211	0.0994
GO:0019430	IPI00692468;IPI00695965	Biological Process	Removal of superoxide radicals	7	2	211	0.0994
GO:0030850	IPI00694504;IPI00718311	Biological Process	Prostate gland development	6	2	211	0.0994
GO:0030888	IPI00693338;IPI00912603	Biological Process	Regulation of B cell proliferation	8	2	211	0.0994
GO:0031952	IPI00693338;IPI00703753	Biological Process	Regulation of protein autophosphorylation	10	2	211	0.0994
GO:0035335	IPI00690308;IPI00693338	Biological Process	Peptidyl-tyrosine dephosphorylation	9	2	211	0.0994
GO:0046173	IPI00686601;IPI00692819	Biological Process	Polyol biosynthetic process	8	2	211	0.0994
GO:0046887	IPI00697184;IPI00703776	Biological Process	Positive regulation of hormone secretion	10	2	211	0.0994
GO:0050673	IPI00699355;IPI00715354	Biological Process	Epithelial cell proliferation	5	2	211	0.0994
GO:0071451	IPI00692468;IPI00695965	Biological Process	Cellular response to superoxide	9	2	211	0.0994
GO:0051493	IPI00688921;IPI00690160;IPI00700789;IPI00704977;IPI00706141;IPI00711750;IPI00904732	Biological Process	Regulation of cytoskeleton organization	6	7	211	0.1022
GO:0008285	IPI00687842;IPI00692468;IPI00697184;IPI00699355;IPI00706141;IPI00707587;IPI00715354;IPI00716121;IPI00912603	Biological Process	Negative regulation of cell proliferation	5	9	211	0.1028
GO:0010720	IPI00687601;IPI00701698;IPI00716121	Biological Process	Positive regulation of cell development	9	3	211	0.1040

GO:0019882	IPI00691963;IPI00695176;IPI00698993	Biological Process	Antigen processing and presentation	4	3	211	0.1040
GO:0031497	IPI00698039;IPI00705000;IPI00728768	Biological Process	Chromatin assembly	11	3	211	0.1040
GO:0043244	IPI00688921;IPI00704728;IPI00706141	Biological Process	Regulation of protein complex disassembly	8	3	211	0.1040
GO:0051289	IPI00686225;IPI00687657;IPI00692468	Biological Process	Protein homotetramerization	10	3	211	0.1040
GO:0010942	IPI00686966;IPI00689325;IPI00693338;IPI00701698;IPI00703731;IPI00706141	Biological Process	Positive regulation of cell death	7	6	211	0.1059
GO:0016042	IPI00694312;IPI00695965;IPI00699355;IPI00702650;IPI00842934	Biological Process	Lipid catabolic process	6	5	211	0.1075
GO:0032535	IPI00687601;IPI00688921;IPI00690160;IPI00706141;IPI00711750	Biological Process	Regulation of cellular component size	5	5	211	0.1075
GO:0042221	IPI00685792;IPI00686966;IPI00687372;IPI00687539;IPI00687625;IPI00689750;IPI00690160;IPI00692468;IPI00692676;IPI00694204;IPI00694751;IPI00695508;IPI00695965;IPI00697184;IPI00697196;IPI00700547;IPI00702650;IPI00703547;IPI00703753;IPI00708921;IPI00713573;IPI00718311;IPI00742596;IPI00842934;IPI00944429	Biological Process	Response to chemical	4	25	211	0.1076

GO:0051301	IPI00699798;IPI00699803;IPI00701223;IPI00703380;IPI00705941;IPI00707718;IPI00904732	Biological Process	Cell division	5	7	211	0.1086
GO:2000026	IPI00687539;IPI00687601;IPI00689228;IPI00691963;IPI00693338;IPI00696930;IPI00697184;IPI00698039;IPI00701698;IPI00706141;IPI00707101;IPI00716121;IPI00717119	Biological Process	Regulation of multicellular organismal development	6	13	211	0.1107
GO:0048869	IPI00686966;IPI00687372;IPI00687539;IPI00687601;IPI00688489;IPI00689228;IPI00689750;IPI00690094;IPI00690446;IPI00691963;IPI00692468;IPI00692676;IPI00693338;IPI00694204;IPI00694504;IPI00695666;IPI00695890;IPI00698900;IPI00699355;IPI00707359;IPI00714673;IPI00715354;IPI00716121;IPI00718311;IPI00721270;IPI00871133;IPI01018577	Biological Process	Cellular developmental process	5	27	211	0.1114
GO:0031347	IPI00686841;IPI00694751;IPI00697757;IPI00701698;IPI00707101;IPI00871133	Biological Process	Regulation of defense response	6	6	211	0.1132

GO:0001655	IPI00694504;IPI00697184;IPI00703547;IPI00718311	Biological Process	Urogenital system development	7	4	211	0.1149
GO:0044242	IPI00694312;IPI00699355;IPI00702650;IPI00842934	Biological Process	Cellular lipid catabolic process	6	4	211	0.1149
GO:0050863	IPI00690094;IPI00690160;IPI00693338;IPI00912603	Biological Process	Regulation of T cell activation	7	4	211	0.1149
GO:0006915	IPI00686966;IPI00688489;IPI00692468;IPI00694739;IPI00694751;IPI00699355;IPI00701698;IPI00703129;IPI00704728;IPI00704835;IPI00708921;IPI00715354;IPI00912603	Biological Process	Apoptotic process	7	13	211	0.1150
GO:0010927	IPI00688489;IPI00694204;IPI00698900	Biological Process	Cellular component assembly involved in morphogenesis	7	3	211	0.1172
GO:0019395	IPI00694312;IPI00699355;IPI00702650	Biological Process	Fatty acid oxidation	10	3	211	0.1172
GO:0031333	IPI00688921;IPI00706141;IPI00904732	Biological Process	Negative regulation of protein complex assembly	7	3	211	0.1172
GO:0033044	IPI00690446;IPI00703776;IPI00842934	Biological Process	Regulation of chromosome organization	6	3	211	0.1172
GO:0034440	IPI00694312;IPI00699355;IPI00702650	Biological Process	Lipid oxidation	7	3	211	0.1172
GO:0000394	IPI00700295	Biological Process	RNA splicing, via endonucleolytic cleavage and ligation	11	1	211	0.1176
GO:0001315	IPI00692468	Biological Process	Age-dependent response to reactive oxygen species	8	1	211	0.1176
GO:0001911	IPI00693338	Biological Process	Negative regulation of leukocyte mediated cytotoxicity	6	1	211	0.1176
GO:0001915	IPI00693338	Biological Process	Negative regulation of T cell mediated cytotoxicity	7	1	211	0.1176
GO:0001980	IPI00697196	Biological Process	Regulation of systemic arterial blood pressure by ischemic conditions	11	1	211	0.1176

GO:0002014	IPI00697196	Biological Process	Vasoconstriction of artery involved in ischemic response to lowering of systemic arterial blood pressure	10	1	211	0.1176
GO:0002029	IPI00703776	Biological Process	Desensitization of G-protein coupled receptor protein signaling pathway	11	1	211	0.1176
GO:0002031	IPI00703776	Biological Process	G-protein coupled receptor internalization	8	1	211	0.1176
GO:0002227	IPI00695965	Biological Process	Innate immune response in mucosa	7	1	211	0.1176
GO:0002378	IPI00693338	Biological Process	Immunoglobulin biosynthetic process	6	1	211	0.1176
GO:0002386	IPI00695142	Biological Process	Immune response in mucosal-associated lymphoid tissue	7	1	211	0.1176
GO:0002396	IPI00691963	Biological Process	MHC protein complex assembly	9	1	211	0.1176
GO:0002397	IPI00691963	Biological Process	MHC class I protein complex assembly	10	1	211	0.1176
GO:0002404	IPI00695142	Biological Process	Antigen sampling in mucosal-associated lymphoid tissue	4	1	211	0.1176
GO:0002406	IPI00695142	Biological Process	Antigen sampling by M cells in mucosal-associated lymphoid tissue	5	1	211	0.1176
GO:0002412	IPI00695142	Biological Process	Antigen transcytosis by M cells in mucosal-associated lymphoid tissue	6	1	211	0.1176
GO:0002501	IPI00691963	Biological Process	Peptide antigen assembly with MHC protein complex	6	1	211	0.1176
GO:0002502	IPI00691963	Biological Process	Peptide antigen assembly with MHC class I protein complex	7	1	211	0.1176
GO:0002710	IPI00693338	Biological Process	Negative regulation of T cell mediated immunity	9	1	211	0.1176
GO:0003032	IPI00692468	Biological Process	Detection of oxygen	6	1	211	0.1176
GO:0003068	IPI00692468	Biological Process	Regulation of systemic arterial blood pressure by acetylcholine	12	1	211	0.1176
GO:0003069	IPI00692468	Biological Process	Vasodilation by acetylcholine involved in regulation of systemic arterial blood pressure	10	1	211	0.1176
GO:0003070	IPI00692468	Biological Process	Regulation of systemic arterial blood pressure by neurotransmitter	11	1	211	0.1176
GO:0003085	IPI00692468	Biological Process	Negative regulation of systemic arterial blood pressure	10	1	211	0.1176
GO:0006020	IPI00692819	Biological Process	Inositol metabolic process	6	1	211	0.1176
GO:0006021	IPI00692819	Biological Process	Inositol biosynthetic process	7	1	211	0.1176
GO:0006065	IPI00712671	Biological Process	UDP-glucuronate biosynthetic process	11	1	211	0.1176
GO:0006147	IPI00716555	Biological Process	Guanine catabolic process	11	1	211	0.1176
GO:0006178	IPI00686225	Biological Process	Guanine salvage	10	1	211	0.1176
GO:0006388	IPI00700295	Biological Process	tRNA splicing, via endonucleolytic cleavage and ligation	12	1	211	0.1176

GO:0006449	IPI00704728	Biological Process	Regulation of translational termination	9	1	211	0.1176
GO:0006452	IPI00704728	Biological Process	Translational frameshifting	9	1	211	0.1176
GO:0006545	IPI00742596	Biological Process	Glycine biosynthetic process	10	1	211	0.1176
GO:0006595	IPI00711759	Biological Process	Polyamine metabolic process	8	1	211	0.1176
GO:0006596	IPI00711759	Biological Process	Polyamine biosynthetic process	9	1	211	0.1176
GO:0006597	IPI00711759	Biological Process	Spermine biosynthetic process	10	1	211	0.1176
GO:0006607	IPI00707359	Biological Process	NLS-bearing protein import into nucleus	7	1	211	0.1176
GO:0006700	IPI00686601	Biological Process	C21-steroid hormone biosynthetic process	6	1	211	0.1176
GO:0006701	IPI00686601	Biological Process	Progesterone biosynthetic process	7	1	211	0.1176
GO:0006880	IPI00687842	Biological Process	Intracellular sequestering of iron ion	12	1	211	0.1176
GO:0006982	IPI00695965	Biological Process	Response to lipid hydroperoxide	7	1	211	0.1176
GO:0008207	IPI00686601	Biological Process	C21-steroid hormone metabolic process	6	1	211	0.1176
GO:0008215	IPI00711759	Biological Process	Spermine metabolic process	9	1	211	0.1176
GO:0009237	IPI00694312	Biological Process	Siderophore metabolic process	6	1	211	0.1176
GO:0009309	IPI00711759	Biological Process	Amine biosynthetic process	7	1	211	0.1176
GO:0010643	IPI00695666	Biological Process	Cell communication by chemical coupling	6	1	211	0.1176
GO:0010644	IPI00695666	Biological Process	Cell communication by electrical coupling	6	1	211	0.1176
GO:0010669	IPI00697184	Biological Process	Epithelial structure maintenance	7	1	211	0.1176
GO:0010880	IPI00695508	Biological Process	Regulation of release of sequestered calcium ion into cytosol by sarcoplasmic reticulum	16	1	211	0.1176
GO:0010898	IPI00695965	Biological Process	Positive regulation of triglyceride catabolic process	11	1	211	0.1176
GO:0016556	IPI00699107	Biological Process	mRNA modification	10	1	211	0.1176
GO:0019290	IPI00694312	Biological Process	Siderophore biosynthetic process	10	1	211	0.1176
GO:0019307	IPI00689323	Biological Process	Mannose biosynthetic process	9	1	211	0.1176
GO:0019427	IPI00696912	Biological Process	Acetyl-CoA biosynthetic process from acetate	9	1	211	0.1176
GO:0019478	IPI00711419	Biological Process	D-amino acid catabolic process	9	1	211	0.1176
GO:0019682	IPI00706942	Biological Process	Glyceraldehyde-3-phosphate metabolic process	6	1	211	0.1176
GO:0021682	IPI00707359	Biological Process	Nerve maturation	9	1	211	0.1176
GO:0022401	IPI00703776	Biological Process	Negative adaptation of signaling pathway	9	1	211	0.1176
GO:0030277	IPI00697184	Biological Process	Maintenance of gastrointestinal epithelium	7	1	211	0.1176
GO:0030505	IPI00703753	Biological Process	Inorganic diphosphate transport	8	1	211	0.1176
GO:0030516	IPI00687601	Biological Process	Regulation of axon extension	9	1	211	0.1176

GO:0030730	IPI00703753	Biological Process	Sequestering of triglyceride	7	1	211	0.1176
GO:0031342	IPI00693338	Biological Process	Negative regulation of cell killing	5	1	211	0.1176
GO:0031427	IPI00742596	Biological Process	Response to methotrexate	6	1	211	0.1176
GO:0032263	IPI00686225	Biological Process	GMP salvage	12	1	211	0.1176
GO:0032264	IPI00686225	Biological Process	IMP salvage	10	1	211	0.1176
GO:0032377	IPI00686601	Biological Process	Regulation of intracellular lipid transport	7	1	211	0.1176
GO:0032379	IPI00686601	Biological Process	Positive regulation of intracellular lipid transport	8	1	211	0.1176
GO:0032380	IPI00686601	Biological Process	Regulation of intracellular sterol transport	8	1	211	0.1176
GO:0032382	IPI00686601	Biological Process	Positive regulation of intracellular sterol transport	9	1	211	0.1176
GO:0032383	IPI00686601	Biological Process	Regulation of intracellular cholesterol transport	9	1	211	0.1176
GO:0032385	IPI00686601	Biological Process	Positive regulation of intracellular cholesterol transport	10	1	211	0.1176
GO:0033194	IPI00695965	Biological Process	Response to hydroperoxide	6	1	211	0.1176
GO:0033261	IPI00693338	Biological Process	Regulation of S phase	NA	1	211	0.1176
GO:0033275	IPI00688489	Biological Process	Actin-myosin filament sliding	8	1	211	0.1176
GO:0034035	IPI00703753	Biological Process	Purine ribonucleoside bisphosphate metabolic process	11	1	211	0.1176
GO:0034401	IPI00690446	Biological Process	Chromatin organization involved in regulation of transcription	12	1	211	0.1176
GO:0034444	IPI00695965	Biological Process	Regulation of plasma lipoprotein particle oxidation	8	1	211	0.1176
GO:0034445	IPI00695965	Biological Process	Negative regulation of plasma lipoprotein particle oxidation	9	1	211	0.1176
GO:0035025	IPI00703776	Biological Process	Positive regulation of Rho protein signal transduction	12	1	211	0.1176
GO:0035067	IPI00690446	Biological Process	Negative regulation of histone acetylation	11	1	211	0.1176
GO:0035105	IPI00689750	Biological Process	Sterol regulatory element binding protein import into nucleus	15	1	211	0.1176
GO:0035502	IPI00703547	Biological Process	Metanephric part of ureteric bud development	8	1	211	0.1176
GO:0035774	IPI00703776	Biological Process	Positive regulation of insulin secretion involved in cellular response to glucose stimulus	12	1	211	0.1176
GO:0035971	IPI00700789	Biological Process	Peptidyl-histidine dephosphorylation	9	1	211	0.1176
GO:0039532	IPI00701698	Biological Process	Negative regulation of viral-induced cytoplasmic pattern recognition receptor signaling pathway	8	1	211	0.1176
GO:0039533	IPI00701698	Biological Process	Regulation of MDA-5 signaling pathway	8	1	211	0.1176

GO:0039534	IPI00701698	Biological Process	Negative regulation of MDA-5 signaling pathway	9	1	211	0.1176
GO:0039536	IPI00701698	Biological Process	Negative regulation of RIG-I signaling pathway	9	1	211	0.1176
GO:0042053	IPI00686225	Biological Process	Regulation of dopamine metabolic process	9	1	211	0.1176
GO:0042069	IPI00686225	Biological Process	Regulation of catecholamine metabolic process	8	1	211	0.1176
GO:0042094	IPI00912603	Biological Process	Interleukin-2 biosynthetic process	8	1	211	0.1176
GO:0042401	IPI00711759	Biological Process	Cellular biogenic amine biosynthetic process	8	1	211	0.1176
GO:0042446	IPI00686601	Biological Process	Hormone biosynthetic process	5	1	211	0.1176
GO:0042448	IPI00686601	Biological Process	Progesterone metabolic process	7	1	211	0.1176
GO:0042554	IPI00692468	Biological Process	Superoxide anion generation	7	1	211	0.1176
GO:0042640	IPI00699355	Biological Process	Anagen	12	1	211	0.1176
GO:0042984	IPI00912603	Biological Process	Regulation of amyloid precursor protein biosynthetic process	9	1	211	0.1176
GO:0042986	IPI00912603	Biological Process	Positive regulation of amyloid precursor protein biosynthetic process	10	1	211	0.1176
GO:0043000	IPI00708921	Biological Process	Golgi to plasma membrane CFTR protein transport	10	1	211	0.1176
GO:0043486	IPI00703854	Biological Process	Histone exchange	10	1	211	0.1176
GO:0044240	IPI00695965	Biological Process	Multicellular organismal lipid catabolic process	7	1	211	0.1176
GO:0045010	IPI00706141	Biological Process	Actin nucleation	12	1	211	0.1176
GO:0045056	IPI00695142	Biological Process	Transcytosis	5	1	211	0.1176
GO:0045719	IPI00703753	Biological Process	Negative regulation of glycogen biosynthetic process	11	1	211	0.1176
GO:0045773	IPI00687601	Biological Process	Positive regulation of axon extension	10	1	211	0.1176
GO:0045901	IPI00704728	Biological Process	Positive regulation of translational elongation	10	1	211	0.1176
GO:0045905	IPI00704728	Biological Process	Positive regulation of translational termination	10	1	211	0.1176
GO:0045915	IPI00686225	Biological Process	Positive regulation of catecholamine metabolic process	9	1	211	0.1176
GO:0045964	IPI00686225	Biological Process	Positive regulation of dopamine metabolic process	10	1	211	0.1176
GO:0046099	IPI00686225	Biological Process	Guanine biosynthetic process	11	1	211	0.1176
GO:0046398	IPI00712671	Biological Process	UDP-glucuronate metabolic process	11	1	211	0.1176
GO:0046416	IPI00711419	Biological Process	D-amino acid metabolic process	8	1	211	0.1176
GO:0046654	IPI00742596	Biological Process	Tetrahydrofolate biosynthetic process	9	1	211	0.1176
GO:0048302	IPI00693338	Biological Process	Regulation of isotype switching to IgG isotypes	10	1	211	0.1176
GO:0048304	IPI00693338	Biological Process	Positive regulation of isotype switching to IgG isotypes	11	1	211	0.1176

GO:0048387	IPI00691963	Biological Process	Negative regulation of retinoic acid receptor signaling pathway	10	1	211	0.1176
GO:0048773	IPI00692468	Biological Process	Erythrophore differentiation	8	1	211	0.1176
GO:0048807	IPI00697184	Biological Process	Female genitalia morphogenesis	8	1	211	0.1176
GO:0048820	IPI00699355	Biological Process	Hair follicle maturation	11	1	211	0.1176
GO:0050427	IPI00703753	Biological Process	3'-phosphoadenosine 5'-phosphosulfate metabolic process	12	1	211	0.1176
GO:0050908	IPI00697184	Biological Process	Detection of light stimulus involved in visual perception	10	1	211	0.1176
GO:0050962	IPI00697184	Biological Process	Detection of light stimulus involved in sensory perception	9	1	211	0.1176
GO:0050992	IPI00944429	Biological Process	Dimethylallyl diphosphate biosynthetic process	7	1	211	0.1176
GO:0050993	IPI00944429	Biological Process	Dimethylallyl diphosphate metabolic process	8	1	211	0.1176
GO:0051014	IPI00706141	Biological Process	Actin filament severing	6	1	211	0.1176
GO:0051024	IPI00697184	Biological Process	Positive regulation of immunoglobulin secretion	9	1	211	0.1176
GO:0051125	IPI00690160	Biological Process	Regulation of actin nucleation	13	1	211	0.1176
GO:0051126	IPI00690160	Biological Process	Negative regulation of actin nucleation	14	1	211	0.1176
GO:0055011	IPI00692676	Biological Process	Atrial cardiac muscle cell differentiation	9	1	211	0.1176
GO:0055014	IPI00692676	Biological Process	Atrial cardiac muscle cell development	10	1	211	0.1176
GO:0055021	IPI00697184	Biological Process	Regulation of cardiac muscle tissue growth	11	1	211	0.1176
GO:0055024	IPI00697184	Biological Process	Regulation of cardiac muscle tissue development	10	1	211	0.1176
GO:0060020	IPI00689228	Biological Process	Bergmann glial cell differentiation	9	1	211	0.1176
GO:0060028	IPI01018577	Biological Process	Convergent extension involved in axis elongation	8	1	211	0.1176
GO:0060043	IPI00697184	Biological Process	Regulation of cardiac muscle cell proliferation	6	1	211	0.1176
GO:0060044	IPI00697184	Biological Process	Negative regulation of cardiac muscle cell proliferation	6	1	211	0.1176
GO:0060059	IPI00697184	Biological Process	Embryonic retina morphogenesis in camera-type eye	8	1	211	0.1176
GO:0060065	IPI00697184	Biological Process	Uterus development	6	1	211	0.1176
GO:0060157	IPI00697184	Biological Process	Urinary bladder development	9	1	211	0.1176
GO:0060315	IPI00695508	Biological Process	Negative regulation of ryanodine-sensitive calcium-release channel activity	11	1	211	0.1176
GO:0060316	IPI00695508	Biological Process	Positive regulation of ryanodine-sensitive calcium-release channel activity	11	1	211	0.1176

GO:0060420	IPI00697184	Biological Process	Regulation of heart growth	10	1	211	0.1176
GO:0060638	IPI00694504	Biological Process	Mesenchymal-epithelial cell signaling	7	1	211	0.1176
GO:0060739	IPI00694504	Biological Process	Mesenchymal-epithelial cell signaling involved in prostate gland development	8	1	211	0.1176
GO:0060770	IPI00716121	Biological Process	Negative regulation of epithelial cell proliferation involved in prostate gland development	7	1	211	0.1176
GO:0061178	IPI00703776	Biological Process	Regulation of insulin secretion involved in cellular response to glucose stimulus	12	1	211	0.1176
GO:0070171	IPI00717119	Biological Process	Negative regulation of tooth mineralization	12	1	211	0.1176
GO:0070307	IPI00689228	Biological Process	Lens fiber cell development	9	1	211	0.1176
GO:0070874	IPI00703753	Biological Process	Negative regulation of glycogen metabolic process	10	1	211	0.1176
GO:0071624	IPI00701698	Biological Process	Positive regulation of granulocyte chemotaxis	8	1	211	0.1176
GO:0072017	IPI00703547	Biological Process	Distal tubule development	9	1	211	0.1176
GO:0072025	IPI00703547	Biological Process	Distal convoluted tubule development	10	1	211	0.1176
GO:0072027	IPI00703547	Biological Process	Connecting tubule development	9	1	211	0.1176
GO:0072044	IPI00703547	Biological Process	Collecting duct development	7	1	211	0.1176
GO:0072205	IPI00703547	Biological Process	Metanephric collecting duct development	8	1	211	0.1176
GO:0072221	IPI00703547	Biological Process	Metanephric distal convoluted tubule development	11	1	211	0.1176
GO:0072235	IPI00703547	Biological Process	Metanephric distal tubule development	10	1	211	0.1176
GO:0072286	IPI00703547	Biological Process	Metanephric connecting tubule development	10	1	211	0.1176
GO:0090023	IPI00701698	Biological Process	Positive regulation of neutrophil chemotaxis	9	1	211	0.1176
GO:0090208	IPI00695965	Biological Process	Positive regulation of triglyceride metabolic process	10	1	211	0.1176
GO:0090240	IPI00703776	Biological Process	Positive regulation of histone H4 acetylation	12	1	211	0.1176
GO:0090241	IPI00690446	Biological Process	Negative regulation of histone H4 acetylation	12	1	211	0.1176
GO:0090343	IPI00689750	Biological Process	Positive regulation of cell aging	7	1	211	0.1176
GO:1900407	IPI00715339	Biological Process	Regulation of cellular response to oxidative stress	7	1	211	0.1176
GO:1901020	IPI00695508	Biological Process	Negative regulation of calcium ion transmembrane transporter activity	10	1	211	0.1176
GO:1901031	IPI00715339	Biological Process	Regulation of response to reactive oxygen species	7	1	211	0.1176
GO:1901163	IPI00701698	Biological Process	Regulation of trophoblast cell migration	10	1	211	0.1176
GO:1901165	IPI00701698	Biological Process	Positive regulation of trophoblast cell migration	11	1	211	0.1176

GO:1901373	IPI00686601	Biological Process	Lipid hydroperoxide transport	5	1	211	0.1176
GO:1901984	IPI00690446	Biological Process	Negative regulation of protein acetylation	11	1	211	0.1176
GO:2000121	IPI00715339	Biological Process	Regulation of removal of superoxide radicals	8	1	211	0.1176
GO:2000471	IPI00693338	Biological Process	Regulation of hematopoietic stem cell migration	9	1	211	0.1176
GO:2000473	IPI00693338	Biological Process	Positive regulation of hematopoietic stem cell migration	10	1	211	0.1176
GO:2000508	IPI00701698	Biological Process	Regulation of dendritic cell chemotaxis	7	1	211	0.1176
GO:2000510	IPI00701698	Biological Process	Positive regulation of dendritic cell chemotaxis	8	1	211	0.1176
GO:2000757	IPI00690446	Biological Process	Negative regulation of peptidyl-lysine acetylation	10	1	211	0.1176
GO:2001258	IPI00695508	Biological Process	Negative regulation of cation channel activity	10	1	211	0.1176
GO:0001836	IPI00692468;IPI00715354	Biological Process	Release of cytochrome c from mitochondria	7	2	211	0.1195
GO:0002700	IPI00693338;IPI00697184	Biological Process	Regulation of production of molecular mediator of immune response	6	2	211	0.1195
GO:0002791	IPI00697184;IPI00703776	Biological Process	Regulation of peptide secretion	8	2	211	0.1195
GO:0003015	IPI00688489;IPI00688651	Biological Process	Heart process	7	2	211	0.1195
GO:0003073	IPI00692468;IPI00697196	Biological Process	Regulation of systemic arterial blood pressure	9	2	211	0.1195
GO:0006801	IPI00692468;IPI00695965	Biological Process	Superoxide metabolic process	6	2	211	0.1195
GO:0007204	IPI00693338;IPI00701166	Biological Process	Positive regulation of cytosolic calcium ion concentration	12	2	211	0.1195
GO:0009225	IPI00689323;IPI00712671	Biological Process	Nucleotide-sugar metabolic process	10	2	211	0.1195
GO:0050764	IPI00691963;IPI00707101	Biological Process	Regulation of phagocytosis	7	2	211	0.1195
GO:0050796	IPI00697184;IPI00703776	Biological Process	Regulation of insulin secretion	11	2	211	0.1195
GO:0051325	IPI00688651;IPI00694214	Biological Process	Interphase	5	2	211	0.1195
GO:0060041	IPI00697184;IPI00703547	Biological Process	Retina development in camera-type eye	11	2	211	0.1195
GO:0060047	IPI00688489;IPI00688651	Biological Process	Heart contraction	8	2	211	0.1195
GO:0070206	IPI00713573;IPI00837992	Biological Process	Protein trimerization	9	2	211	0.1195
GO:0090087	IPI00697184;IPI00703776	Biological Process	Regulation of peptide transport	7	2	211	0.1195

GO:0090276	IPI00697184;IPI00703776	Biological Process	Regulation of peptide hormone secretion	10	2	211	0.1195
GO:0048585	IPI00691963;IPI00693338;IPI00697757;IPI00700789;IPI00701698;IPI00703753;IPI00703776;IPI00707359;IPI00717119;IPI00871133;IPI00912603	Biological Process	Negative regulation of response to stimulus	5	11	211	0.1234
GO:0032501	IPI00686966;IPI00687372;IPI00687539;IPI00687601;IPI00688489;IPI00688651;IPI00689035;IPI00689228;IPI00689750;IPI00690094;IPI00690160;IPI00691963;IPI00692468;IPI00692676;IPI00693338;IPI00693628;IPI00694204;IPI00694504;IPI00695666;IPI00695965;IPI00697184;IPI00697196;IPI00699355;IPI00700295;IPI00701166;IPI00701790;IPI00702028;IPI00703547;IPI00703753;IPI00703854;IPI00706942;IPI00707101;IPI00707359;IPI00712671;IPI00715354;IPI00716121;IPI00717119;IPI00718311;IPI00730144;IPI00839134;IPI00840588;IP	Biological Process	Multicellular organismal process	3	45	211	0.1236

GO:0008150	IPI00685278;IPI00685792;IPI00686183;IPI00686225;IPI00686601;IPI00686803;IPI00686841;IPI00686966;IPI00687372;IPI00687539;IPI00687601;IPI00687625;IPI00687657;IPI00687842;IPI00688489;IPI00688608;IPI00688651;IPI00688921;IPI00689035;IPI00689228;IPI00689323;IPI00689325;IPI00689362;IPI00689750;IPI00689760;IPI00690001;IPI00690094;IPI00690160;IPI00690232;IPI00690308;IPI00690446;IPI00690785;IPI00691212;IPI00691669;IPI00691963;IPI00692468;IPI00692627;IPI00692676;IPI00692819;IPI00692911;IPI00693338;IP	Biological Process	Biological_process	2	176	211	0.1240
GO:0012501	IPI00686966;IPI00688489;IPI00692468;IPI00694739;IPI00694751;IPI00699355;IPI00701698;IPI00703129;IPI00704728;IPI00704835;IPI00708921;IPI00715354;IPI00912603	Biological Process	Programmed cell death	6	13	211	0.1241
GO:0014706	IPI00688489;IPI00689750;IPI00692676;IPI00694504;IPI00697184	Biological Process	Striated muscle tissue development	7	5	211	0.1248
GO:0045087	IPI00686841;IPI00694751;IPI00695965;IPI00701698;IPI00713757	Biological Process	Innate immune response	5	5	211	0.1248

GO:0030833	IPI00688921;IPI00690160;IPI00706141;IPI00711750	Biological Process	Regulation of actin filament polymerization	10	4	211	0.1254
GO:1901607	IPI00694739;IPI00704728;IPI00742596;IPI00883375	Biological Process	Alpha-amino acid biosynthetic process	8	4	211	0.1254
GO:0051239	IPI00686966;IPI00687539;IPI00687601;IPI00688651;IPI00689228;IPI00691963;IPI00693338;IPI00695965;IPI00696930;IPI00697184;IPI00698039;IPI00701166;IPI00701698;IPI00703753;IPI00703776;IPI00706141;IPI00707101;IPI00716121;IPI00716843;IPI00717119	Biological Process	Regulation of multicellular organismal process	5	20	211	0.1254

GO:0044767	IPI00686966;IPI00687372;IPI00687601;IPI00688489;IPI00689035;IPI00689228;IPI00689750;IPI00690094;IPI00691963;IPI00692468;IPI00692676;IPI00693338;IPI00694204;IPI00694504;IPI00695890;IPI00697184;IPI00698900;IPI00699355;IPI00700295;IPI00701790;IPI00702028;IPI00703547;IPI00703854;IPI00706942;IPI00707359;IPI00710385;IPI00712671;IPI00713642;IPI00714673;IPI00718311;IPI00730144;IPI00840588;IPI00847093;IPI01018577	Biological Process	Single-organism developmental process	4	34	211	0.1265

GO:0071840	IPI00686225;IPI00686601;IPI00686966;IPI00687372;IPI00687539;IPI00687601;IPI00687657;IPI00688489;IPI00688921;IPI00689228;IPI00689750;IPI00690160;IPI00690446;IPI00691963;IPI00692468;IPI00694204;IPI00694504;IPI00695506;IPI00695666;IPI00695890;IPI00695965;IPI00698039;IPI00698900;IPI00701223;IPI00701698;IPI00703731;IPI00703776;IPI00703854;IPI00705000;IPI00705941;IPI00706141;IPI00707359;IPI00707587;IPI00707718;IPI00708921;IPI00709922;IPI00711750;IPI00713573;IPI00714673;IPI00715339;IPI00715354;IP	Biological Process	Cellular component organization or biogenesis	3	49	211	0.1277
GO:0006461	IPI00686225;IPI00687539;IPI00687657;IPI00691963;IPI00692468;IPI00698039;IPI00703731;IPI00705000;IPI00707718;IPI00713573;IPI00728768;IPI00837992;IPI01018577	Biological Process	Protein complex assembly	7	13	211	0.1287
GO:0032944	IPI00690160;IPI00693338;IPI00912603	Biological Process	Regulation of mononuclear cell proliferation	7	3	211	0.1309
GO:0050670	IPI00690160;IPI00693338;IPI00912603	Biological Process	Regulation of lymphocyte proliferation	7	3	211	0.1309
GO:0051047	IPI00697184;IPI00703776;IPI00706141	Biological Process	Positive regulation of secretion	7	3	211	0.1309

GO:0055072	IPI00687842;IPI00692468;IPI00694312	Biological Process	Iron ion homeostasis	11	3	211	0.1309
GO:0070663	IPI00690160;IPI00693338;IPI00912603	Biological Process	Regulation of leukocyte proliferation	6	3	211	0.1309
GO:0007610	IPI00686225;IPI00686966;IPI00692468;IPI00703547;IPI00716843	Biological Process	Behavior	3	5	211	0.1339
GO:0051050	IPI00686601;IPI00691963;IPI00695508;IPI00697184;IPI00703776;IPI00706141;IPI00707101;IPI00715354	Biological Process	Positive regulation of transport	7	8	211	0.1341
GO:0051128	IPI00687539;IPI00687601;IPI00688921;IPI00689228;IPI00690160;IPI00690446;IPI00691963;IPI00700789;IPI00701698;IPI00703753;IPI00703776;IPI00704728;IPI00704977;IPI00706141;IPI00707101;IPI00711750;IPI00715354;IPI00842934;IPI00904732	Biological Process	Regulation of cellular component organization	5	19	211	0.1353
GO:0065003	IPI00686225;IPI00687539;IPI00687657;IPI00691963;IPI00692468;IPI00695965;IPI00698039;IPI00701698;IPI00703731;IPI00705000;IPI00707718;IPI00713573;IPI00728768;IPI00837992;IPI01018577	Biological Process	Macromolecular complex assembly	6	15	211	0.1357

GO:0002252	IPI00686841;IPI00690094;IPI00693338;IPI00695506;IPI00701698;IPI00713757	Biological Process	Immune effector process	4	6	211	0.1366
GO:0000096	IPI00694739;IPI00883375	Biological Process	Sulfur amino acid metabolic process	7	2	211	0.1404
GO:0006094	IPI00697184;IPI00706942	Biological Process	Gluconeogenesis	9	2	211	0.1404
GO:0006144	IPI00686225;IPI00716555	Biological Process	Purine nucleobase metabolic process	9	2	211	0.1404
GO:0006721	IPI00692627;IPI00697184	Biological Process	Terpenoid metabolic process	7	2	211	0.1404
GO:0009067	IPI00694739;IPI00883375	Biological Process	Aspartate family amino acid biosynthetic process	9	2	211	0.1404
GO:0009581	IPI00697184;IPI00703776	Biological Process	Detection of external stimulus	5	2	211	0.1404
GO:0030278	IPI00703753;IPI00707101	Biological Process	Regulation of ossification	6	2	211	0.1404
GO:0030595	IPI00690160;IPI00694751	Biological Process	Leukocyte chemotaxis	5	2	211	0.1404
GO:0031345	IPI00687539;IPI00689228	Biological Process	Negative regulation of cell projection organization	7	2	211	0.1404
GO:0032371	IPI00686601;IPI00695965	Biological Process	Regulation of sterol transport	7	2	211	0.1404
GO:0032374	IPI00686601;IPI00695965	Biological Process	Regulation of cholesterol transport	8	2	211	0.1404
GO:0046883	IPI00697184;IPI00703776	Biological Process	Regulation of hormone secretion	9	2	211	0.1404
GO:0051693	IPI00688921;IPI00706141	Biological Process	Actin filament capping	12	2	211	0.1404
GO:0060560	IPI00687601;IPI01018577	Biological Process	Developmental growth involved in morphogenesis	5	2	211	0.1404

GO:0048523	IPI00687539;IPI00687842;IPI00688921;IPI00689228;IPI00690160;IPI00690446;IPI00691963;IPI00692468;IPI00692676;IPI00693338;IPI00694739;IPI00695965;IPI00696930;IPI00697184;IPI00698039;IPI00699355;IPI00700789;IPI00701166;IPI00701698;IPI00703731;IPI00703753;IPI00703776;IPI00705660;IPI00706141;IPI00707359;IPI00707587;IPI00708921;IPI00715354;IPI00716121;IPI00717119;IPI00842934;IPI00904732;IPI00912603;IPI01018577	Biological Process	Negative regulation of cellular process	4	34	211	0.1406
GO:0070271	IPI00686225;IPI00687539;IPI00687657;IPI00691963;IPI00692468;IPI00698039;IPI00703731;IPI00705000;IPI00707718;IPI00713573;IPI00728768;IPI00837992;IPI01018577	Biological Process	Protein complex biogenesis	5	13	211	0.1433
GO:0050767	IPI00687539;IPI00687601;IPI00689228;IPI00691963;IPI00716121	Biological Process	Regulation of neurogenesis	9	5	211	0.1433
GO:0060537	IPI00688489;IPI00689750;IPI00692676;IPI00694504;IPI00697184	Biological Process	Muscle tissue development	6	5	211	0.1433

GO:0032101	IPI00694751;IPI00697757;IPI00701166;IPI00701698;IPI00707101;IPI00871133	Biological Process	Regulation of response to external stimulus	5	6	211	0.1448
GO:0060284	IPI00687539;IPI00687601;IPI00689228;IPI00691963;IPI00701698;IPI00716121	Biological Process	Regulation of cell development	8	6	211	0.1448
GO:0003007	IPI00688489;IPI00692676;IPI00697184	Biological Process	Heart morphogenesis	9	3	211	0.1452
GO:0032507	IPI00695666;IPI00701223;IPI00707359	Biological Process	Maintenance of protein location in cell	6	3	211	0.1452
GO:0048863	IPI00693338;IPI00871133;IPI01018577	Biological Process	Stem cell differentiation	7	3	211	0.1452
GO:0016051	IPI00689323;IPI00692819;IPI00697184;IPI00706942	Biological Process	Carbohydrate biosynthetic process	6	4	211	0.1476
GO:0010557	IPI00689750;IPI00691963;IPI00694851;IPI00699355;IPI00701698;IPI00703776;IPI00704728;IPI00705463;IPI00716158;IPI00842934;IPI00912603;IPI01018577	Biological Process	Positive regulation of macromolecule biosynthetic process	8	12	211	0.1497
GO:0006811	IPI00687842;IPI00688651;IPI00690160;IPI00693338;IPI00695965;IPI00699355;IPI00703753;IPI00706458;IPI00716195	Biological Process	Ion transport	5	9	211	0.1498
GO:0032956	IPI00688921;IPI00690160;IPI00700789;IPI00706141;IPI00711750	Biological Process	Regulation of actin cytoskeleton organization	7	5	211	0.1530

GO:0051960	IPI00687539;IPI00687601;IPI00689228;IPI00691963;IPI00716121	Biological Process	Regulation of nervous system development	8	5	211	0.1530
GO:2000145	IPI00687539;IPI00689750;IPI00690160;IPI00693338;IPI00700789;IPI00701698;IPI00704977;IPI00707359	Biological Process	Regulation of cell motility	7	8	211	0.1547
GO:0002376	IPI00686841;IPI00687842;IPI00690094;IPI00690160;IPI00691963;IPI00692468;IPI00693338;IPI00694751;IPI00695142;IPI00695176;IPI00695506;IPI00695965;IPI00698993;IPI00701698;IPI00703753;IPI00713757	Biological Process	Immune system process	3	16	211	0.1562
GO:0002697	IPI00693338;IPI00697184;IPI00701698;IPI00871133	Biological Process	Regulation of immune effector process	5	4	211	0.1593
GO:0045664	IPI00687539;IPI00687601;IPI00689228;IPI00691963	Biological Process	Regulation of neuron differentiation	10	4	211	0.1593
GO:0048285	IPI00701223;IPI00705941;IPI00842934;IPI00904732	Biological Process	Organelle fission	5	4	211	0.1593
GO:0045185	IPI00695666;IPI00701223;IPI00707359	Biological Process	Maintenance of protein location	6	3	211	0.1599

GO:0007049	IPI00688651;IPI00691963;IPI00694214;IPI00699798;IPI00699803;IPI00701223;IPI00703380;IPI00703854;IPI00705941;IPI00707718;IPI00711573;IPI00904732	Biological Process	Cell cycle	5	12	211	0.1606
GO:0003206	IPI00692676;IPI00697184	Biological Process	Cardiac chamber morphogenesis	10	2	211	0.1620
GO:0006953	IPI00691212;IPI00707101	Biological Process	Acute-phase response	8	2	211	0.1620
GO:0010631	IPI00693628;IPI00699355	Biological Process	Epithelial cell migration	9	2	211	0.1620
GO:0030835	IPI00688921;IPI00706141	Biological Process	Negative regulation of actin filament depolymerization	11	2	211	0.1620
GO:0042102	IPI00690160;IPI00693338	Biological Process	Positive regulation of T cell proliferation	8	2	211	0.1620
GO:0045727	IPI00701698;IPI00704728	Biological Process	Positive regulation of translation	9	2	211	0.1620
GO:0051480	IPI00693338;IPI00701166	Biological Process	Cytosolic calcium ion homeostasis	11	2	211	0.1620
GO:0090132	IPI00693628;IPI00699355	Biological Process	Epithelium migration	6	2	211	0.1620
GO:0010638	IPI00690446;IPI00701698;IPI00703776;IPI00706141;IPI00842934	Biological Process	Positive regulation of organelle organization	5	5	211	0.1629
GO:0008219	IPI00686966;IPI00688489;IPI00692468;IPI00694739;IPI00694751;IPI00699355;IPI00701698;IPI00703129;IPI00704728;IPI00704835;IPI00708921;IPI00715354;IPI00912603	Biological Process	Cell death	5	13	211	0.1641

GO:0042127	IPI00687842;IPI00690160;IPI00691963;IPI00692468;IPI00693338;IPI00694504;IPI00697184;IPI00699355;IPI00706141;IPI00707587;IPI00715354;IPI00716121;IPI00912603	Biological Process	Regulation of cell proliferation	5	13	211	0.1641
GO:0009893	IPI00686225;IPI00686601;IPI00689750;IPI00690446;IPI00691963;IPI00692468;IPI00693338;IPI00694504;IPI00694851;IPI00695965;IPI00699355;IPI00701698;IPI00703776;IPI00704728;IPI00705463;IPI00716158;IPI00717759;IPI00842934;IPI00912603;IPI01018577	Biological Process	Positive regulation of metabolic process	4	20	211	0.1645

GO:0048731	IPI00686966;IPI00687372;IPI00687539;IPI00687601;IPI00688489;IPI00689035;IPI00689228;IPI00689750;IPI00690094;IPI00691963;IPI00692468;IPI00692676;IPI00693338;IPI00694204;IPI00694504;IPI00695666;IPI00697184;IPI00699355;IPI00700295;IPI00702028;IPI00703547;IPI00707359;IPI00715354;IPI00716121;IPI00717119;IPI00718311;IPI00730144;IPI00840588;IPI00847093;IPI01018577	Biological Process	System development	6	30	211	0.1667
GO:0040012	IPI00687539;IPI00689750;IPI00690160;IPI00693338;IPI00700789;IPI00701698;IPI00704977;IPI00707359	Biological Process	Regulation of locomotion	4	8	211	0.1692
GO:0016265	IPI00686966;IPI00688489;IPI00692468;IPI00694739;IPI00694751;IPI00699355;IPI00701698;IPI00703129;IPI00704728;IPI00704835;IPI00708921;IPI00715354;IPI00912603	Biological Process	Death	4	13	211	0.1695

GO:0051049	IPI00686601;IPI00687601;IPI00688651;IPI00691963;IPI00695506;IPI00695508;IPI00695965;IPI00697184;IPI00703753;IPI00703776;IPI00706141;IPI00707101;IPI00715354	Biological Process	Regulation of transport	6	13	211	0.1695
GO:0044699	IPI00685792;IPI00686601;IPI00686966;IPI00687372;IPI00687539;IPI00687601;IPI00687657;IPI00687842;IPI00688489;IPI00688651;IPI00688921;IPI00689035;IPI00689228;IPI00689325;IPI00689750;IPI00689760;IPI00690094;IPI00690160;IPI00690446;IPI00691963;IPI00692468;IPI00692676;IPI00692819;IPI00692911;IPI00693338;IPI00693628;IPI00694204;IPI00694214;IPI00694504;IPI00694580;IPI00694739;IPI00694751;IPI00694851;IPI00695142;IPI00695506;IPI00695666;IPI00695890;IPI00695965;IPI00697184;IPI00697196;IPI00698039;IP	Biological Process	Single-organism process	3	105	211	0.1699
GO:0000103	IPI00730144	Biological Process	Sulfate assimilation	6	1	211	0.1711
GO:0001306	IPI00692468	Biological Process	Age-dependent response to oxidative stress	7	1	211	0.1711
GO:0001702	IPI00712671	Biological Process	Gastrulation with mouth forming second	9	1	211	0.1711
GO:0001868	IPI00871133	Biological Process	Regulation of complement activation, lectin pathway	7	1	211	0.1711
GO:0001869	IPI00871133	Biological Process	Negative regulation of complement activation, lectin pathway	8	1	211	0.1711
GO:0002011	IPI00707359	Biological Process	Morphogenesis of an epithelial sheet	7	1	211	0.1711

GO:0002244	IPI00693338	Biological Process	Hematopoietic progenitor cell differentiation	7	1	211	0.1711
GO:0002286	IPI00695506	Biological Process	T cell activation involved in immune response	8	1	211	0.1711
GO:0002317	IPI00690094	Biological Process	Plasma cell differentiation	10	1	211	0.1711
GO:0002523	IPI00694751	Biological Process	Leukocyte migration involved in inflammatory response	5	1	211	0.1711
GO:0002704	IPI00693338	Biological Process	Negative regulation of leukocyte mediated immunity	7	1	211	0.1711
GO:0002707	IPI00693338	Biological Process	Negative regulation of lymphocyte mediated immunity	8	1	211	0.1711
GO:0002820	IPI00693338	Biological Process	Negative regulation of adaptive immune response	7	1	211	0.1711
GO:0002823	IPI00693338	Biological Process	Negative regulation of adaptive immune response based on somatic recombination of immune receptors built from immunoglobulin superfamily domains	8	1	211	0.1711
GO:0002925	IPI00693338	Biological Process	Positive regulation of humoral immune response mediated by circulating immunoglobulin	11	1	211	0.1711
GO:0003338	IPI00703547	Biological Process	Metanephros morphogenesis	11	1	211	0.1711
GO:0003401	IPI01018577	Biological Process	Axis elongation	6	1	211	0.1711
GO:0006083	IPI00696912	Biological Process	Acetate metabolic process	9	1	211	0.1711
GO:0006168	IPI00686225	Biological Process	Adenine salvage	10	1	211	0.1711
GO:0006933	IPI00693338	Biological Process	Negative regulation of cell adhesion involved in substrate-bound cell migration	9	1	211	0.1711
GO:0006991	IPI00689750	Biological Process	Response to sterol depletion	5	1	211	0.1711
GO:0007161	IPI00714673	Biological Process	Calcium-independent cell-matrix adhesion	7	1	211	0.1711
GO:0007571	IPI00692468	Biological Process	Age-dependent general metabolic decline	6	1	211	0.1711
GO:0007602	IPI00703776	Biological Process	Phototransduction	7	1	211	0.1711
GO:0008608	IPI00701223	Biological Process	Attachment of spindle microtubules to kinetochore	8	1	211	0.1711
GO:0008612	IPI00704728	Biological Process	Peptidyl-lysine modification to peptidyl-hypusine	6	1	211	0.1711
GO:0009584	IPI00697184	Biological Process	Detection of visible light	7	1	211	0.1711
GO:0010842	IPI00703547	Biological Process	Retina layer formation	13	1	211	0.1711
GO:0010896	IPI00695965	Biological Process	Regulation of triglyceride catabolic process	10	1	211	0.1711
GO:0014012	IPI00694504	Biological Process	Peripheral nervous system axon regeneration	9	1	211	0.1711
GO:0014823	IPI00692468	Biological Process	Response to activity	4	1	211	0.1711
GO:0023058	IPI00703776	Biological Process	Adaptation of signaling pathway	8	1	211	0.1711

GO:0030540	IPI00697184	Biological Process	Female genitalia development	7	1	211	0.1711
GO:0030951	IPI00689750	Biological Process	Establishment or maintenance of microtubule cytoskeleton polarity	7	1	211	0.1711
GO:0031103	IPI00694504	Biological Process	Axon regeneration	8	1	211	0.1711
GO:0031115	IPI00904732	Biological Process	Negative regulation of microtubule polymerization	10	1	211	0.1711
GO:0032331	IPI00696930	Biological Process	Negative regulation of chondrocyte differentiation	9	1	211	0.1711
GO:0032364	IPI00692468	Biological Process	Oxygen homeostasis	8	1	211	0.1711
GO:0032413	IPI00695508	Biological Process	Negative regulation of ion transmembrane transporter activity	9	1	211	0.1711
GO:0032623	IPI00912603	Biological Process	Interleukin-2 production	6	1	211	0.1711
GO:0032689	IPI00701698	Biological Process	Negative regulation of interferon-gamma production	8	1	211	0.1711
GO:0032717	IPI00703776	Biological Process	Negative regulation of interleukin-8 production	8	1	211	0.1711
GO:0032796	IPI00690160	Biological Process	Uropod organization	6	1	211	0.1711
GO:0032933	IPI00689750	Biological Process	SREBP signaling pathway	14	1	211	0.1711
GO:0032957	IPI00686601	Biological Process	Inositol trisphosphate metabolic process	8	1	211	0.1711
GO:0032958	IPI00686601	Biological Process	Inositol phosphate biosynthetic process	9	1	211	0.1711
GO:0032959	IPI00686601	Biological Process	Inositol trisphosphate biosynthetic process	10	1	211	0.1711
GO:0034113	IPI00693338	Biological Process	Heterotypic cell-cell adhesion	7	1	211	0.1711
GO:0034442	IPI00695965	Biological Process	Regulation of lipoprotein oxidation	7	1	211	0.1711
GO:0034443	IPI00695965	Biological Process	Negative regulation of lipoprotein oxidation	8	1	211	0.1711
GO:0035112	IPI00697184	Biological Process	Genitalia morphogenesis	7	1	211	0.1711
GO:0035372	IPI00904732	Biological Process	Protein localization to microtubule	10	1	211	0.1711
GO:0039531	IPI00701698	Biological Process	Regulation of viral-induced cytoplasmic pattern recognition receptor signaling pathway	7	1	211	0.1711
GO:0039535	IPI00701698	Biological Process	Regulation of RIG-I signaling pathway	8	1	211	0.1711
GO:0040020	IPI00691963	Biological Process	Regulation of meiosis	6	1	211	0.1711
GO:0042089	IPI00912603	Biological Process	Cytokine biosynthetic process	7	1	211	0.1711
GO:0042107	IPI00912603	Biological Process	Cytokine metabolic process	6	1	211	0.1711
GO:0042481	IPI00717119	Biological Process	Regulation of odontogenesis	10	1	211	0.1711
GO:0043044	IPI00703854	Biological Process	ATP-dependent chromatin remodeling	9	1	211	0.1711
GO:0043103	IPI00686225	Biological Process	Hypoxanthine salvage	10	1	211	0.1711
GO:0043149	IPI00703776	Biological Process	Stress fiber assembly	8	1	211	0.1711

GO:0044241	IPI00695965	Biological Process	Lipid digestion	6	1	211	0.1711
GO:0044243	IPI00695965	Biological Process	Multicellular organismal catabolic process	6	1	211	0.1711
GO:0044550	IPI00694312	Biological Process	Secondary metabolite biosynthetic process	6	1	211	0.1711
GO:0045110	IPI00721270	Biological Process	Intermediate filament bundle assembly	8	1	211	0.1711
GO:0045191	IPI00693338	Biological Process	Regulation of isotype switching	9	1	211	0.1711
GO:0045445	IPI00690094	Biological Process	Myoblast differentiation	8	1	211	0.1711
GO:0045586	IPI00693338	Biological Process	Regulation of gamma-delta T cell differentiation	11	1	211	0.1711
GO:0045588	IPI00693338	Biological Process	Positive regulation of gamma-delta T cell differentiation	12	1	211	0.1711
GO:0045653	IPI00698039	Biological Process	Negative regulation of megakaryocyte differentiation	10	1	211	0.1711
GO:0045654	IPI00706141	Biological Process	Positive regulation of megakaryocyte differentiation	10	1	211	0.1711
GO:0045723	IPI00695965	Biological Process	Positive regulation of fatty acid biosynthetic process	11	1	211	0.1711
GO:0045740	IPI00691963	Biological Process	Positive regulation of DNA replication	11	1	211	0.1711
GO:0045824	IPI00871133	Biological Process	Negative regulation of innate immune response	7	1	211	0.1711
GO:0045830	IPI00693338	Biological Process	Positive regulation of isotype switching	10	1	211	0.1711
GO:0045911	IPI00693338	Biological Process	Positive regulation of DNA recombination	11	1	211	0.1711
GO:0045916	IPI00871133	Biological Process	Negative regulation of complement activation	7	1	211	0.1711
GO:0046038	IPI00686225	Biological Process	GMP catabolic process	12	1	211	0.1711
GO:0046083	IPI00686225	Biological Process	Adenine metabolic process	10	1	211	0.1711
GO:0046084	IPI00686225	Biological Process	Adenine biosynthetic process	11	1	211	0.1711
GO:0046516	IPI00704728	Biological Process	Hypusine metabolic process	8	1	211	0.1711
GO:0046640	IPI00693338	Biological Process	Regulation of alpha-beta T cell proliferation	9	1	211	0.1711
GO:0046641	IPI00693338	Biological Process	Positive regulation of alpha-beta T cell proliferation	10	1	211	0.1711
GO:0046643	IPI00693338	Biological Process	Regulation of gamma-delta T cell activation	8	1	211	0.1711
GO:0046645	IPI00693338	Biological Process	Positive regulation of gamma-delta T cell activation	9	1	211	0.1711
GO:0046838	IPI00692819	Biological Process	Phosphorylated carbohydrate dephosphorylation	8	1	211	0.1711
GO:0046855	IPI00692819	Biological Process	Inositol phosphate dephosphorylation	10	1	211	0.1711
GO:0048385	IPI00691963	Biological Process	Regulation of retinoic acid receptor signaling pathway	9	1	211	0.1711
GO:0048539	IPI00693338	Biological Process	Bone marrow development	6	1	211	0.1711
GO:0048639	IPI00687601	Biological Process	Positive regulation of developmental growth	7	1	211	0.1711

GO:0050772	IPI00687601	Biological Process	Positive regulation of axonogenesis	10	1	211	0.1711
GO:0050855	IPI00693338	Biological Process	Regulation of B cell receptor signaling pathway	9	1	211	0.1711
GO:0050857	IPI00693338	Biological Process	Positive regulation of antigen receptor-mediated signaling pathway	9	1	211	0.1711
GO:0050931	IPI00692468	Biological Process	Pigment cell differentiation	7	1	211	0.1711
GO:0051023	IPI00697184	Biological Process	Regulation of immunoglobulin secretion	8	1	211	0.1711
GO:0051238	IPI00687842	Biological Process	Sequestering of metal ion	5	1	211	0.1711
GO:0051445	IPI00691963	Biological Process	Regulation of meiotic cell cycle	5	1	211	0.1711
GO:0051546	IPI00699355	Biological Process	Keratinocyte migration	10	1	211	0.1711
GO:0060026	IPI01018577	Biological Process	Convergent extension	7	1	211	0.1711
GO:0060068	IPI00697184	Biological Process	Vagina development	8	1	211	0.1711
GO:0060314	IPI00695508	Biological Process	Regulation of ryanodine-sensitive calcium-release channel activity	10	1	211	0.1711
GO:0060445	IPI00707359	Biological Process	Branching involved in salivary gland morphogenesis	7	1	211	0.1711
GO:0060736	IPI00718311	Biological Process	Prostate gland growth	7	1	211	0.1711
GO:0060740	IPI00694504	Biological Process	Prostate gland epithelium morphogenesis	8	1	211	0.1711
GO:0060768	IPI00716121	Biological Process	Regulation of epithelial cell proliferation involved in prostate gland development	6	1	211	0.1711
GO:0060993	IPI00703547	Biological Process	Kidney morphogenesis	10	1	211	0.1711
GO:0070129	IPI00701698	Biological Process	Regulation of mitochondrial translation	9	1	211	0.1711
GO:0070131	IPI00701698	Biological Process	Positive regulation of mitochondrial translation	10	1	211	0.1711
GO:0070170	IPI00717119	Biological Process	Regulation of tooth mineralization	11	1	211	0.1711
GO:0070488	IPI00694751	Biological Process	Neutrophil aggregation	9	1	211	0.1711
GO:0071492	IPI00703731	Biological Process	Cellular response to UV-A	9	1	211	0.1711
GO:0071501	IPI00689750	Biological Process	Cellular response to sterol depletion	6	1	211	0.1711
GO:0071622	IPI00701698	Biological Process	Regulation of granulocyte chemotaxis	7	1	211	0.1711
GO:0072170	IPI00703547	Biological Process	Metanephric tubule development	7	1	211	0.1711
GO:0072207	IPI00703547	Biological Process	Metanephric epithelium development	11	1	211	0.1711
GO:0072234	IPI00703547	Biological Process	Metanephric nephron tubule development	9	1	211	0.1711
GO:0072243	IPI00703547	Biological Process	Metanephric nephron epithelium development	12	1	211	0.1711
GO:0072698	IPI00904732	Biological Process	Protein localization to microtubule cytoskeleton	9	1	211	0.1711
GO:0090022	IPI00701698	Biological Process	Regulation of neutrophil chemotaxis	8	1	211	0.1711
GO:0090322	IPI00715339	Biological Process	Regulation of superoxide metabolic process	7	1	211	0.1711

GO:0090342	IPI00689750	Biological Process	Regulation of cell aging	6	1	211	0.1711
GO:1900024	IPI00701698	Biological Process	Regulation of substrate adhesion-dependent cell spreading	10	1	211	0.1711
GO:1900026	IPI00701698	Biological Process	Positive regulation of substrate adhesion-dependent cell spreading	11	1	211	0.1711
GO:2000242	IPI00716121	Biological Process	Negative regulation of reproductive process	5	1	211	0.1711
GO:2000243	IPI00701698	Biological Process	Positive regulation of reproductive process	5	1	211	0.1711
GO:2000258	IPI00871133	Biological Process	Negative regulation of protein activation cascade	8	1	211	0.1711
GO:0010876	IPI00686601;IPI00695965;IPI00699355;IPI00703753	Biological Process	Lipid localization	5	4	211	0.1712
GO:0007275	IPI00686966;IPI00687372;IPI00687539;IPI00687601;IPI00688489;IPI00689035;IPI00689228;IPI00689750;IPI00690094;IPI00691963;IPI00692468;IPI00692676;IPI00693338;IPI00694204;IPI00694504;IPI00695666;IPI00697184;IPI00699355;IPI00700295;IPI00701790;IPI00702028;IPI00703547;IPI00703854;IPI00706942;IPI00707359;IPI00712671;IPI00715354;IPI00716121;IPI00717119;IPI00718311;IPI00730144;IPI00840588;IPI00847093;IPI00903569;IPI01018577	Biological Process	Multicellular organismal development	5	35	211	0.1732

GO:0022008	IPI00686966;IPI00687372;IPI00687539;IPI00687601;IPI00689228;IPI00691963;IPI00692468;IPI00694504;IPI00695666;IPI00707359;IPI00716121	Biological Process	Neurogenesis	7	11	211	0.1741
GO:0006260	IPI00694214;IPI00695890;IPI00703854	Biological Process	DNA replication	9	3	211	0.1751
GO:0006323	IPI00698039;IPI00705000;IPI00728768	Biological Process	DNA packaging	10	3	211	0.1751
GO:0006333	IPI00698039;IPI00705000;IPI00728768	Biological Process	Chromatin assembly or disassembly	7	3	211	0.1751
GO:0046165	IPI00686601;IPI00692819;IPI00944429	Biological Process	Alcohol biosynthetic process	7	3	211	0.1751
GO:0050870	IPI00690094;IPI00690160;IPI00693338	Biological Process	Positive regulation of T cell activation	8	3	211	0.1751
GO:0048699	IPI00686966;IPI00687372;IPI00687539;IPI00687601;IPI00689228;IPI00691963;IPI00692468;IPI00694504;IPI00695666;IPI00716121	Biological Process	Generation of neurons	8	10	211	0.1758
GO:0006457	IPI00689325;IPI00690785;IPI00691963;IPI00698900;IPI00699107;IPI00703731;IPI00707452;IPI00714481	Biological Process	Protein folding	7	8	211	0.1766
GO:0044711	IPI00686601;IPI00692819;IPI00694312;IPI00694739;IPI00704728;IPI00712671;IPI00742596;IPI00883375;IPI00944429	Biological Process	Single-organism biosynthetic process	5	9	211	0.1767

GO:0010243	IPI00697184;IPI00702650;IPI00703753;IPI00713573;IPI00742596;IPI00842934	Biological Process	Response to organonitrogen compound	5	6	211	0.1801
GO:0008064	IPI00688921;IPI00690160;IPI00706141;IPI00711750	Biological Process	Regulation of actin polymerization or depolymerization	9	4	211	0.1835
GO:0030832	IPI00688921;IPI00690160;IPI00706141;IPI00711750	Biological Process	Regulation of actin filament length	8	4	211	0.1835
GO:0044708	IPI00686966;IPI00692468;IPI00703547;IPI00716843	Biological Process	Single-organism behavior	4	4	211	0.1835
GO:0051249	IPI00690094;IPI00690160;IPI00693338;IPI00912603	Biological Process	Regulation of lymphocyte activation	6	4	211	0.1835
GO:0009968	IPI00691963;IPI00693338;IPI00700789;IPI00701698;IPI00703753;IPI00703776;IPI00707359;IPI00717119;IPI00912603	Biological Process	Negative regulation of signal transduction	8	9	211	0.1838
GO:0003205	IPI00692676;IPI00697184	Biological Process	Cardiac chamber development	9	2	211	0.1842
GO:0022403	IPI00688651;IPI00694214	Biological Process	Cell cycle phase	4	2	211	0.1842
GO:0030183	IPI00690094;IPI00693338	Biological Process	B cell differentiation	9	2	211	0.1842
GO:0030307	IPI00687601;IPI00715354	Biological Process	Positive regulation of cell growth	7	2	211	0.1842
GO:0048145	IPI00687842;IPI00692468	Biological Process	Regulation of fibroblast proliferation	6	2	211	0.1842
GO:0048593	IPI00697184;IPI00703547	Biological Process	Camera-type eye morphogenesis	11	2	211	0.1842
GO:0050769	IPI00687601;IPI00716121	Biological Process	Positive regulation of neurogenesis	10	2	211	0.1842
GO:0050864	IPI00693338;IPI00912603	Biological Process	Regulation of B cell activation	7	2	211	0.1842
GO:0051099	IPI00685792;IPI00703776	Biological Process	Positive regulation of binding	6	2	211	0.1842

GO:0051607	IPI00686841;IPI00693338	Biological Process	Defense response to virus	5	2	211	0.1842
GO:0090130	IPI00693628;IPI00699355	Biological Process	Tissue migration	5	2	211	0.1842
GO:0048518	IPI00686225;IPI00686601;IPI00686966;IPI00687539;IPI00687601;IPI00688651;IPI00689325;IPI00689750;IPI00690094;IPI00690160;IPI00690446;IPI00691963;IPI00692468;IPI00693338;IPI00694504;IPI00694751;IPI00694851;IPI00695508;IPI00695965;IPI00697184;IPI00699355;IPI00700789;IPI00701698;IPI00703731;IPI00703776;IPI00704728;IPI00705463;IPI00706141;IPI00707101;IPI00713757;IPI00715354;IPI00716121;IPI00716158;IPI00717759;IPI00842934;IPI00912603;IPI01018577	Biological Process	Positive regulation of biological process	5	37	211	0.1893
GO:0048608	IPI00694504;IPI00697184;IPI00718311	Biological Process	Reproductive structure development	5	3	211	0.1906
GO:0051262	IPI00686225;IPI00687657;IPI00692468	Biological Process	Protein tetramerization	9	3	211	0.1906
GO:0061458	IPI00694504;IPI00697184;IPI00718311	Biological Process	Reproductive system development	7	3	211	0.1906

GO:0045935	IPI00689750;IPI00691963;IPI00693338;IPI00694851;IPI00699355;IPI00703776;IPI00705463;IPI00716158;IPI00717759;IPI00842934;IPI01018577	Biological Process	Positive regulation of nucleobase-containing compound metabolic process	8	11	211	0.1930
GO:0043065	IPI00689325;IPI00693338;IPI00701698;IPI00703731;IPI00706141	Biological Process	Positive regulation of apoptotic process	9	5	211	0.1942
GO:0043254	IPI00688921;IPI00690160;IPI00706141;IPI00711750;IPI00904732	Biological Process	Regulation of protein complex assembly	6	5	211	0.1942
GO:0031325	IPI00686225;IPI00689750;IPI00690446;IPI00691963;IPI00692468;IPI00693338;IPI00694851;IPI00695965;IPI00699355;IPI00701698;IPI00703776;IPI00704728;IPI00705463;IPI00716158;IPI00717759;IPI00842934;IPI00912603;IPI01018577	Biological Process	Positive regulation of cellular metabolic process	6	18	211	0.1960
GO:0007517	IPI00688489;IPI00689750;IPI00694504;IPI00702028	Biological Process	Muscle organ development	8	4	211	0.1960
GO:0030198	IPI00707587;IPI00709922;IPI00715339;IPI00840588	Biological Process	Extracellular matrix organization	6	4	211	0.1960
GO:0030258	IPI00692819;IPI00694312;IPI00699355;IPI00702650	Biological Process	Lipid modification	6	4	211	0.1960
GO:0040007	IPI00687601;IPI00699355;IPI00718311;IPI01018577	Biological Process	Growth	3	4	211	0.1960

GO:0043062	IPI00707587;IPI00709922;IPI00715339;IPI00840588	Biological Process	Extracellular structure organization	5	4	211	0.1960
GO:0065007	IPI00685278;IPI00685792;IPI00686225;IPI00686601;IPI00686841;IPI00686966;IPI00687539;IPI00687601;IPI00687625;IPI00687842;IPI00688651;IPI00688921;IPI00689228; IPI00689325;IPI00689750;IPI00690001;IPI00690094;IPI00690160;IPI00690446;IPI00691212;IPI00691963;IPI00692468;IPI00692627;IPI00692676;IPI00692819;IPI00692911; IPI00693338;IPI00694214;IPI00694312;IPI00694504;IPI00694580;IPI00694739;IPI00694751;IPI00694851;IPI00695331;IPI00695489;IPI00695506;IPI00695508;IPI00695666; IPI00695890;IPI00695965;IP	Biological Process	Biological regulation	3	87	211	0.2005
GO:0055086	IPI00686225;IPI00687539;IPI00689323;IPI00703753;IPI00706942;IPI00712671;IPI00712775;IPI00716555;IPI00726650;IPI00742596;IPI00883375;IPI01028487	Biological Process	Nucleobase-containing small molecule metabolic process	7	12	211	0.2021
GO:0043068	IPI00689325;IPI00693338;IPI00701698;IPI00703731;IPI00706141	Biological Process	Positive regulation of programmed cell death	8	5	211	0.2050

GO:0055065	IPI00687842;IPI00692468;IPI00693338;IPI00694312;IPI00701166	Biological Process	Metal ion homeostasis	9	5	211	0.2050
GO:0010975	IPI00687539;IPI00687601;IPI00689228	Biological Process	Regulation of neuron projection development	7	3	211	0.2065
GO:0002285	IPI00690094;IPI00695506	Biological Process	Lymphocyte activation involved in immune response	7	2	211	0.2068
GO:0002526	IPI00691212;IPI00707101	Biological Process	Acute inflammatory response	7	2	211	0.2068
GO:0006081	IPI00697184;IPI00706942	Biological Process	Cellular aldehyde metabolic process	5	2	211	0.2068
GO:0006119	IPI00711233;IPI01028487	Biological Process	Oxidative phosphorylation	13	2	211	0.2068
GO:0006366	IPI00694851;IPI00703776	Biological Process	Transcription from RNA polymerase II promoter	11	2	211	0.2068
GO:0007272	IPI00699355;IPI00707359	Biological Process	Ensheathment of neurons	8	2	211	0.2068
GO:0008366	IPI00699355;IPI00707359	Biological Process	Axon ensheathment	9	2	211	0.2068
GO:0008637	IPI00692468;IPI00715354	Biological Process	Apoptotic mitochondrial changes	6	2	211	0.2068
GO:0030837	IPI00688921;IPI00706141	Biological Process	Negative regulation of actin filament polymerization	11	2	211	0.2068
GO:0032946	IPI00690160;IPI00693338	Biological Process	Positive regulation of mononuclear cell proliferation	7	2	211	0.2068
GO:0043242	IPI00688921;IPI00706141	Biological Process	Negative regulation of protein complex disassembly	8	2	211	0.2068
GO:0050671	IPI00690160;IPI00693338	Biological Process	Positive regulation of lymphocyte proliferation	8	2	211	0.2068
GO:0051896	IPI00701698;IPI00707359	Biological Process	Regulation of protein kinase B signaling	9	2	211	0.2068
GO:0060348	IPI00693338;IPI00730144	Biological Process	Bone development	8	2	211	0.2068
GO:0070613	IPI00701698;IPI00871133	Biological Process	Regulation of protein processing	8	2	211	0.2068
GO:1901880	IPI00688921;IPI00706141	Biological Process	Negative regulation of protein depolymerization	11	2	211	0.2068

GO:0009719	IPI00692676;IPI00694204;IPI00697184;IPI00702650;IPI00703753;IPI00713573;IPI00742596;IPI00842934	Biological Process	Response to endogenous stimulus	4	8	211	0.2079
GO:0040008	IPI00686966;IPI00687601;IPI00697184;IPI00703753;IPI00715354;IPI00904104	Biological Process	Regulation of growth	4	6	211	0.2085
GO:0050801	IPI00687842;IPI00692468;IPI00693338;IPI00694312;IPI00701166;IPI00703753	Biological Process	Ion homeostasis	7	6	211	0.2085
GO:0030334	IPI00687539;IPI00689750;IPI00690160;IPI00693338;IPI00701698;IPI00704977;IPI00707359	Biological Process	Regulation of cell migration	8	7	211	0.2091

GO:0006807	IPI00685278;IPI00686225;IPI00686803;IPI00687539;IPI00688608;IPI00689323;IPI00690232;IPI00692468;IPI00692676;IPI00692911;IPI00694214;IPI00694312;IPI00694739;IPI00694851;IPI00695600;IPI00695890;IPI00695965;IPI00697081;IPI00697891;IPI00698039;IPI00699107;IPI00700295;IPI00700547;IPI00701698;IPI00702700;IPI00703753;IPI00703776;IPI00703854;IPI00704728;IPI00705000;IPI00706942;IPI00708018;IPI00711419;IPI00711759;IPI00712671;IPI00712775;IPI00713642;IPI00715287;IPI00716555;IPI00717759;IPI00718311;IP	Biological Process	Nitrogen compound metabolic process	4	47	211	0.2110
GO:0023057	IPI00691963;IPI00693338;IPI00700789;IPI00701698;IPI00703753;IPI00703776;IPI00707359;IPI00717119;IPI00912603	Biological Process	Negative regulation of signaling	5	9	211	0.2132
GO:0051270	IPI00687539;IPI00689750;IPI00690160;IPI00693338;IPI00700789;IPI00701698;IPI00704977;IPI00707359	Biological Process	Regulation of cellular component movement	6	8	211	0.2161
GO:0007507	IPI00688489;IPI00689750;IPI00692468;IPI00692676;IPI00697184	Biological Process	Heart development	8	5	211	0.2161

GO:0048513	IPI00686966;IPI00688489;IPI00689228;IPI00689750;IPI00690094;IPI00692468;IPI00692676;IPI00693338;IPI00694204;IPI00694504;IPI00697184;IPI00699355;IPI00700295;IPI00702028;IPI00703547;IPI00707359;IPI00715354;IPI00717119;IPI00718311;IPI00847093;IPI01018577	Biological Process	Organ development	7	21	211	0.2178
GO:0001845	IPI00690160	Biological Process	Phagolysosome assembly	7	1	211	0.2214
GO:0002862	IPI00697757	Biological Process	Negative regulation of inflammatory response to antigenic stimulus	7	1	211	0.2214
GO:0002921	IPI00871133	Biological Process	Negative regulation of humoral immune response	7	1	211	0.2214
GO:0002923	IPI00693338	Biological Process	Regulation of humoral immune response mediated by circulating immunoglobulin	10	1	211	0.2214
GO:0003044	IPI00692468	Biological Process	Regulation of systemic arterial blood pressure mediated by a chemical signal	10	1	211	0.2214
GO:0003407	IPI00703547	Biological Process	Neural retina development	12	1	211	0.2214
GO:0006145	IPI00716555	Biological Process	Purine nucleobase catabolic process	10	1	211	0.2214
GO:0006208	IPI00687539	Biological Process	Pyrimidine nucleobase catabolic process	10	1	211	0.2214
GO:0006367	IPI00694851	Biological Process	Transcription initiation from RNA polymerase II promoter	10	1	211	0.2214
GO:0006642	IPI00842934	Biological Process	Triglyceride mobilization	9	1	211	0.2214
GO:0008045	IPI00687372	Biological Process	Mmotor neuron axon guidance	8	1	211	0.2214
GO:0009298	IPI00689323	Biological Process	GDP-mannose biosynthetic process	11	1	211	0.2214
GO:0009396	IPI00742596	Biological Process	Folic acid-containing compound biosynthetic process	8	1	211	0.2214
GO:0009593	IPI00692468	Biological Process	Detection of chemical stimulus	5	1	211	0.2214
GO:0010613	IPI00688651	Biological Process	Positive regulation of cardiac muscle hypertrophy	11	1	211	0.2214
GO:0010829	IPI00703753	Biological Process	Negative regulation of glucose transport	10	1	211	0.2214
GO:0014742	IPI00688651	Biological Process	Positive regulation of muscle hypertrophy	8	1	211	0.2214

GO:0014866	IPI00688489	Biological Process	Skeletal myofibril assembly	9	1	211	0.2214
GO:0030240	IPI00688489	Biological Process	Skeletal muscle thin filament assembly	10	1	211	0.2214
GO:0030300	IPI00695965	Biological Process	Regulation of intestinal cholesterol absorption	9	1	211	0.2214
GO:0030502	IPI00707101	Biological Process	Negative regulation of bone mineralization	8	1	211	0.2214
GO:0030643	IPI00703753	Biological Process	Cellular phosphate ion homeostasis	10	1	211	0.2214
GO:0030890	IPI00693338	Biological Process	Positive regulation of B cell proliferation	9	1	211	0.2214
GO:0031057	IPI00690446	Biological Process	Negative regulation of histone modification	10	1	211	0.2214
GO:0031102	IPI00694504	Biological Process	Neuron projection regeneration	7	1	211	0.2214
GO:0031146	IPI00700547	Biological Process	SCF-dependent proteasomal ubiquitin-dependent protein catabolic process	11	1	211	0.2214
GO:0031294	IPI00690094	Biological Process	Lymphocyte costimulation	4	1	211	0.2214
GO:0031295	IPI00690094	Biological Process	T cell costimulation	5	1	211	0.2214
GO:0032261	IPI00686225	Biological Process	Purine nucleotide salvage	9	1	211	0.2214
GO:0032649	IPI00701698	Biological Process	Regulation of interferon-gamma production	7	1	211	0.2214
GO:0032695	IPI00701698	Biological Process	Negative regulation of interleukin-12 production	8	1	211	0.2214
GO:0032715	IPI00703776	Biological Process	Negative regulation of interleukin-6 production	8	1	211	0.2214
GO:0033238	IPI00686225	Biological Process	Regulation of cellular amine metabolic process	7	1	211	0.2214
GO:0033240	IPI00686225	Biological Process	Positive regulation of cellular amine metabolic process	8	1	211	0.2214
GO:0033483	IPI00692468	Biological Process	Gas homeostasis	7	1	211	0.2214
GO:0034372	IPI00695965	Biological Process	Very-low-density lipoprotein particle remodeling	8	1	211	0.2214
GO:0034389	IPI00703731	Biological Process	Lipid particle organization	5	1	211	0.2214
GO:0034643	IPI00687601	Biological Process	Establishment of mitochondrion localization, microtubule-mediated	6	1	211	0.2214
GO:0035066	IPI00703776	Biological Process	Positive regulation of histone acetylation	11	1	211	0.2214
GO:0036296	IPI00692468	Biological Process	Response to increased oxygen levels	6	1	211	0.2214
GO:0042098	IPI00693338	Biological Process	T cell proliferation	7	1	211	0.2214
GO:0042130	IPI00912603	Biological Process	Negative regulation of T cell proliferation	9	1	211	0.2214
GO:0042256	IPI00701698	Biological Process	Mature ribosome assembly	7	1	211	0.2214
GO:0042310	IPI00697196	Biological Process	Vasoconstriction	9	1	211	0.2214
GO:0042574	IPI00697184	Biological Process	Retinal metabolic process	6	1	211	0.2214
GO:0043096	IPI00686225	Biological Process	Purine nucleobase salvage	9	1	211	0.2214
GO:0043383	IPI00693338	Biological Process	Negative T cell selection	11	1	211	0.2214

GO:0044380	IPI00904732	Biological Process	Protein localization to cytoskeleton	8	1	211	0.2214
GO:0045059	IPI00693338	Biological Process	Positive thymic T cell selection	12	1	211	0.2214
GO:0045060	IPI00693338	Biological Process	Negative thymic T cell selection	12	1	211	0.2214
GO:0045776	IPI00692468	Biological Process	Negative regulation of blood pressure	9	1	211	0.2214
GO:0046100	IPI00686225	Biological Process	Hypoxanthine metabolic process	10	1	211	0.2214
GO:0046101	IPI00686225	Biological Process	Hypoxanthine biosynthetic process	11	1	211	0.2214
GO:0046325	IPI00703753	Biological Process	Negative regulation of glucose import	11	1	211	0.2214
GO:0046579	IPI00703776	Biological Process	Positive regulation of Ras protein signal transduction	11	1	211	0.2214
GO:0046627	IPI00703753	Biological Process	Negative regulation of insulin receptor signaling pathway	12	1	211	0.2214
GO:0047497	IPI00687601	Biological Process	Mitochondrion transport along microtubule	9	1	211	0.2214
GO:0048066	IPI00692468	Biological Process	Developmental pigmentation	5	1	211	0.2214
GO:0048251	IPI00715339	Biological Process	Elastic fiber assembly	8	1	211	0.2214
GO:0048675	IPI00687601	Biological Process	Axon extension	8	1	211	0.2214
GO:0050687	IPI00701698	Biological Process	Negative regulation of defense response to virus	6	1	211	0.2214
GO:0050746	IPI00695965	Biological Process	Regulation of lipoprotein metabolic process	6	1	211	0.2214
GO:0050748	IPI00695965	Biological Process	Negative regulation of lipoprotein metabolic process	7	1	211	0.2214
GO:0050832	IPI00718757	Biological Process	Defense response to fungus	7	1	211	0.2214
GO:0050872	IPI00690446	Biological Process	White fat cell differentiation	8	1	211	0.2214
GO:0050906	IPI00697184	Biological Process	Detection of stimulus involved in sensory perception	8	1	211	0.2214
GO:0050918	IPI00690160	Biological Process	Positive chemotaxis	6	1	211	0.2214
GO:0051006	IPI00695965	Biological Process	Positive regulation of lipoprotein lipase activity	9	1	211	0.2214
GO:0051057	IPI00703776	Biological Process	Positive regulation of small GTPase mediated signal transduction	10	1	211	0.2214
GO:0051095	IPI00695890	Biological Process	Regulation of helicase activity	7	1	211	0.2214
GO:0051096	IPI00695890	Biological Process	Positive regulation of helicase activity	7	1	211	0.2214
GO:0051350	IPI00700789	Biological Process	Negative regulation of lyase activity	6	1	211	0.2214
GO:0051983	IPI00701223	Biological Process	Regulation of chromosome segregation	6	1	211	0.2214
GO:0055062	IPI00703753	Biological Process	Phosphate ion homeostasis	10	1	211	0.2214
GO:0055093	IPI00692468	Biological Process	Response to hyperoxia	5	1	211	0.2214
GO:0060441	IPI00707359	Biological Process	Epithelial tube branching involved in lung morphogenesis	8	1	211	0.2214
GO:0060512	IPI00694504	Biological Process	Prostate gland morphogenesis	7	1	211	0.2214

GO:0060612	IPI00699355	Biological Process	Adipose tissue development	8	1	211	0.2214
GO:0060669	IPI01018577	Biological Process	Embryonic placenta morphogenesis	6	1	211	0.2214
GO:0060742	IPI00718311	Biological Process	Epithelial cell differentiation involved in prostate gland development	8	1	211	0.2214
GO:0061162	IPI00687601	Biological Process	Establishment of monopolar cell polarity	7	1	211	0.2214
GO:0061326	IPI00703547	Biological Process	Renal tubule development	7	1	211	0.2214
GO:0061339	IPI00687601	Biological Process	Establishment or maintenance of monopolar cell polarity	6	1	211	0.2214
GO:0061387	IPI00687601	Biological Process	Regulation of extent of cell growth	7	1	211	0.2214
GO:0070141	IPI00703731	Biological Process	Response to UV-A	8	1	211	0.2214
GO:0070252	IPI00688489	Biological Process	Actin-mediated cell contraction	7	1	211	0.2214
GO:0070306	IPI00689228	Biological Process	Lens fiber cell differentiation	8	1	211	0.2214
GO:0070486	IPI00694751	Biological Process	Leukocyte aggregation	8	1	211	0.2214
GO:0071173	IPI00701223	Biological Process	Spindle assembly checkpoint	9	1	211	0.2214
GO:0071545	IPI00692819	Biological Process	Inositol phosphate catabolic process	9	1	211	0.2214
GO:0072009	IPI00703547	Biological Process	Nephron epithelium development	11	1	211	0.2214
GO:0072080	IPI00703547	Biological Process	Nephron tubule development	8	1	211	0.2214
GO:0072502	IPI00703753	Biological Process	Cellular trivalent inorganic anion homeostasis	9	1	211	0.2214
GO:0072506	IPI00703753	Biological Process	Trivalent inorganic anion homeostasis	9	1	211	0.2214
GO:0072529	IPI00687539	Biological Process	Pyrimidine-containing compound catabolic process	7	1	211	0.2214
GO:0085029	IPI00715339	Biological Process	Extracellular matrix assembly	7	1	211	0.2214
GO:0090382	IPI00690160	Biological Process	Phagosome maturation	5	1	211	0.2214
GO:1900077	IPI00703753	Biological Process	Negative regulation of cellular response to insulin stimulus	11	1	211	0.2214
GO:1901021	IPI00695508	Biological Process	Positive regulation of calcium ion transmembrane transporter activity	10	1	211	0.2214
GO:1901985	IPI00703776	Biological Process	Positive regulation of protein acetylation	11	1	211	0.2214
GO:1990138	IPI00687601	Biological Process	Neuron projection extension	7	1	211	0.2214
GO:2000249	IPI00700789	Biological Process	Regulation of actin cytoskeleton reorganization	8	1	211	0.2214
GO:2000758	IPI00703776	Biological Process	Positive regulation of peptidyl-lysine acetylation	10	1	211	0.2214
GO:0008652	IPI00694739;IPI00704728;IPI00742596;IPI00883375	Biological Process	Cellular amino acid biosynthetic process	7	4	211	0.2219

GO:0000280	IPI00701223;IPI00705941;IPI00904732	Biological Process	Nuclear division	6	3	211	0.2227
GO:0007067	IPI00701223;IPI00705941;IPI00904732	Biological Process	Mitotic nuclear division	7	3	211	0.2227
GO:0051251	IPI00690094;IPI00690160;IPI00693338	Biological Process	Positive regulation of lymphocyte activation	7	3	211	0.2227
GO:0055076	IPI00687842;IPI00692468;IPI00694312	Biological Process	Transition metal ion homeostasis	10	3	211	0.2227
GO:0048878	IPI00687842;IPI00692468;IPI00693338;IPI00694312;IPI00695965;IPI00697184;IPI00701166;IPI00703753	Biological Process	Chemical homeostasis	6	8	211	0.2243
GO:0032970	IPI00688921;IPI00690160;IPI00700789;IPI00706141;IPI00711750	Biological Process	Regulation of actin filament-based process	6	5	211	0.2273
GO:0045596	IPI00691963;IPI00692468;IPI00696930;IPI00698039;IPI00703753	Biological Process	Negative regulation of cell differentiation	8	5	211	0.2273
GO:0045597	IPI00687601;IPI00693338;IPI00701698;IPI00706141;IPI00716121	Biological Process	Positive regulation of cell differentiation	8	5	211	0.2273
GO:0010648	IPI00691963;IPI00693338;IPI00700789;IPI00701698;IPI00703753;IPI00703776;IPI00707359;IPI00717119;IPI00912603	Biological Process	Negative regulation of cell communication	7	9	211	0.2286
GO:0001892	IPI00694204;IPI01018577	Biological Process	Embryonic placenta development	5	2	211	0.2297
GO:0019228	IPI00699355;IPI00707359	Biological Process	Neuronal action potential	7	2	211	0.2297
GO:0019751	IPI00686601;IPI00692819	Biological Process	Polyol metabolic process	7	2	211	0.2297
GO:0030834	IPI00688921;IPI00706141	Biological Process	Regulation of actin filament depolymerization	10	2	211	0.2297

GO:0032869	IPI00703753;IPI00842934	Biological Process	Cellular response to insulin stimulus	9	2	211	0.2297
GO:0045807	IPI00691963;IPI00707101	Biological Process	Positive regulation of endocytosis	7	2	211	0.2297
GO:0070665	IPI00690160;IPI00693338	Biological Process	Positive regulation of leukocyte proliferation	6	2	211	0.2297
GO:0071229	IPI00685792;IPI00713573	Biological Process	Cellular response to acid chemical	6	2	211	0.2297
GO:0042981	IPI00686966;IPI00689325;IPI00689750;IPI00692468;IPI00692676;IPI00693338;IPI00694739;IPI00694751;IPI00701698;IPI00703731;IPI00705660;IPI00706141;IPI00708921;IPI00715354	Biological Process	Regulation of apoptotic process	8	14	211	0.2348
GO:0002694	IPI00690094;IPI00690160;IPI00693338;IPI00912603	Biological Process	Regulation of leukocyte activation	5	4	211	0.2351
GO:0007423	IPI00689228;IPI00697184;IPI00703547;IPI00847093	Biological Process	Sensory organ development	8	4	211	0.2351
GO:0052548	IPI00686966;IPI00694751;IPI00715354;IPI00871133	Biological Process	Regulation of endopeptidase activity	9	4	211	0.2351
GO:0044282	IPI00692819;IPI00694312;IPI00699355;IPI00702650;IPI00711419;IPI00842934	Biological Process	Small molecule catabolic process	6	6	211	0.2384
GO:0044712	IPI00692819;IPI00694312;IPI00699355;IPI00702650;IPI00711419;IPI00842934	Biological Process	Single-organism catabolic process	5	6	211	0.2384

GO:0010628	IPI00689750;IPI00691963;IPI00694504;IPI00694851;IPI00699355;IPI00703776;IPI00705463;IPI00716158;IPI00842934;IPI01018577	Biological Process	Positive regulation of gene expression	8	10	211	0.2389
GO:0043484	IPI00685278;IPI00701698;IPI00717759	Biological Process	Regulation of RNA splicing	11	3	211	0.2391
GO:0071103	IPI00698039;IPI00705000;IPI00728768	Biological Process	DNA conformation change	9	3	211	0.2391
GO:0050789	IPI00685278;IPI00686225;IPI00686601;IPI00686841;IPI00686966;IPI00687539;IPI00687601;IPI00687625;IPI00687842;IPI00688651;IPI00688921;IPI00689228;IPI00689325;<br>IPI00689750;IPI00690001;IPI00690094;IPI00690160;IPI00690446;IPI00691212;IPI00691963;IPI00692468;IPI00692676;IPI00692819;IPI00692911;IPI00693338;IPI00694214;<br>IPI00694504;IPI00694580;IPI00694739;IPI00694751;IPI00694851;IPI00695331;IPI00695489;IPI00695506;IPI00695508;IPI00695890;IPI00695965;IPI00696930;IPI00697184;<br>IPI00697757;IPI00698039;IP	Biological Process	Regulation of biological process	4	81	211	0.2447
GO:0001701	IPI00689228;IPI00694204;IPI00700295;IPI00701790;IPI00703854;IPI00847093;IPI01018577	Biological Process	In utero embryonic development	9	7	211	0.2454

GO:0009888	IPI00688489;IPI00689228;IPI00689750;IPI00692676;IPI00694504;IPI00697184;IPI00699355;IPI00703547;IPI00707359;IPI00715354;IPI00717119;IPI00718311;IPI00721270;IPI01018577	Biological Process	Ttissue development	5	14	211	0.2471
GO:0048522	IPI00686225;IPI00686966;IPI00687539;IPI00687601;IPI00689325;IPI00689750;IPI00690094;IPI00690160;IPI00690446;IPI00691963;IPI00692468;IPI00693338;IPI00694504;IPI00694851;IPI00695965;IPI00697184;IPI00699355;IPI00700789;IPI00701698;IPI00703731;IPI00703776;IPI00704728;IPI00705463;IPI00706141;IPI00715354;IPI00716121;IPI00716158;IPI00717759;IPI00842934;IPI00912603;IPI01018577	Biological Process	Positive regulation of cellular process	4	31	211	0.2483
GO:0009314	IPI00692468;IPI00697184;IPI00703731;IPI00703776	Biological Process	Response to radiation	5	4	211	0.2485
GO:0052547	IPI00686966;IPI00694751;IPI00715354;IPI00871133	Biological Process	Regulation of peptidase activity	8	4	211	0.2485

GO:2000147	IPI00690160;IPI00693338;IPI00700789;IPI00701698	Biological Process	Positive regulation of cell motility	8	4	211	0.2485
GO:0001894	IPI00690160;IPI00697184	Biological Process	Tissue homeostasis	6	2	211	0.2527
GO:0006576	IPI00695965;IPI00711759	Biological Process	Cellular biogenic amine metabolic process	7	2	211	0.2527
GO:0009066	IPI00694739;IPI00883375	Biological Process	Aspartate family amino acid metabolic process	8	2	211	0.2527
GO:0021782	IPI00689228;IPI00707359	Biological Process	Glial cell development	8	2	211	0.2527
GO:0042398	IPI00704728;IPI00742596	Biological Process	Cellular modified amino acid biosynthetic process	6	2	211	0.2527
GO:0042542	IPI00692468;IPI00695965	Biological Process	Response to hydrogen peroxide	7	2	211	0.2527
GO:0044106	IPI00695965;IPI00711759	Biological Process	Cellular amine metabolic process	6	2	211	0.2527
GO:0044272	IPI00694739;IPI00883375	Biological Process	Sulfur compound biosynthetic process	6	2	211	0.2527
GO:0048592	IPI00697184;IPI00703547	Biological Process	Eye morphogenesis	10	2	211	0.2527
GO:0060326	IPI00690160;IPI00694751	Biological Process	Cell chemotaxis	8	2	211	0.2527
GO:0043067	IPI00686966;IPI00689325;IPI00689750;IPI00692468;IPI00692676;IPI00693338;IPI00694739;IPI00694751;IPI00701698;IPI00703731;IPI00705660;IPI00706141;IPI00708921; IPI00715354	Biological Process	Regulation of programmed cell death	7	14	211	0.2534

GO:0006793	IPI00686225;IPI00686601;IPI00688651;IPI00689323;IPI00690308;IPI00692819;IPI00693338;IPI00695965;IPI00696912;IPI00696930;IPI00699798;IPI00700789;IPI00703753;IPI00706942;IPI00711233;IPI00712671;IPI00712775;IPI00726650;IPI00742596;IPI00944429;IPI01018577;IPI01028487	Biological Process	Phosphorus metabolic process	5	22	211	0.2547
GO:0002696	IPI00690094;IPI00690160;IPI00693338	Biological Process	Positive regulation of leukocyte activation	6	3	211	0.2558
GO:0006820	IPI00695965;IPI00699355;IPI00703753	Biological Process	Anion transport	6	3	211	0.2558
GO:0050867	IPI00690094;IPI00690160;IPI00693338	Biological Process	Positive regulation of cell activation	7	3	211	0.2558
GO:0051052	IPI00691963;IPI00693338;IPI01018577	Biological Process	Regulation of DNA metabolic process	9	3	211	0.2558
GO:1901617	IPI00686601;IPI00692819;IPI00944429	Biological Process	Organic hydroxy compound biosynthetic process	6	3	211	0.2558
GO:0006259	IPI00694214;IPI00695890;IPI00698039;IPI00700547;IPI00703854;IPI00705000;IPI00728768;IPI01018577	Biological Process	DNA metabolic process	8	8	211	0.2585
GO:0016054	IPI00694312;IPI00699355;IPI00702650;IPI00711419;IPI00842934	Biological Process	Organic acid catabolic process	6	5	211	0.2620

GO:0046395	IPI00694312;IPI00699355;IPI00702650;IPI00711419;IPI00842934	Biological Process	Carboxylic acid catabolic process	7	5	211	0.2620
GO:0040017	IPI00690160;IPI00693338;IPI00700789;IPI00701698	Biological Process	Positive regulation of locomotion	5	4	211	0.2621
GO:0042451	IPI00686225;IPI00726650;IPI00883375;IPI01028487	Biological Process	Purine nucleoside biosynthetic process	9	4	211	0.2621
GO:0046129	IPI00686225;IPI00726650;IPI00883375;IPI01028487	Biological Process	Purine ribonucleoside biosynthetic process	10	4	211	0.2621
GO:0051272	IPI00690160;IPI00693338;IPI00700789;IPI00701698	Biological Process	Positive regulation of cellular component movement	7	4	211	0.2621
GO:0070887	IPI00685792;IPI00686966;IPI00687539;IPI00689750;IPI00690160;IPI00692468;IPI00694751;IPI00695965;IPI00703547;IPI00703753;IPI00708921;IPI00713573;IPI00718311;IPI00842934	Biological Process	Cellular response to chemical stimulus	5	14	211	0.2661
GO:0044283	IPI00686601;IPI00692819;IPI00694739;IPI00704728;IPI00712671;IPI00742596;IPI00883375;IPI00944429	Biological Process	Small molecule biosynthetic process	6	8	211	0.2673
GO:0000266	IPI00842934	Biological Process	Mitochondrial fission	6	1	211	0.2687
GO:0001649	IPI00692676	Biological Process	Osteoblast differentiation	7	1	211	0.2687
GO:0001837	IPI01018577	Biological Process	Epithelial to mesenchymal transition	10	1	211	0.2687
GO:0002690	IPI00701698	Biological Process	Positive regulation of leukocyte chemotaxis	7	1	211	0.2687
GO:0002714	IPI00693338	Biological Process	Positive regulation of B cell mediated immunity	9	1	211	0.2687
GO:0002832	IPI00701698	Biological Process	Negative regulation of response to biotic stimulus	6	1	211	0.2687

GO:0002861	IPI00697757	Biological Process	Regulation of inflammatory response to antigenic stimulus	6	1	211	0.2687
GO:0002891	IPI00693338	Biological Process	Positive regulation of immunoglobulin mediated immune response	10	1	211	0.2687
GO:0003084	IPI00697196	Biological Process	Positive regulation of systemic arterial blood pressure	10	1	211	0.2687
GO:0005979	IPI00703753	Biological Process	Regulation of glycogen biosynthetic process	10	1	211	0.2687
GO:0006085	IPI00696912	Biological Process	Acetyl-CoA biosynthetic process	8	1	211	0.2687
GO:0006177	IPI00686225	Biological Process	GMP biosynthetic process	12	1	211	0.2687
GO:0006516	IPI00700547	Biological Process	Glycoprotein catabolic process	7	1	211	0.2687
GO:0006929	IPI00693338	Biological Process	Substrate-dependent cell migration	8	1	211	0.2687
GO:0007016	IPI00707359	Biological Process	Cytoskeletal anchoring at plasma membrane	6	1	211	0.2687
GO:0007628	IPI00686966	Biological Process	Adult walking behavior	6	1	211	0.2687
GO:0009620	IPI00718757	Biological Process	Response to fungus	7	1	211	0.2687
GO:0010332	IPI00692468	Biological Process	Response to gamma radiation	7	1	211	0.2687
GO:0010559	IPI00912603	Biological Process	Regulation of glycoprotein biosynthetic process	8	1	211	0.2687
GO:0010560	IPI00912603	Biological Process	Positive regulation of glycoprotein biosynthetic process	9	1	211	0.2687
GO:0010677	IPI00703753	Biological Process	Negative regulation of cellular carbohydrate metabolic process	7	1	211	0.2687
GO:0010962	IPI00703753	Biological Process	Regulation of glucan biosynthetic process	9	1	211	0.2687
GO:0014002	IPI00689228	Biological Process	Astrocyte development	9	1	211	0.2687
GO:0014065	IPI00701698	Biological Process	Phosphatidylinositol 3-kinase signaling	10	1	211	0.2687
GO:0022011	IPI00707359	Biological Process	Myelination in peripheral nervous system	11	1	211	0.2687
GO:0022600	IPI00697184	Biological Process	Digestive system process	6	1	211	0.2687
GO:0030002	IPI00703753	Biological Process	Cellular anion homeostasis	8	1	211	0.2687
GO:0030282	IPI00717119	Biological Process	Bone mineralization	6	1	211	0.2687
GO:0030500	IPI00707101	Biological Process	Regulation of bone mineralization	7	1	211	0.2687
GO:0030521	IPI00694851	Biological Process	Androgen receptor signaling pathway	9	1	211	0.2687
GO:0030889	IPI00912603	Biological Process	Negative regulation of B cell proliferation	9	1	211	0.2687
GO:0030952	IPI00689750	Biological Process	Establishment or maintenance of cytoskeleton polarity	6	1	211	0.2687
GO:0031099	IPI00694504	Biological Process	Regeneration	5	1	211	0.2687
GO:0031113	IPI00904732	Biological Process	Rgulation of microtubule polymerization	9	1	211	0.2687
GO:0032292	IPI00707359	Biological Process	Peripheral nervous system axon ensheathment	10	1	211	0.2687

GO:0032410	IPI00695508	Biological Process	Negative regulation of transporter activity	8	1	211	0.2687
GO:0033138	IPI00703776	Biological Process	Positive regulation of peptidyl-serine phosphorylation	10	1	211	0.2687
GO:0034370	IPI00695965	Biological Process	Triglyceride-rich lipoprotein particle remodeling	7	1	211	0.2687
GO:0042100	IPI00693338	Biological Process	B cell proliferation	7	1	211	0.2687
GO:0043243	IPI00704728	Biological Process	Positive regulation of protein complex disassembly	8	1	211	0.2687
GO:0043368	IPI00693338	Biological Process	Positive T cell selection	11	1	211	0.2687
GO:0043462	IPI00703268	Biological Process	Regulation of ATPase activity	13	1	211	0.2687
GO:0043691	IPI00695965	Biological Process	Reverse cholesterol transport	8	1	211	0.2687
GO:0045061	IPI00693338	Biological Process	Thymic T cell selection	11	1	211	0.2687
GO:0045577	IPI00693338	Biological Process	Regulation of B cell differentiation	10	1	211	0.2687
GO:0045912	IPI00703753	Biological Process	Negative regulation of carbohydrate metabolic process	7	1	211	0.2687
GO:0046827	IPI00715354	Biological Process	Positive regulation of protein export from nucleus	9	1	211	0.2687
GO:0048026	IPI00717759	Biological Process	Positive regulation of mRNA splicing, via spliceosome	13	1	211	0.2687
GO:0048588	IPI00687601	Biological Process	Developmental cell growth	6	1	211	0.2687
GO:0048806	IPI00697184	Biological Process	Genitalia development	6	1	211	0.2687
GO:0050868	IPI00912603	Biological Process	Negative regulation of T cell activation	8	1	211	0.2687
GO:0051086	IPI00698900	Biological Process	Chaperone mediated protein folding independent of cofactor	10	1	211	0.2687
GO:0051209	IPI00693338	Biological Process	Release of sequestered calcium ion into cytosol	14	1	211	0.2687
GO:0051282	IPI00693338	Biological Process	Regulation of sequestering of calcium ion	12	1	211	0.2687
GO:0051283	IPI00693338	Biological Process	Negative regulation of sequestering of calcium ion	13	1	211	0.2687
GO:0051654	IPI00687601	Biological Process	Establishment of mitochondrion localization	6	1	211	0.2687
GO:0051764	IPI00687539	Biological Process	Actin crosslink formation	8	1	211	0.2687
GO:0055003	IPI00688489	Biological Process	Cardiac myofibril assembly	9	1	211	0.2687
GO:0060401	IPI00693338	Biological Process	Cytosolic calcium ion transport	10	1	211	0.2687
GO:0060402	IPI00693338	Biological Process	Calcium ion transport into cytosol	13	1	211	0.2687
GO:0060425	IPI00707359	Biological Process	Lung morphogenesis	9	1	211	0.2687
GO:0060706	IPI00694204	Biological Process	Cell differentiation involved in embryonic placenta development	7	1	211	0.2687
GO:0070873	IPI00703753	Biological Process	Regulation of glycogen metabolic process	9	1	211	0.2687

GO:0070935	IPI00687625	Biological Process	3'-UTR-mediated mRNA stabilization	12	1	211	0.2687
GO:0072073	IPI00703547	Biological Process	Kidney epithelium development	10	1	211	0.2687
GO:0090207	IPI00695965	Biological Process	Regulation of triglyceride metabolic process	9	1	211	0.2687
GO:1901019	IPI00695508	Biological Process	Regulation of calcium ion transmembrane transporter activity	9	1	211	0.2687
GO:2001251	IPI00690446	Biological Process	Negative regulation of chromosome organization	7	1	211	0.2687
GO:2001259	IPI00695508	Biological Process	Positive regulation of cation channel activity	10	1	211	0.2687
GO:0044087	IPI00687539;IPI00688921;IPI00690160;IPI00706141;IPI00711750;IPI00904732	Biological Process	Regulation of cellular component biogenesis	5	6	211	0.2696
GO:0009416	IPI00697184;IPI00703731;IPI00703776	Biological Process	Response to light stimulus	6	3	211	0.2725
GO:0010941	IPI00686966;IPI00689325;IPI00689750;IPI00692468;IPI00692676;IPI00693338;IPI00694739;IPI00694751;IPI00701698;IPI00703731;IPI00705660;IPI00706141;IPI00708921; IPI00715354	Biological Process	Regulation of cell death	6	14	211	0.2725
GO:0006325	IPI00690446;IPI00698039;IPI00703854;IPI00705000;IPI00728768	Biological Process	Chromatin organization	6	5	211	0.2738
GO:0055080	IPI00687842;IPI00692468;IPI00693338;IPI00694312;IPI00701166	Biological Process	Cation homeostasis	8	5	211	0.2738
GO:0001818	IPI00701698;IPI00703776	Biological Process	Negative regulation of cytokine production	7	2	211	0.2758
GO:0006986	IPI00697196;IPI00700547	Biological Process	Response to unfolded protein	6	2	211	0.2758
GO:0008217	IPI00692468;IPI00697196	Biological Process	Regulation of blood pressure	8	2	211	0.2758
GO:0009308	IPI00695965;IPI00711759	Biological Process	Amine metabolic process	6	2	211	0.2758

GO:0030522	IPI00694851;IPI00699355	Biological Process	Intracellular receptor signaling pathway	7	2	211	0.2758
GO:0032368	IPI00686601;IPI00695965	Biological Process	Regulation of lipid transport	6	2	211	0.2758
GO:0042440	IPI00686225;IPI00694312	Biological Process	Pigment metabolic process	5	2	211	0.2758
GO:0050900	IPI00690160;IPI00694751	Biological Process	Leukocyte migration	4	2	211	0.2758
GO:0070372	IPI00694739;IPI00703776	Biological Process	Regulation of ERK1 and ERK2 cascade	11	2	211	0.2758
GO:0071375	IPI00703753;IPI00842934	Biological Process	Cellular response to peptide hormone stimulus	8	2	211	0.2758
GO:1901653	IPI00703753;IPI00842934	Biological Process	Cellular response to peptide	8	2	211	0.2758
GO:1901879	IPI00688921;IPI00706141	Biological Process	Regulation of protein depolymerization	10	2	211	0.2758
GO:2000021	IPI00693338;IPI00695508	Biological Process	Regulation of ion homeostasis	6	2	211	0.2758
GO:0000226	IPI00687601;IPI00689750;IPI00701223;IPI00707359	Biological Process	Microtubule cytoskeleton organization	6	4	211	0.2759
GO:0050865	IPI00690094;IPI00690160;IPI00693338;IPI00912603	Biological Process	Regulation of cell activation	6	4	211	0.2759
GO:0080134	IPI00686841;IPI00694751;IPI00697757;IPI00701166;IPI00701698;IPI00707101;IPI00715339;IPI00871133;IPI01018577	Biological Process	Regulation of response to stress	5	9	211	0.2771
GO:0006812	IPI00687842;IPI00688651;IPI00690160;IPI00693338;IPI00706458;IPI00716195	Biological Process	Cation transport	6	6	211	0.2802
GO:0009165	IPI00686225;IPI00689323;IPI00712671;IPI00726650;IPI00742596;IPI01028487	Biological Process	Nucleotide biosynthetic process	9	6	211	0.2802

GO:0050776	IPI00686841;IPI00693338;IPI00697757;IPI00701698;IPI00713757;IPI00871133	Biological Process	Regulation of immune response	5	6	211	0.2802
GO:1901293	IPI00686225;IPI00689323;IPI00712671;IPI00726650;IPI00742596;IPI01028487	Biological Process	Nucleoside phosphate biosynthetic process	8	6	211	0.2802
GO:1901698	IPI00697184;IPI00702650;IPI00703753;IPI00713573;IPI00742596;IPI00842934	Biological Process	Response to nitrogen compound	5	6	211	0.2802
GO:0006796	IPI00686225;IPI00686601;IPI00688651;IPI00689323;IPI00690308;IPI00692819;IPI00693338;IPI00695965;IPI00696930;IPI00699798;IPI00700789;IPI00703753;IPI00706942;IPI00711233;IPI00712671;IPI00712775;IPI00726650;IPI00742596;IPI00944429;IPI01018577;IPI01028487	Biological Process	Phosphate-containing compound metabolic process	6	21	211	0.2832
GO:0048646	IPI00688489;IPI00689035;IPI00692676;IPI00694204;IPI00697184;IPI00698900;IPI00703547;IPI00840588	Biological Process	Anatomical structure formation involved in morphogenesis	5	8	211	0.2851
GO:0060341	IPI00686601;IPI00687601;IPI00688651;IPI00695506;IPI00695508;IPI00697184;IPI00703776;IPI00715354	Biological Process	Regulation of cellular localization	5	8	211	0.2851

GO:0006869	IPI00686601;IPI00695965;IPI00699355	Biological Process	Lipid transport	5	3	211	0.2894
GO:0032844	IPI00693338;IPI00695508;IPI00703776	Biological Process	Regulation of homeostatic process	5	3	211	0.2894
GO:0034504	IPI00689750;IPI00691963;IPI00707359	Biological Process	Protein localization to nucleus	8	3	211	0.2894
GO:0043281	IPI00686966;IPI00694751;IPI00715354	Biological Process	Regulation of cysteine-type endopeptidase activity involved in apoptotic process	11	3	211	0.2894
GO:2000116	IPI00686966;IPI00694751;IPI00715354	Biological Process	Regulation of cysteine-type endopeptidase activity	10	3	211	0.2894
GO:0042060	IPI00699355;IPI00701166;IPI00714673;IPI00730144	Biological Process	Wound healing	6	4	211	0.2897
GO:0006091	IPI00692468;IPI00698589;IPI00699798;IPI00703753;IPI00706942;IPI00707559;IPI00708438;IPI00711233;IPI00712677;IPI01028487	Biological Process	Generation of precursor metabolites and energy	5	10	211	0.2930

GO:0006139	IPI00685278;IPI00686225;IPI00686803;IPI00687539;IPI00689323;IPI00690232;IPI00692676;IPI00692911;IPI00694214;IPI00694851;IPI00695600;IPI00695890;IPI00697891;IPI00698039;IPI00699107;IPI00700295;IPI00700547;IPI00701698;IPI00703753;IPI00703776;IPI00703854;IPI00705000;IPI00706942;IPI00708018;IPI00712671;IPI00712775;IPI00713642;IPI00716555;IPI00717759;IPI00726650;IPI00728768;IPI00742596;IPI00883375;IPI01018577;IPI01028487	Biological Process	Nucleobase-containing compound metabolic process	6	35	211	0.2932
GO:0006979	IPI00686183;IPI00692468;IPI00695965;IPI00712677;IPI00727017	Biological Process	Response to oxidative stress	5	5	211	0.2978
GO:0006694	IPI00686601;IPI00944429	Biological Process	Steroid biosynthetic process	7	2	211	0.2988
GO:0006956	IPI00701698;IPI00713757	Biological Process	Complement activation	5	2	211	0.2988
GO:0006959	IPI00701698;IPI00713757	Biological Process	Humoral immune response	5	2	211	0.2988
GO:0034614	IPI00692468;IPI00695965	Biological Process	Cellular response to reactive oxygen species	7	2	211	0.2988
GO:0035966	IPI00697196;IPI00700547	Biological Process	Response to topologically incorrect protein	5	2	211	0.2988
GO:0042113	IPI00690094;IPI00693338	Biological Process	B cell activation	6	2	211	0.2988
GO:0061077	IPI00698900;IPI00703731	Biological Process	Chaperone-mediated protein folding	8	2	211	0.2988

GO:1901564	IPI00686225;IPI00687539;IPI00688608;IPI00692468;IPI00694312;IPI00694739;IPI00695965;IPI00697081;IPI00702700;IPI00703753;IPI00704728;IPI00706942;IPI00711419;IPI00711759;IPI00712775;IPI00715287;IPI00716555;IPI00718311;IPI00726650;IPI00742596;IPI00883375;IPI01028487	Biological Process	Organonitrogen compound metabolic process	5	22	211	0.3009
GO:0031589	IPI00690160;IPI00699355;IPI00714673	Biological Process	Cell-substrate adhesion	5	3	211	0.3064
GO:0048568	IPI00694204;IPI00697184;IPI01018577	Biological Process	Embryonic organ development	7	3	211	0.3064
GO:1901135	IPI00686225;IPI00688608;IPI00689323;IPI00697081;IPI00700547;IPI00702700;IPI00703753;IPI00706942;IPI00712671;IPI00712775;IPI00715287;IPI00726650;IPI00883375;IPI01028487	Biological Process	Carbohydrate derivative metabolic process	5	14	211	0.3122
GO:0032989	IPI00687372;IPI00687601;IPI00688489;IPI00694204;IPI00695890;IPI00698900;IPI00714673;IPI01018577	Biological Process	Cellular component morphogenesis	6	8	211	0.3124

GO:0090407	IPI00686225;IPI00686601;IPI00689323;IPI00712671;IPI00726650;IPI00742596;IPI00944429;IPI01028487	Biological Process	Organophosphate biosynthetic process	6	8	211	0.3124
GO:0000082	IPI00688651	Biological Process	G1/S transition of mitotic cell cycle	8	1	211	0.3131
GO:0001656	IPI00703547	Biological Process	Metanephros development	10	1	211	0.3131
GO:0001914	IPI00693338	Biological Process	Regulation of T cell mediated cytotoxicity	6	1	211	0.3131
GO:0001916	IPI00693338	Biological Process	Positive regulation of T cell mediated cytotoxicity	7	1	211	0.3131
GO:0002313	IPI00690094	Biological Process	Mature B cell differentiation involved in immune response	9	1	211	0.3131
GO:0002335	IPI00690094	Biological Process	Mature B cell differentiation	10	1	211	0.3131
GO:0002687	IPI00701698	Biological Process	Positive regulation of leukocyte migration	6	1	211	0.3131
GO:0002922	IPI00693338	Biological Process	Positive regulation of humoral immune response	7	1	211	0.3131
GO:0006120	IPI00711233	Biological Process	Mitochondrial electron transport, NADH to ubiquinone	16	1	211	0.3131
GO:0006167	IPI00726650	Biological Process	AMP biosynthetic process	11	1	211	0.3131
GO:0007431	IPI00707359	Biological Process	Salivary gland development	8	1	211	0.3131
GO:0007435	IPI00707359	Biological Process	Salivary gland morphogenesis	9	1	211	0.3131
GO:0008361	IPI00687601	Biological Process	Regulation of cell size	6	1	211	0.3131
GO:0009070	IPI00742596	Biological Process	Serine family amino acid biosynthetic process	9	1	211	0.3131
GO:0010611	IPI00688651	Biological Process	Regulation of cardiac muscle hypertrophy	10	1	211	0.3131
GO:0010770	IPI00701698	Biological Process	Positive regulation of cell morphogenesis involved in differentiation	10	1	211	0.3131
GO:0010872	IPI00695965	Biological Process	Regulation of cholesterol esterification	10	1	211	0.3131
GO:0010873	IPI00695965	Biological Process	Positive regulation of cholesterol esterification	11	1	211	0.3131
GO:0010955	IPI00871133	Biological Process	Negative regulation of protein processing	8	1	211	0.3131
GO:0010976	IPI00687539	Biological Process	Positive regulation of neuron projection development	8	1	211	0.3131
GO:0014037	IPI00707359	Biological Process	Schwann cell differentiation	8	1	211	0.3131
GO:0014044	IPI00707359	Biological Process	Schwann cell development	9	1	211	0.3131
GO:0014743	IPI00688651	Biological Process	Regulation of muscle hypertrophy	8	1	211	0.3131
GO:0015698	IPI00703753	Biological Process	Inorganic anion transport	7	1	211	0.3131
GO:0015908	IPI00699355	Biological Process	Fatty acid transport	10	1	211	0.3131
GO:0015914	IPI00695965	Biological Process	Phospholipid transport	8	1	211	0.3131

GO:0016202	IPI00697184	Biological Process	Regulation of striated muscle tissue development	10	1	211	0.3131
GO:0017156	IPI00706141	Biological Process	Calcium ion-dependent exocytosis	7	1	211	0.3131
GO:0019184	IPI00694312	Biological Process	Nonribosomal peptide biosynthetic process	9	1	211	0.3131
GO:0019233	IPI00839134	Biological Process	Sensory perception of pain	8	1	211	0.3131
GO:0019673	IPI00689323	Biological Process	GDP-mannose metabolic process	11	1	211	0.3131
GO:0019748	IPI00694312	Biological Process	Secondary metabolic process	5	1	211	0.3131
GO:0021675	IPI00707359	Biological Process	Nerve development	8	1	211	0.3131
GO:0030593	IPI00694751	Biological Process	Neutrophil chemotaxis	7	1	211	0.3131
GO:0031111	IPI00904732	Biological Process	Negative regulation of microtubule polymerization or depolymerization	9	1	211	0.3131
GO:0031577	IPI00701223	Biological Process	Spindle checkpoint	8	1	211	0.3131
GO:0032370	IPI00686601	Biological Process	Positive regulation of lipid transport	7	1	211	0.3131
GO:0032373	IPI00686601	Biological Process	Positive regulation of sterol transport	8	1	211	0.3131
GO:0032376	IPI00686601	Biological Process	Positive regulation of cholesterol transport	9	1	211	0.3131
GO:0032414	IPI00695508	Biological Process	Positive regulation of ion transmembrane transporter activity	9	1	211	0.3131
GO:0032881	IPI00703753	Biological Process	Regulation of polysaccharide metabolic process	7	1	211	0.3131
GO:0032885	IPI00703753	Biological Process	Regulation of polysaccharide biosynthetic process	8	1	211	0.3131
GO:0032945	IPI00912603	Biological Process	Negative regulation of mononuclear cell proliferation	7	1	211	0.3131
GO:0033209	IPI00708921	Biological Process	Tumor necrosis factor-mediated signaling pathway	9	1	211	0.3131
GO:0033700	IPI00695965	Biological Process	Phospholipid efflux	9	1	211	0.3131
GO:0035272	IPI00707359	Biological Process	Exocrine system development	7	1	211	0.3131
GO:0035384	IPI00696912	Biological Process	Thioester biosynthetic process	6	1	211	0.3131
GO:0042304	IPI00695965	Biological Process	Regulation of fatty acid biosynthetic process	10	1	211	0.3131
GO:0043173	IPI00686225	Biological Process	Nucleotide salvage	10	1	211	0.3131
GO:0043502	IPI00688651	Biological Process	Regulation of muscle adaptation	8	1	211	0.3131
GO:0044058	IPI00695965	Biological Process	Regulation of digestive system process	7	1	211	0.3131
GO:0045058	IPI00693338	Biological Process	T cell selection	10	1	211	0.3131
GO:0045665	IPI00691963	Biological Process	Negative regulation of neuron differentiation	11	1	211	0.3131
GO:0045739	IPI01018577	Biological Process	Positive regulation of DNA repair	11	1	211	0.3131
GO:0045777	IPI00697196	Biological Process	Positive regulation of blood pressure	9	1	211	0.3131

GO:0046037	IPI00686225	Biological Process	GMP metabolic process	12	1	211	0.3131
GO:0046620	IPI00697184	Biological Process	Regulation of organ growth	6	1	211	0.3131
GO:0046626	IPI00703753	Biological Process	Regulation of insulin receptor signaling pathway	11	1	211	0.3131
GO:0046653	IPI00742596	Biological Process	Tetrahydrofolate metabolic process	8	1	211	0.3131
GO:0046849	IPI00703753	Biological Process	Bone remodeling	6	1	211	0.3131
GO:0048634	IPI00697184	Biological Process	Regulation of muscle organ development	9	1	211	0.3131
GO:0048708	IPI00689228	Biological Process	Astrocyte differentiation	8	1	211	0.3131
GO:0050672	IPI00912603	Biological Process	Negative regulation of lymphocyte proliferation	8	1	211	0.3131
GO:0050869	IPI00912603	Biological Process	Negative regulation of B cell activation	8	1	211	0.3131
GO:0050996	IPI00695965	Biological Process	Positive regulation of lipid catabolic process	8	1	211	0.3131
GO:0051491	IPI00687539	Biological Process	Positive regulation of filopodium assembly	9	1	211	0.3131
GO:0051898	IPI00707359	Biological Process	Negative regulation of protein kinase B signaling	10	1	211	0.3131
GO:0060048	IPI00688651	Biological Process	Cardiac muscle contraction	9	1	211	0.3131
GO:0061351	IPI00695666	Biological Process	Neural precursor cell proliferation	5	1	211	0.3131
GO:0065005	IPI00695965	Biological Process	Protein-lipid complex assembly	7	1	211	0.3131
GO:0070664	IPI00912603	Biological Process	Negative regulation of leukocyte proliferation	6	1	211	0.3131
GO:0071616	IPI00696912	Biological Process	Acyl-CoA biosynthetic process	7	1	211	0.3131
GO:0071621	IPI00694751	Biological Process	Granulocyte chemotaxis	6	1	211	0.3131
GO:0072384	IPI00687601	Biological Process	Organelle transport along microtubule	8	1	211	0.3131
GO:0090398	IPI00691963	Biological Process	Cellular senescence	6	1	211	0.3131
GO:1901861	IPI00697184	Biological Process	Regulation of muscle tissue development	7	1	211	0.3131
GO:2000648	IPI00693338	Biological Process	Positive regulation of stem cell proliferation	6	1	211	0.3131
GO:2001257	IPI00695508	Biological Process	Regulation of cation channel activity	9	1	211	0.3131
GO:0051260	IPI00686225;IPI00687539;IPI00687657;IPI00692468	Biological Process	Protein homooligomerization	9	4	211	0.3177
GO:0019218	IPI00686601;IPI00695965	Biological Process	Regulation of steroid metabolic process	7	2	211	0.3217
GO:0021700	IPI00699355;IPI00707359	Biological Process	Developmental maturation	5	2	211	0.3217
GO:0022612	IPI00694504;IPI00707359	Biological Process	Gland morphogenesis	9	2	211	0.3217
GO:0022904	IPI00692468;IPI00711233	Biological Process	Respiratory electron transport chain	8	2	211	0.3217
GO:0031346	IPI00687539;IPI00687601	Biological Process	Positive regulation of cell projection organization	7	2	211	0.3217

GO:0032103	IPI00694751;IPI00701698	Biological Process	Positive regulation of response to external stimulus	6	2	211	0.3217
GO:0048024	IPI00701698;IPI00717759	Biological Process	Regulation of mRNA splicing, via spliceosome	12	2	211	0.3217
GO:0048864	IPI00693338;IPI01018577	Biological Process	Stem cell development	8	2	211	0.3217
GO:0072376	IPI00701698;IPI00713757	Biological Process	Protein activation cascade	6	2	211	0.3217
GO:0032386	IPI00686601;IPI00687601;IPI00695506;IPI00695508;IPI00715354	Biological Process	Regulation of intracellular transport	6	5	211	0.3221
GO:0097190	IPI00686966;IPI00692468;IPI00715354	Biological Process	Apoptotic signaling pathway	7	3	211	0.3234
GO:0009887	IPI00688489;IPI00692676;IPI00694504;IPI00697184;IPI00703547;IPI00707359	Biological Process	Organ morphogenesis	8	6	211	0.3234
GO:0009792	IPI00689228;IPI00694204;IPI00697184;IPI00700295;IPI00701790;IPI00703854;IPI00847093;IPI01018577	Biological Process	Embryo development ending in birth or egg hatching	7	8	211	0.3309
GO:0043009	IPI00689228;IPI00694204;IPI00697184;IPI00700295;IPI00701790;IPI00703854;IPI00847093;IPI01018577	Biological Process	Chordate embryonic development	8	8	211	0.3309
GO:0000278	IPI00688651;IPI00701223;IPI00705941;IPI00904732	Biological Process	Mitotic cell cycle	6	4	211	0.3317
GO:0022604	IPI00687601;IPI00688921;IPI00690160;IPI00701698	Biological Process	Regulation of cell morphogenesis	8	4	211	0.3317

GO:0051254	IPI00689750;IPI00694851;IPI00699355;IPI00703776;IPI00705463;IPI00716158;IPI00717759;IPI00842934;IPI01018577	Biological Process	Positive regulation of RNA metabolic process	10	9	211	0.3370
GO:1901700	IPI00687625;IPI00692468;IPI00695965;IPI00697184;IPI00702650;IPI00703753;IPI00713573;IPI00742596;IPI00842934	Biological Process	Response to oxygen-containing compound	5	9	211	0.3370
GO:0001558	IPI00687601;IPI00703753;IPI00715354	Biological Process	Regulation of cell growth	6	3	211	0.3404
GO:0001667	IPI00693628;IPI00699355	Biological Process	Ameboidal-type cell migration	8	2	211	0.3444
GO:0001824	IPI00701790;IPI00703854	Biological Process	Blastocyst development	10	2	211	0.3444
GO:0002263	IPI00690094;IPI00695506	Biological Process	Cell activation involved in immune response	5	2	211	0.3444
GO:0002366	IPI00690094;IPI00695506	Biological Process	Leukocyte activation involved in immune response	6	2	211	0.3444
GO:0010001	IPI00689228;IPI00707359	Biological Process	Glial cell differentiation	7	2	211	0.3444
GO:0010950	IPI00686966;IPI00694751	Biological Process	Positive regulation of endopeptidase activity	11	2	211	0.3444
GO:0010952	IPI00686966;IPI00694751	Biological Process	Positive regulation of peptidase activity	10	2	211	0.3444
GO:0043280	IPI00686966;IPI00694751	Biological Process	Positive regulation of cysteine-type endopeptidase activity involved in apoptotic process	13	2	211	0.3444
GO:0045927	IPI00687601;IPI00715354	Biological Process	Positive regulation of growth	5	2	211	0.3444
GO:0072593	IPI00692468;IPI00695965	Biological Process	Reactive oxygen species metabolic process	5	2	211	0.3444
GO:2001056	IPI00686966;IPI00694751	Biological Process	Positive regulation of cysteine-type endopeptidase activity	12	2	211	0.3444

GO:0051276	IPI00690446;IPI00698039;IPI00701223;IPI00703854;IPI00705000;IPI00728768	Biological Process	Chromosome organization	5	6	211	0.3454
GO:0044281	IPI00686225;IPI00686601;IPI00687539;IPI00688608;IPI00689323;IPI00689325;IPI00692468;IPI00692819;IPI00694312;IPI00694739;IPI00695965;IPI00696912;IPI00697081;IPI00699355;IPI00702650;IPI00703753;IPI00704728;IPI00706942;IPI00711419;IPI00712671;IPI00712775;IPI00716555;IPI00726650;IPI00742596;IPI00842934;IPI00883375;IPI00944429;IPI01028487	Biological Process	Small molecule metabolic process	5	28	211	0.3460

GO:1901360	IPI00685278;IPI00686225;IPI00686601;IPI00686803;IPI00687539;IPI00689323;IPI00690232;IPI00692676;IPI00692911;IPI00694214;IPI00694312;IPI00694851;IPI00695600;IPI00695890;IPI00695965;IPI00697891;IPI00698039;IPI00699107;IPI00700295;IPI00700547;IPI00701698;IPI00703753;IPI00703776;IPI00703854;IPI00705000;IPI00706942;IPI00708018;IPI00712671;IPI00712775;IPI00713642;IPI00716555;IPI00717759;IPI00726650;IPI00728768;IPI00742596;IPI00883375;IPI00944429;IPI01018577;IPI01028487	Biological Process	Organic cyclic compound metabolic process	5	39	211	0.3480
GO:0000070	IPI00701223	Biological Process	Mitotic sister chromatid segregation	8	1	211	0.3548
GO:0000079	IPI00715354	Biological Process	Regulation of cyclin-dependent protein serine/threonine kinase activity	12	1	211	0.3548
GO:0000819	IPI00701223	Biological Process	Sister chromatid segregation	6	1	211	0.3548
GO:0001657	IPI00703547	Biological Process	Ureteric bud development	7	1	211	0.3548
GO:0001910	IPI00693338	Biological Process	Regulation of leukocyte mediated cytotoxicity	5	1	211	0.3548
GO:0001912	IPI00693338	Biological Process	Positive regulation of leukocyte mediated cytotoxicity	6	1	211	0.3548
GO:0002088	IPI00689228	Biological Process	Lens development in camera-type eye	11	1	211	0.3548
GO:0002712	IPI00693338	Biological Process	Regulation of B cell mediated immunity	8	1	211	0.3548
GO:0002889	IPI00693338	Biological Process	Regulation of immunoglobulin mediated immune response	9	1	211	0.3548
GO:0006013	IPI00689323	Biological Process	Mannose metabolic process	9	1	211	0.3548
GO:0006509	IPI00707359	Biological Process	Membrane protein ectodomain proteolysis	8	1	211	0.3548
GO:0006760	IPI00742596	Biological Process	Folic acid-containing compound metabolic process	7	1	211	0.3548

GO:0006957	IPI00713757	Biological Process	Complement activation, alternative pathway	6	1	211	0.3548
GO:0007601	IPI00697184	Biological Process	Visual perception	9	1	211	0.3548
GO:0008299	IPI00944429	Biological Process	Isoprenoid biosynthetic process	6	1	211	0.3548
GO:0009113	IPI00686225	Biological Process	Purine nucleobase biosynthetic process	10	1	211	0.3548
GO:0014031	IPI01018577	Biological Process	Mesenchymal cell development	9	1	211	0.3548
GO:0015718	IPI00699355	Biological Process	Monocarboxylic acid transport	9	1	211	0.3548
GO:0015748	IPI00695965	Biological Process	Organophosphate ester transport	5	1	211	0.3548
GO:0019915	IPI00703753	Biological Process	Lipid storage	6	1	211	0.3548
GO:0031214	IPI00717119	Biological Process	Biomineral tissue development	8	1	211	0.3548
GO:0031341	IPI00693338	Biological Process	Regulation of cell killing	4	1	211	0.3548
GO:0031343	IPI00693338	Biological Process	Positive regulation of cell killing	5	1	211	0.3548
GO:0032655	IPI00701698	Biological Process	Regulation of interleukin-12 production	7	1	211	0.3548
GO:0033120	IPI00717759	Biological Process	Positive regulation of RNA splicing	11	1	211	0.3548
GO:0033135	IPI00703776	Biological Process	Regulation of peptidyl-serine phosphorylation	9	1	211	0.3548
GO:0033144	IPI00691963	Biological Process	Negative regulation of intracellular steroid hormone receptor signaling pathway	10	1	211	0.3548
GO:0033865	IPI00703753	Biological Process	Nucleoside bisphosphate metabolic process	9	1	211	0.3548
GO:0033875	IPI00703753	Biological Process	Ribonucleoside bisphosphate metabolic process	10	1	211	0.3548
GO:0034032	IPI00703753	Biological Process	Purine nucleoside bisphosphate metabolic process	10	1	211	0.3548
GO:0034109	IPI00694751	Biological Process	Homotypic cell-cell adhesion	7	1	211	0.3548
GO:0034367	IPI00695965	Biological Process	Macromolecular complex remodeling	6	1	211	0.3548
GO:0034368	IPI00695965	Biological Process	Protein-lipid complex remodeling	7	1	211	0.3548
GO:0034369	IPI00695965	Biological Process	Plasma lipoprotein particle remodeling	6	1	211	0.3548
GO:0036294	IPI00689750	Biological Process	Cellular response to decreased oxygen levels	7	1	211	0.3548
GO:0042181	IPI00686601	Biological Process	Ketone biosynthetic process	6	1	211	0.3548
GO:0042255	IPI00701698	Biological Process	Ribosome assembly	6	1	211	0.3548
GO:0042559	IPI00742596	Biological Process	Pteridine-containing compound biosynthetic process	7	1	211	0.3548
GO:0042744	IPI00695965	Biological Process	Hydrogen peroxide catabolic process	6	1	211	0.3548
GO:0043029	IPI00690160	Biological Process	T cell homeostasis	6	1	211	0.3548
GO:0043473	IPI00692468	Biological Process	Pigmentation	4	1	211	0.3548
GO:0043542	IPI00693628	Biological Process	Endothelial cell migration	10	1	211	0.3548

GO:0045429	IPI00692468	Biological Process	Positive regulation of nitric oxide biosynthetic process	9	1	211	0.3548
GO:0045744	IPI00703776	Biological Process	Negative regulation of G-protein coupled receptor protein signaling pathway	10	1	211	0.3548
GO:0045923	IPI00695965	Biological Process	Positive regulation of fatty acid metabolic process	11	1	211	0.3548
GO:0046033	IPI00726650	Biological Process	AMP metabolic process	12	1	211	0.3548
GO:0046174	IPI00692819	Biological Process	Polyol catabolic process	8	1	211	0.3548
GO:0046634	IPI00693338	Biological Process	Regulation of alpha-beta T cell activation	8	1	211	0.3548
GO:0046635	IPI00693338	Biological Process	Positive regulation of alpha-beta T cell activation	9	1	211	0.3548
GO:0046889	IPI00695965	Biological Process	Positive regulation of lipid biosynthetic process	7	1	211	0.3548
GO:0048762	IPI01018577	Biological Process	Mesenchymal cell differentiation	8	1	211	0.3548
GO:0050714	IPI00697184	Biological Process	Positive regulation of protein secretion	8	1	211	0.3548
GO:0050732	IPI00693338	Biological Process	Negative regulation of peptidyl-tyrosine phosphorylation	10	1	211	0.3548
GO:0050853	IPI00693338	Biological Process	B cell receptor signaling pathway	8	1	211	0.3548
GO:0050871	IPI00693338	Biological Process	Positive regulation of B cell activation	8	1	211	0.3548
GO:0050953	IPI00697184	Biological Process	Sensory perception of light stimulus	8	1	211	0.3548
GO:0051279	IPI00695508	Biological Process	Regulation of release of sequestered calcium ion into cytosol	15	1	211	0.3548
GO:0051339	IPI00700789	Biological Process	Regulation of lyase activity	6	1	211	0.3548
GO:0051646	IPI00687601	Biological Process	Mitochondrion localization	6	1	211	0.3548
GO:0060193	IPI00695965	Biological Process	Positive regulation of lipase activity	7	1	211	0.3548
GO:0070374	IPI00703776	Biological Process	Positive regulation of ERK1 and ERK2 cascade	12	1	211	0.3548
GO:0071456	IPI00689750	Biological Process	Cellular response to hypoxia	6	1	211	0.3548
GO:0043436	IPI00688608;IPI00692468;IPI00694312;IPI00694739;IPI00696912;IPI00697081;IPI00699355;IPI00702650;IPI00703753;IPI00704728;IPI00711419;IPI00712671;IPI00742596;IPI00842934;IPI00883375	Biological Process	Oxoacid metabolic process	6	15	211	0.3551

GO:0051248	IPI00690446;IPI00693338;IPI00695965;IPI00703753;IPI00703776;IPI00871133	Biological Process	Negative regulation of protein metabolic process	7	6	211	0.3565
GO:0006935	IPI00687372;IPI00690160;IPI00694751	Biological Process	Chemotaxis	5	3	211	0.3574
GO:0031344	IPI00687539;IPI00687601;IPI00689228	Biological Process	Regulation of cell projection organization	6	3	211	0.3574
GO:0042330	IPI00687372;IPI00690160;IPI00694751	Biological Process	Taxis	4	3	211	0.3574
GO:0048732	IPI00694504;IPI00707359;IPI00718311	Biological Process	Gland development	8	3	211	0.3574
GO:1901605	IPI00694739;IPI00704728;IPI00711419;IPI00742596;IPI00883375	Biological Process	Alpha-amino acid metabolic process	7	5	211	0.3590
GO:0060429	IPI00689228;IPI00694504;IPI00703547;IPI00707359;IPI00715354;IPI00718311;IPI00721270;IPI01018577	Biological Process	Epithelium development	6	8	211	0.3590
GO:0009163	IPI00686225;IPI00726650;IPI00883375;IPI01028487	Biological Process	Nucleoside biosynthetic process	8	4	211	0.3599
GO:0031400	IPI00690446;IPI00693338;IPI00703753;IPI00703776	Biological Process	Negative regulation of protein modification process	9	4	211	0.3599
GO:0042455	IPI00686225;IPI00726650;IPI00883375;IPI01028487	Biological Process	Ribonucleoside biosynthetic process	9	4	211	0.3599
GO:0072522	IPI00686225;IPI00726650;IPI00883375;IPI01028487	Biological Process	Purine-containing compound biosynthetic process	7	4	211	0.3599
GO:1901659	IPI00686225;IPI00726650;IPI00883375;IPI01028487	Biological Process	Glycosyl compound biosynthetic process	6	4	211	0.3599

GO:0010604	IPI00689750;IPI00690446;IPI00691963;IPI00693338;IPI00694504;IPI00694851;IPI00699355;IPI00701698;IPI00703776;IPI00704728;IPI00705463;IPI00716158;IPI00717759;IPI00842934;IPI00912603;IPI01018577	Biological Process	Positive regulation of macromolecule metabolic process	6	16	211	0.3630
GO:0001508	IPI00699355;IPI00707359	Biological Process	Action potential	6	2	211	0.3669
GO:0007162	IPI00693338;IPI00701166	Biological Process	Negative regulation of cell adhesion	5	2	211	0.3669
GO:0010769	IPI00687601;IPI00701698	Biological Process	Regulation of cell morphogenesis involved in differentiation	9	2	211	0.3669
GO:0010951	IPI00715354;IPI00871133	Biological Process	Negative regulation of endopeptidase activity	11	2	211	0.3669
GO:0030323	IPI00697184;IPI00707359	Biological Process	Respiratory tube development	7	2	211	0.3669
GO:0030324	IPI00697184;IPI00707359	Biological Process	Lung development	8	2	211	0.3669
GO:0048871	IPI00690160;IPI00697184	Biological Process	Multicellular organismal homeostasis	5	2	211	0.3669
GO:0050680	IPI00699355;IPI00716121	Biological Process	Negative regulation of epithelial cell proliferation	6	2	211	0.3669
GO:0060541	IPI00697184;IPI00707359	Biological Process	Respiratory system development	7	2	211	0.3669

GO:0019222	IPI00685278;IPI00686225;IPI00686601;IPI00686966;IPI00687625;IPI00689750;IPI00690001;IPI00690446;IPI00691963;IPI00692468;IPI00692676;IPI00692911;IPI00693338;IPI00694214;IPI00694504;IPI00694751;IPI00694851;IPI00695331;IPI00695489;IPI00695890;IPI00695965;IPI00696930;IPI00699355;IPI00700542;IPI00700789;IPI00701698;IPI00703268;IPI00703731;IPI00703753;IPI00703776;IPI00704728;IPI00705463;IPI00715339;IPI00715354;IPI00716121;IPI00716158;IPI00717759;IPI00718311;IPI00732368;IPI00842934;IPI00871133;IP	Biological Process	Regulation of metabolic process	4	44	211	0.3698
GO:0008283	IPI00693338;IPI00695666;IPI00699355;IPI00703854;IPI00715354	Biological Process	Cell proliferation	4	5	211	0.3713
GO:0043408	IPI00693338;IPI00694739;IPI00703776;IPI00707359;IPI00718311	Biological Process	Regulation of MAPK cascade	10	5	211	0.3713

GO:2000112	IPI00689750;IPI00690446;IPI00691963;IPI00692468;IPI00692676;IPI00692911;IPI00694214;IPI00694851;IPI00695331;IPI00696930;IPI00699355;IPI00700542;IPI00701698;IPI00703731;IPI00703753;IPI00703776;IPI00704728;IPI00705463;IPI00716158;IPI00842934;IPI00912603;IPI01018577	Biological Process	Regulation of cellular macromolecule biosynthetic process	7	22	211	0.3719
GO:0034641	IPI00685278;IPI00686225;IPI00686803;IPI00687539;IPI00689323;IPI00690232;IPI00692468;IPI00692676;IPI00692911;IPI00694214;IPI00694312;IPI00694851;IPI00695600;IPI00695890;IPI00695965;IPI00697891;IPI00698039;IPI00699107;IPI00700295;IPI00700547;IPI00701698;IPI00703753;IPI00703776;IPI00703854;IPI00705000;IPI00706942;IPI00708018;IPI00711759;IPI00712671;IPI00712775;IPI00713642;IPI00716555;IPI00717759;IPI00726650;IPI00728768;IPI00742596;IPI00883375;IPI01018577;IPI01028487	Biological Process	Cellular nitrogen compound metabolic process	5	39	211	0.3737

GO:0006066	IPI00686601;IPI00692819;IPI00695965;IPI00944429	Biological Process	Alcohol metabolic process	6	4	211	0.3741
GO:0030155	IPI00693338;IPI00699798;IPI00701166;IPI00701698	Biological Process	Regulation of cell adhesion	5	4	211	0.3741
GO:0008202	IPI00686601;IPI00695965;IPI00944429	Biological Process	Steroid metabolic process	6	3	211	0.3743
GO:0061564	IPI00687372;IPI00687601;IPI00694504	Biological Process	Axon development	7	3	211	0.3743
GO:0006082	IPI00688608;IPI00692468;IPI00694312;IPI00694739;IPI00696912;IPI00697081;IPI00699355;IPI00702650;IPI00703753;IPI00704728;IPI00711419;IPI00712671;IPI00742596; IPI00842934;IPI00883375	Biological Process	Organic acid metabolic process	5	15	211	0.3753
GO:0045893	IPI00689750;IPI00694851;IPI00699355;IPI00703776;IPI00705463;IPI00716158;IPI00842934;IPI01018577	Biological Process	Positive regulation of transcription, DNA-templated	12	8	211	0.3779
GO:0045944	IPI00689750;IPI00694851;IPI00703776;IPI00705463;IPI00716158;IPI00842934	Biological Process	Positive regulation of transcription from RNA polymerase II promoter	13	6	211	0.3787
GO:0051094	IPI00687601;IPI00689750;IPI00693338;IPI00701698;IPI00706141;IPI00716121	Biological Process	Positive regulation of developmental process	5	6	211	0.3787

GO:0046483	IPI00685278;IPI00686225;IPI00686803;IPI00687539;IPI00689323;IPI00690232;IPI00692676;IPI00692911;IPI00694214;IPI00694312;IPI00694851;IPI00695600;IPI00695890;IPI00697891;IPI00698039;IPI00699107;IPI00700295;IPI00700547;IPI00701698;IPI00703753;IPI00703776;IPI00703854;IPI00705000;IPI00706942;IPI00708018;IPI00712671;IPI00712775;IPI00713642;IPI00716555;IPI00717759;IPI00726650;IPI00728768;IPI00742596;IPI00883375;IPI01018577;IPI01028487	Biological Process	Heterocycle metabolic process	5	36	211	0.3804
GO:0009790	IPI00689228;IPI00694204;IPI00697184;IPI00700295;IPI00701790;IPI00703854;IPI00706942;IPI00712671;IPI00847093;IPI01018577	Biological Process	Embryo development	6	10	211	0.3834

GO:0006725	IPI00685278;IPI00686225;IPI00686803;IPI00687539;IPI00689323;IPI00690232;IPI00692676;IPI00692911;IPI00694214;IPI00694312;IPI00694851;IPI00695600;IPI00695890;IPI00697891;IPI00698039;IPI00699107;IPI00700295;IPI00700547;IPI00701698;IPI00703753;IPI00703776;IPI00703854;IPI00705000;IPI00706942;IPI00708018;IPI00712671;IPI00712775;IPI00713642;IPI00716555;IPI00717759;IPI00726650;IPI00728768;IPI00742596;IPI00883375;IPI01018577;IPI01028487	Biological Process	Cellular aromatic compound metabolic process	5	36	211	0.3849
GO:0009889	IPI00689750;IPI00690001;IPI00690446;IPI00691963;IPI00692468;IPI00692676;IPI00692911;IPI00694214;IPI00694851;IPI00695331;IPI00695965;IPI00696930;IPI00699355;IPI00700542;IPI00701698;IPI00703731;IPI00703753;IPI00703776;IPI00704728;IPI00705463;IPI00716158;IPI00842934;IPI00912603;IPI01018577	Biological Process	Regulation of biosynthetic process	5	24	211	0.3880

GO:0006873	IPI00687842;IPI00693338;IPI00701166;IPI00703753	Biological Process	Cellular ion homeostasis	7	4	211	0.3881
GO:0001101	IPI00685792;IPI00713573	Biological Process	Response to acid chemical	5	2	211	0.3891
GO:0006606	IPI00689750;IPI00707359	Biological Process	Protein import into nucleus	6	2	211	0.3891
GO:0008203	IPI00695965;IPI00944429	Biological Process	Cholesterol metabolic process	8	2	211	0.3891
GO:0010466	IPI00715354;IPI00871133	Biological Process	Negative regulation of peptidase activity	10	2	211	0.3891
GO:0044744	IPI00689750;IPI00707359	Biological Process	Protein targeting to nucleus	7	2	211	0.3891
GO:0045834	IPI00686601;IPI00695965	Biological Process	Positive regulation of lipid metabolic process	7	2	211	0.3891
GO:0050684	IPI00701698;IPI00717759	Biological Process	Regulation of mRNA processing	11	2	211	0.3891
GO:0051170	IPI00689750;IPI00707359	Biological Process	Nuclear import	9	2	211	0.3891
GO:0072001	IPI00697184;IPI00703547	Biological Process	Rrenal system development	8	2	211	0.3891
GO:0072659	IPI00686601;IPI00708921	Biological Process	Protein localization to plasma membrane	6	2	211	0.3891
GO:0097193	IPI00686966;IPI00715354	Biological Process	Intrinsic apoptotic signaling pathway	8	2	211	0.3891
GO:0051252	IPI00685278;IPI00689750;IPI00690446;IPI00691963;IPI00692468;IPI00692676;IPI00692911;IPI00694851;IPI00695331;IPI00696930;IPI00699355;IPI00700542;IPI00701698;IPI00703731;IPI00703776;IPI00705463;IPI00716158;IPI00717759;IPI00842934;IPI01018577	Biological Process	Regulation of RNA metabolic process	9	20	211	0.3893

GO:0008380	IPI00685278;IPI00690232;IPI00695600;IPI00700295;IPI00701698;IPI00717759	Biological Process	RNA splicing	10	6	211	0.3898
GO:0001501	IPI00693338;IPI00697184;IPI00730144	Biological Process	Skeletal system development	7	3	211	0.3912
GO:0045892	IPI00691963;IPI00692676;IPI00699355;IPI00701698;IPI00703731;IPI00842934;IPI01018577	Biological Process	Negative regulation of transcription, DNA-templated	12	7	211	0.3939
GO:0000018	IPI00693338	Biological Process	Regulation of DNA recombination	10	1	211	0.3940
GO:0001578	IPI00687601	Biological Process	Microtubule bundle formation	7	1	211	0.3940
GO:0002028	IPI00688651	Biological Process	Regulation of sodium ion transport	9	1	211	0.3940
GO:0002377	IPI00693338	Biological Process	Immunoglobulin production	5	1	211	0.3940
GO:0002440	IPI00693338	Biological Process	Production of molecular mediator of immune response	4	1	211	0.3940
GO:0002688	IPI00701698	Biological Process	Regulation of leukocyte chemotaxis	6	1	211	0.3940
GO:0002709	IPI00693338	Biological Process	Regulation of T cell mediated immunity	8	1	211	0.3940
GO:0002711	IPI00693338	Biological Process	Positive regulation of T cell mediated immunity	9	1	211	0.3940
GO:0006098	IPI00706942	Biological Process	Pentose-phosphate shunt	14	1	211	0.3940
GO:0006188	IPI00686225	Biological Process	IMP biosynthetic process	12	1	211	0.3940
GO:0006206	IPI00687539	Biological Process	Pyrimidine nucleobase metabolic process	9	1	211	0.3940
GO:0006448	IPI00704728	Biological Process	Regulation of translational elongation	9	1	211	0.3940
GO:0006740	IPI00706942	Biological Process	NADPH regeneration	13	1	211	0.3940
GO:0007528	IPI00694504	Biological Process	Neuromuscular junction development	6	1	211	0.3940
GO:0008630	IPI00715354	Biological Process	Intrinsic apoptotic signaling pathway in response to DNA damage	9	1	211	0.3940
GO:0009306	IPI00702891	Biological Process	Protein secretion	6	1	211	0.3940
GO:0010822	IPI00701698	Biological Process	Positive regulation of mitochondrion organization	7	1	211	0.3940
GO:0014068	IPI00699355	Biological Process	Positive regulation of phosphatidylinositol 3-kinase signaling	12	1	211	0.3940
GO:0016049	IPI00687601	Biological Process	Cell growth	5	1	211	0.3940
GO:0031529	IPI00842934	Biological Process	Ruffle organization	6	1	211	0.3940
GO:0031623	IPI00703776	Biological Process	Receptor internalization	7	1	211	0.3940

GO:0032092	IPI00703776	Biological Process	Positive regulation of protein binding	7	1	211	0.3940
GO:0032943	IPI00693338	Biological Process	Mononuclear cell proliferation	6	1	211	0.3940
GO:0033619	IPI00707359	Biological Process	Membrane protein proteolysis	7	1	211	0.3940
GO:0034644	IPI00703731	Biological Process	Cellular response to UV	8	1	211	0.3940
GO:0042743	IPI00695965	Biological Process	Hydrogen peroxide metabolic process	6	1	211	0.3940
GO:0043043	IPI00694312	Biological Process	Peptide biosynthetic process	8	1	211	0.3940
GO:0043627	IPI00694204	Biological Process	Response to estrogen	7	1	211	0.3940
GO:0044770	IPI00688651	Biological Process	Cell cycle phase transition	7	1	211	0.3940
GO:0044772	IPI00688651	Biological Process	Mitotic cell cycle phase transition	7	1	211	0.3940
GO:0046040	IPI00686225	Biological Process	IMP metabolic process	12	1	211	0.3940
GO:0046651	IPI00693338	Biological Process	Lymphocyte proliferation	6	1	211	0.3940
GO:0048025	IPI00701698	Biological Process	Negative regulation of mRNA splicing, via spliceosome	13	1	211	0.3940
GO:0048873	IPI00690160	Biological Process	Homeostasis of number of cells within a tissue	7	1	211	0.3940
GO:0050685	IPI00717759	Biological Process	Positive regulation of mRNA processing	12	1	211	0.3940
GO:0050686	IPI00701698	Biological Process	Negative regulation of mRNA processing	12	1	211	0.3940
GO:0050729	IPI00694751	Biological Process	Positive regulation of inflammatory response	8	1	211	0.3940
GO:0050770	IPI00687601	Biological Process	Regulation of axonogenesis	9	1	211	0.3940
GO:0050994	IPI00695965	Biological Process	Regulation of lipid catabolic process	7	1	211	0.3940
GO:0051329	IPI00688651	Biological Process	Mitotic interphase	7	1	211	0.3940
GO:0051489	IPI00687539	Biological Process	Regulation of filopodium assembly	8	1	211	0.3940
GO:0051881	IPI00692468	Biological Process	Regulation of mitochondrial membrane potential	6	1	211	0.3940
GO:0051897	IPI00701698	Biological Process	Positive regulation of protein kinase B signaling	10	1	211	0.3940
GO:0055081	IPI00703753	Biological Process	Anion homeostasis	8	1	211	0.3940
GO:0060021	IPI00702620	Biological Process	Palate development	5	1	211	0.3940
GO:0070661	IPI00693338	Biological Process	Leukocyte proliferation	5	1	211	0.3940
GO:0072006	IPI00703547	Biological Process	Nephron development	10	1	211	0.3940
GO:1901070	IPI00686225	Biological Process	Guanosine-containing compound biosynthetic process	11	1	211	0.3940
GO:0008284	IPI00690160;IPI00691963;IPI00693338;IPI00694504;IPI00699355	Biological Process	Positive regulation of cell proliferation	5	5	211	0.3960

GO:0003008	IPI00688489;IPI00688651;IPI00692468;IPI00697184;IPI00697196;IPI00699355;IPI00701166;IPI00707359;IPI00839134	Biological Process	System process	5	9	211	0.3988
GO:1901566	IPI00686225;IPI00694312;IPI00694739;IPI00704728;IPI00711759;IPI00726650;IPI00742596;IPI00883375;IPI01028487	Biological Process	Organonitrogen compound biosynthetic process	6	9	211	0.4077
GO:0006575	IPI00692468;IPI00704728;IPI00742596	Biological Process	Cellular modified amino acid metabolic process	5	3	211	0.4079
GO:0009127	IPI00686225;IPI00726650;IPI01028487	Biological Process	Purine nucleoside monophosphate biosynthetic process	10	3	211	0.4079
GO:0009168	IPI00686225;IPI00726650;IPI01028487	Biological Process	Purine ribonucleoside monophosphate biosynthetic process	11	3	211	0.4079
GO:0030335	IPI00690160;IPI00693338;IPI00701698	Biological Process	Positive regulation of cell migration	9	3	211	0.4079
GO:0042391	IPI00692468;IPI00699355;IPI00707359	Biological Process	Regulation of membrane potential	5	3	211	0.4079
GO:0006355	IPI00689750;IPI00690446;IPI00691963;IPI00692468;IPI00692676;IPI00692911;IPI00694851;IPI00695331;IPI00696930;IPI00699355;IPI00700542;IPI00701698;IPI00703731; IPI00703776;IPI00705463;IPI00716158;IPI00842934;IPI01018577	Biological Process	Rregulation of transcription, DNA-templated	11	18	211	0.4082

GO:0016053	IPI00694739;IPI00704728;IPI00712671;IPI00742596;IPI00883375	Biological Process	Organic acid biosynthetic process	6	5	211	0.4083
GO:0018193	IPI00688608;IPI00688651;IPI00696930;IPI00697081;IPI00704728	Biological Process	Peptidyl-amino acid modification	8	5	211	0.4083
GO:0046394	IPI00694739;IPI00704728;IPI00712671;IPI00742596;IPI00883375	Biological Process	Carboxylic acid biosynthetic process	7	5	211	0.4083
GO:0048583	IPI00686841;IPI00686966;IPI00688651;IPI00690094;IPI00691963;IPI00693338;IPI00694739;IPI00694751;IPI00697757;IPI00699355;IPI00700789;IPI00701166;IPI00701698;IPI00703753;IPI00703776;IPI00707101;IPI00707359;IPI00713757;IPI00715339;IPI00717119;IPI00718311;IPI00871133;IPI00912603;IPI01018577	Biological Process	Regulation of response to stimulus	4	24	211	0.4097

GO:0044763	IPI00685792;IPI00686601;IPI00686966;IPI00687372;IPI00687539;IPI00687601;IPI00687657;IPI00687842;IPI00688489;IPI00688651;IPI00688921;IPI00689035;IPI00689228;IPI00689325;IPI00689750;IPI00689760;IPI00690094;IPI00690160;IPI00690446;IPI00691963;IPI00692468;IPI00692676;IPI00692819;IPI00692911;IPI00693338;IPI00693628;IPI00694204;IPI00694214;IPI00694504;IPI00694580;IPI00694739;IPI00694751;IPI00694851;IPI00695506;IPI00695666;IPI00695890;IPI00695965;IPI00698039;IPI00698900;IPI00699002;IPI00699355;IP	Biological Process	Single-organism cellular process	4	89	211	0.4100
GO:0007600	IPI00697184;IPI00839134	Biological Process	Sensory perception	7	2	211	0.4109
GO:0015711	IPI00695965;IPI00699355	Biological Process	Organic anion transport	7	2	211	0.4109
GO:0016125	IPI00695965;IPI00944429	Biological Process	Sterol metabolic process	7	2	211	0.4109
GO:0042063	IPI00689228;IPI00707359	Biological Process	Gliogenesis	8	2	211	0.4109
GO:0016310	IPI00688651;IPI00692819;IPI00696930;IPI00711233;IPI01018577;IPI01028487	Biological Process	Phosphorylation	7	6	211	0.4120

GO:0065008	IPI00686601;IPI00687601;IPI00687625;IPI00687842;IPI00688921;IPI00689325;IPI00690160;IPI00691963;IPI00692468;IPI00692627;IPI00693338;IPI00694312;IPI00694580;IPI00695666;IPI00695965;IPI00697184;IPI00697196;IPI00699355;IPI00701166;IPI00701223;IPI00703753;IPI00706141;IPI00707359;IPI00711750;IPI00730144	Biological Process	Regulation of biological quality	4	25	211	0.4142
GO:0072521	IPI00686225;IPI00703753;IPI00712775;IPI00716555;IPI00726650;IPI00883375;IPI01028487	Biological Process	Purine-containing compound metabolic process	6	7	211	0.4143
GO:2001141	IPI00689750;IPI00690446;IPI00691963;IPI00692468;IPI00692676;IPI00692911;IPI00694851;IPI00695331;IPI00696930;IPI00699355;IPI00700542;IPI00701698;IPI00703731;IPI00703776;IPI00705463;IPI00716158;IPI00842934;IPI01018577	Biological Process	Regulation of RNA biosynthetic process	9	18	211	0.4144

GO:2000113	IPI00691963;IPI00692676;IPI00699355;IPI00701698;IPI00703731;IPI00703753;IPI00842934;IPI01018577	Biological Process	Negative regulation of cellular macromolecule biosynthetic process	8	8	211	0.4158
GO:0055114	IPI00692468;IPI00694312;IPI00699355;IPI00699798;IPI00702650;IPI00706942;IPI00707559;IPI00708438;IPI00711233;IPI00712677	Biological Process	Oxidation-reduction process	5	10	211	0.4171
GO:0007399	IPI00686966;IPI00687372;IPI00687539;IPI00687601;IPI00689228;IPI00691963;IPI00692468;IPI00694504;IPI00695666;IPI00699355;IPI00707359;IPI00716121	Biological Process	Nervous system development	7	12	211	0.4172
GO:0007005	IPI00686966;IPI00692468;IPI00695890;IPI00715354;IPI00842934	Biological Process	Mitochondrion organization	5	5	211	0.4206
GO:0048666	IPI00686966;IPI00687372;IPI00687601;IPI00692468;IPI00694504	Biological Process	Neuron development	10	5	211	0.4206
GO:0046649	IPI00690094;IPI00693338;IPI00695506	Biological Process	Lymphocyte activation	5	3	211	0.4245
GO:0009890	IPI00690001;IPI00691963;IPI00692676;IPI00699355;IPI00701698;IPI00703731;IPI00703753;IPI00842934;IPI01018577	Biological Process	Negative regulation of biosynthetic process	6	9	211	0.4255

GO:0031326	IPI00689750;IPI00690446;IPI00691963;IPI00692468;IPI00692676;IPI00692911;IPI00694214;IPI00694851;IPI00695331;IPI00695965;IPI00696930;IPI00699355;IPI00700542;IPI00701698;IPI00703731;IPI00703753;IPI00703776;IPI00704728;IPI00705463;IPI00716158;IPI00842934;IPI00912603;IPI01018577	Biological Process	Regulation of cellular biosynthetic process	6	23	211	0.4273
GO:0010556	IPI00689750;IPI00690446;IPI00691963;IPI00692468;IPI00692676;IPI00692911;IPI00694214;IPI00694851;IPI00695331;IPI00696930;IPI00699355;IPI00700542;IPI00701698;IPI00703731;IPI00703753;IPI00703776;IPI00704728;IPI00705463;IPI00716158;IPI00842934;IPI00912603;IPI01018577	Biological Process	Regulation of macromolecule biosynthetic process	7	22	211	0.4285

GO:0009653	IPI00687372;IPI00687601;IPI00688489;IPI00689035;IPI00692676;IPI00694204;IPI00694504;IPI00695890;IPI00697184;IPI00698900;IPI00703547;IPI00707359;IPI00712671;IPI00714673;IPI00840588;IPI01018577	Biological Process	Anatomical structure morphogenesis	4	16	211	0.4290
GO:0000904	IPI00687372;IPI00687601;IPI00714673;IPI01018577	Biological Process	Cell morphogenesis involved in differentiation	8	4	211	0.4301
GO:0014070	IPI00694204;IPI00695965;IPI00742596;IPI00944429	Biological Process	Response to organic cyclic compound	6	4	211	0.4301
GO:0055082	IPI00687842;IPI00693338;IPI00701166;IPI00703753	Biological Process	Cellular chemical homeostasis	6	4	211	0.4301
GO:0000381	IPI00717759	Biological Process	Regulation of alternative mRNA splicing, via spliceosome	13	1	211	0.4308
GO:0001942	IPI00699355	Biological Process	Hair follicle development	10	1	211	0.4308
GO:0001960	IPI00693338	Biological Process	Negative regulation of cytokine-mediated signaling pathway	10	1	211	0.4308
GO:0002474	IPI00691963	Biological Process	Antigen processing and presentation of peptide antigen via MHC class I	6	1	211	0.4308
GO:0006084	IPI00696912	Biological Process	Acetyl-CoA metabolic process	8	1	211	0.4308
GO:0006414	IPI00704728	Biological Process	Translational elongation	8	1	211	0.4308
GO:0006611	IPI00691963	Biological Process	Protein export from nucleus	6	1	211	0.4308
GO:0006826	IPI00687842	Biological Process	Iron ion transport	9	1	211	0.4308
GO:0006941	IPI00688651	Biological Process	Striated muscle contraction	8	1	211	0.4308
GO:0007031	IPI00686601	Biological Process	Peroxisome organization	5	1	211	0.4308
GO:0007126	IPI00701223	Biological Process	Meiotic nuclear division	5	1	211	0.4308
GO:0007212	IPI00716843	Biological Process	Dopamine receptor signaling pathway	9	1	211	0.4308
GO:0007422	IPI00707359	Biological Process	Peripheral nervous system development	8	1	211	0.4308
GO:0008154	IPI00687657	Biological Process	Actin polymerization or depolymerization	8	1	211	0.4308
GO:0016525	IPI00716121	Biological Process	Negative regulation of angiogenesis	11	1	211	0.4308

GO:0019048	IPI00707359	Biological Process	Modulation by virus of host morphology or physiology	6	1	211	0.4308
GO:0022404	IPI00699355	Biological Process	Molting cycle process	5	1	211	0.4308
GO:0022405	IPI00699355	Biological Process	Hair cycle process	6	1	211	0.4308
GO:0030195	IPI00701166	Biological Process	Negative regulation of blood coagulation	8	1	211	0.4308
GO:0031110	IPI00904732	Biological Process	Regulation of microtubule polymerization or depolymerization	8	1	211	0.4308
GO:0032411	IPI00695508	Biological Process	Positive regulation of transporter activity	8	1	211	0.4308
GO:0032465	IPI00695508	Biological Process	Regulation of cytokinesis	8	1	211	0.4308
GO:0032675	IPI00703776	Biological Process	Regulation of interleukin-6 production	7	1	211	0.4308
GO:0032677	IPI00703776	Biological Process	Regulation of interleukin-8 production	7	1	211	0.4308
GO:0033143	IPI00691963	Biological Process	Regulation of intracellular steroid hormone receptor signaling pathway	9	1	211	0.4308
GO:0034446	IPI00714673	Biological Process	Substrate adhesion-dependent cell spreading	9	1	211	0.4308
GO:0042168	IPI00694312	Biological Process	Heme metabolic process	8	1	211	0.4308
GO:0042180	IPI00686601	Biological Process	Cellular ketone metabolic process	5	1	211	0.4308
GO:0042303	IPI00699355	Biological Process	Molting cycle	5	1	211	0.4308
GO:0042633	IPI00699355	Biological Process	Hair cycle	6	1	211	0.4308
GO:0042775	IPI00711233	Biological Process	Mitochondrial ATP synthesis coupled electron transport	15	1	211	0.4308
GO:0043467	IPI00703753	Biological Process	Regulation of generation of precursor metabolites and energy	6	1	211	0.4308
GO:0043489	IPI00687625	Biological Process	RNA stabilization	10	1	211	0.4308
GO:0044236	IPI00695965	Biological Process	Multicellular organismal metabolic process	5	1	211	0.4308
GO:0045428	IPI00692468	Biological Process	Regulation of nitric oxide biosynthetic process	8	1	211	0.4308
GO:0046112	IPI00686225	Biological Process	Nucleobase biosynthetic process	9	1	211	0.4308
GO:0046164	IPI00692819	Biological Process	Alcohol catabolic process	7	1	211	0.4308
GO:0046470	IPI00695965	Biological Process	Phosphatidylcholine metabolic process	9	1	211	0.4308
GO:0048002	IPI00691963	Biological Process	Antigen processing and presentation of peptide antigen	5	1	211	0.4308
GO:0048255	IPI00687625	Biological Process	mRNA stabilization	11	1	211	0.4308
GO:0048520	IPI00701698	Biological Process	Positive regulation of behavior	4	1	211	0.4308
GO:0050688	IPI00701698	Biological Process	Regulation of defense response to virus	6	1	211	0.4308
GO:0050728	IPI00697757	Biological Process	Negative regulation of inflammatory response	8	1	211	0.4308
GO:0050921	IPI00701698	Biological Process	Positive regulation of chemotaxis	5	1	211	0.4308
GO:0051250	IPI00912603	Biological Process	Negative regulation of lymphocyte activation	7	1	211	0.4308

GO:0055008	IPI00688489	Biological Process	Cardiac muscle tissue morphogenesis	10	1	211	0.4308
GO:0060761	IPI00693338	Biological Process	Negative regulation of response to cytokine stimulus	8	1	211	0.4308
GO:0070584	IPI00695890	Biological Process	Mitochondrion morphogenesis	6	1	211	0.4308
GO:0071453	IPI00689750	Biological Process	Cellular response to oxygen levels	6	1	211	0.4308
GO:0071482	IPI00703731	Biological Process	Cellular response to light stimulus	7	1	211	0.4308
GO:0071825	IPI00695965	Biological Process	Protein-lipid complex subunit organization	6	1	211	0.4308
GO:0071827	IPI00695965	Biological Process	Plasma lipoprotein particle organization	5	1	211	0.4308
GO:0072091	IPI00693338	Biological Process	Regulation of stem cell proliferation	6	1	211	0.4308
GO:1900047	IPI00701166	Biological Process	Negative regulation of hemostasis	8	1	211	0.4308
GO:2000377	IPI00715339	Biological Process	Regulation of reactive oxygen species metabolic process	6	1	211	0.4308
GO:2001022	IPI01018577	Biological Process	Positive regulation of response to DNA damage stimulus	8	1	211	0.4308
GO:0000302	IPI00692468;IPI00695965	Biological Process	Response to reactive oxygen species	6	2	211	0.4324
GO:0042110	IPI00693338;IPI00695506	Biological Process	T cell activation	6	2	211	0.4324
GO:0045088	IPI00686841;IPI00871133	Biological Process	Regulation of innate immune response	6	2	211	0.4324
GO:0046777	IPI00688651;IPI01018577	Biological Process	Protein autophosphorylation	9	2	211	0.4324
GO:0060249	IPI00690160;IPI00697184	Biological Process	Anatomical structure homeostasis	6	2	211	0.4324
GO:1901069	IPI00686225;IPI00712775	Biological Process	Guanosine-containing compound catabolic process	11	2	211	0.4324
GO:0046128	IPI00686225;IPI00703753;IPI00712775;IPI00726650;IPI00883375;IPI01028487	Biological Process	Purine ribonucleoside metabolic process	10	6	211	0.4341
GO:0050877	IPI00692468;IPI00697184;IPI00697196;IPI00699355;IPI00707359;IPI00839134	Biological Process	Neurological system process	6	6	211	0.4341

GO:0009117	IPI00686225;IPI00689323;IPI00703753;IPI00706942;IPI00712671;IPI00712775;IPI00726650;IPI00742596;IPI01028487	Biological Process	Nucleotide metabolic process	9	9	211	0.4344
GO:0010605	IPI00690446;IPI00691963;IPI00692676;IPI00693338;IPI00695965;IPI00699355;IPI00701698;IPI00703731;IPI00703753;IPI00703776;IPI00842934;IPI00871133;IPI01018577	Biological Process	Negative regulation of macromolecule metabolic process	6	13	211	0.4391
GO:0034654	IPI00686225;IPI00689323;IPI00692676;IPI00692911;IPI00694851;IPI00701698;IPI00703776;IPI00712671;IPI00726650;IPI00742596;IPI00883375;IPI01028487	Biological Process	Nucleobase-containing compound biosynthetic process	7	12	211	0.4402
GO:0002253	IPI00693338;IPI00701698;IPI00713757	Biological Process	Activation of immune response	4	3	211	0.4409
GO:0006753	IPI00686225;IPI00689323;IPI00703753;IPI00706942;IPI00712671;IPI00712775;IPI00726650;IPI00742596;IPI01028487	Biological Process	Nucleoside phosphate metabolic process	8	9	211	0.4433

GO:0051246	IPI00690446;IPI00693338;IPI00694214;IPI00695489;IPI00695965;IPI00701698;IPI00703753;IPI00703776;IPI00704728;IPI00715354;IPI00716121;IPI00732368;IPI00842934;IPI00871133;IPI00912603	Biological Process	Regulation of protein metabolic process	6	15	211	0.4437
GO:0051707	IPI00686841;IPI00687625;IPI00691669;IPI00693338;IPI00718757	Biological Process	Response to other organism	6	5	211	0.4450
GO:0009892	IPI00690001;IPI00690446;IPI00691963;IPI00692676;IPI00693338;IPI00695965;IPI00699355;IPI00701698;IPI00703731;IPI00703753;IPI00703776;IPI00842934;IPI00871133;IPI01018577	Biological Process	Negative regulation of metabolic process	4	14	211	0.4451
GO:0042278	IPI00686225;IPI00703753;IPI00712775;IPI00726650;IPI00883375;IPI01028487	Biological Process	Purine nucleoside metabolic process	9	6	211	0.4451
GO:0044255	IPI00692627;IPI00692819;IPI00694312;IPI00695965;IPI00697184;IPI00699355;IPI00702650;IPI00718311;IPI00842934;IPI00944429	Biological Process	Cellular lipid metabolic process	5	10	211	0.4508
GO:0030098	IPI00690094;IPI00693338	Biological Process	Lymphocyte differentiation	8	2	211	0.4534
GO:0070482	IPI00689750;IPI00692468	Biological Process	Response to oxygen levels	5	2	211	0.4534

GO:0072358	IPI00688489;IPI00689035;IPI00689750;IPI00692468;IPI00692676;IPI00697184;IPI00840588	Biological Process	Cardiovascular system development	7	7	211	0.4549
GO:0072359	IPI00688489;IPI00689035;IPI00689750;IPI00692468;IPI00692676;IPI00697184;IPI00840588	Biological Process	Circulatory system development	7	7	211	0.4549
GO:0002684	IPI00690094;IPI00690160;IPI00693338;IPI00701698;IPI00713757	Biological Process	Positive regulation of immune system process	5	5	211	0.4571
GO:0009607	IPI00686841;IPI00687625;IPI00691669;IPI00693338;IPI00718757	Biological Process	Response to biotic stimulus	4	5	211	0.4571
GO:0009100	IPI00688608;IPI00697081;IPI00700547	Biological Process	Glycoprotein metabolic process	6	3	211	0.4571
GO:0019216	IPI00686601;IPI00695965;IPI00718311	Biological Process	Regulation of lipid metabolic process	6	3	211	0.4571
GO:0051188	IPI00694312;IPI00696912;IPI00742596	Biological Process	Cofactor biosynthetic process	6	3	211	0.4571
GO:0042592	IPI00687842;IPI00689325;IPI00690160;IPI00692468;IPI00693338;IPI00694312;IPI00694580;IPI00695965;IPI00697184;IPI00701166;IPI00703753	Biological Process	Homeostatic process	5	11	211	0.4573
GO:0001889	IPI00692468	Biological Process	Liver development	8	1	211	0.4654
GO:0002260	IPI00690160	Biological Process	Lymphocyte homeostasis	5	1	211	0.4654
GO:0002695	IPI00912603	Biological Process	Negative regulation of leukocyte activation	6	1	211	0.4654
GO:0006352	IPI00694851	Biological Process	DNA-templated transcription, initiation	9	1	211	0.4654
GO:0006458	IPI00698900	Biological Process	De novo protein folding	8	1	211	0.4654
GO:0006544	IPI00742596	Biological Process	Glycine metabolic process	9	1	211	0.4654

GO:0006695	IPI00944429	Biological Process	Cholesterol biosynthetic process	9	1	211	0.4654
GO:0008584	IPI00697184	Biological Process	Male gonad development	8	1	211	0.4654
GO:0010522	IPI00695508	Biological Process	Regulation of calcium ion transport into cytosol	14	1	211	0.4654
GO:0014066	IPI00699355	Biological Process	Regulation of phosphatidylinositol 3-kinase signaling	11	1	211	0.4654
GO:0030512	IPI00717119	Biological Process	Negative regulation of transforming growth factor beta receptor signaling pathway	12	1	211	0.4654
GO:0030518	IPI00694851	Biological Process	Intracellular steroid hormone receptor signaling pathway	8	1	211	0.4654
GO:0030838	IPI00706141	Biological Process	Positive regulation of actin filament polymerization	11	1	211	0.4654
GO:0032526	IPI00697184	Biological Process	Response to retinoic acid	6	1	211	0.4654
GO:0033077	IPI00693338	Biological Process	T cell differentiation in thymus	10	1	211	0.4654
GO:0033119	IPI00701698	Biological Process	Negative regulation of RNA splicing	11	1	211	0.4654
GO:0033344	IPI00695965	Biological Process	Cholesterol efflux	8	1	211	0.4654
GO:0042773	IPI00711233	Biological Process	ATP synthesis coupled electron transport	14	1	211	0.4654
GO:0043001	IPI00708921	Biological Process	Golgi to plasma membrane protein transport	9	1	211	0.4654
GO:0043901	IPI00701698	Biological Process	Negative regulation of multi-organism process	5	1	211	0.4654
GO:0045582	IPI00693338	Biological Process	Positive regulation of T cell differentiation	11	1	211	0.4654
GO:0046825	IPI00715354	Biological Process	Regulation of protein export from nucleus	8	1	211	0.4654
GO:0048015	IPI00701698	Biological Process	Phosphatidylinositol-mediated signaling	9	1	211	0.4654
GO:0048017	IPI00701698	Biological Process	Inositol lipid-mediated signaling	8	1	211	0.4654
GO:0048644	IPI00688489	Biological Process	Muscle organ morphogenesis	9	1	211	0.4654
GO:0048741	IPI00694504	Biological Process	Skeletal muscle fiber development	11	1	211	0.4654
GO:0050852	IPI00693338	Biological Process	T cell receptor signaling pathway	8	1	211	0.4654
GO:0051004	IPI00695965	Biological Process	Regulation of lipoprotein lipase activity	8	1	211	0.4654
GO:0051084	IPI00698900	Biological Process	De novo posttranslational protein folding	9	1	211	0.4654
GO:0060415	IPI00688489	Biological Process	Muscle tissue morphogenesis	10	1	211	0.4654
GO:0061008	IPI00692468	Biological Process	Hepaticobiliary system development	7	1	211	0.4654
GO:1900076	IPI00703753	Biological Process	Regulation of cellular response to insulin stimulus	10	1	211	0.4654

GO:0010468	IPI00685278;IPI00687625;IPI00689750;IPI00690446;IPI00691963;IPI00692468;IPI00692676;IPI00692911;IPI00694214;IPI00694504;IPI00694851;IPI00695331;IPI00696930;IPI00699355;IPI00700542;IPI00701698;IPI00703731;IPI00703776;IPI00704728;IPI00705463;IPI00716158;IPI00717759;IPI00842934;IPI00871133;IPI01018577	Biological Process	Regulation of gene expression	7	25	211	0.4681
GO:0019637	IPI00686225;IPI00686601;IPI00689323;IPI00692819;IPI00695965;IPI00703753;IPI00706942;IPI00712671;IPI00712775;IPI00726650;IPI00742596;IPI00944429;IPI01028487	Biological Process	Organophosphate metabolic process	6	13	211	0.4685
GO:0015980	IPI00692468;IPI00699798;IPI00708438;IPI00711233	Biological Process	Energy derivation by oxidation of organic compounds	6	4	211	0.4713
GO:0006875	IPI00687842;IPI00693338;IPI00701166	Biological Process	Cellular metal ion homeostasis	9	3	211	0.4732
GO:0045333	IPI00692468;IPI00708438;IPI00711233	Biological Process	Cellular respiration	7	3	211	0.4732
GO:0051046	IPI00697184;IPI00703776;IPI00706141	Biological Process	Regulation of secretion	6	3	211	0.4732
GO:0071417	IPI00703753;IPI00713573;IPI00842934	Biological Process	Cellular response to organonitrogen compound	7	3	211	0.4732

GO:0006096	IPI00698589;IPI00706942	Biological Process	Glycolytic process	7	2	211	0.4739
GO:0006874	IPI00693338;IPI00701166	Biological Process	Cellular calcium ion homeostasis	10	2	211	0.4739
GO:0009615	IPI00686841;IPI00693338	Biological Process	Rsponse to virus	7	2	211	0.4739
GO:0030336	IPI00687539;IPI00707359	Biological Process	Negative regulation of cell migration	9	2	211	0.4739
GO:0050794	IPI00685278;IPI00686225;IPI00686601;IPI00686966;IPI00687539;IPI00687601;IPI00687842;IPI00688651;IPI00688921;IPI00689228;IPI00689325;IPI00689750;IPI00690094;IPI00690160;IPI00690446;IPI00691963;IPI00692468;IPI00692676;IPI00692819;IPI00692911;IPI00693338;IPI00694214;IPI00694504;IPI00694580;IPI00694739;IPI00694751;IPI00694851;IPI00695331;IPI00695506;IPI00695508;IPI00695965;IPI00696930;IPI00697184;IPI00698039;IPI00699355;IPI00699798;IPI00700542;IPI00700789;IPI00701166;IPI00701223;IPI00701698;IP	Biological Process	Regulation of cellular process	4	70	211	0.4741
GO:0051247	IPI00690446;IPI00693338;IPI00701698;IPI00703776;IPI00704728;IPI00842934;IPI00912603	Biological Process	Positive regulation of protein metabolic process	7	7	211	0.4750

GO:0044271	IPI00686225;IPI00689323;IPI00692676;IPI00692911;IPI00694312;IPI00694851;IPI00701698;IPI00703776;IPI00711759;IPI00712671;IPI00726650;IPI00742596;IPI00883375;IPI01028487	Biological Process	Cellular nitrogen compound biosynthetic process	6	14	211	0.4805
GO:0010558	IPI00691963;IPI00692676;IPI00699355;IPI00701698;IPI00703731;IPI00703753;IPI00842934;IPI01018577	Biological Process	Negative regulation of macromolecule biosynthetic process	8	8	211	0.4817
GO:0009124	IPI00686225;IPI00726650;IPI01028487	Biological Process	Nucleoside monophosphate biosynthetic process	9	3	211	0.4890
GO:0009156	IPI00686225;IPI00726650;IPI01028487	Biological Process	Ribonucleoside monophosphate biosynthetic process	10	3	211	0.4890
GO:0030162	IPI00695489;IPI00716121;IPI00732368	Biological Process	Regulation of proteolysis	7	3	211	0.4890
GO:0055074	IPI00693338;IPI00701166	Biological Process	Calcium ion homeostasis	10	2	211	0.4940
GO:2000146	IPI00687539;IPI00707359	Biological Process	Negative regulation of cell motility	8	2	211	0.4940
GO:0001776	IPI00690160	Biological Process	Leukocyte homeostasis	4	1	211	0.4979
GO:0002312	IPI00690094	Biological Process	B cell activation involved in immune response	8	1	211	0.4979
GO:0002705	IPI00693338	Biological Process	Positive regulation of leukocyte mediated immunity	7	1	211	0.4979
GO:0002708	IPI00693338	Biological Process	Positive regulation of lymphocyte mediated immunity	8	1	211	0.4979
GO:0002821	IPI00693338	Biological Process	Positive regulation of adaptive immune response	7	1	211	0.4979
GO:0002824	IPI00693338	Biological Process	Positive regulation of adaptive immune response based on somatic recombination of immune receptors built from immunoglobulin superfamily domains	8	1	211	0.4979
GO:0006275	IPI00691963	Biological Process	Regulation of DNA replication	10	1	211	0.4979

GO:0006338	IPI00703854	Biological Process	Chromatin remodeling	8	1	211	0.4979
GO:0006662	IPI00689325	Biological Process	Glycerol ether metabolic process	7	1	211	0.4979
GO:0006893	IPI00708921	Biological Process	Golgi to plasma membrane transport	8	1	211	0.4979
GO:0006984	IPI00689750	Biological Process	ER-nucleus signaling pathway	7	1	211	0.4979
GO:0006998	IPI00689750	Biological Process	Nuclear envelope organization	6	1	211	0.4979
GO:0007044	IPI00714673	Biological Process	Cell-substrate junction assembly	7	1	211	0.4979
GO:0007187	IPI00716843	Biological Process	G-protein coupled receptor signaling pathway, coupled to cyclic nucleotide second messenger	9	1	211	0.4979
GO:0007188	IPI00716843	Biological Process	Aadenylate cyclase-modulating G-protein coupled receptor signaling pathway	10	1	211	0.4979
GO:0007338	IPI00689035	Biological Process	Single fertilization	6	1	211	0.4979
GO:0009451	IPI00699107	Biological Process	RNA modification	9	1	211	0.4979
GO:0015918	IPI00695965	Biological Process	Sterol transport	6	1	211	0.4979
GO:0016126	IPI00944429	Biological Process	Sterol biosynthetic process	8	1	211	0.4979
GO:0016358	IPI00687601	Biological Process	Dendrite development	7	1	211	0.4979
GO:0018105	IPI00688651	Biological Process	Peptidyl-serine phosphorylation	9	1	211	0.4979
GO:0018108	IPI00696930	Biological Process	Peptidyl-tyrosine phosphorylation	9	1	211	0.4979
GO:0018212	IPI00696930	Biological Process	Peptidyl-tyrosine modification	9	1	211	0.4979
GO:0018904	IPI00689325	Biological Process	Ether metabolic process	6	1	211	0.4979
GO:0019217	IPI00695965	Biological Process	Regulation of fatty acid metabolic process	10	1	211	0.4979
GO:0030048	IPI00688489	Biological Process	Actin filament-based movement	6	1	211	0.4979
GO:0030301	IPI00695965	Biological Process	Cholesterol transport	7	1	211	0.4979
GO:0030865	IPI00691963	Biological Process	Cortical cytoskeleton organization	6	1	211	0.4979
GO:0030866	IPI00691963	Biological Process	Cortical actin cytoskeleton organization	7	1	211	0.4979
GO:0031397	IPI00703776	Biological Process	Negative regulation of protein ubiquitination	12	1	211	0.4979
GO:0032412	IPI00695508	Biological Process	Regulation of ion transmembrane transporter activity	8	1	211	0.4979
GO:0042558	IPI00742596	Biological Process	Pteridine-containing compound metabolic process	6	1	211	0.4979
GO:0043154	IPI00715354	Biological Process	Negative regulation of cysteine-type endopeptidase activity involved in apoptotic process	13	1	211	0.4979
GO:0043487	IPI00687625	Biological Process	Regulation of RNA stability	9	1	211	0.4979
GO:0043488	IPI00687625	Biological Process	Regulation of mRNA stability	10	1	211	0.4979

GO:0044003	IPI00707359	Biological Process	Modification by symbiont of host morphology or physiology	6	1	211	0.4979
GO:0045580	IPI00693338	Biological Process	Regulation of T cell differentiation	10	1	211	0.4979
GO:0045621	IPI00693338	Biological Process	Positive regulation of lymphocyte differentiation	10	1	211	0.4979
GO:0045639	IPI00706141	Biological Process	Positive regulation of myeloid cell differentiation	9	1	211	0.4979
GO:0046148	IPI00686225	Biological Process	Pigment biosynthetic process	6	1	211	0.4979
GO:0046545	IPI00697184	Biological Process	Development of primary female sexual characteristics	7	1	211	0.4979
GO:0048706	IPI00697184	Biological Process	Embryonic skeletal system development	9	1	211	0.4979
GO:0048771	IPI00703753	Biological Process	Tissue remodeling	5	1	211	0.4979
GO:0050819	IPI00701166	Biological Process	Negative regulation of coagulation	7	1	211	0.4979
GO:0050866	IPI00912603	Biological Process	Negative regulation of cell activation	7	1	211	0.4979
GO:0060485	IPI01018577	Biological Process	Mesenchyme development	8	1	211	0.4979
GO:0070301	IPI00695965	Biological Process	Cellular response to hydrogen peroxide	8	1	211	0.4979
GO:0071356	IPI00708921	Biological Process	Cellular response to tumor necrosis factor	8	1	211	0.4979
GO:2000117	IPI00715354	Biological Process	Negative regulation of cysteine-type endopeptidase activity	12	1	211	0.4979
GO:2001244	IPI00686966	Biological Process	Positive regulation of intrinsic apoptotic signaling pathway	10	1	211	0.4979
GO:0032270	IPI00690446;IPI00693338;IPI00701698;IPI00703776;IPI00704728;IPI00842934	Biological Process	Positive regulation of cellular protein metabolic process	8	6	211	0.4995
GO:1901362	IPI00686225;IPI00686601;IPI00689323;IPI00692676;IPI00692911;IPI00694851;IPI00701698;IPI00703776;IPI00712671;IPI00726650;IPI00742596;IPI00883375;IPI00944429;IPI01028487	Biological Process	Organic cyclic compound biosynthetic process	6	14	211	0.5015
GO:0003006	IPI00694504;IPI00697184;IPI00718311	Biological Process	Developmental process involved in reproduction	4	3	211	0.5046

GO:0030182	IPI00686966;IPI00687372;IPI00687601;IPI00692468;IPI00694504	Biological Process	Neuron differentiation	9	5	211	0.5048
GO:0009119	IPI00686225;IPI00703753;IPI00712775;IPI00726650;IPI00883375;IPI01028487	Biological Process	Ribonucleoside metabolic process	9	6	211	0.5101
GO:0022603	IPI00687601;IPI00688921;IPI00690160;IPI00701698;IPI00716121;IPI00717119	Biological Process	Regulation of anatomical structure morphogenesis	5	6	211	0.5101
GO:0080090	IPI00685278;IPI00686225;IPI00686601;IPI00689750;IPI00690446;IPI00691963;IPI00692468;IPI00692676;IPI00692911;IPI00693338;IPI00694214;IPI00694851;IPI00695331;IPI00695489;IPI00695965;IPI00696930;IPI00699355;IPI00700542;IPI00701698;IPI00703268;IPI00703731;IPI00703753;IPI00703776;IPI00704728;IPI00705463;IPI00715354;IPI00716121;IPI00716158;IPI00717759;IPI00718311;IPI00732368;IPI00842934;IPI00871133;IPI00912603;IPI01018577	Biological Process	Regulation of primary metabolic process	5	35	211	0.5110
GO:0046434	IPI00686225;IPI00692819;IPI00703753;IPI00712775	Biological Process	Organophosphate catabolic process	6	4	211	0.5113
GO:0007163	IPI00687601;IPI00689750	Biological Process	Establishment or maintenance of cell polarity	5	2	211	0.5137

GO:0007596	IPI00701166;IPI00730144	Biological Process	Blood coagulation	6	2	211	0.5137
GO:0010959	IPI00688651;IPI00695508	Biological Process	Regulation of metal ion transport	8	2	211	0.5137
GO:0032870	IPI00703753;IPI00842934	Biological Process	Cellular response to hormone stimulus	7	2	211	0.5137
GO:0040013	IPI00687539;IPI00707359	Biological Process	Negative regulation of locomotion	5	2	211	0.5137
GO:0050817	IPI00701166;IPI00730144	Biological Process	Coagulation	5	2	211	0.5137
GO:0051028	IPI00690232;IPI00704728	Biological Process	mRNA transport	8	2	211	0.5137
GO:0051098	IPI00685792;IPI00703776	Biological Process	Regulation of binding	5	2	211	0.5137
GO:0072503	IPI00693338;IPI00701166	Biological Process	Cellular divalent inorganic cation homeostasis	9	2	211	0.5137
GO:0090305	IPI00686803;IPI00708018	Biological Process	Nucleic acid phosphodiester bond hydrolysis	8	2	211	0.5137
GO:0051253	IPI00691963;IPI00692676;IPI00699355;IPI00701698;IPI00703731;IPI00842934;IPI01018577	Biological Process	Negative regulation of RNA metabolic process	10	7	211	0.5146
GO:0031324	IPI00690446;IPI00691963;IPI00692676;IPI00693338;IPI00695965;IPI00699355;IPI00701698;IPI00703731;IPI00703753;IPI00703776;IPI00842934;IPI01018577	Biological Process	Negative regulation of cellular metabolic process	6	12	211	0.5162
GO:0031327	IPI00691963;IPI00692676;IPI00699355;IPI00701698;IPI00703731;IPI00703753;IPI00842934;IPI01018577	Biological Process	Negative regulation of cellular biosynthetic process	7	8	211	0.5186
GO:0009152	IPI00686225;IPI00726650;IPI01028487	Biological Process	Purine ribonucleotide biosynthetic process	11	3	211	0.5200

GO:0048610	IPI00694504;IPI00701223;IPI00718311	Biological Process	Cellular process involved in reproduction	NA	3	211	0.5200
GO:1901575	IPI00686225;IPI00687539;IPI00692819;IPI00694312;IPI00695176;IPI00695965;IPI00697757;IPI00698589;IPI00699355;IPI00700547;IPI00701790;IPI00702650;IPI00703753;IPI00703776;IPI00706942;IPI00707875;IPI00711419;IPI00712775;IPI00716555;IPI00842934	Biological Process	Organic substance catabolic process	5	20	211	0.5201
GO:0019725	IPI00687842;IPI00689325;IPI00693338;IPI00694580;IPI00701166;IPI00703753	Biological Process	Cellular homeostasis	5	6	211	0.5207
GO:0090304	IPI00685278;IPI00686803;IPI00690232;IPI00692676;IPI00692911;IPI00694214;IPI00694851;IPI00695600;IPI00695890;IPI00697891;IPI00698039;IPI00699107;IPI00700295;IPI00700547;IPI00701698;IPI00703776;IPI00703854;IPI00705000;IPI00708018;IPI00713642;IPI00717759;IPI00728768;IPI01018577	Biological Process	Nucleic acid metabolic process	7	23	211	0.5222

GO:0010629	IPI00691963;IPI00692676;IPI00699355;IPI00701698;IPI00703731;IPI00842934;IPI01018577	Biological Process	Negative regulation of gene expression	8	7	211	0.5244
GO:1901615	IPI00686601;IPI00692819;IPI00695965;IPI00944429	Biological Process	Organic hydroxy compound metabolic process	5	4	211	0.5244
GO:0009605	IPI00687372;IPI00690160;IPI00694751;IPI00697184;IPI00703776	Biological Process	Response to external stimulus	4	5	211	0.5280
GO:0000187	IPI00693338	Biological Process	Activation of MAPK activity	13	1	211	0.5285
GO:0001816	IPI00912603	Biological Process	Cytokine production	5	1	211	0.5285
GO:0002455	IPI00701698	Biological Process	Humoral immune response mediated by circulating immunoglobulin	9	1	211	0.5285
GO:0006282	IPI01018577	Biological Process	Regulation of DNA repair	10	1	211	0.5285
GO:0006730	IPI00742596	Biological Process	One-carbon metabolic process	5	1	211	0.5285
GO:0006778	IPI00694312	Biological Process	Porphyrin-containing compound metabolic process	7	1	211	0.5285
GO:0006879	IPI00687842	Biological Process	Cellular iron ion homeostasis	11	1	211	0.5285
GO:0006892	IPI00708921	Biological Process	Post-Golgi vesicle-mediated transport	7	1	211	0.5285
GO:0006958	IPI00701698	Biological Process	Complement activation, classical pathway	10	1	211	0.5285
GO:0008277	IPI00703776	Biological Process	Regulation of G-protein coupled receptor protein signaling pathway	9	1	211	0.5285
GO:0010212	IPI00692468	Biological Process	Response to ionizing radiation	6	1	211	0.5285
GO:0010811	IPI00701698	Biological Process	Positive regulation of cell-substrate adhesion	6	1	211	0.5285
GO:0010906	IPI00703753	Biological Process	Regulation of glucose metabolic process	7	1	211	0.5285
GO:0015850	IPI00695965	Biological Process	Organic hydroxy compound transport	5	1	211	0.5285
GO:0022898	IPI00695508	Biological Process	Regulation of transmembrane transporter activity	7	1	211	0.5285
GO:0030010	IPI00687601	Biological Process	Establishment of cell polarity	6	1	211	0.5285
GO:0030032	IPI00688921	Biological Process	Lamellipodium assembly	7	1	211	0.5285
GO:0030199	IPI00707587	Biological Process	Collagen fibril organization	7	1	211	0.5285
GO:0042552	IPI00707359	Biological Process	Myelination	10	1	211	0.5285
GO:0043112	IPI00703776	Biological Process	Receptor metabolic process	6	1	211	0.5285

GO:0043255	IPI00703753	Biological Process	Regulation of carbohydrate biosynthetic process	7	1	211	0.5285
GO:0045787	IPI00691963	Biological Process	Positive regulation of cell cycle	6	1	211	0.5285
GO:0046546	IPI00697184	Biological Process	Development of primary male sexual characteristics	7	1	211	0.5285
GO:0046660	IPI00697184	Biological Process	Female sex differentiation	6	1	211	0.5285
GO:0051321	IPI00701223	Biological Process	Meiotic cell cycle	4	1	211	0.5285
GO:0071230	IPI00713573	Biological Process	Cellular response to amino acid stimulus	7	1	211	0.5285
GO:0071478	IPI00703731	Biological Process	Cellular response to radiation	6	1	211	0.5285
GO:0090101	IPI00717119	Biological Process	Negative regulation of transmembrane receptor protein serine/threonine kinase signaling pathway	11	1	211	0.5285
GO:0097006	IPI00695965	Biological Process	Regulation of plasma lipoprotein particle levels	5	1	211	0.5285
GO:1901616	IPI00692819	Biological Process	Organic hydroxy compound catabolic process	6	1	211	0.5285
GO:0007009	IPI00686601;IPI00708921	Biological Process	Plasma membrane organization	5	2	211	0.5328
GO:0007599	IPI00701166;IPI00730144	Biological Process	Hemostasis	6	2	211	0.5328
GO:0034599	IPI00692468;IPI00695965	Biological Process	Cellular response to oxidative stress	6	2	211	0.5328
GO:0045454	IPI00689325;IPI00694580	Biological Process	Cell redox homeostasis	6	2	211	0.5328
GO:0045637	IPI00698039;IPI00706141	Biological Process	Regulation of myeloid cell differentiation	8	2	211	0.5328
GO:0050678	IPI00699355;IPI00716121	Biological Process	Regulation of epithelial cell proliferation	6	2	211	0.5328
GO:0051222	IPI00697184;IPI00715354	Biological Process	Positive regulation of protein transport	7	2	211	0.5328
GO:0051271	IPI00687539;IPI00707359	Biological Process	Negative regulation of cellular component movement	7	2	211	0.5328
GO:0072507	IPI00693338;IPI00701166	Biological Process	Divalent inorganic cation homeostasis	9	2	211	0.5328
GO:0010564	IPI00691963;IPI00693338;IPI00695508	Biological Process	Regulation of cell cycle process	7	3	211	0.5350
GO:0051051	IPI00687601;IPI00695508;IPI00703753	Biological Process	Negative regulation of transport	7	3	211	0.5350
GO:1901699	IPI00703753;IPI00713573;IPI00842934	Biological Process	Cellular response to nitrogen compound	6	3	211	0.5350

GO:0060255	IPI00685278;IPI00687625;IPI00689750;IPI00690446;IPI00691963;IPI00692468;IPI00692676;IPI00692911;IPI00693338;IPI00694214;IPI00694504;IPI00694851;IPI00695331;IPI00695489;IPI00695965;IPI00696930;IPI00699355;IPI00700542;IPI00701698;IPI00703731;IPI00703753;IPI00703776;IPI00704728;IPI00705463;IPI00715354;IPI00716121;IPI00716158;IPI00717759;IPI00732368;IPI00842934;IPI00871133;IPI00912603;IPI01018577	Biological Process	Regulation of macromolecule metabolic process	6	33	211	0.5393
GO:0016477	IPI00690160;IPI00693338;IPI00693628;IPI00694751;IPI00699355	Biological Process	Cell migration	7	5	211	0.5394
GO:0016071	IPI00685278;IPI00690232;IPI00695600;IPI00697891;IPI00699107;IPI00701698;IPI00717759	Biological Process	mRNA metabolic process	9	7	211	0.5436
GO:0071310	IPI00686966;IPI00687539;IPI00703547;IPI00703753;IPI00708921;IPI00713573;IPI00718311;IPI00842934	Biological Process	Cellular response to organic substance	6	8	211	0.5457

GO:0019438	IPI00686225;IPI00689323;IPI00692676;IPI00692911;IPI00694851;IPI00701698;IPI00703776;IPI00712671;IPI00726650;IPI00742596;IPI00883375;IPI01028487	Biological Process	Aromatic compound biosynthetic process	6	12	211	0.5459
GO:0006164	IPI00686225;IPI00726650;IPI01028487	Biological Process	Purine nucleotide biosynthetic process	10	3	211	0.5499
GO:0030003	IPI00687842;IPI00693338;IPI00701166	Biological Process	Cellular cation homeostasis	8	3	211	0.5499
GO:0030855	IPI00689228;IPI00715354;IPI00718311;IPI00721270	Biological Process	Epithelial cell differentiation	7	4	211	0.5500
GO:0032269	IPI00690446;IPI00693338;IPI00703753;IPI00703776	Biological Process	Negative regulation of cellular protein metabolic process	8	4	211	0.5500
GO:1901701	IPI00692468;IPI00695965;IPI00703753;IPI00713573;IPI00842934	Biological Process	Cellular response to oxygen-containing compound	6	5	211	0.5507
GO:0030100	IPI00691963;IPI00707101	Biological Process	Regulation of endocytosis	6	2	211	0.5513
GO:0006928	IPI00687372;IPI00687601;IPI00688489;IPI00690160;IPI00693338;IPI00693628;IPI00694751;IPI00699355	Biological Process	Cellular component movement	5	8	211	0.5545
GO:0002706	IPI00693338	Biological Process	Regulation of lymphocyte mediated immunity	7	1	211	0.5572
GO:0002819	IPI00693338	Biological Process	Regulation of adaptive immune response	6	1	211	0.5572
GO:0002822	IPI00693338	Biological Process	Regulation of adaptive immune response based on somatic recombination of immune receptors built from immunoglobulin superfamily domains	7	1	211	0.5572
GO:0002831	IPI00701698	Biological Process	Regulation of response to biotic stimulus	5	1	211	0.5572
GO:0006739	IPI00706942	Biological Process	NADP metabolic process	12	1	211	0.5572

GO:0007050	IPI00691963	Biological Process	Cell cycle arrest	7	1	211	0.5572
GO:0008033	IPI00700295	Biological Process	tRNA processing	11	1	211	0.5572
GO:0008344	IPI00686966	Biological Process	Adult locomotory behavior	5	1	211	0.5572
GO:0015849	IPI00699355	Biological Process	Organic acid transport	5	1	211	0.5572
GO:0030217	IPI00693338	Biological Process	T cell differentiation	9	1	211	0.5572
GO:0030433	IPI00700547	Biological Process	ER-associated ubiquitin-dependent protein catabolic process	11	1	211	0.5572
GO:0032273	IPI00706141	Biological Process	Positive regulation of protein polymerization	8	1	211	0.5572
GO:0033013	IPI00694312	Biological Process	Tetrapyrrole metabolic process	6	1	211	0.5572
GO:0033500	IPI00697184	Biological Process	Carbohydrate homeostasis	7	1	211	0.5572
GO:0042593	IPI00697184	Biological Process	Glucose homeostasis	8	1	211	0.5572
GO:0046324	IPI00703753	Biological Process	Regulation of glucose import	10	1	211	0.5572
GO:0046942	IPI00699355	Biological Process	Carboxylic acid transport	8	1	211	0.5572
GO:0050795	IPI00701698	Biological Process	Regulation of behavior	4	1	211	0.5572
GO:0050851	IPI00693338	Biological Process	Antigen receptor-mediated signaling pathway	7	1	211	0.5572
GO:0050920	IPI00701698	Biological Process	Regulation of chemotaxis	5	1	211	0.5572
GO:0051302	IPI00695508	Biological Process	Regulation of cell division	6	1	211	0.5572
GO:0060491	IPI00687539	Biological Process	Regulation of cell projection assembly	7	1	211	0.5572
GO:2000027	IPI00717119	Biological Process	Regulation of organ morphogenesis	9	1	211	0.5572
GO:2001242	IPI00686966	Biological Process	Regulation of intrinsic apoptotic signaling pathway	9	1	211	0.5572
GO:0009116	IPI00686225;IPI00703753;IPI00712775;IPI00726650;IPI00883375;IPI01028487	Biological Process	Nucleoside metabolic process	8	6	211	0.5620
GO:0030030	IPI00687372;IPI00687601;IPI00688921;IPI00690160;IPI00694504;IPI00842934	Biological Process	Cell projection organization	5	6	211	0.5620

GO:0044710	IPI00686225;IPI00686601;IPI00687539;IPI00688608;IPI00689323;IPI00689325;IPI00692468;IPI00692627;IPI00692819;IPI00694312;IPI00694739;IPI00695965;IPI00696912;IPI00697081;IPI00697184;IPI00699355;IPI00699798;IPI00702650;IPI00703753;IPI00704728;IPI00706942;IPI00707559;IPI00708438;IPI00711233;IPI00711419;IPI00712671;IPI00712677;IPI00712775;IPI00716555;IPI00718311;IPI00726650;IPI00742596;IPI00842934;IPI00883375;IPI00944429;IPI01028487	Biological Process	Single-organism metabolic process	4	36	211	0.5632
GO:0016482	IPI00689750;IPI00691963;IPI00692911;IPI00693338;IPI00699002;IPI00705941;IPI00707359;IPI00708921	Biological Process	Cytoplasmic transport	7	8	211	0.5634
GO:0044057	IPI00688651;IPI00695965;IPI00716843	Biological Process	Regulation of system process	6	3	211	0.5644
GO:0045321	IPI00690094;IPI00693338;IPI00695506	Biological Process	Leukocyte activation	4	3	211	0.5644
GO:0072523	IPI00686225;IPI00712775;IPI00716555	Biological Process	Purine-containing compound catabolic process	7	3	211	0.5644

GO:0044248	IPI00686225;IPI00687539;IPI00694312;IPI00694751;IPI00695176;IPI00695965;IPI00697757;IPI00699355;IPI00700547;IPI00702650;IPI00703753;IPI00703776;IPI00707875;IPI00711419;IPI00712775;IPI00716555;IPI00842934	Biological Process	Cellular catabolic process	5	17	211	0.5679
GO:0010498	IPI00700547;IPI00703776	Biological Process	Proteasomal protein catabolic process	9	2	211	0.5694
GO:0017038	IPI00689750;IPI00707359	Biological Process	Protein import	5	2	211	0.5694
GO:0032388	IPI00686601;IPI00715354	Biological Process	Positive regulation of intracellular transport	7	2	211	0.5694
GO:0043161	IPI00700547;IPI00703776	Biological Process	Proteasome-mediated ubiquitin-dependent protein catabolic process	10	2	211	0.5694
GO:0043410	IPI00693338;IPI00703776	Biological Process	Positive regulation of MAPK cascade	11	2	211	0.5694
GO:1901068	IPI00686225;IPI00712775	Biological Process	Guanosine-containing compound metabolic process	11	2	211	0.5694
GO:0051726	IPI00690446;IPI00691963;IPI00693338;IPI00695508;IPI00715354	Biological Process	Regulation of cell cycle	6	5	211	0.5729
GO:0006897	IPI00690160;IPI00703776;IPI00718381;IPI00868597	Biological Process	Endocytosis	7	4	211	0.5748
GO:0007017	IPI00687601;IPI00689750;IPI00701223;IPI00707359	Biological Process	Microtubule-based process	5	4	211	0.5748
GO:0019318	IPI00689323;IPI00697184;IPI00698589;IPI00706942	Biological Process	Hexose metabolic process	8	4	211	0.5748
GO:0022900	IPI00692468;IPI00707559;IPI00711233;IPI00712677	Biological Process	Electron transport chain	6	4	211	0.5748

GO:0006644	IPI00692819;IPI00695965;IPI00944429	Biological Process	Phospholipid metabolic process	7	3	211	0.5787
GO:0010035	IPI00692468;IPI00695508;IPI00695965	Biological Process	Response to inorganic substance	5	3	211	0.5787
GO:0050778	IPI00693338;IPI00701698;IPI00713757	Biological Process	Positive regulation of immune response	6	3	211	0.5787
GO:0009056	IPI00686225;IPI00687539;IPI00692819;IPI00694312;IPI00694751;IPI00695176;IPI00695965;IPI00697757;IPI00698589;IPI00699355;IPI00700547;IPI00701790;IPI00702650;IPI00703753;IPI00703776;IPI00706942;IPI00707875;IPI00711419;IPI00712775;IPI00716555;IPI00842934	Biological Process	Catabolic process	4	21	211	0.5793
GO:0000041	IPI00687842	Biological Process	Transition metal ion transport	8	1	211	0.5841
GO:0002429	IPI00693338	Biological Process	Immune response-activating cell surface receptor signaling pathway	6	1	211	0.5841
GO:0002768	IPI00693338	Biological Process	Immune response-regulating cell surface receptor signaling pathway	7	1	211	0.5841
GO:0006637	IPI00696912	Biological Process	Acyl-CoA metabolic process	7	1	211	0.5841
GO:0006641	IPI00842934	Biological Process	Triglyceride metabolic process	8	1	211	0.5841
GO:0006665	IPI00718311	Biological Process	Sphingolipid metabolic process	6	1	211	0.5841
GO:0006749	IPI00692468	Biological Process	Glutathione metabolic process	8	1	211	0.5841
GO:0006936	IPI00688651	Biological Process	Muscle contraction	7	1	211	0.5841
GO:0010821	IPI00701698	Biological Process	Regulation of mitochondrion organization	6	1	211	0.5841
GO:0010827	IPI00703753	Biological Process	Regulation of glucose transport	9	1	211	0.5841
GO:0015988	IPI00716195	Biological Process	Energy coupled proton transmembrane transport, against electrochemical gradient	10	1	211	0.5841

GO:0015991	IPI00716195	Biological Process	ATP hydrolysis coupled proton transport	11	1	211	0.5841
GO:0017015	IPI00717119	Biological Process	Regulation of transforming growth factor beta receptor signaling pathway	11	1	211	0.5841
GO:0018205	IPI00704728	Biological Process	Peptidyl-lysine modification	9	1	211	0.5841
GO:0030193	IPI00701166	Biological Process	Regulation of blood coagulation	7	1	211	0.5841
GO:0031532	IPI00842934	Biological Process	Actin cytoskeleton reorganization	7	1	211	0.5841
GO:0032088	IPI00703776	Biological Process	Negative regulation of NF-kappaB transcription factor activity	14	1	211	0.5841
GO:0032102	IPI00697757	Biological Process	Negative regulation of response to external stimulus	6	1	211	0.5841
GO:0032434	IPI00732368	Biological Process	Regulation of proteasomal ubiquitin-dependent protein catabolic process	11	1	211	0.5841
GO:0035383	IPI00696912	Biological Process	Thioester metabolic process	6	1	211	0.5841
GO:0042439	IPI00695965	Biological Process	Ethanolamine-containing compound metabolic process	8	1	211	0.5841
GO:0042632	IPI00695965	Biological Process	Cholesterol homeostasis	9	1	211	0.5841
GO:0045444	IPI00690446	Biological Process	Fat cell differentiation	7	1	211	0.5841
GO:0046661	IPI00697184	Biological Process	Male sex differentiation	6	1	211	0.5841
GO:0050708	IPI00697184	Biological Process	Regulation of protein secretion	7	1	211	0.5841
GO:0050808	IPI00694504	Biological Process	Synapse organization	5	1	211	0.5841
GO:0051701	IPI00707359	Biological Process	Interaction with host	6	1	211	0.5841
GO:0055092	IPI00695965	Biological Process	Sterol homeostasis	8	1	211	0.5841
GO:0090002	IPI00708921	Biological Process	Establishment of protein localization to plasma membrane	7	1	211	0.5841
GO:1900046	IPI00701166	Biological Process	Regulation of hemostasis	7	1	211	0.5841
GO:0006007	IPI00698589;IPI00706942	Biological Process	Glucose catabolic process	9	2	211	0.5869
GO:0006518	IPI00692468;IPI00694312	Biological Process	Peptide metabolic process	7	2	211	0.5869
GO:0007409	IPI00687372;IPI00687601	Biological Process	Axonogenesis	8	2	211	0.5869
GO:0008544	IPI00699355;IPI00715354	Biological Process	Epidermis development	9	2	211	0.5869
GO:0051348	IPI00700789;IPI01028487	Biological Process	Negative regulation of transferase activity	6	2	211	0.5869

GO:1901657	IPI00686225;IPI00703753;IPI00712775;IPI00726650;IPI00883375;IPI01028487	Biological Process	Glycosyl compound metabolic process	5	6	211	0.5919
GO:0006417	IPI00694214;IPI00701698;IPI00704728	Biological Process	Regulation of translation	8	3	211	0.5926
GO:0034097	IPI00687539;IPI00690160;IPI00708921	Biological Process	Response to cytokine	6	3	211	0.5926
GO:1901292	IPI00686225;IPI00703753;IPI00712775	Biological Process	Nucleoside phosphate catabolic process	8	3	211	0.5926
GO:0032787	IPI00694312;IPI00696912;IPI00699355;IPI00702650;IPI00842934	Biological Process	Monocarboxylic acid metabolic process	8	5	211	0.5946
GO:0044092	IPI00695508;IPI00700789;IPI00703776;IPI00715354;IPI00871133;IPI01028487	Biological Process	Negative regulation of molecular function	5	6	211	0.6017
GO:0018130	IPI00686225;IPI00689323;IPI00692676;IPI00692911;IPI00694851;IPI00701698;IPI00703776;IPI00712671;IPI00726650;IPI00742596;IPI00883375;IPI01028487	Biological Process	Heterocycle biosynthetic process	6	12	211	0.6032
GO:0002521	IPI00690094;IPI00693338	Biological Process	Leukocyte differentiation	7	2	211	0.6039
GO:0009108	IPI00696912;IPI00742596	Biological Process	Coenzyme biosynthetic process	7	2	211	0.6039
GO:0043269	IPI00688651;IPI00695508	Biological Process	Regulation of ion transport	6	2	211	0.6039
GO:0050657	IPI00690232;IPI00704728	Biological Process	Nucleic acid transport	8	2	211	0.6039
GO:0050658	IPI00690232;IPI00704728	Biological Process	RNA transport	7	2	211	0.6039
GO:0051236	IPI00690232;IPI00704728	Biological Process	Establishment of RNA localization	6	2	211	0.6039

GO:0071345	IPI00687539;IPI00708921	Biological Process	Cellular response to cytokine stimulus	7	2	211	0.6039
GO:0006006	IPI00697184;IPI00698589;IPI00706942	Biological Process	Glucose metabolic process	9	3	211	0.6063
GO:0009260	IPI00686225;IPI00726650;IPI01028487	Biological Process	Ribonucleotide biosynthetic process	10	3	211	0.6063
GO:0016337	IPI00693338;IPI00694751;IPI00695965	Biological Process	Single organismal cell-cell adhesion	6	3	211	0.6063
GO:0002685	IPI00701698	Biological Process	Regulation of leukocyte migration	5	1	211	0.6094
GO:0002703	IPI00693338	Biological Process	Regulation of leukocyte mediated immunity	6	1	211	0.6094
GO:0003012	IPI00688651	Biological Process	Muscle system process	6	1	211	0.6094
GO:0005977	IPI00699798	Biological Process	Glycogen metabolic process	8	1	211	0.6094
GO:0006073	IPI00699798	Biological Process	Cellular glucan metabolic process	7	1	211	0.6094
GO:0006909	IPI00690160	Biological Process	Phagocytosis	5	1	211	0.6094
GO:0006914	IPI00694751	Biological Process	Autophagy	6	1	211	0.6094
GO:0006919	IPI00694751	Biological Process	Activation of cysteine-type endopeptidase activity involved in apoptotic process	14	1	211	0.6094
GO:0008016	IPI00716843	Biological Process	Regulation of heart contraction	9	1	211	0.6094
GO:0009069	IPI00742596	Biological Process	Serine family amino acid metabolic process	8	1	211	0.6094
GO:0010675	IPI00703753	Biological Process	Regulation of cellular carbohydrate metabolic process	6	1	211	0.6094
GO:0030216	IPI00715354	Biological Process	Keratinocyte differentiation	9	1	211	0.6094
GO:0032387	IPI00687601	Biological Process	Negative regulation of intracellular transport	7	1	211	0.6094
GO:0032409	IPI00695508	Biological Process	Regulation of transporter activity	7	1	211	0.6094
GO:0034765	IPI00695508	Biological Process	Regulation of ion transmembrane transport	7	1	211	0.6094
GO:0043604	IPI00694312	Biological Process	Amide biosynthetic process	7	1	211	0.6094
GO:0044042	IPI00699798	Biological Process	Glucan metabolic process	7	1	211	0.6094
GO:0045619	IPI00693338	Biological Process	Regulation of lymphocyte differentiation	9	1	211	0.6094
GO:0046834	IPI00692819	Biological Process	Lipid phosphorylation	8	1	211	0.6094
GO:0046854	IPI00692819	Biological Process	Phosphatidylinositol phosphorylation	10	1	211	0.6094
GO:0048562	IPI00697184	Biological Process	Embryonic organ morphogenesis	8	1	211	0.6094
GO:0051817	IPI00707359	Biological Process	Modification of morphology or physiology of other organism involved in symbiotic interaction	5	1	211	0.6094
GO:0060191	IPI00695965	Biological Process	Regulation of lipase activity	7	1	211	0.6094

GO:0070507	IPI00904732	Biological Process	Regulation of microtubule cytoskeleton organization	7	1	211	0.6094
GO:0097202	IPI00694751	Biological Process	Activation of cysteine-type endopeptidase activity	13	1	211	0.6094
GO:0005996	IPI00689323;IPI00697184;IPI00698589;IPI00706942	Biological Process	Monosaccharide metabolic process	7	4	211	0.6106
GO:0006631	IPI00694312;IPI00699355;IPI00702650;IPI00842934	Biological Process	Fatty acid metabolic process	9	4	211	0.6106
GO:0006629	IPI00686601;IPI00692627;IPI00692819;IPI00694312;IPI00695965;IPI00697184;IPI00699355;IPI00702650;IPI00718311;IPI00842934;IPI00944429	Biological Process	Lipid metabolic process	5	11	211	0.6107
GO:0040011	IPI00687372;IPI00690160;IPI00693338;IPI00693628;IPI00694751;IPI00699355	Biological Process	Locomotion	3	6	211	0.6113
GO:0009150	IPI00686225;IPI00703753;IPI00712775;IPI00726650;IPI01028487	Biological Process	Purine ribonucleotide metabolic process	11	5	211	0.6156

GO:0051171	IPI00685278;IPI00686225;IPI00689750;IPI00690446;IPI00691963;IPI00692468;IPI00692676;IPI00692911;IPI00693338;IPI00694851;IPI00695331;IPI00696930;IPI00699355; IPI00700542;IPI00701698;IPI00703268;IPI00703731;IPI00703776;IPI00705463;IPI00716158;IPI00717759;IPI00842934;IPI01018577	Biological Process	Regulation of nitrogen compound metabolic process	5	23	211	0.6185
GO:0046390	IPI00686225;IPI00726650;IPI01028487	Biological Process	Ribose phosphate biosynthetic process	7	3	211	0.6196
GO:0001933	IPI00693338;IPI00703753	Biological Process	Negative regulation of protein phosphorylation	10	2	211	0.6204
GO:0019320	IPI00698589;IPI00706942	Biological Process	Hexose catabolic process	8	2	211	0.6204
GO:0048667	IPI00687372;IPI00687601	Biological Process	Cell morphogenesis involved in neuron differentiation	11	2	211	0.6204
GO:0006397	IPI00685278;IPI00690232;IPI00695600;IPI00697891;IPI00701698;IPI00717759	Biological Process	mRNA processing	10	6	211	0.6208
GO:1901137	IPI00686225;IPI00689323;IPI00712671;IPI00726650;IPI00883375;IPI01028487	Biological Process	Carbohydrate derivative biosynthetic process	6	6	211	0.6208
GO:0048729	IPI00688489;IPI00694504;IPI00707359;IPI01018577	Biological Process	Tissue morphogenesis	5	4	211	0.6221
GO:0048870	IPI00690160;IPI00693338;IPI00693628;IPI00694751;IPI00699355	Biological Process	Ccell motility	6	5	211	0.6259

GO:0051674	IPI00690160;IPI00693338;IPI00693628;IPI00694751;IPI00699355	Biological Process	Localization of cell	4	5	211	0.6259
GO:0051223	IPI00695506;IPI00697184;IPI00715354	Biological Process	Regulation of protein transport	7	3	211	0.6327
GO:0001959	IPI00693338	Biological Process	Regulation of cytokine-mediated signaling pathway	9	1	211	0.6332
GO:0006109	IPI00703753	Biological Process	Regulation of carbohydrate metabolic process	6	1	211	0.6332
GO:0006112	IPI00699798	Biological Process	Energy reserve metabolic process	7	1	211	0.6332
GO:0006898	IPI00703776	Biological Process	Receptor-mediated endocytosis	8	1	211	0.6332
GO:0007059	IPI00701223	Biological Process	Chromosome segregation	5	1	211	0.6332
GO:0007411	IPI00687372	Biological Process	Axon guidance	7	1	211	0.6332
GO:0010565	IPI00695965	Biological Process	Regulation of cellular ketone metabolic process	6	1	211	0.6332
GO:0010970	IPI00687601	Biological Process	Microtubule-based transport	7	1	211	0.6332
GO:0030534	IPI00686966	Biological Process	Adult behavior	5	1	211	0.6332
GO:0030705	IPI00687601	Biological Process	Cytoskeleton-dependent intracellular transport	6	1	211	0.6332
GO:0034637	IPI00692819	Biological Process	Cellular carbohydrate biosynthetic process	6	1	211	0.6332
GO:0042157	IPI00695965	Biological Process	Lipoprotein metabolic process	6	1	211	0.6332
GO:0045638	IPI00698039	Biological Process	Negative regulation of myeloid cell differentiation	9	1	211	0.6332
GO:0048545	IPI00694204	Biological Process	Response to steroid hormone	6	1	211	0.6332
GO:0048747	IPI00694504	Biological Process	Muscle fiber development	10	1	211	0.6332
GO:0048754	IPI00707359	Biological Process	Branching morphogenesis of an epithelial tube	7	1	211	0.6332
GO:0050818	IPI00701166	Biological Process	Regulation of coagulation	6	1	211	0.6332
GO:0061448	IPI00699355	Biological Process	Connective tissue development	6	1	211	0.6332
GO:0090257	IPI00688651	Biological Process	Regulation of muscle system process	7	1	211	0.6332
GO:0097485	IPI00687372	Biological Process	Neuron projection guidance	6	1	211	0.6332
GO:1902107	IPI00693338	Biological Process	Positive regulation of leukocyte differentiation	9	1	211	0.6332
GO:0045934	IPI00691963;IPI00692676;IPI00699355;IPI00701698;IPI00703731;IPI00842934;IPI01018577	Biological Process	Negative regulation of nucleobase-containing compound metabolic process	8	7	211	0.6346

GO:0051172	IPI00691963;IPI00692676;IPI00699355;IPI00701698;IPI00703731;IPI00842934;IPI01018577	Biological Process	Negative regulation of nitrogen compound metabolic process	6	7	211	0.6346
GO:0006403	IPI00690232;IPI00704728	Biological Process	RNA localization	5	2	211	0.6364
GO:0015931	IPI00690232;IPI00704728	Biological Process	Nucleobase-containing compound transport	7	2	211	0.6364
GO:0046365	IPI00698589;IPI00706942	Biological Process	Monosaccharide catabolic process	7	2	211	0.6364
GO:0048812	IPI00687372;IPI00687601	Biological Process	Neuron projection morphogenesis	7	2	211	0.6364
GO:0019752	IPI00692468;IPI00694312;IPI00694739;IPI00696912;IPI00699355;IPI00702650;IPI00704728;IPI00711419;IPI00712671;IPI00742596;IPI00842934;IPI00883375	Biological Process	Carboxylic acid metabolic process	7	12	211	0.6373
GO:0051704	IPI00686841;IPI00687625;IPI00691669;IPI00693338;IPI00707359;IPI00718757	Biological Process	Multi-organism process	3	6	211	0.6393
GO:0051186	IPI00694312;IPI00696912;IPI00706942;IPI00742596	Biological Process	Cofactor metabolic process	5	4	211	0.6445
GO:0002009	IPI00694504;IPI00707359;IPI01018577	Biological Process	Morphogenesis of an epithelium	6	3	211	0.6454
GO:0033993	IPI00687625;IPI00694204;IPI00697184	Biological Process	Response to lipid	6	3	211	0.6454
GO:0046486	IPI00692819;IPI00695965;IPI00842934	Biological Process	Glycerolipid metabolic process	6	3	211	0.6454
GO:0048598	IPI00697184;IPI00712671;IPI01018577	Biological Process	Embryonic morphogenesis	7	3	211	0.6454

GO:0010608	IPI00687625;IPI00691963;IPI00694214;IPI00701698;IPI00704728	Biological Process	Posttranscriptional regulation of gene expression	8	5	211	0.6459
GO:0044270	IPI00686225;IPI00687539;IPI00703753;IPI00712775;IPI00716555	Biological Process	Cellular nitrogen compound catabolic process	6	5	211	0.6459
GO:0046700	IPI00686225;IPI00687539;IPI00703753;IPI00712775;IPI00716555	Biological Process	Heterocycle catabolic process	6	5	211	0.6459
GO:0022402	IPI00688651;IPI00691963;IPI00694214;IPI00701223;IPI00705941;IPI00904732	Biological Process	Cell cycle process	6	6	211	0.6484
GO:0030163	IPI00695176;IPI00697757;IPI00700547;IPI00701790;IPI00703776;IPI00707875	Biological Process	Protein catabolic process	7	6	211	0.6484
GO:0042326	IPI00693338;IPI00703753	Biological Process	Negative regulation of phosphorylation	9	2	211	0.6518
GO:0001822	IPI00703547	Biological Process	Kidney development	9	1	211	0.6556
GO:0002699	IPI00693338	Biological Process	Positive regulation of immune effector process	6	1	211	0.6556
GO:0006638	IPI00842934	Biological Process	Neutral lipid metabolic process	6	1	211	0.6556
GO:0006639	IPI00842934	Biological Process	Acylglycerol metabolic process	7	1	211	0.6556
GO:0007369	IPI00712671	Biological Process	Gastrulation	8	1	211	0.6556
GO:0008406	IPI00697184	Biological Process	Gonad development	7	1	211	0.6556
GO:0009791	IPI00692468	Biological Process	Post-embryonic development	6	1	211	0.6556
GO:0016032	IPI00707359	Biological Process	Viral process	5	1	211	0.6556
GO:0019221	IPI00708921	Biological Process	Cytokine-mediated signaling pathway	8	1	211	0.6556
GO:0019362	IPI00706942	Biological Process	Pyridine nucleotide metabolic process	10	1	211	0.6556
GO:0030308	IPI00703753	Biological Process	Negative regulation of cell growth	7	1	211	0.6556
GO:0032886	IPI00904732	Biological Process	Regulation of microtubule-based process	6	1	211	0.6556
GO:0035821	IPI00707359	Biological Process	Modification of morphology or physiology of other organism	4	1	211	0.6556
GO:0042742	IPI00691669	Biological Process	Defense response to bacterium	7	1	211	0.6556
GO:0043200	IPI00713573	Biological Process	Response to amino acid	6	1	211	0.6556

GO:0043406	IPI00693338	Biological Process	Positive regulation of MAP kinase activity	13	1	211	0.6556
GO:0043524	IPI00692468	Biological Process	Negative regulation of neuron apoptotic process	11	1	211	0.6556
GO:0044764	IPI00707359	Biological Process	Multi-organism cellular process	4	1	211	0.6556
GO:0046496	IPI00706942	Biological Process	Nicotinamide nucleotide metabolic process	11	1	211	0.6556
GO:0060759	IPI00693338	Biological Process	Regulation of response to cytokine stimulus	7	1	211	0.6556
GO:0061041	IPI00701166	Biological Process	Regulation of wound healing	7	1	211	0.6556
GO:0090092	IPI00717119	Biological Process	Regulation of transmembrane receptor protein serine/threonine kinase signaling pathway	10	1	211	0.6556
GO:0090288	IPI00717119	Biological Process	Negative regulation of cellular response to growth factor stimulus	9	1	211	0.6556
GO:1901215	IPI00692468	Biological Process	Negative regulation of neuron death	8	1	211	0.6556
GO:2001020	IPI01018577	Biological Process	Regulation of response to DNA damage stimulus	7	1	211	0.6556
GO:0006163	IPI00686225;IPI00703753;IPI00712775;IPI00726650;IPI01028487	Biological Process	Purine nucleotide metabolic process	10	5	211	0.6557
GO:0006468	IPI00688651;IPI00696930;IPI01018577	Biological Process	Protein phosphorylation	8	3	211	0.6578
GO:0031175	IPI00687372;IPI00687601;IPI00694504	Biological Process	Neuron projection development	6	3	211	0.6578
GO:0019219	IPI00685278;IPI00689750;IPI00690446;IPI00691963;IPI00692468;IPI00692676;IPI00692911;IPI00693338;IPI00694851;IPI00695331;IPI00696930;IPI00699355;IPI00700542;IPI00701698;IPI00703268;IPI00703731;IPI00703776;IPI00705463;IPI00716158;IPI00717759;IPI00842934;IPI01018577	Biological Process	Regulation of nucleobase-containing compound metabolic process	7	22	211	0.6637

GO:0051603	IPI00695176;IPI00697757;IPI00700547;IPI00703776;IPI00707875	Biological Process	Proteolysis involved in cellular protein catabolic process	8	5	211	0.6653
GO:0001525	IPI00689035;IPI00840588	Biological Process	Angiogenesis	10	2	211	0.6667
GO:0051346	IPI00715354;IPI00871133	Biological Process	Negative regulation of hydrolase activity	6	2	211	0.6667
GO:0034613	IPI00686601;IPI00689750;IPI00691963;IPI00695666;IPI00701223;IPI00705941;IPI00707359;IPI00708921;IPI00710408;IPI00714481;IPI00715339;IPI00904732	Biological Process	Cellular protein localization	6	12	211	0.6699
GO:0009126	IPI00686225;IPI00726650;IPI01028487	Biological Process	Purine nucleoside monophosphate metabolic process	10	3	211	0.6700
GO:0009167	IPI00686225;IPI00726650;IPI01028487	Biological Process	Purine ribonucleoside monophosphate metabolic process	11	3	211	0.6700
GO:0009259	IPI00686225;IPI00703753;IPI00712775;IPI00726650;IPI01028487	Biological Process	Ribonucleotide metabolic process	10	5	211	0.6747
GO:0070727	IPI00686601;IPI00689750;IPI00691963;IPI00695666;IPI00701223;IPI00705941;IPI00707359;IPI00708921;IPI00710408;IPI00714481;IPI00715339;IPI00904732	Biological Process	Cellular macromolecule localization	5	12	211	0.6762
GO:0043086	IPI00700789;IPI00715354;IPI00871133;IPI01028487	Biological Process	Negative regulation of catalytic activity	5	4	211	0.6764
GO:0009566	IPI00689035	Biological Process	Fertilization	5	1	211	0.6766
GO:0031349	IPI00694751	Biological Process	Positive regulation of defense response	7	1	211	0.6766
GO:0032147	IPI00693338	Biological Process	Activation of protein kinase activity	12	1	211	0.6766

GO:0034612	IPI00708921	Biological Process	Response to tumor necrosis factor	7	1	211	0.6766
GO:0034762	IPI00695508	Biological Process	Regulation of transmembrane transport	6	1	211	0.6766
GO:0043433	IPI00703776	Biological Process	Negative regulation of sequence-specific DNA binding transcription factor activity	13	1	211	0.6766
GO:0045785	IPI00701698	Biological Process	Positive regulation of cell adhesion	5	1	211	0.6766
GO:0046824	IPI00715354	Biological Process	Positive regulation of nucleocytoplasmic transport	9	1	211	0.6766
GO:0046890	IPI00695965	Biological Process	Regulation of lipid biosynthetic process	7	1	211	0.6766
GO:0046916	IPI00687842	Biological Process	Cellular transition metal ion homeostasis	10	1	211	0.6766
GO:0051924	IPI00695508	Biological Process	Regulation of calcium ion transport	10	1	211	0.6766
GO:2001235	IPI00686966	Biological Process	Positive regulation of apoptotic signaling pathway	9	1	211	0.6766
GO:0033157	IPI00695506;IPI00715354	Biological Process	Regulation of intracellular protein transport	7	2	211	0.6810
GO:0019693	IPI00686225;IPI00703753;IPI00712775;IPI00726650;IPI01028487	Biological Process	Ribose phosphate metabolic process	7	5	211	0.6839
GO:0051345	IPI00686966;IPI00694751;IPI00695890;IPI00695965	Biological Process	Positive regulation of hydrolase activity	6	4	211	0.6866
GO:0051336	IPI00686966;IPI00694751;IPI00695890;IPI00695965;IPI00703268;IPI00715354;IPI00871133	Biological Process	Regulation of hydrolase activity	6	7	211	0.6917

GO:0044238	IPI00685278;IPI00686225;IPI00686601;IPI00686803;IPI00686966;IPI00687539;IPI00688608;IPI00688651;IPI00689323;IPI00689325;IPI00690232;IPI00690308;IPI00690785;IPI00691963;IPI00692468;IPI00692627;IPI00692676;IPI00692819;IPI00692911;IPI00693338;IPI00694214;IPI00694312;IPI00694739;IPI00694851;IPI00695176;IPI00695600;IPI00695890;IPI00695965;IPI00696930;IPI00697081;IPI00697184;IPI00697757;IPI00697891;IPI00698039;IPI00698589;IPI00698900;IPI00699107;IPI00699355;IPI00699798;IPI00700295;IPI00700542;IP	Biological Process	Primary metabolic process	4	81	211	0.6918
GO:0034622	IPI00691963;IPI00698039;IPI00701698;IPI00705000;IPI00728768	Biological Process	Cellular macromolecular complex assembly	7	5	211	0.6930
GO:0044257	IPI00695176;IPI00697757;IPI00700547;IPI00703776;IPI00707875	Biological Process	Cellular protein catabolic process	7	5	211	0.6930
GO:1901565	IPI00686225;IPI00687539;IPI00711419;IPI00712775;IPI00716555	Biological Process	Organonitrogen compound catabolic process	6	5	211	0.6930
GO:0043588	IPI00699355;IPI00715354	Biological Process	Skin development	8	2	211	0.6949
GO:0044262	IPI00692819;IPI00699798	Biological Process	Cellular carbohydrate metabolic process	5	2	211	0.6949
GO:0001763	IPI00707359	Biological Process	Morphogenesis of a branching structure	5	1	211	0.6963
GO:0005976	IPI00699798	Biological Process	Polysaccharide metabolic process	6	1	211	0.6963
GO:0009411	IPI00703731	Biological Process	Response to UV	7	1	211	0.6963

GO:0016064	IPI00701698	Biological Process	Immunoglobulin mediated immune response	8	1	211	0.6963
GO:0040014	IPI00686966	Biological Process	Regulation of multicellular organism growth	6	1	211	0.6963
GO:0044264	IPI00699798	Biological Process	Cellular polysaccharide metabolic process	6	1	211	0.6963
GO:0045137	IPI00697184	Biological Process	Development of primary sexual characteristics	6	1	211	0.6963
GO:0050730	IPI00693338	Biological Process	Regulation of peptidyl-tyrosine phosphorylation	9	1	211	0.6963
GO:0055088	IPI00695965	Biological Process	Lipid homeostasis	7	1	211	0.6963
GO:0061136	IPI00732368	Biological Process	Regulation of proteasomal protein catabolic process	10	1	211	0.6963
GO:0061138	IPI00707359	Biological Process	Morphogenesis of a branching epithelium	6	1	211	0.6963
GO:0072524	IPI00706942	Biological Process	Pyridine-containing compound metabolic process	6	1	211	0.6963
GO:0072527	IPI00687539	Biological Process	Pyrimidine-containing compound metabolic process	6	1	211	0.6963
GO:0006520	IPI00692468;IPI00694739;IPI00704728;IPI00711419;IPI00742596;IPI00883375	Biological Process	Cellular amino acid metabolic process	6	6	211	0.6999
GO:1901361	IPI00686225;IPI00687539;IPI00703753;IPI00712775;IPI00716555	Biological Process	Organic cyclic compound catabolic process	6	5	211	0.7019
GO:0006732	IPI00696912;IPI00706942;IPI00742596	Biological Process	Coenzyme metabolic process	6	3	211	0.7044
GO:0032990	IPI00687372;IPI00687601;IPI00695890	Biological Process	Cell part morphogenesis	7	3	211	0.7044
GO:0006650	IPI00692819;IPI00695965	Biological Process	Glycerophospholipid metabolic process	8	2	211	0.7083
GO:0009143	IPI00703753;IPI00712775	Biological Process	Nucleoside triphosphate catabolic process	9	2	211	0.7083
GO:0044724	IPI00698589;IPI00706942	Biological Process	Single-organism carbohydrate catabolic process	6	2	211	0.7083
GO:0050878	IPI00701166;IPI00730144	Biological Process	Regulation of body fluid levels	5	2	211	0.7083
GO:0000075	IPI00701223	Biological Process	Cell cycle checkpoint	7	1	211	0.7148
GO:0006099	IPI00708438	Biological Process	Tricarboxylic acid cycle	10	1	211	0.7148
GO:0006643	IPI00718311	Biological Process	Membrane lipid metabolic process	6	1	211	0.7148
GO:0006997	IPI00689750	Biological Process	Nucleus organization	5	1	211	0.7148
GO:0007160	IPI00714673	Biological Process	Cell-matrix adhesion	6	1	211	0.7148

GO:0009125	IPI00686225	Biological Process	Nucleoside monophosphate catabolic process	9	1	211	0.7148
GO:0009128	IPI00686225	Biological Process	Purine nucleoside monophosphate catabolic process	10	1	211	0.7148
GO:0009158	IPI00686225	Biological Process	Ribonucleoside monophosphate catabolic process	10	1	211	0.7148
GO:0009169	IPI00686225	Biological Process	Purine ribonucleoside monophosphate catabolic process	11	1	211	0.7148
GO:0009913	IPI00715354	Biological Process	Epidermal cell differentiation	8	1	211	0.7148
GO:0019724	IPI00701698	Biological Process	B cell mediated immunity	7	1	211	0.7148
GO:0050821	IPI00691963	Biological Process	Protein stabilization	10	1	211	0.7148
GO:0051495	IPI00706141	Biological Process	Positive regulation of cytoskeleton organization	7	1	211	0.7148
GO:0051592	IPI00695508	Biological Process	Response to calcium ion	7	1	211	0.7148
GO:0001775	IPI00690094;IPI00693338;IPI00695506	Biological Process	Cell activation	5	3	211	0.7153
GO:0035295	IPI00697184;IPI00703547;IPI00707359	Biological Process	Tube development	6	3	211	0.7153
GO:0070201	IPI00695506;IPI00697184;IPI00715354	Biological Process	Regulation of establishment of protein localization	7	3	211	0.7153
GO:0071495	IPI00703753;IPI00713573;IPI00842934	Biological Process	Cellular response to endogenous stimulus	5	3	211	0.7153
GO:0032268	IPI00690446;IPI00693338;IPI00694214;IPI00701698;IPI00703753;IPI00703776;IPI00704728;IPI00715354;IPI00732368;IPI00842934	Biological Process	Regulation of cellular protein metabolic process	7	10	211	0.7154
GO:0031401	IPI00690446;IPI00693338;IPI00703776;IPI00842934	Biological Process	Positive regulation of protein modification process	9	4	211	0.7159
GO:0043603	IPI00692468;IPI00694312	Biological Process	Cellular amide metabolic process	6	2	211	0.7212
GO:0009123	IPI00686225;IPI00726650;IPI01028487	Biological Process	Nucleoside monophosphate metabolic process	9	3	211	0.7258
GO:0009161	IPI00686225;IPI00726650;IPI01028487	Biological Process	Ribonucleoside monophosphate metabolic process	10	3	211	0.7258

GO:0009628	IPI00689750;IPI00692468;IPI00697184;IPI00703731;IPI00703776	Biological Process	Response to abiotic stimulus	4	5	211	0.7275
GO:0033554	IPI00689750;IPI00691963;IPI00692468;IPI00694504;IPI00695965;IPI00700547;IPI00715354;IPI01018577	Biological Process	Cellular response to stress	5	8	211	0.7277
GO:0044237	IPI00685278;IPI00686225;IPI00686601;IPI00686803;IPI00687539;IPI00688608;IPI00688651;IPI00689323;IPI00689325;IPI00690232;IPI00690308;IPI00690785;IPI00691963;IPI00692468;IPI00692627;IPI00692676;IPI00692819;IPI00692911;IPI00693338;IPI00694214;IPI00694312;IPI00694739;IPI00694751;IPI00694851;IPI00695176;IPI00695600;IPI00695890;IPI00695965;IPI00696912;IPI00696930;IPI00697081;IPI00697184;IPI00697757;IPI00697891;IPI00698039;IPI00698589;IPI00698900;IPI00699107;IPI00699355;IPI00699798;IPI00700295;IP	Biological Process	Cellular metabolic process	4	80	211	0.7296
GO:0002757	IPI00693338	Biological Process	Immune response-activating signal transduction	5	1	211	0.7322
GO:0002764	IPI00693338	Biological Process	Immune response-regulating signaling pathway	6	1	211	0.7322
GO:0006184	IPI00712775	Biological Process	GTP catabolic process	12	1	211	0.7322
GO:0006754	IPI01028487	Biological Process	ATP biosynthetic process	11	1	211	0.7322
GO:0007519	IPI00694504	Biological Process	Skeletal muscle tissue development	10	1	211	0.7322
GO:0010810	IPI00701698	Biological Process	Regulation of cell-substrate adhesion	6	1	211	0.7322

GO:0031334	IPI00706141	Biological Process	Positive regulation of protein complex assembly	7	1	211	0.7322
GO:0043393	IPI00703776	Biological Process	Regulation of protein binding	6	1	211	0.7322
GO:0060538	IPI00694504	Biological Process	Skeletal muscle organ development	9	1	211	0.7322
GO:0009154	IPI00686225;IPI00712775	Biological Process	Purine ribonucleotide catabolic process	11	2	211	0.7336
GO:0009261	IPI00686225;IPI00712775	Biological Process	Ribonucleotide catabolic process	10	2	211	0.7336
GO:0010563	IPI00693338;IPI00703753	Biological Process	Negative regulation of phosphorus metabolic process	6	2	211	0.7336
GO:0045936	IPI00693338;IPI00703753	Biological Process	Negative regulation of phosphate metabolic process	8	2	211	0.7336
GO:0072657	IPI00686601;IPI00708921	Biological Process	Protein localization to membrane	5	2	211	0.7336
GO:0030097	IPI00690094;IPI00692468;IPI00693338	Biological Process	Hemopoiesis	6	3	211	0.7360
GO:0033365	IPI00689750;IPI00691963;IPI00707359;IPI00904732	Biological Process	Protein localization to organelle	7	4	211	0.7430
GO:0051338	IPI00693338;IPI00700789;IPI00715354;IPI01028487	Biological Process	Regulation of transferase activity	6	4	211	0.7430

GO:0071704	IPI00685278;IPI00686225;IPI00686601;IPI00686803;IPI00686966;IPI00687539;IPI00688608;IPI00688651;IPI00689323;IPI00689325;IPI00690232;IPI00690308;IPI00690785;IPI00691963;IPI00692468;IPI00692627;IPI00692676;IPI00692819;IPI00692911;IPI00693338;IPI00694214;IPI00694312;IPI00694739;IPI00694851;IPI00695176;IPI00695600;IPI00695890;IPI00695965;IPI00696912;IPI00696930;IPI00697081;IPI00697184;IPI00697757;IPI00697891;IPI00698039;IPI00698589;IPI00698900;IPI00699107;IPI00699355;IPI00699798;IPI00700295;IP	Biological Process	Organic substance metabolic process	4	84	211	0.7455
GO:0001817	IPI00701698;IPI00703776	Biological Process	Regulation of cytokine production	6	2	211	0.7455
GO:0006195	IPI00686225;IPI00712775	Biological Process	Purine nucleotide catabolic process	10	2	211	0.7455
GO:0016052	IPI00698589;IPI00706942	Biological Process	Carbohydrate catabolic process	6	2	211	0.7455
GO:0071900	IPI00693338;IPI00715354	Biological Process	Regulation of protein serine/threonine kinase activity	11	2	211	0.7455
GO:0009967	IPI00686966;IPI00690094;IPI00693338;IPI00699355;IPI00701698;IPI00703776	Biological Process	Positive regulation of signal transduction	8	6	211	0.7462
GO:0048584	IPI00686966;IPI00690094;IPI00693338;IPI00694751;IPI00699355;IPI00701698;IPI00703776;IPI00713757;IPI01018577	Biological Process	Positive regulation of response to stimulus	5	9	211	0.7462

GO:0001666	IPI00689750	Biological Process	Response to hypoxia	5	1	211	0.7485
GO:0006733	IPI00706942	Biological Process	Oxidoreduction coenzyme metabolic process	7	1	211	0.7485
GO:0007548	IPI00697184	Biological Process	Sex differentiation	5	1	211	0.7485
GO:0009060	IPI00708438	Biological Process	Aerobic respiration	8	1	211	0.7485
GO:0036293	IPI00689750	Biological Process	Response to decreased oxygen levels	6	1	211	0.7485
GO:0043900	IPI00701698	Biological Process	Regulation of multi-organism process	4	1	211	0.7485
GO:0045786	IPI00691963	Biological Process	Negative regulation of cell cycle	6	1	211	0.7485
GO:0006351	IPI00692676;IPI00692911;IPI00694851;IPI00701698;IPI00703776	Biological Process	Transcription, DNA-templated	10	5	211	0.7515
GO:1901136	IPI00686225;IPI00700547;IPI00712775	Biological Process	Carbohydrate derivative catabolic process	6	3	211	0.7556
GO:0009987	IPI00685278;IPI00685792;IPI00686225;IPI00686601;IPI00686803;IPI00686966;IPI00687372;IPI00687539;IPI00687601;IPI00687657;IPI00687842;IPI00688489;IPI00688608;IPI00688651;IPI00688921;IPI00689035;IPI00689228;IPI00689323;IPI00689325;IPI00689750;IPI00689760;IPI00690094;IPI00690160;IPI00690232;IPI00690308;IPI00690446;IPI00690785;IPI00691963;IPI00692468;IPI00692627;IPI00692676;IPI00692819;IPI00692911;IPI00693338;IPI00693628;IPI00694204;IPI00694214;IPI00694312;IPI00694504;IPI00694580;IPI00694739;IP	Biological Process	Cellular process	3	134	211	0.7560
GO:0006152	IPI00686225;IPI00712775	Biological Process	Purine nucleoside catabolic process	9	2	211	0.7570
GO:0042454	IPI00686225;IPI00712775	Biological Process	Ribonucleoside catabolic process	9	2	211	0.7570

GO:0046130	IPI00686225;IPI00712775	Biological Process	Purine ribonucleoside catabolic process	10	2	211	0.7570
GO:0008152	IPI00685278;IPI00686225;IPI00686601;IPI00686803;IPI00686841;IPI00686966;IPI00687539;IPI00688608;IPI00688651;IPI00689323;IPI00689325;IPI00690232;IPI00690308;IPI00690785;IPI00691963;IPI00692468;IPI00692627;IPI00692676;IPI00692819;IPI00692911;IPI00693338;IPI00694214;IPI00694312;IPI00694739;IPI00694751;IPI00694851;IPI00695176;IPI00695600;IPI00695890;IPI00695965;IPI00696912;IPI00696930;IPI00697081;IPI00697184;IPI00697757;IPI00697891;IPI00698039;IPI00698589;IPI00698900;IPI00699107;IPI00699355;IP	Biological Process	Metabolic process	3	91	211	0.7590
GO:0000902	IPI00687372;IPI00687601;IPI00714673;IPI01018577	Biological Process	Cell morphogenesis	7	4	211	0.7600
GO:0002449	IPI00701698	Biological Process	Lymphocyte mediated immunity	6	1	211	0.7639
GO:0002460	IPI00701698	Biological Process	Adaptive immune response based on somatic recombination of immune receptors built from immunoglobulin superfamily domains	6	1	211	0.7639
GO:0008360	IPI00690160	Biological Process	Regulation of cell shape	9	1	211	0.7639
GO:0031396	IPI00703776	Biological Process	Regulation of protein ubiquitination	11	1	211	0.7639
GO:0032496	IPI00687625	Biological Process	Response to lipopolysaccharide	7	1	211	0.7639
GO:0090287	IPI00717119	Biological Process	Regulation of cellular response to growth factor stimulus	8	1	211	0.7639
GO:0090316	IPI00715354	Biological Process	Positive regulation of intracellular protein transport	8	1	211	0.7639

GO:0000003	IPI00689035;IPI00694504;IPI00697184;IPI00701223;IPI00718311	Biological Process	Reproduction	3	5	211	0.7666
GO:0032774	IPI00692676;IPI00692911;IPI00694851;IPI00701698;IPI00703776	Biological Process	RNA biosynthetic process	8	5	211	0.7666
GO:0043066	IPI00692468;IPI00692676;IPI00694739;IPI00705660;IPI00708921	Biological Process	Negative regulation of apoptotic process	9	5	211	0.7666
GO:0009617	IPI00687625;IPI00691669	Biological Process	Response to bacterium	7	2	211	0.7680
GO:0060627	IPI00691963;IPI00707101	Biological Process	Regulation of vesicle-mediated transport	5	2	211	0.7680
GO:0044093	IPI00685792;IPI00686966;IPI00693338;IPI00694751;IPI00695508;IPI00695890;IPI00695965;IPI00703776	Biological Process	Positive regulation of molecular function	5	8	211	0.7722
GO:0048534	IPI00690094;IPI00692468;IPI00693338	Biological Process	Hematopoietic or lymphoid organ development	5	3	211	0.7740
GO:0010647	IPI00686966;IPI00690094;IPI00693338;IPI00699355;IPI00701698;IPI00703776	Biological Process	Positive regulation of cell communication	7	6	211	0.7740
GO:0023056	IPI00686966;IPI00690094;IPI00693338;IPI00699355;IPI00701698;IPI00703776	Biological Process	Positive regulation of signaling	5	6	211	0.7740
GO:0006818	IPI00716195	Biological Process	Hydrogen transport	5	1	211	0.7783
GO:0007186	IPI00716843	Biological Process	G-protein coupled receptor signaling pathway	8	1	211	0.7783
GO:0009145	IPI01028487	Biological Process	Purine nucleoside triphosphate biosynthetic process	10	1	211	0.7783
GO:0009206	IPI01028487	Biological Process	Purine ribonucleoside triphosphate biosynthetic process	11	1	211	0.7783
GO:0015992	IPI00716195	Biological Process	Proton transport	8	1	211	0.7783
GO:0022618	IPI00701698	Biological Process	Ribonucleoprotein complex assembly	8	1	211	0.7783

GO:0031331	IPI00695965	Biological Process	Positive regulation of cellular catabolic process	7	1	211	0.7783
GO:0043409	IPI00707359	Biological Process	Negative regulation of MAPK cascade	11	1	211	0.7783
GO:0043523	IPI00692468	Biological Process	Regulation of neuron apoptotic process	10	1	211	0.7783
GO:0045926	IPI00703753	Biological Process	Negative regulation of growth	5	1	211	0.7783
GO:0071902	IPI00693338	Biological Process	Positive regulation of protein serine/threonine kinase activity	12	1	211	0.7783
GO:0006281	IPI00700547;IPI01018577	Biological Process	DNA repair	9	2	211	0.7786
GO:0009164	IPI00686225;IPI00712775	Biological Process	Nucleoside catabolic process	8	2	211	0.7786
GO:0032940	IPI00702891;IPI00706141	Biological Process	Secretion by cell	5	2	211	0.7786
GO:0043170	IPI00685278;IPI00686803;IPI00686966;IPI00688608;IPI00688651;IPI00689325;IPI00690232;IPI00690308;IPI00690785;IPI00691963;IPI00692676;IPI00692911;IPI00693338;IPI00694214;IPI00694851;IPI00695176;IPI00695600;IPI00695890;IPI00695965;IPI00696930;IPI00697081;IPI00697757;IPI00697891;IPI00698039;IPI00698900;IPI00699107;IPI00699798;IPI00700295;IPI00700542;IPI00700547;IPI00700789;IPI00701698;IPI00701790;IPI00702700;IPI00703731;IPI00703776;IPI00703854;IPI00704728;IPI00705000;IPI00706624;IPI00707359;IP	Biological Process	Macromolecule metabolic process	5	55	211	0.7802

GO:0031323	IPI00685278;IPI00686225;IPI00689750;IPI00690446;IPI00691963;IPI00692468;IPI00692676;IPI00692911;IPI00693338;IPI00694214;IPI00694851;IPI00695331;IPI00695965;IPI00696930;IPI00699355;IPI00700542;IPI00701698;IPI00703268;IPI00703731;IPI00703753;IPI00703776;IPI00704728;IPI00705463;IPI00715339;IPI00715354;IPI00716158;IPI00717759;IPI00732368;IPI00842934;IPI00912603;IPI01018577	Biological Process	Regulation of cellular metabolic process	5	31	211	0.7866
GO:0065009	IPI00685792;IPI00686966;IPI00692468;IPI00693338;IPI00694751;IPI00695508;IPI00695890;IPI00695965;IPI00700789;IPI00703268;IPI00703776;IPI00715354;IPI00871133;IPI01028487	Biological Process	Regulation of molecular function	4	14	211	0.7867
GO:0043069	IPI00692468;IPI00692676;IPI00694739;IPI00705660;IPI00708921	Biological Process	Negative regulation of programmed cell death	8	5	211	0.7880
GO:0009166	IPI00686225;IPI00712775	Biological Process	Nucleotide catabolic process	9	2	211	0.7887
GO:0019226	IPI00699355;IPI00707359	Biological Process	Transmission of nerve impulse	6	2	211	0.7887

GO:0071705	IPI00690232;IPI00704728	Biological Process	Nitrogen compound transport	6	2	211	0.7887
GO:0009141	IPI00703753;IPI00712775;IPI01028487	Biological Process	Nucleoside triphosphate metabolic process	9	3	211	0.7913
GO:0002237	IPI00687625	Biological Process	Response to molecule of bacterial origin	6	1	211	0.7918
GO:0008654	IPI00944429	Biological Process	Phospholipid biosynthetic process	6	1	211	0.7918
GO:0016055	IPI00714481	Biological Process	Wnt signaling pathway	8	1	211	0.7918
GO:0034329	IPI00714673	Biological Process	Cell junction assembly	6	1	211	0.7918
GO:0044403	IPI00707359	Biological Process	Symbiosis, encompassing mutualism through parasitism	5	1	211	0.7918
GO:0044419	IPI00707359	Biological Process	Interspecies interaction between organisms	4	1	211	0.7918
GO:0045765	IPI00716121	Biological Process	Regulation of angiogenesis	11	1	211	0.7918
GO:0046039	IPI00712775	Biological Process	GTP metabolic process	12	1	211	0.7918
GO:0046488	IPI00692819	Biological Process	Phosphatidylinositol metabolic process	9	1	211	0.7918
GO:0051168	IPI00691963	Biological Process	Nuclear export	9	1	211	0.7918
GO:0071214	IPI00703731	Biological Process	Cellular response to abiotic stimulus	5	1	211	0.7918
GO:0071363	IPI00686966	Biological Process	Cellular response to growth factor stimulus	7	1	211	0.7918
GO:0071826	IPI00701698	Biological Process	Ribonucleoprotein complex subunit organization	6	1	211	0.7918
GO:1901214	IPI00692468	Biological Process	Regulation of neuron death	7	1	211	0.7918

GO:0051716	IPI00685792;IPI00686966;IPI00687539;IPI00689750;IPI00690160;IPI00691963;IPI00692468;IPI00692819;IPI00693338;IPI00694504;IPI00694751;IPI00694851;IPI00695965;IPI00699355;IPI00700547;IPI00701698;IPI00703547;IPI00703731;IPI00703753;IPI00703776;IPI00705941;IPI00708921;IPI00713573;IPI00714481;IPI00715354;IPI00716843;IPI00718311;IPI00842934;IPI01018577	Biological Process	Cellular response to stimulus	4	29	211	0.7923
GO:0060548	IPI00692468;IPI00692676;IPI00694739;IPI00705660;IPI00708921	Biological Process	Negative regulation of cell death	7	5	211	0.7947
GO:0051641	IPI00686601;IPI00687601;IPI00687842;IPI00689750;IPI00691963;IPI00692911;IPI00693338;IPI00695666;IPI00699002;IPI00701223;IPI00702891;IPI00705941;IPI00706141;IPI00707359;IPI00708921;IPI00710408;IPI00714481;IPI00715339;IPI00904732	Biological Process	Cellular localization	4	19	211	0.7979
GO:1901658	IPI00686225;IPI00712775	Biological Process	Glycosyl compound catabolic process	6	2	211	0.7985

GO:1901576	IPI00686225;IPI00686601;IPI00689323;IPI00692676;IPI00692819;IPI00692911;IPI00693338;IPI00694214;IPI00694312;IPI00694739;IPI00694851;IPI00695890;IPI00696912;IPI00697184;IPI00701698;IPI00703776;IPI00703854;IPI00704728;IPI00706942;IPI00711759;IPI00712671;IPI00713536;IPI00715091;IPI00726650;IPI00742596;IPI00883375;IPI00944429;IPI01028487	Biological Process	Organic substance biosynthetic process	5	28	211	0.8003

GO:0006810	IPI00686601;IPI00687601;IPI00687842;IPI00688651;IPI00689362;IPI00689750;IPI00690160;IPI00690232;IPI00691212;IPI00691963;IPI00692911;IPI00693338;IPI00695142;IPI00695965;IPI00699002;IPI00699355;IPI00702891;IPI00703753;IPI00703776;IPI00703854;IPI00704728;IPI00705941;IPI00706141;IPI00706458;IPI00707359;IPI00708921;IPI00710408;IPI00716195;IPI00718381;IPI00838607;IPI00868597	Biological Process	Transport	5	31	211	0.8028
GO:0002250	IPI00701698	Biological Process	Adaptive immune response	5	1	211	0.8045
GO:0009896	IPI00695965	Biological Process	Positive regulation of catabolic process	6	1	211	0.8045
GO:0070848	IPI00686966	Biological Process	Response to growth factor	6	1	211	0.8045
GO:1901342	IPI00716121	Biological Process	Regulation of vasculature development	8	1	211	0.8045
GO:1901606	IPI00711419	Biological Process	Alpha-amino acid catabolic process	8	1	211	0.8045
GO:2001233	IPI00686966	Biological Process	Regulation of apoptotic signaling pathway	8	1	211	0.8045

GO:0044765	IPI00686601;IPI00687601;IPI00687842;IPI00688651;IPI00689750;IPI00690160;IPI00691963;IPI00693338;IPI00695142;IPI00695965;IPI00699355;IPI00702891;IPI00703753;IPI00703776;IPI00706141;IPI00706458;IPI00707359;IPI00708921;IPI00716195	Biological Process	Single-organism transport	4	19	211	0.8055
GO:0031399	IPI00690446;IPI00693338;IPI00703753;IPI00703776;IPI00715354;IPI00842934	Biological Process	Regulation of protein modification process	8	6	211	0.8056
GO:0002520	IPI00690094;IPI00692468;IPI00693338	Biological Process	Immune system development	4	3	211	0.8074
GO:0048858	IPI00687372;IPI00687601	Biological Process	Cell projection morphogenesis	6	2	211	0.8078
GO:0022414	IPI00689035;IPI00694504;IPI00697184;IPI00718311	Biological Process	Reproductive process	3	4	211	0.8127
GO:0032880	IPI00695506;IPI00697184;IPI00715354	Biological Process	Regulation of protein localization	6	3	211	0.8151
GO:0007018	IPI00687601	Biological Process	Microtubule-based movement	6	1	211	0.8165
GO:0009142	IPI01028487	Biological Process	Nucleoside triphosphate biosynthetic process	9	1	211	0.8165
GO:0009201	IPI01028487	Biological Process	Ribonucleoside triphosphate biosynthetic process	10	1	211	0.8165
GO:0050790	IPI00686966;IPI00692468;IPI00693338;IPI00694751;IPI00695890;IPI00695965;IPI00700789;IPI00703268;IPI00715354;IPI00871133;IPI01028487	Biological Process	Regulation of catalytic activity	5	11	211	0.8165

GO:0009966	IPI00686966;IPI00690094;IPI00691963;IPI00693338;IPI00694739;IPI00699355;IPI00700789;IPI00701698;IPI00703753;IPI00703776;IPI00707359;IPI00717119;IPI00718311;IPI00912603	Biological Process	Regulation of signal transduction	7	14	211	0.8168
GO:0048514	IPI00689035;IPI00840588	Biological Process	Blood vessel morphogenesis	9	2	211	0.8168
GO:0080135	IPI00715339;IPI01018577	Biological Process	Regulation of cellular response to stress	6	2	211	0.8168
GO:0033036	IPI00686601;IPI00689750;IPI00690232;IPI00691963;IPI00695666;IPI00695965;IPI00699355;IPI00701223;IPI00702891;IPI00703753;IPI00703776;IPI00703854;IPI00704728;IPI00705941;IPI00707359;IPI00708921;IPI00710408;IPI00714481;IPI00715339;IPI00904732	Biological Process	Macromolecule localization	4	20	211	0.8171
GO:0019439	IPI00686225;IPI00703753;IPI00712775;IPI00716555	Biological Process	Aromatic compound catabolic process	6	4	211	0.8195
GO:0006508	IPI00686966;IPI00695176;IPI00697757;IPI00700542;IPI00700547;IPI00703776;IPI00706624;IPI00707359;IPI00707875	Biological Process	Proteolysis	6	9	211	0.8221
GO:0006974	IPI00700547;IPI00715354;IPI01018577	Biological Process	Cellular response to DNA damage stimulus	6	3	211	0.8225

GO:0023051	IPI00686966;IPI00690094;IPI00691963;IPI00693338;IPI00694739;IPI00697184;IPI00699355;IPI00700789;IPI00701698;IPI00703753;IPI00703776;IPI00707359;IPI00717119;IPI00718311;IPI00912603	Biological Process	Regulation of signaling	4	15	211	0.8240
GO:0051179	IPI00686601;IPI00687601;IPI00687842;IPI00688651;IPI00689362;IPI00689750;IPI00690160;IPI00690232;IPI00691212;IPI00691963;IPI00692911;IPI00693338;IPI00693628;IPI00694751;IPI00695142;IPI00695666;IPI00695965;IPI00699002;IPI00699355;IPI00701223;IPI00702891;IPI00703753;IPI00703776;IPI00703854;IPI00704728;IPI00705941;IPI00706141;IPI00706458;IPI00707359;IPI00708921;IPI00710408;IPI00714481;IPI00715339;IPI00716195;IPI00718381;IPI00838607;IPI00868597;IPI00904732	Biological Process	Localization	3	38	211	0.8253
GO:0006396	IPI00685278;IPI00690232;IPI00695600;IPI00697891;IPI00700295;IPI00701698;IPI00717759	Biological Process	RNA processing	9	7	211	0.8266

GO:0051234	IPI00686601;IPI00687601;IPI00687842;IPI00688651;IPI00689362;IPI00689750;IPI00690160;IPI00690232;IPI00691212;IPI00691963;IPI00692911;IPI00693338;IPI00695142;IPI00695965;IPI00699002;IPI00699355;IPI00702891;IPI00703753;IPI00703776;IPI00703854;IPI00704728;IPI00705941;IPI00706141;IPI00706458;IPI00707359;IPI00708921;IPI00710408;IPI00716195;IPI00718381;IPI00838607;IPI00868597	Biological Process	Establishment of localization	4	31	211	0.8270
GO:0000377	IPI00717759	Biological Process	RNA splicing, via transesterification reactions with bulged adenosine as nucleophile	12	1	211	0.8277
GO:0000398	IPI00717759	Biological Process	mRNA splicing, via spliceosome	11	1	211	0.8277
GO:0009063	IPI00711419	Biological Process	Cellular amino acid catabolic process	7	1	211	0.8277
GO:1902105	IPI00693338	Biological Process	Regulation of leukocyte differentiation	8	1	211	0.8277
GO:0005975	IPI00689323;IPI00692819;IPI00697184;IPI00698589;IPI00699798;IPI00706203;IPI00706942	Biological Process	Carbohydrate metabolic process	5	7	211	0.8317
GO:0035637	IPI00699355;IPI00707359	Biological Process	Multicellular organismal signaling	5	2	211	0.8336

GO:0010646	IPI00686966;IPI00690094;IPI00691963;IPI00693338;IPI00694739;IPI00697184;IPI00699355;IPI00700789;IPI00701698;IPI00703753;IPI00703776;IPI00707359;IPI00717119;IPI00718311;IPI00912603	Biological Process	Regulation of cell communication	6	15	211	0.8352
GO:0006511	IPI00700547;IPI00703776;IPI00707875	Biological Process	Ubiquitin-dependent protein catabolic process	9	3	211	0.8366
GO:0000375	IPI00717759	Biological Process	RNA splicing, via transesterification reactions	11	1	211	0.8382
GO:0030031	IPI00688921	Biological Process	Cell projection assembly	6	1	211	0.8382
GO:0035023	IPI00703776	Biological Process	Regulation of Rho protein signal transduction	11	1	211	0.8382
GO:0048872	IPI00690160	Biological Process	Homeostasis of number of cells	6	1	211	0.8382
GO:0001934	IPI00693338;IPI00703776	Biological Process	Positive regulation of protein phosphorylation	10	2	211	0.8415

GO:0019538	IPI00686966;IPI00688608;IPI00688651;IPI00689325;IPI00690308;IPI00690785;IPI00691963;IPI00693338;IPI00695176;IPI00695965;IPI00696930;IPI00697081;IPI00697757;IPI00698900;IPI00699107;IPI00699798;IPI00700542;IPI00700547;IPI00700789;IPI00701698;IPI00701790;IPI00703731;IPI00703776;IPI00704728;IPI00706624;IPI00707359;IPI00707452;IPI00707875;IPI00713536;IPI00713757;IPI00714481;IPI00714673;IPI00715091;IPI00912603;IPI01018577	Biological Process	Protein metabolic process	5	35	211	0.8439
GO:0002443	IPI00701698	Biological Process	Leukocyte mediated immunity	5	1	211	0.8481
GO:0010038	IPI00695508	Biological Process	Response to metal ion	6	1	211	0.8481
GO:0031647	IPI00691963	Biological Process	Regulation of protein stability	9	1	211	0.8481
GO:0034220	IPI00716195	Biological Process	Ion transmembrane transport	6	1	211	0.8481
GO:0090150	IPI00708921	Biological Process	Establishment of protein localization to membrane	6	1	211	0.8481

GO:0009058	IPI00686225;IPI00686601;IPI00689323;IPI00692676;IPI00692819;IPI00692911;IPI00693338;IPI00694214;IPI00694312;IPI00694739;IPI00694851;IPI00695890;IPI00696912;IPI00697184;IPI00701698;IPI00703776;IPI00703854;IPI00704728;IPI00706942;IPI00711759;IPI00712671;IPI00713536;IPI00715091;IPI00726650;IPI00742596;IPI00883375;IPI00944429;IPI01028487	Biological Process	Biosynthetic process	4	28	211	0.8485
GO:0009057	IPI00695176;IPI00697757;IPI00700547;IPI00701790;IPI00703776;IPI00707875	Biological Process	Macromolecule catabolic process	6	6	211	0.8489
GO:0042327	IPI00693338;IPI00703776	Biological Process	Positive regulation of phosphorylation	9	2	211	0.8491
GO:0001932	IPI00693338;IPI00703753;IPI00703776;IPI00715354	Biological Process	Regulation of protein phosphorylation	9	4	211	0.8501
GO:0044723	IPI00689323;IPI00692819;IPI00697184;IPI00698589;IPI00706942	Biological Process	Single-organism carbohydrate metabolic process	6	5	211	0.8535
GO:0019941	IPI00700547;IPI00703776;IPI00707875	Biological Process	Modification-dependent protein catabolic process	8	3	211	0.8559
GO:0030900	IPI00686966	Biological Process	Forebrain development	10	1	211	0.8574
GO:0043405	IPI00693338	Biological Process	Regulation of MAP kinase activity	12	1	211	0.8574
GO:0034655	IPI00686225;IPI00703753;IPI00712775	Biological Process	Nucleobase-containing compound catabolic process	7	3	211	0.8619

GO:0043632	IPI00700547;IPI00703776;IPI00707875	Biological Process	Modification-dependent macromolecule catabolic process	7	3	211	0.8619
GO:0044265	IPI00695176;IPI00697757;IPI00700547;IPI00703776;IPI00707875	Biological Process	Cellular macromolecule catabolic process	6	5	211	0.8634
GO:0015672	IPI00716195	Biological Process	Monovalent inorganic cation transport	7	1	211	0.8661
GO:0034330	IPI00714673	Biological Process	Cell junction organization	5	1	211	0.8661
GO:0042254	IPI00701698	Biological Process	Ribosome biogenesis	6	1	211	0.8661
GO:0043123	IPI00690094	Biological Process	Positive regulation of I-kappaB kinase/NF-kappaB signaling	10	1	211	0.8661
GO:0045860	IPI00693338	Biological Process	Positive regulation of protein kinase activity	11	1	211	0.8661
GO:0044249	IPI00686225;IPI00686601;IPI00689323;IPI00692676;IPI00692819;IPI00692911;IPI00694214;IPI00694312;IPI00694739;IPI00694851;IPI00695890;IPI00696912;IPI00701698;IPI00703776;IPI00703854;IPI00704728;IPI00711759;IPI00712671;IPI00713536;IPI00715091;IPI00726650;IPI00742596;IPI00883375;IPI00944429;IPI01028487	Biological Process	Cellular biosynthetic process	5	25	211	0.8677
GO:0072594	IPI00689750;IPI00707359	Biological Process	Establishment of protein localization to organelle	8	2	211	0.8698
GO:0042176	IPI00732368	Biological Process	Regulation of protein catabolic process	8	1	211	0.8743
GO:0006887	IPI00706141	Biological Process	Exocytosis	6	1	211	0.8820
GO:0046822	IPI00715354	Biological Process	Regulation of nucleocytoplasmic transport	8	1	211	0.8820

GO:0008104	IPI00686601;IPI00689750;IPI00691963;IPI00695666;IPI00701223;IPI00702891;IPI00703776;IPI00703854;IPI00704728;IPI00705941;IPI00707359;IPI00708921;IPI00710408;IPI00714481;IPI00715339;IPI00904732	Biological Process	Protein localization	5	16	211	0.8820
GO:0019953	IPI00689035;IPI00697184	Biological Process	Sexual reproduction	4	2	211	0.8821
GO:0046903	IPI00702891;IPI00706141	Biological Process	Secretion	5	2	211	0.8821
GO:0043412	IPI00688608;IPI00688651;IPI00690308;IPI00693338;IPI00696930;IPI00697081;IPI00699107;IPI00699798;IPI00700789;IPI00703776;IPI00704728;IPI00714673;IPI01018577	Biological Process	Macromolecule modification	6	13	211	0.8877
GO:0007417	IPI00686966;IPI00689228	Biological Process	Central nervous system development	8	2	211	0.8879
GO:0051090	IPI00703776	Biological Process	Regulation of sequence-specific DNA binding transcription factor activity	12	1	211	0.8892
GO:0043085	IPI00686966;IPI00693338;IPI00694751;IPI00695890;IPI00695965	Biological Process	Positive regulation of catalytic activity	5	5	211	0.8899
GO:0042325	IPI00693338;IPI00703753;IPI00703776;IPI00715354	Biological Process	Regulation of phosphorylation	8	4	211	0.8899

GO:0006464	IPI00688608;IPI00688651;IPI00690308;IPI00693338;IPI00696930;IPI00697081;IPI00699798;IPI00700789;IPI00703776;IPI00704728;IPI00714673;IPI01018577	Biological Process	Cellular protein modification process	7	12	211	0.8933
GO:0036211	IPI00688608;IPI00688651;IPI00690308;IPI00693338;IPI00696930;IPI00697081;IPI00699798;IPI00700789;IPI00703776;IPI00704728;IPI00714673;IPI01018577	Biological Process	Protein modification process	6	12	211	0.8933
GO:0009205	IPI00712775;IPI01028487	Biological Process	Purine ribonucleoside triphosphate metabolic process	11	2	211	0.8933
GO:0007283	IPI00697184	Biological Process	Spermatogenesis	7	1	211	0.8960
GO:0046034	IPI01028487	Biological Process	ATP metabolic process	12	1	211	0.8960
GO:0048232	IPI00697184	Biological Process	Male gamete generation	6	1	211	0.8960
GO:0016070	IPI00685278;IPI00690232;IPI00692676;IPI00692911;IPI00694851;IPI00695600;IPI00697891;IPI00699107;IPI00700295;IPI00701698;IPI00703776;IPI00713642;IPI00717759	Biological Process	RNA metabolic process	8	13	211	0.8961
GO:0010562	IPI00693338;IPI00703776	Biological Process	Positive regulation of phosphorus metabolic process	6	2	211	0.8986
GO:0045937	IPI00693338;IPI00703776	Biological Process	Positive regulation of phosphate metabolic process	8	2	211	0.8986
GO:0009203	IPI00712775	Biological Process	Ribonucleoside triphosphate catabolic process	10	1	211	0.9024
GO:0009207	IPI00712775	Biological Process	Purine ribonucleoside triphosphate catabolic process	11	1	211	0.9024

GO:0034470	IPI00700295	Biological Process	ncRNA processing	10	1	211	0.9024
GO:0060562	IPI00707359	Biological Process	Epithelial tube morphogenesis	8	1	211	0.9024
GO:0009144	IPI00712775;IPI01028487	Biological Process	Purine nucleoside triphosphate metabolic process	10	2	211	0.9036
GO:0044267	IPI00688608;IPI00688651;IPI00689325;IPI00690308;IPI00690785;IPI00691963;IPI00693338;IPI00695176;IPI00696930;IPI00697081;IPI00697757;IPI00698900;IPI00699107;IPI00699798;IPI00700547;IPI00700789;IPI00703731;IPI00703776;IPI00704728;IPI00707452;IPI00707875;IPI00713536;IPI00714481;IPI00714673;IPI00715091;IPI01018577	Biological Process	Cellular protein metabolic process	6	26	211	0.9041
GO:0009146	IPI00712775	Biological Process	Purine nucleoside triphosphate catabolic process	10	1	211	0.9084
GO:0043122	IPI00690094	Biological Process	Regulation of I-kappaB kinase/NF-kappaB signaling	9	1	211	0.9084
GO:0001568	IPI00689035;IPI00840588	Biological Process	Blood vessel development	8	2	211	0.9084
GO:0009199	IPI00712775;IPI01028487	Biological Process	Ribonucleoside triphosphate metabolic process	10	2	211	0.9084
GO:0001944	IPI00689035;IPI00840588	Biological Process	Vasculature development	7	2	211	0.9129
GO:0016568	IPI00703854	Biological Process	Chromatin modification	7	1	211	0.9140
GO:0033674	IPI00693338	Biological Process	Positive regulation of kinase activity	10	1	211	0.9140
GO:0035239	IPI00707359	Biological Process	Tube morphogenesis	7	1	211	0.9140
GO:0051656	IPI00687601	Biological Process	Establishment of organelle localization	5	1	211	0.9140
GO:0006399	IPI00700295	Biological Process	tRNA metabolic process	10	1	211	0.9193
GO:0051347	IPI00693338	Biological Process	Positive regulation of transferase activity	6	1	211	0.9193

GO:0044260	IPI00685278;IPI00688608;IPI00688651;IPI00689325;IPI00690232;IPI00690308;IPI00690785;IPI00691963;IPI00692676;IPI00692911;IPI00693338;IPI00694214;IPI00694851;IPI00695176;IPI00695600;IPI00695890;IPI00696930;IPI00697081;IPI00697757;IPI00697891;IPI00698039;IPI00698900;IPI00699107;IPI00699798;IPI00700295;IPI00700547;IPI00700789;IPI00701698;IPI00703731;IPI00703776;IPI00703854;IPI00704728;IPI00705000;IPI00707452;IPI00707875;IPI00713536;IPI00713642;IPI00714481;IPI00714673;IPI00715091;IPI00717759;IP	Biological Process	Cellular macromolecule metabolic process	5	43	211	0.9212
GO:0007267	IPI00694504	Biological Process	Cell-cell signaling	6	1	211	0.9289
GO:0045859	IPI00693338;IPI00715354	Biological Process	Regulation of protein kinase activity	10	2	211	0.9291
GO:0007420	IPI00686966	Biological Process	Brain development	9	1	211	0.9332
GO:0006605	IPI00689750;IPI00707359	Biological Process	Protein targeting	6	2	211	0.9361
GO:0007276	IPI00697184	Biological Process	Gamete generation	5	1	211	0.9373
GO:0006886	IPI00689750;IPI00691963;IPI00705941;IPI00707359;IPI00708921;IPI00710408	Biological Process	Intracellular protein transport	7	6	211	0.9387
GO:0016192	IPI00690160;IPI00695142;IPI00703776;IPI00706141;IPI00708921;IPI00710408;IPI00718381;IPI00868597	Biological Process	Vesicle-mediated transport	6	8	211	0.9413

GO:0044702	IPI00689035;IPI00697184	Biological Process	Single organism reproductive process	4	2	211	0.9424
GO:0044802	IPI00686601;IPI00689750;IPI00708921	Biological Process	Single-organism membrane organization	4	3	211	0.9434
GO:0016567	IPI00703776	Biological Process	Protein ubiquitination	10	1	211	0.9448
GO:0071702	IPI00686601;IPI00689750;IPI00690232;IPI00691963;IPI00695965;IPI00699355;IPI00702891;IPI00703776;IPI00703854;IPI00704728;IPI00705941;IPI00707359;IPI00708921;IPI00710408	Biological Process	Organic substance transport	6	14	211	0.9455
GO:0008610	IPI00686601;IPI00944429	Biological Process	Lipid biosynthetic process	6	2	211	0.9482
GO:0051640	IPI00687601	Biological Process	Organelle localization	5	1	211	0.9482
GO:0031329	IPI00695965;IPI00703268;IPI00732368	Biological Process	Regulation of cellular catabolic process	6	3	211	0.9484
GO:0043549	IPI00693338;IPI00715354	Biological Process	Regulation of kinase activity	9	2	211	0.9508
GO:0051240	IPI00688651	Biological Process	Positive regulation of multicellular organismal process	5	1	211	0.9514
GO:0061024	IPI00686601;IPI00689750;IPI00708921	Biological Process	Membrane organization	5	3	211	0.9531
GO:0022613	IPI00701698	Biological Process	Ribonucleoprotein complex biogenesis	5	1	211	0.9544
GO:0055085	IPI00716195	Biological Process	Transmembrane transport	5	1	211	0.9544
GO:0007165	IPI00686966;IPI00689750;IPI00692468;IPI00692819;IPI00693338;IPI00694504;IPI00694851;IPI00699355;IPI00701698;IPI00703776;IPI00705941;IPI00708921;IPI00714481;IPI00715354;IPI00716843	Biological Process	Signal transduction	6	15	211	0.9572

GO:0009059	IPI00692676;IPI00692911;IPI00693338;IPI00694214;IPI00694851;IPI00695890;IPI00701698;IPI00703776;IPI00703854;IPI00704728;IPI00713536;IPI00715091	Biological Process	Macromolecule biosynthetic process	6	12	211	0.9612
GO:0048193	IPI00708921	Biological Process	Golgi vesicle transport	6	1	211	0.9623
GO:0043623	IPI00691963	Biological Process	Cellular protein complex assembly	8	1	211	0.9646
GO:0032446	IPI00703776	Biological Process	Protein modification by small protein conjugation	9	1	211	0.9668
GO:0015031	IPI00689750;IPI00691963;IPI00702891;IPI00703776;IPI00703854;IPI00704728;IPI00705941;IPI00707359;IPI00708921;IPI00710408	Biological Process	Protein transport	6	10	211	0.9677
GO:0009894	IPI00695965;IPI00703268;IPI00732368	Biological Process	Regulation of catabolic process	5	3	211	0.9679
GO:0046907	IPI00687601;IPI00689750;IPI00691963;IPI00692911;IPI00693338;IPI00699002;IPI00705941;IPI00707359;IPI00708921;IPI00710408	Biological Process	Intracellular transport	6	10	211	0.9688
GO:0045184	IPI00689750;IPI00691963;IPI00702891;IPI00703776;IPI00703854;IPI00704728;IPI00705941;IPI00707359;IPI00708921;IPI00710408	Biological Process	Establishment of protein localization	6	10	211	0.9698
GO:0007166	IPI00693338;IPI00708921;IPI00714481;IPI00716843	Biological Process	Cell surface receptor signaling pathway	7	4	211	0.9703

GO:0009118	IPI00703268	Biological Process	Regulation of nucleoside metabolic process	9	1	211	0.9707
GO:0030811	IPI00703268	Biological Process	Regulation of nucleotide catabolic process	10	1	211	0.9707
GO:0033121	IPI00703268	Biological Process	Regulation of purine nucleotide catabolic process	11	1	211	0.9707
GO:0051649	IPI00687601;IPI00689750;IPI00691963;IPI00692911;IPI00693338;IPI00699002;IPI00702891;IPI00705941;IPI00706141;IPI00707359;IPI00708921;IPI00710408	Biological Process	Establishment of localization in cell	5	12	211	0.9717
GO:0034645	IPI00692676;IPI00692911;IPI00694214;IPI00694851;IPI00695890;IPI00701698;IPI00703776;IPI00703854;IPI00704728;IPI00713536;IPI00715091	Biological Process	Cellular macromolecule biosynthetic process	6	11	211	0.9764
GO:0048609	IPI00697184	Biological Process	Multicellular organismal reproductive process	5	1	211	0.9787
GO:0032504	IPI00697184	Biological Process	Multicellular organism reproduction	4	1	211	0.9800
GO:0023052	IPI00686966;IPI00689750;IPI00692468;IPI00692819;IPI00693338;IPI00694504;IPI00694851;IPI00699355;IPI00701698;IPI00703776;IPI00705941;IPI00707359;IPI00708921;IPI00714481;IPI00715354;IPI00716843	Biological Process	Signaling	3	16	211	0.9804

GO:0044700	IPI00686966;IPI00689750;IPI00692468;IPI00692819;IPI00693338;IPI00694504;IPI00694851;IPI00699355;IPI00701698;IPI00703776;IPI00705941;IPI00707359;IPI00708921;IPI00714481;IPI00715354;IPI00716843	Biological Process	Single organism signaling	4	16	211	0.9804
GO:0046578	IPI00703776	Biological Process	Regulation of Ras protein signal transduction	10	1	211	0.9813
GO:0007154	IPI00686966;IPI00689750;IPI00692468;IPI00692819;IPI00693338;IPI00694504;IPI00694851;IPI00695666;IPI00699355;IPI00701698;IPI00703776;IPI00705941;IPI00707359;IPI00708921;IPI00714481;IPI00715354;IPI00716843	Biological Process	Cell communication	5	17	211	0.9816
GO:0010467	IPI00685278;IPI00690232;IPI00692676;IPI00692911;IPI00694851;IPI00695600;IPI00697891;IPI00700295;IPI00701698;IPI00703776;IPI00704728;IPI00713536;IPI00715091;IPI00717759	Biological Process	Gene expression	6	14	211	0.9816
GO:0006140	IPI00703268	Biological Process	Regulation of nucleotide metabolic process	10	1	211	0.9845
GO:0034660	IPI00700295	Biological Process	NcRNA metabolic process	9	1	211	0.9845
GO:1900542	IPI00703268	Biological Process	Regulation of purine nucleotide metabolic process	11	1	211	0.9845

GO:0006412	IPI00704728;IPI00713536;IPI00715091	Biological Process	Translation	7	3	211	0.9847
GO:0051056	IPI00703776	Biological Process	Regulation of small GTPase mediated signal transduction	9	1	211	0.9855
GO:0019220	IPI00693338;IPI00703268;IPI00703753;IPI00703776;IPI00715354	Biological Process	Regulation of phosphate metabolic process	7	5	211	0.9873
GO:0051174	IPI00693338;IPI00703268;IPI00703753;IPI00703776;IPI00715354	Biological Process	Regulation of phosphorus metabolic process	6	5	211	0.9873
GO:0007264	IPI00705941	Biological Process	Small GTPase mediated signal transduction	8	1	211	0.9880
GO:0070647	IPI00703776	Biological Process	Protein modification by small protein conjugation or removal	8	1	211	0.9918
GO:0035556	IPI00686966;IPI00701698;IPI00705941;IPI00715354	Biological Process	Intracellular signal transduction	7	4	211	0.9955
GO:0005576	IPI00687539;IPI00688608;IPI00689035;IPI00689362;IPI00689760;IPI00690094;IPI00691212;IPI00691669;IPI00691963;IPI00694504;IPI00694751;IPI00695142;IPI00695489;IPI00695965;IPI00696930;IPI00697081;IPI00697184;IPI00697196;IPI00698993;IPI00700542;IPI00701166;IPI00701698;IPI00702700;IPI00703753;IPI00704877;IPI00705660;IPI00706624;IPI00707101;IPI00707359;IPI00707587;IPI00709922;IPI00710385;IPI00712366;IPI00713573;IPI00713757;IPI00714673;IPI00715339;IPI00715354;IPI00715486;IPI00716121;IPI00716123;IP	Cellular Component	Extracellular region	3	52	211	0.0000

GO:0031012	IPI00688608;IPI00689760;IPI00690094;IPI00694504;IPI00696930;IPI00697081;IPI00700542;IPI00706624;IPI00707359;IPI00707587;IPI00709922;IPI00710385;IPI00712366;IPI00713573;IPI00714673;IPI00715339;IPI00715486;IPI00716121;IPI00716123;IPI00716158;IPI00717116;IPI00717119;IPI00718698;IPI00837992;IPI00840588;IPI00923985	Cellular Component	Extracellular matrix	3	26	211	0.0000

GO:0044421	IPI00687539;IPI00688608;IPI00689760;IPI00690094;IPI00691212;IPI00691963;IPI00694504;IPI00695489;IPI00695965;IPI00696930;IPI00697081;IPI00700542;IPI00701166;IPI00703753;IPI00705660;IPI00706624;IPI00707101;IPI00707359;IPI00707587;IPI00709922;IPI00710385;IPI00712366;IPI00714673;IPI00715339;IPI00715486;IPI00716121;IPI00716123;IPI00716158;IPI00717116;IPI00717119;IPI00718311;IPI00718698;IPI00837992;IPI00840588;IPI00871133;IPI00923985	Cellular Component	Extracellular region part	4	36	211	0.0000

GO:0005615	IPI00687539;IPI00688608;IPI00690094;IPI00691212;IPI00691963;IPI00694504;IPI00695489;IPI00695965;IPI00696930;IPI00700542;IPI00701166;IPI00703753;IPI00705660;IPI00706624;IPI00707101;IPI00707359;IPI00707587;IPI00709922;IPI00712366;IPI00714673;IPI00715339;IPI00716121;IPI00716158;IPI00717116;IPI00718311;IPI00718698;IPI00837992;IPI00840588;IPI00871133;IPI00923985	Cellular Component	Extracellular space	5	30	211	0.0000
GO:0005578	IPI00688608;IPI00689760;IPI00690094;IPI00694504;IPI00697081;IPI00706624;IPI00707359;IPI00707587;IPI00709922;IPI00710385;IPI00712366;IPI00714673;IPI00715486;IPI00716123;IPI00716158;IPI00717119;IPI00718698;IPI00840588	Cellular Component	Proteinaceous extracellular matrix	5	18	211	0.0000

GO:0005882	IPI00685418;IPI00689228;IPI00689750;IPI00692588;IPI00694204;IPI00694214;IPI00708921;IPI00721270;IPI00905865	Cellular Component	Intermediate filament	11	9	211	0.0000
GO:0045111	IPI00685418;IPI00689228;IPI00689750;IPI00692588;IPI00694204;IPI00694214;IPI00703731;IPI00708921;IPI00721270;IPI00905865	Cellular Component	Intermediate filament cytoskeleton	10	10	211	0.0001
GO:0005884	IPI00687539;IPI00688489;IPI00689005;IPI00690160;IPI00695506;IPI00698900;IPI00727017	Cellular Component	Actin filament	11	7	211	0.0007
GO:0005788	IPI00689325;IPI00691963;IPI00699107;IPI00702891;IPI00704729;IPI00708234;IPI00716493	Cellular Component	Endoplasmic reticulum lumen	11	7	211	0.0025
GO:0042383	IPI00688651;IPI00694204;IPI00697081;IPI00707359;IPI00713573;IPI00837992;IPI00905865	Cellular Component	Sarcolemma	6	7	211	0.0025
GO:0045095	IPI00685418;IPI00694214;IPI00708921;IPI00721270	Cellular Component	Keratin filament	12	4	211	0.0031
GO:0044448	IPI00688921;IPI00690160;IPI00698900;IPI00699803;IPI00703380;IPI00707718;IPI00727017;IPI00904732	Cellular Component	Cell cortex part	10	8	211	0.0083

GO:0015629	IPI00687539;IPI00688489;IPI00688921;IPI00689005;IPI00690160;IPI00692676;IPI00695506;IPI00695666;IPI00698039;IPI00698900;IPI00707718;IPI00713141;IPI00714405;IPI00727017;IPI00829551	Cellular Component	Actin cytoskeleton	10	15	211	0.0126
GO:0005856	IPI00685418;IPI00687539;IPI00687601;IPI00687657;IPI00688489;IPI00688921;IPI00689005;IPI00689228;IPI00689750;IPI00690160;IPI00692588;IPI00692676;IPI00694204;IPI00694214;IPI00694751;IPI00695506;IPI00695508;IPI00695666;IPI00698039;IPI00698900;IPI00699803;IPI00703268;IPI00703380;IPI00703731;IPI00707359;IPI00707718;IPI00708921;IPI00711750;IPI00713141;IPI00714405;IPI00718757;IPI00721270;IPI00727017;IPI00829551;IPI00904732;IPI00905865	Cellular Component	Cytoskeleton	9	36	211	0.0136

GO:0044430	IPI00685418;IPI00687539;IPI00687601;IPI00688489;IPI00688921;IPI00689005;IPI00689228;IPI00689750;IPI00690160;IPI00692588;IPI00694204;IPI00694214;IPI00695506;IPI00695508;IPI00698900;IPI00699803;IPI00703380;IPI00707718;IPI00708921;IPI00713141;IPI00714405;IPI00721270;IPI00727017;IPI00829551;IPI00904732;IPI00905865	Cellular Component	Cytoskeletal part	10	26	211	0.0176
GO:0042641	IPI00688489;IPI00707718;IPI00713141;IPI00714405;IPI00727017;IPI00829551	Cellular Component	Actomyosin	11	6	211	0.0195
GO:0033018	IPI00691963;IPI00716493	Cellular Component	Sarcoplasmic reticulum lumen	10	2	211	0.0202
GO:0005938	IPI00688921;IPI00690160;IPI00698900;IPI00699803;IPI00703380;IPI00706141;IPI00707718;IPI00727017;IPI00904732	Cellular Component	Cell cortex	9	9	211	0.0240
GO:0030424	IPI00687372;IPI00688651;IPI00703380;IPI00703547;IPI00707359;IPI00707718	Cellular Component	Axon	7	6	211	0.0250
GO:0031941	IPI00687539;IPI00698900;IPI00727017	Cellular Component	Filamentous actin	12	3	211	0.0252

GO:0044420	IPI00694504;IPI00706624;IPI00707359;IPI00709922;IPI00714673;IPI00715339;IPI00715486;IPI00716158	Cellular Component	Extracellular matrix part	4	8	211	0.0267
GO:0031674	IPI00688489;IPI00694204;IPI00706458;IPI00713141;IPI00905865	Cellular Component	I band	12	5	211	0.0281
GO:0030016	IPI00688489;IPI00694204;IPI00698900;IPI00706458;IPI00713141;IPI00905865	Cellular Component	Myofibril	10	6	211	0.0315
GO:0016528	IPI00688651;IPI00691963;IPI00716493	Cellular Component	Sarcoplasm	8	3	211	0.0322
GO:0016529	IPI00688651;IPI00691963;IPI00716493	Cellular Component	Sarcoplasmic reticulum	9	3	211	0.0322
GO:0031105	IPI00699803;IPI00703380;IPI00707718	Cellular Component	Septin complex	11	3	211	0.0322
GO:0032156	IPI00699803;IPI00703380;IPI00707718	Cellular Component	Septin cytoskeleton	10	3	211	0.0322
GO:0001725	IPI00707718;IPI00713141;IPI00714405;IPI00727017;IPI00829551	Cellular Component	Stress fiber	12	5	211	0.0412
GO:0030863	IPI00688921;IPI00690160;IPI00698900;IPI00727017;IPI00904732	Cellular Component	Cortical cytoskeleton	11	5	211	0.0412
GO:0043292	IPI00688489;IPI00694204;IPI00698900;IPI00706458;IPI00713141;IPI00905865	Cellular Component	Contractile fiber	9	6	211	0.0430

GO:0042995	IPI00687372;IPI00687539;IPI00687657;IPI00688651;IPI00688921;IPI00689228;IPI00690001;IPI00690160;IPI00693755;IPI00695506;IPI00703380;IPI00703547;IPI00703776;IPI00707359;IPI00707718;IPI00711750;IPI00829551;IPI00904732	Cellular Component	Cell projection	5	18	211	0.0439
GO:0005604	IPI00694504;IPI00707359;IPI00709922;IPI00714673;IPI00715486	Cellular Component	Basement membrane	6	5	211	0.0463
GO:0032432	IPI00707718;IPI00713141;IPI00714405;IPI00727017;IPI00829551	Cellular Component	Actin filament bundle	11	5	211	0.0463
GO:0044297	IPI00687372;IPI00687539;IPI00688651;IPI00689323;IPI00703547	Cellular Component	Cell body	5	5	211	0.0463
GO:0031252	IPI00687539;IPI00687657;IPI00688921;IPI00689228;IPI00690160;IPI00693755;IPI00695506;IPI00707359;IPI00829551	Cellular Component	Cell leading edge	5	9	211	0.0544
GO:0030017	IPI00688489;IPI00694204;IPI00706458;IPI00713141;IPI00905865	Cellular Component	Sarcomere	11	5	211	0.0574
GO:0033267	IPI00688651;IPI00703380;IPI00707359	Cellular Component	Axon part	8	3	211	0.0582
GO:0044449	IPI00688489;IPI00694204;IPI00706458;IPI00713141;IPI00905865	Cellular Component	Contractile fiber part	10	5	211	0.0768

GO:0030018	IPI00694204;IPI00706458;IPI00713141;IPI00905865	Cellular Component	Z disc	13	4	211	0.0771
GO:0001891	IPI00690160;IPI00695506	Cellular Component	Phagocytic cup	7	2	211	0.0804
GO:0044304	IPI00688651;IPI00707359	Cellular Component	Main axon	9	2	211	0.0804
GO:0043005	IPI00687372;IPI00687539;IPI00688651;IPI00690001;IPI00703380;IPI00703547;IPI00707359;IPI00707718	Cellular Component	Neuron projection	6	8	211	0.0818
GO:0005839	IPI00695176;IPI00697757;IPI00707875	Cellular Component	Proteasome core complex	8	3	211	0.0915
GO:0042470	IPI00689325;IPI00690001;IPI00702891;IPI00705941;IPI00716121;IPI00716493	Cellular Component	Melanosome	12	6	211	0.0921
GO:0048770	IPI00689325;IPI00690001;IPI00702891;IPI00705941;IPI00716121;IPI00716493	Cellular Component	Pigment granule	11	6	211	0.0921

GO:0071944	IPI00685792;IPI00686966;IPI00687372;IPI00688651;IPI00688921;IPI00689228;IPI00690160;IPI00691963;IPI00693338;IPI00694204;IPI00694751;IPI00695142;IPI00695506;IPI00695666;IPI00697081;IPI00698900;IPI00698993;IPI00699803;IPI00701698;IPI00702028;IPI00703129;IPI00703380;IPI00703753;IPI00703776;IPI00706141;IPI00706458;IPI00707359;IPI00707718;IPI00708018;IPI00708438;IPI00710408;IPI00713573;IPI00714481;IPI00714673;IPI00716843;IPI00721270;IPI00727017;IPI00837992;IPI00904732;IPI00905865;IPI00912603	Cellular Component	Cell periphery	5	41	211	0.0921
GO:0043025	IPI00687372;IPI00688651;IPI00689323;IPI00703547	Cellular Component	Neuronal cell body	6	4	211	0.0951

GO:0031974	IPI00685278;IPI00685418;IPI00686966;IPI00689325;IPI00689750;IPI00690446;IPI00691963;IPI00692468;IPI00692911;IPI00694126;IPI00694851;IPI00695331;IPI00695600;IPI00695890;IPI00698039;IPI00698900;IPI00699107;IPI00699798;IPI00701698;IPI00702620;IPI00702650;IPI00702891;IPI00703731;IPI00704729;IPI00707359;IPI00707559;IPI00708018;IPI00708234;IPI00708311;IPI00711419;IPI00716493;IPI00732368;IPI01018577	Cellular Component	Membrane-enclosed lumen	3	33	211	0.0967

GO:0043234	IPI00685418;IPI00686601;IPI00686966;IPI00687539;IPI00687601;IPI00688489;IPI00688921;IPI00689005;IPI00689228;IPI00689750;IPI00690160;IPI00690446;IPI00691963;IPI00692588;IPI00693324;IPI00694204;IPI00694214;IPI00694851;IPI00695176;IPI00695331;IPI00695506;IPI00695508;IPI00697757;IPI00698039;IPI00698900;IPI00698993;IPI00699798;IPI00699803;IPI00700295;IPI00700547;IPI00701223;IPI00701790;IPI00702650;IPI00703129;IPI00703380;IPI00704728;IPI00705000;IPI00707359;IPI00707718;IPI00707875;IPI00708311;IP	Cellular Component	Protein complex	4	57	211	0.1122
GO:0097458	IPI00687372;IPI00687539;IPI00688651;IPI00689323;IPI00690001;IPI00703380;IPI00703547;IPI00707359;IPI00707718	Cellular Component	Neuron part	5	9	211	0.1137
GO:0005921	IPI00695666	Cellular Component	Gap junction	5	1	211	0.1176
GO:0009279	IPI00707359	Cellular Component	Cell outer membrane	7	1	211	0.1176
GO:0016011	IPI00707359	Cellular Component	Dystroglycan complex	8	1	211	0.1176
GO:0016507	IPI00702650	Cellular Component	Mitochondrial fatty acid beta-oxidation multienzyme complex	12	1	211	0.1176
GO:0016592	IPI00694851	Cellular Component	Mediator complex	13	1	211	0.1176
GO:0030313	IPI00707359	Cellular Component	Cell envelope	6	1	211	0.1176
GO:0030981	IPI00904732	Cellular Component	Cortical microtubule cytoskeleton	12	1	211	0.1176
GO:0031595	IPI00732368	Cellular Component	Nuclear proteasome complex	8	1	211	0.1176
GO:0031597	IPI00732368	Cellular Component	Cytosolic proteasome complex	8	1	211	0.1176

GO:0033011	IPI00688921	Cellular Component	Perinuclear theca	9	1	211	0.1176
GO:0033150	IPI00688921	Cellular Component	Cytoskeletal calyx	10	1	211	0.1176
GO:0036125	IPI00702650	Cellular Component	Fatty acid beta-oxidation multienzyme complex	5	1	211	0.1176
GO:0042643	IPI00688489	Cellular Component	Actomyosin, actin portion	12	1	211	0.1176
GO:0044462	IPI00707359	Cellular Component	External encapsulating structure part	6	1	211	0.1176
GO:0071953	IPI00715339	Cellular Component	Elastic fiber	5	1	211	0.1176
GO:0000777	IPI00701223;IPI00703380	Cellular Component	Condensed chromosome kinetochore	10	2	211	0.1195
GO:0070013	IPI00685278;IPI00685418;IPI00689325;IPI00689750;IPI00690446;IPI00691963;IPI00692468;IPI00692911;IPI00694126;IPI00694851;IPI00695331;IPI00695600;IPI00695890;IPI00698039;IPI00698900;IPI00699107;IPI00699798;IPI00701698;IPI00702620;IPI00702650;IPI00702891;IPI00703731;IPI00704729;IPI00707359;IPI00707559;IPI00708018;IPI00708234;IPI00711419;IPI00716493;IPI00732368;IPI01018577	Cellular Component	Intracellular organelle lumen	9	31	211	0.1197

GO:0043233	IPI00685278;IPI00685418;IPI00689325;IPI00689750;IPI00690446;IPI00691963;IPI00692468;IPI00692911;IPI00694126;IPI00694851;IPI00695331;IPI00695600;IPI00695890;IPI00698039;IPI00698900;IPI00699107;IPI00699798;IPI00701698;IPI00702620;IPI00702650;IPI00702891;IPI00703731;IPI00704729;IPI00707359;IPI00707559;IPI00708018;IPI00708234;IPI00711419;IPI00716493;IPI00732368;IPI01018577	Cellular Component	Organelle lumen	4	31	211	0.1225

GO:0043228	IPI00685418;IPI00687539;IPI00687601;IPI00687657;IPI00688489;IPI00688921;IPI00689005;IPI00689228;IPI00689750;IPI00690160;IPI00692588;IPI00692676;IPI00694126;IPI00694204;IPI00694214;IPI00694751;IPI00695331;IPI00695506;IPI00695508;IPI00695666;IPI00695890;IPI00698039;IPI00698900;IPI00699798;IPI00699803;IPI00701223;IPI00701698;IPI00702650;IPI00703268;IPI00703380;IPI00703731;IPI00703776;IPI00705000;IPI00706458;IPI00707359;IPI00707718;IPI00708018;IPI00708921;IPI00711419;IPI00711750;IPI00713141;IP	Cellular Component	Non-membrane-bounded organelle	4	51	211	0.1271

GO:0043232	IPI00685418;IPI00687539;IPI00687601;IPI00687657;IPI00688489;IPI00688921;IPI00689005;IPI00689228;IPI00689750;IPI00690160;IPI00692588;IPI00692676;IPI00694126;IPI00694204;IPI00694214;IPI00694751;IPI00695331;IPI00695506;IPI00695508;IPI00695666;IPI00695890;IPI00698039;IPI00698900;IPI00699798;IPI00699803;IPI00701223;IPI00701698;IPI00702650;IPI00703268;IPI00703380;IPI00703731;IPI00703776;IPI00705000;IPI00706458;IPI00707359;IPI00707718;IPI00708018;IPI00708921;IPI00711419;IPI00711750;IPI00713141;IP	Cellular Component	Intracellular non-membrane-bounded organelle	8	51	211	0.1271
GO:0030027	IPI00687539;IPI00687657;IPI00688921;IPI00690160;IPI00707359	Cellular Component	Lamellipodium	6	5	211	0.1339
GO:0045202	IPI00688651;IPI00703380;IPI00707359;IPI00707718;IPI00839134;IPI00905865	Cellular Component	Synapse	3	6	211	0.1366
GO:0000779	IPI00701223;IPI00703380	Cellular Component	Condensed chromosome, centromeric region	11	2	211	0.1404
GO:0000786	IPI00698039;IPI00705000	Cellular Component	Nucleosome	12	2	211	0.1620
GO:1990104	IPI00698039;IPI00705000	Cellular Component	DNA bending complex	5	2	211	0.1620
GO:0005614	IPI00694504	Cellular Component	Interstitial matrix	6	1	211	0.1711
GO:0030312	IPI00707359	Cellular Component	External encapsulating structure	5	1	211	0.1711
GO:0030877	IPI00708311	Cellular Component	Beta-catenin destruction complex	9	1	211	0.1711
GO:0043194	IPI00688651	Cellular Component	Axon initial segment	9	1	211	0.1711

GO:0031965	IPI00690001;IPI00696729;IPI00702028;IPI00702620	Cellular Component	Nuclear membrane	12	4	211	0.1835
GO:0031594	IPI00688651;IPI00905865	Cellular Component	Neuromuscular junction	4	2	211	0.1842
GO:0097060	IPI00707359;IPI00839134	Cellular Component	Synaptic membrane	7	2	211	0.1842
GO:0000502	IPI00695176;IPI00697757;IPI00701790;IPI00707875;IPI00732368	Cellular Component	Proteasome complex	7	5	211	0.1942
GO:0030425	IPI00690001;IPI00703547;IPI00707718	Cellular Component	Dendrite	7	3	211	0.2065
GO:0005783	IPI00688651;IPI00689325;IPI00690785;IPI00691963;IPI00692911;IPI00696616;IPI00696729;IPI00699002;IPI00699107;IPI00702891;IPI00704728;IPI00704729;IPI00708234;IPI00714481;IPI00716493;IPI00732368;IPI00838607;IPI00847093	Cellular Component	Endoplasmic reticulum	9	18	211	0.2212
GO:0002102	IPI00727017	Cellular Component	Podosome	9	1	211	0.2214
GO:0005610	IPI00715486	Cellular Component	Laminin-5 complex	9	1	211	0.2214
GO:0012510	IPI00710408	Cellular Component	Trans-Golgi network transport vesicle membrane	13	1	211	0.2214
GO:0030130	IPI00710408	Cellular Component	Clathrin coat of trans-Golgi network vesicle	14	1	211	0.2214
GO:0030315	IPI00688651	Cellular Component	T-tubule	7	1	211	0.2214
GO:0030670	IPI00690160	Cellular Component	Phagocytic vesicle membrane	13	1	211	0.2214
GO:0033017	IPI00688651	Cellular Component	Sarcoplasmic reticulum membrane	10	1	211	0.2214
GO:0033268	IPI00707359	Cellular Component	Node of Ranvier	9	1	211	0.2214
GO:0045120	IPI00697891	Cellular Component	Pronucleus	10	1	211	0.2214
GO:0071203	IPI00688921	Cellular Component	WASH complex	9	1	211	0.2214
GO:0072669	IPI00700295	Cellular Component	tRNA-splicing ligase complex	7	1	211	0.2214

GO:0005793	IPI00696729;IPI00714673;IPI00838607	Cellular Component	Endoplasmic reticulum-Golgi intermediate compartment	9	3	211	0.2227
GO:0005635	IPI00689750;IPI00690001;IPI00696729;IPI00702028;IPI00702620;IPI00704728;IPI00708311	Cellular Component	Nuclear envelope	11	7	211	0.2270
GO:0044463	IPI00687539;IPI00688651;IPI00703380;IPI00707359;IPI00707718;IPI00904732	Cellular Component	Cell projection part	6	6	211	0.2384
GO:0005667	IPI00690446;IPI00695331;IPI00842934	Cellular Component	Transcription factor complex	7	3	211	0.2391
GO:0014704	IPI00688651;IPI00905865	Cellular Component	Intercalated disc	6	2	211	0.2527
GO:0032993	IPI00698039;IPI00705000	Cellular Component	Protein-DNA complex	4	2	211	0.2527
GO:0044291	IPI00688651;IPI00905865	Cellular Component	Cell-cell contact zone	5	2	211	0.2527
GO:0044456	IPI00703380;IPI00707359;IPI00839134	Cellular Component	Synapse part	3	3	211	0.2558
GO:0009986	IPI00687372;IPI00691963;IPI00693338;IPI00695965;IPI00697081;IPI00702891;IPI00703753	Cellular Component	Cell surface	5	7	211	0.2643

GO:0031981	IPI00685278;IPI00685418;IPI00689750;IPI00690446;IPI00692911;IPI00694126;IPI00694851;IPI00695331;IPI00695600;IPI00698039;IPI00698900;IPI00699798;IPI00701698;IPI00702620;IPI00702650;IPI00703731;IPI00707359;IPI00708018;IPI00711419;IPI00732368;IPI01018577	Cellular Component	Nuclear lumen	11	21	211	0.2675
GO:0000791	IPI01018577	Cellular Component	Euchromatin	12	1	211	0.2687
GO:0005638	IPI00689750	Cellular Component	Lamin filament	11	1	211	0.2687
GO:0005640	IPI00696729	Cellular Component	Nuclear outer membrane	8	1	211	0.2687
GO:0005719	IPI01018577	Cellular Component	Nuclear euchromatin	14	1	211	0.2687
GO:0005771	IPI00705660	Cellular Component	Multivesicular body	11	1	211	0.2687
GO:0016010	IPI00707359	Cellular Component	Dystrophin-associated glycoprotein complex	7	1	211	0.2687
GO:0019005	IPI00700547	Cellular Component	SCF ubiquitin ligase complex	9	1	211	0.2687
GO:0033179	IPI00716195	Cellular Component	Proton-transporting V-type ATPase, V0 domain	7	1	211	0.2687
GO:0034451	IPI00708921	Cellular Component	Centriolar satellite	10	1	211	0.2687
GO:0035267	IPI00698900	Cellular Component	NuA4 histone acetyltransferase complex	11	1	211	0.2687
GO:0035371	IPI00904732	Cellular Component	Microtubule plus-end	12	1	211	0.2687
GO:0042824	IPI00691963	Cellular Component	MHC class I peptide loading complex	14	1	211	0.2687
GO:0043189	IPI00698900	Cellular Component	H4/H2A histone acetyltransferase complex	10	1	211	0.2687
GO:0043679	IPI00703380	Cellular Component	Axon terminus	9	1	211	0.2687
GO:0070938	IPI00707359	Cellular Component	Contractile ring	5	1	211	0.2687

GO:0044432	IPI00688651;IPI00689325;IPI00691963;IPI00696616;IPI00699107;IPI00702891;IPI00704728;IPI00704729;IPI00708234;IPI00716493;IPI00732368	Cellular Component	Endoplasmic reticulum part	10	11	211	0.2766
GO:0005654	IPI00685278;IPI00685418;IPI00690446;IPI00694851;IPI00695600;IPI00698039;IPI00698900;IPI00699798;IPI00702620;IPI00707359;IPI01018577	Cellular Component	Nucleoplasm	11	11	211	0.2992
GO:0005925	IPI00687657;IPI00692676;IPI00693338;IPI00829551	Cellular Component	Focal adhesion	6	4	211	0.3036
GO:0034399	IPI00685418;IPI00689750;IPI00692911	Cellular Component	Nuclear periphery	12	3	211	0.3064
GO:0044459	IPI00685792;IPI00686966;IPI00687372;IPI00688651;IPI00690160;IPI00691963;IPI00693338;IPI00695142;IPI00695506;IPI00698993;IPI00707359;IPI00710408;IPI00714673; IPI00716843;IPI00904732	Cellular Component	Plasma membrane part	6	15	211	0.3088
GO:0005641	IPI00708311	Cellular Component	Nuclear envelope lumen	12	1	211	0.3131
GO:0005720	IPI01018577	Cellular Component	Nuclear heterochromatin	14	1	211	0.3131
GO:0005881	IPI00904732	Cellular Component	Cytoplasmic microtubule	9	1	211	0.3131
GO:0008290	IPI00688921	Cellular Component	F-actin capping protein complex	11	1	211	0.3131
GO:0016460	IPI00713141	Cellular Component	Myosin II complex	12	1	211	0.3131
GO:0031143	IPI00703776	Cellular Component	Pseudopodium	6	1	211	0.3131
GO:0035631	IPI00686966	Cellular Component	CD40 receptor complex	8	1	211	0.3131

GO:0043034	IPI00694204	Cellular Component	Costamere	11	1	211	0.3131
GO:0043197	IPI00707718	Cellular Component	Dendritic spine	8	1	211	0.3131
GO:0070688	IPI00699798	Cellular Component	MLL5-L complex	9	1	211	0.3131
GO:0072357	IPI00699798	Cellular Component	PTW/PP1 phosphatase complex	6	1	211	0.3131
GO:0005924	IPI00687657;IPI00692676;IPI00693338;IPI00829551	Cellular Component	Cell-substrate adherens junction	5	4	211	0.3177
GO:0005905	IPI00703776;IPI00710408	Cellular Component	Coated pit	5	2	211	0.3217
GO:0001772	IPI00690160	Cellular Component	Immunological synapse	7	1	211	0.3548
GO:0005577	IPI00714673	Cellular Component	Fibrinogen complex	5	1	211	0.3548
GO:0005583	IPI00716158	Cellular Component	Fibrillar collagen trimer	6	1	211	0.3548
GO:0005652	IPI00689750	Cellular Component	Nuclear lamina	11	1	211	0.3548
GO:0005916	IPI00905865	Cellular Component	Fascia adherens	6	1	211	0.3548
GO:0030140	IPI00710408	Cellular Component	Trans-Golgi network transport vesicle	12	1	211	0.3548
GO:0030666	IPI00690160	Cellular Component	Endocytic vesicle membrane	12	1	211	0.3548
GO:0042612	IPI00698993	Cellular Component	MHC class I protein complex	8	1	211	0.3548
GO:0043256	IPI00715486	Cellular Component	Laminin complex	8	1	211	0.3548
GO:0044306	IPI00703380	Cellular Component	Neuron projection terminus	7	1	211	0.3548
GO:0044309	IPI00707718	Cellular Component	Neuron spine	7	1	211	0.3548
GO:0009897	IPI00687372;IPI00691963;IPI00693338	Cellular Component	External side of plasma membrane	6	3	211	0.3574
GO:0000785	IPI00698039;IPI00703776;IPI00705000;IPI01018577	Cellular Component	Chromatin	11	4	211	0.3599
GO:0009295	IPI00695890;IPI00702650	Cellular Component	Nucleoid	3	2	211	0.3669
GO:0042645	IPI00695890;IPI00702650	Cellular Component	Mitochondrial nucleoid	12	2	211	0.3669

GO:0005886	IPI00685792;IPI00686966;IPI00687372;IPI00688651;IPI00689228;IPI00690160;IPI00691963;IPI00693338;IPI00694204;IPI00694751;IPI00695142;IPI00695506;IPI00695666;IPI00697081;IPI00698993;IPI00701698;IPI00702028;IPI00703129;IPI00703753;IPI00703776;IPI00706458;IPI00707359;IPI00708018;IPI00708438;IPI00710408;IPI00713573;IPI00714481;IPI00714673;IPI00716843;IPI00837992;IPI00904732;IPI00905865;IPI00912603	Cellular Component	Plasma membrane	5	33	211	0.3687
GO:0005912	IPI00687657;IPI00692676;IPI00693338;IPI00829551;IPI00905865	Cellular Component	Adherens junction	5	5	211	0.3837
GO:0030055	IPI00687657;IPI00692676;IPI00693338;IPI00829551	Cellular Component	Cell-substrate junction	4	4	211	0.3881
GO:0005798	IPI00710408;IPI00868597	Cellular Component	Golgi-associated vesicle	11	2	211	0.3891
GO:0001726	IPI00693755;IPI00695506;IPI00829551	Cellular Component	Ruffle	6	3	211	0.3912
GO:0030136	IPI00703380;IPI00710408;IPI00868597	Cellular Component	Clathrin-coated vesicle	12	3	211	0.3912
GO:0005876	IPI00695508	Cellular Component	Spindle microtubule	10	1	211	0.3940
GO:0019773	IPI00707875	Cellular Component	Proteasome core complex, alpha-subunit complex	9	1	211	0.3940
GO:0042611	IPI00698993	Cellular Component	MHC protein complex	7	1	211	0.3940

GO:0042627	IPI00695965	Cellular Component	Chylomicron	7	1	211	0.3940
GO:0005730	IPI00694126;IPI00695331;IPI00698039;IPI00699798;IPI00701698;IPI00702650;IPI00703731;IPI00708018;IPI00711419	Cellular Component	Nucleolus	11	9	211	0.3988
GO:0031970	IPI00686966;IPI00708311	Cellular Component	Organelle envelope lumen	10	2	211	0.4109
GO:0019867	IPI00696729;IPI00703129;IPI00707359	Cellular Component	Outer membrane	4	3	211	0.4245
GO:0000792	IPI01018577	Cellular Component	Heterochromatin	12	1	211	0.4308
GO:0030125	IPI00710408	Cellular Component	Clathrin vesicle coat	11	1	211	0.4308
GO:0030132	IPI00710408	Cellular Component	Clathrin coat of coated pit	11	1	211	0.4308
GO:0044450	IPI00708921	Cellular Component	Microtubule organizing center part	9	1	211	0.4308
GO:0022627	IPI00713536;IPI00715091	Cellular Component	Cytosolic small ribosomal subunit	12	2	211	0.4324

GO:0032991	IPI00685278;IPI00685418;IPI00686601;IPI00686966;IPI00687539;IPI00687601;IPI00687625;IPI00688489;IPI00688921;IPI00689005;IPI00689228;IPI00689750;IPI00690160;IPI00690446;IPI00691963;IPI00692588;IPI00693324;IPI00694204;IPI00694214;IPI00694851;IPI00695176;IPI00695331;IPI00695506;IPI00695508;IPI00695600;IPI00695965;IPI00697757;IPI00698039;IPI00698900;IPI00698993;IPI00699798;IPI00699803;IPI00700295;IPI00700547;IPI00701223;IPI00701790;IPI00702650;IPI00703129;IPI00703380;IPI00704728;IPI00705000;IP	Cellular Component	Macromolecular complex	3	64	211	0.4354

GO:0005634	IPI00685278;IPI00685418;IPI00686601;IPI00686803;IPI00686841;IPI00687625;IPI00688651;IPI00689005;IPI00689750;IPI00690001;IPI00690232;IPI00690446;IPI00691963;IPI00692676;IPI00692819;IPI00692911;IPI00693755;IPI00694126;IPI00694214;IPI00694580;IPI00694851;IPI00695176;IPI00695331;IPI00695600;IPI00696729;IPI00697757;IPI00697891;IPI00698039;IPI00698900;IPI00699002;IPI00699107;IPI00699355;IPI00699798;IPI00701223;IPI00701698;IPI00701790;IPI00702028;IPI00702620;IPI00702650;IPI00703547;IPI00703731;IP	Cellular Component	Nucleus	9	64	211	0.4465
GO:0000793	IPI00701223;IPI00703380	Cellular Component	Condensed chromosome	10	2	211	0.4534
GO:0016363	IPI00685418;IPI00692911	Cellular Component	Nuclear matrix	11	2	211	0.4534
GO:0045121	IPI00707359;IPI00843257	Cellular Component	Membrane raft	5	2	211	0.4534
GO:0005777	IPI00686601;IPI00689228;IPI00944429	Cellular Component	Peroxisome	10	3	211	0.4571
GO:0042579	IPI00686601;IPI00689228;IPI00944429	Cellular Component	Microbody	9	3	211	0.4571
GO:0005605	IPI00715486	Cellular Component	Basal lamina	7	1	211	0.4654
GO:0033176	IPI00716195	Cellular Component	Proton-transporting V-type ATPase complex	6	1	211	0.4654
GO:0045211	IPI00707359	Cellular Component	Postsynaptic membrane	8	1	211	0.4654
GO:0045335	IPI00690160	Cellular Component	Phagocytic vesicle	12	1	211	0.4654

GO:0070161	IPI00687657;IPI00692676;IPI00693338;IPI00829551;IPI00905865	Cellular Component	Anchoring junction	4	5	211	0.4692
GO:0030054	IPI00687657;IPI00688651;IPI00692676;IPI00693338;IPI00695666;IPI00707359;IPI00707718;IPI00829551;IPI00905865	Cellular Component	Cell junction	3	9	211	0.4786
GO:0008021	IPI00703380	Cellular Component	Synaptic vesicle	11	1	211	0.4979
GO:0031461	IPI00700547	Cellular Component	Cullin-RING ubiquitin ligase complex	8	1	211	0.4979
GO:0033177	IPI00716195	Cellular Component	Proton-transporting two-sector ATPase complex, proton-transporting domain	6	1	211	0.4979
GO:0034361	IPI00695965	Cellular Component	Very-low-density lipoprotein particle	8	1	211	0.4979
GO:0034385	IPI00695965	Cellular Component	Triglyceride-rich lipoprotein particle	7	1	211	0.4979
GO:0044427	IPI00698039;IPI00701223;IPI00703380;IPI00703776;IPI00705000;IPI01018577	Cellular Component	Chromosomal part	10	6	211	0.5101
GO:0000776	IPI00701223;IPI00703380	Cellular Component	Kinetochore	9	2	211	0.5137
GO:0005581	IPI00706624;IPI00716158	Cellular Component	Collagen trimer	3	2	211	0.5137
GO:0015935	IPI00713536;IPI00715091	Cellular Component	Small ribosomal subunit	11	2	211	0.5137
GO:0016324	IPI00695142;IPI00714673	Cellular Component	Apical plasma membrane	6	2	211	0.5137
GO:0030133	IPI00697081;IPI00710408	Cellular Component	Transport vesicle	11	2	211	0.5137

GO:0044446	IPI00685278;IPI00685418;IPI00686966;IPI00687539;IPI00687601;IPI00688489;IPI00688651;IPI00688921;IPI00689005;IPI00689228;IPI00689325;IPI00689750;IPI00690001;IPI00690160;IPI00690446;IPI00691963;IPI00692468;IPI00692588;IPI00692911;IPI00694126;IPI00694204;IPI00694214;IPI00694849;IPI00694851;IPI00695331;IPI00695506;IPI00695508;IPI00695600;IPI00695890;IPI00696616;IPI00696729;IPI00698039;IPI00698900;IPI00699107;IPI00699798;IPI00699803;IPI00701223;IPI00701698;IPI00702028;IPI00702620;IPI00702650;IP	Cellular Component	Intracellular organelle part	8	77	211	0.5237
GO:0005844	IPI00691963	Cellular Component	Polysome	8	1	211	0.5285
GO:0019897	IPI00685792;IPI00716843	Cellular Component	Extrinsic component of plasma membrane	7	2	211	0.5328
GO:0005819	IPI00695508;IPI00703380;IPI00904732	Cellular Component	Spindle	9	3	211	0.5350

GO:0044428	IPI00685278;IPI00685418;IPI00689750;IPI00690001;IPI00690446;IPI00692911;IPI00694126;IPI00694851;IPI00695331;IPI00695600;IPI00696729;IPI00698039;IPI00698900;IPI00699798;IPI00701698;IPI00702028;IPI00702620;IPI00702650;IPI00703731;IPI00704728;IPI00707359;IPI00708018;IPI00708311;IPI00711419;IPI00732368;IPI00839224;IPI01018577	Cellular Component	Nuclear part	10	27	211	0.5418

GO:0044422	IPI00685278;IPI00685418;IPI00686966;IPI00687539;IPI00687601;IPI00688489;IPI00688651;IPI00688921;IPI00689005;IPI00689228;IPI00689325;IPI00689750;IPI00690001;IPI00690160;IPI00690446;IPI00691963;IPI00692468;IPI00692588;IPI00692911;IPI00694126;IPI00694204;IPI00694214;IPI00694849;IPI00694851;IPI00695331;IPI00695506;IPI00695508;IPI00695600;IPI00695890;IPI00696616;IPI00696729;IPI00698039;IPI00698900;IPI00699107;IPI00699798;IPI00699803;IPI00701223;IPI00701698;IPI00702028;IPI00702620;IPI00702650;IP	Cellular Component	Organelle part	4	78	211	0.5503
GO:0009898	IPI00686966;IPI00716843	Cellular Component	Cytoplasmic side of plasma membrane	6	2	211	0.5513
GO:0048471	IPI00688921;IPI00689750;IPI00691963;IPI00692911;IPI00699002;IPI00868597	Cellular Component	Perinuclear region of cytoplasm	9	6	211	0.5519
GO:0016023	IPI00689325;IPI00690001;IPI00690160;IPI00697081;IPI00702891;IPI00703380;IPI00703776;IPI00705941;IPI00710408;IPI00716121;IPI00716493;IPI00868597	Cellular Component	Cytoplasmic membrane-bounded vesicle	10	12	211	0.5532
GO:0000123	IPI00698900	Cellular Component	Histone acetyltransferase complex	8	1	211	0.5572
GO:0005834	IPI00716843	Cellular Component	Heterotrimeric G-protein complex	7	1	211	0.5572

GO:0030426	IPI00687539	Cellular Component	Growth cone	6	1	211	0.5572
GO:0030427	IPI00687539	Cellular Component	Site of polarized growth	5	1	211	0.5572
GO:0031901	IPI00718381	Cellular Component	Early endosome membrane	11	1	211	0.5572
GO:0034364	IPI00695965	Cellular Component	High-density lipoprotein particle	7	1	211	0.5572
GO:0005759	IPI00692468;IPI00695890;IPI00701698;IPI00702650;IPI00707559	Cellular Component	Mitochondrial matrix	11	5	211	0.5838
GO:0005852	IPI00693324	Cellular Component	Eukaryotic translation initiation factor 3 complex	9	1	211	0.5841
GO:0030660	IPI00710408	Cellular Component	Golgi-associated vesicle membrane	12	1	211	0.5841
GO:0031988	IPI00689325;IPI00690001;IPI00690160;IPI00697081;IPI00702891;IPI00703380;IPI00703776;IPI00705941;IPI00710408;IPI00716121;IPI00716493;IPI00868597	Cellular Component	membrane-bounded vesicle	5	12	211	0.6032
GO:0030496	IPI00703380;IPI00711573	Cellular Component	Midbody	5	2	211	0.6039
GO:0030658	IPI00710408	Cellular Component	Transport vesicle membrane	12	1	211	0.6094
GO:0031982	IPI00689325;IPI00690001;IPI00690160;IPI00697081;IPI00702891;IPI00703380;IPI00703776;IPI00705941;IPI00706458;IPI00710408;IPI00716121;IPI00716493;IPI00868597	Cellular Component	Vesicle	4	13	211	0.6098

GO:0031410	IPI00689325;IPI00690001;IPI00690160;IPI00697081;IPI00702891;IPI00703380;IPI00703776;IPI00705941;IPI00710408;IPI00716121;IPI00716493;IPI00868597	Cellular Component	Cytoplasmic vesicle	9	12	211	0.6101
GO:0005694	IPI00698039;IPI00701223;IPI00703380;IPI00703776;IPI00705000;IPI01018577	Cellular Component	Chromosome	9	6	211	0.6113
GO:0031975	IPI00686966;IPI00689750;IPI00690001;IPI00692468;IPI00694849;IPI00696729;IPI00702028;IPI00702620;IPI00702650;IPI00703129;IPI00704728;IPI00707359;IPI00708311;IPI00708438;IPI00711233;IPI00712677;IPI00839134	Cellular Component	Envelope	5	17	211	0.6160
GO:0016459	IPI00713141	Cellular Component	Myosin complex	11	1	211	0.6332
GO:0030665	IPI00710408	Cellular Component	Clathrin-coated vesicle membrane	13	1	211	0.6332
GO:0030864	IPI00690160	Cellular Component	Cortical actin cytoskeleton	12	1	211	0.6332
GO:0031234	IPI00716843	Cellular Component	Extrinsic component of cytoplasmic side of plasma membrane	7	1	211	0.6332
GO:0032994	IPI00695965	Cellular Component	Protein-lipid complex	4	1	211	0.6332
GO:0034358	IPI00695965	Cellular Component	Plasma lipoprotein particle	6	1	211	0.6332
GO:0000775	IPI00701223;IPI00703380	Cellular Component	Chromosome, centromeric region	12	2	211	0.6364
GO:0046930	IPI00703129;IPI00704728	Cellular Component	Pore complex	7	2	211	0.6518
GO:0000922	IPI00695508	Cellular Component	Spindle pole	10	1	211	0.6556
GO:0005758	IPI00686966	Cellular Component	Mitochondrial intermembrane space	12	1	211	0.6556

GO:0034708	IPI00699798	Cellular Component	Methyltransferase complex	7	1	211	0.6556
GO:0035097	IPI00699798	Cellular Component	Histone methyltransferase complex	8	1	211	0.6556
GO:0030135	IPI00703380;IPI00710408;IPI00868597	Cellular Component	Coated vesicle	11	3	211	0.6578
GO:0031968	IPI00696729;IPI00703129	Cellular Component	Organelle outer membrane	7	2	211	0.6667
GO:0005875	IPI00687601	Cellular Component	Microtubule associated complex	11	1	211	0.6766
GO:0005913	IPI00905865	Cellular Component	Cell-cell adherens junction	5	1	211	0.6766
GO:0017053	IPI00690446	Cellular Component	Transcriptional repressor complex	5	1	211	0.6766
GO:0030175	IPI00707359	Cellular Component	Filopodium	6	1	211	0.6766
GO:0005769	IPI00716195;IPI00718381	Cellular Component	Early endosome	10	2	211	0.6810
GO:0016607	IPI00695600;IPI00702620	Cellular Component	Nuclear speck	14	2	211	0.6810
GO:0005829	IPI00686225;IPI00686966;IPI00687539;IPI00687601;IPI00689228;IPI00690001;IPI00691963;IPI00695506;IPI00695508;IPI00698900;IPI00700789;IPI00703547;IPI00703776;IPI00713536;IPI00713642;IPI00715091;IPI00732368	Cellular Component	Cytosol	9	17	211	0.6887
GO:0031967	IPI00686966;IPI00689750;IPI00690001;IPI00692468;IPI00694849;IPI00696729;IPI00702028;IPI00702620;IPI00702650;IPI00703129;IPI00704728;IPI00708311;IPI00708438;IPI00711233;IPI00712677;IPI00839134	Cellular Component	Organelle envelope	9	16	211	0.7011

GO:0044451	IPI00690446;IPI00694851;IPI00695600;IPI00698900;IPI00699798;IPI00702620	Cellular Component	Nucleoplasm part	12	6	211	0.7080
GO:0022626	IPI00713536;IPI00715091	Cellular Component	Cytosolic ribosome	11	2	211	0.7083
GO:0008287	IPI00699798	Cellular Component	Protein serine/threonine phosphatase complex	5	1	211	0.7148
GO:0032153	IPI00703380	Cellular Component	Cell division site	5	1	211	0.7148
GO:0032154	IPI00703380	Cellular Component	Cleavage furrow	7	1	211	0.7148
GO:0032155	IPI00703380	Cellular Component	Cell division site part	6	1	211	0.7148
GO:0019898	IPI00685792;IPI00716843	Cellular Component	Extrinsic component of membrane	5	2	211	0.7212
GO:0022624	IPI00701790	Cellular Component	Proteasome accessory complex	8	1	211	0.7322
GO:0030118	IPI00710408	Cellular Component	Clathrin coat	10	1	211	0.7322
GO:0043235	IPI00686966	Cellular Component	Receptor complex	5	1	211	0.7322
GO:0045177	IPI00695142;IPI00714673	Cellular Component	Apical part of cell	5	2	211	0.7336
GO:0005911	IPI00688651;IPI00695666;IPI00905865	Cellular Component	Cell-cell junction	4	3	211	0.7360

GO:0005575	IPI00685278;IPI00685418;IPI00685792;IPI00686183;IPI00686225;IPI00686601;IPI00686803;IPI00686841;IPI00686966;IPI00687372;IPI00687539;IPI00687601;IPI00687625;IPI00687657;IPI00687842;IPI00688489;IPI00688608;IPI00688651;IPI00688921;IPI00689005;IPI00689228;IPI00689323;IPI00689325;IPI00689750;IPI00689760;IPI00690001;IPI00690094;IPI00690160;IPI00690232;IPI00690446;IPI00690785;IPI00691212;IPI00691963;IPI00692468;IPI00692588;IPI00692627;IPI00692676;IPI00692819;IPI00692911;IPI00693324;IPI00693338;IP	Cellular Component	Cellular_component	2	179	211	0.7413
GO:0000790	IPI01018577	Cellular Component	Nuclear chromatin	13	1	211	0.7639
GO:0016469	IPI00716195	Cellular Component	Proton-transporting two-sector ATPase complex	5	1	211	0.7639
GO:0030120	IPI00710408	Cellular Component	Vesicle coat	10	1	211	0.7639
GO:0042175	IPI00688651;IPI00691963;IPI00696616;IPI00696729;IPI00704728;IPI00732368	Cellular Component	Nuclear outer membrane-endoplasmic reticulum membrane network	6	6	211	0.7740
GO:0005802	IPI00868597	Cellular Component	Trans-Golgi network	10	1	211	0.7783
GO:0030139	IPI00690160	Cellular Component	Endocytic vesicle	11	1	211	0.7783
GO:0044391	IPI00713536;IPI00715091	Cellular Component	Ribosomal subunit	10	2	211	0.7887
GO:0030176	IPI00691963	Cellular Component	Integral component of endoplasmic reticulum membrane	13	1	211	0.7918
GO:0071013	IPI00695600;IPI00839224	Cellular Component	Catalytic step 2 spliceosome	9	2	211	0.7985
GO:0005813	IPI00695508;IPI00708921;IPI00904732	Cellular Component	Centrosome	9	3	211	0.7995

GO:0000151	IPI00700547	Cellular Component	Ubiquitin ligase complex	7	1	211	0.8045
GO:0005770	IPI00705660	Cellular Component	Late endosome	10	1	211	0.8045
GO:0044445	IPI00713536;IPI00715091;IPI00732368	Cellular Component	Cytosolic part	10	3	211	0.8074
GO:0070469	IPI00711233;IPI00712677	Cellular Component	Respiratory chain	5	2	211	0.8078
GO:0005643	IPI00704728	Cellular Component	Nuclear pore	12	1	211	0.8165
GO:0005747	IPI00711233	Cellular Component	Mitochondrial respiratory chain complex I	15	1	211	0.8165
GO:0030964	IPI00711233	Cellular Component	NADH dehydrogenase complex	6	1	211	0.8165
GO:0045271	IPI00711233	Cellular Component	Respiratory chain complex I	7	1	211	0.8165
GO:0031227	IPI00691963	Cellular Component	Intrinsic component of endoplasmic reticulum membrane	12	1	211	0.8382
GO:0031253	IPI00904732	Cellular Component	Cell projection membrane	7	1	211	0.8382
GO:0005681	IPI00685278;IPI00695600;IPI00839224	Cellular Component	Spliceosomal complex	8	3	211	0.8497
GO:0030662	IPI00710408	Cellular Component	Coated vesicle membrane	12	1	211	0.8574
GO:0005789	IPI00688651;IPI00691963;IPI00696616;IPI00704728;IPI00732368	Cellular Component	Endoplasmic reticulum membrane	11	5	211	0.8585
GO:0005741	IPI00703129	Cellular Component	Mitochondrial outer membrane	13	1	211	0.8661
GO:0005746	IPI00711233	Cellular Component	Mitochondrial respiratory chain	14	1	211	0.8661
GO:0044454	IPI01018577	Cellular Component	Nuclear chromosome part	12	1	211	0.8743
GO:0031966	IPI00686966;IPI00692468;IPI00694849;IPI00702650;IPI00703129;IPI00708438;IPI00711233;IPI00712677;IPI00839134	Cellular Component	Mitochondrial membrane	12	9	211	0.8759
GO:0030659	IPI00690160;IPI00710408	Cellular Component	Cytoplasmic vesicle membrane	11	2	211	0.8761
GO:0016604	IPI00695600;IPI00702620	Cellular Component	Nuclear body	13	2	211	0.8821

GO:0005743	IPI00692468;IPI00694849;IPI00702650;IPI00708438;IPI00711233;IPI00712677;IPI00839134	Cellular Component	Mitochondrial inner membrane	13	7	211	0.8838
GO:0005874	IPI00695508;IPI00904732	Cellular Component	Microtubule	11	2	211	0.8879
GO:0012505	IPI00688651;IPI00689750;IPI00690001;IPI00691963;IPI00696616;IPI00696729;IPI00702028;IPI00702620;IPI00703776;IPI00704728;IPI00708311;IPI00710408;IPI00732368	Cellular Component	Endomembrane system	5	13	211	0.8906
GO:0044433	IPI00690160;IPI00710408	Cellular Component	Cytoplasmic vesicle part	10	2	211	0.8986
GO:0005740	IPI00686966;IPI00692468;IPI00694849;IPI00702650;IPI00703129;IPI00708438;IPI00711233;IPI00712677;IPI00839134	Cellular Component	Mitochondrial envelope	11	9	211	0.8989
GO:0019866	IPI00692468;IPI00694849;IPI00702650;IPI00708438;IPI00711233;IPI00712677;IPI00839134	Cellular Component	Organelle inner membrane	7	7	211	0.9012
GO:0044429	IPI00686966;IPI00692468;IPI00694849;IPI00695890;IPI00701698;IPI00702650;IPI00703129;IPI00707559;IPI00708438;IPI00711233;IPI00712677;IPI00839134	Cellular Component	Mitochondrial part	10	12	211	0.9042

GO:0005815	IPI00695508;IPI00708921;IPI00904732	Cellular Component	Microtubule organizing center	11	3	211	0.9067
GO:0000228	IPI01018577	Cellular Component	Nuclear chromosome	11	1	211	0.9140
GO:0030117	IPI00710408	Cellular Component	Membrane coat	9	1	211	0.9140
GO:0031301	IPI00691963	Cellular Component	Integral component of organelle membrane	7	1	211	0.9140
GO:0048475	IPI00710408	Cellular Component	Coated membrane	4	1	211	0.9140
GO:0012506	IPI00690160;IPI00710408	Cellular Component	Vesicle membrane	5	2	211	0.9173
GO:0005887	IPI00686966	Cellular Component	Integral component of plasma membrane	7	1	211	0.9289
GO:0031300	IPI00691963	Cellular Component	Intrinsic component of organelle membrane	6	1	211	0.9373
GO:0043229	IPI00685278;IPI00685418;IPI00686601;IPI00686803;IPI00686841;IPI00686966;IPI00687539;IPI00687601;IPI00687625;IPI00687657;IPI00687842;IPI00688489;IPI00688651;IPI00688921;IPI00689005;IPI00689228;IPI00689325;IPI00689750;IPI00690001;IPI00690160;IPI00690232;IPI00690446;IPI00690785;IPI00691963;IPI00692468;IPI00692588;IPI00692676;IPI00692819;IPI00692911;IPI00693755;IPI00694126;IPI00694204;IPI00694214;IPI00694580;IPI00694751;IPI00694849;IPI00694851;IPI00695176;IPI00695331;IPI00695506;IPI00695508;IP	Cellular Component	Intracellular organelle	7	126	211	0.9405

GO:0043226	IPI00685278;IPI00685418;IPI00686601;IPI00686803;IPI00686841;IPI00686966;IPI00687539;IPI00687601;IPI00687625;IPI00687657;IPI00687842;IPI00688489;IPI00688651;IPI00688921;IPI00689005;IPI00689228;IPI00689325;IPI00689750;IPI00690001;IPI00690160;IPI00690232;IPI00690446;IPI00690785;IPI00691963;IPI00692468;IPI00692588;IPI00692676;IPI00692819;IPI00692911;IPI00693755;IPI00694126;IPI00694204;IPI00694214;IPI00694580;IPI00694751;IPI00694849;IPI00694851;IPI00695176;IPI00695331;IPI00695506;IPI00695508;IP	Cellular Component	Organelle	3	126	211	0.9426
GO:0010008	IPI00718381	Cellular Component	Endosome membrane	11	1	211	0.9482
GO:0044440	IPI00718381	Cellular Component	Endosomal part	10	1	211	0.9482
GO:0031226	IPI00686966	Cellular Component	Intrinsic component of plasma membrane	7	1	211	0.9514
GO:0005768	IPI00705660;IPI00716195;IPI00718381	Cellular Component	Endosome	9	3	211	0.9630
GO:0000323	IPI00718311	Cellular Component	Lytic vacuole	10	1	211	0.9668
GO:0005764	IPI00718311	Cellular Component	Lysosome	11	1	211	0.9668
GO:0044431	IPI00710408;IPI00868597	Cellular Component	Golgi apparatus part	10	2	211	0.9696
GO:0005840	IPI00713536;IPI00715091	Cellular Component	Ribosome	9	2	211	0.9727
GO:0044455	IPI00711233	Cellular Component	Mitochondrial membrane part	13	1	211	0.9758

GO:0005794	IPI00694214;IPI00696729;IPI00710408;IPI00718311;IPI00838607;IPI00868597;IPI00904732	Cellular Component	Golgi apparatus	9	7	211	0.9829
GO:0005773	IPI00718311	Cellular Component	Vacuole	9	1	211	0.9855
GO:0000139	IPI00710408	Cellular Component	Golgi membrane	11	1	211	0.9864
GO:0031090	IPI00686966;IPI00688651;IPI00690001;IPI00690160;IPI00691963;IPI00692468;IPI00694849;IPI00696616;IPI00696729;IPI00702028;IPI00702620;IPI00702650;IPI00703129;IPI00704728;IPI00708438;IPI00710408;IPI00711233;IPI00712677;IPI00718381;IPI00732368;IPI00839134	Cellular Component	Organelle membrane	4	21	211	0.9866

GO:0044444	IPI00686225;IPI00686601;IPI00686803;IPI00686966;IPI00687539;IPI00687601;IPI00687842;IPI00688489;IPI00688651;IPI00688921;IPI00689228;IPI00689325;IPI00689750;IPI00690001;IPI00690160;IPI00690785;IPI00691963;IPI00692468;IPI00692819;IPI00692911;IPI00693324;IPI00694204;IPI00694214;IPI00694849;IPI00695506;IPI00695508;IPI00695890;IPI00696616;IPI00696729;IPI00697081;IPI00698900;IPI00699002;IPI00699107;IPI00699803;IPI00700789;IPI00701698;IPI00702650;IPI00702891;IPI00703129;IPI00703380;IPI00703547;IP	Cellular Component	Cytoplasmic part	8	80	211	0.9870
GO:0015630	IPI00687601;IPI00695508;IPI00703380;IPI00708921;IPI00904732	Cellular Component	Microtubule cytoskeleton	10	5	211	0.9873

GO:0005737	IPI00685418;IPI00686183;IPI00686225;IPI00686601;IPI00686803;IPI00686966;IPI00687539;IPI00687601;IPI00687657;IPI00687842;IPI00688489;IPI00688651;IPI00688921;IPI00689228;IPI00689323;IPI00689325;IPI00689750;IPI00690001;IPI00690160;IPI00690785;IPI00691963;IPI00692468;IPI00692627;IPI00692676;IPI00692819;IPI00692911;IPI00693324;IPI00693755;IPI00694126;IPI00694204;IPI00694214;IPI00694312;IPI00694580;IPI00694739;IPI00694751;IPI00694849;IPI00695176;IPI00695506;IPI00695508;IPI00695890;IPI00696616;IP	Cellular Component	Cytoplasm	7	119	211	0.9880
GO:0030529	IPI00685278;IPI00687625;IPI00691963;IPI00695600;IPI00713536;IPI00715091;IPI00839224	Cellular Component	Ribonucleoprotein complex	7	7	211	0.9927

GO:0005623	IPI00685278;IPI00685418;IPI00685792;IPI00686183;IPI00686225;IPI00686601;IPI00686803;IPI00686841;IPI00686966;IPI00687372;IPI00687539;IPI00687601;IPI00687625;IPI00687657;IPI00687842;IPI00688489;IPI00688651;IPI00688921;IPI00689005;IPI00689228;IPI00689323;IPI00689325;IPI00689750;IPI00690001;IPI00690094;IPI00690160;IPI00690232;IPI00690446;IPI00690785;IPI00691963;IPI00692468;IPI00692588;IPI00692627;IPI00692676;IPI00692819;IPI00692911;IPI00693324;IPI00693338;IPI00693755;IPI00694126;IPI00694204;IP	Cellular Component	Cell	3	155	211	0.9957

GO:0044464	IPI00685278;IPI00685418;IPI00685792;IPI00686183;IPI00686225;IPI00686601;IPI00686803;IPI00686841;IPI00686966;IPI00687372;IPI00687539;IPI00687601;IPI00687625;IPI00687657;IPI00687842;IPI00688489;IPI00688651;IPI00688921;IPI00689005;IPI00689228;IPI00689323;IPI00689325;IPI00689750;IPI00690001;IPI00690094;IPI00690160;IPI00690232;IPI00690446;IPI00690785;IPI00691963;IPI00692468;IPI00692588;IPI00692627;IPI00692676;IPI00692819;IPI00692911;IPI00693324;IPI00693338;IPI00693755;IPI00694126;IPI00694204;IP	Cellular Component	Cell part	4	155	211	0.9957

GO:0044424	IPI00685278;IPI00685418;IPI00686183;IPI00686225;IPI00686601;IPI00686803;IPI00686841;IPI00686966;IPI00687539;IPI00687601;IPI00687625;IPI00687657;IPI00687842;IPI00688489;IPI00688651;IPI00688921;IPI00689005;IPI00689228;IPI00689323;IPI00689325;IPI00689750;IPI00690001;IPI00690160;IPI00690232;IPI00690446;IPI00690785;IPI00691963;IPI00692468;IPI00692588;IPI00692627;IPI00692676;IPI00692819;IPI00692911;IPI00693324;IPI00693755;IPI00694126;IPI00694204;IPI00694214;IPI00694312;IPI00694580;IPI00694739;IP	Cellular Component	Intracellular part	6	143	211	0.9964

GO:0043231	IPI00685278;IPI00685418;IPI00686601;IPI00686803;IPI00686841;IPI00686966;IPI00687625;IPI00687842;IPI00688651;IPI00689005;IPI00689228;IPI00689325;IPI00689750;IPI00690001;IPI00690160;IPI00690232;IPI00690446;IPI00690785;IPI00691963;IPI00692468;IPI00692676;IPI00692819;IPI00692911;IPI00693755;IPI00694126;IPI00694214;IPI00694580;IPI00694849;IPI00694851;IPI00695176;IPI00695331;IPI00695600;IPI00695890;IPI00696616;IPI00696729;IPI00697081;IPI00697757;IPI00697891;IPI00698039;IPI00698900;IPI00699002;IP	Cellular Component	Intracellular membrane-bounded organelle	8	101	211	0.9968

GO:0043227	IPI00685278;IPI00685418;IPI00686601;IPI00686803;IPI00686841;IPI00686966;IPI00687625;IPI00687842;IPI00688651;IPI00689005;IPI00689228;IPI00689325;IPI00689750;IPI00690001;IPI00690160;IPI00690232;IPI00690446;IPI00690785;IPI00691963;IPI00692468;IPI00692676;IPI00692819;IPI00692911;IPI00693755;IPI00694126;IPI00694214;IPI00694580;IPI00694849;IPI00694851;IPI00695176;IPI00695331;IPI00695600;IPI00695890;IPI00696616;IPI00696729;IPI00697081;IPI00697757;IPI00697891;IPI00698039;IPI00698900;IPI00699002;IP	Cellular Component	Membrane-bounded organelle	4	101	211	0.9972
GO:0005739	IPI00686601;IPI00686803;IPI00686966;IPI00687842;IPI00692468;IPI00692819;IPI00694849;IPI00695890;IPI00701698;IPI00702650;IPI00703129;IPI00707559;IPI00708018;IPI00708438;IPI00711233;IPI00712677;IPI00715527;IPI00716881;IPI00718311;IPI00839134;IPI00944429	Cellular Component	Mitochondrion	9	21	211	0.9983

GO:0016020	IPI00685792;IPI00686966;IPI00687372;IPI00688651;IPI00688921;IPI00689035;IPI00689228;IPI00690001;IPI00690160;IPI00690785;IPI00691963;IPI00692468;IPI00693338;IPI00694204;IPI00694751;IPI00694849;IPI00695142;IPI00695506;IPI00695666;IPI00696616;IPI00696729;IPI00697081;IPI00698993;IPI00701698;IPI00702028;IPI00702620;IPI00702650;IPI00703129;IPI00703753;IPI00703776;IPI00704728;IPI00706458;IPI00707359;IPI00708018;IPI00708438;IPI00710408;IPI00711233;IPI00712677;IPI00713573;IPI00714481;IPI00714673;IP	Cellular Component	Membrane	3	53	211	0.9985

GO:0005622	IPI00685278;IPI00685418;IPI00686183;IPI00686225;IPI00686601;IPI00686803;IPI00686841;IPI00686966;IPI00687539;IPI00687601;IPI00687625;IPI00687657;IPI00687842;IPI00688489;IPI00688651;IPI00688921;IPI00689005;IPI00689228;IPI00689323;IPI00689325;IPI00689750;IPI00690001;IPI00690094;IPI00690160;IPI00690232;IPI00690446;IPI00690785;IPI00691963;IPI00692468;IPI00692588;IPI00692627;IPI00692676;IPI00692819;IPI00692911;IPI00693324;IPI00693755;IPI00694126;IPI00694204;IPI00694214;IPI00694312;IPI00694580;IP	Cellular Component	Intracellular	5	145	211	0.9985

GO:0044425	IPI00685792;IPI00686966;IPI00687372;IPI00688651;IPI00690160;IPI00691963;IPI00693338;IPI00695142;IPI00695506;IPI00696616;IPI00696729;IPI00698993;IPI00703129;IPI00703776;IPI00704728;IPI00707359;IPI00710408;IPI00711233;IPI00712677;IPI00714673;IPI00716195;IPI00716843;IPI00732368;IPI00838607;IPI00843257;IPI00904732	Cellular Component	Membrane part	4	26	211	0.9996
GO:0016021	IPI00686966;IPI00687372;IPI00691963;IPI00696616;IPI00703129;IPI00704728;IPI00707359;IPI00711233;IPI00712677;IPI00838607	Cellular Component	Integral component of membrane	6	10	211	1.0000
GO:0031224	IPI00686966;IPI00687372;IPI00691963;IPI00696616;IPI00703129;IPI00704728;IPI00707359;IPI00711233;IPI00712677;IPI00838607	Cellular Component	Intrinsic component of membrane	5	10	211	1.0000

GO:0005509	IPI00685792;IPI00689760;IPI00690785;IPI00691963;IPI00693628;IPI00693755;IPI00694751;IPI00695506;IPI00695508;IPI00696729;IPI00696930;IPI00703547;IPI00705660;IPI00706141;IPI00706458;IPI00707359;IPI00708234;IPI00709922;IPI00713141;IPI00715339;IPI00716493;IPI00717119;IPI00718698;IPI00843257;IPI00847093	Molecular Function	Calcium ion binding	7	25	211	0.0000
GO:0097367	IPI00687539;IPI00688608;IPI00689760;IPI00690094;IPI00693338;IPI00697081;IPI00701698;IPI00706624;IPI00715486;IPI00718698	Molecular Function	Carbohydrate derivative binding	4	10	211	0.0004
GO:0005539	IPI00687539;IPI00688608;IPI00689760;IPI00693338;IPI00697081;IPI00701698;IPI00706624;IPI00715486;IPI00718698	Molecular Function	Glycosaminoglycan binding	5	9	211	0.0007

GO:0061134	IPI00695489;IPI00701166;IPI00702700;IPI00703776;IPI00704735;IPI00706624;IPI00707101;IPI00709922;IPI00714673;IPI00715287;IPI00716121;IPI00871133	Molecular Function	Peptidase regulator activity	4	12	211	0.0028
GO:0050840	IPI00688608;IPI00697081;IPI00707359;IPI00840588	Molecular Function	Extracellular matrix binding	4	4	211	0.0081
GO:0004866	IPI00695489;IPI00701166;IPI00702700;IPI00703776;IPI00704735;IPI00707101;IPI00715287;IPI00716121;IPI00871133	Molecular Function	Endopeptidase inhibitor activity	6	9	211	0.0139
GO:0030414	IPI00695489;IPI00701166;IPI00702700;IPI00703776;IPI00704735;IPI00707101;IPI00715287;IPI00716121;IPI00871133	Molecular Function	Peptidase inhibitor activity	5	9	211	0.0153
GO:0061135	IPI00695489;IPI00701166;IPI00702700;IPI00703776;IPI00704735;IPI00707101;IPI00715287;IPI00716121;IPI00871133	Molecular Function	Endopeptidase regulator activity	5	9	211	0.0184
GO:0036041	IPI00686601;IPI00699355	Molecular Function	Long-chain fatty acid binding	6	2	211	0.0202
GO:0051879	IPI00703731;IPI00703854	Molecular Function	Hsp90 protein binding	6	2	211	0.0202
GO:0003714	IPI00690446;IPI00700542;IPI00701698;IPI01018577	Molecular Function	Transcription corepressor activity	6	4	211	0.0205

GO:0004867	IPI00695489;IPI00702700;IPI00704735;IPI00715287;IPI00716121;IPI00871133	Molecular Function	Serine-type endopeptidase inhibitor activity	7	6	211	0.0221
GO:0005319	IPI00686601;IPI00695965;IPI00697184	Molecular Function	Lipid transporter activity	5	3	211	0.0252
GO:0008307	IPI00694204;IPI00704977;IPI00707359	Molecular Function	Structural constituent of muscle	4	3	211	0.0252
GO:0003712	IPI00690446;IPI00694851;IPI00695331;IPI00700542;IPI00701698;IPI00842934;IPI01018577	Molecular Function	Transcription cofactor activity	5	7	211	0.0463
GO:0031072	IPI00703731;IPI00703854	Molecular Function	Heat shock protein binding	5	2	211	0.0467
GO:0000988	IPI00690446;IPI00694851;IPI00695331;IPI00700542;IPI00701698;IPI00842934;IPI01018577	Molecular Function	Protein binding transcription factor activity	3	7	211	0.0545
GO:0000989	IPI00690446;IPI00694851;IPI00695331;IPI00700542;IPI00701698;IPI00842934;IPI01018577	Molecular Function	Transcription factor binding transcription factor activity	4	7	211	0.0545
GO:1901681	IPI00687539;IPI00693338;IPI00706624;IPI00715486;IPI00718698	Molecular Function	Sulfur compound binding	4	5	211	0.0574
GO:0004857	IPI00695489;IPI00701166;IPI00702700;IPI00703776;IPI00704735;IPI00707101;IPI00715287;IPI00716121;IPI00871133	Molecular Function	Enzyme inhibitor activity	4	9	211	0.0580
GO:0016504	IPI00706624;IPI00709922;IPI00714673	Molecular Function	Peptidase activator activity	5	3	211	0.0582

GO:0043178	IPI00686601;IPI00695965;IPI00703547	Molecular Function	Alcohol binding	5	3	211	0.0582
GO:0043169	IPI00685792;IPI00686225;IPI00686841;IPI00687116;IPI00687842;IPI00689760;IPI00690785;IPI00691963;IPI00692468;IPI00692676;IPI00692819;IPI00693628;IPI00693755;IPI00694739;IPI00694751;IPI00694849;IPI00695506;IPI00695508;IPI00695600;IPI00695965;IPI00696729;IPI00696912;IPI00696930;IPI00699355;IPI00699798;IPI00700295;IPI00700542;IPI00703547;IPI00703753;IPI00705463;IPI00705660;IPI00706141;IPI00706203;IPI00706458;IPI00707359;IPI00708234;IPI00709922;IPI00713141;IPI00715339;IPI00716493;IPI00716555;IP	Molecular Function	Cation binding	5	51	211	0.0624
GO:0005504	IPI00686601;IPI00699355	Molecular Function	Fatty acid binding	5	2	211	0.0628
GO:0005540	IPI00689760;IPI00701698	Molecular Function	Hyaluronic acid binding	6	2	211	0.0628
GO:0005515	IPI00686225;IPI00687539;IPI00690160;IPI00692676;IPI00700547;IPI00701698;IPI00702620;IPI00703731;IPI00703854;IPI00706141;IPI00707359;IPI00716843;IPI00904732	Molecular Function	Protein binding	4	13	211	0.0661

GO:0008201	IPI00693338;IPI00706624;IPI00715486;IPI00718698	Molecular Function	Heparin binding	6	4	211	0.0688
GO:0016853	IPI00689323;IPI00689325;IPI00690785;IPI00698589;IPI00703731;IPI00706942;IPI00944429	Molecular Function	Isomerase activity	4	7	211	0.0736
GO:0005198	IPI00685418;IPI00689228;IPI00689750;IPI00692588;IPI00694204;IPI00694214;IPI00698900;IPI00704977;IPI00707359;IPI00708921;IPI00710408;IPI00713536;IPI00715091;IPI00721270;IPI00905865	Molecular Function	Structural molecule activity	3	15	211	0.0784

GO:0046872	IPI00685792;IPI00686225;IPI00686841;IPI00687116;IPI00687842;IPI00689760;IPI00690785;IPI00691963;IPI00692468;IPI00692676;IPI00692819;IPI00693628;IPI00693755;IPI00694739;IPI00694751;IPI00694849;IPI00695506;IPI00695508;IPI00695600;IPI00695965;IPI00696729;IPI00696930;IPI00699355;IPI00699798;IPI00700295;IPI00700542;IPI00703547;IPI00703753;IPI00705463;IPI00705660;IPI00706141;IPI00706458;IPI00707359;IPI00708234;IPI00709922;IPI00713141;IPI00715339;IPI00716493;IPI00716555;IPI00716843;IPI00717119;IP	Molecular Function	Metal ion binding	6	49	211	0.0784
GO:0051015	IPI00690160;IPI00706141	Molecular Function	Actin filament binding	7	2	211	0.0804
GO:0004298	IPI00695176;IPI00697757;IPI00707875	Molecular Function	Threonine-type endopeptidase activity	8	3	211	0.0915
GO:0004869	IPI00701166;IPI00703776;IPI00707101	Molecular Function	Cysteine-type endopeptidase inhibitor activity	7	3	211	0.0915
GO:0070003	IPI00695176;IPI00697757;IPI00707875	Molecular Function	Threonine-type peptidase activity	7	3	211	0.0915
GO:0001104	IPI00690446;IPI00694851	Molecular Function	RNA polymerase II transcription cofactor activity	6	2	211	0.0994
GO:0016868	IPI00689323;IPI00698589	Molecular Function	Intramolecular transferase activity, phosphotransferases	6	2	211	0.0994

GO:0042578	IPI00686841;IPI00690308;IPI00692819;IPI00693338;IPI00698589;IPI00699798;IPI00700789;IPI00842934	Molecular Function	Phosphoric ester hydrolase activity	6	8	211	0.1033
GO:0016860	IPI00689325;IPI00706942;IPI00944429	Molecular Function	Intramolecular oxidoreductase activity	5	3	211	0.1040
GO:0016791	IPI00690308;IPI00692819;IPI00693338;IPI00698589;IPI00699798;IPI00700789;IPI00842934	Molecular Function	Phosphatase activity	7	7	211	0.1152
GO:0001846	IPI00701698	Molecular Function	Opsonin binding	5	1	211	0.1176
GO:0001848	IPI00701698	Molecular Function	Complement binding	5	1	211	0.1176
GO:0001849	IPI00701698	Molecular Function	Complement component C1q binding	6	1	211	0.1176
GO:0003972	IPI00700295	Molecular Function	RNA ligase (ATP) activity	7	1	211	0.1176
GO:0003979	IPI00712671	Molecular Function	UDP-glucose 6-dehydrogenase activity	7	1	211	0.1176
GO:0004001	IPI00726650	Molecular Function	Adenosine kinase activity	9	1	211	0.1176
GO:0004032	IPI00700920	Molecular Function	Alditol:NADP+ 1-oxidoreductase activity	9	1	211	0.1176
GO:0004082	IPI00698589	Molecular Function	Bisphosphoglycerate mutase activity	7	1	211	0.1176
GO:0004083	IPI00698589	Molecular Function	Bisphosphoglycerate 2-phosphatase activity	9	1	211	0.1176
GO:0004146	IPI00742596	Molecular Function	Dihydrofolate reductase activity	7	1	211	0.1176
GO:0004422	IPI00686225	Molecular Function	Hypoxanthine phosphoribosyltransferase activity	7	1	211	0.1176
GO:0004452	IPI00944429	Molecular Function	Isopentenyl-diphosphate delta-isomerase activity	7	1	211	0.1176
GO:0004615	IPI00689323	Molecular Function	Phosphomannomutase activity	7	1	211	0.1176
GO:0004619	IPI00698589	Molecular Function	Phosphoglycerate mutase activity	7	1	211	0.1176
GO:0004802	IPI00904104	Molecular Function	Transketolase activity	6	1	211	0.1176
GO:0004807	IPI00706942	Molecular Function	Triose-phosphate isomerase activity	7	1	211	0.1176
GO:0005246	IPI00700789	Molecular Function	Calcium channel regulator activity	4	1	211	0.1176
GO:0005548	IPI00686601	Molecular Function	Phospholipid transporter activity	6	1	211	0.1176
GO:0008175	IPI00694126	Molecular Function	tRNA methyltransferase activity	8	1	211	0.1176
GO:0008195	IPI00842934	Molecular Function	Phosphatidate phosphatase activity	8	1	211	0.1176
GO:0008452	IPI00700295	Molecular Function	RNA ligase activity	6	1	211	0.1176

GO:0008526	IPI00686601	Molecular Function	Phosphatidylinositol transporter activity	7	1	211	0.1176
GO:0008892	IPI00716555	Molecular Function	Guanine deaminase activity	7	1	211	0.1176
GO:0016427	IPI00694126	Molecular Function	tRNA (cytosine) methyltransferase activity	9	1	211	0.1176
GO:0016428	IPI00694126	Molecular Function	tRNA (cytosine-5-)-methyltransferase activity	10	1	211	0.1176
GO:0016768	IPI00711759	Molecular Function	Spermine synthase activity	6	1	211	0.1176
GO:0016936	IPI00690094	Molecular Function	Galactoside binding	5	1	211	0.1176
GO:0017018	IPI00699798	Molecular Function	Myosin phosphatase activity	10	1	211	0.1176
GO:0017061	IPI00883375	Molecular Function	S-methyl-5-thioadenosine phosphorylase activity	7	1	211	0.1176
GO:0017124	IPI00687539	Molecular Function	SH3 domain binding	6	1	211	0.1176
GO:0019855	IPI00700789	Molecular Function	Calcium channel inhibitor activity	5	1	211	0.1176
GO:0030331	IPI00703731	Molecular Function	Estrogen receptor binding	9	1	211	0.1176
GO:0030984	IPI00701698	Molecular Function	Kininogen binding	5	1	211	0.1176
GO:0031690	IPI00701698	Molecular Function	Adrenergic receptor binding	7	1	211	0.1176
GO:0033814	IPI00686601	Molecular Function	Propanoyl-CoA C-acyltransferase activity	8	1	211	0.1176
GO:0034416	IPI00698589	Molecular Function	Bisphosphoglycerate phosphatase activity	8	1	211	0.1176
GO:0034632	IPI00697184	Molecular Function	Retinol transporter activity	6	1	211	0.1176
GO:0035374	IPI00687539	Molecular Function	Chondroitin sulfate binding	6	1	211	0.1176
GO:0036042	IPI00686601	Molecular Function	Long-chain fatty acyl-CoA binding	7	1	211	0.1176
GO:0042165	IPI00839134	Molecular Function	Neurotransmitter binding	4	1	211	0.1176
GO:0046570	IPI00694739	Molecular Function	Methylthioribulose 1-phosphate dehydratase activity	7	1	211	0.1176
GO:0047006	IPI00700920	Molecular Function	17-alpha,20-alpha-dihydroxypregn-4-en-3-one dehydrogenase activity	7	1	211	0.1176
GO:0047429	IPI00703753	Molecular Function	Nucleoside-triphosphate diphosphatase activity	8	1	211	0.1176
GO:0050115	IPI00699798	Molecular Function	Myosin-light-chain-phosphatase activity	8	1	211	0.1176
GO:0051010	IPI00904732	Molecular Function	Microtubule plus-end binding	8	1	211	0.1176
GO:0052657	IPI00686225	Molecular Function	Guanine phosphoribosyltransferase activity	7	1	211	0.1176
GO:0052832	IPI00692819	Molecular Function	Inositol monophosphate 3-phosphatase activity	10	1	211	0.1176
GO:0052833	IPI00692819	Molecular Function	Inositol monophosphate 4-phosphatase activity	10	1	211	0.1176
GO:0070538	IPI00686601	Molecular Function	Oleic acid binding	7	1	211	0.1176
GO:0070539	IPI00699355	Molecular Function	Linoleic acid binding	7	1	211	0.1176

GO:0001076	IPI00690446;IPI00694851	Molecular Function	RNA polymerase II transcription factor binding transcription factor activity	5	2	211	0.1195
GO:0003779	IPI00690160;IPI00706141	Molecular Function	Actin binding	6	2	211	0.1195
GO:0008134	IPI00692676;IPI00703731	Molecular Function	Transcription factor binding	5	2	211	0.1195
GO:0016405	IPI00696912;IPI00708438	Molecular Function	CoA-ligase activity	6	2	211	0.1195
GO:0033293	IPI00686601;IPI00699355	Molecular Function	Monocarboxylic acid binding	7	2	211	0.1195
GO:0030246	IPI00689760;IPI00690094;IPI00691963;IPI00700547;IPI00703753	Molecular Function	Carbohydrate binding	4	5	211	0.1339
GO:0008092	IPI00690160;IPI00706141;IPI00904732	Molecular Function	Cytoskeletal protein binding	5	3	211	0.1452
GO:0001664	IPI00701698;IPI00716843	Molecular Function	G-protein coupled receptor binding	6	2	211	0.1620
GO:0005200	IPI00689228;IPI00698900	Molecular Function	Structural constituent of cytoskeleton	4	2	211	0.1620
GO:0016877	IPI00696912;IPI00708438	Molecular Function	Ligase activity, forming carbon-sulfur bonds	5	2	211	0.1620
GO:0016878	IPI00696912;IPI00708438	Molecular Function	Acid-thiol ligase activity	6	2	211	0.1620
GO:0001758	IPI00692627	Molecular Function	Retinal dehydrogenase activity	7	1	211	0.1711
GO:0003858	IPI00694312	Molecular Function	3-hydroxybutyrate dehydrogenase activity	7	1	211	0.1711
GO:0003878	IPI00708438	Molecular Function	ATP citrate synthase activity	7	1	211	0.1711
GO:0004020	IPI00730144	Molecular Function	Adenylylsulfate kinase activity	7	1	211	0.1711
GO:0004045	IPI00690308	Molecular Function	Aminoacyl-tRNA hydrolase activity	7	1	211	0.1711
GO:0004300	IPI00702650	Molecular Function	Enoyl-CoA hydratase activity	7	1	211	0.1711
GO:0004322	IPI00687842	Molecular Function	Ferroxidase activity	7	1	211	0.1711
GO:0004563	IPI00706203	Molecular Function	Beta-N-acetylhexosaminidase activity	8	1	211	0.1711
GO:0004776	IPI00708438	Molecular Function	Succinate-CoA ligase (GDP-forming) activity	8	1	211	0.1711
GO:0004779	IPI00730144	Molecular Function	Sulfate adenylyltransferase activity	8	1	211	0.1711
GO:0004781	IPI00730144	Molecular Function	Sulfate adenylyltransferase (ATP) activity	9	1	211	0.1711
GO:0005006	IPI00696930	Molecular Function	Epidermal growth factor-activated receptor activity	10	1	211	0.1711

GO:0005499	IPI00703547	Molecular Function	Vitamin D binding	6	1	211	0.1711
GO:0008934	IPI00692819	Molecular Function	Inositol monophosphate 1-phosphatase activity	10	1	211	0.1711
GO:0008969	IPI00700789	Molecular Function	Phosphohistidine phosphatase activity	8	1	211	0.1711
GO:0015288	IPI00703129	Molecular Function	Porin activity	8	1	211	0.1711
GO:0016229	IPI00700920	Molecular Function	Steroid dehydrogenase activity	5	1	211	0.1711
GO:0016509	IPI00702650	Molecular Function	Long-chain-3-hydroxyacyl-CoA dehydrogenase activity	8	1	211	0.1711
GO:0016724	IPI00687842	Molecular Function	Oxidoreductase activity, oxidizing metal ions, oxygen as acceptor	6	1	211	0.1711
GO:0016744	IPI00904104	Molecular Function	Transferase activity, transferring aldehyde or ketonic groups	5	1	211	0.1711
GO:0016863	IPI00944429	Molecular Function	Intramolecular oxidoreductase activity, transposing C=C bonds	6	1	211	0.1711
GO:0016918	IPI00697184	Molecular Function	Retinal binding	7	1	211	0.1711
GO:0022829	IPI00703129	Molecular Function	Wide pore channel activity	7	1	211	0.1711
GO:0033764	IPI00700920	Molecular Function	Steroid dehydrogenase activity, acting on the CH-OH group of donors, NAD or NADP as acceptor	6	1	211	0.1711
GO:0035258	IPI00703731	Molecular Function	Steroid hormone receptor binding	8	1	211	0.1711
GO:0043236	IPI00707359	Molecular Function	Laminin binding	5	1	211	0.1711
GO:0051183	IPI00697184	Molecular Function	Vitamin transporter activity	4	1	211	0.1711
GO:0052834	IPI00692819	Molecular Function	Inositol monophosphate phosphatase activity	9	1	211	0.1711
GO:0097177	IPI00701698	Molecular Function	Mitochondrial ribosome binding	6	1	211	0.1711
GO:0004721	IPI00690308;IPI00693338;IPI00699798;IPI00700789	Molecular Function	Phosphoprotein phosphatase activity	8	4	211	0.1712
GO:0016209	IPI00686183;IPI00692468;IPI00694751;IPI00695965	Molecular Function	Antioxidant activity	3	4	211	0.1712
GO:0008144	IPI00690785;IPI00699355;IPI00742596	Molecular Function	Drug binding	4	3	211	0.1751
GO:0003713	IPI00694851;IPI00695331;IPI00842934;IPI01018577	Molecular Function	Transcription coactivator activity	6	4	211	0.1960
GO:0003697	IPI00695331;IPI00695890	Molecular Function	Single-stranded DNA binding	8	2	211	0.2068
GO:0003746	IPI00704728;IPI00712775	Molecular Function	Translation elongation factor activity	8	2	211	0.2068

GO:0016866	IPI00689323;IPI00698589	Molecular Function	Intramolecular transferase activity	5	2	211	0.2068
GO:0001106	IPI00690446	Molecular Function	RNA polymerase II transcription corepressor activity	7	1	211	0.2214
GO:0001191	IPI00690446	Molecular Function	RNA polymerase II transcription factor binding transcription factor activity involved in negative regulation of transcription	6	1	211	0.2214
GO:0003707	IPI00699355	Molecular Function	Steroid hormone receptor activity	5	1	211	0.2214
GO:0003987	IPI00696912	Molecular Function	acetate-CoA ligase activity	7	1	211	0.2214
GO:0004033	IPI00700920	Molecular Function	Aldo-keto reductase (NADP) activity	7	1	211	0.2214
GO:0004181	IPI00700542	Molecular Function	Metallocarboxypeptidase activity	9	1	211	0.2214
GO:0004645	IPI00883375	Molecular Function	Phosphorylase activity	7	1	211	0.2214
GO:0004775	IPI00708438	Molecular Function	Succinate-CoA ligase (ADP-forming) activity	8	1	211	0.2214
GO:0004879	IPI00699355	Molecular Function	Ligand-activated sequence-specific DNA binding RNA polymerase II transcription factor activity	6	1	211	0.2214
GO:0005212	IPI00689228	Molecular Function	Structural constituent of eye lens	4	1	211	0.2214
GO:0008106	IPI00700920	Molecular Function	Alcohol dehydrogenase (NADP+) activity	8	1	211	0.2214
GO:0008173	IPI00694126	Molecular Function	RNA methyltransferase activity	7	1	211	0.2214
GO:0008200	IPI00700789	Molecular Function	Ion channel inhibitor activity	5	1	211	0.2214
GO:0015929	IPI00706203	Molecular Function	Hexosaminidase activity	7	1	211	0.2214
GO:0016248	IPI00700789	Molecular Function	Channel inhibitor activity	4	1	211	0.2214
GO:0016722	IPI00687842	Molecular Function	Oxidoreductase activity, oxidizing metal ions	5	1	211	0.2214
GO:0016812	IPI00687539	Molecular Function	Hydrolase activity, acting on carbon-nitrogen (but not peptide) bonds, in cyclic amides	6	1	211	0.2214
GO:0016886	IPI00700295	Molecular Function	Ligase activity, forming phosphoric ester bonds	5	1	211	0.2214
GO:0019206	IPI00726650	Molecular Function	Nucleoside kinase activity	8	1	211	0.2214
GO:0019239	IPI00716555	Molecular Function	Deaminase activity	4	1	211	0.2214
GO:0019841	IPI00697184	Molecular Function	Retinol binding	7	1	211	0.2214
GO:0030544	IPI00703731	Molecular Function	Hsp70 protein binding	6	1	211	0.2214
GO:0031210	IPI00695965	Molecular Function	Phosphatidylcholine binding	6	1	211	0.2214
GO:0031821	IPI00716843	Molecular Function	G-protein coupled serotonin receptor binding	7	1	211	0.2214
GO:0035257	IPI00703731	Molecular Function	Nuclear hormone receptor binding	7	1	211	0.2214
GO:0050997	IPI00695965	Molecular Function	Quaternary ammonium group binding	4	1	211	0.2214

GO:0005496	IPI00686601;IPI00703547	Molecular Function	Steroid binding	5	2	211	0.2297
GO:0043022	IPI00701698;IPI00704728	Molecular Function	Ribosome binding	5	2	211	0.2297
GO:0003729	IPI00691963;IPI00701698;IPI00717759;IPI00742596	Molecular Function	mRNA binding	8	4	211	0.2351
GO:0004725	IPI00690308;IPI00693338	Molecular Function	Protein tyrosine phosphatase activity	9	2	211	0.2527
GO:0016763	IPI00686225;IPI00883375	Molecular Function	Transferase activity, transferring pentosyl groups	6	2	211	0.2527
GO:0030145	IPI00692468;IPI00944429	Molecular Function	Manganese ion binding	8	2	211	0.2527
GO:0051287	IPI00690446;IPI00694312;IPI00712671	Molecular Function	NAD binding	6	3	211	0.2558
GO:0016616	IPI00690446;IPI00694312;IPI00700920;IPI00702650;IPI00712671	Molecular Function	Oxidoreductase activity, acting on the CH-OH group of donors, NAD or NADP as acceptor	6	5	211	0.2620
GO:0004872	IPI00694851;IPI00696930;IPI00699355;IPI00703753	Molecular Function	Receptor activity	3	4	211	0.2621
GO:0001530	IPI00697757	Molecular Function	Lipopolysaccharide binding	5	1	211	0.2687
GO:0001948	IPI00700547	Molecular Function	Glycoprotein binding	5	1	211	0.2687
GO:0004551	IPI00703753	Molecular Function	Nucleotide diphosphatase activity	8	1	211	0.2687
GO:0004683	IPI00688651	Molecular Function	Calmodulin-dependent protein kinase activity	9	1	211	0.2687
GO:0004774	IPI00708438	Molecular Function	Succinate-CoA ligase activity	7	1	211	0.2687
GO:0004784	IPI00692468	Molecular Function	Superoxide dismutase activity	6	1	211	0.2687
GO:0005501	IPI00697184	Molecular Function	Retinoid binding	6	1	211	0.2687
GO:0005546	IPI00706141	Molecular Function	Phosphatidylinositol-4,5-bisphosphate binding	9	1	211	0.2687
GO:0008553	IPI00716195	Molecular Function	Hydrogen-exporting ATPase activity, phosphorylative mechanism	15	1	211	0.2687
GO:0016208	IPI00696912	Molecular Function	AMP binding	9	1	211	0.2687
GO:0016247	IPI00700789	Molecular Function	Channel regulator activity	3	1	211	0.2687
GO:0016721	IPI00692468	Molecular Function	Oxidoreductase activity, acting on superoxide radicals as acceptor	5	1	211	0.2687
GO:0019840	IPI00697184	Molecular Function	Isoprenoid binding	5	1	211	0.2687
GO:0031625	IPI00702620	Molecular Function	Ubiquitin protein ligase binding	7	1	211	0.2687

GO:0044389	IPI00702620	Molecular Function	Small conjugating protein ligase binding	6	1	211	0.2687
GO:0046912	IPI00708438	Molecular Function	Transferase activity, transferring acyl groups, acyl groups converted into alkyl on transfer	6	1	211	0.2687
GO:0051427	IPI00703731	Molecular Function	Hormone receptor binding	6	1	211	0.2687
GO:0060228	IPI00695965	Molecular Function	Phosphatidylcholine-sterol O-acyltransferase activator activity	5	1	211	0.2687
GO:0070566	IPI00730144	Molecular Function	Adenylyltransferase activity	7	1	211	0.2687
GO:0005102	IPI00701698;IPI00703731;IPI00716843	Molecular Function	Receptor binding	5	3	211	0.2725
GO:0031406	IPI00686601;IPI00699355;IPI00706141	Molecular Function	Carboxylic acid binding	6	3	211	0.2725
GO:0016836	IPI00694739;IPI00702650	Molecular Function	Hydro-lyase activity	6	2	211	0.2758
GO:0003954	IPI00711233;IPI00712677	Molecular Function	NADH dehydrogenase activity	6	2	211	0.2988
GO:0008137	IPI00711233;IPI00712677	Molecular Function	NADH dehydrogenase (ubiquinone) activity	8	2	211	0.2988
GO:0015035	IPI00689325;IPI00694580	Molecular Function	Protein disulfide oxidoreductase activity	7	2	211	0.2988
GO:0050136	IPI00711233;IPI00712677	Molecular Function	NADH dehydrogenase (quinone) activity	7	2	211	0.2988
GO:0000062	IPI00686601	Molecular Function	Fatty-acyl-CoA binding	6	1	211	0.3131
GO:0001786	IPI00706141	Molecular Function	Phosphatidylserine binding	6	1	211	0.3131
GO:0001871	IPI00703753	Molecular Function	Pattern binding	4	1	211	0.3131
GO:0003756	IPI00689325	Molecular Function	Protein disulfide isomerase activity	7	1	211	0.3131
GO:0003823	IPI00695142	Molecular Function	Antigen binding	4	1	211	0.3131
GO:0004180	IPI00700542	Molecular Function	Carboxypeptidase activity	8	1	211	0.3131
GO:0004714	IPI00696930	Molecular Function	Transmembrane receptor protein tyrosine kinase activity	9	1	211	0.3131
GO:0008199	IPI00687842	Molecular Function	Ferric iron binding	9	1	211	0.3131
GO:0015248	IPI00695965	Molecular Function	Sterol transporter activity	6	1	211	0.3131
GO:0016862	IPI00689325	Molecular Function	Intramolecular oxidoreductase activity, interconverting keto- and enol-groups	6	1	211	0.3131
GO:0016864	IPI00689325	Molecular Function	Intramolecular oxidoreductase activity, transposing S-S bonds	6	1	211	0.3131
GO:0017091	IPI00687625	Molecular Function	AU-rich element binding	7	1	211	0.3131
GO:0017127	IPI00695965	Molecular Function	Cholesterol transporter activity	7	1	211	0.3131

GO:0030247	IPI00703753	Molecular Function	Polysaccharide binding	5	1	211	0.3131
GO:0043027	IPI00703776	Molecular Function	Cysteine-type endopeptidase inhibitor activity involved in apoptotic process	8	1	211	0.3131
GO:0052745	IPI00692819	Molecular Function	Inositol phosphate phosphatase activity	8	1	211	0.3131
GO:0016788	IPI00686803;IPI00686841;IPI00690308;IPI00692819;IPI00693338;IPI00698589;IPI00699798;IPI00700789;IPI00708018;IPI00711419;IPI00842934	Molecular Function	Hydrolase activity, acting on ester bonds	5	11	211	0.3145
GO:0019842	IPI00697184;IPI00703547	Molecular Function	Vitamin binding	5	2	211	0.3217
GO:0016614	IPI00690446;IPI00694312;IPI00700920;IPI00702650;IPI00712671	Molecular Function	Oxidoreductase activity, acting on CH-OH group of donors	5	5	211	0.3221
GO:0050662	IPI00686601;IPI00690446;IPI00694312;IPI00702650;IPI00707559;IPI00712671;IPI00742596	Molecular Function	Coenzyme binding	5	7	211	0.3230
GO:0003755	IPI00690785;IPI00703731	Molecular Function	Peptidyl-prolyl cis-trans isomerase activity	6	2	211	0.3444
GO:0015036	IPI00689325;IPI00694580	Molecular Function	Disulfide oxidoreductase activity	6	2	211	0.3444
GO:0016835	IPI00694739;IPI00702650	Molecular Function	Carbon-oxygen lyase activity	5	2	211	0.3444
GO:0003857	IPI00702650	Molecular Function	3-hydroxyacyl-CoA dehydrogenase activity	7	1	211	0.3548
GO:0004029	IPI00692627	Molecular Function	Aldehyde dehydrogenase (NAD) activity	7	1	211	0.3548
GO:0004602	IPI00686183	Molecular Function	Glutathione peroxidase activity	7	1	211	0.3548
GO:0008017	IPI00904732	Molecular Function	Microtubule binding	7	1	211	0.3548
GO:0008408	IPI00708018	Molecular Function	3'-5' exonuclease activity	8	1	211	0.3548
GO:0015485	IPI00686601	Molecular Function	Cholesterol binding	7	1	211	0.3548
GO:0016814	IPI00716555	Molecular Function	Hydrolase activity, acting on carbon-nitrogen (but not peptide) bonds, in cyclic amidines	6	1	211	0.3548
GO:0019199	IPI00696930	Molecular Function	Transmembrane receptor protein kinase activity	8	1	211	0.3548

GO:0031683	IPI00716843	Molecular Function	G-protein beta/gamma-subunit complex binding	6	1	211	0.3548
GO:0016859	IPI00690785;IPI00703731	Molecular Function	cis-trans isomerase activity	5	2	211	0.3669
GO:0008308	IPI00703129	Molecular Function	Voltage-gated anion channel activity	10	1	211	0.3940
GO:0016861	IPI00706942	Molecular Function	Intramolecular oxidoreductase activity, interconverting aldoses and ketoses	6	1	211	0.3940
GO:0016655	IPI00711233;IPI00712677	Molecular Function	Oxidoreductase activity, acting on NAD(P)H, quinone or similar compound as acceptor	6	2	211	0.4109
GO:0009055	IPI00689325;IPI00694580;IPI00707559	Molecular Function	Electron carrier activity	3	3	211	0.4245
GO:0005044	IPI00703753	Molecular Function	Scavenger receptor activity	5	1	211	0.4308
GO:0005244	IPI00703129	Molecular Function	Voltage-gated ion channel activity	9	1	211	0.4308
GO:0005527	IPI00690785	Molecular Function	Macrolide binding	5	1	211	0.4308
GO:0005528	IPI00690785	Molecular Function	FK506 binding	6	1	211	0.4308
GO:0016408	IPI00686601	Molecular Function	C-acyltransferase activity	7	1	211	0.4308
GO:0019904	IPI00687539	Molecular Function	Protein domain specific binding	5	1	211	0.4308
GO:0022832	IPI00703129	Molecular Function	Voltage-gated channel activity	8	1	211	0.4308
GO:0032934	IPI00686601	Molecular Function	Sterol binding	6	1	211	0.4308
GO:0043028	IPI00703776	Molecular Function	Cysteine-type endopeptidase regulator activity involved in apoptotic process	4	1	211	0.4308
GO:0043566	IPI00695331;IPI00695890	Molecular Function	Structure-specific DNA binding	7	2	211	0.4324
GO:0005525	IPI00699803;IPI00703380;IPI00705941;IPI00707718;IPI00708438;IPI00712775;IPI00714302;IPI00716843	Molecular Function	GTP binding	9	8	211	0.4630
GO:0005253	IPI00703129	Molecular Function	Anion channel activity	9	1	211	0.4654
GO:0008235	IPI00700542	Molecular Function	Metalloexopeptidase activity	8	1	211	0.4654
GO:0015631	IPI00904732	Molecular Function	Tubulin binding	6	1	211	0.4654
GO:0043021	IPI00701698;IPI00704728	Molecular Function	Ribonucleoprotein complex binding	4	2	211	0.4739

GO:0019001	IPI00699803;IPI00703380;IPI00705941;IPI00707718;IPI00708438;IPI00712775;IPI00714302;IPI00716843	Molecular Function	Guanyl nucleotide binding	7	8	211	0.4817
GO:0032561	IPI00699803;IPI00703380;IPI00705941;IPI00707718;IPI00708438;IPI00712775;IPI00714302;IPI00716843	Molecular Function	Guanyl ribonucleotide binding	8	8	211	0.4817
GO:0048037	IPI00686601;IPI00690446;IPI00694312;IPI00702650;IPI00707559;IPI00708438;IPI00712671;IPI00742596	Molecular Function	Cofactor binding	4	8	211	0.4910
GO:0038023	IPI00696930;IPI00699355	Molecular Function	Signaling receptor activity	4	2	211	0.4940
GO:0003727	IPI00685278	Molecular Function	Single-stranded RNA binding	7	1	211	0.4979
GO:0004129	IPI00694849	Molecular Function	Cytochrome-c oxidase activity	6	1	211	0.4979
GO:0008081	IPI00686841	Molecular Function	Phosphoric diester hydrolase activity	7	1	211	0.4979
GO:0015002	IPI00694849	Molecular Function	Heme-copper terminal oxidase activity	5	1	211	0.4979
GO:0016675	IPI00694849	Molecular Function	Oxidoreductase activity, acting on a heme group of donors	5	1	211	0.4979
GO:0016676	IPI00694849	Molecular Function	Oxidoreductase activity, acting on a heme group of donors, oxygen as acceptor	6	1	211	0.4979
GO:0038024	IPI00703753	Molecular Function	Cargo receptor activity	4	1	211	0.4979
GO:1901981	IPI00706141	Molecular Function	Phosphatidylinositol phosphate binding	7	1	211	0.4979

GO:0005488	IPI00685278;IPI00685792;IPI00686225;IPI00686601;IPI00686841;IPI00687116;IPI00687539;IPI00687625;IPI00687842;IPI00688489;IPI00688608;IPI00688651;IPI00689760;IPI00690094;IPI00690160;IPI00690232;IPI00690446;IPI00690785;IPI00691963;IPI00692468;IPI00692676;IPI00692819;IPI00693324;IPI00693338;IPI00693628;IPI00693755;IPI00694312;IPI00694739;IPI00694751;IPI00694849;IPI00695142;IPI00695331;IPI00695506;IPI00695508;IPI00695600;IPI00695890;IPI00695965;IPI00696729;IPI00696912;IPI00696930;IPI00697070;IP	Molecular Function	Binding	3	110	211	0.5142
GO:0030234	IPI00695489;IPI00695965;IPI00701166;IPI00702700;IPI00703776;IPI00704735;IPI00706624;IPI00707101;IPI00709922;IPI00714673;IPI00715287;IPI00716121;IPI00871133	Molecular Function	Enzyme regulator activity	3	13	211	0.5194
GO:0015662	IPI00716195	Molecular Function	ATPase activity, coupled to transmembrane movement of ions, phosphorylative mechanism	14	1	211	0.5285
GO:0016646	IPI00742596	Molecular Function	Oxidoreductase activity, acting on the CH-NH group of donors, NAD or NADP as acceptor	6	1	211	0.5285
GO:0072341	IPI00706141	Molecular Function	Modified amino acid binding	4	1	211	0.5285
GO:0004518	IPI00686803;IPI00708018	Molecular Function	Nuclease activity	6	2	211	0.5328

GO:0016667	IPI00689325;IPI00694580	Molecular Function	Oxidoreductase activity, acting on a sulfur group of donors	5	2	211	0.5513
GO:0004527	IPI00708018	Molecular Function	Exonuclease activity	7	1	211	0.5572
GO:0004722	IPI00699798	Molecular Function	Protein serine/threonine phosphatase activity	9	1	211	0.5572
GO:0016628	IPI00694312	Molecular Function	Oxidoreductase activity, acting on the CH-CH group of donors, NAD or NADP as acceptor	6	1	211	0.5572
GO:0019205	IPI00726650	Molecular Function	Nucleobase-containing compound kinase activity	7	1	211	0.5572
GO:0008289	IPI00686601;IPI00695965;IPI00697184;IPI00697757;IPI00699355;IPI00703547;IPI00704735;IPI00706141;IPI00718381;IPI00868597	Molecular Function	Lipid binding	4	10	211	0.5575
GO:0008047	IPI00695965;IPI00703776;IPI00706624;IPI00709922;IPI00714673	Molecular Function	Enzyme activator activity	4	5	211	0.5619
GO:0005507	IPI00695965	Molecular Function	Copper ion binding	8	1	211	0.5841
GO:0016597	IPI00706141	Molecular Function	Amino acid binding	7	1	211	0.5841
GO:0016491	IPI00686183;IPI00687842;IPI00689325;IPI00690446;IPI00692468;IPI00692627;IPI00694312;IPI00694580;IPI00694849;IPI00700920;IPI00702650;IPI00707559;IPI00711233; IPI00712671;IPI00712677;IPI00742596	Molecular Function	Oxidoreductase activity	4	16	211	0.6038
GO:0001071	IPI00690446;IPI00699355;IPI01018577	Molecular Function	Nucleic acid binding transcription factor activity	3	3	211	0.6063
GO:0003700	IPI00690446;IPI00699355;IPI01018577	Molecular Function	Sequence-specific DNA binding transcription factor activity	4	3	211	0.6063
GO:0000981	IPI00699355	Molecular Function	Sequence-specific DNA binding RNA polymerase II transcription factor activity	5	1	211	0.6094

GO:0016645	IPI00742596	Molecular Function	Oxidoreductase activity, acting on the CH-NH group of donors	5	1	211	0.6094
GO:0022836	IPI00703129	Molecular Function	Gated channel activity	7	1	211	0.6094
GO:0022839	IPI00703129	Molecular Function	Ion gated channel activity	8	1	211	0.6094
GO:0003723	IPI00685278;IPI00686841;IPI00687625;IPI00690232;IPI00691963;IPI00693324;IPI00695600;IPI00697891;IPI00701698;IPI00704728;IPI00705463;IPI00712775;IPI00715091;IPI00717759;IPI00742596	Molecular Function	RNA binding	6	15	211	0.6159
GO:0016810	IPI00687539;IPI00716555	Molecular Function	Hydrolase activity, acting on carbon-nitrogen (but not peptide) bonds	5	2	211	0.6204
GO:0008135	IPI00693324;IPI00704728;IPI00712775	Molecular Function	Translation factor activity, nucleic acid binding	7	3	211	0.6327
GO:0004601	IPI00686183	Molecular Function	Peroxidase activity	6	1	211	0.6332
GO:0016684	IPI00686183	Molecular Function	Oxidoreductase activity, acting on peroxide as acceptor	5	1	211	0.6332
GO:0016651	IPI00711233;IPI00712677	Molecular Function	Oxidoreductase activity, acting on NAD(P)H	5	2	211	0.6518
GO:0004519	IPI00686803	Molecular Function	Endonuclease activity	7	1	211	0.6556
GO:0004713	IPI00696930	Molecular Function	Protein tyrosine kinase activity	8	1	211	0.6556
GO:0004888	IPI00696930	Molecular Function	Transmembrane signaling receptor activity	5	1	211	0.6556
GO:0008757	IPI00694126	Molecular Function	S-adenosylmethionine-dependent methyltransferase activity	7	1	211	0.6556
GO:0032403	IPI00716843	Molecular Function	Protein complex binding	5	1	211	0.6556
GO:0015078	IPI00694849;IPI00716195	Molecular Function	Hydrogen ion transmembrane transporter activity	10	2	211	0.6667
GO:0004871	IPI00690094;IPI00696930;IPI00699355;IPI00716843	Molecular Function	Signal transducer activity	4	4	211	0.6764
GO:0060089	IPI00690094;IPI00696930;IPI00699355;IPI00716843	Molecular Function	Molecular transducer activity	3	4	211	0.6764

GO:0016620	IPI00692627	Molecular Function	Oxidoreductase activity, acting on the aldehyde or oxo group of donors, NAD or NADP as acceptor	6	1	211	0.6766
GO:0019829	IPI00716195	Molecular Function	Cation-transporting ATPase activity	14	1	211	0.6766
GO:0005216	IPI00703129	Molecular Function	Ion channel activity	8	1	211	0.6963
GO:0022838	IPI00703129	Molecular Function	Substrate-specific channel activity	7	1	211	0.6963
GO:0015267	IPI00703129	Molecular Function	Channel activity	6	1	211	0.7148
GO:0016765	IPI00711759	Molecular Function	Transferase activity, transferring alkyl or aryl (other than methyl) groups	5	1	211	0.7148
GO:0022803	IPI00703129	Molecular Function	Passive transmembrane transporter activity	5	1	211	0.7148
GO:0042277	IPI00703731	Molecular Function	Peptide binding	5	1	211	0.7148
GO:0042625	IPI00716195	Molecular Function	ATPase activity, coupled to transmembrane movement of ions	13	1	211	0.7148
GO:0004842	IPI00700547;IPI01018577	Molecular Function	Ubiquitin-protein transferase activity	6	2	211	0.7212
GO:0033218	IPI00703731	Molecular Function	Amide binding	4	1	211	0.7322
GO:0050661	IPI00742596	Molecular Function	NADP binding	6	1	211	0.7322
GO:0052689	IPI00690308	Molecular Function	Carboxylic ester hydrolase activity	6	1	211	0.7322
GO:0043565	IPI00699355;IPI01018577	Molecular Function	Sequence-specific DNA binding	7	2	211	0.7455
GO:0004553	IPI00706203	Molecular Function	Hydrolase activity, hydrolyzing O-glycosyl compounds	6	1	211	0.7485
GO:0003924	IPI00705941;IPI00712775;IPI00716843	Molecular Function	GTPase activity	9	3	211	0.7556
GO:0015077	IPI00694849;IPI00716195	Molecular Function	Monovalent inorganic cation transmembrane transporter activity	9	2	211	0.7570
GO:0019787	IPI00700547;IPI01018577	Molecular Function	Small conjugating protein transferase activity	5	2	211	0.7570

GO:0043167	IPI00685792;IPI00686225;IPI00686601;IPI00686841;IPI00687116;IPI00687539;IPI00687842;IPI00688489;IPI00688651;IPI00689760;IPI00690785;IPI00691963;IPI00692468;IPI00692676;IPI00692819;IPI00693338;IPI00693628;IPI00693755;IPI00694739;IPI00694751;IPI00694849;IPI00695506;IPI00695508;IPI00695600;IPI00695965;IPI00696729;IPI00696912;IPI00696930;IPI00698900;IPI00699355;IPI00699798;IPI00699803;IPI00700295;IPI00700542;IPI00701790;IPI00703380;IPI00703547;IPI00703753;IPI00704735;IPI00705463;IPI00705660;IP	Molecular Function	Ion binding	4	71	211	0.7581
GO:0004175	IPI00686966;IPI00695176;IPI00697757;IPI00707875	Molecular Function	Endopeptidase activity	7	4	211	0.7600
GO:0004252	IPI00686966	Molecular Function	Serine-type endopeptidase activity	8	1	211	0.7639
GO:0016903	IPI00692627	Molecular Function	Oxidoreductase activity, acting on the aldehyde or oxo group of donors	5	1	211	0.7639
GO:0019899	IPI00702620	Molecular Function	Enzyme binding	5	1	211	0.7639
GO:0042803	IPI00686225	Molecular Function	Protein homodimerization activity	6	1	211	0.7639
GO:0016746	IPI00686601;IPI00708438	Molecular Function	Transferase activity, transferring acyl groups	5	2	211	0.7887
GO:0008509	IPI00703129	Molecular Function	Anion transmembrane transporter activity	7	1	211	0.7918
GO:0016798	IPI00706203	Molecular Function	Hydrolase activity, acting on glycosyl bonds	5	1	211	0.7918
GO:0042626	IPI00716195	Molecular Function	ATPase activity, coupled to transmembrane movement of substances	12	1	211	0.8045
GO:0042802	IPI00686225	Molecular Function	Identical protein binding	5	1	211	0.8045
GO:0046983	IPI00686225	Molecular Function	Protein dimerization activity	5	1	211	0.8045

GO:0003676	IPI00685278;IPI00686841;IPI00687625;IPI00690232;IPI00691963;IPI00693324;IPI00695331;IPI00695600;IPI00695890;IPI00697070;IPI00697891;IPI00698039;IPI00699355;IPI00701698;IPI00703753;IPI00703776;IPI00704728;IPI00705000;IPI00705463;IPI00708018;IPI00712775;IPI00715091;IPI00717759;IPI00742596;IPI00839224;IPI01018577	Molecular Function	Nucleic acid binding	5	26	211	0.8134
GO:0016779	IPI00730144	Molecular Function	Nucleotidyltransferase activity	6	1	211	0.8165
GO:0016820	IPI00716195	Molecular Function	Hydrolase activity, acting on acid anhydrides, catalyzing transmembrane movement of substances	6	1	211	0.8165
GO:0043492	IPI00716195	Molecular Function	ATPase activity, coupled to movement of substances	11	1	211	0.8165
GO:0016757	IPI00686225;IPI00883375	Molecular Function	Transferase activity, transferring glycosyl groups	5	2	211	0.8168
GO:0016829	IPI00694739;IPI00702650	Molecular Function	Lyase activity	4	2	211	0.8168
GO:0046914	IPI00687116;IPI00687842;IPI00692468;IPI00692676;IPI00695600;IPI00695965;IPI00699355;IPI00700542;IPI00705463;IPI00716555;IPI00718193;IPI00829551;IPI00944429;IPI01018577	Molecular Function	Transition metal ion binding	7	14	211	0.8247
GO:0008236	IPI00686966	Molecular Function	Serine-type peptidase activity	7	1	211	0.8277
GO:0017171	IPI00686966	Molecular Function	Serine hydrolase activity	5	1	211	0.8277

GO:0035091	IPI00706141	Molecular Function	Phosphatidylinositol binding	6	1	211	0.8277
GO:0003674	IPI00685278;IPI00685792;IPI00686183;IPI00686225;IPI00686601;IPI00686803;IPI00686841;IPI00686966;IPI00687116;IPI00687539;IPI00687625;IPI00687842;IPI00688489;IPI00688608;IPI00688651;IPI00689228;IPI00689323;IPI00689325;IPI00689760;IPI00690094;IPI00690160;IPI00690232;IPI00690308;IPI00690446;IPI00690785;IPI00691963;IPI00692468;IPI00692627;IPI00692676;IPI00692819;IPI00693324;IPI00693338;IPI00693628;IPI00693755;IPI00694126;IPI00694204;IPI00694312;IPI00694580;IPI00694739;IPI00694751;IPI00694849;IP	Molecular Function	Molecular_function	2	148	211	0.8319
GO:0000287	IPI00686225;IPI00944429	Molecular Function	Magnesium ion binding	7	2	211	0.8336
GO:0015399	IPI00716195	Molecular Function	Primary active transmembrane transporter activity	6	1	211	0.8382
GO:0015405	IPI00716195	Molecular Function	P-P-bond-hydrolysis-driven transmembrane transporter activity	7	1	211	0.8382
GO:0003677	IPI00695331;IPI00695890;IPI00698039;IPI00699355;IPI00703776;IPI00705000;IPI00705463;IPI01018577	Molecular Function	DNA binding	6	8	211	0.8404
GO:0008168	IPI00694126	Molecular Function	Methyltransferase activity	6	1	211	0.8481
GO:0016881	IPI00700547;IPI01018577	Molecular Function	Amino acid ligase activity	6	2	211	0.8491

GO:0008270	IPI00687116;IPI00692676;IPI00695600;IPI00699355;IPI00700542;IPI00705463;IPI00716555;IPI00718193;IPI00829551;IPI01018577	Molecular Function	Zinc ion binding	8	10	211	0.8568
GO:0008238	IPI00700542	Molecular Function	Exopeptidase activity	7	1	211	0.8574
GO:0016627	IPI00694312	Molecular Function	Oxidoreductase activity, acting on the CH-CH group of donors	5	1	211	0.8574
GO:0016741	IPI00694126	Molecular Function	Transferase activity, transferring one-carbon groups	5	1	211	0.8574
GO:0016758	IPI00883375	Molecular Function	Transferase activity, transferring hexosyl groups	6	1	211	0.8574
GO:0022892	IPI00686601;IPI00694849;IPI00695965;IPI00697184;IPI00703129;IPI00716195	Molecular Function	Substrate-specific transporter activity	4	6	211	0.8584
GO:0016787	IPI00686803;IPI00686841;IPI00686966;IPI00687539;IPI00688489;IPI00690308;IPI00692819;IPI00693338;IPI00695176;IPI00697757;IPI00698589;IPI00699798;IPI00700542;IPI00700789;IPI00701790;IPI00703753;IPI00705941;IPI00706203;IPI00707875;IPI00708018;IPI00711419;IPI00712775;IPI00716195;IPI00716555;IPI00716843;IPI00716881;IPI00842934;IPI00845215;IPI00944429	Molecular Function	Hydrolase activity	4	29	211	0.8625

GO:0022890	IPI00694849;IPI00716195	Molecular Function	Inorganic cation transmembrane transporter activity	8	2	211	0.8698
GO:0016747	IPI00686601	Molecular Function	Transferase activity, transferring acyl groups other than amino-acyl groups	6	1	211	0.8743
GO:0070011	IPI00686966;IPI00695176;IPI00697757;IPI00700542;IPI00707875	Molecular Function	Peptidase activity, acting on L-amino acid peptides	6	5	211	0.8816
GO:0016874	IPI00696912;IPI00700295;IPI00700547;IPI00708438;IPI01018577	Molecular Function	Ligase activity	4	5	211	0.8858
GO:0005506	IPI00687842	Molecular Function	Iron ion binding	8	1	211	0.8892
GO:0044212	IPI00703776	Molecular Function	Transcription regulatory region DNA binding	8	1	211	0.8892
GO:0050660	IPI00707559	Molecular Function	Flavin adenine dinucleotide binding	6	1	211	0.8892
GO:0000975	IPI00703776	Molecular Function	Regulatory region DNA binding	7	1	211	0.8960
GO:0001067	IPI00703776	Molecular Function	Regulatory region nucleic acid binding	6	1	211	0.8960
GO:0003743	IPI00693324	Molecular Function	Translation initiation factor activity	8	1	211	0.9024
GO:0008237	IPI00700542	Molecular Function	Metallopeptidase activity	7	1	211	0.9024
GO:0015075	IPI00694849;IPI00703129;IPI00716195	Molecular Function	Ion transmembrane transporter activity	6	3	211	0.9024
GO:0008233	IPI00686966;IPI00695176;IPI00697757;IPI00700542;IPI00707875	Molecular Function	Peptidase activity	5	5	211	0.9118
GO:0022804	IPI00716195	Molecular Function	Active transmembrane transporter activity	5	1	211	0.9140
GO:0008324	IPI00694849;IPI00716195	Molecular Function	Cation transmembrane transporter activity	7	2	211	0.9173
GO:0016879	IPI00700547;IPI01018577	Molecular Function	Ligase activity, forming carbon-nitrogen bonds	5	2	211	0.9327
GO:0022891	IPI00694849;IPI00703129;IPI00716195	Molecular Function	Substrate-specific transmembrane transporter activity	5	3	211	0.9350
GO:0005096	IPI00703776	Molecular Function	GTPase activator activity	5	1	211	0.9373
GO:0004672	IPI00688651;IPI00696930;IPI01018577	Molecular Function	Protein kinase activity	7	3	211	0.9434
GO:0016773	IPI00688651;IPI00696930;IPI00726650;IPI00730144;IPI01018577	Molecular Function	Phosphotransferase activity, alcohol group as acceptor	6	5	211	0.9445

GO:0003735	IPI00713536;IPI00715091	Molecular Function	Structural constituent of ribosome	4	2	211	0.9454
GO:0003682	IPI00695890	Molecular Function	Chromatin binding	4	1	211	0.9514
GO:0022857	IPI00694849;IPI00703129;IPI00716195	Molecular Function	Transmembrane transporter activity	4	3	211	0.9531
GO:0036094	IPI00685278;IPI00686225;IPI00686601;IPI00687625;IPI00688489;IPI00688651;IPI00690446;IPI00694312;IPI00695600;IPI00695965;IPI00696912;IPI00697184;IPI00697891;IPI00698900;IPI00699803;IPI00700295;IPI00701790;IPI00702650;IPI00703129;IPI00703380;IPI00703547;IPI00703753;IPI00704735;IPI00705463;IPI00705941;IPI00707559;IPI00707718;IPI00708438;IPI00712671;IPI00712775;IPI00714302;IPI00716843;IPI00717759;IPI00730144;IPI00742596;IPI00839224;IPI00867339	Molecular Function	Small molecule binding	4	37	211	0.9559
GO:0005215	IPI00686601;IPI00694849;IPI00695965;IPI00697184;IPI00703129;IPI00716195	Molecular Function	Transporter activity	3	6	211	0.9580
GO:0016887	IPI00688489;IPI00716195	Molecular Function	ATPase activity	9	2	211	0.9741
GO:0016462	IPI00688489;IPI00701790;IPI00703753;IPI00705941;IPI00712775;IPI00716195;IPI00716843	Molecular Function	Pyrophosphatase activity	7	7	211	0.9765

GO:0016818	IPI00688489;IPI00701790;IPI00703753;IPI00705941;IPI00712775;IPI00716195;IPI00716843	Molecular Function	Hydrolase activity, acting on acid anhydrides, in phosphorus-containing anhydrides	6	7	211	0.9765
GO:0016817	IPI00688489;IPI00701790;IPI00703753;IPI00705941;IPI00712775;IPI00716195;IPI00716843	Molecular Function	Hydrolase activity, acting on acid anhydrides	5	7	211	0.9774
GO:0016301	IPI00688651;IPI00696930;IPI00726650;IPI00730144;IPI01018577	Molecular Function	Kinase activity	6	5	211	0.9777
GO:0017111	IPI00688489;IPI00701790;IPI00705941;IPI00712775;IPI00716195;IPI00716843	Molecular Function	Nucleoside-triphosphatase activity	8	6	211	0.9811

GO:0000166	IPI00685278;IPI00686225;IPI00687625;IPI00688489;IPI00688651;IPI00690446;IPI00694312;IPI00695600;IPI00696912;IPI00697891;IPI00698900;IPI00699803;IPI00700295;IPI00701790;IPI00702650;IPI00703129;IPI00703380;IPI00703753;IPI00704735;IPI00705463;IPI00705941;IPI00707559;IPI00707718;IPI00708438;IPI00712671;IPI00712775;IPI00714302;IPI00716843;IPI00717759;IPI00730144;IPI00742596;IPI00839224;IPI00867339	Molecular Function	Nucleotide binding	5	33	211	0.9820

GO:1901265	IPI00685278;IPI00686225;IPI00687625;IPI00688489;IPI00688651;IPI00690446;IPI00694312;IPI00695600;IPI00696912;IPI00697891;IPI00698900;IPI00699803;IPI00700295;IPI00701790;IPI00702650;IPI00703129;IPI00703380;IPI00703753;IPI00704735;IPI00705463;IPI00705941;IPI00707559;IPI00707718;IPI00708438;IPI00712671;IPI00712775;IPI00714302;IPI00716843;IPI00717759;IPI00730144;IPI00742596;IPI00839224;IPI00867339	Molecular Function	Nucleoside phosphate binding	5	33	211	0.9820

GO:0097159	IPI00685278;IPI00686225;IPI00686601;IPI00686841;IPI00687625;IPI00688489;IPI00688651;IPI00690232;IPI00690446;IPI00690785;IPI00691963;IPI00693324;IPI00694312;IPI00695331;IPI00695600;IPI00695890;IPI00696912;IPI00697070;IPI00697891;IPI00698039;IPI00698900;IPI00699355;IPI00699803;IPI00700295;IPI00701698;IPI00701790;IPI00702650;IPI00703129;IPI00703380;IPI00703547;IPI00703753;IPI00703776;IPI00704728;IPI00704735;IPI00705000;IPI00705463;IPI00705941;IPI00707559;IPI00707718;IPI00708018;IPI00708438;IP	Molecular Function	Organic cyclic compound binding	4	52	211	0.9874
GO:0042623	IPI00716195	Molecular Function	ATPase activity, coupled	10	1	211	0.9880
GO:0004674	IPI00688651	Molecular Function	Protein serine/threonine kinase activity	8	1	211	0.9888
GO:0030695	IPI00703776	Molecular Function	GTPase regulator activity	5	1	211	0.9923
GO:0005543	IPI00695965;IPI00706141	Molecular Function	Phospholipid binding	5	2	211	0.9928

GO:0001883	IPI00688489;IPI00688651;IPI00696912;IPI00698900;IPI00699803;IPI00700295;IPI00701790;IPI00703380;IPI00703753;IPI00704735;IPI00705941;IPI00707718;IPI00708438;IPI00712775;IPI00714302;IPI00716843;IPI00730144;IPI00867339	Molecular Function	Purine nucleoside binding	6	18	211	0.9931
GO:0032550	IPI00688489;IPI00688651;IPI00696912;IPI00698900;IPI00699803;IPI00700295;IPI00701790;IPI00703380;IPI00703753;IPI00704735;IPI00705941;IPI00707718;IPI00708438;IPI00712775;IPI00714302;IPI00716843;IPI00730144;IPI00867339	Molecular Function	Purine ribonucleoside binding	7	18	211	0.9931

GO:1901363	IPI00685278;IPI00686225;IPI00686841;IPI00687625;IPI00688489;IPI00688651;IPI00690232;IPI00690446;IPI00690785;IPI00691963;IPI00693324;IPI00694312;IPI00695331;IPI00695600;IPI00695890;IPI00696912;IPI00697070;IPI00697891;IPI00698039;IPI00698900;IPI00699355;IPI00699803;IPI00700295;IPI00701698;IPI00701790;IPI00702650;IPI00703129;IPI00703380;IPI00703753;IPI00703776;IPI00704728;IPI00704735;IPI00705000;IPI00705463;IPI00705941;IPI00707559;IPI00707718;IPI00708018;IPI00708438;IPI00712671;IPI00712775;IP	Molecular Function	Heterocyclic compound binding	4	50	211	0.9932
GO:0060589	IPI00703776	Molecular Function	Nucleoside-triphosphatase regulator activity	4	1	211	0.9933
GO:0032549	IPI00688489;IPI00688651;IPI00696912;IPI00698900;IPI00699803;IPI00700295;IPI00701790;IPI00703380;IPI00703753;IPI00704735;IPI00705941;IPI00707718;IPI00708438;IPI00712775;IPI00714302;IPI00716843;IPI00730144;IPI00867339	Molecular Function	Ribonucleoside binding	6	18	211	0.9933

GO:0001882	IPI00688489;IPI00688651;IPI00696912;IPI00698900;IPI00699803;IPI00700295;IPI00701790;IPI00703380;IPI00703753;IPI00704735;IPI00705941;IPI00707718;IPI00708438;IPI00712775;IPI00714302;IPI00716843;IPI00730144;IPI00867339	Molecular Function	Nucleoside binding	5	18	211	0.9935
GO:0016772	IPI00688651;IPI00696930;IPI00726650;IPI00730144;IPI01018577	Molecular Function	Transferase activity, transferring phosphorus-containing groups	5	5	211	0.9936
GO:0032555	IPI00688489;IPI00688651;IPI00696912;IPI00698900;IPI00699803;IPI00700295;IPI00701790;IPI00703380;IPI00703753;IPI00704735;IPI00705941;IPI00707718;IPI00708438;IPI00712775;IPI00714302;IPI00716843;IPI00730144;IPI00867339	Molecular Function	Purine ribonucleotide binding	7	18	211	0.9939

GO:0017076	IPI00688489;IPI00688651;IPI00696912;IPI00698900;IPI00699803;IPI00700295;IPI00701790;IPI00703380;IPI00703753;IPI00704735;IPI00705941;IPI00707718;IPI00708438;IPI00712775;IPI00714302;IPI00716843;IPI00730144;IPI00867339	Molecular Function	Purine nucleotide binding	6	18	211	0.9943
GO:0032553	IPI00688489;IPI00688651;IPI00696912;IPI00698900;IPI00699803;IPI00700295;IPI00701790;IPI00703380;IPI00703753;IPI00704735;IPI00705941;IPI00707718;IPI00708438;IPI00712775;IPI00714302;IPI00716843;IPI00730144;IPI00867339	Molecular Function	Ribonucleotide binding	6	18	211	0.9952
GO:0035639	IPI00688489;IPI00688651;IPI00698900;IPI00699803;IPI00700295;IPI00701790;IPI00703380;IPI00703753;IPI00704735;IPI00705941;IPI00707718;IPI00708438;IPI00712775;IPI00714302;IPI00716843;IPI00730144;IPI00867339	Molecular Function	Purine ribonucleoside triphosphate binding	6	17	211	0.9961

GO:0016740	IPI00686225;IPI00686601;IPI00688651;IPI00694126;IPI00696930;IPI00708438;IPI00711759;IPI00726650;IPI00730144;IPI00883375;IPI00904104;IPI01018577	Molecular Function	Transferase activity	4	12	211	0.9962
GO:0003824	IPI00686183;IPI00686225;IPI00686601;IPI00686803;IPI00686841;IPI00686966;IPI00687539;IPI00687842;IPI00688489;IPI00688651;IPI00689323;IPI00689325;IPI00690308;IPI00690446;IPI00690785;IPI00692468;IPI00692627;IPI00692819;IPI00693338;IPI00694126;IPI00694312;IPI00694580;IPI00694739;IPI00694849;IPI00695176;IPI00696912;IPI00696930;IPI00697757;IPI00698589;IPI00699798;IPI00700295;IPI00700542;IPI00700547;IPI00700789;IPI00700920;IPI00701790;IPI00702650;IPI00703731;IPI00703753;IPI00705941;IPI00706203;IP	Molecular Function	Catalytic activity	3	65	211	0.9975

GO:0043168	IPI00686601;IPI00687539;IPI00688489;IPI00688651;IPI00693338;IPI00695965;IPI00696912;IPI00698900;IPI00699355;IPI00699803;IPI00700295;IPI00701790;IPI00703380;IPI00703753;IPI00704735;IPI00705941;IPI00706141;IPI00706624;IPI00707559;IPI00707718;IPI00708438;IPI00712775;IPI00714302;IPI00715486;IPI00716843;IPI00718698;IPI00730144;IPI00867339	Molecular Function	Anion binding	5	28	211	0.9976
GO:0032559	IPI00688489;IPI00688651;IPI00696912;IPI00698900;IPI00700295;IPI00701790;IPI00703753;IPI00704735;IPI00730144;IPI00867339	Molecular Function	Adenyl ribonucleotide binding	8	10	211	0.9993
GO:0030554	IPI00688489;IPI00688651;IPI00696912;IPI00698900;IPI00700295;IPI00701790;IPI00703753;IPI00704735;IPI00730144;IPI00867339	Molecular Function	Adenyl nucleotide binding	7	10	211	0.9994

GO:0005524	IPI00688489;IPI00688651;IPI00698900;IPI00700295;IPI00701790;IPI00703753;IPI00704735;IPI00730144;IPI00867339	Molecular Function	ATP binding	9	9	211	0.9997

S gene number indicated the number of significantly up-regulated proteins annotated to one certain GO term by GO analysis; TS gene number indicated the total number of significantly up-regulated proteins annotated by GO analysis.

Table S8 GO categories assigned to the differentially down-regulated proteins in mammary glands between RS- and AH-fed dairy cows

GO id	Gene list	GO term	GO description	GO class	S gene number	TS gene number	P-value of Fisher's Exact Test
GO:0008150	IPI00685510;IPI00685617;IPI00685653;IPI00685678;IPI00685769;IPI00685804;IPI00686595;IPI00686872;IPI00687375;IPI00687395;IPI00687478;IPI00687578;IPI00687634;IPI00687661;IPI00687662;IPI00687839;IPI00688337;IPI00688400;IPI00688446;IPI00688451;IPI00688474;IPI00688704;IPI00688727;IPI00688980;IPI00689165;IPI00689226;IPI00689365;IPI00689379;IPI00689565;IPI00689629;IPI00689641;IPI00689899;IPI00690309;IPI00690581;IPI00690789;IPI00691086;IPI00691093;IPI00691649;IPI00691826;IPI00692417;IPI00692668;IPI00692739;IPI00692963;IPI00693452;IPI00693579;IPI00693824;IPI00693842;IPI006	Biological Process	Biological_process	2	194	246	0.6863

GO:0009987	IPI00685510;IPI00685617;IPI00685769;IPI00685804;IPI00686872;IPI00687395;IPI00687478;IPI00687578;IPI00687634;IPI00687661;IPI00687662;IPI00687839;IPI00688337;IPI00688400;IPI00688446;IPI00688474;IPI00688704;IPI00688727;IPI00688980;IPI00689226;IPI00689365;IPI00689379;IPI00689565;IPI00689629;IPI00689641;IPI00690309;IPI00690581;IPI00690789;IPI00691093;IPI00691649;IPI00691826;IPI00692417;IPI00692963;IPI00693579;IPI00693824;IPI00694198;IPI00694430;IPI00694444;IPI00694565;IPI00694688;IPI00695042;IPI00695852;IPI00695952;IPI00696116;IPI00696748;IPI00697194;IPI00697349;IPI006	Biological Process	Cellular process	3	158	246	0.6936

GO:0044699	IPI00685510;IPI00685769;IPI00686595;IPI00686872;IPI00687478;IPI00687578;IPI00688400;IPI00688474;IPI00688704;IPI00688980;IPI00689226;IPI00689365;IPI00689379;IPI00689641;IPI00690309;IPI00690581;IPI00691086;IPI00691093;IPI00691826;IPI00692417;IPI00692963;IPI00693452;IPI00693579;IPI00693824;IPI00694198;IPI00694597;IPI00695042;IPI00695852;IPI00696116;IPI00696748;IPI00697349;IPI00697595;IPI00697648;IPI00698059;IPI00698213;IPI00698339;IPI00698823;IPI00698916;IPI00699039;IPI00699372;IPI00700228;IPI00702768;IPI00703084;IPI00703813;IPI00703954;IPI00704902;IPI00705104;IPI007	Biological Process	Single-organism process	3	112	246	0.5977

GO:0008152	IPI00685510;IPI00685617;IPI00685804;IPI00686872;IPI00687395;IPI00687478;IPI00687634;IPI00687661;IPI00687662;IPI00687839;IPI00688337;IPI00688446;IPI00688727;IPI00689165;IPI00689365;IPI00689379;IPI00689565;IPI00689629;IPI00689641;IPI00690309;IPI00690581;IPI00690789;IPI00691093;IPI00691649;IPI00694430;IPI00694444;IPI00694565;IPI00694597;IPI00694688;IPI00695952;IPI00696748;IPI00697194;IPI00698059;IPI00698077;IPI00698213;IPI00698339;IPI00698823;IPI00698916;IPI00699039;IPI00699226;IPI00699827;IPI00700228;IPI00701747;IPI00702948;IPI00703022;IPI00703084;IPI00704041;IPI007	Biological Process	Metabolic process	3	104	246	0.8426

GO:0071704	IPI00685510;IPI00685617;IPI00685804;IPI00686872;IPI00687395;IPI00687634;IPI00687661;IPI00687662;IPI00687839;IPI00688337;IPI00688446;IPI00688727;IPI00689165;IPI00689365;IPI00689379;IPI00689565;IPI00689629;IPI00689641;IPI00690309;IPI00690581;IPI00690789;IPI00691093;IPI00691649;IPI00694430;IPI00694444;IPI00694565;IPI00694597;IPI00694688;IPI00695952;IPI00696748;IPI00697194;IPI00698059;IPI00698077;IPI00698213;IPI00698339;IPI00698823;IPI00698916;IPI00699039;IPI00699226;IPI00699827;IPI00700228;IPI00701747;IPI00702948;IPI00703022;IPI00703084;IPI00704041;IPI00704332;IPI007	Biological Process	Organic substance metabolic process	4	100	246	0.6704

GO:0044763	IPI00685510;IPI00685769;IPI00686872;IPI00687478;IPI00687578;IPI00688400;IPI00688474;IPI00688704;IPI00688980;IPI00689226;IPI00689365;IPI00689379;IPI00689641;IPI00690309;IPI00690581;IPI00691826;IPI00692417;IPI00692963;IPI00693579;IPI00693824;IPI00694198;IPI00695042;IPI00695852;IPI00696116;IPI00696748;IPI00697349;IPI00697595;IPI00697648;IPI00698059;IPI00698213;IPI00698339;IPI00698823;IPI00698916;IPI00699372;IPI00700228;IPI00702768;IPI00703084;IPI00703813;IPI00703954;IPI00704902;IPI00705104;IPI00705237;IPI00705378;IPI00705583;IPI00706142;IPI00706314;IPI00707455;IPI007	Biological Process	Single-organism cellular process	4	100	246	0.5854

GO:0044237	IPI00685510;IPI00685617;IPI00685804;IPI00686872;IPI00687395;IPI00687478;IPI00687634;IPI00687661;IPI00687662;IPI00687839;IPI00688337;IPI00688446;IPI00688727;IPI00689365;IPI00689565;IPI00689629;IPI00689641;IPI00690309;IPI00690581;IPI00690789;IPI00691093;IPI00691649;IPI00694430;IPI00694444;IPI00694565;IPI00694688;IPI00695952;IPI00696748;IPI00697194;IPI00698059;IPI00698077;IPI00698213;IPI00698339;IPI00698823;IPI00698916;IPI00699039;IPI00699226;IPI00699827;IPI00700228;IPI00701747;IPI00702948;IPI00703022;IPI00703084;IPI00704041;IPI00704332;IPI00704902;IPI00705104;IPI007	Biological Process	Cellular metabolic process	4	93	246	0.7523

GO:0044238	IPI00685510;IPI00685617;IPI00685804;IPI00686872;IPI00687395;IPI00687634;IPI00687661;IPI00687662;IPI00687839;IPI00688337;IPI00688446;IPI00688727;IPI00689165;IPI00689365;IPI00689379;IPI00689565;IPI00689629;IPI00689641;IPI00690309;IPI00690581;IPI00690789;IPI00691649;IPI00694430;IPI00694444;IPI00694565;IPI00694597;IPI00694688;IPI00696748;IPI00697194;IPI00698077;IPI00698213;IPI00698339;IPI00698823;IPI00698916;IPI00699039;IPI00699226;IPI00699827;IPI00700228;IPI00701747;IPI00702948;IPI00703022;IPI00703084;IPI00704041;IPI00704332;IPI00704902;IPI00705104;IPI00705159;IPI007	Biological Process	Primary metabolic process	4	92	246	0.7986

GO:0065007	IPI00685510;IPI00685617;IPI00685678;IPI00687375;IPI00687478;IPI00688451;IPI00688704;IPI00688980;IPI00689379;IPI00690309;IPI00690581;IPI00690789;IPI00691826;IPI00692668;IPI00693452;IPI00693579;IPI00693824;IPI00693842;IPI00694198;IPI00694430;IPI00694565;IPI00694688;IPI00695037;IPI00695042;IPI00696116;IPI00696748;IPI00697595;IPI00697917;IPI00698059;IPI00698213;IPI00698339;IPI00698823;IPI00698916;IPI00702948;IPI00703084;IPI00703813;IPI00703925;IPI00704902;IPI00705159;IPI00705237;IPI00705441;IPI00705583;IPI00706142;IPI00706314;IPI00706879;IPI00707685;IPI00707776;IPI007	Biological Process	Biological regulation	3	91	246	0.6572

GO:0050789	IPI00685510;IPI00685617;IPI00685678;IPI00687375;IPI00687478;IPI00688451;IPI00688704;IPI00688980;IPI00689379;IPI00690309;IPI00690581;IPI00690789;IPI00691826;IPI00692668;IPI00693452;IPI00693579;IPI00693824;IPI00693842;IPI00694198;IPI00694430;IPI00694565;IPI00694688;IPI00695037;IPI00695042;IPI00696116;IPI00696748;IPI00697595;IPI00697917;IPI00698213;IPI00698339;IPI00698823;IPI00698916;IPI00703084;IPI00703925;IPI00704902;IPI00705159;IPI00705237;IPI00705441;IPI00705583;IPI00706142;IPI00706314;IPI00706879;IPI00707685;IPI00707776;IPI00707857;IPI00708070;IPI00708244;IPI007	Biological Process	Regulation of biological process	4	85	246	0.6755

GO:0050794	IPI00685510;IPI00685617;IPI00687478;IPI00688451;IPI00688704;IPI00688980;IPI00689379;IPI00690309;IPI00690581;IPI00690789;IPI00691826;IPI00692668;IPI00693579;IPI00693824;IPI00693842;IPI00694198;IPI00694430;IPI00694565;IPI00694688;IPI00695037;IPI00695042;IPI00696116;IPI00696748;IPI00697595;IPI00697917;IPI00698213;IPI00698823;IPI00698916;IPI00703084;IPI00703925;IPI00704902;IPI00705159;IPI00705237;IPI00705441;IPI00705583;IPI00706142;IPI00706314;IPI00707776;IPI00707857;IPI00708070;IPI00708244;IPI00709124;IPI00709876;IPI00709888;IPI00710274;IPI00711457;IPI00712263;IPI007	Biological Process	Regulation of cellular process	4	77	246	0.7009

GO:0043170	IPI00685510;IPI00685804;IPI00687395;IPI00687634;IPI00687662;IPI00687839;IPI00688337;IPI00688446;IPI00688727;IPI00689165;IPI00689365;IPI00689379;IPI00689565;IPI00689629;IPI00689641;IPI00690309;IPI00690581;IPI00691649;IPI00694444;IPI00694565;IPI00694597;IPI00694688;IPI00696748;IPI00698077;IPI00698213;IPI00698339;IPI00698823;IPI00698916;IPI00699039;IPI00699827;IPI00700228;IPI00701747;IPI00702948;IPI00703022;IPI00704041;IPI00704902;IPI00705104;IPI00705739;IPI00706142;IPI00707386;IPI00707455;IPI00707537;IPI00707776;IPI00707815;IPI00707857;IPI00708210;IPI00708246;IPI007	Biological Process	Macromolecule metabolic process	5	74	246	0.2974

GO:0044260	IPI00685510;IPI00685804;IPI00687395;IPI00687634;IPI00687662;IPI00687839;IPI00688337;IPI00688446;IPI00688727;IPI00689365;IPI00689565;IPI00689629;IPI00689641;IPI00690309;IPI00690581;IPI00691649;IPI00694444;IPI00694565;IPI00694688;IPI00696748;IPI00698077;IPI00698213;IPI00698339;IPI00698823;IPI00698916;IPI00699039;IPI00699827;IPI00700228;IPI00701747;IPI00702948;IPI00703022;IPI00704041;IPI00704902;IPI00705104;IPI00705739;IPI00706142;IPI00707386;IPI00707455;IPI00707537;IPI00707776;IPI00707815;IPI00708210;IPI00708246;IPI00709124;IPI00709409;IPI00709876;IPI00710859;IPI007	Biological Process	Cellular macromolecule metabolic process	5	65	246	0.2586

GO:0051179	IPI00685510;IPI00685617;IPI00686872;IPI00687578;IPI00689226;IPI00690581;IPI00691086;IPI00691826;IPI00692417;IPI00692739;IPI00692963;IPI00693452;IPI00693824;IPI00694198;IPI00694597;IPI00695852;IPI00696116;IPI00697349;IPI00697595;IPI00698213;IPI00698823;IPI00699372;IPI00700228;IPI00701747;IPI00703084;IPI00703813;IPI00703954;IPI00705378;IPI00705933;IPI00706037;IPI00706314;IPI00707815;IPI00707857;IPI00708037;IPI00708162;IPI00708597;IPI00709876;IPI00709888;IPI00710204;IPI00710377;IPI00710664;IPI00710920;IPI00711457;IPI00712858;IPI00713460;IPI00713474;IPI00713597;IPI007	Biological Process	Localization	3	65	246	0.0176

GO:0050896	IPI00685510;IPI00685653;IPI00685678;IPI00688704;IPI00688980;IPI00690309;IPI00690581;IPI00691826;IPI00693824;IPI00694198;IPI00695042;IPI00696116;IPI00696748;IPI00697595;IPI00698213;IPI00698823;IPI00700673;IPI00702768;IPI00703084;IPI00703813;IPI00705159;IPI00705565;IPI00705583;IPI00707455;IPI00707776;IPI00707815;IPI00707857;IPI00708244;IPI00709124;IPI00709876;IPI00710664;IPI00710920;IPI00711457;IPI00712399;IPI00712524;IPI00712596;IPI00713049;IPI00713474;IPI00713597;IPI00714656;IPI00715059;IPI00716157;IPI00717764;IPI00717930;IPI00718152;IPI00718678;IPI00727050;IPI007	Biological Process	Response to stimulus	3	59	246	0.4741

GO:0051234	IPI00685510;IPI00686872;IPI00689226;IPI00690581;IPI00691086;IPI00691826;IPI00692417;IPI00692739;IPI00692963;IPI00693452;IPI00693824;IPI00694198;IPI00694597;IPI00695852;IPI00696116;IPI00697349;IPI00698213;IPI00698823;IPI00699372;IPI00700228;IPI00701747;IPI00703084;IPI00703813;IPI00703954;IPI00705378;IPI00705933;IPI00706037;IPI00706314;IPI00707815;IPI00707857;IPI00708037;IPI00708162;IPI00708597;IPI00709876;IPI00709888;IPI00710204;IPI00710377;IPI00710664;IPI00710920;IPI00711457;IPI00712858;IPI00713460;IPI00713474;IPI00714269;IPI00714656;IPI00714660;IPI00717764;IPI007	Biological Process	Establishment of localization	4	57	246	0.0099

GO:0006810	IPI00685510;IPI00686872;IPI00689226;IPI00690581;IPI00691086;IPI00691826;IPI00692417;IPI00692739;IPI00692963;IPI00693452;IPI00693824;IPI00694198;IPI00694597;IPI00695852;IPI00696116;IPI00697349;IPI00698213;IPI00698823;IPI00699372;IPI00700228;IPI00701747;IPI00703084;IPI00703813;IPI00703954;IPI00705378;IPI00705933;IPI00706037;IPI00706314;IPI00707815;IPI00707857;IPI00708037;IPI00708162;IPI00708597;IPI00709876;IPI00709888;IPI00710204;IPI00710377;IPI00710664;IPI00710920;IPI00711457;IPI00712858;IPI00713460;IPI00713474;IPI00714269;IPI00714656;IPI00714660;IPI00717764;IPI007	Biological Process	Transport	5	57	246	0.0075

GO:0009058	IPI00685617;IPI00686872;IPI00687634;IPI00687661;IPI00687662;IPI00688727;IPI00689365;IPI00689565;IPI00690789;IPI00691093;IPI00694430;IPI00694444;IPI00694565;IPI00694688;IPI00695952;IPI00697194;IPI00698077;IPI00698213;IPI00698823;IPI00699039;IPI00699226;IPI00699827;IPI00701747;IPI00702948;IPI00703084;IPI00704332;IPI00704902;IPI00705159;IPI00705565;IPI00705739;IPI00706142;IPI00706879;IPI00707386;IPI00707537;IPI00707857;IPI00708210;IPI00708246;IPI00709409;IPI00709888;IPI00710377;IPI00710859;IPI00712384;IPI00712524;IPI00714621;IPI00715066;IPI00715868;IPI00716860;IPI007	Biological Process	Biosynthetic process	4	53	246	0.0117

GO:1901576	IPI00685617;IPI00686872;IPI00687634;IPI00687661;IPI00687662;IPI00688727;IPI00689365;IPI00689565;IPI00690789;IPI00691093;IPI00694430;IPI00694444;IPI00694565;IPI00694688;IPI00695952;IPI00697194;IPI00698077;IPI00698213;IPI00698823;IPI00699039;IPI00699226;IPI00699827;IPI00701747;IPI00702948;IPI00703084;IPI00704332;IPI00704902;IPI00705159;IPI00705565;IPI00705739;IPI00706142;IPI00706879;IPI00707386;IPI00707537;IPI00707857;IPI00708210;IPI00708246;IPI00709409;IPI00709888;IPI00710377;IPI00710859;IPI00712384;IPI00712524;IPI00714621;IPI00715066;IPI00715868;IPI00716860;IPI007	Biological Process	Organic substance biosynthetic process	5	53	246	0.0066

GO:0044249	IPI00685617;IPI00686872;IPI00687634;IPI00687661;IPI00687662;IPI00688727;IPI00689365;IPI00689565;IPI00690789;IPI00691093;IPI00694430;IPI00694444;IPI00694565;IPI00694688;IPI00695952;IPI00698077;IPI00698213;IPI00698823;IPI00699039;IPI00699226;IPI00699827;IPI00701747;IPI00702948;IPI00703084;IPI00704332;IPI00704902;IPI00705159;IPI00705565;IPI00705739;IPI00706142;IPI00706879;IPI00707386;IPI00707537;IPI00708210;IPI00708246;IPI00709409;IPI00709888;IPI00710377;IPI00710859;IPI00712384;IPI00712524;IPI00714621;IPI00715066;IPI00715868;IPI00716860;IPI00717048;IPI00718379;IPI007	Biological Process	Cellular biosynthetic process	5	50	246	0.0088

GO:0019538	IPI00685510;IPI00685804;IPI00687634;IPI00687662;IPI00687839;IPI00688727;IPI00689165;IPI00689365;IPI00689379;IPI00689565;IPI00689629;IPI00690309;IPI00690581;IPI00691649;IPI00694444;IPI00694565;IPI00694597;IPI00694688;IPI00698077;IPI00698823;IPI00698916;IPI00699039;IPI00699827;IPI00702948;IPI00704041;IPI00705739;IPI00706142;IPI00707386;IPI00707455;IPI00707537;IPI00707776;IPI00707815;IPI00708210;IPI00708246;IPI00709409;IPI00710664;IPI00710859;IPI00712384;IPI00715414;IPI00715640;IPI00715868;IPI00716860;IPI00717048;IPI00718379;IPI00718678;IPI00727591;IPI00731432;IPI008	Biological Process	Protein metabolic process	5	50	246	0.3520

GO:0071840	IPI00685510;IPI00687578;IPI00688474;IPI00689226;IPI00689365;IPI00689379;IPI00689641;IPI00689899;IPI00690309;IPI00691093;IPI00693579;IPI00694198;IPI00696116;IPI00697648;IPI00698339;IPI00702948;IPI00703954;IPI00704902;IPI00705104;IPI00705237;IPI00706314;IPI00707776;IPI00707815;IPI00707857;IPI00708070;IPI00708162;IPI00708244;IPI00708767;IPI00709124;IPI00709876;IPI00709888;IPI00710274;IPI00710834;IPI00711457;IPI00712288;IPI00712399;IPI00713474;IPI00716830;IPI00718678;IPI00727591;IPI00731432;IPI00826022;IPI00843305;IPI00866975;IPI00924039	Biological Process	Cellular component organization or biogenesis	3	45	246	0.7330

GO:0016043	IPI00685510;IPI00687578;IPI00688474;IPI00689226;IPI00689365;IPI00689379;IPI00689641;IPI00689899;IPI00690309;IPI00691093;IPI00693579;IPI00694198;IPI00696116;IPI00697648;IPI00698339;IPI00703954;IPI00704902;IPI00705104;IPI00705237;IPI00706314;IPI00707776;IPI00707815;IPI00707857;IPI00708070;IPI00708162;IPI00708244;IPI00708767;IPI00709124;IPI00709876;IPI00709888;IPI00710274;IPI00710834;IPI00711457;IPI00712288;IPI00712399;IPI00713474;IPI00716830;IPI00718678;IPI00727591;IPI00731432;IPI00826022;IPI00843305;IPI00866975;IPI00924039	Biological Process	Cellular component organization	4	44	246	0.6754

GO:0006807	IPI00685510;IPI00685804;IPI00686872;IPI00687395;IPI00688337;IPI00688446;IPI00689365;IPI00689641;IPI00691093;IPI00694430;IPI00696748;IPI00698059;IPI00698213;IPI00698339;IPI00699039;IPI00699226;IPI00700228;IPI00701747;IPI00702948;IPI00703022;IPI00704332;IPI00704902;IPI00705104;IPI00705159;IPI00705565;IPI00706142;IPI00708246;IPI00709124;IPI00709888;IPI00710377;IPI00712263;IPI00712399;IPI00712524;IPI00715066;IPI00717764;IPI00717930;IPI00718678;IPI00760457;IPI00839150;IPI00867017;IPI00871114;IPI00876805;IPI01017508;IPI01028182	Biological Process	Nitrogen compound metabolic process	4	44	246	0.7858

GO:0044267	IPI00685510;IPI00685804;IPI00687634;IPI00687662;IPI00687839;IPI00688727;IPI00689365;IPI00689565;IPI00689629;IPI00690309;IPI00690581;IPI00691649;IPI00694444;IPI00694565;IPI00694688;IPI00698077;IPI00698823;IPI00698916;IPI00699827;IPI00702948;IPI00704041;IPI00705739;IPI00706142;IPI00707386;IPI00707455;IPI00707537;IPI00707776;IPI00707815;IPI00708210;IPI00708246;IPI00709409;IPI00710859;IPI00712384;IPI00715868;IPI00716860;IPI00717048;IPI00718379;IPI00718678;IPI00727591;IPI00731432;IPI00825680;IPI00867017;IPI00906031	Biological Process	Cellular protein metabolic process	6	43	246	0.2111

GO:0051716	IPI00685510;IPI00688704;IPI00688980;IPI00690309;IPI00690581;IPI00691826;IPI00693824;IPI00694198;IPI00695042;IPI00696116;IPI00696748;IPI00697595;IPI00698213;IPI00698823;IPI00702768;IPI00707455;IPI00707776;IPI00707815;IPI00707857;IPI00708244;IPI00709876;IPI00711457;IPI00712399;IPI00712524;IPI00713049;IPI00713474;IPI00713597;IPI00714656;IPI00715059;IPI00716157;IPI00717764;IPI00718152;IPI00718678;IPI00727050;IPI00731432;IPI00826022;IPI00829520;IPI00847122;IPI00866858;IPI00908033;IPI01017588;IPI01028455	Biological Process	Cellular response to stimulus	4	42	246	0.2967

GO:0019222	IPI00685510;IPI00685617;IPI00685678;IPI00687375;IPI00688451;IPI00690309;IPI00690581;IPI00692668;IPI00693842;IPI00694430;IPI00694688;IPI00695037;IPI00696748;IPI00698213;IPI00698339;IPI00698823;IPI00698916;IPI00703925;IPI00704902;IPI00705159;IPI00705237;IPI00706142;IPI00707685;IPI00707776;IPI00707857;IPI00709876;IPI00710274;IPI00712263;IPI00712384;IPI00713049;IPI00713597;IPI00714898;IPI00715059;IPI00716725;IPI00717764;IPI00847122;IPI00866858;IPI00866975;IPI00867230;IPI00876805;IPI01000210;IPI01003286	Biological Process	Regulation of metabolic process	4	42	246	0.8620

GO:0032501	IPI00685510;IPI00686595;IPI00687578;IPI00688704;IPI00690309;IPI00691093;IPI00691826;IPI00693824;IPI00696116;IPI00697648;IPI00698059;IPI00698823;IPI00699039;IPI00702768;IPI00703084;IPI00703813;IPI00705159;IPI00705583;IPI00707776;IPI00707857;IPI00708070;IPI00708244;IPI00709124;IPI00709876;IPI00709888;IPI00710274;IPI00710920;IPI00712399;IPI00713049;IPI00713597;IPI00715059;IPI00716830;IPI00717764;IPI00731432;IPI00826022;IPI00839150;IPI00866975;IPI00876805;IPI01003286	Biological Process	Multicellular organismal process	3	39	246	0.8137

GO:0048518	IPI00685510;IPI00685617;IPI00690309;IPI00690789;IPI00693452;IPI00694430;IPI00694565;IPI00695037;IPI00695042;IPI00696116;IPI00697917;IPI00698213;IPI00703084;IPI00704902;IPI00705159;IPI00705237;IPI00705583;IPI00706879;IPI00707776;IPI00707857;IPI00709124;IPI00709876;IPI00709888;IPI00710274;IPI00712858;IPI00713474;IPI00713597;IPI00714269;IPI00714898;IPI00715059;IPI00716725;IPI00716830;IPI00717764;IPI00847122;IPI00852533;IPI00866858;IPI00866975;IPI01028468	Biological Process	Positive regulation of biological process	5	38	246	0.4627

GO:0044765	IPI00685510;IPI00686872;IPI00690581;IPI00691086;IPI00692417;IPI00692963;IPI00693452;IPI00694597;IPI00695852;IPI00696116;IPI00697349;IPI00698823;IPI00699372;IPI00703084;IPI00703813;IPI00703954;IPI00705378;IPI00706314;IPI00707815;IPI00708162;IPI00708597;IPI00709876;IPI00709888;IPI00710377;IPI00710664;IPI00710920;IPI00711457;IPI00712858;IPI00713460;IPI00713474;IPI00714269;IPI00714656;IPI00714660;IPI00717764;IPI00718152;IPI00730219;IPI00866975;IPI01003286	Biological Process	Single-organism transport	4	38	246	0.0162

GO:0034641	IPI00685510;IPI00685804;IPI00686872;IPI00687395;IPI00688337;IPI00688446;IPI00689365;IPI00689641;IPI00691093;IPI00694430;IPI00696748;IPI00698213;IPI00698339;IPI00699226;IPI00700228;IPI00701747;IPI00702948;IPI00703022;IPI00704332;IPI00704902;IPI00705104;IPI00705159;IPI00705565;IPI00706142;IPI00708246;IPI00709124;IPI00709888;IPI00710377;IPI00712263;IPI00712399;IPI00712524;IPI00718678;IPI00839150;IPI00867017;IPI00876805;IPI01017508;IPI01028182	Biological Process	Cellular nitrogen compound metabolic process	5	37	246	0.8500

GO:0032502	IPI00685510;IPI00686595;IPI00687578;IPI00688704;IPI00690309;IPI00691093;IPI00691826;IPI00693824;IPI00696748;IPI00697648;IPI00699039;IPI00702768;IPI00703813;IPI00705583;IPI00706142;IPI00707776;IPI00707857;IPI00708070;IPI00708244;IPI00709124;IPI00709876;IPI00709888;IPI00710274;IPI00710920;IPI00713049;IPI00713313;IPI00713597;IPI00715059;IPI00716830;IPI00717764;IPI00731432;IPI00826022;IPI00839150;IPI00866975;IPI00876805;IPI00924039;IPI01003286	Biological Process	Developmental process	3	37	246	0.7037

GO:0010467	IPI00685510;IPI00685804;IPI00687395;IPI00687634;IPI00687662;IPI00688337;IPI00688446;IPI00688727;IPI00689365;IPI00689565;IPI00694444;IPI00694565;IPI00694688;IPI00698077;IPI00698213;IPI00699827;IPI00700228;IPI00702948;IPI00703022;IPI00704902;IPI00705739;IPI00706142;IPI00707386;IPI00707537;IPI00707815;IPI00708210;IPI00708246;IPI00709409;IPI00710859;IPI00712263;IPI00712384;IPI00712524;IPI00715868;IPI00716860;IPI00717048;IPI00718379;IPI00867017	Biological Process	Gene expression	6	37	246	0.0268

GO:0044707	IPI00685510;IPI00686595;IPI00687578;IPI00688704;IPI00691093;IPI00691826;IPI00693824;IPI00696116;IPI00697648;IPI00698059;IPI00698823;IPI00699039;IPI00702768;IPI00703084;IPI00703813;IPI00705159;IPI00705583;IPI00707776;IPI00707857;IPI00708070;IPI00708244;IPI00709124;IPI00709876;IPI00709888;IPI00710274;IPI00710920;IPI00713049;IPI00713597;IPI00715059;IPI00716830;IPI00717764;IPI00731432;IPI00826022;IPI00839150;IPI00866975;IPI00876805;IPI01003286	Biological Process	Single-multicellular organism process	4	37	246	0.8443

GO:1901360	IPI00685510;IPI00686872;IPI00687395;IPI00688337;IPI00688446;IPI00689365;IPI00689641;IPI00690789;IPI00691093;IPI00694430;IPI00696748;IPI00697194;IPI00698059;IPI00698213;IPI00698339;IPI00698823;IPI00699226;IPI00700228;IPI00701747;IPI00702948;IPI00703022;IPI00704332;IPI00704902;IPI00705104;IPI00705159;IPI00706142;IPI00708246;IPI00709124;IPI00709888;IPI00710377;IPI00712263;IPI00712399;IPI00712524;IPI00718678;IPI00867017;IPI01017508	Biological Process	Organic cyclic compound metabolic process	5	36	246	0.8717

GO:0031323	IPI00685510;IPI00685617;IPI00688451;IPI00690309;IPI00692668;IPI00693842;IPI00694430;IPI00694688;IPI00695037;IPI00696748;IPI00698213;IPI00698823;IPI00698916;IPI00703925;IPI00704902;IPI00705159;IPI00705237;IPI00706142;IPI00707776;IPI00707857;IPI00709876;IPI00710274;IPI00712263;IPI00712384;IPI00713049;IPI00713597;IPI00714898;IPI00715059;IPI00716725;IPI00717764;IPI00847122;IPI00866858;IPI00866975;IPI00876805;IPI01000210;IPI01003286	Biological Process	Regulation of cellular metabolic process	5	36	246	0.8071

GO:0044710	IPI00685510;IPI00685617;IPI00686872;IPI00687478;IPI00687661;IPI00689365;IPI00690789;IPI00694430;IPI00695952;IPI00697194;IPI00698213;IPI00698823;IPI00699039;IPI00699226;IPI00703084;IPI00704332;IPI00705159;IPI00705565;IPI00706879;IPI00707857;IPI00708246;IPI00709876;IPI00709888;IPI00710377;IPI00714621;IPI00715059;IPI00715066;IPI00717758;IPI00730219;IPI00731432;IPI00760457;IPI00839150;IPI00867017;IPI00871114;IPI00906031;IPI01028182	Biological Process	Single-organism metabolic process	4	36	246	0.8767

GO:0048856	IPI00685510;IPI00687578;IPI00688704;IPI00690309;IPI00691093;IPI00691826;IPI00693824;IPI00696748;IPI00697648;IPI00699039;IPI00702768;IPI00703813;IPI00705583;IPI00707776;IPI00707857;IPI00708070;IPI00708244;IPI00709124;IPI00709876;IPI00709888;IPI00710274;IPI00710920;IPI00713049;IPI00713313;IPI00713597;IPI00715059;IPI00716830;IPI00717764;IPI00731432;IPI00826022;IPI00839150;IPI00866975;IPI00876805;IPI00924039;IPI01003286	Biological Process	Anatomical structure development	4	35	246	0.6067

GO:0006725	IPI00685510;IPI00686872;IPI00687395;IPI00688337;IPI00688446;IPI00689365;IPI00689641;IPI00691093;IPI00694430;IPI00696748;IPI00698059;IPI00698213;IPI00698339;IPI00699226;IPI00700228;IPI00701747;IPI00702948;IPI00703022;IPI00704332;IPI00704902;IPI00705104;IPI00705159;IPI00706142;IPI00708246;IPI00709124;IPI00709888;IPI00710377;IPI00712263;IPI00712399;IPI00712524;IPI00712596;IPI00718678;IPI00867017;IPI01017508	Biological Process	Cellular aromatic compound metabolic process	5	34	246	0.8485
GO:0007154	IPI00685510;IPI00688704;IPI00688980;IPI00690309;IPI00690581;IPI00691826;IPI00693824;IPI00694198;IPI00695042;IPI00696116;IPI00697595;IPI00698059;IPI00698213;IPI00707776;IPI00708070;IPI00708244;IPI00709876;IPI00711457;IPI00713049;IPI00713474;IPI00713597;IPI00714656;IPI00715059;IPI00717764;IPI00718152;IPI00727050;IPI00731432;IPI00829520;IPI00847122;IPI00876805;IPI00908033;IPI01017588;IPI01028455	Biological Process	Cell communication	5	33	246	0.3595

GO:0009059	IPI00687634;IPI00687662;IPI00688727;IPI00689365;IPI00689565;IPI00694444;IPI00694565;IPI00694688;IPI00698077;IPI00698213;IPI00698823;IPI00699039;IPI00699827;IPI00701747;IPI00702948;IPI00704902;IPI00705739;IPI00706142;IPI00707386;IPI00707537;IPI00707857;IPI00708210;IPI00708246;IPI00709409;IPI00710859;IPI00712384;IPI00712524;IPI00715868;IPI00716860;IPI00717048;IPI00718379;IPI00731432;IPI00867017	Biological Process	Macromolecule biosynthetic process	6	33	246	0.0136
GO:0080090	IPI00685510;IPI00685617;IPI00688451;IPI00690309;IPI00692668;IPI00693842;IPI00694430;IPI00694688;IPI00695037;IPI00696748;IPI00698213;IPI00704902;IPI00705159;IPI00705237;IPI00706142;IPI00707685;IPI00707776;IPI00707857;IPI00709876;IPI00710274;IPI00712263;IPI00712384;IPI00713049;IPI00713597;IPI00714898;IPI00715059;IPI00716725;IPI00717764;IPI00847122;IPI00866858;IPI00866975;IPI00876805;IPI01003286	Biological Process	Regulation of primary metabolic process	5	33	246	0.9120

GO:0046483	IPI00685510;IPI00686872;IPI00687395;IPI00688337;IPI00688446;IPI00689365;IPI00689641;IPI00691093;IPI00694430;IPI00696748;IPI00698213;IPI00698339;IPI00699226;IPI00700228;IPI00701747;IPI00702948;IPI00703022;IPI00704332;IPI00704902;IPI00705104;IPI00705159;IPI00706142;IPI00708246;IPI00709124;IPI00709888;IPI00710377;IPI00712263;IPI00712399;IPI00712524;IPI00718678;IPI00867017;IPI01017508	Biological Process	Heterocycle metabolic process	5	32	246	0.9149
GO:0007275	IPI00685510;IPI00686595;IPI00687578;IPI00688704;IPI00691093;IPI00691826;IPI00693824;IPI00697648;IPI00699039;IPI00702768;IPI00703813;IPI00705583;IPI00707776;IPI00707857;IPI00708070;IPI00708244;IPI00709124;IPI00709876;IPI00709888;IPI00710274;IPI00710920;IPI00713049;IPI00713597;IPI00715059;IPI00716830;IPI00717764;IPI00731432;IPI00826022;IPI00839150;IPI00866975;IPI00876805;IPI01003286	Biological Process	Multicellular organismal development	5	32	246	0.6980

GO:0034645	IPI00687634;IPI00687662;IPI00688727;IPI00689365;IPI00689565;IPI00694444;IPI00694565;IPI00694688;IPI00698077;IPI00698213;IPI00698823;IPI00699039;IPI00699827;IPI00701747;IPI00702948;IPI00704902;IPI00705739;IPI00706142;IPI00707386;IPI00707537;IPI00708210;IPI00708246;IPI00709409;IPI00710859;IPI00712384;IPI00712524;IPI00715868;IPI00716860;IPI00717048;IPI00718379;IPI00867017	Biological Process	Cellular macromolecule biosynthetic process	6	31	246	0.0292
GO:0006139	IPI00685510;IPI00686872;IPI00687395;IPI00688337;IPI00688446;IPI00689365;IPI00689641;IPI00694430;IPI00696748;IPI00698213;IPI00698339;IPI00699226;IPI00700228;IPI00701747;IPI00702948;IPI00703022;IPI00704332;IPI00704902;IPI00705104;IPI00705159;IPI00706142;IPI00708246;IPI00709124;IPI00709888;IPI00710377;IPI00712263;IPI00712399;IPI00712524;IPI00718678;IPI00867017;IPI01017508	Biological Process	Nucleobase-containing compound metabolic process	6	31	246	0.8675

GO:0023052	IPI00685510;IPI00688704;IPI00688980;IPI00690309;IPI00690581;IPI00691826;IPI00693824;IPI00694198;IPI00695042;IPI00696116;IPI00697595;IPI00698059;IPI00698213;IPI00707776;IPI00708070;IPI00708244;IPI00709876;IPI00711457;IPI00713049;IPI00713474;IPI00713597;IPI00715059;IPI00717764;IPI00718152;IPI00727050;IPI00731432;IPI00829520;IPI00847122;IPI00876805;IPI00908033;IPI01017588	Biological Process	Signaling	3	31	246	0.3860
GO:0044700	IPI00685510;IPI00688704;IPI00688980;IPI00690309;IPI00690581;IPI00691826;IPI00693824;IPI00694198;IPI00695042;IPI00696116;IPI00697595;IPI00698059;IPI00698213;IPI00707776;IPI00708070;IPI00708244;IPI00709876;IPI00711457;IPI00713049;IPI00713474;IPI00713597;IPI00715059;IPI00717764;IPI00718152;IPI00727050;IPI00731432;IPI00829520;IPI00847122;IPI00876805;IPI00908033;IPI01017588	Biological Process	Single organism signaling	4	31	246	0.3860

GO:0051641	IPI00685510;IPI00690581;IPI00692417;IPI00692963;IPI00695852;IPI00696116;IPI00698213;IPI00698823;IPI00700228;IPI00703084;IPI00703954;IPI00707815;IPI00707857;IPI00708037;IPI00708162;IPI00709888;IPI00711457;IPI00713460;IPI00713474;IPI00714269;IPI00714660;IPI00715059;IPI00716830;IPI00717764;IPI00718152;IPI00730219;IPI00866975;IPI00876805;IPI01003286;IPI01028455	Biological Process	Cellular localization	4	30	246	0.2438
GO:0044767	IPI00685510;IPI00687578;IPI00688704;IPI00690309;IPI00691093;IPI00691826;IPI00697648;IPI00699039;IPI00702768;IPI00703813;IPI00705583;IPI00707776;IPI00707857;IPI00708070;IPI00708244;IPI00709124;IPI00709876;IPI00710274;IPI00710920;IPI00713049;IPI00715059;IPI00716830;IPI00717764;IPI00731432;IPI00826022;IPI00839150;IPI00866975;IPI00876805;IPI00924039;IPI01003286	Biological Process	Single-organism developmental process	4	30	246	0.6864

GO:0033036	IPI00685617;IPI00689226;IPI00690581;IPI00691826;IPI00692417;IPI00692963;IPI00693824;IPI00694198;IPI00694597;IPI00696116;IPI00698823;IPI00700228;IPI00703084;IPI00703954;IPI00705933;IPI00707815;IPI00707857;IPI00708037;IPI00709876;IPI00713460;IPI00714269;IPI00714660;IPI00715059;IPI00716830;IPI00717764;IPI00718152;IPI00727050;IPI00829520;IPI00867311	Biological Process	Macromolecule localization	4	29	246	0.4317
GO:0048519	IPI00685510;IPI00690309;IPI00690581;IPI00693579;IPI00694688;IPI00695042;IPI00698213;IPI00698823;IPI00703084;IPI00705159;IPI00705441;IPI00706314;IPI00707857;IPI00708070;IPI00709876;IPI00710274;IPI00712263;IPI00712384;IPI00712524;IPI00712858;IPI00713049;IPI00715059;IPI00716725;IPI00731432;IPI00845178;IPI00847122;IPI00866975;IPI01003286;IPI01028455	Biological Process	Negative regulation of biological process	5	29	246	0.8926

GO:0006996	IPI00687578;IPI00688474;IPI00689226;IPI00689365;IPI00689379;IPI00689641;IPI00693579;IPI00694198;IPI00697648;IPI00698339;IPI00704902;IPI00705104;IPI00706314;IPI00707776;IPI00707815;IPI00708070;IPI00708162;IPI00708767;IPI00709876;IPI00709888;IPI00710274;IPI00712288;IPI00712399;IPI00713474;IPI00718678;IPI00727591;IPI00843305;IPI00866975;IPI00924039	Biological Process	Organelle organization	4	29	246	0.4548
GO:0048522	IPI00685510;IPI00685617;IPI00690309;IPI00690789;IPI00694430;IPI00694565;IPI00695037;IPI00697917;IPI00698213;IPI00703084;IPI00704902;IPI00705237;IPI00705583;IPI00707776;IPI00707857;IPI00709124;IPI00709876;IPI00709888;IPI00710274;IPI00712858;IPI00713597;IPI00714898;IPI00715059;IPI00716725;IPI00716830;IPI00717764;IPI00847122;IPI00852533;IPI00866975	Biological Process	Positive regulation of cellular process	4	29	246	0.7139

GO:0060255	IPI00685510;IPI00685617;IPI00690309;IPI00690581;IPI00693842;IPI00694688;IPI00695037;IPI00696748;IPI00698213;IPI00698339;IPI00704902;IPI00705237;IPI00706142;IPI00707685;IPI00707776;IPI00707857;IPI00709876;IPI00710274;IPI00712263;IPI00712384;IPI00713597;IPI00714898;IPI00715059;IPI00716725;IPI00717764;IPI00847122;IPI00866975;IPI00876805;IPI01003286	Biological Process	Regulation of macromolecule metabolic process	6	29	246	0.9615
GO:0071702	IPI00689226;IPI00690581;IPI00691086;IPI00691826;IPI00692417;IPI00692963;IPI00693824;IPI00694198;IPI00694597;IPI00696116;IPI00698823;IPI00700228;IPI00703084;IPI00703954;IPI00705933;IPI00707815;IPI00707857;IPI00708037;IPI00708597;IPI00713460;IPI00714269;IPI00714656;IPI00714660;IPI00717764;IPI00718152;IPI00727050;IPI00829520;IPI00867311	Biological Process	Organic substance transport	6	28	246	0.2104

GO:0048731	IPI00685510;IPI00687578;IPI00691093;IPI00691826;IPI00693824;IPI00697648;IPI00699039;IPI00702768;IPI00705583;IPI00707776;IPI00707857;IPI00708070;IPI00708244;IPI00709876;IPI00709888;IPI00710274;IPI00710920;IPI00713049;IPI00713597;IPI00715059;IPI00716830;IPI00717764;IPI00731432;IPI00826022;IPI00839150;IPI00866975;IPI00876805;IPI01003286	Biological Process	System development	6	28	246	0.5999
GO:0042221	IPI00685510;IPI00690309;IPI00690581;IPI00696116;IPI00697595;IPI00698823;IPI00702768;IPI00703084;IPI00703813;IPI00705159;IPI00707455;IPI00707815;IPI00707857;IPI00708244;IPI00709124;IPI00709876;IPI00712524;IPI00712596;IPI00715059;IPI00716157;IPI00717764;IPI00730219;IPI00731432;IPI00760457;IPI00826022;IPI00866858;IPI01028182	Biological Process	Response to chemical	4	27	246	0.1804

GO:0007165	IPI00685510;IPI00688704;IPI00688980;IPI00690309;IPI00690581;IPI00691826;IPI00693824;IPI00694198;IPI00695042;IPI00697595;IPI00698213;IPI00707776;IPI00708244;IPI00709876;IPI00711457;IPI00713049;IPI00713474;IPI00713597;IPI00715059;IPI00717764;IPI00718152;IPI00727050;IPI00731432;IPI00829520;IPI00847122;IPI00908033;IPI01017588	Biological Process	Signal transduction	6	27	246	0.4267
GO:0008104	IPI00685617;IPI00689226;IPI00690581;IPI00691826;IPI00692417;IPI00692963;IPI00693824;IPI00694198;IPI00696116;IPI00703084;IPI00703954;IPI00705933;IPI00707815;IPI00707857;IPI00708037;IPI00709876;IPI00713460;IPI00714269;IPI00714660;IPI00715059;IPI00716830;IPI00717764;IPI00718152;IPI00727050;IPI00829520;IPI00867311	Biological Process	Protein localization	5	26	246	0.3789

GO:0048583	IPI00685510;IPI00685617;IPI00688451;IPI00690309;IPI00690581;IPI00692668;IPI00697917;IPI00698823;IPI00705237;IPI00707776;IPI00707857;IPI00709876;IPI00709888;IPI00710274;IPI00712858;IPI00713049;IPI00713474;IPI00713597;IPI00715059;IPI00716830;IPI00717764;IPI00731432;IPI00845178;IPI00852533;IPI00866858;IPI01000210	Biological Process	Regulation of response to stimulus	4	26	246	0.5540
GO:0051649	IPI00685510;IPI00690581;IPI00692417;IPI00692963;IPI00695852;IPI00696116;IPI00698213;IPI00698823;IPI00700228;IPI00703084;IPI00703954;IPI00707815;IPI00708037;IPI00708162;IPI00709888;IPI00711457;IPI00713460;IPI00713474;IPI00714269;IPI00714660;IPI00717764;IPI00718152;IPI00730219;IPI00866975;IPI01003286	Biological Process	Establishment of localization in cell	5	25	246	0.3245

GO:0048523	IPI00685510;IPI00690309;IPI00690581;IPI00693579;IPI00694688;IPI00695042;IPI00698213;IPI00698823;IPI00703084;IPI00705159;IPI00705441;IPI00706314;IPI00707857;IPI00708070;IPI00709876;IPI00710274;IPI00712263;IPI00712384;IPI00712858;IPI00713049;IPI00715059;IPI00845178;IPI00847122;IPI01003286;IPI01028455	Biological Process	Negative regulation of cellular process	4	25	246	0.9348
GO:0044281	IPI00685510;IPI00686872;IPI00689365;IPI00690789;IPI00694430;IPI00695952;IPI00697194;IPI00698823;IPI00699039;IPI00699226;IPI00703084;IPI00704332;IPI00705159;IPI00705565;IPI00708246;IPI00709888;IPI00710377;IPI00714621;IPI00715066;IPI00717758;IPI00760457;IPI00839150;IPI00867017;IPI00871114;IPI01028182	Biological Process	Small molecule metabolic process	5	25	246	0.8475

GO:0090304	IPI00685510;IPI00687395;IPI00688337;IPI00688446;IPI00689365;IPI00689641;IPI00696748;IPI00698213;IPI00698339;IPI00700228;IPI00701747;IPI00702948;IPI00703022;IPI00704902;IPI00705104;IPI00706142;IPI00708246;IPI00709124;IPI00712263;IPI00712399;IPI00712524;IPI00718678;IPI00867017;IPI01017508	Biological Process	Nucleic acid metabolic process	7	24	246	0.7310
GO:0010646	IPI00685510;IPI00685617;IPI00688451;IPI00690309;IPI00692668;IPI00697917;IPI00698823;IPI00705237;IPI00707776;IPI00707857;IPI00709876;IPI00709888;IPI00710274;IPI00712858;IPI00713049;IPI00713474;IPI00713597;IPI00715059;IPI00716830;IPI00717764;IPI00845178;IPI00852533;IPI00866858;IPI01000210	Biological Process	Regulation of cell communication	6	24	246	0.3409

GO:0006412	IPI00687634;IPI00687662;IPI00688727;IPI00689365;IPI00689565;IPI00694444;IPI00694565;IPI00694688;IPI00698077;IPI00699827;IPI00702948;IPI00705739;IPI00707386;IPI00707537;IPI00708210;IPI00708246;IPI00709409;IPI00710859;IPI00712384;IPI00715868;IPI00716860;IPI00717048;IPI00718379;IPI00867017	Biological Process	Translation	7	24	246	5.32E-05
GO:0048513	IPI00685510;IPI00687578;IPI00691093;IPI00691826;IPI00697648;IPI00702768;IPI00705583;IPI00707776;IPI00707857;IPI00708070;IPI00708244;IPI00709876;IPI00709888;IPI00710274;IPI00710920;IPI00715059;IPI00717764;IPI00731432;IPI00826022;IPI00839150;IPI00866975;IPI00876805;IPI01003286	Biological Process	Organ development	7	23	246	0.2970

GO:1901564	IPI00685804;IPI00686872;IPI00689365;IPI00691093;IPI00694430;IPI00698059;IPI00699039;IPI00699226;IPI00704332;IPI00705159;IPI00705565;IPI00708246;IPI00709888;IPI00710377;IPI00715066;IPI00717764;IPI00717930;IPI00760457;IPI00839150;IPI00867017;IPI00871114;IPI00876805;IPI01028182	Biological Process	Organonitrogen compound metabolic process	5	23	246	0.4925
GO:0065009	IPI00685510;IPI00685617;IPI00685678;IPI00687375;IPI00688451;IPI00690309;IPI00692668;IPI00696748;IPI00698916;IPI00703925;IPI00705237;IPI00706142;IPI00707776;IPI00709876;IPI00710274;IPI00713049;IPI00713597;IPI00715059;IPI00717764;IPI00847122;IPI00866858;IPI00867230;IPI01000210	Biological Process	Regulation of molecular function	4	23	246	0.2455

GO:0051171	IPI00685510;IPI00688451;IPI00690309;IPI00690581;IPI00692668;IPI00693842;IPI00694688;IPI00695037;IPI00698213;IPI00704902;IPI00705237;IPI00706142;IPI00707776;IPI00707857;IPI00709876;IPI00710274;IPI00712263;IPI00713049;IPI00714898;IPI00866858;IPI00866975;IPI00876805;IPI01003286	Biological Process	Regulation of nitrogen compound metabolic process	5	23	246	0.8603
GO:0023051	IPI00685510;IPI00685617;IPI00688451;IPI00690309;IPI00692668;IPI00697917;IPI00705237;IPI00707776;IPI00707857;IPI00709876;IPI00709888;IPI00710274;IPI00712858;IPI00713049;IPI00713474;IPI00713597;IPI00715059;IPI00716830;IPI00717764;IPI00845178;IPI00852533;IPI00866858;IPI01000210	Biological Process	Regulation of signaling	4	23	246	0.4024

GO:0044085	IPI00685510;IPI00687578;IPI00688474;IPI00689226;IPI00689365;IPI00689641;IPI00689899;IPI00697648;IPI00698339;IPI00702948;IPI00704902;IPI00705104;IPI00705237;IPI00707857;IPI00708070;IPI00708244;IPI00708767;IPI00709876;IPI00710834;IPI00711457;IPI00712399;IPI00866975	Biological Process	Cellular component biogenesis	4	22	246	0.4941
GO:0045184	IPI00689226;IPI00690581;IPI00691826;IPI00692417;IPI00692963;IPI00693824;IPI00694198;IPI00696116;IPI00703084;IPI00703954;IPI00705933;IPI00707815;IPI00707857;IPI00708037;IPI00713460;IPI00714269;IPI00714660;IPI00717764;IPI00718152;IPI00727050;IPI00829520;IPI00867311	Biological Process	Establishment of protein localization	6	22	246	0.3067
GO:0015031	IPI00689226;IPI00690581;IPI00691826;IPI00692417;IPI00692963;IPI00693824;IPI00694198;IPI00696116;IPI00703084;IPI00703954;IPI00705933;IPI00707815;IPI00707857;IPI00708037;IPI00713460;IPI00714269;IPI00714660;IPI00717764;IPI00718152;IPI00727050;IPI00829520;IPI00867311	Biological Process	Protein transport	6	22	246	0.2945

GO:0065008	IPI00685510;IPI00687478;IPI00690309;IPI00690581;IPI00696116;IPI00698059;IPI00698823;IPI00702948;IPI00703084;IPI00703813;IPI00705159;IPI00708070;IPI00708244;IPI00709876;IPI00709888;IPI00710664;IPI00711457;IPI00713313;IPI00715066;IPI00867237;IPI00876805;IPI01028455	Biological Process	Regulation of biological quality	4	22	246	0.8831
GO:0050790	IPI00685510;IPI00685617;IPI00685678;IPI00687375;IPI00688451;IPI00690309;IPI00692668;IPI00696748;IPI00698916;IPI00703925;IPI00705237;IPI00706142;IPI00709876;IPI00710274;IPI00713049;IPI00713597;IPI00715059;IPI00717764;IPI00847122;IPI00866858;IPI00867230;IPI01000210	Biological Process	Regulation of catalytic activity	5	22	246	0.0973
GO:0032879	IPI00690309;IPI00693452;IPI00694198;IPI00695042;IPI00696116;IPI00698916;IPI00703084;IPI00705159;IPI00705441;IPI00707857;IPI00708070;IPI00709876;IPI00712858;IPI00714269;IPI00715059;IPI00716725;IPI00845178;IPI00847122;IPI00866858;IPI00866975;IPI00906031;IPI01028468	Biological Process	Regulation of localization	4	22	246	0.1226

GO:0019219	IPI00685510;IPI00688451;IPI00690309;IPI00692668;IPI00693842;IPI00694688;IPI00695037;IPI00698213;IPI00704902;IPI00705237;IPI00706142;IPI00707776;IPI00707857;IPI00709876;IPI00710274;IPI00712263;IPI00713049;IPI00714898;IPI00866858;IPI00866975;IPI00876805;IPI01003286	Biological Process	Regulation of nucleobase-containing compound metabolic process	7	22	246	0.8831
GO:0009966	IPI00685510;IPI00685617;IPI00688451;IPI00690309;IPI00692668;IPI00697917;IPI00705237;IPI00707776;IPI00707857;IPI00709876;IPI00709888;IPI00710274;IPI00712858;IPI00713049;IPI00713474;IPI00713597;IPI00716830;IPI00717764;IPI00845178;IPI00852533;IPI00866858;IPI01000210	Biological Process	Regulation of signal transduction	7	22	246	0.3633
GO:0006950	IPI00685510;IPI00685653;IPI00685678;IPI00690309;IPI00690581;IPI00696748;IPI00698213;IPI00700673;IPI00703084;IPI00705565;IPI00707455;IPI00709876;IPI00710664;IPI00712399;IPI00714656;IPI00716157;IPI00717764;IPI00717930;IPI00718678;IPI00731432;IPI00843305;IPI01028455	Biological Process	Response to stress	4	22	246	0.7916

GO:0022607	IPI00685510;IPI00687578;IPI00688474;IPI00689226;IPI00689365;IPI00689641;IPI00689899;IPI00697648;IPI00698339;IPI00704902;IPI00705104;IPI00705237;IPI00707857;IPI00708070;IPI00708244;IPI00708767;IPI00709876;IPI00710834;IPI00711457;IPI00712399;IPI00866975	Biological Process	Cellular component assembly	5	21	246	0.3951
GO:0046907	IPI00685510;IPI00690581;IPI00692963;IPI00695852;IPI00696116;IPI00698213;IPI00698823;IPI00700228;IPI00703954;IPI00707815;IPI00708037;IPI00708162;IPI00709888;IPI00711457;IPI00713460;IPI00713474;IPI00714269;IPI00714660;IPI00730219;IPI00866975;IPI01003286	Biological Process	Intracellular transport	6	21	246	0.3819
GO:0030154	IPI00685510;IPI00687578;IPI00688704;IPI00690309;IPI00693824;IPI00696748;IPI00697648;IPI00705583;IPI00706142;IPI00707857;IPI00709876;IPI00710274;IPI00713049;IPI00713313;IPI00713597;IPI00716830;IPI00866975;IPI00876805;IPI00924039;IPI01003286	Biological Process	Cell differentiation	6	20	246	0.7422

GO:0048869	IPI00685510;IPI00687578;IPI00688704;IPI00690309;IPI00693824;IPI00696748;IPI00697648;IPI00705583;IPI00706142;IPI00707857;IPI00709876;IPI00710274;IPI00713049;IPI00713313;IPI00713597;IPI00716830;IPI00866975;IPI00876805;IPI00924039;IPI01003286	Biological Process	Cellular developmental process	5	20	246	0.8461
GO:0009653	IPI00685510;IPI00687578;IPI00688704;IPI00697648;IPI00699039;IPI00702768;IPI00707776;IPI00707857;IPI00708070;IPI00708244;IPI00709876;IPI00710274;IPI00710920;IPI00713049;IPI00826022;IPI00839150;IPI00866975;IPI00876805;IPI00924039	Biological Process	Anatomical structure morphogenesis	4	19	246	0.3869
GO:0035556	IPI00688980;IPI00690309;IPI00691826;IPI00693824;IPI00694198;IPI00695042;IPI00698213;IPI00708244;IPI00709876;IPI00711457;IPI00713474;IPI00713597;IPI00715059;IPI00717764;IPI00718152;IPI00727050;IPI00829520;IPI00847122;IPI00908033	Biological Process	Intracellular signal transduction	7	19	246	0.0722

GO:0043933	IPI00687578;IPI00688474;IPI00689226;IPI00689365;IPI00689641;IPI00689899;IPI00697648;IPI00698339;IPI00704902;IPI00705104;IPI00707857;IPI00708070;IPI00708244;IPI00708767;IPI00709876;IPI00710834;IPI00711457;IPI00712399;IPI00866975	Biological Process	Macromolecular complex subunit organization	5	19	246	0.2985
GO:0010468	IPI00685510;IPI00690309;IPI00694688;IPI00695037;IPI00698213;IPI00698339;IPI00704902;IPI00705237;IPI00706142;IPI00707776;IPI00707857;IPI00709876;IPI00712263;IPI00712384;IPI00714898;IPI00715059;IPI00866975;IPI00876805;IPI01003286	Biological Process	Regulation of gene expression	7	19	246	0.9759
GO:0010033	IPI00690309;IPI00690581;IPI00696116;IPI00698823;IPI00702768;IPI00703084;IPI00703813;IPI00707857;IPI00708244;IPI00709124;IPI00709876;IPI00712524;IPI00712596;IPI00715059;IPI00717764;IPI00731432;IPI00760457;IPI00826022;IPI01028182	Biological Process	Response to organic substance	5	19	246	0.1642

GO:0070887	IPI00690309;IPI00690581;IPI00696116;IPI00697595;IPI00698823;IPI00702768;IPI00707455;IPI00707815;IPI00707857;IPI00708244;IPI00709876;IPI00712524;IPI00715059;IPI00716157;IPI00717764;IPI00731432;IPI00826022;IPI00866858	Biological Process	Cellular response to chemical stimulus	5	18	246	0.1341
GO:0071822	IPI00687578;IPI00688474;IPI00689226;IPI00689365;IPI00689641;IPI00689899;IPI00697648;IPI00698339;IPI00705104;IPI00707857;IPI00708070;IPI00708244;IPI00708767;IPI00709876;IPI00710834;IPI00711457;IPI00712399;IPI00866975	Biological Process	Protein complex subunit organization	6	18	246	0.1791
GO:0009889	IPI00685510;IPI00690309;IPI00690581;IPI00695037;IPI00698213;IPI00704902;IPI00705159;IPI00706142;IPI00707776;IPI00707857;IPI00709876;IPI00710274;IPI00712384;IPI00714898;IPI00715059;IPI00866975;IPI00876805;IPI01003286	Biological Process	Regulation of biosynthetic process	5	18	246	0.9621

GO:0019220	IPI00685510;IPI00685617;IPI00688451;IPI00690309;IPI00692668;IPI00696748;IPI00698916;IPI00703925;IPI00705237;IPI00709876;IPI00710274;IPI00713049;IPI00713597;IPI00715059;IPI00717764;IPI00847122;IPI00866858;IPI01000210	Biological Process	Regulation of phosphate metabolic process	7	18	246	0.1208
GO:0051174	IPI00685510;IPI00685617;IPI00688451;IPI00690309;IPI00692668;IPI00696748;IPI00698916;IPI00703925;IPI00705237;IPI00709876;IPI00710274;IPI00713049;IPI00713597;IPI00715059;IPI00717764;IPI00847122;IPI00866858;IPI01000210	Biological Process	Regulation of phosphorus metabolic process	6	18	246	0.1208
GO:0031326	IPI00685510;IPI00690309;IPI00695037;IPI00698213;IPI00704902;IPI00705159;IPI00706142;IPI00707776;IPI00707857;IPI00709876;IPI00710274;IPI00712384;IPI00714898;IPI00715059;IPI00866975;IPI00876805;IPI01003286	Biological Process	Regulation of cellular biosynthetic process	6	17	246	0.9705

GO:0010556	IPI00685510;IPI00690309;IPI00690581;IPI00695037;IPI00698213;IPI00704902;IPI00706142;IPI00707776;IPI00707857;IPI00709876;IPI00710274;IPI00712384;IPI00714898;IPI00715059;IPI00866975;IPI00876805;IPI01003286	Biological Process	Regulation of macromolecule biosynthetic process	7	17	246	0.9524
GO:0016192	IPI00685510;IPI00692417;IPI00692963;IPI00693824;IPI00695852;IPI00696116;IPI00698823;IPI00706037;IPI00708037;IPI00708162;IPI00709876;IPI00713460;IPI00713474;IPI00714660;IPI00843305;IPI00866975;IPI01003286	Biological Process	Vesicle-mediated transport	6	17	246	0.3337
GO:0065003	IPI00688474;IPI00689226;IPI00689365;IPI00689641;IPI00689899;IPI00698339;IPI00704902;IPI00705104;IPI00707857;IPI00708244;IPI00708767;IPI00709876;IPI00710834;IPI00711457;IPI00712399;IPI00866975	Biological Process	Macromolecular complex assembly	6	16	246	0.2073
GO:0006793	IPI00685510;IPI00685617;IPI00686872;IPI00687661;IPI00690309;IPI00694430;IPI00699226;IPI00703084;IPI00704332;IPI00705159;IPI00706879;IPI00709888;IPI00710377;IPI00839150;IPI00871114;IPI00906031	Biological Process	Phosphorus metabolic process	5	16	246	0.9220

GO:0009893	IPI00685510;IPI00685617;IPI00690309;IPI00694430;IPI00695037;IPI00704902;IPI00705237;IPI00707857;IPI00709876;IPI00713597;IPI00714898;IPI00715059;IPI00716725;IPI00717764;IPI00847122;IPI00866975	Biological Process	Positive regulation of metabolic process	4	16	246	0.7355
GO:2000112	IPI00685510;IPI00690309;IPI00695037;IPI00698213;IPI00704902;IPI00706142;IPI00707776;IPI00707857;IPI00709876;IPI00710274;IPI00712384;IPI00714898;IPI00715059;IPI00866975;IPI00876805;IPI01003286	Biological Process	Regulation of cellular macromolecule biosynthetic process	7	16	246	0.9604
GO:0016070	IPI00685510;IPI00687395;IPI00688337;IPI00688446;IPI00689365;IPI00698213;IPI00700228;IPI00702948;IPI00703022;IPI00704902;IPI00706142;IPI00708246;IPI00712263;IPI00712524;IPI00867017;IPI01017508	Biological Process	RNA metabolic process	8	16	246	0.8712
GO:0006629	IPI00685510;IPI00685617;IPI00687661;IPI00690789;IPI00694430;IPI00697194;IPI00698823;IPI00703084;IPI00705565;IPI00706879;IPI00709876;IPI00714621;IPI00715059;IPI00717758;IPI00839150	Biological Process	Lipid metabolic process	5	15	246	0.3816

GO:0006796	IPI00685510;IPI00685617;IPI00686872;IPI00687661;IPI00690309;IPI00694430;IPI00699226;IPI00703084;IPI00704332;IPI00705159;IPI00706879;IPI00709888;IPI00710377;IPI00839150;IPI00906031	Biological Process	Phosphate-containing compound metabolic process	6	15	246	0.9359
GO:0031325	IPI00685510;IPI00685617;IPI00690309;IPI00694430;IPI00695037;IPI00704902;IPI00707857;IPI00709876;IPI00713597;IPI00714898;IPI00715059;IPI00716725;IPI00717764;IPI00847122;IPI00866975	Biological Process	Positive regulation of cellular metabolic process	6	15	246	0.6945
GO:0010604	IPI00685510;IPI00685617;IPI00690309;IPI00695037;IPI00704902;IPI00705237;IPI00707857;IPI00709876;IPI00713597;IPI00714898;IPI00715059;IPI00716725;IPI00717764;IPI00847122;IPI00866975	Biological Process	Positive regulation of macromolecule metabolic process	6	15	246	0.6945
GO:0006461	IPI00688474;IPI00689226;IPI00689365;IPI00689641;IPI00689899;IPI00698339;IPI00705104;IPI00707857;IPI00708244;IPI00708767;IPI00709876;IPI00710834;IPI00711457;IPI00712399;IPI00866975	Biological Process	Protein complex assembly	7	15	246	0.1173

GO:0070271	IPI00688474;IPI00689226;IPI00689365;IPI00689641;IPI00689899;IPI00698339;IPI00705104;IPI00707857;IPI00708244;IPI00708767;IPI00709876;IPI00710834;IPI00711457;IPI00712399;IPI00866975	Biological Process	Protein complex biogenesis	5	15	246	0.1320
GO:0051239	IPI00685510;IPI00690309;IPI00690581;IPI00695042;IPI00703084;IPI00705159;IPI00706879;IPI00707776;IPI00707857;IPI00708070;IPI00709876;IPI00712524;IPI00713474;IPI00717764;IPI00845178	Biological Process	Regulation of multicellular organismal process	5	15	246	0.7597
GO:0051252	IPI00685510;IPI00690309;IPI00694688;IPI00695037;IPI00698213;IPI00704902;IPI00706142;IPI00707776;IPI00707857;IPI00709876;IPI00712263;IPI00714898;IPI00866975;IPI00876805;IPI01003286	Biological Process	Regulation of RNA metabolic process	9	15	246	0.9434
GO:0051049	IPI00690309;IPI00693452;IPI00694198;IPI00696116;IPI00698916;IPI00703084;IPI00705159;IPI00709876;IPI00712858;IPI00714269;IPI00715059;IPI00716725;IPI00866975;IPI00906031;IPI01028468	Biological Process	Regulation of transport	6	15	246	0.1587

GO:0009056	IPI00690581;IPI00694430;IPI00698059;IPI00698823;IPI00704041;IPI00704902;IPI00709124;IPI00710377;IPI00715059;IPI00715066;IPI00716157;IPI00717764;IPI00871114;IPI01017508	Biological Process	Catabolic process	4	14	246	0.9945
GO:0006082	IPI00689365;IPI00694430;IPI00698823;IPI00699039;IPI00703084;IPI00705565;IPI00708246;IPI00714621;IPI00715066;IPI00717758;IPI00760457;IPI00867017;IPI00871114;IPI01028182	Biological Process	Organic acid metabolic process	5	14	246	0.7017
GO:0043436	IPI00689365;IPI00694430;IPI00698823;IPI00699039;IPI00703084;IPI00705565;IPI00708246;IPI00714621;IPI00715066;IPI00717758;IPI00760457;IPI00867017;IPI00871114;IPI01028182	Biological Process	Oxoacid metabolic process	6	14	246	0.6819
GO:0009888	IPI00685510;IPI00696748;IPI00697648;IPI00699039;IPI00707857;IPI00708070;IPI00709876;IPI00709888;IPI00710274;IPI00710920;IPI00713049;IPI00713313;IPI00876805;IPI01003286	Biological Process	Tssue development	5	14	246	0.4451

GO:0019752	IPI00689365;IPI00694430;IPI00698823;IPI00703084;IPI00705565;IPI00708246;IPI00714621;IPI00715066;IPI00717758;IPI00760457;IPI00867017;IPI00871114;IPI01028182	Biological Process	Carboxylic acid metabolic process	7	13	246	0.7351
GO:0042592	IPI00685510;IPI00687478;IPI00690581;IPI00698823;IPI00702948;IPI00703084;IPI00703813;IPI00709876;IPI00709888;IPI00710664;IPI00711457;IPI00867237;IPI00876805	Biological Process	Homeostatic process	5	13	246	0.4283
GO:1901362	IPI00686872;IPI00690789;IPI00691093;IPI00697194;IPI00698213;IPI00699226;IPI00704332;IPI00704902;IPI00705159;IPI00706142;IPI00709888;IPI00710377;IPI00712524	Biological Process	Organic cyclic compound biosynthetic process	6	13	246	0.8027
GO:2001141	IPI00685510;IPI00690309;IPI00695037;IPI00698213;IPI00704902;IPI00706142;IPI00707776;IPI00707857;IPI00709876;IPI00714898;IPI00866975;IPI00876805;IPI01003286	Biological Process	Regulation of RNA biosynthetic process	9	13	246	0.9532
GO:0006355	IPI00685510;IPI00690309;IPI00695037;IPI00698213;IPI00704902;IPI00706142;IPI00707776;IPI00707857;IPI00709876;IPI00714898;IPI00866975;IPI00876805;IPI01003286	Biological Process	Regulation of transcription, DNA-templated	11	13	246	0.9516

GO:1901700	IPI00690309;IPI00690581;IPI00696116;IPI00702768;IPI00703084;IPI00707857;IPI00708244;IPI00709876;IPI00715059;IPI00716157;IPI00731432;IPI00760457;IPI00826022	Biological Process	Response to oxygen-containing compound	5	13	246	0.1092
GO:0044248	IPI00690581;IPI00694430;IPI00698059;IPI00698823;IPI00704041;IPI00704902;IPI00709124;IPI00710377;IPI00715066;IPI00716157;IPI00871114;IPI01017508	Biological Process	Cellular catabolic process	5	12	246	0.9815
GO:0044271	IPI00686872;IPI00691093;IPI00698213;IPI00699226;IPI00704332;IPI00704902;IPI00705159;IPI00706142;IPI00709888;IPI00710377;IPI00712524;IPI00876805	Biological Process	Cellular nitrogen compound biosynthetic process	6	12	246	0.8578
GO:0006464	IPI00685510;IPI00689629;IPI00690309;IPI00698823;IPI00698916;IPI00706142;IPI00707776;IPI00718678;IPI00727591;IPI00731432;IPI00825680;IPI00906031	Biological Process	Cellular protein modification process	7	12	246	0.9675
GO:0006811	IPI00686872;IPI00693452;IPI00699372;IPI00703813;IPI00708597;IPI00709876;IPI00709888;IPI00710377;IPI00710664;IPI00710920;IPI00711457;IPI00714656	Biological Process	Ion transport	5	12	246	0.0537

GO:0043412	IPI00685510;IPI00689629;IPI00690309;IPI00698823;IPI00698916;IPI00706142;IPI00707776;IPI00718678;IPI00727591;IPI00731432;IPI00825680;IPI00906031	Biological Process	Macromolecule modification	6	12	246	0.9822
GO:0009892	IPI00690309;IPI00690581;IPI00694688;IPI00698213;IPI00698823;IPI00705159;IPI00709876;IPI00710274;IPI00712263;IPI00712384;IPI00713049;IPI01003286	Biological Process	Negative regulation of metabolic process	4	12	246	0.8361
GO:1901575	IPI00690581;IPI00694430;IPI00698059;IPI00704041;IPI00704902;IPI00709124;IPI00710377;IPI00715059;IPI00715066;IPI00717764;IPI00871114;IPI01017508	Biological Process	Organic substance catabolic process	5	12	246	0.9963
GO:0019637	IPI00685617;IPI00686872;IPI00687661;IPI00694430;IPI00699226;IPI00703084;IPI00704332;IPI00705159;IPI00706879;IPI00709888;IPI00710377;IPI00839150	Biological Process	Organophosphate metabolic process	6	12	246	0.7741
GO:0036211	IPI00685510;IPI00689629;IPI00690309;IPI00698823;IPI00698916;IPI00706142;IPI00707776;IPI00718678;IPI00727591;IPI00731432;IPI00825680;IPI00906031	Biological Process	Protein modification process	6	12	246	0.9675

GO:0051246	IPI00685510;IPI00685617;IPI00690309;IPI00696748;IPI00707685;IPI00709876;IPI00710274;IPI00712384;IPI00713597;IPI00716725;IPI00717764;IPI00847122	Biological Process	Regulation of protein metabolic process	6	12	246	0.8945
GO:0009719	IPI00690309;IPI00696116;IPI00698823;IPI00703813;IPI00707857;IPI00708244;IPI00709876;IPI00712524;IPI00715059;IPI00731432;IPI00826022;IPI01028182	Biological Process	Response to endogenous stimulus	4	12	246	0.0388
GO:0009605	IPI00685510;IPI00690309;IPI00697595;IPI00698213;IPI00702768;IPI00707857;IPI00709876;IPI00710920;IPI00714656;IPI00717764;IPI00866858;IPI01028455	Biological Process	Response to external stimulus	4	12	246	0.0154
GO:0019438	IPI00686872;IPI00691093;IPI00698213;IPI00699226;IPI00704332;IPI00704902;IPI00705159;IPI00706142;IPI00709888;IPI00710377;IPI00712524	Biological Process	Aromatic compound biosynthetic process	6	11	246	0.8270
GO:0022610	IPI00685769;IPI00688400;IPI00697595;IPI00708070;IPI00708824;IPI00709888;IPI00710204;IPI00713313;IPI00713979;IPI00731432;IPI00924039	Biological Process	Biological adhesion	3	11	246	0.2237

GO:1901135	IPI00686872;IPI00694430;IPI00698823;IPI00699039;IPI00699226;IPI00704332;IPI00705159;IPI00709888;IPI00710377;IPI00717764;IPI00717930	Biological Process	Carbohydrate derivative metabolic process	5	11	246	0.8169
GO:0007155	IPI00685769;IPI00688400;IPI00697595;IPI00708070;IPI00708824;IPI00709888;IPI00710204;IPI00713313;IPI00713979;IPI00731432;IPI00924039	Biological Process	Cell adhesion	4	11	246	0.2237
GO:0044255	IPI00685617;IPI00687661;IPI00690789;IPI00694430;IPI00703084;IPI00705565;IPI00706879;IPI00709876;IPI00714621;IPI00717758;IPI00839150	Biological Process	Cellular lipid metabolic process	5	11	246	0.5188
GO:0070727	IPI00690581;IPI00692963;IPI00696116;IPI00703954;IPI00707815;IPI00707857;IPI00708037;IPI00714269;IPI00714660;IPI00715059;IPI00716830	Biological Process	Cellular macromolecule localization	5	11	246	0.9029
GO:0034613	IPI00690581;IPI00692963;IPI00696116;IPI00703954;IPI00707815;IPI00707857;IPI00708037;IPI00714269;IPI00714660;IPI00715059;IPI00716830	Biological Process	Cellular protein localization	6	11	246	0.8997
GO:0071310	IPI00690581;IPI00696116;IPI00698823;IPI00702768;IPI00707857;IPI00708244;IPI00712524;IPI00715059;IPI00717764;IPI00731432;IPI00826022	Biological Process	Cellular response to organic substance	6	11	246	0.3351

GO:0033554	IPI00690309;IPI00690581;IPI00696748;IPI00698213;IPI00707455;IPI00709876;IPI00712399;IPI00714656;IPI00716157;IPI00718678;IPI01028455	Biological Process	Cellular response to stress	5	11	246	0.5456
GO:0018130	IPI00686872;IPI00691093;IPI00698213;IPI00699226;IPI00704332;IPI00704902;IPI00705159;IPI00706142;IPI00709888;IPI00710377;IPI00712524	Biological Process	Heterocycle biosynthetic process	6	11	246	0.8632
GO:0031324	IPI00690309;IPI00694688;IPI00698213;IPI00698823;IPI00705159;IPI00709876;IPI00710274;IPI00712263;IPI00712384;IPI00713049;IPI01003286	Biological Process	Negative regulation of cellular metabolic process	6	11	246	0.8064
GO:0042127	IPI00685617;IPI00690789;IPI00693579;IPI00694565;IPI00703084;IPI00707776;IPI00709876;IPI00710274;IPI00712858;IPI00715059;IPI00845178	Biological Process	Regulation of cell proliferation	5	11	246	0.5456
GO:0051128	IPI00689379;IPI00690309;IPI00693842;IPI00694198;IPI00707857;IPI00710274;IPI00712384;IPI00712858;IPI00716725;IPI00866858;IPI00866975	Biological Process	Regulation of cellular component organization	5	11	246	0.9433
GO:0032268	IPI00685510;IPI00685617;IPI00690309;IPI00696748;IPI00709876;IPI00710274;IPI00712384;IPI00713597;IPI00716725;IPI00717764;IPI00847122	Biological Process	Regulation of cellular protein metabolic process	7	11	246	0.7841

GO:0050793	IPI00685510;IPI00690309;IPI00695042;IPI00705441;IPI00707776;IPI00707857;IPI00708070;IPI00709876;IPI00712263;IPI00712524;IPI00717764	Biological Process	Regulation of developmental process	4	11	246	0.8413
GO:0007264	IPI00688980;IPI00691826;IPI00693824;IPI00694198;IPI00695042;IPI00708244;IPI00718152;IPI00727050;IPI00829520;IPI00847122;IPI00908033	Biological Process	Small GTPase mediated signal transduction	8	11	246	0.0221
GO:0006812	IPI00686872;IPI00693452;IPI00699372;IPI00703813;IPI00709876;IPI00709888;IPI00710377;IPI00710664;IPI00710920;IPI00711457	Biological Process	Cation transport	6	10	246	0.0402
GO:0019725	IPI00685510;IPI00687478;IPI00690581;IPI00703813;IPI00709876;IPI00709888;IPI00710664;IPI00711457;IPI00867237;IPI00876805	Biological Process	Cellular homeostasis	5	10	246	0.1517
GO:0034622	IPI00688474;IPI00689226;IPI00689365;IPI00689641;IPI00698339;IPI00704902;IPI00705104;IPI00708767;IPI00712399;IPI00866975	Biological Process	Cellular macromolecular complex assembly	7	10	246	0.1588
GO:0044092	IPI00685678;IPI00687375;IPI00698916;IPI00703925;IPI00706142;IPI00707776;IPI00709876;IPI00710274;IPI00867230;IPI01000210	Biological Process	Negative regulation of molecular function	5	10	246	0.2130

GO:0007399	IPI00685510;IPI00687578;IPI00691826;IPI00693824;IPI00710274;IPI00713597;IPI00716830;IPI00866975;IPI00876805;IPI01003286	Biological Process	Nervous system development	7	10	246	0.8172
GO:0034654	IPI00686872;IPI00698213;IPI00699226;IPI00704332;IPI00704902;IPI00705159;IPI00706142;IPI00709888;IPI00710377;IPI00712524	Biological Process	Nucleobase-containing compound biosynthetic process	7	10	246	0.8327
GO:1901566	IPI00686872;IPI00691093;IPI00699226;IPI00704332;IPI00705159;IPI00709888;IPI00710377;IPI00715066;IPI00760457;IPI00876805	Biological Process	Organonitrogen compound biosynthetic process	6	10	246	0.4552
GO:0090407	IPI00685617;IPI00686872;IPI00687661;IPI00699226;IPI00703084;IPI00704332;IPI00705159;IPI00706879;IPI00709888;IPI00710377	Biological Process	Organophosphate biosynthetic process	6	10	246	0.2213
GO:0048584	IPI00685617;IPI00697917;IPI00707857;IPI00709876;IPI00709888;IPI00713597;IPI00715059;IPI00716830;IPI00717764;IPI00852533	Biological Process	Positive regulation of response to stimulus	5	10	246	0.8007
GO:0060341	IPI00690309;IPI00696116;IPI00703084;IPI00709876;IPI00712858;IPI00714269;IPI00715059;IPI00866858;IPI00906031;IPI01028468	Biological Process	Regulation of cellular localization	5	10	246	0.1968

GO:0051336	IPI00688451;IPI00690309;IPI00692668;IPI00698916;IPI00703925;IPI00705237;IPI00713049;IPI00866858;IPI00867230;IPI01000210	Biological Process	Regulation of hydrolase activity	6	10	246	0.4649
GO:0043549	IPI00685510;IPI00685617;IPI00690309;IPI00696748;IPI00709876;IPI00710274;IPI00713597;IPI00715059;IPI00717764;IPI00847122	Biological Process	Regulation of kinase activity	9	10	246	0.0662
GO:0042325	IPI00685510;IPI00685617;IPI00690309;IPI00696748;IPI00709876;IPI00710274;IPI00713597;IPI00715059;IPI00717764;IPI00847122	Biological Process	Regulation of phosphorylation	8	10	246	0.2470
GO:0051338	IPI00685510;IPI00685617;IPI00690309;IPI00696748;IPI00709876;IPI00710274;IPI00713597;IPI00715059;IPI00717764;IPI00847122	Biological Process	Regulation of transferase activity	6	10	246	0.0850
GO:0048646	IPI00687578;IPI00688704;IPI00697648;IPI00699039;IPI00707857;IPI00709876;IPI00710274;IPI00713049;IPI00839150	Biological Process	Anatomical structure formation involved in morphogenesis	5	9	246	0.3025
GO:0048878	IPI00685510;IPI00698823;IPI00702948;IPI00703084;IPI00703813;IPI00709876;IPI00709888;IPI00710664;IPI00711457	Biological Process	Chemical homeostasis	6	9	246	0.2358
GO:0016482	IPI00692963;IPI00695852;IPI00696116;IPI00698213;IPI00700228;IPI00707815;IPI00713460;IPI00713474;IPI00714269	Biological Process	Cytoplasmic transport	7	9	246	0.6026

GO:0007010	IPI00687578;IPI00689379;IPI00697648;IPI00708070;IPI00708767;IPI00709888;IPI00710274;IPI00712288;IPI00924039	Biological Process	Cytoskeleton organization	5	9	246	0.8010
GO:0008610	IPI00685617;IPI00687661;IPI00690789;IPI00694430;IPI00697194;IPI00703084;IPI00705565;IPI00706879;IPI00714621	Biological Process	Lipid biosynthetic process	6	9	246	0.1167
GO:0043086	IPI00685678;IPI00687375;IPI00698916;IPI00703925;IPI00706142;IPI00709876;IPI00710274;IPI00867230;IPI01000210	Biological Process	Negative regulation of catalytic activity	5	9	246	0.1040
GO:0009887	IPI00687578;IPI00702768;IPI00707776;IPI00707857;IPI00708070;IPI00708244;IPI00710274;IPI00826022;IPI00839150	Biological Process	Organ morphogenesis	8	9	246	0.1040
GO:0010647	IPI00685617;IPI00697917;IPI00707857;IPI00709876;IPI00709888;IPI00713597;IPI00716830;IPI00717764;IPI00852533	Biological Process	Positive regulation of cell communication	7	9	246	0.5359
GO:0009967	IPI00685617;IPI00697917;IPI00707857;IPI00709876;IPI00709888;IPI00713597;IPI00716830;IPI00717764;IPI00852533	Biological Process	Positive regulation of signal transduction	8	9	246	0.4961
GO:0023056	IPI00685617;IPI00697917;IPI00707857;IPI00709876;IPI00709888;IPI00713597;IPI00716830;IPI00717764;IPI00852533	Biological Process	Positive regulation of signaling	5	9	246	0.5359

GO:0009894	IPI00688451;IPI00690309;IPI00692668;IPI00698823;IPI00705237;IPI00713049;IPI00715059;IPI00716725;IPI00866858	Biological Process	Regulation of catabolic process	5	9	246	0.4246
GO:0031329	IPI00688451;IPI00690309;IPI00692668;IPI00698823;IPI00705237;IPI00713049;IPI00715059;IPI00716725;IPI00866858	Biological Process	Regulation of cellular catabolic process	6	9	246	0.3224
GO:0045859	IPI00685510;IPI00685617;IPI00690309;IPI00696748;IPI00709876;IPI00710274;IPI00713597;IPI00717764;IPI00847122	Biological Process	Regulation of protein kinase activity	10	9	246	0.0811
GO:0031399	IPI00685510;IPI00685617;IPI00690309;IPI00696748;IPI00709876;IPI00710274;IPI00713597;IPI00717764;IPI00847122	Biological Process	Regulation of protein modification process	8	9	246	0.5840
GO:0001932	IPI00685510;IPI00685617;IPI00690309;IPI00696748;IPI00709876;IPI00710274;IPI00713597;IPI00717764;IPI00847122	Biological Process	Regulation of protein phosphorylation	9	9	246	0.2830
GO:0006396	IPI00685510;IPI00687395;IPI00688337;IPI00688446;IPI00700228;IPI00702948;IPI00703022;IPI00704902;IPI00712263	Biological Process	RNA processing	9	9	246	0.7630
GO:0003008	IPI00685510;IPI00696116;IPI00698059;IPI00705159;IPI00707857;IPI00708244;IPI00709876;IPI00710920;IPI00876805	Biological Process	System process	5	9	246	0.5746

GO:1901137	IPI00686872;IPI00698823;IPI00699039;IPI00699226;IPI00704332;IPI00705159;IPI00709888;IPI00710377	Biological Process	Carbohydrate derivative biosynthetic process	6	8	246	0.4772
GO:0008219	IPI00685510;IPI00687578;IPI00697349;IPI00698213;IPI00705378;IPI00706314;IPI00709124;IPI00716830	Biological Process	Cell death	5	8	246	0.8571
GO:0007166	IPI00685510;IPI00688704;IPI00697595;IPI00708244;IPI00709876;IPI00713597;IPI00715059;IPI00731432	Biological Process	Cell surface receptor signaling pathway	7	8	246	0.7761
GO:0006520	IPI00689365;IPI00705565;IPI00708246;IPI00715066;IPI00760457;IPI00867017;IPI00871114;IPI01028182	Biological Process	Cellular amino acid metabolic process	6	8	246	0.5716
GO:0071495	IPI00696116;IPI00698823;IPI00707857;IPI00708244;IPI00712524;IPI00715059;IPI00731432;IPI00826022	Biological Process	Cellular response to endogenous stimulus	5	8	246	0.0812
GO:1901701	IPI00690581;IPI00696116;IPI00707857;IPI00708244;IPI00715059;IPI00716157;IPI00731432;IPI00826022	Biological Process	Cellular response to oxygen-containing compound	6	8	246	0.2319
GO:0051276	IPI00689641;IPI00693579;IPI00698339;IPI00705104;IPI00707776;IPI00712399;IPI00718678;IPI00727591	Biological Process	Chromosome organization	5	8	246	0.2033
GO:0016265	IPI00685510;IPI00687578;IPI00697349;IPI00698213;IPI00705378;IPI00706314;IPI00709124;IPI00716830	Biological Process	Death	4	8	246	0.8619

GO:0006952	IPI00685653;IPI00700673;IPI00703084;IPI00705565;IPI00710664;IPI00716157;IPI00717764;IPI00717930	Biological Process	Defense response	5	8	246	0.3139
GO:0006259	IPI00689641;IPI00696748;IPI00698339;IPI00701747;IPI00705104;IPI00709124;IPI00712399;IPI00718678	Biological Process	DNA metabolic process	8	8	246	0.4008
GO:0060429	IPI00696748;IPI00699039;IPI00710274;IPI00710920;IPI00713049;IPI00713313;IPI00876805;IPI01003286	Biological Process	Epithelium development	6	8	246	0.5199
GO:0006886	IPI00690581;IPI00692963;IPI00696116;IPI00703954;IPI00707815;IPI00708037;IPI00714269;IPI00714660	Biological Process	Intracellular protein transport	7	8	246	0.9028
GO:0016071	IPI00685510;IPI00687395;IPI00688337;IPI00688446;IPI00700228;IPI00703022;IPI00704902;IPI00712263	Biological Process	mRNA metabolic process	9	8	246	0.5614
GO:0006397	IPI00685510;IPI00687395;IPI00688337;IPI00688446;IPI00700228;IPI00703022;IPI00704902;IPI00712263	Biological Process	mRNA processing	10	8	246	0.4772
GO:0051704	IPI00685510;IPI00690581;IPI00703084;IPI00707776;IPI00710664;IPI00713313;IPI00716157;IPI01028455	Biological Process	Multi-organism process	3	8	246	0.4987
GO:0010605	IPI00690581;IPI00694688;IPI00698213;IPI00709876;IPI00710274;IPI00712263;IPI00712384;IPI01003286	Biological Process	Negative regulation of macromolecule metabolic process	6	8	246	0.9699

GO:0022008	IPI00685510;IPI00687578;IPI00710274;IPI00713597;IPI00716830;IPI00866975;IPI00876805;IPI01003286	Biological Process	Neurogenesis	7	8	246	0.6924
GO:0009891	IPI00685510;IPI00690309;IPI00695037;IPI00704902;IPI00707857;IPI00714898;IPI00715059;IPI00866975	Biological Process	Positive regulation of biosynthetic process	6	8	246	0.8313
GO:0043085	IPI00685617;IPI00692668;IPI00705237;IPI00709876;IPI00713597;IPI00715059;IPI00717764;IPI00866858	Biological Process	Positive regulation of catalytic activity	5	8	246	0.6924
GO:0031328	IPI00685510;IPI00690309;IPI00695037;IPI00704902;IPI00707857;IPI00714898;IPI00715059;IPI00866975	Biological Process	Positive regulation of cellular biosynthetic process	7	8	246	0.8082
GO:0010628	IPI00685510;IPI00690309;IPI00695037;IPI00704902;IPI00705237;IPI00707857;IPI00714898;IPI00866975	Biological Process	Positive regulation of gene expression	8	8	246	0.6576
GO:0010557	IPI00685510;IPI00690309;IPI00695037;IPI00704902;IPI00707857;IPI00714898;IPI00715059;IPI00866975	Biological Process	Positive regulation of macromolecule biosynthetic process	8	8	246	0.7622
GO:0044093	IPI00685617;IPI00692668;IPI00705237;IPI00709876;IPI00713597;IPI00715059;IPI00717764;IPI00866858	Biological Process	Positive regulation of molecular function	5	8	246	0.8878
GO:0006508	IPI00685804;IPI00689165;IPI00689379;IPI00690581;IPI00704041;IPI00710664;IPI00715414;IPI00715640	Biological Process	Proteolysis	6	8	246	0.9599

GO:0045595	IPI00685510;IPI00690309;IPI00705441;IPI00707776;IPI00707857;IPI00708070;IPI00709876;IPI00712263	Biological Process	Regulation of cell differentiation	7	8	246	0.7089
GO:2000026	IPI00685510;IPI00690309;IPI00695042;IPI00707776;IPI00707857;IPI00709876;IPI00712524;IPI00717764	Biological Process	Regulation of multicellular organismal development	6	8	246	0.7958
GO:0046578	IPI00688451;IPI00690309;IPI00692668;IPI00705237;IPI00713049;IPI00713474;IPI00866858;IPI01000210	Biological Process	Regulation of Ras protein signal transduction	10	8	246	0.1132
GO:0051056	IPI00688451;IPI00690309;IPI00692668;IPI00705237;IPI00713049;IPI00713474;IPI00866858;IPI01000210	Biological Process	Regulation of small GTPase mediated signal transduction	9	8	246	0.1430
GO:0006915	IPI00685510;IPI00687578;IPI00697349;IPI00698213;IPI00705378;IPI00706314;IPI00709124	Biological Process	Apoptotic process	7	7	246	0.8844
GO:0072358	IPI00707857;IPI00708070;IPI00708244;IPI00709876;IPI00713049;IPI00731432;IPI00839150	Biological Process	Cardiovascular system development	7	7	246	0.6106
GO:0007049	IPI00685510;IPI00687578;IPI00693579;IPI00696748;IPI00712399;IPI00718152;IPI00906031	Biological Process	Cell cycle	5	7	246	0.8662
GO:0048468	IPI00685510;IPI00690309;IPI00697648;IPI00716830;IPI00866975;IPI00876805;IPI00924039	Biological Process	Cell development	7	7	246	0.9403
GO:0006325	IPI00689641;IPI00698339;IPI00705104;IPI00707776;IPI00712399;IPI00718678;IPI00727591	Biological Process	Chromatin organization	6	7	246	0.1230

GO:0072359	IPI00707857;IPI00708070;IPI00708244;IPI00709876;IPI00713049;IPI00731432;IPI00839150	Biological Process	Circulatory system development	7	7	246	0.6106
GO:0009790	IPI00687578;IPI00688704;IPI00699039;IPI00703813;IPI00707857;IPI00709124;IPI00710274	Biological Process	Embryo development	6	7	246	0.8801
GO:0006897	IPI00685510;IPI00693824;IPI00698823;IPI00706037;IPI00708037;IPI00709876;IPI00866975	Biological Process	Endocytosis	7	7	246	0.2054
GO:1901657	IPI00686872;IPI00694430;IPI00699226;IPI00704332;IPI00705159;IPI00709888;IPI00710377	Biological Process	Glycosyl compound metabolic process	5	7	246	0.5898
GO:0048193	IPI00692963;IPI00695852;IPI00696116;IPI00708162;IPI00713460;IPI00713474;IPI01003286	Biological Process	Golgi vesicle transport	6	7	246	0.1075
GO:0002376	IPI00691093;IPI00697595;IPI00698916;IPI00703084;IPI00705583;IPI00709876;IPI00866975	Biological Process	Immune system process	3	7	246	0.9852
GO:0050801	IPI00685510;IPI00703084;IPI00703813;IPI00709876;IPI00709888;IPI00710664;IPI00711457	Biological Process	Ion homeostasis	7	7	246	0.1855
GO:0061024	IPI00691093;IPI00696116;IPI00703954;IPI00709876;IPI00713474;IPI00843305;IPI00866975	Biological Process	Membrane organization	5	7	246	0.6208
GO:0009890	IPI00690581;IPI00698213;IPI00705159;IPI00709876;IPI00710274;IPI00712384;IPI01003286	Biological Process	Negative regulation of biosynthetic process	6	7	246	0.8290

GO:0048585	IPI00690309;IPI00690581;IPI00698823;IPI00709876;IPI00710274;IPI00731432;IPI00845178	Biological Process	Negative regulation of response to stimulus	5	7	246	0.7390
GO:0055086	IPI00686872;IPI00694430;IPI00699226;IPI00704332;IPI00705159;IPI00709888;IPI00710377	Biological Process	Nucleobase-containing small molecule metabolic process	7	7	246	0.8966
GO:0009116	IPI00686872;IPI00694430;IPI00699226;IPI00704332;IPI00705159;IPI00709888;IPI00710377	Biological Process	Nucleoside metabolic process	8	7	246	0.5576
GO:0006753	IPI00686872;IPI00694430;IPI00699226;IPI00704332;IPI00705159;IPI00709888;IPI00710377	Biological Process	Nucleoside phosphate metabolic process	8	7	246	0.8404
GO:0009117	IPI00686872;IPI00694430;IPI00699226;IPI00704332;IPI00705159;IPI00709888;IPI00710377	Biological Process	Nucleotide metabolic process	9	7	246	0.8347
GO:0008284	IPI00685617;IPI00690789;IPI00694565;IPI00703084;IPI00707776;IPI00712858;IPI00715059	Biological Process	Positive regulation of cell proliferation	5	7	246	0.2156
GO:0032270	IPI00685617;IPI00690309;IPI00709876;IPI00713597;IPI00716725;IPI00717764;IPI00847122	Biological Process	Positive regulation of cellular protein metabolic process	8	7	246	0.4902
GO:0051173	IPI00685510;IPI00690309;IPI00695037;IPI00704902;IPI00707857;IPI00714898;IPI00866975	Biological Process	Positive regulation of nitrogen compound metabolic process	6	7	246	0.8662
GO:0045935	IPI00685510;IPI00690309;IPI00695037;IPI00704902;IPI00707857;IPI00714898;IPI00866975	Biological Process	Positive regulation of nucleobase-containing compound metabolic process	8	7	246	0.8230

GO:0051247	IPI00685617;IPI00690309;IPI00709876;IPI00713597;IPI00716725;IPI00717764;IPI00847122	Biological Process	Positive regulation of protein metabolic process	7	7	246	0.6308
GO:0051254	IPI00685510;IPI00690309;IPI00695037;IPI00704902;IPI00707857;IPI00714898;IPI00866975	Biological Process	Positive regulation of RNA metabolic process	10	7	246	0.7623
GO:0045893	IPI00685510;IPI00690309;IPI00695037;IPI00704902;IPI00707857;IPI00714898;IPI00866975	Biological Process	Positive regulation of transcription, DNA-templated	12	7	246	0.6787
GO:0012501	IPI00685510;IPI00687578;IPI00697349;IPI00698213;IPI00705378;IPI00706314;IPI00709124	Biological Process	Programmed cell death	6	7	246	0.8927
GO:0042278	IPI00686872;IPI00694430;IPI00699226;IPI00704332;IPI00705159;IPI00709888;IPI00710377	Biological Process	Purine nucleoside metabolic process	9	7	246	0.4320
GO:0006163	IPI00686872;IPI00694430;IPI00699226;IPI00704332;IPI00705159;IPI00709888;IPI00710377	Biological Process	Purine nucleotide metabolic process	10	7	246	0.4787
GO:0046128	IPI00686872;IPI00694430;IPI00699226;IPI00704332;IPI00705159;IPI00709888;IPI00710377	Biological Process	Purine ribonucleoside metabolic process	10	7	246	0.4202
GO:0009150	IPI00686872;IPI00694430;IPI00699226;IPI00704332;IPI00705159;IPI00709888;IPI00710377	Biological Process	Purine ribonucleotide metabolic process	11	7	246	0.4320
GO:0072521	IPI00686872;IPI00694430;IPI00699226;IPI00704332;IPI00705159;IPI00709888;IPI00710377	Biological Process	Purine-containing compound metabolic process	6	7	246	0.5684

GO:0010941	IPI00690581;IPI00703084;IPI00706314;IPI00709124;IPI00709876;IPI00712858;IPI01028455	Biological Process	Regulation of cell death	6	7	246	0.9796
GO:0032386	IPI00690309;IPI00696116;IPI00709876;IPI00712858;IPI00714269;IPI00906031;IPI01028468	Biological Process	Regulation of intracellular transport	6	7	246	0.1572
GO:0032880	IPI00690309;IPI00696116;IPI00703084;IPI00714269;IPI00866858;IPI00866975;IPI01028468	Biological Process	Regulation of protein localization	6	7	246	0.2693
GO:0009628	IPI00685510;IPI00690309;IPI00707857;IPI00709876;IPI00710920;IPI00717764;IPI00731432	Biological Process	Response to abiotic stimulus	4	7	246	0.5684
GO:0009725	IPI00690309;IPI00696116;IPI00698823;IPI00703813;IPI00709876;IPI00715059;IPI01028182	Biological Process	Response to hormone	5	7	246	0.0678
GO:1901698	IPI00690309;IPI00696116;IPI00707857;IPI00708244;IPI00715059;IPI00731432;IPI00826022	Biological Process	Response to nitrogen compound	5	7	246	0.2583
GO:0010243	IPI00690309;IPI00696116;IPI00707857;IPI00708244;IPI00715059;IPI00731432;IPI00826022	Biological Process	Response to organonitrogen compound	5	7	246	0.1572
GO:0009611	IPI00685653;IPI00700673;IPI00705565;IPI00717764;IPI00717930;IPI00731432;IPI00843305	Biological Process	Response to wounding	5	7	246	0.3966
GO:0009119	IPI00686872;IPI00694430;IPI00699226;IPI00704332;IPI00705159;IPI00709888;IPI00710377	Biological Process	Ribonucleoside metabolic process	9	7	246	0.5017

GO:0009259	IPI00686872;IPI00694430;IPI00699226;IPI00704332;IPI00705159;IPI00709888;IPI00710377	Biological Process	Ribonucleotide metabolic process	10	7	246	0.5017
GO:0019693	IPI00686872;IPI00694430;IPI00699226;IPI00704332;IPI00705159;IPI00709888;IPI00710377	Biological Process	Ribose phosphate metabolic process	7	7	246	0.5131
GO:0044711	IPI00690789;IPI00694430;IPI00695952;IPI00705565;IPI00714621;IPI00715066;IPI00760457	Biological Process	Single-organism biosynthetic process	5	7	246	0.5792
GO:0044802	IPI00691093;IPI00696116;IPI00703954;IPI00709876;IPI00713474;IPI00843305;IPI00866975	Biological Process	Single-organism membrane organization	4	7	246	0.5792
GO:0044283	IPI00690789;IPI00694430;IPI00695952;IPI00705565;IPI00714621;IPI00715066;IPI00760457	Biological Process	Small molecule biosynthetic process	6	7	246	0.5576
GO:0030036	IPI00697648;IPI00708767;IPI00709888;IPI00710274;IPI00712288;IPI00924039	Biological Process	Actin cytoskeleton organization	6	6	246	0.7211
GO:0030029	IPI00697648;IPI00708767;IPI00709888;IPI00710274;IPI00712288;IPI00924039	Biological Process	Actin filament-based process	5	6	246	0.7554
GO:0019439	IPI00694430;IPI00698059;IPI00704902;IPI00709124;IPI00710377;IPI01017508	Biological Process	Aromatic compound catabolic process	6	6	246	0.6535
GO:0001568	IPI00707857;IPI00708244;IPI00709876;IPI00713049;IPI00731432;IPI00839150	Biological Process	Blood vessel development	8	6	246	0.3397
GO:0055082	IPI00685510;IPI00703813;IPI00709876;IPI00709888;IPI00710664;IPI00711457	Biological Process	Cellular chemical homeostasis	6	6	246	0.2059

GO:0006873	IPI00685510;IPI00703813;IPI00709876;IPI00709888;IPI00710664;IPI00711457	Biological Process	Cellular ion homeostasis	7	6	246	0.1732
GO:0071496	IPI00690309;IPI00698213;IPI00707857;IPI00709876;IPI00714656;IPI01028455	Biological Process	Cellular response to external stimulus	5	6	246	0.0184
GO:1901699	IPI00696116;IPI00707857;IPI00708244;IPI00715059;IPI00731432;IPI00826022	Biological Process	Cellular response to nitrogen compound	6	6	246	0.1243
GO:0071417	IPI00696116;IPI00707857;IPI00708244;IPI00715059;IPI00731432;IPI00826022	Biological Process	Cellular response to organonitrogen compound	7	6	246	0.0909
GO:0051186	IPI00691093;IPI00694430;IPI00695952;IPI00703084;IPI00704332;IPI00871114	Biological Process	Cofactor metabolic process	5	6	246	0.4167
GO:0046486	IPI00685617;IPI00687661;IPI00703084;IPI00706879;IPI00709876;IPI00839150	Biological Process	Glycerolipid metabolic process	6	6	246	0.2059
GO:1901659	IPI00686872;IPI00699226;IPI00704332;IPI00705159;IPI00709888;IPI00710377	Biological Process	Glycosyl compound biosynthetic process	6	6	246	0.1528
GO:0009057	IPI00690581;IPI00704041;IPI00704902;IPI00709124;IPI00717764;IPI01017508	Biological Process	Macromolecule catabolic process	6	6	246	0.9271
GO:0030001	IPI00693452;IPI00703813;IPI00709876;IPI00709888;IPI00710664;IPI00711457	Biological Process	Metal ion transport	7	6	246	0.0695
GO:0032787	IPI00694430;IPI00698823;IPI00703084;IPI00705565;IPI00714621;IPI00717758	Biological Process	Monocarboxylic acid metabolic process	8	6	246	0.5652
GO:0031327	IPI00698213;IPI00705159;IPI00709876;IPI00710274;IPI00712384;IPI01003286	Biological Process	Negative regulation of cellular biosynthetic process	7	6	246	0.8862

GO:0051093	IPI00685510;IPI00705441;IPI00708070;IPI00709876;IPI00712263;IPI00712524	Biological Process	Negative regulation of developmental process	5	6	246	0.3653
GO:0010558	IPI00690581;IPI00698213;IPI00709876;IPI00710274;IPI00712384;IPI01003286	Biological Process	Negative regulation of macromolecule biosynthetic process	8	6	246	0.8671
GO:0051172	IPI00694688;IPI00698213;IPI00709876;IPI00710274;IPI00712263;IPI01003286	Biological Process	Negative regulation of nitrogen compound metabolic process	6	6	246	0.8721
GO:0045934	IPI00694688;IPI00698213;IPI00709876;IPI00710274;IPI00712263;IPI01003286	Biological Process	Negative regulation of nucleobase-containing compound metabolic process	8	6	246	0.8721
GO:0050877	IPI00685510;IPI00696116;IPI00698059;IPI00707857;IPI00710920;IPI00876805	Biological Process	Neurological system process	6	6	246	0.5768
GO:0009163	IPI00686872;IPI00699226;IPI00704332;IPI00705159;IPI00709888;IPI00710377	Biological Process	Nucleoside biosynthetic process	8	6	246	0.1528
GO:1901293	IPI00686872;IPI00699226;IPI00704332;IPI00705159;IPI00709888;IPI00710377	Biological Process	Nucleoside phosphate biosynthetic process	8	6	246	0.4039
GO:0009165	IPI00686872;IPI00699226;IPI00704332;IPI00705159;IPI00709888;IPI00710377	Biological Process	Nucleotide biosynthetic process	9	6	246	0.4039
GO:1901361	IPI00694430;IPI00698059;IPI00704902;IPI00709124;IPI00710377;IPI01017508	Biological Process	Organic cyclic compound catabolic process	6	6	246	0.6836
GO:1901565	IPI00694430;IPI00698059;IPI00710377;IPI00715066;IPI00717764;IPI00871114	Biological Process	Organonitrogen compound catabolic process	6	6	246	0.6738
GO:0045937	IPI00685617;IPI00690309;IPI00709876;IPI00713597;IPI00717764;IPI00847122	Biological Process	Positive regulation of phosphate metabolic process	8	6	246	0.3142

GO:0010562	IPI00685617;IPI00690309;IPI00709876;IPI00713597;IPI00717764;IPI00847122	Biological Process	Positive regulation of phosphorus metabolic process	6	6	246	0.3142
GO:0042327	IPI00685617;IPI00690309;IPI00709876;IPI00713597;IPI00717764;IPI00847122	Biological Process	Positive regulation of phosphorylation	9	6	246	0.2172
GO:0031401	IPI00685617;IPI00690309;IPI00709876;IPI00713597;IPI00717764;IPI00847122	Biological Process	Positive regulation of protein modification process	9	6	246	0.5051
GO:0001934	IPI00685617;IPI00690309;IPI00709876;IPI00713597;IPI00717764;IPI00847122	Biological Process	Positive regulation of protein phosphorylation	10	6	246	0.2059
GO:0051050	IPI00693452;IPI00696116;IPI00709876;IPI00714269;IPI00715059;IPI01028468	Biological Process	Positive regulation of transport	7	6	246	0.5174
GO:0042451	IPI00686872;IPI00699226;IPI00704332;IPI00705159;IPI00709888;IPI00710377	Biological Process	Purine nucleoside biosynthetic process	9	6	246	0.0909
GO:0006164	IPI00686872;IPI00699226;IPI00704332;IPI00705159;IPI00709888;IPI00710377	Biological Process	Purine nucleotide biosynthetic process	10	6	246	0.1335
GO:0046129	IPI00686872;IPI00699226;IPI00704332;IPI00705159;IPI00709888;IPI00710377	Biological Process	Purine ribonucleoside biosynthetic process	10	6	246	0.0909
GO:0009152	IPI00686872;IPI00699226;IPI00704332;IPI00705159;IPI00709888;IPI00710377	Biological Process	Purine ribonucleotide biosynthetic process	11	6	246	0.1154
GO:0072522	IPI00686872;IPI00699226;IPI00704332;IPI00705159;IPI00709888;IPI00710377	Biological Process	Purine-containing compound biosynthetic process	7	6	246	0.1528
GO:0042981	IPI00690581;IPI00703084;IPI00709124;IPI00709876;IPI00712858;IPI01028455	Biological Process	Regulation of apoptotic process	8	6	246	0.9888

GO:0051270	IPI00695042;IPI00707857;IPI00708070;IPI00715059;IPI00845178;IPI00847122	Biological Process	Regulation of cellular component movement	6	6	246	0.6431
GO:0070201	IPI00690309;IPI00696116;IPI00703084;IPI00714269;IPI00866858;IPI01028468	Biological Process	Regulation of establishment of protein localization	7	6	246	0.2768
GO:0033124	IPI00688451;IPI00690309;IPI00692668;IPI00705237;IPI00713049;IPI00866858	Biological Process	Regulation of GTP catabolic process	13	6	246	0.2059
GO:0043087	IPI00688451;IPI00690309;IPI00692668;IPI00705237;IPI00713049;IPI00866858	Biological Process	Regulation of GTPase activity	14	6	246	0.1948
GO:0019216	IPI00685617;IPI00690309;IPI00694430;IPI00705159;IPI00709876;IPI00715059	Biological Process	Regulation of lipid metabolic process	6	6	246	0.0834
GO:0043408	IPI00685617;IPI00697917;IPI00709876;IPI00710274;IPI00713597;IPI00717764	Biological Process	Regulation of MAPK cascade	10	6	246	0.3269
GO:0009118	IPI00688451;IPI00690309;IPI00692668;IPI00705237;IPI00713049;IPI00866858	Biological Process	Regulation of nucleoside metabolic process	9	6	246	0.2525
GO:0030811	IPI00688451;IPI00690309;IPI00692668;IPI00705237;IPI00713049;IPI00866858	Biological Process	Regulation of nucleotide catabolic process	10	6	246	0.2525
GO:0006140	IPI00688451;IPI00690309;IPI00692668;IPI00705237;IPI00713049;IPI00866858	Biological Process	Regulation of nucleotide metabolic process	10	6	246	0.3781
GO:0043067	IPI00690581;IPI00703084;IPI00709124;IPI00709876;IPI00712858;IPI01028455	Biological Process	Regulation of programmed cell death	7	6	246	0.9904
GO:0033121	IPI00688451;IPI00690309;IPI00692668;IPI00705237;IPI00713049;IPI00866858	Biological Process	Regulation of purine nucleotide catabolic process	11	6	246	0.2525

GO:1900542	IPI00688451;IPI00690309;IPI00692668;IPI00705237;IPI00713049;IPI00866858	Biological Process	Regulation of purine nucleotide metabolic process	11	6	246	0.3781
GO:0032318	IPI00688451;IPI00690309;IPI00692668;IPI00705237;IPI00713049;IPI00866858	Biological Process	Regulation of Ras GTPase activity	15	6	246	0.0695
GO:0006357	IPI00685510;IPI00695037;IPI00704902;IPI00709876;IPI00714898;IPI01003286	Biological Process	Regulation of transcription from RNA polymerase II promoter	12	6	246	0.8817
GO:0000003	IPI00685510;IPI00690309;IPI00702768;IPI00709876;IPI00712399;IPI00924039	Biological Process	Reproduction	3	6	246	0.7554
GO:0022414	IPI00685510;IPI00690309;IPI00702768;IPI00709876;IPI00712399;IPI00924039	Biological Process	Reproductive process	3	6	246	0.6431
GO:0009991	IPI00690309;IPI00698213;IPI00702768;IPI00709876;IPI00714656;IPI01028455	Biological Process	Response to extracellular stimulus	5	6	246	0.0063
GO:0033993	IPI00690581;IPI00698823;IPI00702768;IPI00703813;IPI00709876;IPI01028182	Biological Process	Response to lipid	6	6	246	0.2059
GO:0031667	IPI00690309;IPI00698213;IPI00702768;IPI00709876;IPI00714656;IPI01028455	Biological Process	Response to nutrient levels	6	6	246	0.0032
GO:0014070	IPI00698823;IPI00702768;IPI00703813;IPI00709876;IPI00712596;IPI01028182	Biological Process	Response to organic cyclic compound	6	6	246	0.2059
GO:0042455	IPI00686872;IPI00699226;IPI00704332;IPI00705159;IPI00709888;IPI00710377	Biological Process	Ribonucleoside biosynthetic process	9	6	246	0.1528
GO:0009260	IPI00686872;IPI00699226;IPI00704332;IPI00705159;IPI00709888;IPI00710377	Biological Process	Ribonucleotide biosynthetic process	10	6	246	0.1732

GO:0046390	IPI00686872;IPI00699226;IPI00704332;IPI00705159;IPI00709888;IPI00710377	Biological Process	Ribose phosphate biosynthetic process	7	6	246	0.1839
GO:0043588	IPI00707857;IPI00708244;IPI00709888;IPI00731432;IPI00826022;IPI01003286	Biological Process	Skin development	8	6	246	0.0762
GO:0055085	IPI00686872;IPI00697349;IPI00699372;IPI00705378;IPI00706314;IPI00712858	Biological Process	Transmembrane transport	5	6	246	0.1732
GO:0001944	IPI00707857;IPI00708244;IPI00709876;IPI00713049;IPI00731432;IPI00839150	Biological Process	Vasculature development	7	6	246	0.3524
GO:0006754	IPI00686872;IPI00699226;IPI00705159;IPI00709888;IPI00710377	Biological Process	ATP biosynthetic process	11	5	246	0.0269
GO:0046034	IPI00686872;IPI00699226;IPI00705159;IPI00709888;IPI00710377	Biological Process	ATP metabolic process	12	5	246	0.1441
GO:0005975	IPI00698823;IPI00699039;IPI00717764;IPI00827121;IPI00906031	Biological Process	Carbohydrate metabolic process	5	5	246	0.9845
GO:0046394	IPI00694430;IPI00705565;IPI00714621;IPI00715066;IPI00760457	Biological Process	Carboxylic acid biosynthetic process	7	5	246	0.5359
GO:0055080	IPI00685510;IPI00709876;IPI00709888;IPI00710664;IPI00711457	Biological Process	Cation homeostasis	8	5	246	0.3830
GO:0030030	IPI00685510;IPI00687578;IPI00690309;IPI00716830;IPI00866975	Biological Process	Cell projection organization	5	5	246	0.8275
GO:0008283	IPI00687578;IPI00710274;IPI00713597;IPI00727591;IPI00760457	Biological Process	Cell proliferation	4	5	246	0.4955
GO:0007267	IPI00685510;IPI00696116;IPI00698059;IPI00708070;IPI00876805	Biological Process	Cell-cell signaling	6	5	246	0.2166

GO:0030003	IPI00685510;IPI00709876;IPI00709888;IPI00710664;IPI00711457	Biological Process	Cellular cation homeostasis	8	5	246	0.2564
GO:0032989	IPI00685510;IPI00687578;IPI00697648;IPI00866975;IPI00924039	Biological Process	Cellular component morphogenesis	6	5	246	0.8586
GO:0006928	IPI00685510;IPI00687578;IPI00697595;IPI00713597;IPI00866858	Biological Process	Cellular component movement	5	5	246	0.9547
GO:0044265	IPI00690581;IPI00704041;IPI00704902;IPI00709124;IPI01017508	Biological Process	Cellular macromolecule catabolic process	6	5	246	0.9316
GO:0006875	IPI00685510;IPI00709876;IPI00709888;IPI00710664;IPI00711457	Biological Process	Cellular metal ion homeostasis	9	5	246	0.1912
GO:0044270	IPI00694430;IPI00704902;IPI00709124;IPI00710377;IPI01017508	Biological Process	Cellular nitrogen compound catabolic process	6	5	246	0.7665
GO:0043623	IPI00688474;IPI00689226;IPI00689365;IPI00708767;IPI00866975	Biological Process	Cellular protein complex assembly	8	5	246	0.3687
GO:0031668	IPI00690309;IPI00698213;IPI00709876;IPI00714656;IPI01028455	Biological Process	Cellular response to extracellular stimulus	6	5	246	0.0105
GO:0031669	IPI00690309;IPI00698213;IPI00709876;IPI00714656;IPI01028455	Biological Process	Cellular response to nutrient levels	7	5	246	0.0050
GO:0043009	IPI00687578;IPI00688704;IPI00703813;IPI00707857;IPI00709124	Biological Process	Chordate embryonic development	8	5	246	0.8697
GO:0006732	IPI00694430;IPI00695952;IPI00703084;IPI00704332;IPI00871114	Biological Process	Coenzyme metabolic process	6	5	246	0.4258
GO:0009792	IPI00687578;IPI00688704;IPI00703813;IPI00707857;IPI00709124	Biological Process	Embryo development ending in birth or egg hatching	7	5	246	0.8697
GO:0030855	IPI00696748;IPI00710274;IPI00713313;IPI00876805;IPI01003286	Biological Process	Epithelial cell differentiation	7	5	246	0.4818

GO:0030198	IPI00685510;IPI00707857;IPI00708244;IPI00731432;IPI00826022	Biological Process	Extracellular matrix organization	6	5	246	0.1333
GO:0043062	IPI00685510;IPI00707857;IPI00708244;IPI00731432;IPI00826022	Biological Process	Extracellular structure organization	5	5	246	0.1333
GO:0006631	IPI00694430;IPI00703084;IPI00705565;IPI00714621;IPI00717758	Biological Process	Fatty acid metabolic process	9	5	246	0.5490
GO:0048699	IPI00685510;IPI00687578;IPI00716830;IPI00866975;IPI01003286	Biological Process	Generation of neurons	8	5	246	0.9070
GO:0006091	IPI00687478;IPI00697194;IPI00698213;IPI00730219;IPI00906031	Biological Process	Generation of precursor metabolites and energy	5	5	246	0.9547
GO:0006650	IPI00685617;IPI00687661;IPI00703084;IPI00706879;IPI00839150	Biological Process	Glycerophospholipid metabolic process	8	5	246	0.1790
GO:0046700	IPI00694430;IPI00704902;IPI00709124;IPI00710377;IPI01017508	Biological Process	Heterocycle catabolic process	6	5	246	0.7665
GO:0006954	IPI00685653;IPI00700673;IPI00705565;IPI00717764;IPI00717930	Biological Process	Inflammatory response	6	5	246	0.1228
GO:0040011	IPI00685510;IPI00687578;IPI00697595;IPI00713597;IPI00866858	Biological Process	Locomotion	3	5	246	0.8586
GO:0055065	IPI00685510;IPI00709876;IPI00709888;IPI00710664;IPI00711457	Biological Process	Metal ion homeostasis	9	5	246	0.2978
GO:0035637	IPI00685510;IPI00696116;IPI00698059;IPI00708070;IPI00876805	Biological Process	Multicellular organismal signaling	5	5	246	0.3402
GO:0007517	IPI00685510;IPI00697648;IPI00708070;IPI00709876;IPI00876805	Biological Process	Muscle organ development	8	5	246	0.1333
GO:0061061	IPI00685510;IPI00697648;IPI00708070;IPI00709876;IPI00876805	Biological Process	Muscle structure development	5	5	246	0.4116

GO:0060537	IPI00685510;IPI00697648;IPI00708070;IPI00709876;IPI00876805	Biological Process	Muscle tissue development	6	5	246	0.2166
GO:0034660	IPI00689365;IPI00702948;IPI00708246;IPI00867017;IPI01017508	Biological Process	ncRNA metabolic process	9	5	246	0.5490
GO:0010648	IPI00690309;IPI00698823;IPI00709876;IPI00710274;IPI00845178	Biological Process	Negative regulation of cell communication	7	5	246	0.8800
GO:0060548	IPI00690581;IPI00703084;IPI00706314;IPI00709876;IPI01028455	Biological Process	Negative regulation of cell death	7	5	246	0.8849
GO:0045596	IPI00685510;IPI00705441;IPI00708070;IPI00709876;IPI00712263	Biological Process	Negative regulation of cell differentiation	8	5	246	0.3260
GO:2000113	IPI00698213;IPI00709876;IPI00710274;IPI00712384;IPI01003286	Biological Process	Negative regulation of cellular macromolecule biosynthetic process	8	5	246	0.9109
GO:0051253	IPI00694688;IPI00698213;IPI00709876;IPI00712263;IPI01003286	Biological Process	Negative regulation of RNA metabolic process	10	5	246	0.8897
GO:0071705	IPI00689226;IPI00691086;IPI00700228;IPI00708597;IPI00710920	Biological Process	Nitrogen compound transport	6	5	246	0.2700
GO:0034655	IPI00694430;IPI00704902;IPI00709124;IPI00710377;IPI01017508	Biological Process	Nucleobase-containing compound catabolic process	7	5	246	0.6692
GO:0009124	IPI00686872;IPI00699226;IPI00705159;IPI00709888;IPI00710377	Biological Process	Nucleoside monophosphate biosynthetic process	9	5	246	0.2038
GO:0009123	IPI00686872;IPI00699226;IPI00705159;IPI00709888;IPI00710377	Biological Process	Nucleoside monophosphate metabolic process	9	5	246	0.4540
GO:0009142	IPI00686872;IPI00699226;IPI00705159;IPI00709888;IPI00710377	Biological Process	Nucleoside triphosphate biosynthetic process	9	5	246	0.0614
GO:0009141	IPI00686872;IPI00699226;IPI00705159;IPI00709888;IPI00710377	Biological Process	Nucleoside triphosphate metabolic process	9	5	246	0.5490

GO:0016053	IPI00694430;IPI00705565;IPI00714621;IPI00715066;IPI00760457	Biological Process	Organic acid biosynthetic process	6	5	246	0.5359
GO:1901615	IPI00685510;IPI00690789;IPI00697194;IPI00698059;IPI00839150	Biological Process	Organic hydroxy compound metabolic process	5	5	246	0.4540
GO:0055114	IPI00687478;IPI00697194;IPI00698213;IPI00730219;IPI00906031	Biological Process	Oxidation-reduction process	5	5	246	0.9780
GO:0006644	IPI00685617;IPI00687661;IPI00703084;IPI00706879;IPI00839150	Biological Process	Phospholipid metabolic process	7	5	246	0.2838
GO:0051094	IPI00690309;IPI00695042;IPI00707776;IPI00707857;IPI00717764	Biological Process	Positive regulation of developmental process	5	5	246	0.6800
GO:0051240	IPI00703084;IPI00705159;IPI00706879;IPI00709876;IPI00713474	Biological Process	Positive regulation of multicellular organismal process	5	5	246	0.2978
GO:0006457	IPI00687839;IPI00689629;IPI00698916;IPI00707455;IPI00707815	Biological Process	Protein folding	7	5	246	0.7398
GO:0033365	IPI00707815;IPI00707857;IPI00714269;IPI00715059;IPI00716830	Biological Process	Protein localization to organelle	7	5	246	0.7009
GO:0051259	IPI00689899;IPI00707857;IPI00708244;IPI00709876;IPI00710834	Biological Process	Protein oligomerization	8	5	246	0.5226
GO:0009127	IPI00686872;IPI00699226;IPI00705159;IPI00709888;IPI00710377	Biological Process	Purine nucleoside monophosphate biosynthetic process	10	5	246	0.1441
GO:0009126	IPI00686872;IPI00699226;IPI00705159;IPI00709888;IPI00710377	Biological Process	Purine nucleoside monophosphate metabolic process	10	5	246	0.3830
GO:0009145	IPI00686872;IPI00699226;IPI00705159;IPI00709888;IPI00710377	Biological Process	Purine nucleoside triphosphate biosynthetic process	10	5	246	0.0420
GO:0009144	IPI00686872;IPI00699226;IPI00705159;IPI00709888;IPI00710377	Biological Process	Purine nucleoside triphosphate metabolic process	10	5	246	0.4955

GO:0009168	IPI00686872;IPI00699226;IPI00705159;IPI00709888;IPI00710377	Biological Process	Purine ribonucleoside monophosphate biosynthetic process	11	5	246	0.1441
GO:0009167	IPI00686872;IPI00699226;IPI00705159;IPI00709888;IPI00710377	Biological Process	Purine ribonucleoside monophosphate metabolic process	11	5	246	0.3830
GO:0009206	IPI00686872;IPI00699226;IPI00705159;IPI00709888;IPI00710377	Biological Process	Purine ribonucleoside triphosphate biosynthetic process	11	5	246	0.0420
GO:0009205	IPI00686872;IPI00699226;IPI00705159;IPI00709888;IPI00710377	Biological Process	Purine ribonucleoside triphosphate metabolic process	11	5	246	0.4680
GO:0022603	IPI00695042;IPI00707776;IPI00707857;IPI00712524;IPI00717764	Biological Process	Regulation of anatomical structure morphogenesis	5	5	246	0.7911
GO:0030334	IPI00695042;IPI00707857;IPI00715059;IPI00845178;IPI00847122	Biological Process	Regulation of cell migration	8	5	246	0.6469
GO:2000145	IPI00695042;IPI00707857;IPI00715059;IPI00845178;IPI00847122	Biological Process	Regulation of cell motility	7	5	246	0.7110
GO:0002682	IPI00690581;IPI00703084;IPI00705583;IPI00709876;IPI00731432	Biological Process	Regulation of immune system process	4	5	246	0.9525
GO:0040012	IPI00695042;IPI00707857;IPI00715059;IPI00845178;IPI00847122	Biological Process	Regulation of locomotion	4	5	246	0.7305
GO:0033043	IPI00689379;IPI00690309;IPI00693842;IPI00710274;IPI00712858	Biological Process	Regulation of organelle organization	5	5	246	0.8643
GO:0050730	IPI00685617;IPI00690309;IPI00709876;IPI00710274;IPI00713597	Biological Process	Regulation of peptidyl-tyrosine phosphorylation	9	5	246	0.0191
GO:0071900	IPI00685617;IPI00696748;IPI00709876;IPI00713597;IPI00717764	Biological Process	Regulation of protein serine/threonine kinase activity	11	5	246	0.2166
GO:0051223	IPI00690309;IPI00696116;IPI00703084;IPI00714269;IPI01028468	Biological Process	Regulation of protein transport	7	5	246	0.3402

GO:0080134	IPI00690309;IPI00690581;IPI00697917;IPI00698823;IPI00709876	Biological Process	Regulation of response to stress	5	5	246	0.9070
GO:0035023	IPI00690309;IPI00705237;IPI00713049;IPI00866858;IPI01000210	Biological Process	Regulation of Rho protein signal transduction	11	5	246	0.0766
GO:0001101	IPI00690309;IPI00707857;IPI00708244;IPI00731432;IPI00826022	Biological Process	Response to acid chemical	5	5	246	0.0269
GO:0043200	IPI00690309;IPI00707857;IPI00708244;IPI00731432;IPI00826022	Biological Process	Response to amino acid	6	5	246	0.0130
GO:0006979	IPI00685510;IPI00685678;IPI00690581;IPI00707455;IPI00716157	Biological Process	Response to oxidative stress	5	5	246	0.4116
GO:0009156	IPI00686872;IPI00699226;IPI00705159;IPI00709888;IPI00710377	Biological Process	Ribonucleoside monophosphate biosynthetic process	10	5	246	0.2038
GO:0009161	IPI00686872;IPI00699226;IPI00705159;IPI00709888;IPI00710377	Biological Process	Ribonucleoside monophosphate metabolic process	10	5	246	0.4540
GO:0009201	IPI00686872;IPI00699226;IPI00705159;IPI00709888;IPI00710377	Biological Process	Ribonucleoside triphosphate biosynthetic process	10	5	246	0.0614
GO:0009199	IPI00686872;IPI00699226;IPI00705159;IPI00709888;IPI00710377	Biological Process	Ribonucleoside triphosphate metabolic process	10	5	246	0.5091
GO:0046903	IPI00692417;IPI00696116;IPI00703084;IPI00709876;IPI00717764	Biological Process	Secretion	5	5	246	0.4399
GO:0001501	IPI00702768;IPI00707776;IPI00707857;IPI00708244;IPI00826022	Biological Process	Skeletal system development	7	5	246	0.1333
GO:0014706	IPI00685510;IPI00697648;IPI00708070;IPI00709876;IPI00876805	Biological Process	Striated muscle tissue development	7	5	246	0.1912
GO:0048729	IPI00699039;IPI00708070;IPI00710274;IPI00710920;IPI00713049	Biological Process	Tissue morphogenesis	5	5	246	0.5620

GO:0035295	IPI00699039;IPI00710920;IPI00713049;IPI00717764;IPI01003286	Biological Process	Tube development	6	5	246	0.4399
GO:0006066	IPI00685510;IPI00690789;IPI00697194;IPI00839150	Biological Process	Alcohol metabolic process	6	4	246	0.4834
GO:0055074	IPI00685510;IPI00709876;IPI00709888;IPI00711457	Biological Process	Calcium ion homeostasis	10	4	246	0.1408
GO:0006816	IPI00693452;IPI00709876;IPI00709888;IPI00711457	Biological Process	Calcium ion transport	9	4	246	0.0631
GO:0022402	IPI00685510;IPI00687578;IPI00693579;IPI00712399	Biological Process	Cell cycle process	6	4	246	0.9465
GO:0016477	IPI00687578;IPI00697595;IPI00713597;IPI00866858	Biological Process	Cell migration	7	4	246	0.8149
GO:0000902	IPI00685510;IPI00687578;IPI00866975;IPI00924039	Biological Process	Cell morphogenesis	7	4	246	0.8506
GO:0048870	IPI00687578;IPI00697595;IPI00713597;IPI00866858	Biological Process	Cell motility	6	4	246	0.8692
GO:0043603	IPI00685804;IPI00705565;IPI00876805;IPI01028182	Biological Process	Cellular amide metabolic process	6	4	246	0.3548
GO:0006874	IPI00685510;IPI00709876;IPI00709888;IPI00711457	Biological Process	Cellular calcium ion homeostasis	10	4	246	0.1280
GO:0072503	IPI00685510;IPI00709876;IPI00709888;IPI00711457	Biological Process	Cellular divalent inorganic cation homeostasis	9	4	246	0.1541
GO:0071229	IPI00707857;IPI00708244;IPI00731432;IPI00826022	Biological Process	Cellular response to acid chemical	6	4	246	0.0270
GO:0071230	IPI00707857;IPI00708244;IPI00731432;IPI00826022	Biological Process	Cellular response to amino acid stimulus	7	4	246	0.0173
GO:0009267	IPI00698213;IPI00709876;IPI00714656;IPI01028455	Biological Process	Cellular response to starvation	8	4	246	0.0101
GO:0031497	IPI00689641;IPI00698339;IPI00705104;IPI00712399	Biological Process	Chromatin assembly	11	4	246	0.0466
GO:0006333	IPI00689641;IPI00698339;IPI00705104;IPI00712399	Biological Process	Chromatin assembly or disassembly	7	4	246	0.0928

GO:0030199	IPI00707857;IPI00708244;IPI00731432;IPI00826022	Biological Process	Collagen fibril organization	7	4	246	0.0173
GO:0072507	IPI00685510;IPI00709876;IPI00709888;IPI00711457	Biological Process	Divalent inorganic cation homeostasis	9	4	246	0.1678
GO:0072511	IPI00693452;IPI00709876;IPI00709888;IPI00711457	Biological Process	Divalent inorganic cation transport	7	4	246	0.0631
GO:0070838	IPI00693452;IPI00709876;IPI00709888;IPI00711457	Biological Process	Divalent metal ion transport	8	4	246	0.0631
GO:0071103	IPI00689641;IPI00698339;IPI00705104;IPI00712399	Biological Process	DNA conformation change	9	4	246	0.1408
GO:0006323	IPI00689641;IPI00698339;IPI00705104;IPI00712399	Biological Process	DNA packaging	10	4	246	0.0928
GO:0022900	IPI00687478;IPI00697194;IPI00698213;IPI00730219	Biological Process	Electron transport chain	6	4	246	0.6894
GO:0007167	IPI00708244;IPI00713597;IPI00715059;IPI00731432	Biological Process	Enzyme linked receptor protein signaling pathway	8	4	246	0.3221
GO:0045017	IPI00685617;IPI00687661;IPI00703084;IPI00706879	Biological Process	Glycerolipid biosynthetic process	6	4	246	0.0724
GO:0046474	IPI00685617;IPI00687661;IPI00703084;IPI00706879	Biological Process	Glycerophospholipid biosynthetic process	7	4	246	0.0631
GO:0001701	IPI00687578;IPI00688704;IPI00703813;IPI00709124	Biological Process	In utero embryonic development	9	4	246	0.8299
GO:0051674	IPI00687578;IPI00697595;IPI00713597;IPI00866858	Biological Process	Localization of cell	4	4	246	0.8692
GO:0007005	IPI00688474;IPI00689365;IPI00706314;IPI00707815	Biological Process	Mitochondrion organization	5	4	246	0.7228
GO:0015672	IPI00686872;IPI00699372;IPI00703813;IPI00710920	Biological Process	Monovalent inorganic cation transport	7	4	246	0.2266
GO:0043066	IPI00690581;IPI00703084;IPI00709876;IPI01028455	Biological Process	Negative regulation of apoptotic process	9	4	246	0.9380
GO:0008285	IPI00693579;IPI00709876;IPI00710274;IPI00845178	Biological Process	Negative regulation of cell proliferation	5	4	246	0.8692

GO:0051129	IPI00712384;IPI00712858;IPI00716725;IPI00866975	Biological Process	Negative regulation of cellular component organization	6	4	246	0.6276
GO:0051346	IPI00698916;IPI00703925;IPI00867230;IPI01000210	Biological Process	Negative regulation of hydrolase activity	6	4	246	0.2897
GO:0043069	IPI00690581;IPI00703084;IPI00709876;IPI01028455	Biological Process	Negative regulation of programmed cell death	8	4	246	0.9465
GO:0051051	IPI00690309;IPI00715059;IPI00716725;IPI00866975	Biological Process	Negative regulation of transport	7	4	246	0.4199
GO:0030182	IPI00685510;IPI00716830;IPI00866975;IPI01003286	Biological Process	Neuron differentiation	9	4	246	0.7903
GO:0006334	IPI00689641;IPI00698339;IPI00705104;IPI00712399	Biological Process	Nucleosome assembly	12	4	246	0.0394
GO:0034728	IPI00689641;IPI00698339;IPI00705104;IPI00712399	Biological Process	Nucleosome organization	7	4	246	0.0928
GO:0051640	IPI00696116;IPI00718152;IPI00876805;IPI01028455	Biological Process	Organelle localization	5	4	246	0.4678
GO:0006518	IPI00685804;IPI00705565;IPI00876805;IPI01028182	Biological Process	Peptide metabolic process	7	4	246	0.2114
GO:0008654	IPI00685617;IPI00687661;IPI00703084;IPI00706879	Biological Process	Phospholipid biosynthetic process	6	4	246	0.1280
GO:0051130	IPI00690309;IPI00707857;IPI00710274;IPI00866858	Biological Process	Positive regulation of cellular component organization	6	4	246	0.8908
GO:0032388	IPI00696116;IPI00709876;IPI00714269;IPI01028468	Biological Process	Positive regulation of intracellular transport	7	4	246	0.1965
GO:0033674	IPI00685617;IPI00713597;IPI00715059;IPI00717764	Biological Process	Positive regulation of kinase activity	10	4	246	0.3384
GO:0045834	IPI00685617;IPI00690309;IPI00694430;IPI00715059	Biological Process	Positive regulation of lipid metabolic process	7	4	246	0.0823
GO:0043410	IPI00685617;IPI00697917;IPI00713597;IPI00717764	Biological Process	Positive regulation of MAPK cascade	11	4	246	0.1965

GO:0045944	IPI00685510;IPI00695037;IPI00704902;IPI00714898	Biological Process	Positive regulation of transcription from RNA polymerase II promoter	13	4	246	0.8225
GO:0051347	IPI00685617;IPI00713597;IPI00715059;IPI00717764	Biological Process	Positive regulation of transferase activity	6	4	246	0.3548
GO:0065004	IPI00689641;IPI00698339;IPI00705104;IPI00712399	Biological Process	Protein-DNA complex assembly	8	4	246	0.0394
GO:0071824	IPI00689641;IPI00698339;IPI00705104;IPI00712399	Biological Process	Protein-DNA complex subunit organization	6	4	246	0.0928
GO:0043112	IPI00709876;IPI00713474;IPI00866975;IPI00876805	Biological Process	Receptor metabolic process	6	4	246	0.0173
GO:0051726	IPI00685510;IPI00696748;IPI00698213;IPI00705237	Biological Process	Regulation of cell cycle	6	4	246	0.8371
GO:0001817	IPI00690581;IPI00703084;IPI00706879;IPI00713474	Biological Process	Regulation of cytokine production	6	4	246	0.3874
GO:0033157	IPI00690309;IPI00696116;IPI00714269;IPI01028468	Biological Process	Regulation of intracellular protein transport	7	4	246	0.3058
GO:0043269	IPI00693452;IPI00698916;IPI00705159;IPI00709876	Biological Process	Regulation of ion transport	6	4	246	0.2266
GO:0010959	IPI00693452;IPI00698916;IPI00705159;IPI00709876	Biological Process	Regulation of metal ion transport	8	4	246	0.1541
GO:0046822	IPI00690309;IPI00714269;IPI00906031;IPI01028468	Biological Process	Regulation of nucleocytoplasmic transport	8	4	246	0.2577
GO:0032101	IPI00690309;IPI00690581;IPI00698823;IPI00709876	Biological Process	Regulation of response to external stimulus	5	4	246	0.5730
GO:0032319	IPI00690309;IPI00705237;IPI00713049;IPI00866858	Biological Process	Regulation of Rho GTPase activity	16	4	246	0.0270
GO:0060627	IPI00694198;IPI00696116;IPI00716725;IPI00866975	Biological Process	Regulation of vesicle-mediated transport	5	4	246	0.4199
GO:0009607	IPI00690581;IPI00703084;IPI00710664;IPI00716157	Biological Process	Response to biotic stimulus	4	4	246	0.7535
GO:0051707	IPI00690581;IPI00703084;IPI00710664;IPI00716157	Biological Process	Response to other organism	6	4	246	0.7435

GO:0042594	IPI00698213;IPI00709876;IPI00714656;IPI01028455	Biological Process	Response to starvation	5	4	246	0.0134
GO:0048545	IPI00698823;IPI00703813;IPI00709876;IPI01028182	Biological Process	Response to steroid hormone	6	4	246	0.0394
GO:0032774	IPI00698213;IPI00704902;IPI00706142;IPI00712524	Biological Process	RNA biosynthetic process	8	4	246	0.9380
GO:0008380	IPI00687395;IPI00688337;IPI00700228;IPI00712263	Biological Process	RNA splicing	10	4	246	0.8299
GO:0032940	IPI00692417;IPI00696116;IPI00703084;IPI00717764	Biological Process	Secretion by cell	5	4	246	0.4360
GO:0007423	IPI00691826;IPI00710274;IPI00826022;IPI01003286	Biological Process	Sensory organ development	8	4	246	0.3221
GO:0016337	IPI00688400;IPI00708070;IPI00709888;IPI00713979	Biological Process	Single organismal cell-cell adhesion	6	4	246	0.4989
GO:0060538	IPI00685510;IPI00697648;IPI00709876;IPI00876805	Biological Process	Skeletal muscle organ development	9	4	246	0.0823
GO:0007519	IPI00685510;IPI00697648;IPI00709876;IPI00876805	Biological Process	Skeletal muscle tissue development	10	4	246	0.0823
GO:0008202	IPI00685510;IPI00690789;IPI00697194;IPI00698823	Biological Process	Steroid metabolic process	6	4	246	0.2577
GO:0007268	IPI00685510;IPI00696116;IPI00698059;IPI00876805	Biological Process	Synaptic transmission	7	4	246	0.2421
GO:0006351	IPI00698213;IPI00704902;IPI00706142;IPI00712524	Biological Process	Transcription, DNA-templated	10	4	246	0.9317
GO:0019226	IPI00685510;IPI00696116;IPI00698059;IPI00876805	Biological Process	Transmission of nerve impulse	6	4	246	0.4520
GO:0032147	IPI00685617;IPI00713597;IPI00717764	Biological Process	Activation of protein kinase activity	12	3	246	0.1605
GO:0002526	IPI00685653;IPI00700673;IPI00717930	Biological Process	Acute inflammatory response	7	3	246	0.0824
GO:0006953	IPI00685653;IPI00700673;IPI00717930	Biological Process	Acute-phase response	8	3	246	0.0574
GO:1901605	IPI00715066;IPI00760457;IPI00871114	Biological Process	Alpha-amino acid metabolic process	7	3	246	0.8287
GO:0043038	IPI00689365;IPI00708246;IPI00867017	Biological Process	Amino acid activation	7	3	246	0.3903

GO:0006022	IPI00699039;IPI00717764;IPI00717930	Biological Process	Aminoglycan metabolic process	6	3	246	0.0824
GO:0001525	IPI00709876;IPI00713049;IPI00839150	Biological Process	Angiogenesis	10	3	246	0.5031
GO:0006820	IPI00708597;IPI00710920;IPI00714656	Biological Process	Anion transport	6	3	246	0.3315
GO:0008015	IPI00705159;IPI00708244;IPI00709876	Biological Process	Blood circulation	7	3	246	0.4289
GO:0048514	IPI00709876;IPI00713049;IPI00839150	Biological Process	Blood vessel morphogenesis	9	3	246	0.7040
GO:0060348	IPI00702768;IPI00707776;IPI00707857	Biological Process	Bone development	8	3	246	0.0824
GO:0060349	IPI00702768;IPI00707776;IPI00707857	Biological Process	Bone morphogenesis	9	3	246	0.0367
GO:0007420	IPI00685510;IPI00687578;IPI00691826	Biological Process	Brain development	9	3	246	0.6198
GO:1901136	IPI00694430;IPI00710377;IPI00717764	Biological Process	Carbohydrate derivative catabolic process	6	3	246	0.8367
GO:0034330	IPI00705237;IPI00708070;IPI00710274	Biological Process	Cell junction organization	5	3	246	0.4289
GO:0000904	IPI00685510;IPI00866975;IPI00924039	Biological Process	Cell morphogenesis involved in differentiation	8	3	246	0.7404
GO:0032990	IPI00685510;IPI00687578;IPI00866975	Biological Process	Cell part morphogenesis	7	3	246	0.7932
GO:0048858	IPI00685510;IPI00687578;IPI00866975	Biological Process	Cell projection morphogenesis	6	3	246	0.6910
GO:0045454	IPI00687478;IPI00690581;IPI00867237	Biological Process	Cell redox homeostasis	6	3	246	0.3512
GO:0045216	IPI00705237;IPI00708070;IPI00710274	Biological Process	Cell-cell junction organization	6	3	246	0.2921
GO:0006974	IPI00696748;IPI00712399;IPI00718678	Biological Process	Cellular response to DNA damage stimulus	6	3	246	0.8900
GO:0071363	IPI00708244;IPI00712524;IPI00731432	Biological Process	Cellular response to growth factor stimulus	7	3	246	0.2921
GO:0032870	IPI00696116;IPI00698823;IPI00715059	Biological Process	Cellular response to hormone stimulus	7	3	246	0.3315
GO:0034599	IPI00690581;IPI00707455;IPI00716157	Biological Process	Cellular response to oxidative stress	6	3	246	0.3512
GO:0071560	IPI00708244;IPI00712524;IPI00731432	Biological Process	Cellular response to transforming growth factor beta stimulus	8	3	246	0.0574
GO:0046916	IPI00685510;IPI00709888;IPI00710664	Biological Process	Cellular transition metal ion homeostasis	10	3	246	0.1605

GO:0007417	IPI00685510;IPI00687578;IPI00691826	Biological Process	Central nervous system development	8	3	246	0.8116
GO:0006935	IPI00685510;IPI00697595;IPI00866858	Biological Process	Chemotaxis	5	3	246	0.4479
GO:0008203	IPI00685510;IPI00690789;IPI00697194	Biological Process	Cholesterol metabolic process	8	3	246	0.2149
GO:0016568	IPI00707776;IPI00718678;IPI00727591	Biological Process	Chromatin modification	7	3	246	0.5554
GO:0003013	IPI00705159;IPI00708244;IPI00709876	Biological Process	Circulatory system process	6	3	246	0.4289
GO:0051188	IPI00691093;IPI00695952;IPI00704332	Biological Process	Cofactor biosynthetic process	6	3	246	0.5554
GO:0016569	IPI00707776;IPI00718678;IPI00727591	Biological Process	Covalent chromatin modification	8	3	246	0.3315
GO:0006281	IPI00696748;IPI00712399;IPI00718678	Biological Process	DNA repair	9	3	246	0.6496
GO:0060350	IPI00702768;IPI00707776;IPI00707857	Biological Process	Endochondral bone morphogenesis	10	3	246	0.0207
GO:0015980	IPI00698213;IPI00730219;IPI00906031	Biological Process	Energy derivation by oxidation of organic compounds	6	3	246	0.7732
GO:0060562	IPI00699039;IPI00710920;IPI00713049	Biological Process	Epithelial tube morphogenesis	8	3	246	0.5209
GO:0006888	IPI00692963;IPI00695852;IPI00713460	Biological Process	ER to Golgi vesicle-mediated transport	7	3	246	0.1605
GO:0001654	IPI00691826;IPI00710274;IPI00826022	Biological Process	Eye development	9	3	246	0.2921
GO:0006633	IPI00694430;IPI00705565;IPI00714621	Biological Process	Fatty acid biosynthetic process	9	3	246	0.2921
GO:0048732	IPI00709876;IPI00710920;IPI00715059	Biological Process	Gland development	8	3	246	0.4479
GO:0042063	IPI00710274;IPI00713597;IPI00876805	Biological Process	Gliogenesis	8	3	246	0.2338
GO:0040007	IPI00685510;IPI00687578;IPI00710920	Biological Process	Growth	3	3	246	0.4850
GO:0048534	IPI00691093;IPI00705583;IPI00866975	Biological Process	Hematopoietic or lymphoid organ development	5	3	246	0.8518
GO:0030097	IPI00691093;IPI00705583;IPI00866975	Biological Process	Hemopoiesis	6	3	246	0.8203
GO:0016570	IPI00707776;IPI00718678;IPI00727591	Biological Process	Histone modification	8	3	246	0.3315
GO:0002520	IPI00691093;IPI00705583;IPI00866975	Biological Process	Immune system development	4	3	246	0.8783
GO:0055088	IPI00698823;IPI00703084;IPI00709876	Biological Process	Lipid homeostasis	7	3	246	0.1781

GO:0010876	IPI00694597;IPI00698823;IPI00709876	Biological Process	Lipid localization	5	3	246	0.4479
GO:0030879	IPI00709876;IPI00710920;IPI00715059	Biological Process	Mammary gland development	9	3	246	0.1781
GO:0007017	IPI00685510;IPI00687578;IPI00689379	Biological Process	Microtubule-based process	5	3	246	0.8444
GO:0006839	IPI00707815;IPI00711457;IPI00730219	Biological Process	Mitochondrial transport	6	3	246	0.2725
GO:0072330	IPI00694430;IPI00705565;IPI00714621	Biological Process	Monocarboxylic acid biosynthetic process	8	3	246	0.3512
GO:0002009	IPI00699039;IPI00710920;IPI00713049	Biological Process	Morphogenesis of an epithelium	6	3	246	0.7404
GO:0031124	IPI00685510;IPI00688446;IPI00704902	Biological Process	mRNA 3'-end processing	11	3	246	0.0465
GO:0032504	IPI00690309;IPI00709876;IPI00712399	Biological Process	Multicellular organism reproduction	4	3	246	0.8367
GO:0048609	IPI00690309;IPI00709876;IPI00712399	Biological Process	Multicellular organismal reproductive process	5	3	246	0.8287
GO:0055001	IPI00685510;IPI00697648;IPI00876805	Biological Process	Muscle cell development	8	3	246	0.3117
GO:0042692	IPI00685510;IPI00697648;IPI00876805	Biological Process	Muscle cell differentiation	7	3	246	0.5884
GO:0048747	IPI00685510;IPI00697648;IPI00876805	Biological Process	Muscle fiber development	10	3	246	0.1270
GO:0010507	IPI00690309;IPI00698823;IPI00713049	Biological Process	Negative regulation of autophagy	8	3	246	0.0058
GO:0009895	IPI00690309;IPI00698823;IPI00713049	Biological Process	Negative regulation of catabolic process	6	3	246	0.1113
GO:0030336	IPI00695042;IPI00845178;IPI00847122	Biological Process	Negative regulation of cell migration	9	3	246	0.2921
GO:2000146	IPI00695042;IPI00845178;IPI00847122	Biological Process	Negative regulation of cell motility	8	3	246	0.3117
GO:0031330	IPI00690309;IPI00698823;IPI00713049	Biological Process	Negative regulation of cellular catabolic process	7	3	246	0.0465
GO:0051271	IPI00695042;IPI00845178;IPI00847122	Biological Process	Negative regulation of cellular component movement	7	3	246	0.3512
GO:0032269	IPI00709876;IPI00710274;IPI00712384	Biological Process	Negative regulation of cellular protein metabolic process	8	3	246	0.8287
GO:0010629	IPI00698213;IPI00709876;IPI01003286	Biological Process	Negative regulation of gene expression	8	3	246	0.9846

GO:0040013	IPI00695042;IPI00845178;IPI00847122	Biological Process	Negative regulation of locomotion	5	3	246	0.3315
GO:0010923	IPI00698916;IPI00703925;IPI01000210	Biological Process	Negative regulation of phosphatase activity	10	3	246	0.0465
GO:0051248	IPI00709876;IPI00710274;IPI00712384	Biological Process	Negative regulation of protein metabolic process	7	3	246	0.9148
GO:0032102	IPI00690309;IPI00690581;IPI00698823	Biological Process	Negative regulation of response to external stimulus	6	3	246	0.0964
GO:0009968	IPI00709876;IPI00710274;IPI00845178	Biological Process	Negative regulation of signal transduction	8	3	246	0.9742
GO:0023057	IPI00709876;IPI00710274;IPI00845178	Biological Process	Negative regulation of signaling	5	3	246	0.9795
GO:0045892	IPI00698213;IPI00709876;IPI01003286	Biological Process	Negative regulation of transcription, DNA-templated	12	3	246	0.9677
GO:0048666	IPI00685510;IPI00716830;IPI00866975	Biological Process	Neuron development	10	3	246	0.8656
GO:0031175	IPI00685510;IPI00716830;IPI00866975	Biological Process	Neuron projection development	6	3	246	0.7518
GO:0051169	IPI00698213;IPI00700228;IPI00714269	Biological Process	Nuclear transport	7	3	246	0.6910
GO:0015931	IPI00689226;IPI00691086;IPI00700228	Biological Process	Nucleobase-containing compound transport	7	3	246	0.4666
GO:0006913	IPI00698213;IPI00700228;IPI00714269	Biological Process	Nucleocytoplasmic transport	8	3	246	0.6910
GO:0070925	IPI00687578;IPI00697648;IPI00704902	Biological Process	Organelle assembly	5	3	246	0.5031
GO:0018193	IPI00689629;IPI00690309;IPI00698916	Biological Process	Peptidyl-amino acid modification	8	3	246	0.8588
GO:0007009	IPI00691093;IPI00696116;IPI00843305	Biological Process	Plasma membrane organization	5	3	246	0.3512
GO:0050867	IPI00703084;IPI00705583;IPI00709876	Biological Process	Positive regulation of cell activation	7	3	246	0.3315
GO:0045597	IPI00690309;IPI00707776;IPI00707857	Biological Process	Positive regulation of cell differentiation	8	3	246	0.7166
GO:0043547	IPI00692668;IPI00705237;IPI00866858	Biological Process	Positive regulation of GTPase activity	15	3	246	0.5554
GO:0051345	IPI00692668;IPI00705237;IPI00866858	Biological Process	Positive regulation of hydrolase activity	6	3	246	0.9056
GO:0043123	IPI00709888;IPI00716830;IPI00852533	Biological Process	Positive regulation of I-kappaB kinase/NF-kappaB signaling	10	3	246	0.4289

GO:0002684	IPI00703084;IPI00705583;IPI00709876	Biological Process	Positive regulation of immune system process	5	3	246	0.8843
GO:0090316	IPI00696116;IPI00714269;IPI01028468	Biological Process	Positive regulation of intracellular protein transport	8	3	246	0.2530
GO:0002696	IPI00703084;IPI00705583;IPI00709876	Biological Process	Positive regulation of leukocyte activation	6	3	246	0.3315
GO:0051251	IPI00703084;IPI00705583;IPI00709876	Biological Process	Positive regulation of lymphocyte activation	7	3	246	0.2921
GO:0050731	IPI00685617;IPI00690309;IPI00713597	Biological Process	Positive regulation of peptidyl-tyrosine phosphorylation	10	3	246	0.0367
GO:0045860	IPI00685617;IPI00713597;IPI00717764	Biological Process	Positive regulation of protein kinase activity	11	3	246	0.4289
GO:0071902	IPI00685617;IPI00713597;IPI00717764	Biological Process	Positive regulation of protein serine/threonine kinase activity	12	3	246	0.2725
GO:0051222	IPI00696116;IPI00714269;IPI01028468	Biological Process	Positive regulation of protein transport	7	3	246	0.3512
GO:0032320	IPI00692668;IPI00705237;IPI00866858	Biological Process	Positive regulation of Ras GTPase activity	16	3	246	0.2338
GO:0050870	IPI00703084;IPI00705583;IPI00709876	Biological Process	Positive regulation of T cell activation	8	3	246	0.2338
GO:0051260	IPI00689899;IPI00709876;IPI00710834	Biological Process	Protein homooligomerization	9	3	246	0.6349
GO:0034504	IPI00707857;IPI00714269;IPI00715059	Biological Process	Protein localization to nucleus	8	3	246	0.3708
GO:0032446	IPI00706142;IPI00718678;IPI00825680	Biological Process	Protein modification by small protein conjugation	9	3	246	0.7627
GO:0070647	IPI00706142;IPI00718678;IPI00825680	Biological Process	Protein modification by small protein conjugation or removal	8	3	246	0.9191
GO:0006605	IPI00703954;IPI00707815;IPI00714269	Biological Process	Protein targeting	6	3	246	0.8900
GO:0016567	IPI00706142;IPI00718678;IPI00825680	Biological Process	Protein ubiquitination	10	3	246	0.6638
GO:0006898	IPI00706037;IPI00709876;IPI00866975	Biological Process	Receptor-mediated endocytosis	8	3	246	0.1270
GO:0032970	IPI00690309;IPI00708070;IPI00710274	Biological Process	Regulation of actin filament-based process	6	3	246	0.7166
GO:0045765	IPI00695042;IPI00712524;IPI00717764	Biological Process	Regulation of angiogenesis	11	3	246	0.2921

GO:0010506	IPI00690309;IPI00698823;IPI00713049	Biological Process	Regulation of autophagy	7	3	246	0.0694
GO:0051098	IPI00685510;IPI00707776;IPI00709876	Biological Process	Regulation of binding	5	3	246	0.3315
GO:0051924	IPI00693452;IPI00698916;IPI00709876	Biological Process	Regulation of calcium ion transport	10	3	246	0.1605
GO:0060828	IPI00707857;IPI00709876;IPI00845178	Biological Process	Regulation of canonical Wnt signaling pathway	10	3	246	0.0824
GO:0050865	IPI00703084;IPI00705583;IPI00709876	Biological Process	Regulation of cell activation	6	3	246	0.5884
GO:0060284	IPI00685510;IPI00690309;IPI00707857	Biological Process	Regulation of cell development	8	3	246	0.7627
GO:0044087	IPI00690309;IPI00710274;IPI00712384	Biological Process	Regulation of cellular component biogenesis	5	3	246	0.8721
GO:0010565	IPI00694430;IPI00709876;IPI00715059	Biological Process	Regulation of cellular ketone metabolic process	6	3	246	0.1270
GO:0080135	IPI00690309;IPI00697917;IPI00698823	Biological Process	Regulation of cellular response to stress	6	3	246	0.7040
GO:0051493	IPI00689379;IPI00690309;IPI00710274	Biological Process	Regulation of cytoskeleton organization	6	3	246	0.8203
GO:0035303	IPI00698916;IPI00703925;IPI01000210	Biological Process	Regulation of dephosphorylation	8	3	246	0.2530
GO:0030100	IPI00694198;IPI00716725;IPI00866975	Biological Process	Regulation of endocytosis	6	3	246	0.3708
GO:0019217	IPI00694430;IPI00709876;IPI00715059	Biological Process	Regulation of fatty acid metabolic process	10	3	246	0.0574
GO:0040008	IPI00685510;IPI00703084;IPI00707776	Biological Process	Regulation of growth	4	3	246	0.8287
GO:0008016	IPI00705159;IPI00708070;IPI00709876	Biological Process	Regulation of heart contraction	9	3	246	0.1113
GO:0032844	IPI00693842;IPI00703084;IPI00709876	Biological Process	Regulation of homeostatic process	5	3	246	0.3708
GO:0043122	IPI00709888;IPI00716830;IPI00852533	Biological Process	Regulation of I-kappaB kinase/NF-kappaB signaling	9	3	246	0.5384
GO:0002694	IPI00703084;IPI00705583;IPI00709876	Biological Process	Regulation of leukocyte activation	5	3	246	0.5384
GO:0051249	IPI00703084;IPI00705583;IPI00709876	Biological Process	Regulation of lymphocyte activation	6	3	246	0.4666
GO:0043405	IPI00685617;IPI00709876;IPI00713597	Biological Process	Regulation of MAP kinase activity	12	3	246	0.4097
GO:0006937	IPI00705159;IPI00708070;IPI00709876	Biological Process	Regulation of muscle contraction	8	3	246	0.0694
GO:0090257	IPI00705159;IPI00708070;IPI00709876	Biological Process	Regulation of muscle system process	7	3	246	0.1270

GO:0010921	IPI00698916;IPI00703925;IPI01000210	Biological Process	Regulation of phosphatase activity	9	3	246	0.1270
GO:0043393	IPI00685510;IPI00707776;IPI00709876	Biological Process	Regulation of protein binding	6	3	246	0.2149
GO:0051046	IPI00696116;IPI00703084;IPI00715059	Biological Process	Regulation of secretion	6	3	246	0.5721
GO:0044057	IPI00705159;IPI00708070;IPI00709876	Biological Process	Regulation of system process	6	3	246	0.6638
GO:0050863	IPI00703084;IPI00705583;IPI00709876	Biological Process	Regulation of T cell activation	7	3	246	0.3512
GO:1901342	IPI00695042;IPI00712524;IPI00717764	Biological Process	Regulation of vasculature development	8	3	246	0.3117
GO:0030111	IPI00707857;IPI00709876;IPI00845178	Biological Process	Regulation of Wnt signaling pathway	9	3	246	0.2338
GO:0009617	IPI00690581;IPI00710664;IPI00716157	Biological Process	Response to bacterium	7	3	246	0.6349
GO:0009743	IPI00690581;IPI00703084;IPI00760457	Biological Process	Response to carbohydrate	6	3	246	0.0574
GO:0034097	IPI00709124;IPI00717764;IPI00731432	Biological Process	Response to cytokine	6	3	246	0.6910
GO:0043627	IPI00703813;IPI00709876;IPI01028182	Biological Process	Response to estrogen	7	3	246	0.0281
GO:0009749	IPI00690581;IPI00703084;IPI00760457	Biological Process	Response to glucose	9	3	246	0.0465
GO:0070848	IPI00708244;IPI00712524;IPI00731432	Biological Process	Response to growth factor	6	3	246	0.3117
GO:0009746	IPI00690581;IPI00703084;IPI00760457	Biological Process	Response to hexose	8	3	246	0.0465
GO:0010035	IPI00709876;IPI00716157;IPI00730219	Biological Process	Response to inorganic substance	5	3	246	0.6776
GO:0032868	IPI00690309;IPI00696116;IPI00715059	Biological Process	Response to insulin	7	3	246	0.1781
GO:0009612	IPI00707857;IPI00710920;IPI00717764	Biological Process	Response to mechanical stimulus	5	3	246	0.1270
GO:0034284	IPI00690581;IPI00703084;IPI00760457	Biological Process	Response to monosaccharide	7	3	246	0.0465
GO:1901652	IPI00690309;IPI00696116;IPI00715059	Biological Process	Response to peptide	6	3	246	0.2725
GO:0043434	IPI00690309;IPI00696116;IPI00715059	Biological Process	Response to peptide hormone	6	3	246	0.2338
GO:0071559	IPI00708244;IPI00712524;IPI00731432	Biological Process	Response to transforming growth factor beta	5	3	246	0.0574
GO:0031123	IPI00685510;IPI00688446;IPI00704902	Biological Process	RNA 3'-end processing	10	3	246	0.1113

GO:0044702	IPI00685510;IPI00690309;IPI00712399	Biological Process	Single organism reproductive process	4	3	246	0.9006
GO:0048705	IPI00702768;IPI00707776;IPI00707857	Biological Process	Skeletal system morphogenesis	8	3	246	0.1113
GO:0016125	IPI00685510;IPI00690789;IPI00697194	Biological Process	Sterol metabolic process	7	3	246	0.2338
GO:0055002	IPI00685510;IPI00697648;IPI00876805	Biological Process	Striated muscle cell development	9	3	246	0.2921
GO:0051146	IPI00685510;IPI00697648;IPI00876805	Biological Process	Striated muscle cell differentiation	8	3	246	0.4666
GO:0006790	IPI00699039;IPI00705565;IPI01028182	Biological Process	Sulfur compound metabolic process	5	3	246	0.4666
GO:0042330	IPI00685510;IPI00697595;IPI00866858	Biological Process	Taxis	4	3	246	0.4479
GO:0055076	IPI00685510;IPI00709888;IPI00710664	Biological Process	Transition metal ion homeostasis	10	3	246	0.2921
GO:0043039	IPI00689365;IPI00708246;IPI00867017	Biological Process	tRNA aminoacylation	11	3	246	0.3903
GO:0006418	IPI00689365;IPI00708246;IPI00867017	Biological Process	tRNA aminoacylation for protein translation	12	3	246	0.3903
GO:0006399	IPI00689365;IPI00708246;IPI00867017	Biological Process	tRNA metabolic process	10	3	246	0.5721
GO:0035239	IPI00699039;IPI00710920;IPI00713049	Biological Process	Tube morphogenesis	7	3	246	0.5554
GO:0016050	IPI00709876;IPI00843305;IPI00866975	Biological Process	Vesicle organization	5	3	246	0.2530
GO:0031532	IPI00709888;IPI00924039	Biological Process	Actin cytoskeleton reorganization	7	2	246	0.2831
GO:0007015	IPI00697648;IPI00708767	Biological Process	Actin filament organization	7	2	246	0.7694
GO:0000187	IPI00685617;IPI00713597	Biological Process	Activation of MAPK activity	13	2	246	0.2296
GO:0006637	IPI00703084;IPI00871114	Biological Process	Acyl-CoA metabolic process	7	2	246	0.2831
GO:0006639	IPI00703084;IPI00709876	Biological Process	Acylglycerol metabolic process	7	2	246	0.3623
GO:0030534	IPI00685510;IPI00698823	Biological Process	Adult behavior	5	2	246	0.3362
GO:0008344	IPI00685510;IPI00698823	Biological Process	Adult locomotory behavior	5	2	246	0.2563
GO:1901607	IPI00715066;IPI00760457	Biological Process	Alpha-amino acid biosynthetic process	8	2	246	0.6317
GO:1901606	IPI00715066;IPI00871114	Biological Process	Alpha-amino acid catabolic process	8	2	246	0.5735
GO:0055081	IPI00703084;IPI00703813	Biological Process	Anion homeostasis	8	2	246	0.1268
GO:0015991	IPI00686872;IPI00699372	Biological Process	ATP hydrolysis coupled proton transport	11	2	246	0.2831
GO:0061564	IPI00685510;IPI00866975	Biological Process	Axon development	7	2	246	0.7147

GO:0007409	IPI00685510;IPI00866975	Biological Process	Axonogenesis	8	2	246	0.6670
GO:0007610	IPI00685510;IPI00698823	Biological Process	Behavior	3	2	246	0.8042
GO:0048754	IPI00710920;IPI00713049	Biological Process	Branching morphogenesis of an epithelial tube	7	2	246	0.3362
GO:0046395	IPI00715066;IPI00871114	Biological Process	Carboxylic acid catabolic process	7	2	246	0.9011
GO:0016024	IPI00703084;IPI00706879	Biological Process	CDP-diacylglycerol biosynthetic process	8	2	246	0.0424
GO:0046341	IPI00703084;IPI00706879	Biological Process	CDP-diacylglycerol metabolic process	9	2	246	0.0424
GO:0001775	IPI00698916;IPI00705583	Biological Process	Cell activation	5	2	246	0.9264
GO:0060326	IPI00697595;IPI00866858	Biological Process	Cell chemotaxis	8	2	246	0.3097
GO:0034329	IPI00705237;IPI00708070	Biological Process	Cell junction assembly	6	2	246	0.5526
GO:0048667	IPI00685510;IPI00866975	Biological Process	Cell morphogenesis involved in neuron differentiation	11	2	246	0.6995
GO:0007043	IPI00705237;IPI00708070	Biological Process	Cell-cell junction assembly	7	2	246	0.2831
GO:0031589	IPI00731432;IPI00924039	Biological Process	Cell-substrate adhesion	5	2	246	0.6497
GO:0008652	IPI00715066;IPI00760457	Biological Process	Cellular amino acid biosynthetic process	7	2	246	0.7567
GO:0009063	IPI00715066;IPI00871114	Biological Process	Cellular amino acid catabolic process	7	2	246	0.6130
GO:0044262	IPI00699039;IPI00906031	Biological Process	Cellular carbohydrate metabolic process	5	2	246	0.7694
GO:0010927	IPI00687578;IPI00697648	Biological Process	Cellular component assembly involved in morphogenesis	7	2	246	0.3881
GO:0006575	IPI00705565;IPI01028182	Biological Process	Cellular modified amino acid metabolic process	5	2	246	0.7433
GO:0044264	IPI00699039;IPI00906031	Biological Process	Cellular polysaccharide metabolic process	6	2	246	0.4134
GO:0044257	IPI00690581;IPI00704041	Biological Process	Cellular protein catabolic process	7	2	246	0.9906
GO:0045333	IPI00698213;IPI00730219	Biological Process	Cellular respiration	7	2	246	0.7931
GO:0042149	IPI00698213;IPI00714656	Biological Process	Cellular response to glucose starvation	9	2	246	0.0424
GO:0032869	IPI00696116;IPI00715059	Biological Process	Cellular response to insulin stimulus	9	2	246	0.2831
GO:0071396	IPI00690581;IPI00698823	Biological Process	Cellular response to lipid	7	2	246	0.6130
GO:0071407	IPI00698823;IPI00702768	Biological Process	Cellular response to organic cyclic compound	7	2	246	0.5311
GO:0071453	IPI00690309;IPI00709876	Biological Process	Cellular response to oxygen levels	6	2	246	0.1515

GO:1901653	IPI00696116;IPI 00715059	Biological Process	Cellular response to peptide	8	2	246	0.3362
GO:0071375	IPI00696116;IPI 00715059	Biological Process	Cellular response to peptide hormone stimulus	8	2	246	0.3362
GO:0042632	IPI00698823;IPI 00709876	Biological Process	Cholesterol homeostasis	9	2	246	0.2831
GO:0007059	IPI00693579;IPI 00712858	Biological Process	Chromosome segregation	5	2	246	0.3362
GO:0015936	IPI00694430;IPI 00704332	Biological Process	Coenzyme A metabolic process	12	2	246	0.0811
GO:0009108	IPI00695952;IPI 00704332	Biological Process	Coenzyme biosynthetic process	7	2	246	0.6836
GO:0032964	IPI00707857;IPI 00731432	Biological Process	Collagen biosynthetic process	6	2	246	0.0424
GO:0032963	IPI00707857;IPI 00731432	Biological Process	Collagen metabolic process	7	2	246	0.1032
GO:0001816	IPI00703084;IPI 00717764	Biological Process	Cytokine production	5	2	246	0.2296
GO:0050663	IPI00703084;IPI 00717764	Biological Process	Cytokine secretion	7	2	246	0.0424
GO:0042742	IPI00710664;IPI 00716157	Biological Process	Defense response to bacterium	7	2	246	0.3623
GO:0016358	IPI00685510;IPI 00866975	Biological Process	Dendrite development	7	2	246	0.2031
GO:0048589	IPI00685510;IPI 00710920	Biological Process	Developmental growth	5	2	246	0.4134
GO:0060560	IPI00685510;IPI 00710920	Biological Process	Developmental growth involved in morphogenesis	5	2	246	0.1770
GO:0003006	IPI00690309;IPI 00702768	Biological Process	Developmental process involved in reproduction	4	2	246	0.8147
GO:0048598	IPI00699039;IPI 00710274	Biological Process	Embryonic morphogenesis	7	2	246	0.8952
GO:0001958	IPI00702768;IPI 00707857	Biological Process	Endochondral ossification	7	2	246	0.0607
GO:0007032	IPI00694198;IPI 00713474	Biological Process	Endosome organization	5	2	246	0.1770
GO:0015988	IPI00686872;IPI 00699372	Biological Process	Energy coupled proton transmembrane transport, against electrochemical gradient	10	2	246	0.2831
GO:0008544	IPI00709888;IPI 01003286	Biological Process	Epidermis development	9	2	246	0.6670
GO:0051656	IPI00696116;IPI 00718152	Biological Process	Establishment of organelle localization	5	2	246	0.7816
GO:0090150	IPI00696116;IPI 00703954	Biological Process	Establishment of protein localization to membrane	6	2	246	0.6497
GO:0072594	IPI00707815;IPI 00714269	Biological Process	Establishment of protein localization to organelle	8	2	246	0.9171
GO:0051236	IPI00689226;IPI 00700228	Biological Process	Establishment of RNA localization	6	2	246	0.6836
GO:0051650	IPI00696116;IPI 00718152	Biological Process	Establishment of vesicle localization	6	2	246	0.4859
GO:0006887	IPI00692417;IPI 00696116	Biological Process	Exocytosis	6	2	246	0.7147

GO:1901570	IPI00705565;IPI 00714621	Biological Process	Fatty acid derivative biosynthetic process	6	2	246	0.1770
GO:1901568	IPI00705565;IPI 00714621	Biological Process	Fatty acid derivative metabolic process	5	2	246	0.3097
GO:0030900	IPI00685510;IPI 00687578	Biological Process	Forebrain development	10	2	246	0.6670
GO:0007276	IPI00690309;IPI 00712399	Biological Process	Gamete generation	5	2	246	0.8343
GO:0022612	IPI00709876;IPI 00710920	Biological Process	Gland morphogenesis	9	2	246	0.3881
GO:0006749	IPI00705565;IPI 01028182	Biological Process	Glutathione metabolic process	8	2	246	0.2831
GO:0009101	IPI00698823;IPI 00699039	Biological Process	Glycoprotein biosynthetic process	7	2	246	0.6670
GO:0009100	IPI00698823;IPI 00699039	Biological Process	Glycoprotein metabolic process	6	2	246	0.7816
GO:0030203	IPI00699039;IPI 00717930	Biological Process	Glycosaminoglycan metabolic process	7	2	246	0.2031
GO:1901658	IPI00694430;IPI 00710377	Biological Process	Glycosyl compound catabolic process	6	2	246	0.8601
GO:0006893	IPI00696116;IPI 00713474	Biological Process	Golgi to plasma membrane transport	8	2	246	0.2031
GO:0007507	IPI00708070;IPI 00731432	Biological Process	Heart development	8	2	246	0.8753
GO:0006818	IPI00686872;IPI 00699372	Biological Process	Hydrogen transport	5	2	246	0.5311
GO:0046456	IPI00705565;IPI 00714621	Biological Process	Icosanoid biosynthetic process	8	2	246	0.1770
GO:0006690	IPI00705565;IPI 00714621	Biological Process	Icosanoid metabolic process	8	2	246	0.3097
GO:0002252	IPI00703084;IPI 00705583	Biological Process	Immune effector process	4	2	246	0.9011
GO:0007229	IPI00697595;IPI 00731432	Biological Process	Integrin-mediated signaling pathway	8	2	246	0.2563
GO:0051701	IPI00713313;IPI 01028455	Biological Process	Interaction with host	6	2	246	0.2831
GO:0044419	IPI00713313;IPI 01028455	Biological Process	Interspecies interaction between organisms	4	2	246	0.5526
GO:0034220	IPI00686872;IPI 00699372	Biological Process	Ion transmembrane transport	6	2	246	0.6497
GO:0045321	IPI00698916;IPI 00705583	Biological Process	Leukocyte activation	4	2	246	0.8519
GO:0006869	IPI00694597;IPI 00698823	Biological Process	Lipid transport	5	2	246	0.6317
GO:0007626	IPI00685510;IPI 00698823	Biological Process	Locomotory behavior	4	2	246	0.4859
GO:0030324	IPI00717764;IPI 01003286	Biological Process	Lung development	8	2	246	0.4381
GO:0046649	IPI00698916;IPI 00705583	Biological Process	Lymphocyte activation	5	2	246	0.7567
GO:0031294	IPI00705583;IPI 00709876	Biological Process	Lymphocyte costimulation	4	2	246	0.0424
GO:0060443	IPI00709876;IPI 00710920	Biological Process	Mammary gland morphogenesis	10	2	246	0.1770
GO:0061025	IPI00696116;IPI 00843305	Biological Process	Membrane fusion	6	2	246	0.4381

GO:0000226	IPI00687578;IPI00689379	Biological Process	Microtubule cytoskeleton organization	6	2	246	0.8042
GO:0033108	IPI00688474;IPI00689365	Biological Process	Mitochondrial respiratory chain complex assembly	6	2	246	0.2563
GO:0000278	IPI00685510;IPI00693579	Biological Process	Mitotic cell cycle	6	2	246	0.8433
GO:0044003	IPI00713313;IPI01028455	Biological Process	Modification by symbiont of host morphology or physiology	6	2	246	0.2031
GO:0035821	IPI00713313;IPI01028455	Biological Process	Modification of morphology or physiology of other organism	4	2	246	0.3623
GO:0051817	IPI00713313;IPI01028455	Biological Process	Modification of morphology or physiology of other organism involved in symbiotic interaction	5	2	246	0.3097
GO:0043632	IPI00690581;IPI00704041	Biological Process	Modification-dependent macromolecule catabolic process	7	2	246	0.9756
GO:0019941	IPI00690581;IPI00704041	Biological Process	Modification-dependent protein catabolic process	8	2	246	0.9740
GO:0061138	IPI00710920;IPI00713049	Biological Process	Morphogenesis of a branching epithelium	6	2	246	0.4134
GO:0001763	IPI00710920;IPI00713049	Biological Process	Morphogenesis of a branching structure	5	2	246	0.4134
GO:0000398	IPI00687395;IPI00700228	Biological Process	mRNA splicing, via spliceosome	11	2	246	0.6130
GO:0051028	IPI00689226;IPI00700228	Biological Process	mRNA transport	8	2	246	0.5936
GO:0035264	IPI00687578;IPI00710920	Biological Process	Multicellular organism growth	5	2	246	0.2031
GO:0044259	IPI00707857;IPI00731432	Biological Process	Multicellular organismal macromolecule metabolic process	6	2	246	0.1268
GO:0044236	IPI00707857;IPI00731432	Biological Process	Multicellular organismal metabolic process	5	2	246	0.1515
GO:0051100	IPI00707776;IPI00709876	Biological Process	Negative regulation of binding	6	2	246	0.2563
GO:0090090	IPI00709876;IPI00845178	Biological Process	Negative regulation of canonical Wnt signaling pathway	11	2	246	0.1770
GO:0007162	IPI00707857;IPI00710274	Biological Process	Negative regulation of cell adhesion	5	2	246	0.4381
GO:0045806	IPI00716725;IPI00866975	Biological Process	Negative regulation of endocytosis	7	2	246	0.1770
GO:0050680	IPI00709876;IPI00845178	Biological Process	Negative regulation of epithelial cell proliferation	6	2	246	0.4381

GO:0050777	IPI00690581;IPI00731432	Biological Process	Negative regulation of immune response	6	2	246	0.2031
GO:0002683	IPI00690581;IPI00731432	Biological Process	Negative regulation of immune system process	5	2	246	0.6130
GO:0046426	IPI00709876;IPI00710274	Biological Process	Negative regulation of JAK-STAT cascade	10	2	246	0.0424
GO:0033673	IPI00709876;IPI00710274	Biological Process	Negative regulation of kinase activity	10	2	246	0.6130
GO:0016242	IPI00690309;IPI00698823	Biological Process	Negative regulation of macroautophagy	9	2	246	0.0266
GO:0043409	IPI00709876;IPI00710274	Biological Process	Negative regulation of MAPK cascade	11	2	246	0.5311
GO:0051241	IPI00690581;IPI00705159	Biological Process	Negative regulation of multicellular organismal process	5	2	246	0.8753
GO:0050732	IPI00709876;IPI00710274	Biological Process	Negative regulation of peptidyl-tyrosine phosphorylation	10	2	246	0.1032
GO:0045936	IPI00709876;IPI00710274	Biological Process	Negative regulation of phosphate metabolic process	8	2	246	0.8042
GO:0010563	IPI00709876;IPI00710274	Biological Process	Negative regulation of phosphorus metabolic process	6	2	246	0.8042
GO:0042326	IPI00709876;IPI00710274	Biological Process	Negative regulation of phosphorylation	9	2	246	0.7293
GO:0032091	IPI00707776;IPI00709876	Biological Process	Negative regulation of protein binding	7	2	246	0.2031
GO:0006469	IPI00709876;IPI00710274	Biological Process	Negative regulation of protein kinase activity	11	2	246	0.5735
GO:0031400	IPI00709876;IPI00710274	Biological Process	Negative regulation of protein modification process	9	2	246	0.8601
GO:0001933	IPI00709876;IPI00710274	Biological Process	Negative regulation of protein phosphorylation	10	2	246	0.6995
GO:0032105	IPI00690309;IPI00698823	Biological Process	Negative regulation of response to extracellular stimulus	7	2	246	0.0266
GO:0032108	IPI00690309;IPI00698823	Biological Process	Negative regulation of response to nutrient levels	8	2	246	0.0266
GO:0033119	IPI00694688;IPI00712263	Biological Process	Negative regulation of RNA splicing	11	2	246	0.1770

GO:0000122	IPI00709876;IPI01003286	Biological Process	Negative regulation of transcription from RNA polymerase II promoter	12	2	246	0.8601
GO:0051348	IPI00709876;IPI00710274	Biological Process	Negative regulation of transferase activity	6	2	246	0.6670
GO:0042532	IPI00709876;IPI00710274	Biological Process	Negative regulation of tyrosine phosphorylation of STAT protein	11	2	246	0.0266
GO:0042524	IPI00709876;IPI00710274	Biological Process	Negative regulation of tyrosine phosphorylation of Stat5 protein	12	2	246	0.0266
GO:0030178	IPI00709876;IPI00845178	Biological Process	Negative regulation of Wnt signaling pathway	10	2	246	0.3097
GO:0048812	IPI00685510;IPI00866975	Biological Process	Neuron projection morphogenesis	7	2	246	0.7147
GO:0006836	IPI00696116;IPI00714660	Biological Process	Neurotransmitter transport	5	2	246	0.2296
GO:0006638	IPI00703084;IPI00709876	Biological Process	Neutral lipid metabolic process	6	2	246	0.3623
GO:0050657	IPI00689226;IPI00700228	Biological Process	Nucleic acid transport	8	2	246	0.6836
GO:0033865	IPI00694430;IPI00704332	Biological Process	Nucleoside bisphosphate metabolic process	9	2	246	0.1032
GO:0009164	IPI00694430;IPI00710377	Biological Process	Nucleoside catabolic process	8	2	246	0.8433
GO:1901292	IPI00694430;IPI00710377	Biological Process	Nucleoside phosphate catabolic process	8	2	246	0.8679
GO:0009166	IPI00694430;IPI00710377	Biological Process	Nucleotide catabolic process	9	2	246	0.8519
GO:0042476	IPI00707857;IPI00710274	Biological Process	Odontogenesis	9	2	246	0.1515
GO:0016054	IPI00715066;IPI00871114	Biological Process	Organic acid catabolic process	6	2	246	0.9011
GO:0015711	IPI00708597;IPI00714656	Biological Process	Organic anion transport	7	2	246	0.4859
GO:0046434	IPI00694430;IPI00710377	Biological Process	Organophosphate catabolic process	6	2	246	0.9264
GO:0001503	IPI00702768;IPI00707857	Biological Process	Ossification	5	2	246	0.3097
GO:0018208	IPI00689629;IPI00698916	Biological Process	Peptidyl-proline modification	9	2	246	0.2031
GO:0006661	IPI00685617;IPI00687661	Biological Process	Phosphatidylinositol biosynthetic process	8	2	246	0.1770
GO:0046488	IPI00685617;IPI00687661	Biological Process	Phosphatidylinositol metabolic process	9	2	246	0.5526
GO:0016310	IPI00685510;IPI00690309	Biological Process	Phosphorylation	7	2	246	0.9811
GO:0005976	IPI00699039;IPI00906031	Biological Process	Polysaccharide metabolic process	6	2	246	0.4134
GO:0032233	IPI00690309;IPI00710274	Biological Process	Positive regulation of actin filament bundle assembly	11	2	246	0.1032

GO:0045766	IPI00695042;IPI 00717764	Biological Process	Positive regulation of angiogenesis	12	2	246	0.3623
GO:0051928	IPI00693452;IPI 00709876	Biological Process	Positive regulation of calcium ion transport	11	2	246	0.1515
GO:0090263	IPI00707857;IPI 00709876	Biological Process	Positive regulation of canonical Wnt signaling pathway	11	2	246	0.0424
GO:0009896	IPI00715059;IPI 00716725	Biological Process	Positive regulation of catabolic process	6	2	246	0.5735
GO:0090068	IPI00698213;IPI 00705237	Biological Process	Positive regulation of cell cycle process	8	2	246	0.2296
GO:0030335	IPI00707857;IPI 00715059	Biological Process	Positive regulation of cell migration	9	2	246	0.7433
GO:2000147	IPI00707857;IPI 00715059	Biological Process	Positive regulation of cell motility	8	2	246	0.7816
GO:0031331	IPI00715059;IPI 00716725	Biological Process	Positive regulation of cellular catabolic process	7	2	246	0.5311
GO:0051272	IPI00707857;IPI 00715059	Biological Process	Positive regulation of cellular component movement	7	2	246	0.7931
GO:0001819	IPI00706879;IPI 00713474	Biological Process	Positive regulation of cytokine production	7	2	246	0.4134
GO:0051495	IPI00690309;IPI 00710274	Biological Process	Positive regulation of cytoskeleton organization	7	2	246	0.4381
GO:0045923	IPI00694430;IPI 00715059	Biological Process	Positive regulation of fatty acid metabolic process	11	2	246	0.1032
GO:0046321	IPI00694430;IPI 00715059	Biological Process	Positive regulation of fatty acid oxidation	12	2	246	0.0424
GO:0043270	IPI00693452;IPI 00709876	Biological Process	Positive regulation of ion transport	7	2	246	0.2031
GO:0090218	IPI00685617;IPI 00715059	Biological Process	Positive regulation of lipid kinase activity	11	2	246	0.1268
GO:0040017	IPI00707857;IPI 00715059	Biological Process	Positive regulation of locomotion	5	2	246	0.7931
GO:0043406	IPI00685617;IPI 00713597	Biological Process	Positive regulation of MAP kinase activity	13	2	246	0.3623
GO:0046824	IPI00714269;IPI 01028468	Biological Process	Positive regulation of nucleocyto- plasmic transport	9	2	246	0.3881
GO:0010638	IPI00690309;IPI 00710274	Biological Process	Positive regulation of organelle organization	5	2	246	0.8343

GO:0032321	IPI00705237;IPI00866858	Biological Process	Positive regulation of Rho GTPase activity	17	2	246	0.1268
GO:0051496	IPI00690309;IPI00710274	Biological Process	Positive regulation of stress fiber assembly	10	2	246	0.0811
GO:0030177	IPI00707857;IPI00709876	Biological Process	Positive regulation of Wnt signaling pathway	10	2	246	0.1515
GO:0006892	IPI00696116;IPI00713474	Biological Process	Post-Golgi vesicle-mediated transport	7	2	246	0.2296
GO:0010608	IPI00685510;IPI00712384	Biological Process	Posttranscriptional regulation of gene expression	8	2	246	0.9871
GO:0030163	IPI00690581;IPI00704041	Biological Process	Protein catabolic process	7	2	246	0.9957
GO:0051291	IPI00707857;IPI00708244	Biological Process	Protein heterooligomerization	9	2	246	0.1770
GO:0070208	IPI00707857;IPI00708244	Biological Process	Protein heterotrimerization	10	2	246	0.0811
GO:0072657	IPI00696116;IPI00703954	Biological Process	Protein localization to membrane	5	2	246	0.8042
GO:0051604	IPI00685804;IPI00707815	Biological Process	Protein maturation	6	2	246	0.3097
GO:0000413	IPI00689629;IPI00698916	Biological Process	Protein peptidyl-prolyl isomerization	10	2	246	0.1770
GO:0006468	IPI00685510;IPI00690309	Biological Process	Protein phosphorylation	8	2	246	0.9011
GO:0009306	IPI00703084;IPI00717764	Biological Process	Protein secretion	6	2	246	0.1268
GO:0070206	IPI00707857;IPI00708244	Biological Process	Protein trimerization	9	2	246	0.1515
GO:0051603	IPI00690581;IPI00704041	Biological Process	Proteolysis involved in cellular protein catabolic process	8	2	246	0.9886
GO:0015992	IPI00686872;IPI00699372	Biological Process	Proton transport	8	2	246	0.5311
GO:0034032	IPI00694430;IPI00704332	Biological Process	Purine nucleoside bisphosphate metabolic process	10	2	246	0.1032
GO:0006152	IPI00694430;IPI00710377	Biological Process	Purine nucleoside catabolic process	9	2	246	0.8247
GO:0006195	IPI00694430;IPI00710377	Biological Process	Purine nucleotide catabolic process	10	2	246	0.8147
GO:0046130	IPI00694430;IPI00710377	Biological Process	Purine ribonucleoside catabolic process	10	2	246	0.8247
GO:0009154	IPI00694430;IPI00710377	Biological Process	Purine ribonucleotide catabolic process	11	2	246	0.8042
GO:0072523	IPI00694430;IPI00710377	Biological Process	Purine-containing compound catabolic process	7	2	246	0.8519
GO:0007265	IPI00708244;IPI00847122	Biological Process	Ras protein signal transduction	9	2	246	0.3623
GO:0031623	IPI00709876;IPI00866975	Biological Process	Receptor internalization	7	2	246	0.1268

GO:0032956	IPI00690309;IPI00710274	Biological Process	Regulation of actin cytoskeleton organization	7	2	246	0.8247
GO:0032231	IPI00690309;IPI00710274	Biological Process	Regulation of actin filament bundle assembly	10	2	246	0.1032
GO:0090066	IPI00690309;IPI00709876	Biological Process	Regulation of anatomical structure size	5	2	246	0.8753
GO:2001233	IPI00709876;IPI00712858	Biological Process	Regulation of apoptotic signaling pathway	8	2	246	0.5735
GO:0032012	IPI00688451;IPI00713474	Biological Process	Regulation of ARF protein signal transduction	11	2	246	0.3097
GO:0008217	IPI00705159;IPI00708244	Biological Process	Regulation of blood pressure	8	2	246	0.3362
GO:0006109	IPI00694430;IPI00715059	Biological Process	Regulation of carbohydrate metabolic process	6	2	246	0.3362
GO:0030155	IPI00707857;IPI00710274	Biological Process	Regulation of cell adhesion	5	2	246	0.8679
GO:0010564	IPI00698213;IPI00705237	Biological Process	Regulation of cell cycle process	7	2	246	0.8343
GO:0010675	IPI00694430;IPI00715059	Biological Process	Regulation of cellular carbohydrate metabolic process	6	2	246	0.3097
GO:0051052	IPI00693842;IPI00710274	Biological Process	Regulation of DNA metabolic process	9	2	246	0.5936
GO:0050678	IPI00709876;IPI00845178	Biological Process	Regulation of epithelial cell proliferation	6	2	246	0.6130
GO:0046320	IPI00694430;IPI00715059	Biological Process	Regulation of fatty acid oxidation	11	2	246	0.0811
GO:0010906	IPI00694430;IPI00715059	Biological Process	Regulation of glucose metabolic process	7	2	246	0.2296
GO:0050776	IPI00690581;IPI00731432	Biological Process	Regulation of immune response	5	2	246	0.9599
GO:0046425	IPI00709876;IPI00710274	Biological Process	Regulation of JAK-STAT cascade	9	2	246	0.1515
GO:0046890	IPI00690309;IPI00705159	Biological Process	Regulation of lipid biosynthetic process	7	2	246	0.3881
GO:0043550	IPI00685617;IPI00715059	Biological Process	Regulation of lipid kinase activity	10	2	246	0.1515
GO:0016241	IPI00690309;IPI00698823	Biological Process	Regulation of macroautophagy	8	2	246	0.1032
GO:0042391	IPI00708070;IPI00709876	Biological Process	Regulation of membrane potential	5	2	246	0.7433
GO:0040014	IPI00685510;IPI00703084	Biological Process	Regulation of multicellular organism growth	6	2	246	0.4134
GO:0051147	IPI00690309;IPI00712263	Biological Process	Regulation of muscle cell differentiation	8	2	246	0.1032
GO:0001505	IPI00696116;IPI00698059	Biological Process	Regulation of neurotransmitter levels	5	2	246	0.2831

GO:0042306	IPI00690309;IPI00714269	Biological Process	Regulation of protein import into nucleus	9	2	246	0.4623
GO:1900180	IPI00690309;IPI00714269	Biological Process	Regulation of protein localization to nucleus	6	2	246	0.4859
GO:0061097	IPI00685510;IPI00847122	Biological Process	Regulation of protein tyrosine kinase activity	10	2	246	0.0607
GO:0030162	IPI00707685;IPI00716725	Biological Process	Regulation of proteolysis	7	2	246	0.8042
GO:0032104	IPI00690309;IPI00698823	Biological Process	Regulation of response to extracellular stimulus	6	2	246	0.1268
GO:0032107	IPI00690309;IPI00698823	Biological Process	Regulation of response to nutrient levels	7	2	246	0.1268
GO:0043484	IPI00694688;IPI00712263	Biological Process	Regulation of RNA splicing	11	2	246	0.5735
GO:0051492	IPI00690309;IPI00710274	Biological Process	Regulation of stress fiber assembly	9	2	246	0.1032
GO:0006942	IPI00705159;IPI00708070	Biological Process	Regulation of striated muscle contraction	9	2	246	0.1032
GO:0002026	IPI00705159;IPI00709876	Biological Process	Regulation of the force of heart contraction	10	2	246	0.1268
GO:0006417	IPI00685510;IPI00712384	Biological Process	Regulation of translation	8	2	246	0.8679
GO:0032680	IPI00690581;IPI00713474	Biological Process	Regulation of tumor necrosis factor production	8	2	246	0.2831
GO:0042509	IPI00709876;IPI00710274	Biological Process	Regulation of tyrosine phosphorylation of STAT protein	10	2	246	0.0811
GO:0042522	IPI00709876;IPI00710274	Biological Process	Regulation of tyrosine phosphorylation of Stat5 protein	11	2	246	0.0266
GO:0036075	IPI00702768;IPI00707857	Biological Process	Replacement ossification	6	2	246	0.0607
GO:0022904	IPI00698213;IPI00730219	Biological Process	Respiratory electron transport chain	8	2	246	0.3881
GO:0060541	IPI00717764;IPI01003286	Biological Process	Respiratory system development	7	2	246	0.4381
GO:0030323	IPI00717764;IPI01003286	Biological Process	Respiratory tube development	7	2	246	0.4381
GO:0046677	IPI00702768;IPI00709124	Biological Process	Response to antibiotic	6	2	246	0.0811
GO:0051592	IPI00709876;IPI00730219	Biological Process	Response to calcium ion	7	2	246	0.4381
GO:0036293	IPI00690309;IPI00709876	Biological Process	Response to decreased oxygen levels	6	2	246	0.4859
GO:0042493	IPI00698823;IPI00705159	Biological Process	Response to drug	5	2	246	0.5735
GO:0001666	IPI00690309;IPI00709876	Biological Process	Response to hypoxia	5	2	246	0.4859
GO:0010038	IPI00709876;IPI00730219	Biological Process	Response to metal ion	6	2	246	0.6497

GO:0070482	IPI00690309;IPI00709876	Biological Process	Response to oxygen levels	5	2	246	0.5311
GO:0009314	IPI00685510;IPI00731432	Biological Process	Response to radiation	5	2	246	0.7816
GO:0009636	IPI00702768;IPI00709124	Biological Process	Response to toxic substance	5	2	246	0.2296
GO:0034612	IPI00709124;IPI00717764	Biological Process	Response to tumor necrosis factor	7	2	246	0.3881
GO:0022613	IPI00702948;IPI00704902	Biological Process	Ribonucleoprotein complex biogenesis	5	2	246	0.8753
GO:0033875	IPI00694430;IPI00704332	Biological Process	Ribonucleoside bisphosphate metabolic process	10	2	246	0.1032
GO:0042454	IPI00694430;IPI00710377	Biological Process	Ribonucleoside catabolic process	9	2	246	0.8247
GO:0009261	IPI00694430;IPI00710377	Biological Process	Ribonucleotide catabolic process	10	2	246	0.8042
GO:0006401	IPI00704902;IPI01017508	Biological Process	RNA catabolic process	8	2	246	0.4134
GO:0006403	IPI00689226;IPI00700228	Biological Process	RNA localization	5	2	246	0.7147
GO:0000375	IPI00687395;IPI00700228	Biological Process	RNA splicing, via transesterification reactions	11	2	246	0.6317
GO:0000377	IPI00687395;IPI00700228	Biological Process	RNA splicing, via transesterification reactions with bulged adenosine as nucleophile	12	2	246	0.6130
GO:0050658	IPI00689226;IPI00700228	Biological Process	RNA transport	7	2	246	0.6836
GO:0016072	IPI00702948;IPI01017508	Biological Process	rRNA metabolic process	10	2	246	0.5311
GO:0007600	IPI00707857;IPI00710920	Biological Process	Sensory perception	7	2	246	0.4859
GO:0050954	IPI00707857;IPI00710920	Biological Process	Sensory perception of mechanical stimulus	8	2	246	0.2031
GO:0007605	IPI00707857;IPI00710920	Biological Process	Sensory perception of sound	9	2	246	0.2031
GO:0019953	IPI00690309;IPI00712399	Biological Process	Sexual reproduction	4	2	246	0.9264
GO:0044708	IPI00685510;IPI00698823	Biological Process	Single-organism behavior	4	2	246	0.7147
GO:0044723	IPI00698823;IPI00699039	Biological Process	Single-organism carbohydrate metabolic process	6	2	246	0.9979
GO:0044712	IPI00715066;IPI00871114	Biological Process	Single-organism catabolic process	5	2	246	0.9488
GO:0048741	IPI00685510;IPI00697648	Biological Process	Skeletal muscle fiber development	11	2	246	0.1770
GO:0043589	IPI00707857;IPI00708244	Biological Process	Skin morphogenesis	9	2	246	0.1032
GO:0044282	IPI00715066;IPI00871114	Biological Process	Small molecule catabolic process	6	2	246	0.9488
GO:0006694	IPI00690789;IPI00697194	Biological Process	Steroid biosynthetic process	7	2	246	0.3623
GO:0055092	IPI00698823;IPI00709876	Biological Process	sterol homeostasis	8	2	246	0.2831

GO:0044403	IPI00713313;IPI01028455	Biological Process	Symbiosis, encompassing mutualism through parasitism	5	2	246	0.5526
GO:0031295	IPI00705583;IPI00709876	Biological Process	T cell costimulation	5	2	246	0.0424
GO:0035383	IPI00703084;IPI00871114	Biological Process	Thioester metabolic process	6	2	246	0.2831
GO:0007179	IPI00708244;IPI00731432	Biological Process	Transforming growth factor beta receptor signaling pathway	10	2	246	0.1268
GO:0007178	IPI00708244;IPI00731432	Biological Process	Transmembrane receptor protein serine/threonine kinase signaling pathway	9	2	246	0.2031
GO:0007169	IPI00713597;IPI00715059	Biological Process	Transmembrane receptor protein tyrosine kinase signaling pathway	9	2	246	0.5735
GO:0006641	IPI00703084;IPI00709876	Biological Process	Triglyceride metabolic process	8	2	246	0.2831
GO:0006511	IPI00690581;IPI00704041	Biological Process	Ubiquitin-dependent protein catabolic process	9	2	246	0.9687
GO:0006636	IPI00705565;IPI00714621	Biological Process	Unsaturated fatty acid biosynthetic process	10	2	246	0.1770
GO:0033559	IPI00705565;IPI00714621	Biological Process	Unsaturated fatty acid metabolic process	10	2	246	0.3097
GO:0001570	IPI00709876;IPI00713049	Biological Process	Vasculogenesis	7	2	246	0.1515
GO:0051648	IPI00696116;IPI00718152	Biological Process	Vesicle localization	6	2	246	0.5311
GO:0042060	IPI00731432;IPI00843305	Biological Process	Wound healing	6	2	246	0.8147
GO:0006370	IPI00703022	Biological Process	7-methylguanosine mRNA capping	11	1	246	0.2520
GO:0009452	IPI00703022	Biological Process	7-methylguanosine RNA capping	11	1	246	0.2520
GO:0006084	IPI00871114	Biological Process	Acetyl-CoA metabolic process	8	1	246	0.4799
GO:0030041	IPI00708767	Biological Process	Actin filament polymerization	9	1	246	0.3044
GO:0008154	IPI00708767	Biological Process	Actin polymerization or depolymerization	8	1	246	0.4799
GO:0001508	IPI00708070	Biological Process	Action potential	6	1	246	0.7666
GO:0032864	IPI00866858	Biological Process	Activation of Cdc42 GTPase activity	19	1	246	0.1957
GO:0007250	IPI00717764	Biological Process	Activation of NF-kappaB-inducing kinase activity	13	1	246	0.2520
GO:0032863	IPI00866858	Biological Process	Activation of Rac GTPase activity	19	1	246	0.2520
GO:0032856	IPI00866858	Biological Process	Activation of Ras GTPase activity	17	1	246	0.3985
GO:0032862	IPI00866858	Biological Process	Activation of Rho GTPase activity	18	1	246	0.3044

GO:0031032	IPI00697648	Biological Process	Actomyosin structure organization	7	1	246	0.6876
GO:0046463	IPI00703084	Biological Process	Acylglycerol biosynthetic process	7	1	246	0.2520
GO:0007628	IPI00698823	Biological Process	Adult walking behavior	6	1	246	0.3044
GO:0015800	IPI00708597	Biological Process	Aidic amino acid transport	10	1	246	0.2520
GO:0006419	IPI00689365	Biological Process	Alanyl-tRNA aminoacylation	13	1	246	0.2520
GO:0046165	IPI00690789	Biological Process	Alcohol biosynthetic process	7	1	246	0.7983
GO:0043604	IPI00876805	Biological Process	Amide biosynthetic process	7	1	246	0.6640
GO:0009308	IPI00839150	Biological Process	Amine metabolic process	6	1	246	0.6876
GO:0006865	IPI00708597	Biological Process	Amino acid transport	9	1	246	0.4407
GO:0046348	IPI00717764	Biological Process	Amino sugar catabolic process	7	1	246	0.3985
GO:0006040	IPI00717764	Biological Process	Amino sugar metabolic process	6	1	246	0.5164
GO:0006026	IPI00717764	Biological Process	Aminoglycan catabolic process	7	1	246	0.2520
GO:0015696	IPI00710920	Biological Process	Ammonium transport	8	1	246	0.2520
GO:0048532	IPI00876805	Biological Process	Anatomical structure arrangement	5	1	246	0.2520
GO:0060249	IPI00876805	Biological Process	Anatomical structure homeostasis	6	1	246	0.8125
GO:0009952	IPI00686595	Biological Process	Anterior/posterior pattern specification	8	1	246	0.3985
GO:0006309	IPI00709124	Biological Process	Apoptotic DNA fragmentation	10	1	246	0.3985
GO:0008637	IPI00706314	Biological Process	Apoptotic mitochondrial changes	6	1	246	0.6113
GO:0030262	IPI00709124	Biological Process	Apoptotic nuclear changes	10	1	246	0.4799
GO:0097190	IPI00698213	Biological Process	Apoptotic signaling pathway	7	1	246	0.8956
GO:0048736	IPI00687578	Biological Process	Appendage development	6	1	246	0.5504
GO:0035107	IPI00687578	Biological Process	Appendage morphogenesis	7	1	246	0.5504
GO:0009068	IPI00871114	Biological Process	Aspartate family amino acid catabolic process	9	1	246	0.3985
GO:0009066	IPI00871114	Biological Process	Aspartate family amino acid metabolic process	8	1	246	0.6640
GO:0008306	IPI00685510	Biological Process	Associative learning	7	1	246	0.3532
GO:0006200	IPI00710377	Biological Process	ATP catabolic process	11	1	246	0.7096
GO:0015986	IPI00686872	Biological Process	ATP synthesis coupled proton transport	12	1	246	0.5819

GO:0042491	IPI01003286	Biological Process	Auditory receptor cell differentiation	11	1	246	0.3044
GO:0006914	IPI00698823	Biological Process	Autophagy	6	1	246	0.6640
GO:0008088	IPI00685510	Biological Process	Axon cargo transport	8	1	246	0.6386
GO:0016198	IPI00685510	Biological Process	Axon choice point recognition	8	1	246	0.1351
GO:0007411	IPI00685510	Biological Process	Axon guidance	7	1	246	0.6876
GO:0016199	IPI00685510	Biological Process	Axon midline choice point recognition	9	1	246	0.1351
GO:0042113	IPI00705583	Biological Process	B cell activation	6	1	246	0.7096
GO:0002312	IPI00705583	Biological Process	B cell activation involved in immune response	8	1	246	0.5504
GO:0030183	IPI00705583	Biological Process	B cell differentiation	9	1	246	0.5819
GO:0006284	IPI00718678	Biological Process	Base-excision repair	10	1	246	0.3044
GO:0008206	IPI00698823	Biological Process	Bile acid metabolic process	9	1	246	0.1957
GO:0031214	IPI00707857	Biological Process	Biomineral tissue development	8	1	246	0.3985
GO:0001824	IPI00688704	Biological Process	Blastocyst development	10	1	246	0.7490
GO:0001825	IPI00688704	Biological Process	Blastocyst formation	11	1	246	0.4407
GO:0007589	IPI00709876	Biological Process	Body fluid secretion	6	1	246	0.5164
GO:0010171	IPI00707857	Biological Process	Body morphogenesis	5	1	246	0.4799
GO:0060346	IPI00707857	Biological Process	Bone trabecula formation	9	1	246	0.1957
GO:0061430	IPI00707857	Biological Process	Bone trabecula morphogenesis	6	1	246	0.1957
GO:0048854	IPI00687578	Biological Process	Brain morphogenesis	10	1	246	0.2520
GO:0009082	IPI00715066	Biological Process	Branched-chain amino acid biosynthetic process	8	1	246	0.1351
GO:0009083	IPI00715066	Biological Process	Branched-chain amino acid catabolic process	8	1	246	0.3532
GO:0009081	IPI00715066	Biological Process	Branched-chain amino acid metabolic process	7	1	246	0.3985
GO:0060444	IPI00710920	Biological Process	Branching involved in mammary gland duct morphogenesis	8	1	246	0.3532
GO:0086069	IPI00708070	Biological Process	Bundle of His cell to Purkinje myocyte communication	7	1	246	0.3044
GO:0017156	IPI00696116	Biological Process	Calcium ion-dependent exocytosis	7	1	246	0.3532
GO:0016339	IPI00709888	Biological Process	Calcium-dependent cell-cell adhesion via plasma membrane cell adhesion molecules	7	1	246	0.1351

GO:0019722	IPI00711457	Biological Process	Calcium-mediated signaling	9	1	246	0.2520
GO:0043010	IPI00710274	Biological Process	Camera-type eye development	10	1	246	0.7831
GO:0060070	IPI00709876	Biological Process	Canonical Wnt signaling pathway	9	1	246	0.4407
GO:0016051	IPI00699039	Biological Process	Carbohydrate biosynthetic process	6	1	246	0.8956
GO:1901264	IPI00691086	Biological Process	Carbohydrate derivative transport	5	1	246	0.2520
GO:0033500	IPI00702948	Biological Process	Carbohydrate homeostasis	7	1	246	0.6113
GO:0008643	IPI00714656	Biological Process	Carbohydrate transport	5	1	246	0.2520
GO:0046942	IPI00708597	Biological Process	Carboxylic acid transport	8	1	246	0.6113
GO:0003205	IPI00708070	Biological Process	Cardiac chamber development	9	1	246	0.5819
GO:0003206	IPI00708070	Biological Process	Cardiac chamber morphogenesis	10	1	246	0.5504
GO:0061337	IPI00708070	Biological Process	Cardiac conduction	6	1	246	0.3532
GO:0086001	IPI00708070	Biological Process	Cardiac muscle cell action potential	7	1	246	0.3532
GO:0086002	IPI00708070	Biological Process	Cardiac muscle cell action potential involved in contraction	10	1	246	0.3532
GO:0048738	IPI00708070	Biological Process	Cardiac muscle tissue development	9	1	246	0.7666
GO:0055008	IPI00708070	Biological Process	Cardiac muscle tissue morphogenesis	10	1	246	0.4799
GO:0003231	IPI00708070	Biological Process	Cardiac ventricle development	10	1	246	0.4799
GO:0003208	IPI00708070	Biological Process	Cardiac ventricle morphogenesis	11	1	246	0.3985
GO:0051216	IPI00707857	Biological Process	Cartilage development	8	1	246	0.6113
GO:0060351	IPI00707857	Biological Process	Cartilage development involved in endochondral bone morphogenesis	11	1	246	0.2520
GO:0042424	IPI00698059	Biological Process	Catecholamine catabolic process	7	1	246	0.1957
GO:0006584	IPI00698059	Biological Process	Catecholamine metabolic process	6	1	246	0.2520
GO:0019614	IPI00698059	Biological Process	Catechol-containing compound catabolic process	8	1	246	0.1957
GO:0009712	IPI00698059	Biological Process	Catechol-containing compound metabolic process	7	1	246	0.2520
GO:0070836	IPI00709876	Biological Process	Caveola assembly	8	1	246	0.1957
GO:0072584	IPI00709876	Biological Process	Caveolin-mediated endocytosis	9	1	246	0.1351

GO:0002263	IPI00705583	Biological Process	Cell activation involved in immune response	5	1	246	0.7490
GO:0086065	IPI00708070	Biological Process	Cell communication involved in cardiac conduction	6	1	246	0.3532
GO:0000075	IPI00712399	Biological Process	Cell cycle checkpoint	7	1	246	0.7666
GO:0022403	IPI00685510	Biological Process	Cell cycle phase	4	1	246	0.5819
GO:0051301	IPI00906031	Biological Process	Cell division	5	1	246	0.9885
GO:0016049	IPI00685510	Biological Process	Cell growth	5	1	246	0.4407
GO:0001906	IPI01028455	Biological Process	Cell killing	3	1	246	0.4799
GO:0048469	IPI00685510	Biological Process	Cell maturation	8	1	246	0.5504
GO:0030031	IPI00687578	Biological Process	Cell projection assembly	6	1	246	0.8791
GO:0008037	IPI00685510	Biological Process	Cell recognition	5	1	246	0.4407
GO:0086019	IPI00708070	Biological Process	Cell-cell signaling involved in cardiac conduction	7	1	246	0.1957
GO:0007160	IPI00731432	Biological Process	Cell-matrix adhesion	6	1	246	0.7666
GO:0097285	IPI00685510	Biological Process	Cell-type specific apoptotic process	8	1	246	0.6113
GO:0044106	IPI00839150	Biological Process	Cellular amine metabolic process	6	1	246	0.6640
GO:0030002	IPI00703813	Biological Process	Cellular anion homeostasis	8	1	246	0.3044
GO:0006576	IPI00839150	Biological Process	Cellular biogenic amine metabolic process	7	1	246	0.6640
GO:0034637	IPI00699039	Biological Process	Cellular carbohydrate biosynthetic process	6	1	246	0.6876
GO:0022411	IPI00709124	Biological Process	Cellular component disassembly	5	1	246	0.7300
GO:0006921	IPI00709124	Biological Process	Cellular component disassembly involved in execution phase of apoptosis	9	1	246	0.4799
GO:0006878	IPI00685510	Biological Process	Cellular copper ion homeostasis	11	1	246	0.2520
GO:0045123	IPI00697595	Biological Process	Cellular extravasation	5	1	246	0.1957
GO:0006073	IPI00906031	Biological Process	Cellular glucan metabolic process	7	1	246	0.6640
GO:0006879	IPI00710664	Biological Process	Cellular iron ion homeostasis	11	1	246	0.5819
GO:0042180	IPI00695952	Biological Process	Cellular ketone metabolic process	5	1	246	0.4799
GO:0030026	IPI00709888	Biological Process	Cellular manganese ion homeostasis	11	1	246	0.1351
GO:0030643	IPI00703813	Biological Process	Cellular phosphate ion homeostasis	10	1	246	0.2520
GO:0033059	IPI00718152	Biological Process	Cellular pigmentation	5	1	246	0.2520

GO:0033692	IPI00699039	Biological Process	Cellular polysaccharide biosynthetic process	7	1	246	0.5819
GO:0048610	IPI00690309	Biological Process	Cellular process involved in reproduction	NA	1	246	0.9567
GO:0022412	IPI00690309	Biological Process	Cellular process involved in reproduction in multicellular organism	5	1	246	0.7983
GO:0071214	IPI00707857	Biological Process	Cellular response to abiotic stimulus	5	1	246	0.8380
GO:0071216	IPI00690581	Biological Process	Cellular response to biotic stimulus	5	1	246	0.7300
GO:0071345	IPI00717764	Biological Process	Cellular response to cytokine stimulus	7	1	246	0.9029
GO:0036294	IPI00690309	Biological Process	Cellular response to decreased oxygen levels	7	1	246	0.3985
GO:0070301	IPI00716157	Biological Process	Cellular response to hydrogen peroxide	8	1	246	0.5504
GO:0071455	IPI00709876	Biological Process	Cellular response to hyperoxia	6	1	246	0.1957
GO:0071456	IPI00690309	Biological Process	Cellular response to hypoxia	6	1	246	0.3985
GO:0036295	IPI00709876	Biological Process	Cellular response to increased oxygen levels	7	1	246	0.1957
GO:0071222	IPI00690581	Biological Process	Cellular response to lipopolysaccharide	8	1	246	0.6876
GO:0071402	IPI00698823	Biological Process	Cellular response to lipoprotein particle stimulus	7	1	246	0.1351
GO:0071404	IPI00698823	Biological Process	Cellular response to low-density lipoprotein particle stimulus	8	1	246	0.1351
GO:0071260	IPI00707857	Biological Process	Cellular response to mechanical stimulus	6	1	246	0.5504
GO:0071219	IPI00690581	Biological Process	Cellular response to molecule of bacterial origin	6	1	246	0.7096
GO:0034614	IPI00716157	Biological Process	Cellular response to reactive oxygen species	7	1	246	0.7096
GO:0071383	IPI00698823	Biological Process	Cellular response to steroid hormone stimulus	8	1	246	0.3532
GO:0035967	IPI00690581	Biological Process	Cellular response to topologically incorrect protein	6	1	246	0.3985
GO:0071356	IPI00717764	Biological Process	Cellular response to tumor necrosis factor	8	1	246	0.5504
GO:0034620	IPI00690581	Biological Process	Cellular response to unfolded protein	7	1	246	0.3985
GO:0071466	IPI00707815	Biological Process	Cellular response to xenobiotic stimulus	6	1	246	0.2520

GO:0072502	IPI00703813	Biological Process	Cellular trivalent inorganic anion homeostasis	9	1	246	0.2520
GO:0021695	IPI00687578	Biological Process	Cerebellar cortex development	13	1	246	0.5164
GO:0021696	IPI00687578	Biological Process	Cerebellar cortex morphogenesis	13	1	246	0.3044
GO:0021549	IPI00687578	Biological Process	Cerebellum development	12	1	246	0.5504
GO:0021587	IPI00687578	Biological Process	Cerebellum morphogenesis	12	1	246	0.3532
GO:0006032	IPI00717764	Biological Process	Chitin catabolic process	8	1	246	0.1957
GO:0006030	IPI00717764	Biological Process	Chitin metabolic process	7	1	246	0.1957
GO:0006821	IPI00710920	Biological Process	Chloride transport	8	1	246	0.1957
GO:0006695	IPI00690789	Biological Process	Cholesterol biosynthetic process	9	1	246	0.5164
GO:0033344	IPI00698823	Biological Process	Cholesterol efflux	8	1	246	0.5164
GO:0030301	IPI00698823	Biological Process	Cholesterol transport	7	1	246	0.5504
GO:0051304	IPI00693579	Biological Process	Chromosome separation	7	1	246	0.1351
GO:0042384	IPI00687578	Biological Process	Cilium assembly	6	1	246	0.3044
GO:0060271	IPI00687578	Biological Process	Cilium morphogenesis	7	1	246	0.6386
GO:0044782	IPI00687578	Biological Process	Cilium organization	6	1	246	0.3044
GO:0048268	IPI00866975	Biological Process	Clathrin coat assembly	8	1	246	0.3532
GO:0015937	IPI00704332	Biological Process	Coenzyme A biosynthetic process	11	1	246	0.3044
GO:0015938	IPI00694430	Biological Process	Coenzyme A catabolic process	11	1	246	0.1351
GO:0009109	IPI00694430	Biological Process	Coenzyme catabolic process	7	1	246	0.1957
GO:0051187	IPI00694430	Biological Process	Cofactor catabolic process	6	1	246	0.3532
GO:0050890	IPI00685510	Biological Process	Cognition	7	1	246	0.6386
GO:0048668	IPI00685510	Biological Process	Collateral sprouting	7	1	246	0.1351
GO:0048669	IPI00685510	Biological Process	Collateral sprouting in absence of injury	8	1	246	0.1351
GO:0002065	IPI00696748	Biological Process	Columnar/cuboidal epithelial cell differentiation	8	1	246	0.2520
GO:0061448	IPI00707857	Biological Process	Connective tissue development	6	1	246	0.6876
GO:0055070	IPI00685510	Biological Process	Copper ion homeostasis	11	1	246	0.2520
GO:0006613	IPI00703954	Biological Process	Cotranslational protein targeting to membrane	8	1	246	0.4799
GO:0021545	IPI00876805	Biological Process	Cranial nerve development	9	1	246	0.1957
GO:0021602	IPI00876805	Biological Process	Cranial nerve morphogenesis	10	1	246	0.1957
GO:0021604	IPI00876805	Biological Process	Cranial nerve structural organization	11	1	246	0.1351
GO:0071034	IPI01017508	Biological Process	CUT catabolic process	10	1	246	0.1957
GO:0071043	IPI01017508	Biological Process	CUT metabolic process	10	1	246	0.1957

GO:0017004	IPI00688474	Biological Process	Cytochrome complex assembly	9	1	246	0.1957
GO:0001897	IPI01028455	Biological Process	Cytolysis by symbiont of host cells	7	1	246	0.1351
GO:0051715	IPI01028455	Biological Process	Cytolysis in other organism	5	1	246	0.1351
GO:0051801	IPI01028455	Biological Process	Cytolysis in other organism involved in symbiotic interaction	6	1	246	0.1351
GO:0033962	IPI00704902	Biological Process	Cytoplasmic mRNA processing body assembly	6	1	246	0.3532
GO:0030705	IPI00685510	Biological Process	Cytoskeleton-dependent intracellular transport	6	1	246	0.6876
GO:0051480	IPI00709876	Biological Process	Cytosolic calcium ion homeostasis	11	1	246	0.5504
GO:0000290	IPI00704902	Biological Process	Deadenylation-dependent decapping of nuclear-transcribed mRNA	12	1	246	0.1957
GO:0051607	IPI00703084	Biological Process	Defense response to virus	5	1	246	0.5819
GO:0070837	IPI00714656	Biological Process	Dehydroascorbic acid transport	8	1	246	0.1351
GO:0048813	IPI00866975	Biological Process	Dendrite morphogenesis	8	1	246	0.3044
GO:0016311	IPI00906031	Biological Process	Dephosphorylation	7	1	246	0.9098
GO:0002159	IPI00708070	Biological Process	Desmosome assembly	8	1	246	0.2520
GO:0002934	IPI00708070	Biological Process	Desmosome organization	7	1	246	0.3044
GO:0009582	IPI00710920	Biological Process	Detection of abiotic stimulus	5	1	246	0.4407
GO:0009581	IPI00710920	Biological Process	Detection of external stimulus	5	1	246	0.5164
GO:0050982	IPI00710920	Biological Process	Detection of mechanical stimulus	6	1	246	0.3044
GO:0050974	IPI00710920	Biological Process	Detection of mechanical stimulus involved in sensory perception	9	1	246	0.1957
GO:0050910	IPI00710920	Biological Process	Detection of mechanical stimulus involved in sensory perception of sound	10	1	246	0.1957
GO:0051606	IPI00710920	Biological Process	Detection of stimulus	4	1	246	0.5819
GO:0050906	IPI00710920	Biological Process	Detection of stimulus involved in sensory perception	8	1	246	0.2520
GO:0048588	IPI00685510	Biological Process	Developmental cell growth	6	1	246	0.3044
GO:0021700	IPI00685510	Biological Process	Developmental maturation	5	1	246	0.7300
GO:0006835	IPI00708597	Biological Process	Dicarboxylic acid transport	9	1	246	0.3532

GO:0055123	IPI00731432	Biological Process	Digestive system development	7	1	246	0.3985
GO:0048565	IPI00731432	Biological Process	Digestive tract development	7	1	246	0.3985
GO:0036072	IPI00707857	Biological Process	Direct ossification	6	1	246	0.1957
GO:0044004	IPI01028455	Biological Process	Disruption by symbiont of host cell	7	1	246	0.1351
GO:0044364	IPI01028455	Biological Process	Disruption of cells of other organism	5	1	246	0.3044
GO:0051818	IPI01028455	Biological Process	Disruption of cells of other organism involved in symbiotic interaction	6	1	246	0.1957
GO:0006308	IPI00709124	Biological Process	DNA catabolic process	8	1	246	0.4799
GO:0000737	IPI00709124	Biological Process	DNA catabolic process, endonucleolytic	9	1	246	0.3985
GO:0000077	IPI00712399	Biological Process	DNA damage checkpoint	9	1	246	0.5164
GO:0031570	IPI00712399	Biological Process	DNA integrity checkpoint	8	1	246	0.5504
GO:0006310	IPI00712399	Biological Process	DNA recombination	9	1	246	0.7300
GO:0006260	IPI00701747	Biological Process	DNA replication	9	1	246	0.7983
GO:0042420	IPI00698059	Biological Process	Dopamine catabolic process	7	1	246	0.1957
GO:0042417	IPI00698059	Biological Process	Dopamine metabolic process	6	1	246	0.1957
GO:0007549	IPI00698339	Biological Process	Dosage compensation	9	1	246	0.2520
GO:0006302	IPI00712399	Biological Process	Double-strand break repair	10	1	246	0.6640
GO:0000724	IPI00712399	Biological Process	Double-strand break repair via homologous recombination	11	1	246	0.3044
GO:0043583	IPI01003286	Biological Process	Ear development	9	1	246	0.6640
GO:0007398	IPI00710274	Biological Process	Ectoderm development	6	1	246	0.2520
GO:0001838	IPI00699039	Biological Process	Embryonic epithelial tube formation	9	1	246	0.7983
GO:0048706	IPI00707857	Biological Process	Embryonic skeletal system development	9	1	246	0.5504
GO:0010256	IPI00713474	Biological Process	Endomembrane system organization	5	1	246	0.2520
GO:0032469	IPI00685510	Biological Process	Endoplasmic reticulum calcium ion homeostasis	11	1	246	0.3985
GO:0030968	IPI00690581	Biological Process	Endoplasmic reticulum unfolded protein response	8	1	246	0.3985
GO:0016197	IPI00866975	Biological Process	Endosomal transport	6	1	246	0.9276
GO:0001885	IPI00876805	Biological Process	Endothelial cell development	9	1	246	0.4407
GO:0045446	IPI00876805	Biological Process	Endothelial cell differentiation	8	1	246	0.4407
GO:0003158	IPI00876805	Biological Process	Endothelium development	6	1	246	0.4799

GO:0015985	IPI00686872	Biological Process	Energy coupled proton transport, down electrochemical gradient	10	1	246	0.5819
GO:0006112	IPI00906031	Biological Process	Energy reserve metabolic process	7	1	246	0.6876
GO:0009913	IPI01003286	Biological Process	Epidermal cell differentiation	8	1	246	0.7666
GO:0002064	IPI00876805	Biological Process	Epithelial cell development	8	1	246	0.8876
GO:0072175	IPI00699039	Biological Process	Epithelial tube formation	9	1	246	0.7983
GO:0006983	IPI00690581	Biological Process	ER overload response	8	1	246	0.1957
GO:0030433	IPI00690581	Biological Process	ER-associated ubiquitin-dependent protein catabolic process	11	1	246	0.6113
GO:0006984	IPI00690581	Biological Process	ER-nucleus signaling pathway	7	1	246	0.5504
GO:0060856	IPI00876805	Biological Process	Establishment of blood-brain barrier	8	1	246	0.1957
GO:0008065	IPI00876805	Biological Process	Establishment of blood-nerve barrier	10	1	246	0.1957
GO:0060857	IPI00876805	Biological Process	Establishment of glial blood-brain barrier	9	1	246	0.1351
GO:0032401	IPI00718152	Biological Process	Establishment of melanosome localization	8	1	246	0.1351
GO:0051905	IPI00718152	Biological Process	Establishment of pigment granule localization	7	1	246	0.1351
GO:0072655	IPI00707815	Biological Process	Establishment of protein localization to mitochondrion	9	1	246	0.6876
GO:0090002	IPI00696116	Biological Process	Establishment of protein localization to plasma membrane	7	1	246	0.6386
GO:0097480	IPI00696116	Biological Process	Establishment of synaptic vesicle localization	7	1	246	0.6640
GO:0042439	IPI00839150	Biological Process	Ethanolamine-containing compound metabolic process	8	1	246	0.6386
GO:0097194	IPI00709124	Biological Process	Execution phase of apoptosis	8	1	246	0.5164
GO:0043206	IPI00731432	Biological Process	Extracellular fibril organization	7	1	246	0.1957
GO:0048592	IPI00826022	Biological Process	Eye morphogenesis	10	1	246	0.6640
GO:0060324	IPI00707857	Biological Process	Face development	6	1	246	0.3532
GO:0060325	IPI00707857	Biological Process	Face morphogenesis	7	1	246	0.3532
GO:0055089	IPI00703084	Biological Process	Fatty acid homeostasis	9	1	246	0.1957
GO:0007631	IPI00685510	Biological Process	Feeding behavior	4	1	246	0.3532
GO:0097435	IPI00731432	Biological Process	Fibril organization	5	1	246	0.1957
GO:0001704	IPI00710274	Biological Process	Formation of primary germ layer	9	1	246	0.5504
GO:0051319	IPI00685510	Biological Process	G2 phase	5	1	246	0.1351

GO:0033483	IPI00709876	Biological Process	Gas homeostasis	7	1	246	0.2520
GO:0007369	IPI00710274	Biological Process	Gastrulation	8	1	246	0.7096
GO:0003001	IPI00696116	Biological Process	Generation of a signal involved in cell-cell signaling	NA	1	246	0.5504
GO:0007281	IPI00690309	Biological Process	Germ cell development	6	1	246	0.7666
GO:0021782	IPI00876805	Biological Process	Glial cell development	8	1	246	0.6640
GO:0010001	IPI00876805	Biological Process	Glial cell differentiation	7	1	246	0.7490
GO:0008347	IPI00713597	Biological Process	Glial cell migration	8	1	246	0.1957
GO:0014009	IPI00710274	Biological Process	Glial cell proliferation	9	1	246	0.1957
GO:0044042	IPI00906031	Biological Process	Glucan metabolic process	7	1	246	0.6640
GO:1901072	IPI00717764	Biological Process	Glucosamine-containing compound catabolic process	8	1	246	0.1957
GO:1901071	IPI00717764	Biological Process	Glucosamine-containing compound metabolic process	7	1	246	0.3532
GO:0042593	IPI00702948	Biological Process	Glucose homeostasis	8	1	246	0.6113
GO:0046323	IPI00714656	Biological Process	Glucose import	9	1	246	0.1351
GO:0015758	IPI00714656	Biological Process	Glucose transport	8	1	246	0.1351
GO:0007215	IPI00685510	Biological Process	Glutamate receptor signaling pathway	8	1	246	0.1957
GO:0006542	IPI00760457	Biological Process	Glutamine biosynthetic process	10	1	246	0.1351
GO:0009084	IPI00760457	Biological Process	Glutamine family amino acid biosynthetic process	9	1	246	0.4799
GO:0009064	IPI00760457	Biological Process	Glutamine family amino acid metabolic process	8	1	246	0.7831
GO:0006541	IPI00760457	Biological Process	Glutamine metabolic process	9	1	246	0.3985
GO:0006425	IPI00708246	Biological Process	Glutaminyl-tRNA aminoacylation	13	1	246	0.1351
GO:0005977	IPI00906031	Biological Process	Glycogen metabolic process	8	1	246	0.6640
GO:0070085	IPI00698823	Biological Process	Glycosylation	7	1	246	0.9029
GO:0032468	IPI00709888	Biological Process	Golgi calcium ion homeostasis	11	1	246	0.1351
GO:0032472	IPI00709888	Biological Process	Golgi calcium ion transport	10	1	246	0.1351
GO:0007030	IPI00708162	Biological Process	Golgi organization	5	1	246	0.7096
GO:0043001	IPI00696116	Biological Process	Golgi to plasma membrane protein transport	9	1	246	0.5164
GO:0071621	IPI00697595	Biological Process	Granulocyte chemotaxis	6	1	246	0.3532
GO:0035315	IPI01003286	Biological Process	Hair cell differentiation	10	1	246	0.3044
GO:0060322	IPI00707857	Biological Process	Head development	5	1	246	0.3532
GO:0060323	IPI00707857	Biological Process	Head morphogenesis	6	1	246	0.3532
GO:0003007	IPI00708070	Biological Process	Heart morphogenesis	9	1	246	0.7666

GO:0019836	IPI01028455	Biological Process	Hemolysis by symbiont of host erythrocytes	8	1	246	0.1351
GO:0044179	IPI01028455	Biological Process	Hemolysis in other organism	6	1	246	0.1351
GO:0052331	IPI01028455	Biological Process	Hemolysis in other organism involved in symbiotic interaction	7	1	246	0.1351
GO:0015012	IPI00699039	Biological Process	Heparan sulfate proteoglycan biosynthetic process	9	1	246	0.1351
GO:0015014	IPI00699039	Biological Process	Heparan sulfate proteoglycan biosynthetic process, polysaccharide chain biosynthetic process	8	1	246	0.1351
GO:0030201	IPI00699039	Biological Process	Heparan sulfate proteoglycan metabolic process	7	1	246	0.1957
GO:0030202	IPI00699039	Biological Process	Heparin metabolic process	9	1	246	0.1351
GO:0008645	IPI00714656	Biological Process	Hexose transport	7	1	246	0.1351
GO:0030902	IPI00687578	Biological Process	Hindbrain development	10	1	246	0.6113
GO:0021575	IPI00687578	Biological Process	Hindbrain morphogenesis	11	1	246	0.3532
GO:0034969	IPI00707776	Biological Process	Histone arginine methylation	8	1	246	0.1957
GO:0071110	IPI00727591	Biological Process	Histone biotinylation	9	1	246	0.1351
GO:0034970	IPI00707776	Biological Process	Histone H3-R2 methylation	9	1	246	0.1351
GO:0016571	IPI00707776	Biological Process	Histone methylation	7	1	246	0.3532
GO:0006398	IPI00688446	Biological Process	Histone mRNA 3'-end processing	12	1	246	0.1351
GO:0008334	IPI00688446	Biological Process	Histone mRNA metabolic process	10	1	246	0.1957
GO:0016574	IPI00718678	Biological Process	Histone ubiquitination	9	1	246	0.5164
GO:0007156	IPI00713979	Biological Process	Homophilic cell adhesion via plasma membrane adhesion molecules	7	1	246	0.5504
GO:0030212	IPI00717930	Biological Process	Hyaluronan metabolic process	9	1	246	0.3532
GO:0042744	IPI00716157	Biological Process	Hydrogen peroxide catabolic process	6	1	246	0.3985
GO:0042743	IPI00716157	Biological Process	Hydrogen peroxide metabolic process	6	1	246	0.4407
GO:0006955	IPI00705583	Biological Process	Immune response	4	1	246	0.9983
GO:0000188	IPI00709876	Biological Process	Inactivation of MAPK activity	14	1	246	0.1957
GO:0001826	IPI00688704	Biological Process	Inner cell mass cell differentiation	7	1	246	0.1351
GO:0048839	IPI01003286	Biological Process	Inner ear development	10	1	246	0.6386

GO:0060113	IPI01003286	Biological Process	Inner ear receptor cell differentiation	11	1	246	0.3532
GO:0015698	IPI00710920	Biological Process	Inorganic anion transport	7	1	246	0.3532
GO:0048017	IPI00715059	Biological Process	Inositol lipid-mediated signaling	8	1	246	0.5164
GO:0008286	IPI00715059	Biological Process	Insulin receptor signaling pathway	10	1	246	0.3985
GO:0048009	IPI00715059	Biological Process	Insulin-like growth factor receptor signaling pathway	10	1	246	0.3044
GO:0032623	IPI00703084	Biological Process	Interleukin-2 production	6	1	246	0.1957
GO:0070970	IPI00703084	Biological Process	Interleukin-2 secretion	8	1	246	0.1351
GO:0032637	IPI00717764	Biological Process	Interleukin-8 production	6	1	246	0.1351
GO:0072606	IPI00717764	Biological Process	Interleukin-8 secretion	8	1	246	0.1351
GO:0045110	IPI00708070	Biological Process	Intermediate filament bundle assembly	8	1	246	0.1957
GO:0045104	IPI00708070	Biological Process	Intermediate filament cytoskeleton organization	6	1	246	0.3985
GO:0045109	IPI00708070	Biological Process	Intermediate filament organization	7	1	246	0.3985
GO:0045103	IPI00708070	Biological Process	Intermediate filament-based process	5	1	246	0.3985
GO:0051325	IPI00685510	Biological Process	Interphase	5	1	246	0.4799
GO:0030520	IPI00707776	Biological Process	Intracellular estrogen receptor signaling pathway	9	1	246	0.1957
GO:0030522	IPI00707776	Biological Process	Intracellular receptor signaling pathway	7	1	246	0.6876
GO:0030518	IPI00707776	Biological Process	Intracellular steroid hormone receptor signaling pathway	8	1	246	0.5164
GO:0006891	IPI01003286	Biological Process	Intra-Golgi vesicle-mediated transport	7	1	246	0.6876
GO:0001957	IPI00707857	Biological Process	Intramembranous ossification	7	1	246	0.1957
GO:0097193	IPI00698213	Biological Process	Intrinsic apoptotic signaling pathway	8	1	246	0.7831
GO:0072332	IPI00698213	Biological Process	Intrinsic apoptotic signaling pathway by p53 class mediator	9	1	246	0.3985
GO:0035235	IPI00685510	Biological Process	Ionotropic glutamate receptor signaling pathway	9	1	246	0.1351
GO:0055072	IPI00710664	Biological Process	Iron ion homeostasis	11	1	246	0.7490
GO:0006826	IPI00710664	Biological Process	Iron ion transport	9	1	246	0.4799

GO:0009097	IPI00715066	Biological Process	Isoleucine biosynthetic process	9	1	246	0.1351
GO:0006550	IPI00715066	Biological Process	Isoleucine catabolic process	9	1	246	0.1351
GO:0006549	IPI00715066	Biological Process	Isoleucine metabolic process	8	1	246	0.1351
GO:0008299	IPI00690789	Biological Process	Isoprenoid biosynthetic process	6	1	246	0.3985
GO:0006720	IPI00690789	Biological Process	Isoprenoid metabolic process	6	1	246	0.6876
GO:0042181	IPI00695952	Biological Process	Ketone biosynthetic process	6	1	246	0.3985
GO:0001907	IPI01028455	Biological Process	Killing by symbiont of host cells	6	1	246	0.1351
GO:0051883	IPI01028455	Biological Process	Killing of cells in other organism involved in symbiotic interaction	5	1	246	0.1957
GO:0031640	IPI01028455	Biological Process	Killing of cells of other organism	4	1	246	0.3044
GO:0007595	IPI00709876	Biological Process	Lactation	10	1	246	0.4407
GO:0015807	IPI00708597	Biological Process	L-amino acid transport	10	1	246	0.2520
GO:0007612	IPI00685510	Biological Process	Learning	6	1	246	0.4407
GO:0007611	IPI00685510	Biological Process	Learning or memory	5	1	246	0.6386
GO:0002088	IPI00710274	Biological Process	Lens development in camera-type eye	11	1	246	0.3985
GO:0070306	IPI00710274	Biological Process	Lens fiber cell differentiation	8	1	246	0.2520
GO:0009098	IPI00715066	Biological Process	Leucine biosynthetic process	9	1	246	0.1351
GO:0006551	IPI00715066	Biological Process	Leucine metabolic process	8	1	246	0.1957
GO:0002366	IPI00705583	Biological Process	Leukocyte activation involved in immune response	6	1	246	0.7490
GO:0030595	IPI00697595	Biological Process	Leukocyte chemotaxis	5	1	246	0.5164
GO:0002521	IPI00705583	Biological Process	Leukocyte differentiation	7	1	246	0.9029
GO:0050900	IPI00697595	Biological Process	Leukocyte migration	4	1	246	0.6876
GO:0019370	IPI00705565	Biological Process	Leukotriene biosynthetic process	9	1	246	0.3985
GO:0015813	IPI00708597	Biological Process	L-glutamate transport	10	1	246	0.2520
GO:0060173	IPI00687578	Biological Process	Limb development	7	1	246	0.5504
GO:0035108	IPI00687578	Biological Process	Limb morphogenesis	8	1	246	0.5504
GO:0016042	IPI00715059	Biological Process	Lipid catabolic process	6	1	246	0.9375
GO:0019915	IPI00709876	Biological Process	Lipid storage	6	1	246	0.3985
GO:0042157	IPI00694597	Biological Process	Lipoprotein metabolic process	6	1	246	0.6876
GO:0006691	IPI00705565	Biological Process	Lleukotriene metabolic process	9	1	246	0.4407

GO:0019477	IPI00871114	Biological Process	L-lysine catabolic process	11	1	246	0.1957
GO:0019474	IPI00871114	Biological Process	L-lysine catabolic process to acetyl-CoA	12	1	246	0.1957
GO:0033512	IPI00871114	Biological Process	L-lysine catabolic process to acetyl-CoA via saccharopine	13	1	246	0.1957
GO:0046440	IPI00871114	Biological Process	L-lysine metabolic process	10	1	246	0.1957
GO:0002285	IPI00705583	Biological Process	Lymphocyte activation involved in immune response	7	1	246	0.6113
GO:0030098	IPI00705583	Biological Process	Lymphocyte differentiation	8	1	246	0.8258
GO:0006554	IPI00871114	Biological Process	Lysine catabolic process	10	1	246	0.1957
GO:0006553	IPI00871114	Biological Process	Lysine metabolic process	9	1	246	0.1957
GO:0007041	IPI00698823	Biological Process	Lysosomal transport	7	1	246	0.6640
GO:0043413	IPI00698823	Biological Process	Macromolecule glycosylation	8	1	246	0.8791
GO:0043414	IPI00707776	Biological Process	Macromolecule methylation	5	1	246	0.6113
GO:0051235	IPI01028455	Biological Process	Maintenance of location	4	1	246	0.8601
GO:0051651	IPI01028455	Biological Process	Maintenance of location in cell	5	1	246	0.8258
GO:0051659	IPI01028455	Biological Process	Maintenance of mitochondrion location	7	1	246	0.1351
GO:0048496	IPI00708070	Biological Process	Maintenance of organ identity	9	1	246	0.1351
GO:0051657	IPI01028455	Biological Process	Maintenance of organelle location	6	1	246	0.1351
GO:0043490	IPI00730219	Biological Process	Malate-aspartate shuttle	9	1	246	0.1957
GO:0048232	IPI00712399	Biological Process	Male gamete generation	6	1	246	0.9276
GO:2001294	IPI00694430	Biological Process	Malonyl-CoA catabolic process	12	1	246	0.1351
GO:2001293	IPI00694430	Biological Process	Malonyl-CoA metabolic process	13	1	246	0.1957
GO:0060763	IPI00710920	Biological Process	Mammary duct terminal end bud growth	10	1	246	0.1351
GO:0060603	IPI00710920	Biological Process	Mammary gland duct morphogenesis	9	1	246	0.4407
GO:0061180	IPI00710920	Biological Process	Mammary gland epithelium development	10	1	246	0.5819
GO:0060056	IPI00709876	Biological Process	Mammary gland involution	11	1	246	0.1351
GO:0055071	IPI00709888	Biological Process	Manganese ion homeostasis	11	1	246	0.1351
GO:0000165	IPI00709876	Biological Process	MAPK cascade	9	1	246	0.7300
GO:0007618	IPI00685510	Biological Process	Mating	5	1	246	0.1351
GO:0007617	IPI00685510	Biological Process	Mating behavior	6	1	246	0.1351
GO:0002335	IPI00705583	Biological Process	Mature B cell differentiation	10	1	246	0.3532

GO:0002313	IPI00705583	Biological Process	Mature B cell differentiation involved in immune response	9	1	246	0.3532
GO:0042490	IPI01003286	Biological Process	Mechanoreceptor differentiation	10	1	246	0.3532
GO:0032400	IPI00718152	Biological Process	Melanosome localization	7	1	246	0.1351
GO:0032402	IPI00718152	Biological Process	Melanosome transport	8	1	246	0.1351
GO:0006900	IPI00866975	Biological Process	Membrane budding	6	1	246	0.5504
GO:0051899	IPI00709876	Biological Process	Membrane depolarization	6	1	246	0.2520
GO:0033619	IPI00715640	Biological Process	Membrane protein proteolysis	7	1	246	0.4407
GO:0001765	IPI00709876	Biological Process	Membrane raft assembly	6	1	246	0.1957
GO:0031579	IPI00709876	Biological Process	Membrane raft organization	5	1	246	0.2520
GO:0007498	IPI00710274	Biological Process	Mesoderm development	6	1	246	0.4407
GO:0001707	IPI00710274	Biological Process	Mesoderm formation	10	1	246	0.3985
GO:0048332	IPI00710274	Biological Process	Mesoderm morphogenesis	6	1	246	0.3985
GO:0022037	IPI00687578	Biological Process	Metencephalon development	11	1	246	0.5504
GO:0032259	IPI00707776	Biological Process	Methylation	4	1	246	0.7096
GO:0001578	IPI00689379	Biological Process	Microtubule bundle formation	7	1	246	0.4407
GO:0007018	IPI00685510	Biological Process	Microtubule-based movement	6	1	246	0.8601
GO:0010970	IPI00685510	Biological Process	Microtubule-based transport	7	1	246	0.6876
GO:0070143	IPI00689365	Biological Process	Mitochondrial alanyl-tRNA aminoacylation	11	1	246	0.1351
GO:0051560	IPI00711457	Biological Process	Mitochondrial calcium ion homeostasis	11	1	246	0.3044
GO:0006851	IPI00711457	Biological Process	Mitochondrial calcium ion transport	10	1	246	0.1351
GO:0034551	IPI00688474	Biological Process	Mitochondrial respiratory chain complex III assembly	7	1	246	0.1957
GO:0097033	IPI00688474	Biological Process	Mitochondrial respiratory chain complex III biogenesis	6	1	246	0.1957
GO:0000959	IPI00689365	Biological Process	Mitochondrial RNA metabolic process	9	1	246	0.3044
GO:0032543	IPI00689365	Biological Process	Mitochondrial translation	8	1	246	0.2520
GO:0051646	IPI01028455	Biological Process	Mitochondrion localization	6	1	246	0.3985
GO:0000085	IPI00685510	Biological Process	Mitotic G2 phase	8	1	246	0.1351
GO:0051329	IPI00685510	Biological Process	Mitotic interphase	7	1	246	0.4407
GO:0007067	IPI00693579	Biological Process	Mitotic nuclear division	7	1	246	0.8380

GO:0000070	IPI00693579	Biological Process	Mitotic sister chromatid segregation	8	1	246	0.3985
GO:0051306	IPI00693579	Biological Process	Mitotic sister chromatid separation	9	1	246	0.1351
GO:0019048	IPI00713313	Biological Process	Modulation by virus of host morphology or physiology	6	1	246	0.4799
GO:0015749	IPI00714656	Biological Process	Monosaccharide transport	6	1	246	0.1351
GO:0016331	IPI00699039	Biological Process	Morphogenesis of embryonic epithelium	8	1	246	0.8380
GO:0006402	IPI00704902	Biological Process	mRNA catabolic process	9	1	246	0.6876
GO:0006406	IPI00700228	Biological Process	mRNA export from nucleus	7	1	246	0.5819
GO:0006378	IPI00685510	Biological Process	mRNA polyadenylation	12	1	246	0.4407
GO:0051705	IPI00685510	Biological Process	Multi-organism behavior	4	1	246	0.3044
GO:0044764	IPI00713313	Biological Process	Multi-organism cellular process	4	1	246	0.7096
GO:0044703	IPI00685510	Biological Process	Multi-organism reproductive process	4	1	246	0.5819
GO:0046716	IPI00876805	Biological Process	Muscle cell cellular homeostasis	6	1	246	0.3532
GO:0048644	IPI00708070	Biological Process	Muscle organ morphogenesis	9	1	246	0.5164
GO:0060415	IPI00708070	Biological Process	Muscle tissue morphogenesis	10	1	246	0.5164
GO:0030239	IPI00697648	Biological Process	Myofibril assembly	8	1	246	0.5164
GO:0014904	IPI00876805	Biological Process	Myotube cell development	10	1	246	0.1351
GO:0014902	IPI00876805	Biological Process	Myotube differentiation	9	1	246	0.2520
GO:0034661	IPI01017508	Biological Process	ncRNA catabolic process	9	1	246	0.2520
GO:0034470	IPI00702948	Biological Process	ncRNA processing	10	1	246	0.9327
GO:0070236	IPI00703084	Biological Process	Negative regulation of activation-induced cell death of T cells	9	1	246	0.1351
GO:0002674	IPI00690581	Biological Process	Negative regulation of acute inflammatory response	9	1	246	0.1351
GO:0002865	IPI00690581	Biological Process	Negative regulation of acute inflammatory response to antigenic stimulus	8	1	246	0.1351
GO:0016525	IPI00712524	Biological Process	Negative regulation of angiogenesis	11	1	246	0.4799
GO:2000811	IPI00709876	Biological Process	Negative regulation of anoikis	10	1	246	0.2520

GO:0030514	IPI00709876	Biological Process	Negative regulation of BMP signaling pathway	15	1	246	0.3044
GO:0045792	IPI00690309	Biological Process	Negative regulation of cell size	7	1	246	0.1351
GO:0022408	IPI00710274	Biological Process	Negative regulation of cell-cell adhesion	6	1	246	0.3532
GO:0010812	IPI00707857	Biological Process	Negative regulation of cell-substrate adhesion	6	1	246	0.4799
GO:0001818	IPI00690581	Biological Process	Negative regulation of cytokine production	7	1	246	0.6876
GO:0001960	IPI00709876	Biological Process	Negative regulation of cytokine-mediated signaling pathway	10	1	246	0.4799
GO:0031348	IPI00690581	Biological Process	Negative regulation of defense response	7	1	246	0.6386
GO:0051053	IPI00710274	Biological Process	Negative regulation of DNA metabolic process	10	1	246	0.6386
GO:0008156	IPI00710274	Biological Process	Negative regulation of DNA replication	11	1	246	0.4407
GO:0010951	IPI00867230	Biological Process	Negative regulation of endopeptidase activity	11	1	246	0.7666
GO:0001937	IPI00709876	Biological Process	Negative regulation of endothelial cell proliferation	7	1	246	0.4799
GO:0030857	IPI00709876	Biological Process	Negative regulation of epithelial cell differentiation	9	1	246	0.1957
GO:0010633	IPI00845178	Biological Process	Negative regulation of epithelial cell migration	11	1	246	0.4407
GO:1901194	IPI00712384	Biological Process	Negative regulation of formation of translation preinitiation complex	11	1	246	0.1351
GO:0031947	IPI00705159	Biological Process	Negative regulation of glucocorticoid biosynthetic process	8	1	246	0.1351
GO:0031944	IPI00705159	Biological Process	Negative regulation of glucocorticoid metabolic process	7	1	246	0.1351
GO:0045822	IPI00705159	Biological Process	Negative regulation of heart contraction	10	1	246	0.1957

GO:0032353	IPI00705159	Biological Process	Negative regulation of hormone biosynthetic process	6	1	246	0.1351
GO:0032351	IPI00705159	Biological Process	Negative regulation of hormone metabolic process	6	1	246	0.1351
GO:0046888	IPI00715059	Biological Process	Negative regulation of hormone secretion	10	1	246	0.2520
GO:0050728	IPI00690581	Biological Process	Negative regulation of inflammatory response	8	1	246	0.4799
GO:0002862	IPI00690581	Biological Process	Negative regulation of inflammatory response to antigenic stimulus	7	1	246	0.2520
GO:0046676	IPI00715059	Biological Process	Negative regulation of insulin secretion	11	1	246	0.2520
GO:0032715	IPI00690581	Biological Process	Negative regulation of interleukin-6 production	8	1	246	0.2520
GO:0090317	IPI00690309	Biological Process	Negative regulation of intracellular protein transport	8	1	246	0.5819
GO:0032387	IPI00690309	Biological Process	Negative regulation of intracellular transport	7	1	246	0.6640
GO:2000107	IPI00703084	Biological Process	Negative regulation of leukocyte apoptotic process	11	1	246	0.2520
GO:0051055	IPI00705159	Biological Process	Negative regulation of lipid biosynthetic process	8	1	246	0.3532
GO:0045833	IPI00705159	Biological Process	Negative regulation of lipid metabolic process	7	1	246	0.5164
GO:0010888	IPI00705441	Biological Process	Negative regulation of lipid storage	8	1	246	0.1957
GO:0070229	IPI00703084	Biological Process	Negative regulation of lymphocyte apoptotic process	12	1	246	0.1957
GO:0010745	IPI00705441	Biological Process	Negative regulation of macrophage derived foam cell differentiation	10	1	246	0.2520
GO:0043407	IPI00709876	Biological Process	Negative regulation of MAP kinase activity	13	1	246	0.6113

GO:1901029	IPI00712858	Biological Process	Negative regulation of mitochondrial outer membrane permeabilization involved in apoptotic signaling pathway	12	1	246	0.1351
GO:0010823	IPI00712858	Biological Process	Negative regulation of mitochondrion organization	7	1	246	0.3044
GO:0032769	IPI00709876	Biological Process	Negative regulation of monooxygenase activity	7	1	246	0.1957
GO:0050686	IPI00712263	Biological Process	Negative regulation of mRNA processing	12	1	246	0.4407
GO:0048025	IPI00712263	Biological Process	Negative regulation of mRNA splicing, via spliceosome	13	1	246	0.4407
GO:0051148	IPI00712263	Biological Process	Negative regulation of muscle cell differentiation	9	1	246	0.1957
GO:0060547	IPI00706314	Biological Process	Negative regulation of necrotic cell death	8	1	246	0.1351
GO:0045665	IPI00685510	Biological Process	Negative regulation of neuron differentiation	11	1	246	0.3532
GO:0051534	IPI00690309	Biological Process	Negative regulation of NFAT protein import into nucleus	8	1	246	0.1351
GO:0045019	IPI00709876	Biological Process	Negative regulation of nitric oxide biosynthetic process	8	1	246	0.1351
GO:0051001	IPI00709876	Biological Process	Negative regulation of nitric-oxide synthase activity	8	1	246	0.1351
GO:0051771	IPI00690581	Biological Process	Negative regulation of nitric-oxide synthase biosynthetic process	9	1	246	0.1957
GO:0046823	IPI00690309	Biological Process	Negative regulation of nucleocytoplasmic transport	9	1	246	0.4799
GO:0010639	IPI00712858	Biological Process	Negative regulation of organelle organization	5	1	246	0.8876
GO:0051354	IPI00709876	Biological Process	Negative regulation of oxidoreductase activity	6	1	246	0.2520

GO:0010466	IPI00867230	Biological Process	Negative regulation of peptidase activity	10	1	246	0.7831
GO:0090278	IPI00715059	Biological Process	Negative regulation of peptide hormone secretion	11	1	246	0.2520
GO:0002792	IPI00715059	Biological Process	Negative regulation of peptide secretion	9	1	246	0.2520
GO:0033137	IPI00709876	Biological Process	Negative regulation of peptidyl-serine phosphorylation	10	1	246	0.2520
GO:0032515	IPI00698916	Biological Process	Negative regulation of phosphoprotein phosphatase activity	11	1	246	0.2520
GO:0042308	IPI00690309	Biological Process	Negative regulation of protein import into nucleus	6	1	246	0.3532
GO:0032513	IPI00698916	Biological Process	Negative regulation of protein phosphatase type 2B activity	12	1	246	0.1351
GO:0071901	IPI00709876	Biological Process	Negative regulation of protein serine/threonine kinase activity	12	1	246	0.6640
GO:0051224	IPI00690309	Biological Process	Negative regulation of protein transport	8	1	246	0.7300
GO:0031397	IPI00709876	Biological Process	Negative regulation of protein ubiquitination	12	1	246	0.5504
GO:0048261	IPI00866975	Biological Process	Negative regulation of receptor-mediated endocytosis	8	1	246	0.3044
GO:0060761	IPI00709876	Biological Process	Negative regulation of response to cytokine stimulus	8	1	246	0.4799
GO:0051048	IPI00715059	Biological Process	Negative regulation of secretion	7	1	246	0.5164
GO:0010894	IPI00705159	Biological Process	Negative regulation of steroid biosynthetic process	8	1	246	0.1957
GO:0090032	IPI00705159	Biological Process	Negative regulation of steroid hormone biosynthetic process	7	1	246	0.1351
GO:0045939	IPI00705159	Biological Process	Negative regulation of steroid metabolic process	8	1	246	0.1957

GO:0070233	IPI00703084	Biological Process	Negative regulation of T cell apoptotic process	13	1	246	0.1957
GO:0042992	IPI00690309	Biological Process	Negative regulation of transcription factor import into nucleus	7	1	246	0.1957
GO:0017148	IPI00712384	Biological Process	Negative regulation of translation	9	1	246	0.5819
GO:0090101	IPI00709876	Biological Process	Negative regulation of transmembrane receptor protein serine/threonine kinase signaling pathway	11	1	246	0.5819
GO:0032720	IPI00690581	Biological Process	Negative regulation of tumor necrosis factor production	9	1	246	0.3044
GO:0042518	IPI00710274	Biological Process	Negative regulation of tyrosine phosphorylation of Stat3 protein	12	1	246	0.1351
GO:0021675	IPI00876805	Biological Process	Nerve development	8	1	246	0.3532
GO:0061351	IPI00687578	Biological Process	Neural precursor cell proliferation	5	1	246	0.3532
GO:0061101	IPI00696748	Biological Process	Neuroendocrine cell differentiation	10	1	246	0.1351
GO:0060563	IPI00696748	Biological Process	Neuroepithelial cell differentiation	9	1	246	0.1351
GO:0007528	IPI00685510	Biological Process	Neuromuscular junction development	6	1	246	0.4407
GO:0050905	IPI00685510	Biological Process	Neuromuscular process	7	1	246	0.6113
GO:0050885	IPI00685510	Biological Process	Neuromuscular process controlling balance	8	1	246	0.5164
GO:0051402	IPI00685510	Biological Process	Neuron apoptotic process	9	1	246	0.5164
GO:0070997	IPI00685510	Biological Process	Neuron death	6	1	246	0.5164
GO:0042551	IPI00685510	Biological Process	Neuron maturation	11	1	246	0.2520
GO:0001764	IPI00687578	Biological Process	Neuron migration	8	1	246	0.5164
GO:0097485	IPI00685510	Biological Process	Neuron projection guidance	6	1	246	0.6876
GO:0008038	IPI00685510	Biological Process	Neuron recognition	6	1	246	0.2520
GO:0016322	IPI00685510	Biological Process	Neuron remodeling	12	1	246	0.2520
GO:0042135	IPI00698059	Biological Process	Neurotransmitter catabolic process	6	1	246	0.2520
GO:0042133	IPI00698059	Biological Process	Neurotransmitter metabolic process	5	1	246	0.3044
GO:0045213	IPI00876805	Biological Process	Neurotransmitter receptor metabolic process	7	1	246	0.1351

GO:0007269	IPI00696116	Biological Process	Neurotransmitter secretion	8	1	246	0.4799
GO:0046460	IPI00703084	Biological Process	Neutral lipid biosynthetic process	6	1	246	0.2520
GO:0030593	IPI00697595	Biological Process	Neutrophil chemotaxis	7	1	246	0.3532
GO:0038061	IPI00717764	Biological Process	NIK/NF-kappaB signaling	8	1	246	0.2520
GO:0033484	IPI00709876	Biological Process	Nitric oxide homeostasis	8	1	246	0.1351
GO:0007219	IPI00688704	Biological Process	Notch signaling pathway	8	1	246	0.4799
GO:0000280	IPI00693579	Biological Process	Nuclear division	6	1	246	0.8380
GO:0051168	IPI00700228	Biological Process	Nuclear export	9	1	246	0.8380
GO:0051170	IPI00714269	Biological Process	Nuclear import	9	1	246	0.7831
GO:0051292	IPI00689226	Biological Process	Nuclear pore complex assembly	7	1	246	0.1351
GO:0006999	IPI00689226	Biological Process	Nuclear pore organization	6	1	246	0.1957
GO:0000956	IPI00704902	Biological Process	Nuclear-transcribed mRNA catabolic process	10	1	246	0.6113
GO:0000288	IPI00704902	Biological Process	Nuclear-transcribed mRNA catabolic process, deadenylation-dependent decay	11	1	246	0.2520
GO:0000289	IPI00704902	Biological Process	Nuclear-transcribed mRNA poly(A) tail shortening	12	1	246	0.1351
GO:0090305	IPI00709124	Biological Process	Nucleic acid phosphodiester bond hydrolysis	8	1	246	0.8601
GO:0033866	IPI00704332	Biological Process	Nucleoside bisphosphate biosynthetic process	9	1	246	0.3044
GO:0033869	IPI00694430	Biological Process	Nucleoside bisphosphate catabolic process	9	1	246	0.1351
GO:0009125	IPI00710377	Biological Process	Nucleoside monophosphate catabolic process	9	1	246	0.7666
GO:0015858	IPI00691086	Biological Process	Nucleoside transport	6	1	246	0.1957
GO:0009143	IPI00710377	Biological Process	Nucleoside triphosphate catabolic process	9	1	246	0.9419
GO:0051647	IPI00876805	Biological Process	Nucleus localization	6	1	246	0.3985
GO:0006997	IPI00689226	Biological Process	Nucleus organization	5	1	246	0.7666
GO:0042475	IPI00710274	Biological Process	Odontogenesis of dentin-containing tooth	10	1	246	0.3985
GO:0021772	IPI00687578	Biological Process	Olfactory bulb development	13	1	246	0.2520
GO:0021988	IPI00687578	Biological Process	Olfactory lobe development	12	1	246	0.3044
GO:0021553	IPI00876805	Biological Process	Olfactory nerve development	10	1	246	0.1351
GO:0021627	IPI00876805	Biological Process	Olfactory nerve morphogenesis	11	1	246	0.1351

GO:0021629	IPI00876805	Biological Process	Olfactory nerve structural organization	12	1	246	0.1351
GO:0048285	IPI00693579	Biological Process	Organelle fission	5	1	246	0.9029
GO:0048284	IPI00843305	Biological Process	Organelle fusion	5	1	246	0.6640
GO:0015849	IPI00708597	Biological Process	Organic acid transport	5	1	246	0.6113
GO:1901617	IPI00690789	Biological Process	Organic hydroxy compound biosynthetic process	6	1	246	0.8601
GO:1901616	IPI00698059	Biological Process	Organic hydroxy compound catabolic process	6	1	246	0.5819
GO:0015850	IPI00698823	Biological Process	Organic hydroxy compound transport	5	1	246	0.5819
GO:0001649	IPI00707857	Biological Process	Osteoblast differentiation	7	1	246	0.3044
GO:0006733	IPI00695952	Biological Process	Oxidoreduction coenzyme metabolic process	7	1	246	0.7983
GO:0009405	IPI00707776	Biological Process	Pathogenesis	4	1	246	0.1351
GO:0007389	IPI00686595	Biological Process	Pattern specification process	6	1	246	0.6113
GO:0043043	IPI00876805	Biological Process	Peptide biosynthetic process	8	1	246	0.4407
GO:0018149	IPI00731432	Biological Process	Peptide cross-linking	8	1	246	0.5504
GO:0018209	IPI00690309	Biological Process	Peptidyl-serine modification	9	1	246	0.6640
GO:0018105	IPI00690309	Biological Process	Peptidyl-serine phosphorylation	9	1	246	0.5504
GO:0018210	IPI00690309	Biological Process	Peptidyl-threonine modification	9	1	246	0.3532
GO:0018107	IPI00690309	Biological Process	Peptidyl-threonine phosphorylation	9	1	246	0.2520
GO:0007422	IPI00876805	Biological Process	Peripheral nervous system development	8	1	246	0.4799
GO:0019336	IPI00698059	Biological Process	Phenol-containing compound catabolic process	7	1	246	0.1957
GO:0018958	IPI00698059	Biological Process	Phenol-containing compound metabolic process	6	1	246	0.3044
GO:0055062	IPI00703813	Biological Process	Phosphate ion homeostasis	10	1	246	0.2520
GO:0006654	IPI00706879	Biological Process	Phosphatidic acid biosynthetic process	8	1	246	0.1957
GO:0046473	IPI00706879	Biological Process	Phosphatidic acid metabolic process	9	1	246	0.1957
GO:0046470	IPI00839150	Biological Process	Phosphatidylcholine metabolic process	9	1	246	0.4799
GO:0014065	IPI00715059	Biological Process	Phosphatidylinositol 3-kinase signaling	10	1	246	0.3044

GO:0048015	IPI00715059	Biological Process	Phosphatidylinositol-mediated signaling	9	1	246	0.5164
GO:0055091	IPI00703084	Biological Process	Phospholipid homeostasis	9	1	246	0.1957
GO:0051875	IPI00718152	Biological Process	Pigment granule localization	6	1	246	0.1351
GO:0051904	IPI00718152	Biological Process	Pigment granule transport	7	1	246	0.1351
GO:0043473	IPI00718152	Biological Process	Pigmentation	4	1	246	0.3985
GO:0002317	IPI00705583	Biological Process	Plasma cell differentiation	10	1	246	0.1957
GO:0001778	IPI00843305	Biological Process	Plasma membrane repair	6	1	246	0.2520
GO:0000271	IPI00699039	Biological Process	Polysaccharide biosynthetic process	7	1	246	0.5819
GO:0046931	IPI00689226	Biological Process	Pore complex assembly	8	1	246	0.1351
GO:0006779	IPI00691093	Biological Process	Porphyrin-containing compound biosynthetic process	8	1	246	0.3985
GO:0006778	IPI00691093	Biological Process	Porphyrin-containing compound metabolic process	7	1	246	0.5819
GO:0043128	IPI00685617	Biological Process	Positive regulation of 1-phosphatidylinositol 4-kinase activity	10	1	246	0.1351
GO:0030838	IPI00690309	Biological Process	Positive regulation of actin filament polymerization	11	1	246	0.5164
GO:0042104	IPI00703084	Biological Process	Positive regulation of activated T cell proliferation	9	1	246	0.1957
GO:0043065	IPI00709124	Biological Process	Positive regulation of apoptotic process	9	1	246	0.9677
GO:2001235	IPI00709876	Biological Process	Positive regulation of apoptotic signaling pathway	9	1	246	0.7300
GO:0010524	IPI00709876	Biological Process	Positive regulation of calcium ion transport into cytosol	15	1	246	0.3532
GO:0045913	IPI00715059	Biological Process	Positive regulation of carbohydrate metabolic process	7	1	246	0.4407
GO:0043089	IPI00866858	Biological Process	Positive regulation of Cdc42 GTPase activity	18	1	246	0.2520
GO:0045787	IPI00685510	Biological Process	Positive regulation of cell cycle	6	1	246	0.5819
GO:0071158	IPI00698213	Biological Process	Positive regulation of cell cycle arrest	9	1	246	0.2520

GO:0010942	IPI00709124	Biological Process	Positive regulation of cell death	7	1	246	0.9721
GO:0010720	IPI00707857	Biological Process	Positive regulation of cell development	9	1	246	0.7096
GO:0051781	IPI00705237	Biological Process	Positive regulation of cell division	7	1	246	0.3985
GO:0010770	IPI00707857	Biological Process	Positive regulation of cell morphogenesis involved in differentiation	10	1	246	0.3532
GO:0031346	IPI00690309	Biological Process	Positive regulation of cell projection organization	7	1	246	0.7300
GO:0010676	IPI00715059	Biological Process	Positive regulation of cellular carbohydrate metabolic process	7	1	246	0.3044
GO:1900078	IPI00715059	Biological Process	Positive regulation of cellular response to insulin stimulus	11	1	246	0.3044
GO:0032467	IPI00705237	Biological Process	Positive regulation of cytokinesis	9	1	246	0.2520
GO:0010718	IPI00707857	Biological Process	Positive regulation of epithelial to mesenchymal transition	12	1	246	0.3044
GO:0070374	IPI00717764	Biological Process	Positive regulation of ERK1 and ERK2 cascade	12	1	246	0.3985
GO:0090004	IPI00866858	Biological Process	Positive regulation of establishment of protein localization to plasma membrane	8	1	246	0.3532
GO:2001238	IPI00709876	Biological Process	Positive regulation of extrinsic apoptotic signaling pathway	10	1	246	0.4407
GO:0045600	IPI00707776	Biological Process	Positive regulation of fat cell differentiation	9	1	246	0.3044
GO:0032000	IPI00715059	Biological Process	Positive regulation of fatty acid beta-oxidation	12	1	246	0.1957
GO:0046326	IPI00715059	Biological Process	Positive regulation of glucose import	11	1	246	0.4407
GO:2001275	IPI00715059	Biological Process	Positive regulation of glucose import in response to insulin stimulus	7	1	246	0.3044

GO:0010907	IPI00715059	Biological Process	Positive regulation of glucose metabolic process	8	1	246	0.3044
GO:0010828	IPI00715059	Biological Process	Positive regulation of glucose transport	10	1	246	0.4799
GO:0045725	IPI00715059	Biological Process	Positive regulation of glycogen biosynthetic process	11	1	246	0.2520
GO:0070875	IPI00715059	Biological Process	Positive regulation of glycogen metabolic process	10	1	246	0.2520
GO:0045927	IPI00703084	Biological Process	Positive regulation of growth	5	1	246	0.7490
GO:0045823	IPI00705159	Biological Process	Positive regulation of heart contraction	10	1	246	0.1957
GO:0032846	IPI00709876	Biological Process	Positive regulation of homeostatic process	6	1	246	0.4407
GO:2001244	IPI00709876	Biological Process	Positive regulation of intrinsic apoptotic signaling pathway	10	1	246	0.5504
GO:0010592	IPI00690309	Biological Process	Positive regulation of lamellipodium assembly	9	1	246	0.1957
GO:0070665	IPI00703084	Biological Process	Positive regulation of leukocyte proliferation	6	1	246	0.6386
GO:0046889	IPI00690309	Biological Process	Positive regulation of lipid biosynthetic process	7	1	246	0.3985
GO:0050996	IPI00715059	Biological Process	Positive regulation of lipid catabolic process	8	1	246	0.3532
GO:0050671	IPI00703084	Biological Process	Positive regulation of lymphocyte proliferation	8	1	246	0.6113
GO:0051044	IPI00716725	Biological Process	Positive regulation of membrane protein ectodomain proteolysis	8	1	246	0.2520
GO:0002053	IPI00715059	Biological Process	Positive regulation of mesenchymal cell proliferation	7	1	246	0.2520
GO:0048554	IPI00709876	Biological Process	Positive regulation of metalloenzyme activity	6	1	246	0.1351

GO:0051561	IPI00711457	Biological Process	Positive regulation of mitochondrial calcium ion concentration	12	1	246	0.2520
GO:0045931	IPI00685510	Biological Process	Positive regulation of mitotic cell cycle	8	1	246	0.4799
GO:0032946	IPI00703084	Biological Process	Positive regulation of mononuclear cell proliferation	7	1	246	0.6113
GO:0040018	IPI00703084	Biological Process	Positive regulation of multicellular organism growth	7	1	246	0.3532
GO:0051149	IPI00690309	Biological Process	Positive regulation of muscle cell differentiation	9	1	246	0.2520
GO:0045933	IPI00705159	Biological Process	Positive regulation of muscle contraction	9	1	246	0.1957
GO:0010831	IPI00690309	Biological Process	Positive regulation of myotube differentiation	11	1	246	0.1351
GO:0033138	IPI00709876	Biological Process	Positive regulation of peptidyl-serine phosphorylation	10	1	246	0.3044
GO:0010800	IPI00717764	Biological Process	Positive regulation of peptidyl-threonine phosphorylation	10	1	246	0.1957
GO:0043552	IPI00715059	Biological Process	Positive regulation of phosphatidylinositol 3-kinase activity	10	1	246	0.3985
GO:0043068	IPI00709124	Biological Process	Positive regulation of programmed cell death	8	1	246	0.9700
GO:0031954	IPI00847122	Biological Process	Positive regulation of protein autophosphorylation	11	1	246	0.1957
GO:0045732	IPI00716725	Biological Process	Positive regulation of protein catabolic process	9	1	246	0.5819
GO:0031334	IPI00690309	Biological Process	Positive regulation of protein complex assembly	7	1	246	0.7831
GO:0046827	IPI01028468	Biological Process	Positive regulation of protein export from nucleus	9	1	246	0.3044
GO:0042307	IPI00714269	Biological Process	Positive regulation of protein import into nucleus	6	1	246	0.5819

GO:0051897	IPI00717764	Biological Process	Positive regulation of protein kinase B signaling	10	1	246	0.4407
GO:0032273	IPI00690309	Biological Process	Positive regulation of protein polymerization	8	1	246	0.6113
GO:0045862	IPI00716725	Biological Process	Positive regulation of proteolysis	9	1	246	0.6113
GO:0032851	IPI00692668	Biological Process	Positive regulation of Rab GTPase activity	17	1	246	0.5164
GO:0032855	IPI00866858	Biological Process	Positive regulation of Rac GTPase activity	18	1	246	0.3044
GO:2001016	IPI00690309	Biological Process	Positive regulation of skeletal muscle cell differentiation	9	1	246	0.1351
GO:2000648	IPI00715059	Biological Process	Positive regulation of stem cell proliferation	6	1	246	0.3532
GO:0032874	IPI00697917	Biological Process	Positive regulation of stress-activated MAPK cascade	12	1	246	0.4799
GO:0070304	IPI00697917	Biological Process	Positive regulation of stress-activated protein kinase signaling cascade	10	1	246	0.5164
GO:0051155	IPI00690309	Biological Process	Positive regulation of striated muscle cell differentiation	10	1	246	0.1351
GO:0045989	IPI00705159	Biological Process	Positive regulation of striated muscle contraction	10	1	246	0.1351
GO:0042102	IPI00703084	Biological Process	Positive regulation of T cell proliferation	8	1	246	0.5504
GO:0045945	IPI00690309	Biological Process	Positive regulation of transcription from RNA polymerase III promoter	13	1	246	0.1351
GO:0032760	IPI00713474	Biological Process	Positive regulation of tumor necrosis factor production	9	1	246	0.3985
GO:0045907	IPI00709876	Biological Process	Positive regulation of vasoconstriction	11	1	246	0.1351
GO:0001516	IPI00714621	Biological Process	Prostaglandin biosynthetic process	10	1	246	0.2520
GO:0006693	IPI00714621	Biological Process	Prostaglandin metabolic process	10	1	246	0.3985
GO:0046457	IPI00714621	Biological Process	Prostanoid biosynthetic process	9	1	246	0.2520

GO:0006692	IPI00714621	Biological Process	Prostanoid metabolic process	9	1	246	0.3985
GO:0010498	IPI00690581	Biological Process	Proteasomal protein catabolic process	9	1	246	0.8876
GO:0043161	IPI00690581	Biological Process	Proteasome-mediated ubiquitin-dependent protein catabolic process	10	1	246	0.8876
GO:0008213	IPI00707776	Biological Process	Protein alkylation	8	1	246	0.5164
GO:0009305	IPI00727591	Biological Process	Protein biotinylation	8	1	246	0.1351
GO:0035786	IPI00711457	Biological Process	Protein complex oligomerization	8	1	246	0.1351
GO:0006470	IPI00906031	Biological Process	Protein dephosphorylation	8	1	246	0.8494
GO:0006486	IPI00698823	Biological Process	Protein glycosylation	8	1	246	0.8791
GO:0017038	IPI00714269	Biological Process	Protein import	5	1	246	0.8876
GO:0006606	IPI00714269	Biological Process	Protein import into nucleus	6	1	246	0.7831
GO:0043491	IPI00715059	Biological Process	Protein kinase B signaling	8	1	246	0.2520
GO:0070972	IPI00716830	Biological Process	Protein localization to endoplasmic reticulum	8	1	246	0.5819
GO:0070585	IPI00707815	Biological Process	Protein localization to mitochondrion	8	1	246	0.6876
GO:0072659	IPI00696116	Biological Process	Protein localization to plasma membrane	6	1	246	0.7831
GO:0022417	IPI00707815	Biological Process	Protein maturation by protein folding	8	1	246	0.1351
GO:0006479	IPI00707776	Biological Process	Protein methylation	6	1	246	0.5164
GO:0006513	IPI00718678	Biological Process	Protein monoubiquitination	11	1	246	0.6113
GO:0051258	IPI00708767	Biological Process	Protein polymerization	9	1	246	0.6640
GO:0000209	IPI00718678	Biological Process	Protein polyubiquitination	11	1	246	0.7300
GO:0016485	IPI00685804	Biological Process	Protein processing	7	1	246	0.5819
GO:0006612	IPI00703954	Biological Process	Protein targeting to membrane	7	1	246	0.7300
GO:0006626	IPI00707815	Biological Process	Protein targeting to mitochondrion	6	1	246	0.6640
GO:0044744	IPI00714269	Biological Process	Protein targeting to nucleus	7	1	246	0.7831
GO:0030166	IPI00699039	Biological Process	Proteoglycan biosynthetic process	8	1	246	0.1957
GO:0006029	IPI00699039	Biological Process	Proteoglycan metabolic process	7	1	246	0.3985
GO:0034033	IPI00704332	Biological Process	Purine nucleoside bisphosphate biosynthetic process	10	1	246	0.3044
GO:0034034	IPI00694430	Biological Process	Purine nucleoside bisphosphate catabolic process	10	1	246	0.1351

GO:0009128	IPI00710377	Biological Process	Purine nucleoside monophosphate catabolic process	10	1	246	0.7666
GO:0009146	IPI00710377	Biological Process	Purine nucleoside triphosphate catabolic process	10	1	246	0.9375
GO:0009169	IPI00710377	Biological Process	Purine ribonucleoside monophosphate catabolic process	11	1	246	0.7666
GO:0009207	IPI00710377	Biological Process	Purine ribonucleoside triphosphate catabolic process	11	1	246	0.9327
GO:1901663	IPI00695952	Biological Process	Quinone biosynthetic process	7	1	246	0.3532
GO:1901661	IPI00695952	Biological Process	Quinone metabolic process	6	1	246	0.3985
GO:0032486	IPI00847122	Biological Process	Rap protein signal transduction	10	1	246	0.1351
GO:0072593	IPI00716157	Biological Process	Reactive oxygen species metabolic process	5	1	246	0.7490
GO:2000286	IPI00709876	Biological Process	Receptor internalization involved in canonical Wnt signaling pathway	8	1	246	0.1957
GO:0001881	IPI00713474	Biological Process	Receptor recycling	7	1	246	0.1351
GO:0000725	IPI00712399	Biological Process	Recombinational repair	10	1	246	0.3044
GO:0003002	IPI00686595	Biological Process	Regionalization	7	1	246	0.3985
GO:0043126	IPI00685617	Biological Process	Regulation of 1-phosphatidylinositol 4-kinase activity	9	1	246	0.1351
GO:0030832	IPI00690309	Biological Process	Regulation of actin filament length	8	1	246	0.9162
GO:0030833	IPI00690309	Biological Process	Regulation of actin filament polymerization	10	1	246	0.8791
GO:0008064	IPI00690309	Biological Process	Regulation of actin polymerization or depolymerization	9	1	246	0.9162
GO:0046006	IPI00703084	Biological Process	Regulation of activated T cell proliferation	9	1	246	0.2520
GO:0070235	IPI00703084	Biological Process	Regulation of activation-induced cell death of T cells	8	1	246	0.1351
GO:0002673	IPI00690581	Biological Process	Regulation of acute inflammatory response	8	1	246	0.4407
GO:0002864	IPI00690581	Biological Process	Regulation of acute inflammatory response to antigenic stimulus	7	1	246	0.1957

GO:0000381	IPI00712263	Biological Process	Regulation of alternative mRNA splicing, via spliceosome	13	1	246	0.4799
GO:2000209	IPI00709876	Biological Process	Regulation of anoikis	9	1	246	0.2520
GO:0032312	IPI00688451	Biological Process	Regulation of ARF GTPase activity	16	1	246	0.5164
GO:0030193	IPI00709876	Biological Process	Regulation of blood coagulation	7	1	246	0.6386
GO:0050880	IPI00709876	Biological Process	Regulation of blood vessel size	8	1	246	0.5819
GO:0030510	IPI00709876	Biological Process	Regulation of BMP signaling pathway	14	1	246	0.3044
GO:0050878	IPI00709876	Biological Process	Regulation of body fluid levels	5	1	246	0.9419
GO:1901019	IPI00698916	Biological Process	Regulation of calcium ion transmembrane transporter activity	9	1	246	0.3044
GO:0010522	IPI00709876	Biological Process	Regulation of calcium ion transport into cytosol	14	1	246	0.5164
GO:0043255	IPI00715059	Biological Process	Regulation of carbohydrate biosynthetic process	7	1	246	0.5819
GO:0086004	IPI00708070	Biological Process	Regulation of cardiac muscle cell contraction	9	1	246	0.3532
GO:0086036	IPI00708070	Biological Process	Regulation of cardiac muscle cell membrane potential	6	1	246	0.3985
GO:0055117	IPI00708070	Biological Process	Regulation of cardiac muscle contraction	10	1	246	0.3532
GO:0061035	IPI00707776	Biological Process	Regulation of cartilage development	9	1	246	0.3532
GO:2001257	IPI00698916	Biological Process	Regulation of cation channel activity	9	1	246	0.3532
GO:0043088	IPI00866858	Biological Process	Regulation of Cdc42 GTPase activity	17	1	246	0.3044
GO:0032489	IPI00866858	Biological Process	Regulation of Cdc42 protein signal transduction	12	1	246	0.4407
GO:0071156	IPI00698213	Biological Process	Regulation of cell cycle arrest	8	1	246	0.3044
GO:0051302	IPI00705237	Biological Process	Regulation of cell division	6	1	246	0.6113
GO:0022604	IPI00707857	Biological Process	Regulation of cell morphogenesis	8	1	246	0.9626
GO:0010769	IPI00707857	Biological Process	Regulation of cell morphogenesis involved in differentiation	9	1	246	0.7666
GO:0060491	IPI00690309	Biological Process	Regulation of cell projection assembly	7	1	246	0.6113

GO:0031344	IPI00690309	Biological Process	Regulation of cell projection organization	6	1	246	0.9098
GO:0008361	IPI00690309	Biological Process	Regulation of cell size	6	1	246	0.3532
GO:0022407	IPI00710274	Biological Process	Regulation of cell-cell adhesion	6	1	246	0.6113
GO:0010810	IPI00707857	Biological Process	Regulation of cell-substrate adhesion	6	1	246	0.7831
GO:0032535	IPI00690309	Biological Process	Regulation of cellular component size	5	1	246	0.9375
GO:1900076	IPI00715059	Biological Process	Regulation of cellular response to insulin stimulus	10	1	246	0.5164
GO:0033044	IPI00693842	Biological Process	Regulation of chromosome organization	6	1	246	0.7300
GO:0050818	IPI00709876	Biological Process	Regulation of coagulation	6	1	246	0.6876
GO:0000079	IPI00696748	Biological Process	Regulation of cyclin-dependent protein serine/threonine kinase activity	12	1	246	0.3985
GO:0050707	IPI00703084	Biological Process	Regulation of cytokine secretion	8	1	246	0.4407
GO:0001959	IPI00709876	Biological Process	Regulation of cytokine-mediated signaling pathway	9	1	246	0.6876
GO:0032465	IPI00705237	Biological Process	Regulation of cytokinesis	8	1	246	0.4799
GO:0031347	IPI00690581	Biological Process	Regulation of defense response	6	1	246	0.9741
GO:0048638	IPI00707776	Biological Process	Regulation of developmental growth	6	1	246	0.3532
GO:0006275	IPI00710274	Biological Process	Regulation of DNA replication	10	1	246	0.5504
GO:0052548	IPI00867230	Biological Process	Regulation of endopeptidase activity	9	1	246	0.9375
GO:0001936	IPI00709876	Biological Process	Regulation of endothelial cell proliferation	7	1	246	0.5819
GO:0042058	IPI00685510	Biological Process	Regulation of epidermal growth factor receptor signaling pathway	12	1	246	0.3532
GO:0007176	IPI00685510	Biological Process	Regulation of epidermal growth factor-activated receptor activity	11	1	246	0.1957
GO:0030856	IPI00709876	Biological Process	Regulation of epithelial cell differentiation	8	1	246	0.5164
GO:0010632	IPI00845178	Biological Process	Regulation of epithelial cell migration	10	1	246	0.6876
GO:0010717	IPI00707857	Biological Process	Regulation of epithelial to mesenchymal transition	11	1	246	0.3985

GO:1901184	IPI00685510	Biological Process	Regulation of ERBB signaling pathway	11	1	246	0.3532
GO:0070372	IPI00717764	Biological Process	Regulation of ERK1 and ERK2 cascade	11	1	246	0.6876
GO:2000114	IPI00713979	Biological Process	Regulation of establishment of cell polarity	7	1	246	0.3532
GO:0090003	IPI00866858	Biological Process	Regulation of establishment of protein localization to plasma membrane	7	1	246	0.4799
GO:0032878	IPI00713979	Biological Process	Regulation of establishment or maintenance of cell polarity	6	1	246	0.3985
GO:0017157	IPI00696116	Biological Process	Regulation of exocytosis	7	1	246	0.6113
GO:2001236	IPI00709876	Biological Process	Regulation of extrinsic apoptotic signaling pathway	9	1	246	0.5164
GO:0045598	IPI00707776	Biological Process	Regulation of fat cell differentiation	8	1	246	0.5504
GO:0031998	IPI00715059	Biological Process	Regulation of fatty acid beta-oxidation	11	1	246	0.1957
GO:1901193	IPI00712384	Biological Process	Regulation of formation of translation preinitiation complex	10	1	246	0.1351
GO:0040029	IPI00698339	Biological Process	Regulation of gene expression, epigenetic	8	1	246	0.7096
GO:0043467	IPI00715059	Biological Process	Regulation of generation of precursor metabolites and energy	6	1	246	0.4799
GO:0010962	IPI00715059	Biological Process	Regulation of glucan biosynthetic process	9	1	246	0.3044
GO:0031946	IPI00705159	Biological Process	Regulation of glucocorticoid biosynthetic process	8	1	246	0.1957
GO:0031943	IPI00705159	Biological Process	Regulation of glucocorticoid metabolic process	6	1	246	0.1957
GO:0046324	IPI00715059	Biological Process	Regulation of glucose import	10	1	246	0.6113
GO:2001273	IPI00715059	Biological Process	Regulation of glucose import in response to insulin stimulus	6	1	246	0.3044
GO:0010827	IPI00715059	Biological Process	Regulation of glucose transport	9	1	246	0.6386
GO:0005979	IPI00715059	Biological Process	Regulation of glycogen biosynthetic process	10	1	246	0.3044

GO:0070873	IPI00715059	Biological Process	Regulation of glycogen metabolic process	9	1	246	0.3044
GO:0003420	IPI00707776	Biological Process	Regulation of growth plate cartilage chondrocyte proliferation	6	1	246	0.1351
GO:0002027	IPI00708070	Biological Process	Regulation of heart rate	10	1	246	0.3532
GO:0086091	IPI00708070	Biological Process	Regulation of heart rate by cardiac conduction	7	1	246	0.3044
GO:1900046	IPI00709876	Biological Process	Regulation of hemostasis	7	1	246	0.6386
GO:0035330	IPI00710274	Biological Process	Regulation of hippo signaling	9	1	246	0.1351
GO:0046885	IPI00705159	Biological Process	Regulation of hormone biosynthetic process	6	1	246	0.2520
GO:0010817	IPI00715066	Biological Process	Regulation of hormone levels	5	1	246	0.6386
GO:0032350	IPI00705159	Biological Process	Regulation of hormone metabolic process	5	1	246	0.2520
GO:0046883	IPI00715059	Biological Process	Regulation of hormone secretion	9	1	246	0.5164
GO:0050727	IPI00690581	Biological Process	Regulation of inflammatory response	7	1	246	0.8791
GO:0002861	IPI00690581	Biological Process	Regulation of inflammatory response to antigenic stimulus	6	1	246	0.3044
GO:0050796	IPI00715059	Biological Process	Regulation of insulin secretion	11	1	246	0.4799
GO:0032675	IPI00690581	Biological Process	Regulation of interleukin-6 production	7	1	246	0.4799
GO:0033146	IPI00707776	Biological Process	Regulation of intracellular estrogen receptor signaling pathway	10	1	246	0.2520
GO:0033143	IPI00707776	Biological Process	Regulation of intracellular steroid hormone receptor signaling pathway	9	1	246	0.4799
GO:2001242	IPI00709876	Biological Process	Regulation of intrinsic apoptotic signaling pathway	9	1	246	0.6113
GO:2000021	IPI00709876	Biological Process	Regulation of ion homeostasis	6	1	246	0.6876
GO:0034765	IPI00698916	Biological Process	Regulation of ion transmembrane transport	7	1	246	0.6640
GO:0032412	IPI00698916	Biological Process	Regulation of ion transmembrane transporter activity	8	1	246	0.5504
GO:0010591	IPI00690309	Biological Process	Regulation of lamellipodium assembly	8	1	246	0.2520

GO:2000106	IPI00703084	Biological Process	Regulation of leukocyte apoptotic process	10	1	246	0.3985
GO:0070663	IPI00703084	Biological Process	Regulation of leukocyte proliferation	6	1	246	0.7490
GO:0060191	IPI00698916	Biological Process	Regulation of lipase activity	7	1	246	0.6640
GO:0050994	IPI00715059	Biological Process	Regulation of lipid catabolic process	7	1	246	0.4407
GO:0010883	IPI00705441	Biological Process	Regulation of lipid storage	7	1	246	0.3985
GO:0051004	IPI00698916	Biological Process	Regulation of lipoprotein lipase activity	8	1	246	0.5164
GO:0070228	IPI00703084	Biological Process	Regulation of lymphocyte apoptotic process	11	1	246	0.3044
GO:0050670	IPI00703084	Biological Process	Regulation of lymphocyte proliferation	7	1	246	0.7490
GO:0010743	IPI00705441	Biological Process	Regulation of macrophage derived foam cell differentiation	9	1	246	0.3985
GO:0051043	IPI00716725	Biological Process	Regulation of membrane protein ectodomain proteolysis	7	1	246	0.2520
GO:0010464	IPI00715059	Biological Process	Regulation of mesenchymal cell proliferation	7	1	246	0.3532
GO:0048552	IPI00709876	Biological Process	Regulation of metalloenzyme activity	6	1	246	0.1351
GO:1901028	IPI00712858	Biological Process	Regulation of mitochondrial outer membrane permeabilization involved in apoptotic signaling pathway	11	1	246	0.1957
GO:0010821	IPI00712858	Biological Process	Regulation of mitochondrion organization	6	1	246	0.6386
GO:0007346	IPI00685510	Biological Process	Regulation of mitotic cell cycle	7	1	246	0.8956
GO:0032944	IPI00703084	Biological Process	Regulation of mononuclear cell proliferation	7	1	246	0.7490
GO:0032768	IPI00709876	Biological Process	Regulation of monooxygenase activity	7	1	246	0.4407
GO:0050684	IPI00712263	Biological Process	Regulation of mRNA processing	11	1	246	0.7831
GO:0048024	IPI00712263	Biological Process	Regulation of mRNA splicing, via spliceosome	12	1	246	0.7300
GO:0048634	IPI00690309	Biological Process	Regulation of muscle organ development	9	1	246	0.3532
GO:1901861	IPI00690309	Biological Process	Regulation of muscle tissue development	7	1	246	0.3532
GO:0010830	IPI00690309	Biological Process	Regulation of myotube differentiation	10	1	246	0.1957

GO:0010939	IPI00706314	Biological Process	Regulation of necrotic cell death	7	1	246	0.2520
GO:0051960	IPI00685510	Biological Process	Regulation of nervous system development	8	1	246	0.9567
GO:0050767	IPI00685510	Biological Process	Regulation of neurogenesis	9	1	246	0.9534
GO:0045664	IPI00685510	Biological Process	Regulation of neuron differentiation	10	1	246	0.9029
GO:0051532	IPI00690309	Biological Process	Regulation of NFAT protein import into nucleus	11	1	246	0.1351
GO:1901222	IPI00717764	Biological Process	Regulation of NIK/NF-kappaB signaling	9	1	246	0.2520
GO:0045428	IPI00709876	Biological Process	Regulation of nitric oxide biosynthetic process	8	1	246	0.4799
GO:0080164	IPI00690581	Biological Process	Regulation of nitric oxide metabolic process	6	1	246	0.1351
GO:0050999	IPI00709876	Biological Process	Regulation of nitric-oxide synthase activity	8	1	246	0.3044
GO:0051769	IPI00690581	Biological Process	Regulation of nitric-oxide synthase biosynthetic process	8	1	246	0.1957
GO:2000027	IPI00707776	Biological Process	Regulation of organ morphogenesis	9	1	246	0.6113
GO:0051341	IPI00709876	Biological Process	Regulation of oxidoreductase activity	6	1	246	0.5164
GO:0052547	IPI00867230	Biological Process	Regulation of peptidase activity	8	1	246	0.9419
GO:0090276	IPI00715059	Biological Process	Regulation of peptide hormone secretion	10	1	246	0.4799
GO:0002791	IPI00715059	Biological Process	Regulation of peptide secretion	8	1	246	0.4799
GO:0090087	IPI00715059	Biological Process	Regulation of peptide transport	7	1	246	0.4799
GO:0033135	IPI00709876	Biological Process	Regulation of peptidyl-serine phosphorylation	9	1	246	0.3985
GO:0010799	IPI00717764	Biological Process	Regulation of peptidyl-threonine phosphorylation	9	1	246	0.1957
GO:0043551	IPI00715059	Biological Process	Regulation of phosphatidylinositol 3-kinase activity	9	1	246	0.3985
GO:0043666	IPI00698916	Biological Process	Regulation of phosphoprotein phosphatase activity	10	1	246	0.5164
GO:0032885	IPI00715059	Biological Process	Regulation of polysaccharide biosynthetic process	8	1	246	0.3532

GO:0032881	IPI00715059	Biological Process	Regulation of polysaccharide metabolic process	7	1	246	0.3532
GO:0031952	IPI00847122	Biological Process	Regulation of protein autophosphorylation	10	1	246	0.4407
GO:0042176	IPI00716725	Biological Process	Regulation of protein catabolic process	8	1	246	0.9098
GO:0043254	IPI00690309	Biological Process	Regulation of protein complex assembly	6	1	246	0.9677
GO:0046825	IPI01028468	Biological Process	Regulation of protein export from nucleus	8	1	246	0.5164
GO:0051896	IPI00717764	Biological Process	Regulation of protein kinase B signaling	9	1	246	0.6113
GO:0032512	IPI00698916	Biological Process	Regulation of protein phosphatase type 2B activity	11	1	246	0.1351
GO:0032271	IPI00690309	Biological Process	Regulation of protein polymerization	7	1	246	0.9221
GO:0050708	IPI00703084	Biological Process	Regulation of protein secretion	7	1	246	0.6386
GO:0031396	IPI00709876	Biological Process	Regulation of protein ubiquitination	11	1	246	0.8125
GO:0032313	IPI00692668	Biological Process	Regulation of Rab GTPase activity	16	1	246	0.5164
GO:0032483	IPI00692668	Biological Process	Regulation of Rab protein signal transduction	11	1	246	0.5164
GO:0032314	IPI00690309	Biological Process	Regulation of Rac GTPase activity	17	1	246	0.1957
GO:0035020	IPI00690309	Biological Process	Regulation of Rac protein signal transduction	11	1	246	0.3044
GO:0010469	IPI00685510	Biological Process	Regulation of receptor activity	8	1	246	0.2520
GO:0048259	IPI00866975	Biological Process	Regulation of receptor-mediated endocytosis	7	1	246	0.5504
GO:0060759	IPI00709876	Biological Process	Regulation of response to cytokine stimulus	7	1	246	0.7096
GO:0060314	IPI00698916	Biological Process	Regulation of ryanodine-sensitive calcium-release channel activity	10	1	246	0.1957
GO:2001014	IPI00690309	Biological Process	Regulation of skeletal muscle cell differentiation	8	1	246	0.1957
GO:0048742	IPI00690309	Biological Process	Regulation of skeletal muscle fiber development	11	1	246	0.1957
GO:0048641	IPI00690309	Biological Process	Regulation of skeletal muscle tissue development	11	1	246	0.2520

GO:0006940	IPI00709876	Biological Process	Regulation of smooth muscle contraction	9	1	246	0.2520
GO:0002028	IPI00705159	Biological Process	Regulation of sodium ion transport	9	1	246	0.4407
GO:2000736	IPI00707857	Biological Process	Regulation of stem cell differentiation	8	1	246	0.4407
GO:0072091	IPI00715059	Biological Process	Regulation of stem cell proliferation	6	1	246	0.4799
GO:0090030	IPI00705159	Biological Process	Regulation of steroid hormone biosynthetic process	7	1	246	0.1957
GO:0019218	IPI00705159	Biological Process	Regulation of steroid metabolic process	7	1	246	0.7300
GO:0032872	IPI00697917	Biological Process	Regulation of stress-activated MAPK cascade	11	1	246	0.8125
GO:0070302	IPI00697917	Biological Process	Regulation of stress-activated protein kinase signaling cascade	9	1	246	0.8258
GO:0051153	IPI00690309	Biological Process	Regulation of striated muscle cell differentiation	9	1	246	0.2520
GO:0016202	IPI00690309	Biological Process	Regulation of striated muscle tissue development	10	1	246	0.3532
GO:0050803	IPI00685510	Biological Process	Regulation of synapse structure or activity	5	1	246	0.2520
GO:0070232	IPI00703084	Biological Process	Regulation of T cell apoptotic process	12	1	246	0.1957
GO:0042129	IPI00703084	Biological Process	Regulation of T cell proliferation	8	1	246	0.6640
GO:0032204	IPI00693842	Biological Process	Regulation of telomere maintenance	10	1	246	0.1957
GO:0003057	IPI00709876	Biological Process	Regulation of the force of heart contraction by chemical signal	11	1	246	0.1351
GO:0042990	IPI00690309	Biological Process	Regulation of transcription factor import into nucleus	10	1	246	0.5164
GO:0006359	IPI00690309	Biological Process	Regulation of transcription from RNA polymerase III promoter	12	1	246	0.1957
GO:0006446	IPI00712384	Biological Process	Regulation of translational initiation	9	1	246	0.6386
GO:0090092	IPI00709876	Biological Process	Regulation of transmembrane receptor protein serine/threonine kinase signaling pathway	10	1	246	0.7096

GO:0034762	IPI00698916	Biological Process	Regulation of transmembrane transport	6	1	246	0.7300
GO:0022898	IPI00698916	Biological Process	Regulation of transmembrane transporter activity	7	1	246	0.5819
GO:0032409	IPI00698916	Biological Process	Regulation of transporter activity	7	1	246	0.6640
GO:0035150	IPI00709876	Biological Process	Regulation of tube size	6	1	246	0.5819
GO:0042516	IPI00710274	Biological Process	Regulation of tyrosine phosphorylation of Stat3 protein	11	1	246	0.1351
GO:0019229	IPI00709876	Biological Process	Regulation of vasoconstriction	10	1	246	0.1351
GO:0061041	IPI00709876	Biological Process	Regulation of wound healing	7	1	246	0.7096
GO:0072001	IPI00699039	Biological Process	Renal system development	8	1	246	0.7831
GO:0019098	IPI00685510	Biological Process	Reproductive behavior	4	1	246	0.3532
GO:0017062	IPI00688474	Biological Process	Respiratory chain complex III assembly	10	1	246	0.1957
GO:0097305	IPI00702768	Biological Process	Response to alcohol	6	1	246	0.6113
GO:0034976	IPI00690581	Biological Process	Response to endoplasmic reticulum stress	6	1	246	0.7096
GO:0042542	IPI00716157	Biological Process	Response to hydrogen peroxide	7	1	246	0.6640
GO:0055093	IPI00709876	Biological Process	Response to hyperoxia	5	1	246	0.2520
GO:0036296	IPI00709876	Biological Process	Response to increased oxygen levels	6	1	246	0.2520
GO:0070555	IPI00717764	Biological Process	Response to interleukin-1	7	1	246	0.4407
GO:0070741	IPI00717764	Biological Process	Response to interleukin-6	7	1	246	0.3044
GO:0002931	IPI00709876	Biological Process	Response to ischemia	5	1	246	0.1351
GO:1901654	IPI00709876	Biological Process	Response to ketone	6	1	246	0.4407
GO:0009416	IPI00685510	Biological Process	Response to light stimulus	6	1	246	0.8699
GO:0032496	IPI00690581	Biological Process	Response to lipopolysaccharide	7	1	246	0.8125
GO:0055094	IPI00698823	Biological Process	Response to lipoprotein particle	5	1	246	0.1351
GO:0055098	IPI00698823	Biological Process	Response to low-density lipoprotein particle	6	1	246	0.1351
GO:0002237	IPI00690581	Biological Process	Response to molecule of bacterial origin	6	1	246	0.8380
GO:0007584	IPI00702768	Biological Process	Response to nutrient	7	1	246	0.3532
GO:0032570	IPI00709876	Biological Process	Response to progesterone	7	1	246	0.1351

GO:0000302	IPI00716157	Biological Process	Response to reactive oxygen species	6	1	246	0.8125
GO:0051775	IPI00690581	Biological Process	Response to redox state	4	1	246	0.1957
GO:0035966	IPI00690581	Biological Process	Response to topologically incorrect protein	5	1	246	0.7096
GO:0006986	IPI00690581	Biological Process	Response to unfolded protein	6	1	246	0.6876
GO:0009615	IPI00703084	Biological Process	Response to virus	7	1	246	0.8380
GO:0033273	IPI00702768	Biological Process	Response to vitamin	8	1	246	0.3044
GO:0033280	IPI00702768	Biological Process	Response to vitamin D	9	1	246	0.1957
GO:0009410	IPI00707815	Biological Process	Response to xenobiotic stimulus	5	1	246	0.2520
GO:0030970	IPI00690581	Biological Process	Retrograde protein transport, ER to cytosol	6	1	246	0.1957
GO:0007266	IPI00708244	Biological Process	Rho protein signal transduction	10	1	246	0.3985
GO:0022618	IPI00704902	Biological Process	Ribonucleoprotein complex assembly	8	1	246	0.8258
GO:0071826	IPI00704902	Biological Process	Ribonucleoprotein complex subunit organization	6	1	246	0.8380
GO:0034030	IPI00704332	Biological Process	Ribonucleoside bisphosphate biosynthetic process	10	1	246	0.3044
GO:0034031	IPI00694430	Biological Process	Ribonucleoside bisphosphate catabolic process	10	1	246	0.1351
GO:0009158	IPI00710377	Biological Process	Ribonucleoside monophosphate catabolic process	10	1	246	0.7666
GO:0009203	IPI00710377	Biological Process	Ribonucleoside triphosphate catabolic process	10	1	246	0.9327
GO:0042274	IPI00702948	Biological Process	Ribosomal small subunit biogenesis	7	1	246	0.5164
GO:0042254	IPI00702948	Biological Process	Ribosome biogenesis	6	1	246	0.9029
GO:0036260	IPI00703022	Biological Process	RNA capping	10	1	246	0.2520
GO:0006405	IPI00700228	Biological Process	RNA export from nucleus	8	1	246	0.6640
GO:0043631	IPI00685510	Biological Process	RNA polyadenylation	11	1	246	0.4407
GO:0050810	IPI00705159	Biological Process	Rregulation of steroid biosynthetic process	8	1	246	0.4407
GO:0016075	IPI01017508	Biological Process	rRNA catabolic process	10	1	246	0.1351
GO:0006364	IPI00702948	Biological Process	rRNA processing	11	1	246	0.8125
GO:0031529	IPI00690309	Biological Process	Ruffle organization	6	1	246	0.4407
GO:0014010	IPI00710274	Biological Process	Schwann cell proliferation	10	1	246	0.1957
GO:0019932	IPI00711457	Biological Process	Second-messenger-mediated signaling	8	1	246	0.5164
GO:0032941	IPI00709876	Biological Process	Secretion by tissue	5	1	246	0.5164
GO:0050953	IPI00707857	Biological Process	Sensory perception of light stimulus	8	1	246	0.3985

GO:0006465	IPI00685804	Biological Process	Signal peptide processing	8	1	246	0.3532
GO:0023061	IPI00696116	Biological Process	Signal release	7	1	246	0.5504
GO:0072331	IPI00698213	Biological Process	Signal transduction by p53 class mediator	8	1	246	0.5164
GO:0044801	IPI00843305	Biological Process	Single-organism membrane fusion	5	1	246	0.6876
GO:0044704	IPI00685510	Biological Process	Single-organism reproductive behavior	5	1	246	0.3532
GO:0000819	IPI00693579	Biological Process	Sister chromatid segregation	6	1	246	0.3985
GO:0035914	IPI00876805	Biological Process	Skeletal muscle cell differentiation	7	1	246	0.3985
GO:0030240	IPI00697648	Biological Process	Skeletal muscle thin filament assembly	10	1	246	0.2520
GO:0014866	IPI00697648	Biological Process	Skeletal myofibril assembly	9	1	246	0.2520
GO:0051563	IPI00685510	Biological Process	Smooth endoplasmic reticulum calcium ion homeostasis	12	1	246	0.1351
GO:0006814	IPI00703813	Biological Process	Sodium ion transport	8	1	246	0.1957
GO:0007283	IPI00712399	Biological Process	Spermatogenesis	7	1	246	0.9276
GO:0007051	IPI00687578	Biological Process	Spindle organization	7	1	246	0.5164
GO:0016126	IPI00690789	Biological Process	Sterol biosynthetic process	8	1	246	0.5504
GO:0015918	IPI00698823	Biological Process	Sterol transport	6	1	246	0.5504
GO:0002115	IPI00693452	Biological Process	Store-operated calcium entry	10	1	246	0.1351
GO:0034446	IPI00924039	Biological Process	Substrate adhesion-dependent cell spreading	9	1	246	0.4799
GO:0001967	IPI00685510	Biological Process	Suckling behavior	5	1	246	0.1351
GO:0044272	IPI00699039	Biological Process	Sulfur compound biosynthetic process	6	1	246	0.6640
GO:0007416	IPI00685510	Biological Process	Synapse assembly	6	1	246	0.3044
GO:0050808	IPI00685510	Biological Process	Synapse organization	5	1	246	0.6386
GO:0051124	IPI00685510	Biological Process	Synaptic growth at neuromuscular junction	7	1	246	0.1351
GO:0016079	IPI00696116	Biological Process	Synaptic vesicle exocytosis	9	1	246	0.3044
GO:0097479	IPI00696116	Biological Process	Synaptic vesicle localization	7	1	246	0.6640
GO:0048489	IPI00696116	Biological Process	Synaptic vesicle transport	8	1	246	0.6640
GO:0042110	IPI00698916	Biological Process	T cell activation	6	1	246	0.8125
GO:0021537	IPI00687578	Biological Process	Telencephalon development	11	1	246	0.7983
GO:0033014	IPI00691093	Biological Process	Tetrapyrrole biosynthetic process	7	1	246	0.4407
GO:0033013	IPI00691093	Biological Process	Tetrapyrrole metabolic process	6	1	246	0.6113
GO:0048771	IPI00709876	Biological Process	Tissue remodeling	5	1	246	0.5504
GO:0034505	IPI00707857	Biological Process	Tooth mineralization	10	1	246	0.1351

GO:0031929	IPI00690309	Biological Process	TOR signaling	8	1	246	0.1351
GO:0060343	IPI00707857	Biological Process	Trabecula formation	9	1	246	0.3532
GO:0061383	IPI00707857	Biological Process	Trabecula morphogenesis	5	1	246	0.3985
GO:0070634	IPI00710920	Biological Process	Transepithelial ammonium transport	9	1	246	0.1957
GO:0030321	IPI00710920	Biological Process	Transepithelial chloride transport	9	1	246	0.1351
GO:0070633	IPI00710920	Biological Process	Transepithelial transport	5	1	246	0.2520
GO:0000041	IPI00710664	Biological Process	Transition metal ion transport	8	1	246	0.6386
GO:0019432	IPI00703084	Biological Process	Triglyceride biosynthetic process	8	1	246	0.1957
GO:0072506	IPI00703813	Biological Process	Trivalent inorganic anion homeostasis	9	1	246	0.2520
GO:0070127	IPI00689365	Biological Process	tRNA aminoacylation for mitochondrial protein translation	10	1	246	0.1351
GO:0035148	IPI00699039	Biological Process	Tube formation	8	1	246	0.8258
GO:0006744	IPI00695952	Biological Process	Ubiquinone biosynthetic process	8	1	246	0.3532
GO:0006743	IPI00695952	Biological Process	Ubiquinone metabolic process	7	1	246	0.3532
GO:0001657	IPI00699039	Biological Process	Ureteric bud development	7	1	246	0.3985
GO:0060676	IPI00699039	Biological Process	Ureteric bud formation	10	1	246	0.1351
GO:0060675	IPI00699039	Biological Process	Ureteric bud morphogenesis	8	1	246	0.2520
GO:0001655	IPI00699039	Biological Process	Urogenital system development	7	1	246	0.8699
GO:0007034	IPI00698823	Biological Process	Vacuolar transport	6	1	246	0.7300
GO:0009099	IPI00715066	Biological Process	Valine biosynthetic process	9	1	246	0.1351
GO:0006573	IPI00715066	Biological Process	Valine metabolic process	8	1	246	0.3532
GO:0003018	IPI00709876	Biological Process	Vascular process in circulatory system	7	1	246	0.6386
GO:0042310	IPI00709876	Biological Process	Vasoconstriction	9	1	246	0.2520
GO:0086005	IPI00708070	Biological Process	Ventricular cardiac muscle cell action potential	11	1	246	0.3044
GO:0003229	IPI00708070	Biological Process	Ventricular cardiac muscle tissue development	10	1	246	0.3985
GO:0055010	IPI00708070	Biological Process	Ventricular cardiac muscle tissue morphogenesis	12	1	246	0.3985
GO:0006901	IPI00866975	Biological Process	Vesicle coating	7	1	246	0.5164
GO:0006906	IPI00843305	Biological Process	Vesicle fusion	6	1	246	0.4407
GO:0016032	IPI00713313	Biological Process	Viral process	5	1	246	0.7096
GO:0007632	IPI00685510	Biological Process	Visual behavior	5	1	246	0.3532
GO:0008542	IPI00685510	Biological Process	Visual learning	8	1	246	0.3044
GO:0007601	IPI00707857	Biological Process	Visual perception	9	1	246	0.3985

GO:0051180	IPI00714656	Biological Process	Vitamin transport	5	1	246	0.1351
GO:0016055	IPI00709876	Biological Process	Wnt signaling pathway	8	1	246	0.8380
GO:0006805	IPI00707815	Biological Process	Xenobiotic metabolic process	5	1	246	0.2520
GO:0005575	IPI00685147;IPI00685510;IPI00685617;IPI00685653;IPI00685678;IPI00685695;IPI00685769;IPI00685804;IPI00686595;IPI00686872;IPI00687375;IPI00687395;IPI00687478;IPI00687578;IPI00687634;IPI00687662;IPI00687839;IPI00687906;IPI00688337;IPI00688400;IPI00688446;IPI00688474;IPI00688704;IPI00688727;IPI00688878;IPI00688977;IPI00688980;IPI00689165;IPI00689226;IPI00689365;IPI00689379;IPI00689556;IPI00689565;IPI00689629;IPI00689641;IPI00690309;IPI00690581;IPI00690620;IPI00690789;IPI00691086;IPI00691093;IPI00691649;IPI00691826;IPI00692186;IPI00692668;IPI00692739;IPI00692893;IPI006	Cellular Component	Cellular_component	2	222	246	0.0391

GO:0005623	IPI00685147;IPI00685510;IPI00685617;IPI00685678;IPI00685695;IPI00685769;IPI00685804;IPI00686595;IPI00686872;IPI00687395;IPI00687478;IPI00687578;IPI00687634;IPI00687662;IPI00687839;IPI00687906;IPI00688337;IPI00688400;IPI00688446;IPI00688474;IPI00688704;IPI00688727;IPI00688878;IPI00688977;IPI00688980;IPI00689165;IPI00689226;IPI00689365;IPI00689379;IPI00689556;IPI00689565;IPI00689641;IPI00690309;IPI00690581;IPI00690620;IPI00690789;IPI00691086;IPI00691093;IPI00691649;IPI00691826;IPI00692186;IPI00692668;IPI00692963;IPI00693452;IPI00693579;IPI00693824;IPI00693842;IPI006	Cellular Component	Cell	3	193	246	0.8418

GO:0044464	IPI00685147;IPI00685510;IPI00685617;IPI00685678;IPI00685695;IPI00685769;IPI00685804;IPI00686595;IPI00686872;IPI00687395;IPI00687478;IPI00687578;IPI00687634;IPI00687662;IPI00687839;IPI00687906;IPI00688337;IPI00688400;IPI00688446;IPI00688474;IPI00688704;IPI00688727;IPI00688878;IPI00688977;IPI00688980;IPI00689165;IPI00689226;IPI00689365;IPI00689379;IPI00689556;IPI00689565;IPI00689641;IPI00690309;IPI00690581;IPI00690620;IPI00690789;IPI00691086;IPI00691093;IPI00691649;IPI00691826;IPI00692186;IPI00692668;IPI00692963;IPI00693452;IPI00693579;IPI00693824;IPI00693842;IPI006	Cellular Component	Cell part	4	193	246	0.8418

GO:0005622	IPI00685147;IPI00685510;IPI00685678;IPI00685695;IPI00685769;IPI00685804;IPI00686595;IPI00686872;IPI00687395;IPI00687478;IPI00687578;IPI00687634;IPI00687662;IPI00687839;IPI00687906;IPI00688337;IPI00688400;IPI00688446;IPI00688474;IPI00688704;IPI00688727;IPI00688878;IPI00688977;IPI00688980;IPI00689226;IPI00689365;IPI00689379;IPI00689556;IPI00689565;IPI00689641;IPI00690309;IPI00690581;IPI00690620;IPI00690789;IPI00691093;IPI00691649;IPI00691826;IPI00692186;IPI00692668;IPI00692963;IPI00693579;IPI00693824;IPI00693842;IPI00694038;IPI00694094;IPI00694198;IPI00694430;IPI006	Cellular Component	Intracellular	5	177	246	0.9813

GO:0044424	IPI00685147;IPI00685510;IPI00685678;IPI00685695;IPI00685769;IPI00685804;IPI00686595;IPI00686872;IPI00687395;IPI00687478;IPI00687578;IPI00687634;IPI00687662;IPI00687839;IPI00687906;IPI00688337;IPI00688400;IPI00688446;IPI00688474;IPI00688704;IPI00688727;IPI00688878;IPI00688977;IPI00689226;IPI00689365;IPI00689379;IPI00689556;IPI00689565;IPI00689641;IPI00690309;IPI00690581;IPI00690620;IPI00690789;IPI00691093;IPI00691649;IPI00692186;IPI00692668;IPI00692963;IPI00693579;IPI00693824;IPI00693842;IPI00694038;IPI00694094;IPI00694198;IPI00694430;IPI00694444;IPI00694565;IPI006	Cellular Component	Intracellular part	6	172	246	0.9847

GO:0043229	IPI00685147;IPI00685510;IPI00685695;IPI00685769;IPI00685804;IPI00686595;IPI00686872;IPI00687395;IPI00687578;IPI00687634;IPI00687662;IPI00687839;IPI00687906;IPI00688337;IPI00688446;IPI00688474;IPI00688704;IPI00688727;IPI00688878;IPI00688977;IPI00689226;IPI00689365;IPI00689379;IPI00689556;IPI00689565;IPI00689641;IPI00690309;IPI00690581;IPI00690620;IPI00691093;IPI00691649;IPI00692186;IPI00692668;IPI00692963;IPI00693824;IPI00693842;IPI00694038;IPI00694094;IPI00694198;IPI00694430;IPI00694444;IPI00694565;IPI00694581;IPI00694688;IPI00695548;IPI00695852;IPI00695952;IPI006	Cellular Component	Intracellular organelle	7	151	246	0.8739

GO:0043226	IPI00685147;IPI00685510;IPI00685695;IPI00685769;IPI00685804;IPI00686595;IPI00686872;IPI00687395;IPI00687578;IPI00687634;IPI00687662;IPI00687839;IPI00687906;IPI00688337;IPI00688446;IPI00688474;IPI00688704;IPI00688727;IPI00688878;IPI00688977;IPI00689226;IPI00689365;IPI00689379;IPI00689556;IPI00689565;IPI00689641;IPI00690309;IPI00690581;IPI00690620;IPI00691093;IPI00691649;IPI00692186;IPI00692668;IPI00692963;IPI00693824;IPI00693842;IPI00694038;IPI00694094;IPI00694198;IPI00694430;IPI00694444;IPI00694565;IPI00694581;IPI00694688;IPI00695548;IPI00695852;IPI00695952;IPI006	Cellular Component	Organelle	3	151	246	0.8779

GO:0005737	IPI00685147;IPI00685510;IPI00685678;IPI00685695;IPI00685769;IPI00685804;IPI00686872;IPI00687478;IPI00687578;IPI00687634;IPI00687662;IPI00687839;IPI00687906;IPI00688400;IPI00688474;IPI00688727;IPI00688878;IPI00688977;IPI00689365;IPI00689379;IPI00689556;IPI00689565;IPI00690309;IPI00690581;IPI00690620;IPI00690789;IPI00691093;IPI00691649;IPI00692186;IPI00692668;IPI00692963;IPI00693579;IPI00693824;IPI00694038;IPI00694094;IPI00694198;IPI00694430;IPI00694444;IPI00694565;IPI00694581;IPI00694688;IPI00695852;IPI00695952;IPI00696116;IPI00697164;IPI00697194;IPI00697648;IPI006	Cellular Component	Cytoplasm	7	150	246	0.8412

GO:0044444	IPI00685147;IPI00685510;IPI00685695;IPI00685769;IPI00685804;IPI00686872;IPI00687578;IPI00687634;IPI00687662;IPI00687839;IPI00687906;IPI00688400;IPI00688474;IPI00688727;IPI00688878;IPI00688977;IPI00689365;IPI00689379;IPI00689556;IPI00689565;IPI00690309;IPI00690581;IPI00690620;IPI00690789;IPI00691093;IPI00691649;IPI00692186;IPI00692963;IPI00693824;IPI00694038;IPI00694094;IPI00694198;IPI00694430;IPI00694444;IPI00694565;IPI00694581;IPI00694688;IPI00695852;IPI00695952;IPI00696116;IPI00697194;IPI00697648;IPI00697917;IPI00698059;IPI00698077;IPI00698823;IPI00698916;IPI006	Cellular Component	Cytoplasmic part	8	120	246	0.1753

GO:0043231	IPI00685147;IPI00685510;IPI00685695;IPI00685769;IPI00685804;IPI00686595;IPI00686872;IPI00687395;IPI00687634;IPI00687839;IPI00687906;IPI00688337;IPI00688446;IPI00688474;IPI00688704;IPI00688878;IPI00688977;IPI00689226;IPI00689365;IPI00689556;IPI00689641;IPI00690309;IPI00690581;IPI00690620;IPI00691649;IPI00692668;IPI00692963;IPI00693824;IPI00693842;IPI00694038;IPI00694094;IPI00694198;IPI00694430;IPI00694565;IPI00694581;IPI00695548;IPI00695852;IPI00695952;IPI00696116;IPI00696748;IPI00697164;IPI00697194;IPI00698059;IPI00698213;IPI00698339;IPI00698823;IPI00698916;IPI006	Cellular Component	Intracellular membrane-bounded organelle	8	119	246	0.9972

GO:0043227	IPI00685147;IPI00685510;IPI00685695;IPI00685769;IPI00685804;IPI00686595;IPI00686872;IPI00687395;IPI00687634;IPI00687839;IPI00687906;IPI00688337;IPI00688446;IPI00688474;IPI00688704;IPI00688878;IPI00688977;IPI00689226;IPI00689365;IPI00689556;IPI00689641;IPI00690309;IPI00690581;IPI00690620;IPI00691649;IPI00692668;IPI00692963;IPI00693824;IPI00693842;IPI00694038;IPI00694094;IPI00694198;IPI00694430;IPI00694565;IPI00694581;IPI00695548;IPI00695852;IPI00695952;IPI00696116;IPI00696748;IPI00697164;IPI00697194;IPI00698059;IPI00698213;IPI00698339;IPI00698823;IPI00698916;IPI006	Cellular Component	Membrane-bounded organelle	4	119	246	0.9975

GO:0016020	IPI00685147;IPI00685510;IPI00685617;IPI00685769;IPI00685804;IPI00686872;IPI00687661;IPI00687839;IPI00688474;IPI00688878;IPI00689165;IPI00689226;IPI00689556;IPI00689629;IPI00690309;IPI00690581;IPI00690620;IPI00691086;IPI00691093;IPI00691649;IPI00691826;IPI00692186;IPI00692739;IPI00692893;IPI00692963;IPI00693452;IPI00693824;IPI00694094;IPI00694198;IPI00694581;IPI00694831;IPI00695042;IPI00696116;IPI00697349;IPI00697413;IPI00697595;IPI00698059;IPI00698823;IPI00698916;IPI00699039;IPI00699226;IPI00699372;IPI00699817;IPI00701747;IPI00702768;IPI00703084;IPI00703813;IPI007	Cellular Component	Membrane	3	110	246	0.0010

GO:0044422	IPI00685147;IPI00685510;IPI00685769;IPI00685804;IPI00686595;IPI00686872;IPI00687395;IPI00687578;IPI00687634;IPI00687662;IPI00687839;IPI00688337;IPI00688446;IPI00688474;IPI00688704;IPI00688878;IPI00688977;IPI00689226;IPI00689379;IPI00689556;IPI00689565;IPI00689641;IPI00690309;IPI00690581;IPI00690620;IPI00691093;IPI00692668;IPI00692963;IPI00693824;IPI00694198;IPI00694565;IPI00694581;IPI00694688;IPI00696116;IPI00696748;IPI00697194;IPI00697648;IPI00697917;IPI00698059;IPI00698077;IPI00698213;IPI00698339;IPI00698823;IPI00698916;IPI00699372;IPI00699470;IPI00699817;IPI007	Cellular Component	Organelle part	4	92	246	0.4964

GO:0044446	IPI00685147;IPI00685510;IPI00685769;IPI00685804;IPI00686595;IPI00686872;IPI00687395;IPI00687578;IPI00687634;IPI00687662;IPI00687839;IPI00688337;IPI00688446;IPI00688474;IPI00688704;IPI00688878;IPI00688977;IPI00689226;IPI00689379;IPI00689556;IPI00689565;IPI00689641;IPI00690309;IPI00690581;IPI00690620;IPI00691093;IPI00692668;IPI00692963;IPI00693824;IPI00694198;IPI00694565;IPI00694581;IPI00694688;IPI00696116;IPI00696748;IPI00697194;IPI00697648;IPI00697917;IPI00698059;IPI00698077;IPI00698213;IPI00698339;IPI00698823;IPI00698916;IPI00699372;IPI00699470;IPI00699817;IPI007	Cellular Component	Intracellular organelle part	8	91	246	0.4592

GO:0044425	IPI00685147;IPI00685510;IPI00685617;IPI00685769;IPI00685804;IPI00686872;IPI00687839;IPI00688878;IPI00689165;IPI00689226;IPI00689556;IPI00689629;IPI00690581;IPI00690620;IPI00691086;IPI00691649;IPI00692186;IPI00692739;IPI00692893;IPI00692963;IPI00693452;IPI00694094;IPI00694831;IPI00696116;IPI00697349;IPI00697413;IPI00697595;IPI00698059;IPI00698823;IPI00698916;IPI00699039;IPI00699226;IPI00699372;IPI00699817;IPI00701747;IPI00702768;IPI00703084;IPI00703813;IPI00703954;IPI00705159;IPI00705237;IPI00705378;IPI00706037;IPI00706314;IPI00706879;IPI00708037;IPI00708085;IPI007	Cellular Component	Membrane part	4	88	246	3.82E-07

GO:0031224	IPI00685147;IPI00685510;IPI00685617;IPI00685804;IPI00686872;IPI00687839;IPI00688878;IPI00689165;IPI00689226;IPI00689556;IPI00689629;IPI00690581;IPI00690620;IPI00691086;IPI00691649;IPI00692186;IPI00692739;IPI00692893;IPI00693452;IPI00694094;IPI00694831;IPI00696116;IPI00697349;IPI00697413;IPI00697595;IPI00698059;IPI00698823;IPI00699039;IPI00699226;IPI00699372;IPI00699817;IPI00701747;IPI00702768;IPI00703084;IPI00703813;IPI00703954;IPI00705159;IPI00705378;IPI00706314;IPI00706879;IPI00708085;IPI00708162;IPI00708535;IPI00708597;IPI00708824;IPI00709542;IPI00709876;IPI007	Cellular Component	Intrinsic component of membrane	5	77	246	4.63E-12

GO:0016021	IPI00685147;IPI00685510;IPI00685617;IPI00685804;IPI00686872;IPI00687839;IPI00688878;IPI00689165;IPI00689226;IPI00689556;IPI00689629;IPI00690581;IPI00690620;IPI00691086;IPI00691649;IPI00692186;IPI00692739;IPI00692893;IPI00693452;IPI00694094;IPI00694831;IPI00696116;IPI00697349;IPI00697413;IPI00697595;IPI00698059;IPI00698823;IPI00699039;IPI00699226;IPI00699372;IPI00701747;IPI00703084;IPI00703813;IPI00703954;IPI00705159;IPI00705378;IPI00706314;IPI00706879;IPI00708085;IPI00708162;IPI00708535;IPI00708597;IPI00708824;IPI00709542;IPI00709876;IPI00709888;IPI00710204;IPI007	Cellular Component	Integral component of membrane	6	75	246	6.88E-12

GO:0032991	IPI00685510;IPI00685653;IPI00685769;IPI00686595;IPI00686872;IPI00687395;IPI00687634;IPI00687662;IPI00688337;IPI00688446;IPI00688727;IPI00689226;IPI00689379;IPI00689565;IPI00689641;IPI00690581;IPI00691093;IPI00692963;IPI00693452;IPI00694444;IPI00694565;IPI00694688;IPI00696116;IPI00696748;IPI00697595;IPI00697648;IPI00697917;IPI00698077;IPI00698213;IPI00698339;IPI00699372;IPI00699470;IPI00699827;IPI00700471;IPI00700673;IPI00701747;IPI00702948;IPI00703954;IPI00704041;IPI00704902;IPI00705104;IPI00705237;IPI00705739;IPI00706142;IPI00707386;IPI00707537;IPI00708037;IPI007	Cellular Component	Macromolecular complex	3	70	246	0.6696

GO:0043232	IPI00685510;IPI00687578;IPI00687634;IPI00687662;IPI00688704;IPI00688727;IPI00689379;IPI00689565;IPI00689641;IPI00690620;IPI00691093;IPI00692186;IPI00692668;IPI00694444;IPI00694565;IPI00694688;IPI00697648;IPI00697917;IPI00698077;IPI00698213;IPI00698339;IPI00699827;IPI00700471;IPI00701747;IPI00702948;IPI00704902;IPI00705104;IPI00705237;IPI00705739;IPI00706037;IPI00707386;IPI00707537;IPI00708070;IPI00708210;IPI00708767;IPI00709409;IPI00710274;IPI00710834;IPI00710859;IPI00712288;IPI00712384;IPI00712399;IPI00712858;IPI00713474;IPI00714898;IPI00715868;IPI00716830;IPI007	Cellular Component	Intracellular non-membrane-bounded organelle	8	56	246	0.2341

GO:0043228	IPI00685510;IPI00687578;IPI00687634;IPI00687662;IPI00688704;IPI00688727;IPI00689379;IPI00689565;IPI00689641;IPI00690620;IPI00691093;IPI00692186;IPI00692668;IPI00694444;IPI00694565;IPI00694688;IPI00697648;IPI00697917;IPI00698077;IPI00698213;IPI00698339;IPI00699827;IPI00700471;IPI00701747;IPI00702948;IPI00704902;IPI00705104;IPI00705237;IPI00705739;IPI00706037;IPI00707386;IPI00707537;IPI00708070;IPI00708210;IPI00708767;IPI00709409;IPI00710274;IPI00710834;IPI00710859;IPI00712288;IPI00712384;IPI00712399;IPI00712858;IPI00713474;IPI00714898;IPI00715868;IPI00716830;IPI007	Cellular Component	Non-membrane-bounded organelle	4	56	246	0.2341

GO:0005634	IPI00686595;IPI00687395;IPI00687634;IPI00688337;IPI00688446;IPI00688704;IPI00689226;IPI00689641;IPI00690309;IPI00692668;IPI00693842;IPI00694038;IPI00694565;IPI00695548;IPI00696748;IPI00697164;IPI00698213;IPI00698339;IPI00698823;IPI00699372;IPI00700228;IPI00701747;IPI00702948;IPI00703022;IPI00704332;IPI00704902;IPI00705104;IPI00706142;IPI00707776;IPI00707815;IPI00710274;IPI00712263;IPI00712399;IPI00714269;IPI00714656;IPI00714898;IPI00715059;IPI00727591;IPI00734257;IPI00825680;IPI00845178;IPI00866975;IPI00867017;IPI00867311;IPI00906031;IPI01003286;IPI01017508	Cellular Component	Nucleus	9	47	246	0.9999

GO:0031090	IPI00685147;IPI00685769;IPI00685804;IPI00686872;IPI00687839;IPI00688474;IPI00688878;IPI00689226;IPI00689556;IPI00690581;IPI00690620;IPI00692963;IPI00693824;IPI00694198;IPI00694581;IPI00696116;IPI00698059;IPI00698916;IPI00699372;IPI00699817;IPI00703084;IPI00703954;IPI00705378;IPI00706314;IPI00708162;IPI00708597;IPI00709876;IPI00709888;IPI00711457;IPI00712858;IPI00713460;IPI00713474;IPI00714325;IPI00715640;IPI00716830;IPI00717110;IPI00730219;IPI00843305;IPI00847122;IPI00866975;IPI00867237;IPI00867311;IPI00903861;IPI00906031;IPI01003286	Cellular Component	Organelle membrane	4	45	246	0.1036

GO:0071944	IPI00685510;IPI00685617;IPI00685769;IPI00689165;IPI00689556;IPI00691086;IPI00691093;IPI00691826;IPI00692186;IPI00693452;IPI00693824;IPI00694198;IPI00696116;IPI00697595;IPI00698823;IPI00699372;IPI00702768;IPI00703813;IPI00705159;IPI00705237;IPI00705565;IPI00707815;IPI00708824;IPI00709876;IPI00710274;IPI00710834;IPI00710920;IPI00712288;IPI00713313;IPI00713597;IPI00713979;IPI00714656;IPI00714660;IPI00715059;IPI00716220;IPI00716830;IPI00717930;IPI00718152;IPI00843305;IPI00845178;IPI00866975;IPI00876805;IPI00924039	Cellular Component	Cell periphery	5	43	246	0.2546

GO:0005886	IPI00685510;IPI00685617;IPI00685769;IPI00689165;IPI00689556;IPI00691086;IPI00691093;IPI00691826;IPI00692186;IPI00693452;IPI00693824;IPI00694198;IPI00696116;IPI00697595;IPI00698823;IPI00699372;IPI00702768;IPI00703813;IPI00705159;IPI00705237;IPI00705565;IPI00707815;IPI00708824;IPI00709876;IPI00710274;IPI00710834;IPI00710920;IPI00712288;IPI00713313;IPI00713597;IPI00713979;IPI00714656;IPI00714660;IPI00715059;IPI00716220;IPI00716830;IPI00717930;IPI00718152;IPI00843305;IPI00845178;IPI00866975;IPI00876805;IPI00924039	Cellular Component	Plasma membrane	5	43	246	0.1287

GO:0043234	IPI00685510;IPI00685769;IPI00686872;IPI00688446;IPI00689226;IPI00689379;IPI00689641;IPI00691093;IPI00692963;IPI00693452;IPI00696116;IPI00696748;IPI00697595;IPI00697648;IPI00697917;IPI00698213;IPI00698339;IPI00699372;IPI00699470;IPI00700471;IPI00701747;IPI00703954;IPI00704041;IPI00704902;IPI00705104;IPI00705237;IPI00706142;IPI00708037;IPI00708070;IPI00709876;IPI00712399;IPI00712858;IPI00714325;IPI00714660;IPI00716830;IPI00866975;IPI00876805;IPI00903861;IPI00906031;IPI01017508;IPI01017588;IPI01028455	Cellular Component	Protein complex	4	42	246	0.9896

GO:0005739	IPI00686872;IPI00687839;IPI00687906;IPI00688474;IPI00688977;IPI00689365;IPI00690620;IPI00691649;IPI00694094;IPI00694430;IPI00694581;IPI00695952;IPI00697194;IPI00698059;IPI00702251;IPI00703084;IPI00705378;IPI00706314;IPI00706634;IPI00707455;IPI00709124;IPI00711457;IPI00712858;IPI00714098;IPI00715066;IPI00717758;IPI00718152;IPI00727591;IPI00730219;IPI00760457;IPI00867017;IPI00871114;IPI00904814;IPI00906031;IPI00908033	Cellular Component	Mitochondrion	9	35	246	0.8892
GO:0012505	IPI00685147;IPI00685769;IPI00685804;IPI00687839;IPI00689226;IPI00690309;IPI00690581;IPI00692963;IPI00696116;IPI00698823;IPI00698916;IPI00699817;IPI00701747;IPI00703954;IPI00706037;IPI00708162;IPI00708597;IPI00709876;IPI00709888;IPI00713460;IPI00713474;IPI00714325;IPI00715640;IPI00716830;IPI00717110;IPI00866975;IPI00867237;IPI00903861;IPI01003286	Cellular Component	Endomembrane system	5	29	246	0.0407

GO:0030529	IPI00686595;IPI00687395;IPI00687634;IPI00687662;IPI00688337;IPI00688727;IPI00689565;IPI00694444;IPI00694565;IPI00694688;IPI00698077;IPI00699827;IPI00702948;IPI00704902;IPI00705739;IPI00707386;IPI00707537;IPI00708210;IPI00709409;IPI00710859;IPI00712384;IPI00715868;IPI00716860;IPI00717048;IPI00718379;IPI00904814	Cellular Component	Ribonucleoprotein complex	7	26	246	0.0343
GO:0005576	IPI00685653;IPI00687375;IPI00687578;IPI00689165;IPI00690581;IPI00694597;IPI00700622;IPI00700673;IPI00707685;IPI00707857;IPI00708244;IPI00708946;IPI00710664;IPI00712524;IPI00715174;IPI00716157;IPI00716220;IPI00717764;IPI00717930;IPI00731432;IPI00826022;IPI00867237;IPI00876781;IPI01028455	Cellular Component	Extracellular region	3	24	246	0.2222

GO:0005856	IPI00685510;IPI00687578;IPI00689379;IPI00691093;IPI00692186;IPI00692668;IPI00697648;IPI00697917;IPI00700471;IPI00705237;IPI00706037;IPI00708070;IPI00708767;IPI00710274;IPI00710834;IPI00712288;IPI00712858;IPI00713474;IPI00714898;IPI00716830;IPI00904814;IPI01000210	Cellular Component	Cytoskeleton	9	22	246	0.9132
GO:0044459	IPI00685617;IPI00685769;IPI00689165;IPI00689556;IPI00691086;IPI00692186;IPI00693452;IPI00697595;IPI00698823;IPI00703813;IPI00705159;IPI00705237;IPI00709876;IPI00710834;IPI00710920;IPI00714656;IPI00714660;IPI00715059;IPI00843305;IPI00866975;IPI00876805;IPI00924039	Cellular Component	Plasma membrane part	6	22	246	0.0530
GO:0005840	IPI00687634;IPI00687662;IPI00688727;IPI00689565;IPI00694444;IPI00694565;IPI00694688;IPI00698077;IPI00699827;IPI00702948;IPI00705739;IPI00707386;IPI00707537;IPI00708210;IPI00709409;IPI00710859;IPI00712384;IPI00715868;IPI00716860;IPI00717048;IPI00718379;IPI00904814	Cellular Component	Ribosome	9	22	246	2.74E-06

GO:0005829	IPI00687662;IPI00688400;IPI00690309;IPI00690789;IPI00694038;IPI00694565;IPI00694688;IPI00698077;IPI00698916;IPI00702948;IPI00703954;IPI00705739;IPI00707386;IPI00707776;IPI00710859;IPI00713474;IPI00714621;IPI00718379;IPI00727591;IPI00847122	Cellular Component	Cytosol	9	20	246	0.6826
GO:0005783	IPI00685147;IPI00685695;IPI00685804;IPI00687839;IPI00690581;IPI00692963;IPI00698823;IPI00698916;IPI00699817;IPI00703925;IPI00703954;IPI00708597;IPI00709876;IPI00714325;IPI00715640;IPI00716830;IPI00717110;IPI00717764;IPI00838420;IPI00903861	Cellular Component	Endoplasmic reticulum	9	20	246	0.2701
GO:0044428	IPI00686595;IPI00687395;IPI00687634;IPI00688337;IPI00688446;IPI00688704;IPI00689226;IPI00690309;IPI00694565;IPI00696748;IPI00698213;IPI00698339;IPI00698823;IPI00701747;IPI00705104;IPI00710274;IPI00712399;IPI00727591;IPI00906031;IPI01017508	Cellular Component	Nuclear part	10	20	246	0.9910

GO:0031982	IPI00685510;IPI00685769;IPI00692963;IPI00693824;IPI00694198;IPI00696116;IPI00698823;IPI00699372;IPI00699470;IPI00700622;IPI00706037;IPI00707685;IPI00709876;IPI00710664;IPI00714656;IPI00714660;IPI00716830;IPI00718152;IPI00843305;IPI00866975	Cellular Component	Vesicle	4	20	246	0.1848
GO:0070013	IPI00687634;IPI00688446;IPI00688704;IPI00688977;IPI00689226;IPI00690309;IPI00690620;IPI00694565;IPI00697194;IPI00698213;IPI00698339;IPI00705104;IPI00710274;IPI00712399;IPI00712858;IPI00727591;IPI00867017;IPI00906031;IPI01017508	Cellular Component	Intracellular organelle lumen	9	19	246	0.9815
GO:0031974	IPI00687634;IPI00688446;IPI00688704;IPI00688977;IPI00689226;IPI00690309;IPI00690620;IPI00694565;IPI00697194;IPI00698213;IPI00698339;IPI00705104;IPI00710274;IPI00712399;IPI00712858;IPI00727591;IPI00867017;IPI00906031;IPI01017508	Cellular Component	Membrane-enclosed lumen	3	19	246	0.9904

GO:0043233	IPI00687634;IPI00688446;IPI00688704;IPI00688977;IPI00689226;IPI00690309;IPI00690620;IPI00694565;IPI00697194;IPI00698213;IPI00698339;IPI00705104;IPI00710274;IPI00712399;IPI00712858;IPI00727591;IPI00867017;IPI00906031;IPI01017508	Cellular Component	Organelle lumen	4	19	246	0.9821
GO:0031410	IPI00685510;IPI00685769;IPI00692963;IPI00693824;IPI00694198;IPI00696116;IPI00699372;IPI00699470;IPI00700622;IPI00706037;IPI00707685;IPI00709876;IPI00710664;IPI00714656;IPI00714660;IPI00718152;IPI00843305;IPI00866975	Cellular Component	Cytoplasmic vesicle	9	18	246	0.2310
GO:0016023	IPI00685769;IPI00692963;IPI00693824;IPI00694198;IPI00696116;IPI00699372;IPI00699470;IPI00700622;IPI00706037;IPI00707685;IPI00709876;IPI00710664;IPI00714656;IPI00714660;IPI00718152;IPI00843305;IPI00866975	Cellular Component	Cytoplasmic membrane-bounded vesicle	10	17	246	0.2578

GO:0044421	IPI00685653;IPI00687375;IPI00687578;IPI00690581;IPI00700622;IPI00700673;IPI00707685;IPI00707857;IPI00708244;IPI00712524;IPI00715174;IPI00716157;IPI00717764;IPI00731432;IPI00826022;IPI00867237;IPI01028455	Cellular Component	Extracellular region part	4	17	246	0.1622
GO:0031988	IPI00685769;IPI00692963;IPI00693824;IPI00694198;IPI00696116;IPI00699372;IPI00699470;IPI00700622;IPI00706037;IPI00707685;IPI00709876;IPI00710664;IPI00714656;IPI00714660;IPI00718152;IPI00843305;IPI00866975	Cellular Component	Membrane-bounded vesicle	5	17	246	0.3053
GO:0031975	IPI00686872;IPI00687839;IPI00688474;IPI00689226;IPI00690620;IPI00694581;IPI00698059;IPI00698823;IPI00701747;IPI00703084;IPI00705378;IPI00706314;IPI00711457;IPI00712858;IPI00730219;IPI00906031	Cellular Component	Envelope	5	16	246	0.8843
GO:0005794	IPI00685510;IPI00692963;IPI00698823;IPI00699372;IPI00699470;IPI00703925;IPI00705104;IPI00708162;IPI00709876;IPI00709888;IPI00713460;IPI00713474;IPI00829520;IPI00867237;IPI00867311;IPI01003286	Cellular Component	Golgi apparatus	9	16	246	0.5280

GO:0044429	IPI00686872;IPI00687839;IPI00688474;IPI00688977;IPI00690620;IPI00694581;IPI00697194;IPI00698059;IPI00703084;IPI00705378;IPI00706314;IPI00711457;IPI00712858;IPI00730219;IPI00867017;IPI00906031	Cellular Component	Mitochondrial part	10	16	246	0.8120
GO:0031967	IPI00686872;IPI00687839;IPI00688474;IPI00689226;IPI00690620;IPI00694581;IPI00698059;IPI00698823;IPI00701747;IPI00703084;IPI00705378;IPI00706314;IPI00711457;IPI00712858;IPI00730219;IPI00906031	Cellular Component	Organelle envelope	9	16	246	0.8811
GO:0044430	IPI00685510;IPI00687578;IPI00689379;IPI00691093;IPI00692668;IPI00697648;IPI00697917;IPI00700471;IPI00705237;IPI00708070;IPI00710274;IPI00712288;IPI00712858;IPI00713474;IPI00716830	Cellular Component	Cytoskeletal part	10	15	246	0.8642
GO:0005789	IPI00685147;IPI00685804;IPI00687839;IPI00690581;IPI00692963;IPI00698916;IPI00699817;IPI00703954;IPI00708597;IPI00714325;IPI00715640;IPI00716830;IPI00717110;IPI00903861	Cellular Component	Endoplasmic reticulum membrane	11	14	246	0.0610

GO:0044432	IPI00685147;IPI00685804;IPI00687839;IPI00690581;IPI00692963;IPI00698916;IPI00699817;IPI00703954;IPI00708597;IPI00714325;IPI00715640;IPI00716830;IPI00717110;IPI00903861	Cellular Component	Endoplasmic reticulum part	10	14	246	0.1621
GO:0031981	IPI00687634;IPI00688446;IPI00688704;IPI00689226;IPI00690309;IPI00694565;IPI00698213;IPI00698339;IPI00705104;IPI00710274;IPI00712399;IPI00727591;IPI00906031;IPI01017508	Cellular Component	Nuclear lumen	11	14	246	0.9576
GO:0042175	IPI00685147;IPI00685804;IPI00687839;IPI00690581;IPI00692963;IPI00698916;IPI00699817;IPI00703954;IPI00708597;IPI00714325;IPI00715640;IPI00716830;IPI00717110;IPI00903861	Cellular Component	Nuclear outer membrane-endoplasmic reticulum membrane network	6	14	246	0.0750
GO:0005615	IPI00685653;IPI00690581;IPI00700622;IPI00700673;IPI00707685;IPI00707857;IPI00708244;IPI00715174;IPI00716157;IPI00717764;IPI00731432;IPI00867237;IPI01028455	Cellular Component	Extracellular space	5	13	246	0.1653

GO:0005740	IPI00686872;IPI00687839;IPI00688474;IPI00690620;IPI00694581;IPI00698059;IPI00703084;IPI00705378;IPI00706314;IPI00711457;IPI00712858;IPI00730219;IPI00906031	Cellular Component	Mitochondrial envelope	11	13	246	0.7412
GO:0048471	IPI00685510;IPI00689379;IPI00692963;IPI00696116;IPI00698823;IPI00702948;IPI00703925;IPI00705237;IPI00709876;IPI00710274;IPI00714660;IPI00716830;IPI00866975	Cellular Component	Perinuclear region of cytoplasm	9	13	246	0.0298
GO:0005768	IPI00685769;IPI00689556;IPI00693824;IPI00694198;IPI00694581;IPI00698823;IPI00709876;IPI00710274;IPI00713474;IPI00718152;IPI00847122;IPI00867311	Cellular Component	Endosome	9	12	246	0.1090
GO:0031966	IPI00686872;IPI00688474;IPI00690620;IPI00694581;IPI00698059;IPI00703084;IPI00705378;IPI00706314;IPI00711457;IPI00712858;IPI00730219;IPI00906031	Cellular Component	Mitochondrial membrane	12	12	246	0.7854
GO:0042995	IPI00685510;IPI00693824;IPI00696116;IPI00698916;IPI00703813;IPI00708767;IPI00710274;IPI00714269;IPI00843305;IPI00876805;IPI00924039	Cellular Component	Cell projection	5	11	246	0.7722

GO:0044391	IPI00687662;IPI00689565;IPI00694565;IPI00694688;IPI00698077;IPI00702948;IPI00705739;IPI00707386;IPI00710859;IPI00712384;IPI00718379	Cellular Component	Ribosomal subunit	10	11	246	0.0014
GO:0044431	IPI00692963;IPI00699470;IPI00703925;IPI00708162;IPI00709876;IPI00709888;IPI00713460;IPI00713474;IPI00867237;IPI01003286	Cellular Component	Golgi apparatus part	10	10	246	0.1126
GO:0030054	IPI00696116;IPI00705237;IPI00708070;IPI00710274;IPI00713313;IPI00713979;IPI00716830;IPI00734257;IPI00876805	Cellular Component	Cell junction	3	9	246	0.6559
GO:0044445	IPI00687662;IPI00694565;IPI00694688;IPI00698077;IPI00702948;IPI00705739;IPI00707386;IPI00710859;IPI00718379	Cellular Component	Cytosolic part	10	9	246	0.0811
GO:0022626	IPI00687662;IPI00694565;IPI00694688;IPI00698077;IPI00702948;IPI00705739;IPI00707386;IPI00710859;IPI00718379	Cellular Component	Cytosolic ribosome	11	9	246	0.0045
GO:0015630	IPI00685510;IPI00687578;IPI00689379;IPI00692668;IPI00697917;IPI00705237;IPI00712858;IPI00713474;IPI00716830	Cellular Component	Microtubule cytoskeleton	10	9	246	0.9056
GO:0005743	IPI00688474;IPI00690620;IPI00694581;IPI00703084;IPI00705378;IPI00706314;IPI00711457;IPI00712858;IPI00730219	Cellular Component	Mitochondrial inner membrane	13	9	246	0.8394

GO:0019866	IPI00688474;IPI00690620;IPI00694581;IPI00703084;IPI00705378;IPI00706314;IPI00711457;IPI00712858;IPI00730219	Cellular Component	Organelle inner membrane	7	9	246	0.8632
GO:0016323	IPI00689556;IPI00691086;IPI00692186;IPI00705159;IPI00709876;IPI00710920;IPI00714656;IPI00714660	Cellular Component	Basolateral plasma membrane	6	8	246	0.0047
GO:0009986	IPI00685510;IPI00685769;IPI00688400;IPI00691093;IPI00697595;IPI00714660;IPI00715640;IPI00876805	Cellular Component	Cell surface	5	8	246	0.2618
GO:0044427	IPI00689641;IPI00698339;IPI00701747;IPI00705104;IPI00712399;IPI00727591;IPI00734257;IPI00906031	Cellular Component	Chromosomal part	10	8	246	0.3571
GO:0005694	IPI00689641;IPI00698339;IPI00701747;IPI00705104;IPI00712399;IPI00727591;IPI00734257;IPI00906031	Cellular Component	Chromosome	9	8	246	0.4664
GO:0044433	IPI00685769;IPI00692963;IPI00696116;IPI00699372;IPI00699470;IPI00709876;IPI00843305;IPI00866975	Cellular Component	Cytoplasmic vesicle part	10	8	246	0.0996
GO:0031012	IPI00687375;IPI00702768;IPI00707857;IPI00708244;IPI00712524;IPI00715174;IPI00731432;IPI00826022	Cellular Component	Extracellular matrix	3	8	246	0.3789
GO:0000139	IPI00692963;IPI00708162;IPI00709876;IPI00709888;IPI00713460;IPI00713474;IPI00867237;IPI01003286	Cellular Component	Golgi membrane	11	8	246	0.1510

GO:0005887	IPI00685617;IPI00692186;IPI00693452;IPI00697595;IPI00698823;IPI00709876;IPI00710834;IPI00714656	Cellular Component	Integral component of plasma membrane	7	8	246	0.0189
GO:0031300	IPI00687839;IPI00690581;IPI00699817;IPI00714325;IPI00715640;IPI00867237;IPI00903861;IPI01003286	Cellular Component	Intrinsic component of organelle membrane	6	8	246	0.0236
GO:0031226	IPI00685617;IPI00692186;IPI00693452;IPI00697595;IPI00698823;IPI00709876;IPI00710834;IPI00714656	Cellular Component	Intrinsic component of plasma membrane	7	8	246	0.0355
GO:0030659	IPI00685769;IPI00692963;IPI00696116;IPI00699372;IPI00709876;IPI00843305;IPI00866975	Cellular Component	Cytoplasmic vesicle membrane	11	7	246	0.1483
GO:0031301	IPI00687839;IPI00690581;IPI00714325;IPI00715640;IPI00867237;IPI00903861;IPI01003286	Cellular Component	Integral component of organelle membrane	7	7	246	0.0347
GO:0015934	IPI00689565;IPI00698077;IPI00705739;IPI00707386;IPI00710859;IPI00712384;IPI00718379	Cellular Component	large ribosomal subunit	11	7	246	0.0016
GO:0030141	IPI00685769;IPI00696116;IPI00700622;IPI00707685;IPI00709876;IPI00710664;IPI00714660	Cellular Component	Secretory granule	11	7	246	0.0428
GO:0012506	IPI00685769;IPI00692963;IPI00696116;IPI00699372;IPI00709876;IPI00843305;IPI00866975	Cellular Component	Vesicle membrane	5	7	246	0.2260
GO:0045177	IPI00685510;IPI00689556;IPI00691086;IPI00703813;IPI00709876;IPI00710920	Cellular Component	Apical part of cell	5	6	246	0.0987

GO:0000785	IPI00689641;IPI00698339;IPI00705104;IPI00712399;IPI00727591;IPI00734257	Cellular Component	Chromatin	11	6	246	0.1528
GO:0044420	IPI00702768;IPI00707857;IPI00708244;IPI00712524;IPI00731432;IPI00826022	Cellular Component	Extracellular matrix part	4	6	246	0.2172
GO:0031227	IPI00687839;IPI00690581;IPI00699817;IPI00714325;IPI00715640;IPI00903861	Cellular Component	Intrinsic component of endoplasmic reticulum membrane	12	6	246	0.0283
GO:0045121	IPI00685769;IPI00698823;IPI00709876;IPI00710834;IPI00715059;IPI00876805	Cellular Component	Membrane raft	5	6	246	0.0134
GO:0005730	IPI00687634;IPI00688704;IPI00694565;IPI00698213;IPI00710274;IPI00906031	Cellular Component	Nucleolus	11	6	246	0.8948
GO:0005578	IPI00687375;IPI00707857;IPI00708244;IPI00712524;IPI00731432;IPI00826022	Cellular Component	Proteinaceous extracellular matrix	5	6	246	0.4039
GO:0015629	IPI00691093;IPI00697648;IPI00710274;IPI00712288;IPI00904814	Cellular Component	Actin cytoskeleton	10	5	246	0.9375
GO:0016324	IPI00689556;IPI00691086;IPI00703813;IPI00709876;IPI00710920	Cellular Component	Apical plasma membrane	6	5	246	0.0614
GO:0005581	IPI00707857;IPI00708244;IPI00712524;IPI00731432;IPI00826022	Cellular Component	Collagen trimer	3	5	246	0.0614
GO:0022625	IPI00698077;IPI00705739;IPI00707386;IPI00710859;IPI00718379	Cellular Component	Cytosolic large ribosomal subunit	11	5	246	0.0105
GO:0044440	IPI00689556;IPI00693824;IPI00694198;IPI00847122;IPI00867311	Cellular Component	Endosomal part	10	5	246	0.2838
GO:0010008	IPI00689556;IPI00693824;IPI00694198;IPI00847122;IPI00867311	Cellular Component	Endosome membrane	11	5	246	0.2838

GO:0030176	IPI00687839;IPI00690581;IPI00714325;IPI00715640;IPI00903861	Cellular Component	Integral component of endoplasmic reticulum membrane	13	5	246	0.0480
GO:0005759	IPI00688977;IPI00690620;IPI00697194;IPI00712858;IPI00867017	Cellular Component	Mitochondrial matrix	11	5	246	0.7110
GO:0097458	IPI00685510;IPI00696116;IPI00698916;IPI00714269;IPI00876805	Cellular Component	Neuron part	5	5	246	0.7665
GO:0043005	IPI00685510;IPI00696116;IPI00698916;IPI00714269;IPI00876805	Cellular Component	Neuron projection	6	5	246	0.5747
GO:0005938	IPI00691093;IPI00692186;IPI00709876;IPI00710274	Cellular Component	Cell cortex	9	4	246	0.6776
GO:0031252	IPI00693824;IPI00710274;IPI00843305;IPI00924039	Cellular Component	Cell leading edge	5	4	246	0.7903
GO:0005911	IPI00705237;IPI00708070;IPI00713313;IPI00716830	Cellular Component	Cell-cell junction	4	4	246	0.6532
GO:0030135	IPI00692963;IPI00696116;IPI00706037;IPI00866975	Cellular Component	Coated vesicle	11	4	246	0.5587
GO:0022627	IPI00687662;IPI00694565;IPI00694688;IPI00702948	Cellular Component	Cytosolic small ribosomal subunit	12	4	246	0.1040
GO:1990104	IPI00689641;IPI00698339;IPI00705104;IPI00712399	Cellular Component	DNA bending complex	5	4	246	0.0134
GO:0030139	IPI00685769;IPI00694198;IPI00718152;IPI00866975	Cellular Component	Endocytic vesicle	11	4	246	0.1157
GO:0005583	IPI00707857;IPI00708244;IPI00731432;IPI00826022	Cellular Component	Fibrillar collagen trimer	6	4	246	0.0035
GO:0042470	IPI00693824;IPI00694198;IPI00699372;IPI00714656	Cellular Component	Melanosome	12	4	246	0.4678
GO:0005654	IPI00688446;IPI00690309;IPI00906031;IPI01017508	Cellular Component	Nucleoplasm	11	4	246	0.9924
GO:0000786	IPI00689641;IPI00698339;IPI00705104;IPI00712399	Cellular Component	Nucleosome	12	4	246	0.0134

GO:0048770	IPI00693824;IPI00694198;IPI00699372;IPI00714656	Cellular Component	Pigment granule	11	4	246	0.4678
GO:0032993	IPI00689641;IPI00698339;IPI00705104;IPI00712399	Cellular Component	Protein-DNA complex	4	4	246	0.0328
GO:0055037	IPI00713474;IPI00718152;IPI00847122;IPI00867311	Cellular Component	Recycling endosome	10	4	246	0.0466
GO:0042383	IPI00705159;IPI00709876;IPI00843305;IPI00876805	Cellular Component	Sarcolemma	6	4	246	0.1408
GO:0015935	IPI00687662;IPI00694565;IPI00694688;IPI00702948	Cellular Component	Small ribosomal subunit	11	4	246	0.1541
GO:0005819	IPI00685510;IPI00689379;IPI00705237;IPI00712858	Cellular Component	Spindle	9	4	246	0.4199
GO:0005802	IPI00696116;IPI00709888;IPI00714660;IPI00867311	Cellular Component	Trans-Golgi network	10	4	246	0.1157
GO:0005773	IPI00690309;IPI00698823;IPI00699372;IPI00908033	Cellular Component	Vacuole	9	4	246	0.7333
GO:0070161	IPI00705237;IPI00708070;IPI00710274	Cellular Component	Anchoring junction	4	3	246	0.8900
GO:0005901	IPI00685769;IPI00709876;IPI00715059	Cellular Component	Caveola	7	3	246	0.0465
GO:0044448	IPI00691093;IPI00692186;IPI00710274	Cellular Component	Cell cortex part	10	3	246	0.6043
GO:0044463	IPI00685510;IPI00876805;IPI00924039	Cellular Component	Cell projection part	6	3	246	0.8518
GO:0030136	IPI00696116;IPI00706037;IPI00866975	Cellular Component	Clathrin-coated vesicle	12	3	246	0.4850
GO:0048475	IPI00692963;IPI00708037;IPI00866975	Cellular Component	Coated membrane	4	3	246	0.5554
GO:0030662	IPI00692963;IPI00696116;IPI00866975	Cellular Component	Coated vesicle membrane	12	3	246	0.4097
GO:0000793	IPI00698339;IPI00712399;IPI00906031	Cellular Component	Condensed chromosome	10	3	246	0.2725
GO:0030863	IPI00691093;IPI00692186;IPI00710274	Cellular Component	Cortical cytoskeleton	11	3	246	0.3512
GO:0005769	IPI00693824;IPI00694198;IPI00710274	Cellular Component	Early endosome	10	3	246	0.5209
GO:0019898	IPI00689165;IPI00710274;IPI01000210	Cellular Component	Extrinsic component of membrane	5	3	246	0.5721

GO:0030027	IPI00710274;IPI00843305;IPI00924039	Cellular Component	Lamellipodium	6	3	246	0.5884
GO:0030117	IPI00692963;IPI00708037;IPI00866975	Cellular Component	Membrane coat	9	3	246	0.5554
GO:0005874	IPI00689379;IPI00697917;IPI00716830	Cellular Component	Microtubule	11	3	246	0.8116
GO:0005815	IPI00687578;IPI00692668;IPI00713474	Cellular Component	Microtubule organizing center	11	3	246	0.9499
GO:0030496	IPI00705237;IPI00714656;IPI00906031	Cellular Component	Midbody	5	3	246	0.4289
GO:0005741	IPI00698059;IPI00703084;IPI00906031	Cellular Component	Mitochondrial outer membrane	13	3	246	0.4289
GO:0000228	IPI00698339;IPI00705104;IPI00712399	Cellular Component	Nuclear chromosome	11	3	246	0.5554
GO:0005635	IPI00689226;IPI00698823;IPI00701747	Cellular Component	Nuclear envelope	11	3	246	0.9191
GO:0044451	IPI00688446;IPI00690309;IPI00906031	Cellular Component	Nucleoplasm part	12	3	246	0.9878
GO:0031968	IPI00698059;IPI00703084;IPI00906031	Cellular Component	Organelle outer membrane	7	3	246	0.5031
GO:0019867	IPI00698059;IPI00703084;IPI00906031	Cellular Component	Outer membrane	4	3	246	0.5209
GO:0034358	IPI00685653;IPI00690581;IPI00700673	Cellular Component	Plasma lipoprotein particle	6	3	246	0.1270
GO:0032994	IPI00685653;IPI00690581;IPI00700673	Cellular Component	Protein-lipid complex	4	3	246	0.1270
GO:0030667	IPI00685769;IPI00696116;IPI00709876	Cellular Component	Secretory granule membrane	12	3	246	0.0694
GO:0005681	IPI00686595;IPI00687395;IPI00688337	Cellular Component	Spliceosomal complex	8	3	246	0.9103
GO:0045202	IPI00685510;IPI00696116;IPI00876805	Cellular Component	Synapse	3	3	246	0.7518
GO:0002080	IPI00685769;IPI00709876	Cellular Component	Acrosomal membrane	13	2	246	0.1032
GO:0001669	IPI00685769;IPI00709876	Cellular Component	Acrosomal vesicle	12	2	246	0.2031
GO:0032432	IPI00697648;IPI00712288	Cellular Component	Actin filament bundle	11	2	246	0.6317
GO:0042641	IPI00697648;IPI00712288	Cellular Component	Actomyosin	11	2	246	0.6670
GO:0005912	IPI00708070;IPI00710274	Cellular Component	Adherens junction	5	2	246	0.9456
GO:0043296	IPI00713313;IPI00716830	Cellular Component	Apical junction complex	5	2	246	0.6130
GO:0030119	IPI00708037;IPI00866975	Cellular Component	AP-type membrane coat adaptor complex	10	2	246	0.3362
GO:0030424	IPI00685510;IPI00698916	Cellular Component	Axon	7	2	246	0.6995
GO:0071013	IPI00686595;IPI00687395	Cellular Component	Catalytic step 2 spliceosome	9	2	246	0.8601

GO:0032153	IPI00710274;IPI00906031	Cellular Component	Cell division site	5	2	246	0.4381
GO:0032155	IPI00710274;IPI00906031	Cellular Component	Cell division site part	6	2	246	0.4381
GO:0005813	IPI00687578;IPI00692668	Cellular Component	Centrosome	9	2	246	0.9574
GO:0042583	IPI00700622;IPI00707685	Cellular Component	Chromaffin granule	12	2	246	0.0424
GO:0000775	IPI00701747;IPI00906031	Cellular Component	Chromosome, centromeric region	12	2	246	0.7147
GO:0005801	IPI00708162;IPI00713460	Cellular Component	Cis-Golgi network	9	2	246	0.1268
GO:0030131	IPI00708037;IPI00866975	Cellular Component	Clathrin adaptor complex	11	2	246	0.3097
GO:0030118	IPI00708037;IPI00866975	Cellular Component	Clathrin coat	10	2	246	0.4623
GO:0030665	IPI00696116;IPI00866975	Cellular Component	Clathrin-coated vesicle membrane	13	2	246	0.3362
GO:0032154	IPI00710274;IPI00906031	Cellular Component	Cleavage furrow	7	2	246	0.4381
GO:0005905	IPI00706037;IPI00866975	Cellular Component	Coated pit	5	2	246	0.3881
GO:0005584	IPI00707857;IPI00708244	Cellular Component	Collagen type I trimer	7	2	246	0.0266
GO:0043292	IPI00697648;IPI00876805	Cellular Component	Contractile fiber	9	2	246	0.7694
GO:0044449	IPI00697648;IPI00876805	Cellular Component	Contractile fiber part	10	2	246	0.7147
GO:0030864	IPI00691093;IPI00710274	Cellular Component	Cortical actin cytoskeleton	12	2	246	0.3362
GO:0030425	IPI00685510;IPI00714269	Cellular Component	Dendrite	7	2	246	0.5311
GO:0030057	IPI00705237;IPI00708070	Cellular Component	Desmosome	5	2	246	0.2031
GO:0031901	IPI00693824;IPI00694198	Cellular Component	Early endosome membrane	11	2	246	0.2563
GO:0072546	IPI00714325;IPI00903861	Cellular Component	ER membrane protein complex	14	2	246	0.1515
GO:0030175	IPI00710274;IPI00924039	Cellular Component	Filopodium	6	2	246	0.3881
GO:0005795	IPI00703925;IPI00708162	Cellular Component	Golgi stack	11	2	246	0.2563
GO:0034364	IPI00685653;IPI00700673	Cellular Component	High-density lipoprotein particle	7	2	246	0.2563
GO:0030173	IPI00867237;IPI01003286	Cellular Component	Integral component of Golgi membrane	13	2	246	0.0607
GO:0005882	IPI00700471;IPI00708070	Cellular Component	Intermediate filament	11	2	246	0.4859
GO:0045111	IPI00700471;IPI00708070	Cellular Component	Intermediate filament cytoskeleton	10	2	246	0.6836
GO:0031228	IPI00867237;IPI01003286	Cellular Component	Intrinsic component of Golgi membrane	12	2	246	0.0607
GO:0000776	IPI00701747;IPI00906031	Cellular Component	Kinetochore	9	2	246	0.5936
GO:0005764	IPI00690309;IPI00698823	Cellular Component	Lysosome	11	2	246	0.9067
GO:0000323	IPI00690309;IPI00698823	Cellular Component	Lytic vacuole	10	2	246	0.9067
GO:0042579	IPI00688878;IPI00694430	Cellular Component	Microbody	9	2	246	0.7816
GO:0042645	IPI00690620;IPI00712858	Cellular Component	Mitochondrial nucleoid	12	2	246	0.4381

GO:0030016	IPI00697648;IPI00876805	Cellular Component	Myofibril	10	2	246	0.7293
GO:0016604	IPI00690309;IPI00906031	Cellular Component	Nuclear body	13	2	246	0.9264
GO:0000790	IPI00705104;IPI00712399	Cellular Component	Nuclear chromatin	13	2	246	0.5088
GO:0044454	IPI00705104;IPI00712399	Cellular Component	Nuclear chromosome part	12	2	246	0.6995
GO:0034399	IPI00689226;IPI00727591	Cellular Component	Nuclear periphery	12	2	246	0.6497
GO:0005643	IPI00689226;IPI00701747	Cellular Component	Nuclear pore	12	2	246	0.5936
GO:0009295	IPI00690620;IPI00712858	Cellular Component	Nucleoid	3	2	246	0.4381
GO:0070160	IPI00713313;IPI00716830	Cellular Component	Occluding junction	5	2	246	0.4134
GO:0005777	IPI00688878;IPI00694430	Cellular Component	Peroxisome	10	2	246	0.7816
GO:0046930	IPI00689226;IPI00701747	Cellular Component	Pore complex	7	2	246	0.7293
GO:0008287	IPI00906031;IPI01017588	Cellular Component	Protein serine/threonine phosphatase complex	5	2	246	0.4381
GO:0016469	IPI00686872;IPI00699372	Cellular Component	Proton-transporting two-sector ATPase complex	5	2	246	0.5088
GO:0033177	IPI00686872;IPI00699372	Cellular Component	Proton-transporting two-sector ATPase complex, proton-transporting domain	6	2	246	0.2031
GO:0055038	IPI00847122;IPI00867311	Cellular Component	Recycling endosome membrane	11	2	246	0.1770
GO:0005791	IPI00703954;IPI00715640	Cellular Component	Rough endoplasmic reticulum	10	2	246	0.2296
GO:0001726	IPI00693824;IPI00710274	Cellular Component	Ruffle	6	2	246	0.7293
GO:0030017	IPI00697648;IPI00876805	Cellular Component	Sarcomere	11	2	246	0.6670
GO:0000803	IPI00698339;IPI00712399	Cellular Component	Sex chromosome	10	2	246	0.1032
GO:0031201	IPI00696116;IPI00714660	Cellular Component	SNARE complex	9	2	246	0.1515
GO:0097223	IPI00685769;IPI00709876	Cellular Component	Sperm part	5	2	246	0.2563
GO:0051233	IPI00685510;IPI00705237	Cellular Component	Spindle midzone	10	2	246	0.0607
GO:0001725	IPI00697648;IPI00712288	Cellular Component	Stress fiber	12	2	246	0.6130
GO:0005923	IPI00713313;IPI00716830	Cellular Component	Tight junction	6	2	246	0.4134
GO:0030315	IPI00705159;IPI00843305	Cellular Component	T-tubule	7	2	246	0.0424
GO:0000151	IPI00704041;IPI00706142	Cellular Component	Ubiquitin ligase complex	7	2	246	0.5735
GO:0030120	IPI00692963;IPI00866975	Cellular Component	Vesicle coat	10	2	246	0.5088
GO:0005884	IPI00697648	Cellular Component	Actin filament	11	1	246	0.7666
GO:0031225	IPI00702768	Cellular Component	Anchored component of membrane	6	1	246	0.5504

GO:0030122	IPI00866975	Cellular Component	AP-2 adaptor complex	13	1	246	0.2520
GO:0005776	IPI00908033	Cellular Component	Autophagic vacuole	10	1	246	0.3044
GO:0001740	IPI00698339	Cellular Component	Barr body	12	1	246	0.2520
GO:0045178	IPI00697595	Cellular Component	Basal part of cell	5	1	246	0.3532
GO:0005604	IPI00712524	Cellular Component	Basement membrane	6	1	246	0.8791
GO:0005903	IPI00703813	Cellular Component	Brush border	6	1	246	0.5164
GO:0030014	IPI00704902	Cellular Component	CCR4-NOT complex	7	1	246	0.1351
GO:0031253	IPI00924039	Cellular Component	Cell projection membrane	7	1	246	0.8791
GO:0044291	IPI00708070	Cellular Component	Cell-cell contact zone	5	1	246	0.6640
GO:0030055	IPI00876805	Cellular Component	Cell-substrate junction	4	1	246	0.9721
GO:0005814	IPI00687578	Cellular Component	Centriole	10	1	246	0.2520
GO:0000781	IPI00712399	Cellular Component	Chromosome, telomeric region	12	1	246	0.3044
GO:0044441	IPI00685510	Cellular Component	Ciliary part	7	1	246	0.4799
GO:0035253	IPI00685510	Cellular Component	Ciliary rootlet	11	1	246	0.2520
GO:0005929	IPI00685510	Cellular Component	Cilium	6	1	246	0.7983
GO:0030132	IPI00866975	Cellular Component	Clathrin coat of coated pit	11	1	246	0.4799
GO:0030128	IPI00866975	Cellular Component	Clathrin coat of endocytic vesicle	12	1	246	0.2520
GO:0030125	IPI00866975	Cellular Component	Clathrin vesicle coat	11	1	246	0.4799
GO:0045334	IPI00866975	Cellular Component	Clathrin-coated endocytic vesicle	13	1	246	0.3044
GO:0030669	IPI00866975	Cellular Component	Clathrin-coated endocytic vesicle membrane	14	1	246	0.2520
GO:0005586	IPI00731432	Cellular Component	Collagen type III trimer	7	1	246	0.1351
GO:0005587	IPI00712524	Cellular Component	Collagen type IV trimer	7	1	246	0.1957
GO:0005588	IPI00826022	Cellular Component	Collagen type V trimer	7	1	246	0.1351
GO:0000777	IPI00906031	Cellular Component	Condensed chromosome kinetochore	10	1	246	0.4799
GO:0000779	IPI00906031	Cellular Component	Condensed chromosome, centromeric region	11	1	246	0.5164
GO:0000794	IPI00712399	Cellular Component	Condensed nuclear chromosome	12	1	246	0.5164
GO:0030127	IPI00692963	Cellular Component	COPII vesicle coat	11	1	246	0.3532
GO:0043034	IPI00876805	Cellular Component	Costamere	11	1	246	0.3532
GO:0031461	IPI00704041	Cellular Component	Cullin-RING ubiquitin ligase complex	8	1	246	0.5504
GO:0000307	IPI00696748	Cellular Component	Cyclin-dependent protein kinase holoenzyme complex	7	1	246	0.1351
GO:0005881	IPI00697917	Cellular Component	Cytoplasmic microtubule	9	1	246	0.3532
GO:0000932	IPI00704902	Cellular Component	Cytoplasmic mRNA processing body	10	1	246	0.6876
GO:0009898	IPI00705237	Cellular Component	Cytoplasmic side of plasma membrane	6	1	246	0.8791

GO:0043198	IPI00685510	Cellular Component	Dendritic shaft	8	1	246	0.3044
GO:0043197	IPI00685510	Cellular Component	Dendritic spine	8	1	246	0.3532
GO:0016010	IPI00876805	Cellular Component	Dystrophin-associated glycoprotein complex	7	1	246	0.3044
GO:0030666	IPI00866975	Cellular Component	Endocytic vesicle membrane	12	1	246	0.3985
GO:0005793	IPI00695852	Cellular Component	Endoplasmic reticulum-Golgi intermediate compartment	9	1	246	0.8380
GO:0030134	IPI00692963	Cellular Component	ER to Golgi transport vesicle	12	1	246	0.4407
GO:0012507	IPI00692963	Cellular Component	ER to Golgi transport vesicle membrane	13	1	246	0.3985
GO:0000178	IPI01017508	Cellular Component	Exosome (RNase complex)	7	1	246	0.4407
GO:0009897	IPI00697595	Cellular Component	External side of plasma membrane	6	1	246	0.9098
GO:0019897	IPI00689165	Cellular Component	Extrinsic component of plasma membrane	7	1	246	0.8699
GO:0001939	IPI00714656	Cellular Component	Female pronucleus	11	1	246	0.1351
GO:0031527	IPI00924039	Cellular Component	Filopodium membrane	8	1	246	0.3044
GO:0016600	IPI00685769	Cellular Component	Flotillin complex	8	1	246	0.2520
GO:0000938	IPI00699470	Cellular Component	GARP complex	11	1	246	0.1957
GO:0043073	IPI00712399	Cellular Component	Germ cell nucleus	10	1	246	0.2520
GO:0031985	IPI00708162	Cellular Component	Golgi cisterna	12	1	246	0.4799
GO:0035097	IPI00906031	Cellular Component	Histone methyltransferase complex	8	1	246	0.7096
GO:0031674	IPI00876805	Cellular Component	I band	12	1	246	0.8380
GO:0001772	IPI00685617	Cellular Component	Immunological synapse	7	1	246	0.3985
GO:0071458	IPI00715640	Cellular Component	Integral component of cytoplasmic side of endoplasmic reticulum membrane	14	1	246	0.1351
GO:0071556	IPI00715640	Cellular Component	Integral component of lumenal side of endoplasmic reticulum membrane	14	1	246	0.1351
GO:0008305	IPI00697595	Cellular Component	Integrin complex	8	1	246	0.4407
GO:0014704	IPI00708070	Cellular Component	Intercalated disc	6	1	246	0.6640
GO:0045171	IPI00687578	Cellular Component	Intercellular bridge	5	1	246	0.1351
GO:0045095	IPI00700471	Cellular Component	Keratin filament	12	1	246	0.4407
GO:0031258	IPI00924039	Cellular Component	Lamellipodium membrane	8	1	246	0.3532
GO:0005770	IPI00689556	Cellular Component	Late endosome	10	1	246	0.8494
GO:0031902	IPI00689556	Cellular Component	Late endosome membrane	11	1	246	0.6386
GO:0043219	IPI00714660	Cellular Component	Lateral loop	6	1	246	0.2520
GO:0031256	IPI00924039	Cellular Component	Leading edge membrane	7	1	246	0.7096
GO:0005811	IPI00694198	Cellular Component	Lipid particle	9	1	246	0.6640
GO:0034362	IPI00690581	Cellular Component	Low-density lipoprotein particle	7	1	246	0.3044

GO:0001673	IPI00712399	Cellular Component	Male germ cell nucleus	11	1	246	0.1957
GO:0034708	IPI00906031	Cellular Component	Methyltransferase complex	7	1	246	0.7096
GO:0031903	IPI00688878	Cellular Component	Microbody membrane	10	1	246	0.6640
GO:0044438	IPI00688878	Cellular Component	Microbody part	10	1	246	0.6876
GO:0044450	IPI00687578	Cellular Component	Microtubule organizing center part	9	1	246	0.4799
GO:0072686	IPI00705237	Cellular Component	Mitotic spindle	10	1	246	0.3044
GO:0070688	IPI00906031	Cellular Component	MLL5-L complex	9	1	246	0.3532
GO:0071817	IPI00712858	Cellular Component	MMXD complex	10	1	246	0.1957
GO:0031514	IPI00685510	Cellular Component	Motile cilium	7	1	246	0.5504
GO:0005847	IPI00688446	Cellular Component	mRNA cleavage and polyadenylation specificity factor complex	14	1	246	0.2520
GO:0005849	IPI00688446	Cellular Component	mRNA cleavage factor complex	13	1	246	0.3985
GO:0005771	IPI00689556	Cellular Component	Multivesicular body	11	1	246	0.3044
GO:0032585	IPI00689556	Cellular Component	Multivesicular body membrane	12	1	246	0.1957
GO:0043209	IPI00714660	Cellular Component	Myelin sheath	5	1	246	0.4799
GO:0035749	IPI00714660	Cellular Component	Myelin sheath adaxonal region	5	1	246	0.2520
GO:0036379	IPI00697648	Cellular Component	Myofilament	11	1	246	0.1957
GO:0031594	IPI00685510	Cellular Component	Neuromuscular junction	4	1	246	0.5819
GO:0044306	IPI00876805	Cellular Component	Neuron projection terminus	7	1	246	0.3985
GO:0044309	IPI00685510	Cellular Component	Neuron spine	7	1	246	0.3985
GO:0042564	IPI00698213	Cellular Component	NLS-dependent protein nuclear import complex	8	1	246	0.1351
GO:0019908	IPI00696748	Cellular Component	Nuclear cyclin-dependent protein kinase holoenzyme complex	8	1	246	0.1351
GO:0000176	IPI01017508	Cellular Component	Nuclear exosome (RNase complex)	8	1	246	0.1957
GO:0005652	IPI00727591	Cellular Component	Nuclear lamina	11	1	246	0.3985
GO:0016363	IPI00727591	Cellular Component	Nuclear matrix	11	1	246	0.8258
GO:0031965	IPI00689226	Cellular Component	Nuclear membrane	12	1	246	0.9162
GO:0016607	IPI00906031	Cellular Component	Nuclear speck	14	1	246	0.9327
GO:0031074	IPI00698213	Cellular Component	Nucleocytoplasmic shuttling complex	7	1	246	0.1351
GO:0031984	IPI00708162	Cellular Component	Organelle subcompartment	9	1	246	0.4799
GO:0005778	IPI00688878	Cellular Component	Peroxisomal membrane	12	1	246	0.6640
GO:0044439	IPI00688878	Cellular Component	Peroxisomal part	11	1	246	0.6876
GO:0045335	IPI00718152	Cellular Component	Phagocytic vesicle	12	1	246	0.5164
GO:0016605	IPI00690309	Cellular Component	PML body	14	1	246	0.3985
GO:0045120	IPI00714656	Cellular Component	Pronucleus	10	1	246	0.2520
GO:0000159	IPI01017588	Cellular Component	Protein phosphatase type 2A complex	7	1	246	0.5504
GO:0045259	IPI00686872	Cellular Component	Proton-transporting ATP synthase complex	7	1	246	0.6113

GO:0045263	IPI00686872	Cellular Component	Proton-transporting ATP synthase complex, coupling factor F(o)	8	1	246	0.3985
GO:0033176	IPI00699372	Cellular Component	Proton-transporting V-type ATPase complex	6	1	246	0.5164
GO:0033179	IPI00699372	Cellular Component	Proton-transporting V-type ATPase, V0 domain	7	1	246	0.3044
GO:0072357	IPI00906031	Cellular Component	PTW/PP1 phosphatase complex	6	1	246	0.3532
GO:0043235	IPI00697595	Cellular Component	Receptor complex	5	1	246	0.7831
GO:0005657	IPI00712399	Cellular Component	Replication fork	11	1	246	0.4799
GO:0035770	IPI00704902	Cellular Component	Ribonucleoprotein granule	8	1	246	0.8699
GO:0030867	IPI00703954	Cellular Component	Rough endoplasmic reticulum membrane	11	1	246	0.3985
GO:0016528	IPI00698916	Cellular Component	Sarcoplasm	8	1	246	0.5164
GO:0016529	IPI00698916	Cellular Component	Sarcoplasmic reticulum	9	1	246	0.5164
GO:0033017	IPI00698916	Cellular Component	Sarcoplasmic reticulum membrane	10	1	246	0.2520
GO:0005784	IPI00703954	Cellular Component	Sec61 translocon complex	13	1	246	0.1957
GO:0030935	IPI00712524	Cellular Component	Sheet-forming collagen trimer	6	1	246	0.1957
GO:0030532	IPI00688337	Cellular Component	Small nuclear ribonucleoprotein complex	8	1	246	0.6113
GO:0005790	IPI00692963	Cellular Component	Smooth endoplasmic reticulum	10	1	246	0.1351
GO:0030868	IPI00692963	Cellular Component	Smooth endoplasmic reticulum membrane	11	1	246	0.1351
GO:0097425	IPI00692963	Cellular Component	Smooth endoplasmic reticulum part	11	1	246	0.1351
GO:0042581	IPI00714660	Cellular Component	Specific granule	12	1	246	0.4407
GO:0008091	IPI00691093	Cellular Component	Spectrin	11	1	246	0.2520
GO:0014731	IPI00691093	Cellular Component	Spectrin-associated cytoskeleton	10	1	246	0.3044
GO:0005876	IPI00689379	Cellular Component	Spindle microtubule	10	1	246	0.4407
GO:0000922	IPI00705237	Cellular Component	Spindle pole	10	1	246	0.7096
GO:0005865	IPI00697648	Cellular Component	Striated muscle thin filament	12	1	246	0.1957
GO:0044456	IPI00696116	Cellular Component	Synapse part	3	1	246	0.8601
GO:0008021	IPI00696116	Cellular Component	Synaptic vesicle	11	1	246	0.5504
GO:0030672	IPI00696116	Cellular Component	Synaptic vesicle membrane	12	1	246	0.2520
GO:0070044	IPI00696116	Cellular Component	Synaptobrevin 2-SNAP-25-syntaxin-1a complex	10	1	246	0.1351

GO:0032588	IPI00867311	Cellular Component	Trans-Golgi network membrane	11	1	246	0.1957
GO:0071256	IPI00703954	Cellular Component	Translocon complex	12	1	246	0.1957
GO:0030133	IPI00692963	Cellular Component	Transport vesicle	11	1	246	0.8601
GO:0030658	IPI00692963	Cellular Component	Transport vesicle membrane	12	1	246	0.6640
GO:0034385	IPI00690581	Cellular Component	Triglyceride-rich lipoprotein particle	7	1	246	0.5504
GO:0005689	IPI00686595	Cellular Component	U12-type spliceosomal complex	9	1	246	0.5504
GO:0005683	IPI00688337	Cellular Component	U7 snRNP	9	1	246	0.2520
GO:0005774	IPI00699372	Cellular Component	Vacuolar membrane	11	1	246	0.8258
GO:0044437	IPI00699372	Cellular Component	Vacuolar part	10	1	246	0.8494
GO:0016471	IPI00699372	Cellular Component	Vacuolar proton-transporting V-type ATPase complex	12	1	246	0.3044
GO:0000220	IPI00699372	Cellular Component	Vacuolar proton-transporting V-type ATPase, V0 domain	11	1	246	0.2520
GO:0034361	IPI00690581	Cellular Component	Very-low-density lipoprotein particle	8	1	246	0.5504
GO:0000805	IPI00698339	Cellular Component	X chromosome	11	1	246	0.2520
GO:0001741	IPI00712399	Cellular Component	XY body	11	1	246	0.1957
GO:0030018	IPI00876805	Cellular Component	Z disc	13	1	246	0.8258
GO:0042588	IPI00696116	Cellular Component	Zymogen granule	12	1	246	0.1957
GO:0042589	IPI00696116	Cellular Component	Zymogen granule membrane	13	1	246	0.1351

GO:0003674	IPI00685510;IPI00685678;IPI00685695;IPI00685804;IPI00686595;IPI00686872;IPI00687375;IPI00687478;IPI00687634;IPI00687661;IPI00687662;IPI00687839;IPI00688337;IPI00688400;IPI00688446;IPI00688451;IPI00688727;IPI00688878;IPI00688977;IPI00688980;IPI00689165;IPI00689226;IPI00689365;IPI00689379;IPI00689565;IPI00689629;IPI00689641;IPI00690309;IPI00690355;IPI00690581;IPI00690620;IPI00690789;IPI00691086;IPI00691093;IPI00691649;IPI00691826;IPI00692186;IPI00692668;IPI00692963;IPI00693452;IPI00693579;IPI00693824;IPI00693842;IPI00694094;IPI00694198;IPI00694430;IPI00694444;IPI006	Molecular Function	Molecular_function	2	174	246	0.7949

GO:0005488	IPI00685510;IPI00685695;IPI00686595;IPI00686872;IPI00687375;IPI00687662;IPI00687839;IPI00688337;IPI00688400;IPI00688446;IPI00688451;IPI00688878;IPI00688980;IPI00689365;IPI00689629;IPI00689641;IPI00690309;IPI00690355;IPI00690581;IPI00690620;IPI00690789;IPI00691093;IPI00691826;IPI00692963;IPI00693579;IPI00693824;IPI00694198;IPI00694565;IPI00694597;IPI00694688;IPI00694758;IPI00695042;IPI00696116;IPI00697194;IPI00697648;IPI00697917;IPI00698077;IPI00698213;IPI00698339;IPI00698823;IPI00698916;IPI00699226;IPI00700228;IPI00702768;IPI00703022;IPI00703813;IPI00703925;IPI007	Molecular Function	Binding	3	99	246	0.9999

GO:0003824	IPI00685678;IPI00685804;IPI00687478;IPI00687661;IPI00687839;IPI00688446;IPI00688878;IPI00688977;IPI00689165;IPI00689365;IPI00689379;IPI00689629;IPI00690309;IPI00690620;IPI00690789;IPI00691649;IPI00693579;IPI00693824;IPI00694094;IPI00694430;IPI00694758;IPI00695042;IPI00695548;IPI00695952;IPI00697194;IPI00698059;IPI00698213;IPI00698916;IPI00699039;IPI00699226;IPI00699817;IPI00700228;IPI00702151;IPI00702251;IPI00702768;IPI00703022;IPI00703084;IPI00704078;IPI00704332;IPI00704902;IPI00705159;IPI00705565;IPI00706634;IPI00706879;IPI00707776;IPI00708246;IPI00709124;IPI007	Molecular Function	Catalytic activity	3	75	246	0.9991

GO:0043167	IPI00685510;IPI00685695;IPI00687375;IPI00687662;IPI00688400;IPI00688451;IPI00688980;IPI00689365;IPI00690309;IPI00690620;IPI00690789;IPI00691093;IPI00691826;IPI00692963;IPI00693824;IPI00694198;IPI00694758;IPI00695042;IPI00696116;IPI00697194;IPI00697648;IPI00697917;IPI00699226;IPI00700228;IPI00702768;IPI00703813;IPI00703925;IPI00704332;IPI00705159;IPI00706037;IPI00706634;IPI00707455;IPI00707857;IPI00708244;IPI00708246;IPI00709124;IPI00709888;IPI00710377;IPI00710664;IPI00713979;IPI00714098;IPI00715059;IPI00715174;IPI00715414;IPI00716157;IPI00717758;IPI00717764;IPI007	Molecular Function	Ion binding	4	65	246	0.9990

GO:0097159	IPI00685510;IPI00685695;IPI00687839;IPI00688337;IPI00688446;IPI00688878;IPI00688980;IPI00689365;IPI00689629;IPI00689641;IPI00690309;IPI00690620;IPI00690789;IPI00691826;IPI00693579;IPI00693824;IPI00694198;IPI00694565;IPI00694688;IPI00694758;IPI00695042;IPI00697194;IPI00697648;IPI00698077;IPI00698213;IPI00698339;IPI00698823;IPI00698916;IPI00699226;IPI00700228;IPI00703022;IPI00704332;IPI00704902;IPI00705104;IPI00705159;IPI00706634;IPI00707455;IPI00707776;IPI00708246;IPI00709124;IPI00709888;IPI00710377;IPI00712263;IPI00712399;IPI00714098;IPI00714898;IPI00716157;IPI007	Molecular Function	Organic cyclic compound binding	4	61	246	0.9904

GO:1901363	IPI00685510;IPI00685695;IPI00687839;IPI00688337;IPI00688446;IPI00688878;IPI00688980;IPI00689365;IPI00689629;IPI00689641;IPI00690309;IPI00690620;IPI00690789;IPI00691826;IPI00693579;IPI00693824;IPI00694198;IPI00694565;IPI00694688;IPI00694758;IPI00695042;IPI00697194;IPI00697648;IPI00698077;IPI00698213;IPI00698339;IPI00698916;IPI00699226;IPI00700228;IPI00703022;IPI00704332;IPI00704902;IPI00705104;IPI00705159;IPI00706634;IPI00707455;IPI00707776;IPI00708246;IPI00709124;IPI00709888;IPI00710377;IPI00712263;IPI00712399;IPI00714098;IPI00714898;IPI00716157;IPI00717110;IPI007	Molecular Function	Heterocyclic compound binding	4	60	246	0.9919

GO:0043168	IPI00685510;IPI00685695;IPI00688400;IPI00688451;IPI00688980;IPI00689365;IPI00690309;IPI00690620;IPI00690789;IPI00691093;IPI00691826;IPI00693824;IPI00694198;IPI00694758;IPI00695042;IPI00696116;IPI00697194;IPI00697648;IPI00699226;IPI00700228;IPI00703813;IPI00703925;IPI00704332;IPI00705159;IPI00706037;IPI00706634;IPI00707455;IPI00708246;IPI00709888;IPI00710377;IPI00710664;IPI00714098;IPI00715059;IPI00717758;IPI00718152;IPI00727050;IPI00727591;IPI00760457;IPI00829520;IPI00838420;IPI00843305;IPI00847122;IPI00866858;IPI00866975;IPI00867017;IPI00908033;IPI01000210;IPI010	Molecular Function	Anion binding	5	48	246	0.7021

GO:0036094	IPI00685695;IPI00688878;IPI00688980;IPI00689365;IPI00690309;IPI00690620;IPI00690789;IPI00691826;IPI00693824;IPI00694198;IPI00694758;IPI00695042;IPI00697194;IPI00697648;IPI00698823;IPI00699226;IPI00700228;IPI00704332;IPI00705159;IPI00706634;IPI00707455;IPI00708246;IPI00709888;IPI00710377;IPI00712263;IPI00714098;IPI00714898;IPI00717758;IPI00718152;IPI00727050;IPI00727591;IPI00760457;IPI00829520;IPI00838420;IPI00847122;IPI00867017;IPI00871114;IPI00908033	Molecular Function	Small molecule binding	4	38	246	0.9959

GO:1901265	IPI00685695;IPI00688878;IPI00688980;IPI00689365;IPI00690309;IPI00690620;IPI00690789;IPI00691826;IPI00693824;IPI00694198;IPI00694758;IPI00695042;IPI00697194;IPI00697648;IPI00699226;IPI00700228;IPI00704332;IPI00705159;IPI00706634;IPI00707455;IPI00708246;IPI00709888;IPI00710377;IPI00712263;IPI00714098;IPI00714898;IPI00717758;IPI00718152;IPI00727050;IPI00760457;IPI00829520;IPI00847122;IPI00867017;IPI00871114;IPI00908033	Molecular Function	Nucleoside phosphate binding	5	35	246	0.9976

GO:0000166	IPI00685695;IPI00688878;IPI00688980;IPI00689365;IPI00690309;IPI00690620;IPI00690789;IPI00691826;IPI00693824;IPI00694198;IPI00694758;IPI00695042;IPI00697194;IPI00697648;IPI00699226;IPI00700228;IPI00704332;IPI00705159;IPI00706634;IPI00707455;IPI00708246;IPI00709888;IPI00710377;IPI00712263;IPI00714098;IPI00714898;IPI00717758;IPI00718152;IPI00727050;IPI00760457;IPI00829520;IPI00847122;IPI00867017;IPI00871114;IPI00908033	Molecular Function	Nucleotide binding	5	35	246	0.9976
GO:0005198	IPI00687634;IPI00687662;IPI00688727;IPI00689226;IPI00689565;IPI00691093;IPI00694444;IPI00694565;IPI00694688;IPI00698077;IPI00699827;IPI00700471;IPI00702948;IPI00705739;IPI00707386;IPI00707537;IPI00707857;IPI00708210;IPI00708244;IPI00709409;IPI00709876;IPI00710859;IPI00712384;IPI00712524;IPI00715868;IPI00716830;IPI00716860;IPI00717048;IPI00718379;IPI00731432;IPI00826022;IPI00876805	Molecular Function	Structural molecule activity	3	32	246	7.51E-07

GO:0001882	IPI00685695;IPI00688980;IPI00689365;IPI00690309;IPI00690620;IPI00690789;IPI00691826;IPI00693824;IPI00694198;IPI00694758;IPI00695042;IPI00697648;IPI00699226;IPI00700228;IPI00704332;IPI00705159;IPI00706634;IPI00707455;IPI00708246;IPI00709888;IPI00710377;IPI00714098;IPI00717758;IPI00718152;IPI00727050;IPI00760457;IPI00829520;IPI00847122;IPI00867017;IPI00908033	Molecular Function	Nucleoside binding	5	30	246	0.8156
GO:0001883	IPI00685695;IPI00688980;IPI00689365;IPI00690309;IPI00690620;IPI00690789;IPI00691826;IPI00693824;IPI00694198;IPI00694758;IPI00695042;IPI00697648;IPI00699226;IPI00700228;IPI00704332;IPI00705159;IPI00706634;IPI00707455;IPI00708246;IPI00709888;IPI00710377;IPI00714098;IPI00717758;IPI00718152;IPI00727050;IPI00760457;IPI00829520;IPI00847122;IPI00867017;IPI00908033	Molecular Function	Purine nucleoside binding	6	30	246	0.8086

GO:0017076	IPI00685695;IPI00688980;IPI00689365;IPI00690309;IPI00690620;IPI00690789;IPI00691826;IPI00693824;IPI00694198;IPI00694758;IPI00695042;IPI00697648;IPI00699226;IPI00700228;IPI00704332;IPI00705159;IPI00706634;IPI00707455;IPI00708246;IPI00709888;IPI00710377;IPI00714098;IPI00717758;IPI00718152;IPI00727050;IPI00760457;IPI00829520;IPI00847122;IPI00867017;IPI00908033	Molecular Function	Purine nucleotide binding	6	30	246	0.8292
GO:0032550	IPI00685695;IPI00688980;IPI00689365;IPI00690309;IPI00690620;IPI00690789;IPI00691826;IPI00693824;IPI00694198;IPI00694758;IPI00695042;IPI00697648;IPI00699226;IPI00700228;IPI00704332;IPI00705159;IPI00706634;IPI00707455;IPI00708246;IPI00709888;IPI00710377;IPI00714098;IPI00717758;IPI00718152;IPI00727050;IPI00760457;IPI00829520;IPI00847122;IPI00867017;IPI00908033	Molecular Function	Purine ribonucleoside binding	7	30	246	0.8086

GO:0035639	IPI00685695;IPI00688980;IPI00689365;IPI00690309;IPI00690620;IPI00690789;IPI00691826;IPI00693824;IPI00694198;IPI00694758;IPI00695042;IPI00697648;IPI00699226;IPI00700228;IPI00704332;IPI00705159;IPI00706634;IPI00707455;IPI00708246;IPI00709888;IPI00710377;IPI00714098;IPI00717758;IPI00718152;IPI00727050;IPI00760457;IPI00829520;IPI00847122;IPI00867017;IPI00908033	Molecular Function	Purine ribonucleoside triphosphate binding	6	30	246	0.8013
GO:0032555	IPI00685695;IPI00688980;IPI00689365;IPI00690309;IPI00690620;IPI00690789;IPI00691826;IPI00693824;IPI00694198;IPI00694758;IPI00695042;IPI00697648;IPI00699226;IPI00700228;IPI00704332;IPI00705159;IPI00706634;IPI00707455;IPI00708246;IPI00709888;IPI00710377;IPI00714098;IPI00717758;IPI00718152;IPI00727050;IPI00760457;IPI00829520;IPI00847122;IPI00867017;IPI00908033	Molecular Function	Purine ribonucleotide binding	7	30	246	0.8225

GO:0032549	IPI00685695;IPI00688980;IPI00689365;IPI00690309;IPI00690620;IPI00690789;IPI00691826;IPI00693824;IPI00694198;IPI00694758;IPI00695042;IPI00697648;IPI00699226;IPI00700228;IPI00704332;IPI00705159;IPI00706634;IPI00707455;IPI00708246;IPI00709888;IPI00710377;IPI00714098;IPI00717758;IPI00718152;IPI00727050;IPI00760457;IPI00829520;IPI00847122;IPI00867017;IPI00908033	Molecular Function	Ribonucleoside binding	6	30	246	0.8121
GO:0032553	IPI00685695;IPI00688980;IPI00689365;IPI00690309;IPI00690620;IPI00690789;IPI00691826;IPI00693824;IPI00694198;IPI00694758;IPI00695042;IPI00697648;IPI00699226;IPI00700228;IPI00704332;IPI00705159;IPI00706634;IPI00707455;IPI00708246;IPI00709888;IPI00710377;IPI00714098;IPI00717758;IPI00718152;IPI00727050;IPI00760457;IPI00829520;IPI00847122;IPI00867017;IPI00908033	Molecular Function	Ribonucleotide binding	6	30	246	0.8452

GO:0043169	IPI00685510;IPI00687375;IPI00687662;IPI00688400;IPI00688451;IPI00692963;IPI00697917;IPI00699226;IPI00702768;IPI00703813;IPI00703925;IPI00705159;IPI00707857;IPI00708244;IPI00709124;IPI00709888;IPI00710377;IPI00710664;IPI00713979;IPI00715174;IPI00715414;IPI00716157;IPI00717764;IPI00730219;IPI00838420;IPI00876805;IPI00906031;IPI00935131;IPI01028455	Molecular Function	Cation binding	5	29	246	0.9994
GO:0046872	IPI00685510;IPI00687375;IPI00687662;IPI00688400;IPI00688451;IPI00692963;IPI00697917;IPI00699226;IPI00702768;IPI00703813;IPI00703925;IPI00705159;IPI00707857;IPI00708244;IPI00709124;IPI00709888;IPI00710377;IPI00710664;IPI00713979;IPI00715174;IPI00715414;IPI00716157;IPI00730219;IPI00838420;IPI00876805;IPI00906031;IPI00935131;IPI01028455	Molecular Function	Metal ion binding	6	28	246	0.9993

GO:0016787	IPI00685804;IPI00688446;IPI00689165;IPI00689379;IPI00690620;IPI00693579;IPI00693824;IPI00695042;IPI00699226;IPI00700228;IPI00702768;IPI00704902;IPI00705159;IPI00709124;IPI00709888;IPI00710377;IPI00710664;IPI00712596;IPI00715414;IPI00715640;IPI00717764;IPI00718152;IPI00839150;IPI00847122;IPI00906031;IPI01017508	Molecular Function	Hydrolase activity	4	26	246	0.9951
GO:0003676	IPI00685510;IPI00688337;IPI00688446;IPI00689365;IPI00689641;IPI00690309;IPI00693579;IPI00694565;IPI00694688;IPI00698077;IPI00698213;IPI00698339;IPI00700228;IPI00703022;IPI00704902;IPI00705104;IPI00707776;IPI00709124;IPI00712263;IPI00712399;IPI00867017;IPI01003286;IPI01017508;IPI01028455	Molecular Function	Nucleic acid binding	5	24	246	0.9842

GO:0005215	IPI00686872;IPI00691086;IPI00692186;IPI00693452;IPI00697349;IPI00699226;IPI00699372;IPI00703813;IPI00705159;IPI00705378;IPI00706314;IPI00708946;IPI00709888;IPI00710377;IPI00710920;IPI00711457;IPI00712858;IPI00714269;IPI00714656;IPI00714898;IPI00730219;IPI00854393	Molecular Function	Transporter activity	3	22	246	0.0112
GO:0003735	IPI00687634;IPI00687662;IPI00688727;IPI00689565;IPI00694444;IPI00694565;IPI00694688;IPI00698077;IPI00699827;IPI00702948;IPI00705739;IPI00707386;IPI00707537;IPI00708210;IPI00709409;IPI00710859;IPI00712384;IPI00715868;IPI00716860;IPI00717048;IPI00718379	Molecular Function	Structural constituent of ribosome	4	21	246	9.24E-07
GO:0016740	IPI00687661;IPI00688977;IPI00690309;IPI00694758;IPI00695952;IPI00698213;IPI00699039;IPI00699817;IPI00702151;IPI00703022;IPI00703084;IPI00704332;IPI00705565;IPI00706634;IPI00706879;IPI00707776;IPI00714098;IPI00715066;IPI00827121;IPI00852474;IPI01028182	Molecular Function	Transferase activity	4	21	246	0.8961

GO:0030554	IPI00685695;IPI00689365;IPI00690309;IPI00690620;IPI00690789;IPI00694758;IPI00697648;IPI00699226;IPI00700228;IPI00704332;IPI00705159;IPI00706634;IPI00707455;IPI00708246;IPI00709888;IPI00710377;IPI00714098;IPI00717758;IPI00760457;IPI00867017	Molecular Function	Adenyl nucleotide binding	7	20	246	0.9337
GO:0032559	IPI00685695;IPI00689365;IPI00690309;IPI00690620;IPI00690789;IPI00694758;IPI00697648;IPI00699226;IPI00700228;IPI00704332;IPI00705159;IPI00706634;IPI00707455;IPI00708246;IPI00709888;IPI00710377;IPI00714098;IPI00717758;IPI00760457;IPI00867017	Molecular Function	Adenyl ribonucleotide binding	8	20	246	0.9300
GO:0005524	IPI00685695;IPI00689365;IPI00690309;IPI00690620;IPI00690789;IPI00694758;IPI00697648;IPI00699226;IPI00700228;IPI00704332;IPI00705159;IPI00706634;IPI00707455;IPI00708246;IPI00709888;IPI00710377;IPI00714098;IPI00717758;IPI00760457;IPI00867017	Molecular Function	ATP binding	9	20	246	0.9198

GO:0022892	IPI00686872;IPI00691086;IPI00692186;IPI00693452;IPI00699226;IPI00699372;IPI00703813;IPI00705159;IPI00709888;IPI00710377;IPI00710920;IPI00711457;IPI00714269;IPI00714656;IPI00714898;IPI00730219;IPI00854393	Molecular Function	Substrate-specific transporter activity	4	17	246	0.0309
GO:0030234	IPI00685510;IPI00687375;IPI00688451;IPI00692668;IPI00696748;IPI00700622;IPI00707685;IPI00709876;IPI00710277;IPI00713474;IPI00717930;IPI00866858;IPI00867230;IPI01000210;IPI01017508;IPI01017588	Molecular Function	Enzyme regulator activity	3	16	246	0.4291
GO:0008289	IPI00686872;IPI00688400;IPI00688451;IPI00691093;IPI00694597;IPI00696116;IPI00698823;IPI00703925;IPI00706037;IPI00715059;IPI00717110;IPI00843305;IPI00866858;IPI00866975;IPI01000210;IPI01028455	Molecular Function	Lipid binding	4	16	246	0.1448
GO:0022891	IPI00686872;IPI00691086;IPI00692186;IPI00693452;IPI00699226;IPI00699372;IPI00703813;IPI00705159;IPI00709888;IPI00710377;IPI00710920;IPI00711457;IPI00714656;IPI00714898;IPI00730219;IPI00854393	Molecular Function	Substrate-specific transmembrane transporter activity	5	16	246	0.0030

GO:0022857	IPI00686872;IPI00691086;IPI00692186;IPI00693452;IPI00699226;IPI00699372;IPI00703813;IPI00705159;IPI00709888;IPI00710377;IPI00710920;IPI00711457;IPI00714656;IPI00714898;IPI00730219;IPI00854393	Molecular Function	Transmembrane transporter activity	4	16	246	0.0057
GO:0015075	IPI00686872;IPI00692186;IPI00693452;IPI00699226;IPI00699372;IPI00703813;IPI00705159;IPI00709888;IPI00710377;IPI00710920;IPI00711457;IPI00714898;IPI00730219;IPI00854393	Molecular Function	Ion transmembrane transporter activity	6	14	246	0.0068
GO:0016491	IPI00685678;IPI00687478;IPI00688878;IPI00691649;IPI00694094;IPI00697194;IPI00698059;IPI00704078;IPI00714621;IPI00716157;IPI00838420;IPI00867237;IPI00871114	Molecular Function	Oxidoreductase activity	4	13	246	0.9516
GO:0016817	IPI00690620;IPI00693824;IPI00695042;IPI00699226;IPI00700228;IPI00702768;IPI00705159;IPI00709888;IPI00710377;IPI00718152;IPI00847122	Molecular Function	Hydrolase activity, acting on acid anhydrides	5	11	246	0.8997
GO:0016818	IPI00690620;IPI00693824;IPI00695042;IPI00699226;IPI00700228;IPI00702768;IPI00705159;IPI00709888;IPI00710377;IPI00718152;IPI00847122	Molecular Function	Hydrolase activity, acting on acid anhydrides, in phosphorus-containing anhydrides	6	11	246	0.8965

GO:0005543	IPI00688400;IPI00688451;IPI00691093;IPI00696116;IPI00703925;IPI00706037;IPI00715059;IPI00843305;IPI00866858;IPI00866975;IPI01000210	Molecular Function	Phospholipid binding	5	11	246	0.2158
GO:0016462	IPI00690620;IPI00693824;IPI00695042;IPI00699226;IPI00700228;IPI00702768;IPI00705159;IPI00709888;IPI00710377;IPI00718152;IPI00847122	Molecular Function	Pyrophosphatase activity	7	11	246	0.8965
GO:0046914	IPI00685510;IPI00687662;IPI00688451;IPI00692963;IPI00697917;IPI00709888;IPI00710664;IPI00715414;IPI00838420;IPI00876805;IPI00935131	Molecular Function	Transition metal ion binding	7	11	246	0.9908
GO:0008324	IPI00686872;IPI00693452;IPI00699226;IPI00699372;IPI00703813;IPI00705159;IPI00709888;IPI00710920;IPI00711457;IPI00714898	Molecular Function	Cation transmembrane transporter activity	7	10	246	0.0316
GO:0003677	IPI00685510;IPI00689641;IPI00690309;IPI00698213;IPI00698339;IPI00705104;IPI00707776;IPI00712399;IPI01003286;IPI01028455	Molecular Function	DNA binding	6	10	246	0.8007
GO:0005525	IPI00688980;IPI00691826;IPI00693824;IPI00694198;IPI00695042;IPI00718152;IPI00727050;IPI00829520;IPI00847122;IPI00908033	Molecular Function	GTP binding	9	10	246	0.3677

GO:0019001	IPI00688980;IPI00691826;IPI00693824;IPI00694198;IPI00695042;IPI00718152;IPI00727050;IPI00829520;IPI00847122;IPI00908033	Molecular Function	Guanyl nucleotide binding	7	10	246	0.3871
GO:0032561	IPI00688980;IPI00691826;IPI00693824;IPI00694198;IPI00695042;IPI00718152;IPI00727050;IPI00829520;IPI00847122;IPI00908033	Molecular Function	Guanyl ribonucleotide binding	8	10	246	0.3871
GO:0017111	IPI00690620;IPI00693824;IPI00695042;IPI00699226;IPI00700228;IPI00705159;IPI00709888;IPI00710377;IPI00718152;IPI00847122	Molecular Function	Nucleoside-triphosphatase activity	8	10	246	0.8956
GO:0022804	IPI00692186;IPI00699226;IPI00703813;IPI00705159;IPI00709888;IPI00710377;IPI00710920;IPI00854393	Molecular Function	Active transmembrane transporter activity	5	8	246	0.0131
GO:0016788	IPI00693579;IPI00702768;IPI00704902;IPI00705159;IPI00709124;IPI00839150;IPI00906031;IPI01017508	Molecular Function	Hydrolase activity, acting on ester bonds	5	8	246	0.8313
GO:0022890	IPI00686872;IPI00693452;IPI00699226;IPI00699372;IPI00703813;IPI00705159;IPI00709888;IPI00711457	Molecular Function	Inorganic cation transmembrane transporter activity	8	8	246	0.0701
GO:0016874	IPI00689365;IPI00708246;IPI00717758;IPI00718678;IPI00727591;IPI00760457;IPI00825680;IPI00867017	Molecular Function	Ligase activity	4	8	246	0.6839

GO:0061134	IPI00685510;IPI00687375;IPI00700622;IPI00707685;IPI00709876;IPI00710277;IPI00717930;IPI00867230	Molecular Function	Peptidase regulator activity	4	8	246	0.1592
GO:0003723	IPI00688337;IPI00688446;IPI00693579;IPI00694565;IPI00694688;IPI00698077;IPI00712263;IPI01017508	Molecular Function	RNA binding	6	8	246	0.9978
GO:0004866	IPI00685510;IPI00687375;IPI00700622;IPI00707685;IPI00710277;IPI00717930;IPI00867230	Molecular Function	Endopeptidase inhibitor activity	6	7	246	0.1396
GO:0061135	IPI00685510;IPI00687375;IPI00700622;IPI00707685;IPI00710277;IPI00717930;IPI00867230	Molecular Function	Endopeptidase regulator activity	5	7	246	0.1664
GO:0004857	IPI00685510;IPI00687375;IPI00700622;IPI00707685;IPI00710277;IPI00717930;IPI00867230	Molecular Function	Enzyme inhibitor activity	4	7	246	0.3262
GO:0030414	IPI00685510;IPI00687375;IPI00700622;IPI00707685;IPI00710277;IPI00717930;IPI00867230	Molecular Function	Peptidase inhibitor activity	5	7	246	0.1483
GO:0016772	IPI00687661;IPI00690309;IPI00694758;IPI00698213;IPI00704332;IPI00706634;IPI00714098	Molecular Function	Transferase activity, transferring phosphorus-containing groups	5	7	246	0.9877
GO:0008270	IPI00687662;IPI00688451;IPI00692963;IPI00697917;IPI00715414;IPI00876805;IPI00935131	Molecular Function	Zinc ion binding	8	7	246	0.9946
GO:0030695	IPI00688451;IPI00692668;IPI00713474;IPI00866858;IPI01000210;IPI01017508	Molecular Function	GTPase regulator activity	5	6	246	0.5174

GO:0046873	IPI00693452;IPI00699226;IPI00703813;IPI00705159;IPI00709888;IPI00711457	Molecular Function	Metal ion transmembrane transporter activity	9	6	246	0.0077
GO:0060089	IPI00688400;IPI00698823;IPI00709888;IPI00716830;IPI00852533;IPI00924039	Molecular Function	Molecular transducer activity	3	6	246	0.4550
GO:0060589	IPI00688451;IPI00692668;IPI00713474;IPI00866858;IPI01000210;IPI01017508	Molecular Function	Nucleoside-triphosphatase regulator activity	4	6	246	0.5416
GO:0008233	IPI00685804;IPI00689165;IPI00689379;IPI00710664;IPI00715414;IPI00715640	Molecular Function	Peptidase activity	5	6	246	0.9140
GO:0070011	IPI00685804;IPI00689165;IPI00689379;IPI00710664;IPI00715414;IPI00715640	Molecular Function	Peptidase activity, acting on L-amino acid peptides	6	6	246	0.8817
GO:0004871	IPI00688400;IPI00698823;IPI00709888;IPI00716830;IPI00852533;IPI00924039	Molecular Function	Signal transducer activity	4	6	246	0.4550
GO:0008509	IPI00692186;IPI00703813;IPI00710920;IPI00730219;IPI00854393	Molecular Function	Anion transmembrane transporter activity	7	5	246	0.0480
GO:0016887	IPI00699226;IPI00700228;IPI00705159;IPI00709888;IPI00710377	Molecular Function	ATPase activity	9	5	246	0.7749
GO:0042623	IPI00699226;IPI00700228;IPI00705159;IPI00709888;IPI00710377	Molecular Function	ATPase activity, coupled	10	5	246	0.5996
GO:0005509	IPI00688400;IPI00709888;IPI00713979;IPI00730219;IPI00876805	Molecular Function	Calcium ion binding	7	5	246	0.9719
GO:0008144	IPI00687839;IPI00689629;IPI00690309;IPI00698916;IPI01028455	Molecular Function	Drug binding	4	5	246	0.0315
GO:0005201	IPI00707857;IPI00708244;IPI00712524;IPI00731432;IPI00826022	Molecular Function	Extracellular matrix structural constituent	4	5	246	0.0037

GO:0019003	IPI00691826;IPI00693824;IPI00694198;IPI00695042;IPI00718152	Molecular Function	GDP binding	9	5	246	0.0105
GO:0016301	IPI00690309;IPI00694758;IPI00704332;IPI00706634;IPI00714098	Molecular Function	Kinase activity	6	5	246	0.9929
GO:0016773	IPI00690309;IPI00694758;IPI00704332;IPI00706634;IPI00714098	Molecular Function	Phosphotransferase activity, alcohol group as acceptor	6	5	246	0.9780
GO:0004867	IPI00685510;IPI00700622;IPI00707685;IPI00710277;IPI00717930	Molecular Function	Serine-type endopeptidase inhibitor activity	7	5	246	0.1030
GO:0005083	IPI00688451;IPI00692668;IPI00713474;IPI00866858;IPI01000210	Molecular Function	Small GTPase regulator activity	6	5	246	0.4258
GO:0016746	IPI00688977;IPI00703084;IPI00705565;IPI00706879;IPI00852474	Molecular Function	Transferase activity, transferring acyl groups	5	5	246	0.2700
GO:0043492	IPI00699226;IPI00705159;IPI00709888;IPI00710377	Molecular Function	ATPase activity, coupled to movement of substances	11	4	246	0.1541
GO:0042625	IPI00699226;IPI00705159;IPI00709888;IPI00710377	Molecular Function	ATPase activity, coupled to transmembrane movement of ions	13	4	246	0.0724
GO:0015662	IPI00699226;IPI00705159;IPI00709888;IPI00710377	Molecular Function	ATPase activity, coupled to transmembrane movement of ions, phosphorylative mechanism	14	4	246	0.0173
GO:0042626	IPI00699226;IPI00705159;IPI00709888;IPI00710377	Molecular Function	ATPase activity, coupled to transmembrane movement of substances	12	4	246	0.1408
GO:0015085	IPI00693452;IPI00699226;IPI00709888;IPI00711457	Molecular Function	Calcium ion transmembrane transporter activity	10	4	246	0.0134
GO:0030246	IPI00690355;IPI00705583;IPI00708654;IPI00838420	Molecular Function	Carbohydrate binding	4	4	246	0.3711
GO:0031406	IPI00688400;IPI00727591;IPI00838420;IPI01028455	Molecular Function	Carboxylic acid binding	6	4	246	0.1678
GO:0003682	IPI00686595;IPI00698339;IPI00714898;IPI01003286	Molecular Function	Chromatin binding	4	4	246	0.4834

GO:0072509	IPI00693452;IPI00699226;IPI00709888;IPI00711457	Molecular Function	Divalent inorganic cation transmembrane transporter activity	9	4	246	0.0270
GO:0003924	IPI00693824;IPI00695042;IPI00718152;IPI00847122	Molecular Function	GTPase activity	9	4	246	0.6776
GO:0005085	IPI00713474;IPI00866858;IPI01000210;IPI01017508	Molecular Function	Guanyl-nucleotide exchange factor activity	3	4	246	0.1541
GO:0016820	IPI00699226;IPI00705159;IPI00709888;IPI00710377	Molecular Function	Hydrolase activity, acting on acid anhydrides, catalyzing transmembrane movement of substances	6	4	246	0.1541
GO:0016879	IPI00718678;IPI00727591;IPI00760457;IPI00825680	Molecular Function	Ligase activity, forming carbon-nitrogen bonds	5	4	246	0.7535
GO:0015077	IPI00686872;IPI00699372;IPI00703813;IPI00705159	Molecular Function	Monovalent inorganic cation transmembrane transporter activity	9	4	246	0.4037
GO:0004518	IPI00693579;IPI00704902;IPI00709124;IPI01017508	Molecular Function	Nuclease activity	6	4	246	0.1678
GO:0015405	IPI00699226;IPI00705159;IPI00709888;IPI00710377	Molecular Function	P-P-bond-hydrolysis-driven transmembrane transporter activity	7	4	246	0.1820
GO:0015399	IPI00699226;IPI00705159;IPI00709888;IPI00710377	Molecular Function	Primary active transmembrane transporter activity	6	4	246	0.1820
GO:0004672	IPI00690309;IPI00694758;IPI00706634;IPI00714098	Molecular Function	Protein kinase activity	7	4	246	0.9248
GO:0004674	IPI00690309;IPI00694758;IPI00706634;IPI00714098	Molecular Function	Protein serine/threonine kinase activity	8	4	246	0.7724
GO:0015291	IPI00692186;IPI00703813;IPI00710920;IPI00854393	Molecular Function	Secondary active transmembrane transporter activity	6	4	246	0.0134
GO:0017171	IPI00685804;IPI00689165;IPI00710664;IPI00715414	Molecular Function	Serine hydrolase activity	5	4	246	0.1678
GO:0008236	IPI00685804;IPI00689165;IPI00710664;IPI00715414	Molecular Function	Serine-type peptidase activity	7	4	246	0.1678
GO:0004812	IPI00689365;IPI00708246;IPI00867017	Molecular Function	Aminoacyl-tRNA ligase activity	7	3	246	0.4097

GO:0097367	IPI00685510;IPI00710664;IPI00717764	Molecular Function	Carbohydrate derivative binding	4	3	246	0.5384
GO:0016830	IPI00690789;IPI00694430;IPI00760457	Molecular Function	Carbon-carbon lyase activity	5	3	246	0.1781
GO:0016831	IPI00690789;IPI00694430;IPI00760457	Molecular Function	Carboxy-lyase activity	6	3	246	0.0824
GO:0019829	IPI00699226;IPI00705159;IPI00709888	Molecular Function	Cation-transporting ATPase activity	14	3	246	0.1605
GO:0016859	IPI00687839;IPI00689629;IPI00698916	Molecular Function	Cis-trans isomerase activity	5	3	246	0.1963
GO:0004175	IPI00689165;IPI00689379;IPI00715640	Molecular Function	Endopeptidase activity	7	3	246	0.9379
GO:0008047	IPI00688451;IPI00692668;IPI00709876	Molecular Function	Enzyme activator activity	4	3	246	0.9272
GO:0005528	IPI00687839;IPI00689629;IPI00698916	Molecular Function	FK506 binding	6	3	246	0.0367
GO:0015103	IPI00692186;IPI00703813;IPI00710920	Molecular Function	Inorganic anion transmembrane transporter activity	8	3	246	0.0574
GO:0016853	IPI00687839;IPI00689629;IPI00698916	Molecular Function	Isomerase activity	4	3	246	0.7732
GO:0016876	IPI00689365;IPI00708246;IPI00867017	Molecular Function	Ligase activity, forming aminoacyl-tRNA and related compounds	6	3	246	0.4097
GO:0016875	IPI00689365;IPI00708246;IPI00867017	Molecular Function	Ligase activity, forming carbon-oxygen bonds	5	3	246	0.4097
GO:0016829	IPI00690789;IPI00694430;IPI00760457	Molecular Function	Lyase activity	4	3	246	0.7040
GO:0005527	IPI00687839;IPI00689629;IPI00698916	Molecular Function	Macrolide binding	5	3	246	0.0367
GO:0008168	IPI00695952;IPI00703022;IPI00707776	Molecular Function	Methyltransferase activity	6	3	246	0.3903
GO:0003755	IPI00687839;IPI00689629;IPI00698916	Molecular Function	Peptidyl-prolyl cis-trans isomerase activity	6	3	246	0.1781
GO:0016791	IPI00702768;IPI00705159;IPI00906031	Molecular Function	Phosphatase activity	7	3	246	0.8367
GO:0035091	IPI00703925;IPI00706037;IPI00866975	Molecular Function	Phosphatidylinositol binding	6	3	246	0.3512
GO:0042578	IPI00702768;IPI00705159;IPI00906031	Molecular Function	Phosphoric ester hydrolase activity	6	3	246	0.9006
GO:0000988	IPI00707776;IPI00707815;IPI00714898	Molecular Function	Protein binding transcription factor activity	3	3	246	0.7287
GO:0004872	IPI00688400;IPI00698823;IPI00924039	Molecular Function	Receptor activity	3	3	246	0.5721

GO:0004540	IPI00693579;IPI00704902;IPI01017508	Molecular Function	Ribonuclease activity	7	3	246	0.0465
GO:0038023	IPI00688400;IPI00698823;IPI00924039	Molecular Function	Signaling receptor activity	4	3	246	0.3117
GO:1901681	IPI00685510;IPI00710664;IPI00727591	Molecular Function	Sulfur compound binding	4	3	246	0.4097
GO:0003712	IPI00707776;IPI00707815;IPI00714898	Molecular Function	Transcription cofactor activity	5	3	246	0.7040
GO:0000989	IPI00707776;IPI00707815;IPI00714898	Molecular Function	Transcription factor binding transcription factor activity	4	3	246	0.7287
GO:0016747	IPI00703084;IPI00706879;IPI00852474	Molecular Function	Transferase activity, transferring acyl groups other than amino-acyl groups	6	3	246	0.4479
GO:0016741	IPI00695952;IPI00703022;IPI00707776	Molecular Function	Transferase activity, transferring one-carbon groups	5	3	246	0.4097
GO:0004888	IPI00688400;IPI00698823;IPI00924039	Molecular Function	Transmembrane signaling receptor activity	5	3	246	0.1434
GO:0005545	IPI00703925;IPI00866975	Molecular Function	1-phosphatidylinositol binding	7	2	246	0.0607
GO:0008408	IPI00704902;IPI01017508	Molecular Function	3'-5' exonuclease activity	8	2	246	0.1032
GO:0000175	IPI00704902;IPI01017508	Molecular Function	3'-5'-exoribonuclease activity	10	2	246	0.0424
GO:0016881	IPI00718678;IPI00825680	Molecular Function	Acid-amino acid ligase activity	6	2	246	0.9011
GO:0015301	IPI00692186;IPI00854393	Molecular Function	Anion:anion antiporter activity	8	2	246	0.0266
GO:0015296	IPI00703813;IPI00710920	Molecular Function	Anion:cation symporter activity	9	2	246	0.0607
GO:0016209	IPI00690581;IPI00716157	Molecular Function	Antioxidant activity	3	2	246	0.6995
GO:0015297	IPI00692186;IPI00854393	Molecular Function	Antiporter activity	7	2	246	0.0424
GO:0005262	IPI00693452;IPI00711457	Molecular Function	Calcium channel activity	10	2	246	0.1268
GO:0005544	IPI00688400;IPI00843305	Molecular Function	Calcium-dependent phospholipid binding	6	2	246	0.2296
GO:0005388	IPI00699226;IPI00709888	Molecular Function	Calcium-transporting ATPase activity	15	2	246	0.0424
GO:0005261	IPI00693452;IPI00711457	Molecular Function	Cation channel activity	9	2	246	0.1770
GO:0015267	IPI00693452;IPI00711457	Molecular Function	Channel activity	6	2	246	0.4381
GO:0015108	IPI00692186;IPI00710920	Molecular Function	Chloride transmembrane transporter activity	9	2	246	0.1515
GO:0048037	IPI00697194;IPI01028455	Molecular Function	Cofactor binding	4	2	246	0.9984

GO:0004869	IPI00700622;IPI00867230	Molecular Function	Cysteine-type endopeptidase inhibitor activity	7	2	246	0.3362
GO:0004519	IPI00709124;IPI01017508	Molecular Function	Endonuclease activity	7	2	246	0.3623
GO:0004527	IPI00704902;IPI01017508	Molecular Function	Exonuclease activity	7	2	246	0.2563
GO:0016796	IPI00704902;IPI01017508	Molecular Function	Exonuclease activity, active with either ribo- or deoxyribonucleic acids and producing 5'-phosphomonoesters	8	2	246	0.0424
GO:0004532	IPI00704902;IPI01017508	Molecular Function	Exoribonuclease activity	8	2	246	0.0424
GO:0016896	IPI00704902;IPI01017508	Molecular Function	Exoribonuclease activity, producing 5'-phosphomonoesters	9	2	246	0.0424
GO:0004364	IPI00702151;IPI01028182	Molecular Function	Glutathione transferase activity	6	2	246	0.1770
GO:0005539	IPI00685510;IPI00710664	Molecular Function	Glycosaminoglycan binding	5	2	246	0.7147
GO:0005096	IPI00688451;IPI00692668	Molecular Function	GTPase activator activity	5	2	246	0.8343
GO:0020037	IPI00716157;IPI00717110	Molecular Function	Heme binding	6	2	246	0.5088
GO:0008201	IPI00685510;IPI00710664	Molecular Function	Heparin binding	6	2	246	0.5088
GO:0015078	IPI00686872;IPI00699372	Molecular Function	Hydrogen ion transmembrane transporter activity	10	2	246	0.7433
GO:0005452	IPI00692186;IPI00854393	Molecular Function	Inorganic anion exchanger activity	9	2	246	0.0266
GO:0005216	IPI00693452;IPI00711457	Molecular Function	Ion channel activity	8	2	246	0.4134
GO:0005506	IPI00710664;IPI00838420	Molecular Function	Iron ion binding	8	2	246	0.7293
GO:0030374	IPI00707776;IPI00714898	Molecular Function	Ligand-dependent nuclear receptor transcription coactivator activity	7	2	246	0.1770
GO:0033293	IPI00727591;IPI01028455	Molecular Function	Monocarboxylic acid binding	7	2	246	0.1515
GO:0001071	IPI00693842;IPI01003286	Molecular Function	Nucleic acid binding transcription factor activity	3	2	246	0.8753
GO:0016779	IPI00698213;IPI00704332	Molecular Function	Nucleotidyltransferase activity	6	2	246	0.5936
GO:0008374	IPI00703084;IPI00706879	Molecular Function	O-acyltransferase activity	7	2	246	0.1268
GO:0016667	IPI00687478;IPI00867237	Molecular Function	Oxidoreductase activity, acting on a sulfur group of donors	5	2	246	0.6317

GO:0016614	IPI00691649;IPI 00714621	Molecular Function	Oxidoreductase activity, acting on CH-OH group of donors	5	2	246	0.9264
GO:0022803	IPI00693452;IPI 00711457	Molecular Function	Passive transmembrane transporter activity	5	2	246	0.4381
GO:0005088	IPI00866858;IPI 01000210	Molecular Function	Ras guanyl-nucleotide exchange factor activity	4	2	246	0.3097
GO:0000975	IPI00690309;IPI 00707776	Molecular Function	Regulatory region DNA binding	7	2	246	0.7433
GO:0001067	IPI00690309;IPI 00707776	Molecular Function	Regulatory region nucleic acid binding	6	2	246	0.7433
GO:0005089	IPI00866858;IPI 01000210	Molecular Function	Rho guanyl-nucleotide exchange factor activity	5	2	246	0.2296
GO:0019843	IPI00694565;IPI 00698077	Molecular Function	rRNA binding	7	2	246	0.3362
GO:0008757	IPI00703022;IPI 00707776	Molecular Function	S-adenosylmethionine-dependent methyltransferase activity	7	2	246	0.3623
GO:0043565	IPI00690309;IPI 01003286	Molecular Function	Sequence-specific DNA binding	7	2	246	0.8147
GO:0003700	IPI00693842;IPI 01003286	Molecular Function	Sequence-specific DNA binding transcription factor activity	4	2	246	0.8753
GO:0019787	IPI00718678;IPI 00825680	Molecular Function	Small conjugating protein transferase activity	5	2	246	0.8247
GO:0015081	IPI00703813;IPI 00705159	Molecular Function	Sodium ion transmembrane transporter activity	10	2	246	0.1032
GO:0015294	IPI00703813;IPI 00710920	Molecular Function	Solute:cation symporter activity	8	2	246	0.0607
GO:0005496	IPI00698823;IPI 00717110	Molecular Function	Steroid binding	5	2	246	0.2831
GO:0022838	IPI00693452;IPI 00711457	Molecular Function	Substrate-specific channel activity	7	2	246	0.4134
GO:0015293	IPI00703813;IPI 00710920	Molecular Function	Symporter activity	7	2	246	0.1268
GO:0046906	IPI00716157;IPI 00717110	Molecular Function	Tetrapyrrole binding	5	2	246	0.5526
GO:0003713	IPI00707776;IPI 00714898	Molecular Function	Transcription coactivator activity	6	2	246	0.7293
GO:0044212	IPI00690309;IPI 00707776	Molecular Function	Transcription regulatory region DNA binding	8	2	246	0.7293
GO:0016765	IPI00702151;IPI 01028182	Molecular Function	Transferase activity, transferring alkyl or aryl (other than methyl) groups	5	2	246	0.4381

GO:0016757	IPI00699817;IPI00827121	Molecular Function	Transferase activity, transferring glycosyl groups	5	2	246	0.8753
GO:0016758	IPI00699817;IPI00827121	Molecular Function	Transferase activity, transferring hexosyl groups	6	2	246	0.6670
GO:0004842	IPI00718678;IPI00825680	Molecular Function	Ubiquitin-protein transferase activity	6	2	246	0.7931
GO:0019842	IPI00727591;IPI00838420	Molecular Function	Vitamin binding	5	2	246	0.3881
GO:0003841	IPI00706879	Molecular Function	1-acylglycerol-3-phosphate O-acyltransferase activity	9	1	246	0.1351
GO:0047545	IPI00691649	Molecular Function	2-hydroxyglutarate dehydrogenase activity	6	1	246	0.1351
GO:0080084	IPI00690309	Molecular Function	5S rDNA binding	9	1	246	0.1351
GO:0015464	IPI00688400	Molecular Function	Acetylcholine receptor activity	6	1	246	0.1351
GO:0008376	IPI00827121	Molecular Function	Acetylgalactosaminyltransferase activity	7	1	246	0.3985
GO:0016407	IPI00852474	Molecular Function	Acetyltransferase activity	7	1	246	0.6386
GO:0016880	IPI00760457	Molecular Function	Acid-ammonia (or amide) ligase activity	6	1	246	0.1351
GO:0015172	IPI00730219	Molecular Function	Acidic amino acid transmembrane transporter activity	9	1	246	0.3044
GO:0016411	IPI00706879	Molecular Function	Acylglycerol O-acyltransferase activity	8	1	246	0.1957
GO:0070566	IPI00704332	Molecular Function	Adenylyltransferase activity	7	1	246	0.3044
GO:0004813	IPI00689365	Molecular Function	Alanine-tRNA ligase activity	8	1	246	0.2520
GO:0043178	IPI00698823	Molecular Function	Alcohol binding	5	1	246	0.6113
GO:0031420	IPI00703813	Molecular Function	Alkali metal ion binding	7	1	246	0.3044
GO:0004035	IPI00702768	Molecular Function	Alkaline phosphatase activity	8	1	246	0.1351
GO:0033218	IPI00727591	Molecular Function	Amide binding	4	1	246	0.7831
GO:0016597	IPI00688400	Molecular Function	Amino acid binding	7	1	246	0.6386
GO:0015171	IPI00730219	Molecular Function	Amino acid transmembrane transporter activity	8	1	246	0.4799
GO:0016211	IPI00760457	Molecular Function	Ammonia ligase activity	7	1	246	0.1351
GO:0008060	IPI00688451	Molecular Function	ARF GTPase activator activity	6	1	246	0.5164
GO:0005086	IPI00713474	Molecular Function	ARF guanyl-nucleotide exchange factor activity	4	1	246	0.3532
GO:0016273	IPI00707776	Molecular Function	Arginine N-methyltransferase activity	8	1	246	0.2520

GO:0004815	IPI00867017	Molecular Function	Aspartate-tRNA ligase activity	8	1	246	0.1957
GO:0050560	IPI00867017	Molecular Function	Aspartate-tRNA(Asn) ligase activity	8	1	246	0.1351
GO:0042500	IPI00715640	Molecular Function	Aspartic endopeptidase activity, intramembrane cleaving	9	1	246	0.1351
GO:0004190	IPI00715640	Molecular Function	Aspartic-type endopeptidase activity	8	1	246	0.3044
GO:0070001	IPI00715640	Molecular Function	Aspartic-type peptidase activity	7	1	246	0.3044
GO:0008026	IPI00700228	Molecular Function	ATP-dependent helicase activity	11	1	246	0.9029
GO:0009374	IPI00727591	Molecular Function	Biotin binding	5	1	246	0.1351
GO:0004077	IPI00727591	Molecular Function	Biotin-[acetyl-CoA-carboxylase] ligase activity	7	1	246	0.1351
GO:0018271	IPI00727591	Molecular Function	Biotin-protein ligase activity	6	1	246	0.1351
GO:0004084	IPI00715066	Molecular Function	Branched-chain-amino-acid transaminase activity	7	1	246	0.1351
GO:0015556	IPI00730219	Molecular Function	C4-dicarboxylate transmembrane transporter activity	9	1	246	0.1957
GO:0004198	IPI00689379	Molecular Function	Calcium-dependent cysteine-type endopeptidase activity	9	1	246	0.3532
GO:1901505	IPI00691086	Molecular Function	Carbohydrate derivative transporter activity	4	1	246	0.2520
GO:1901476	IPI00714656	Molecular Function	Carbohydrate transporter activity	4	1	246	0.2520
GO:0046943	IPI00730219	Molecular Function	Carboxylic acid transmembrane transporter activity	7	1	246	0.5164
GO:0052689	IPI00839150	Molecular Function	Carboxylic ester hydrolase activity	6	1	246	0.7831
GO:0004180	IPI00715414	Molecular Function	Carboxypeptidase activity	8	1	246	0.3532
GO:0015377	IPI00710920	Molecular Function	Cation:chloride symporter activity	10	1	246	0.1351
GO:0015144	IPI00714656	Molecular Function	Ccarbohydrate transmembrane transporter activity	6	1	246	0.2520
GO:0017169	IPI00687661	Molecular Function	CDP-alcohol phosphatidyltransferase activity	7	1	246	0.1957
GO:0003881	IPI00687661	Molecular Function	CDP-diacylglycerol-inositol 3-phosphatidyltransferase activity	8	1	246	0.1351
GO:0008061	IPI00717764	Molecular Function	Chitin binding	5	1	246	0.1351

GO:0004568	IPI00717764	Molecular Function	Chitinase activity	7	1	246	0.1957
GO:0015485	IPI00698823	Molecular Function	Cholesterol binding	7	1	246	0.3985
GO:0033961	IPI00712596	Molecular Function	Cis-stilbene-oxide hydrolase activity	7	1	246	0.1351
GO:0050662	IPI00697194	Molecular Function	Coenzyme binding	5	1	246	0.9982
GO:0004197	IPI00689379	Molecular Function	Cysteine-type endopeptidase activity	8	1	246	0.7490
GO:0008234	IPI00689379	Molecular Function	Cysteine-type peptidase activity	7	1	246	0.9276
GO:0003684	IPI00712399	Molecular Function	Damaged DNA binding	7	1	246	0.5164
GO:0004140	IPI00704332	Molecular Function	Dephospho-CoA kinase activity	7	1	246	0.1351
GO:0005310	IPI00730219	Molecular Function	Dicarboxylic acid transmembrane transporter activity	8	1	246	0.3532
GO:0051213	IPI00838420	Molecular Function	Dioxygenase activity	5	1	246	0.5819
GO:0004163	IPI00690789	Molecular Function	Diphosphomevalonate decarboxylase activity	7	1	246	0.1351
GO:0015036	IPI00687478	Molecular Function	Disulfide oxidoreductase activity	6	1	246	0.7490
GO:0034061	IPI00698213	Molecular Function	DNA polymerase activity	7	1	246	0.3985
GO:0003887	IPI00698213	Molecular Function	DNA-directed DNA polymerase activity	8	1	246	0.2520
GO:0009055	IPI00687478	Molecular Function	Electron carrier activity	3	1	246	0.9327
GO:0016803	IPI00712596	Molecular Function	Ether hydrolase activity	6	1	246	0.3044
GO:0008238	IPI00715414	Molecular Function	Exopeptidase activity	7	1	246	0.8956
GO:0005504	IPI01028455	Molecular Function	Fatty acid binding	5	1	246	0.3532
GO:0004324	IPI00697194	Molecular Function	Ferredoxin-NADP+ reductase activity	7	1	246	0.1351
GO:0008199	IPI00710664	Molecular Function	Ferric iron binding	9	1	246	0.3532
GO:0050660	IPI00697194	Molecular Function	Flavin adenine dinucleotide binding	6	1	246	0.9221
GO:0016971	IPI00867237	Molecular Function	Flavin-linked sulfhydryl oxidase activity	8	1	246	0.1351
GO:0004381	IPI00827121	Molecular Function	Fucosylgalactoside 3-alpha-galactosyltransferase activity	8	1	246	0.1351
GO:0008378	IPI00827121	Molecular Function	Galactosyltransferase activity	7	1	246	0.3044
GO:0003840	IPI00705565	Molecular Function	Gamma-glutamyltransferase activity	7	1	246	0.1351
GO:0022836	IPI00693452	Molecular Function	Gated channel activity	7	1	246	0.6640
GO:0005355	IPI00714656	Molecular Function	Glucose transmembrane transporter activity	10	1	246	0.1351

GO:0004351	IPI00760457	Molecular Function	Glutamate decarboxylase activity	7	1	246	0.1351
GO:0004356	IPI00760457	Molecular Function	Glutamate-ammonia ligase activity	8	1	246	0.1351
GO:0004819	IPI00708246	Molecular Function	Glutamine-tRNA ligase activity	8	1	246	0.1351
GO:0004366	IPI00703084	Molecular Function	Glycerol-3-phosphate O-acyltransferase activity	8	1	246	0.1957
GO:0004380	IPI00827121	Molecular Function	Glycoprotein-fucosylgalactoside alpha-N-acetylgalactosaminyltransferase activity	8	1	246	0.1351
GO:0031072	IPI00690789	Molecular Function	Heat shock protein binding	5	1	246	0.3044
GO:0008158	IPI00698823	Molecular Function	Hedgehog receptor activity	6	1	246	0.1351
GO:0004386	IPI00700228	Molecular Function	Helicase activity	9	1	246	0.9375
GO:0004394	IPI00699039	Molecular Function	Heparan sulfate 2-O-sulfotransferase activity	8	1	246	0.1351
GO:0034483	IPI00699039	Molecular Function	Heparan sulfate sulfotransferase activity	7	1	246	0.1351
GO:0015149	IPI00714656	Molecular Function	Hexose transmembrane transporter activity	9	1	246	0.1957
GO:0042054	IPI00707776	Molecular Function	Histone methyltransferase activity	8	1	246	0.4407
GO:0035642	IPI00707776	Molecular Function	Histone methyltransferase activity (H3-R17 specific)	11	1	246	0.1351
GO:0008469	IPI00707776	Molecular Function	Histone-arginine N-methyltransferase activity	10	1	246	0.2520
GO:0030544	IPI00690789	Molecular Function	Hsp70 protein binding	6	1	246	0.2520
GO:0016801	IPI00712596	Molecular Function	Hydrolase activity, acting on ether bonds	5	1	246	0.3985
GO:0016798	IPI00717764	Molecular Function	Hydrolase activity, acting on glycosyl bonds	5	1	246	0.8380
GO:0004553	IPI00717764	Molecular Function	Hydrolase activity, hydrolyzing O-glycosyl compounds	6	1	246	0.7983
GO:0042802	IPI00690789	Molecular Function	Identical protein binding	5	1	246	0.8494
GO:0022839	IPI00693452	Molecular Function	Ion gated channel activity	8	1	246	0.6640
GO:0019207	IPI00696748	Molecular Function	Kinase regulator activity	4	1	246	0.8125
GO:0015179	IPI00730219	Molecular Function	L-amino acid transmembrane transporter activity	9	1	246	0.3985

GO:0031418	IPI00838420	Molecular Function	L-ascorbic acid binding	6	1	246	0.3532
GO:0015183	IPI00730219	Molecular Function	L-aspartate transmembrane transporter activity	10	1	246	0.1957
GO:0005313	IPI00730219	Molecular Function	L-glutamate transmembrane transporter activity	9	1	246	0.3044
GO:0022834	IPI00693452	Molecular Function	Ligand-gated channel activity	8	1	246	0.3532
GO:0015276	IPI00693452	Molecular Function	Ligand-gated ion channel activity	9	1	246	0.3532
GO:0016298	IPI00839150	Molecular Function	Lipase activity	6	1	246	0.4799
GO:0071813	IPI00705441	Molecular Function	Lipoprotein particle binding	6	1	246	0.4799
GO:0052656	IPI00715066	Molecular Function	L-isoleucine transaminase activity	8	1	246	0.1351
GO:0052654	IPI00715066	Molecular Function	L-leucine transaminase activity	8	1	246	0.1351
GO:0030169	IPI00705441	Molecular Function	Low-density lipoprotein particle binding	7	1	246	0.3044
GO:0052655	IPI00715066	Molecular Function	L-valine transaminase activity	8	1	246	0.1351
GO:0004622	IPI00839150	Molecular Function	Lysophospholipase activity	8	1	246	0.1351
GO:0050080	IPI00694430	Molecular Function	Malonyl-CoA decarboxylase activity	7	1	246	0.1351
GO:0030145	IPI00709888	Molecular Function	Manganese ion binding	8	1	246	0.6640
GO:0005384	IPI00709888	Molecular Function	Manganese ion transmembrane transporter activity	11	1	246	0.1351
GO:0015410	IPI00709888	Molecular Function	Manganese-transporting ATPase activity	15	1	246	0.1351
GO:0004181	IPI00715414	Molecular Function	Metallocarboxypeptidas e activity	9	1	246	0.2520
GO:0008191	IPI00687375	Molecular Function	Metalloendopeptidase inhibitor activity	7	1	246	0.1957
GO:0048551	IPI00687375	Molecular Function	Metalloenzyme inhibitor activity	5	1	246	0.1957
GO:0010576	IPI00687375	Molecular Function	Metalloenzyme regulator activity	4	1	246	0.1957
GO:0008235	IPI00715414	Molecular Function	Metalloexopeptidase activity	8	1	246	0.5164
GO:0008237	IPI00715414	Molecular Function	Metallopeptidase activity	7	1	246	0.9327
GO:0072341	IPI00688400	Molecular Function	Modified amino acid binding	4	1	246	0.5819
GO:0048029	IPI00838420	Molecular Function	Monosaccharide binding	5	1	246	0.6640
GO:0015145	IPI00714656	Molecular Function	Monosaccharide transmembrane transporter activity	8	1	246	0.1957

GO:0004483	IPI00703022	Molecular Function	mRNA (nucleoside-2'-O-)-methyltransferase activity	8	1	246	0.1351
GO:0003729	IPI00694688	Molecular Function	mRNA binding	8	1	246	0.9375
GO:0008174	IPI00703022	Molecular Function	mRNA methyltransferase activity	8	1	246	0.1351
GO:0008080	IPI00852474	Molecular Function	N-acetyltransferase activity	8	1	246	0.4799
GO:0016410	IPI00852474	Molecular Function	N-acyltransferase activity	7	1	246	0.5164
GO:0003955	IPI00685678	Molecular Function	NAD(P)H dehydrogenase (quinone) activity	7	1	246	0.1957
GO:0050661	IPI00697194	Molecular Function	NADP binding	6	1	246	0.7831
GO:0030594	IPI00688400	Molecular Function	Neurotransmitter receptor activity	5	1	246	0.1351
GO:0008170	IPI00707776	Molecular Function	N-methyltransferase activity	7	1	246	0.4407
GO:0015932	IPI00691086	Molecular Function	Nucleobase-containing compound transmembrane transporter activity	6	1	246	0.1957
GO:0005337	IPI00691086	Molecular Function	Nucleoside transmembrane transporter activity	7	1	246	0.1957
GO:0005549	IPI00708946	Molecular Function	Odorant binding	4	1	246	0.2520
GO:0008171	IPI00703022	Molecular Function	O-methyltransferase activity	7	1	246	0.3044
GO:0005342	IPI00730219	Molecular Function	Organic acid transmembrane transporter activity	6	1	246	0.5164
GO:0008514	IPI00730219	Molecular Function	Organic anion transmembrane transporter activity	8	1	246	0.5504
GO:0016670	IPI00867237	Molecular Function	Oxidoreductase activity, acting on a sulfur group of donors, oxygen as acceptor	6	1	246	0.1957
GO:0016730	IPI00697194	Molecular Function	Oxidoreductase activity, acting on iron-sulfur proteins as donors	5	1	246	0.1351
GO:0016731	IPI00697194	Molecular Function	Oxidoreductase activity, acting on iron-sulfur proteins as donors, NAD or NADP as acceptor	6	1	246	0.1351
GO:0016651	IPI00685678	Molecular Function	Oxidoreductase activity, acting on NAD(P)H	5	1	246	0.9221

GO:0016655	IPI00685678	Molecular Function	Oxidoreductase activity, acting on NAD(P)H, quinone or similar compound as acceptor	6	1	246	0.7983
GO:0016705	IPI00838420	Molecular Function	Oxidoreductase activity, acting on paired donors, with incorporation or reduction of molecular oxygen	5	1	246	0.8956
GO:0016706	IPI00838420	Molecular Function	Oxidoreductase activity, acting on paired donors, with incorporation or reduction of molecular oxygen, 2-oxoglutarate as one donor, and incorporation of one atom each of oxygen into both donors	6	1	246	0.4799
GO:0016684	IPI00716157	Molecular Function	Oxidoreductase activity, acting on peroxide as acceptor	5	1	246	0.6876
GO:0016701	IPI00838420	Molecular Function	Oxidoreductase activity, acting on single donors with incorporation of molecular oxygen	5	1	246	0.5164
GO:0016702	IPI00838420	Molecular Function	Oxidoreductase activity, acting on single donors with incorporation of molecular oxygen, incorporation of two atoms of oxygen	6	1	246	0.5164
GO:0016627	IPI00704078	Molecular Function	Oxidoreductase activity, acting on the CH-CH group of donors	5	1	246	0.8956
GO:0016628	IPI00704078	Molecular Function	Oxidoreductase activity, acting on the CH-CH group of donors, NAD or NADP as acceptor	6	1	246	0.6113
GO:0016645	IPI00871114	Molecular Function	Oxidoreductase activity, acting on the CH-NH group of donors	5	1	246	0.6640
GO:0016646	IPI00871114	Molecular Function	Oxidoreductase activity, acting on the CH-NH group of donors, NAD or NADP as acceptor	6	1	246	0.5819

GO:0016616	IPI00714621	Molecular Function	Oxidoreductase activity, acting on the CH-OH group of donors, NAD or NADP as acceptor	6	1	246	0.9793
GO:0019825	IPI01028455	Molecular Function	Oxygen binding	4	1	246	0.2520
GO:0004595	IPI00704332	Molecular Function	Pantetheine-phosphate adenylyltransferase activity	8	1	246	0.1351
GO:0016504	IPI00709876	Molecular Function	Peptidase activator activity	5	1	246	0.6113
GO:0031545	IPI00838420	Molecular Function	Peptidyl-proline 4-dioxygenase activity	8	1	246	0.2520
GO:0031543	IPI00838420	Molecular Function	Peptidyl-proline dioxygenase activity	7	1	246	0.2520
GO:0004601	IPI00716157	Molecular Function	Peroxidase activity	6	1	246	0.6876
GO:0019208	IPI01017588	Molecular Function	Phosphatase regulator activity	4	1	246	0.5504
GO:0042301	IPI00703813	Molecular Function	Phosphate ion binding	6	1	246	0.2520
GO:0015114	IPI00703813	Molecular Function	Phosphate ion transmembrane transporter activity	9	1	246	0.1351
GO:1901981	IPI00703925	Molecular Function	Phosphatidylinositol phosphate binding	7	1	246	0.5504
GO:0005547	IPI00703925	Molecular Function	Phosphatidylinositol-3,4,5-trisphosphate binding	8	1	246	0.2520
GO:0043325	IPI00703925	Molecular Function	Phosphatidylinositol-3,4-bisphosphate binding	9	1	246	0.1351
GO:0001786	IPI00688400	Molecular Function	Phosphatidylserine binding	6	1	246	0.3532
GO:0004620	IPI00839150	Molecular Function	Phospholipase activity	7	1	246	0.3985
GO:0004721	IPI00906031	Molecular Function	Phosphoprotein phosphatase activity	8	1	246	0.9098
GO:0016780	IPI00687661	Molecular Function	Phosphotransferase activity, for other substituted phosphate groups	6	1	246	0.1957
GO:0004535	IPI00704902	Molecular Function	Poly(A)-specific ribonuclease activity	11	1	246	0.1351
GO:0015079	IPI00705159	Molecular Function	Potassium ion transmembrane transporter activity	10	1	246	0.2520
GO:0008556	IPI00705159	Molecular Function	Potassium-transporting ATPase activity	15	1	246	0.1957
GO:0036002	IPI00712263	Molecular Function	Pre-mRNA binding	7	1	246	0.3044
GO:0004656	IPI00838420	Molecular Function	Procollagen-proline 4-dioxygenase activity	9	1	246	0.2520

GO:0019798	IPI00838420	Molecular Function	Procollagen-proline dioxygenase activity	8	1	246	0.2520
GO:0005515	IPI00690789	Molecular Function	Protein binding	4	1	246	0.9999
GO:0046983	IPI00690789	Molecular Function	Protein dimerization activity	5	1	246	0.8494
GO:0015035	IPI00687478	Molecular Function	Protein disulfide oxidoreductase activity	7	1	246	0.7096
GO:0042803	IPI00690789	Molecular Function	Protein homodimerization activity	6	1	246	0.8125
GO:0008276	IPI00707776	Molecular Function	Protein methyltransferase activity	7	1	246	0.4407
GO:0019888	IPI01017588	Molecular Function	Protein phosphatase regulator activity	5	1	246	0.4799
GO:0008601	IPI01017588	Molecular Function	Protein phosphatase type 2A regulator activity	6	1	246	0.3532
GO:0004722	IPI00906031	Molecular Function	Protein serine/threonine phosphatase activity	9	1	246	0.6113
GO:0008565	IPI00714269	Molecular Function	Protein transporter activity	5	1	246	0.9221
GO:0016274	IPI00707776	Molecular Function	Protein-arginine N-methyltransferase activity	9	1	246	0.2520
GO:0035242	IPI00707776	Molecular Function	Protein-arginine omega-N asymmetric methyltransferase activity	10	1	246	0.1351
GO:0071814	IPI00705441	Molecular Function	Protein-lipid complex binding	5	1	246	0.4799
GO:0070035	IPI00700228	Molecular Function	Purine NTP-dependent helicase activity	10	1	246	0.9029
GO:0030170	IPI01028455	Molecular Function	Pyridoxal phosphate binding	6	1	246	0.8494
GO:0005097	IPI00692668	Molecular Function	Rab GTPase activator activity	7	1	246	0.5504
GO:0005099	IPI00692668	Molecular Function	Ras GTPase activator activity	6	1	246	0.7666
GO:0000182	IPI00690309	Molecular Function	rDNA binding	8	1	246	0.1351
GO:0043021	IPI00690309	Molecular Function	Ribonucleoprotein complex binding	4	1	246	0.8380
GO:0043022	IPI00690309	Molecular Function	Ribosome binding	5	1	246	0.6386
GO:0008173	IPI00703022	Molecular Function	RNA methyltransferase activity	7	1	246	0.2520
GO:0000982	IPI00693842	Molecular Function	RNA polymerase II core promoter proximal region sequence-specific DNA binding transcription factor activity	6	1	246	0.4407

GO:0001078	IPI00693842	Molecular Function	RNA polymerase II core promoter proximal region sequence-specific DNA binding transcription factor activity involved in negative regulation of transcription	7	1	246	0.2520
GO:0001227	IPI00693842	Molecular Function	RNA polymerase II transcription regulatory region sequence-specific DNA binding transcription factor activity involved in negative regulation of transcription	6	1	246	0.3044
GO:0001016	IPI00690309	Molecular Function	RNA polymerase III regulatory region DNA binding	9	1	246	0.1957
GO:0001030	IPI00690309	Molecular Function	RNA polymerase III type 1 promoter DNA binding	10	1	246	0.1351
GO:0001031	IPI00690309	Molecular Function	RNA polymerase III type 2 promoter DNA binding	10	1	246	0.1351
GO:0001032	IPI00690309	Molecular Function	RNA polymerase III type 3 promoter DNA binding	10	1	246	0.1351
GO:0047131	IPI00871114	Molecular Function	Saccharopine dehydrogenase (NAD+, L-glutamate-forming) activity	8	1	246	0.1351
GO:0047130	IPI00871114	Molecular Function	Saccharopine dehydrogenase (NADP+, L-lysine-forming) activity	8	1	246	0.1351
GO:0004753	IPI00871114	Molecular Function	Saccharopine dehydrogenase activity	7	1	246	0.1351
GO:0008430	IPI00690581	Molecular Function	Selenium binding	4	1	246	0.3044
GO:0000981	IPI00693842	Molecular Function	Sequence-specific DNA binding RNA polymerase II transcription factor activity	5	1	246	0.6640
GO:0004185	IPI00715414	Molecular Function	Serine-type carboxypeptidase activity	9	1	246	0.1957
GO:0004252	IPI00689165	Molecular Function	Serine-type endopeptidase activity	8	1	246	0.8125
GO:0070008	IPI00715414	Molecular Function	Serine-type exopeptidase activity	8	1	246	0.2520
GO:0031402	IPI00703813	Molecular Function	Sodium ion binding	8	1	246	0.1957

GO:0005436	IPI00703813	Molecular Function	Sodium:phosphate symporter activity	10	1	246	0.1351
GO:0005391	IPI00705159	Molecular Function	Sodium:potassium-exchanging ATPase activity	16	1	246	0.1957
GO:0015321	IPI00703813	Molecular Function	Sodium-dependent phosphate transmembrane transporter activity	10	1	246	0.1351
GO:0032934	IPI00698823	Molecular Function	Sterol binding	6	1	246	0.4799
GO:0015279	IPI00693452	Molecular Function	Store-operated calcium channel activity	10	1	246	0.1957
GO:0005200	IPI00691093	Molecular Function	Structural constituent of cytoskeleton	4	1	246	0.5504
GO:0008307	IPI00876805	Molecular Function	Structural constituent of muscle	4	1	246	0.4799
GO:0017056	IPI00689226	Molecular Function	Structural constituent of nuclear pore	4	1	246	0.1957
GO:0051119	IPI00714656	Molecular Function	Sugar transmembrane transporter activity	7	1	246	0.1957
GO:0008146	IPI00699039	Molecular Function	Sulfotransferase activity	6	1	246	0.1957
GO:0016972	IPI00867237	Molecular Function	Thiol oxidase activity	7	1	246	0.1957
GO:0015643	IPI01028455	Molecular Function	Toxic substance binding	4	1	246	0.1957
GO:0047115	IPI00704078	Molecular Function	Trans-1,2-dihydrobenzene-1,2-diol dehydrogenase activity	7	1	246	0.1351
GO:0008483	IPI00715066	Molecular Function	Transaminase activity	6	1	246	0.4407
GO:0016755	IPI00705565	Molecular Function	Transferase activity, transferring amino-acyl groups	6	1	246	0.3985
GO:0016769	IPI00715066	Molecular Function	Transferase activity, transferring nitrogenous groups	5	1	246	0.4799
GO:0016782	IPI00699039	Molecular Function	Transferase activity, transferring sulfur-containing groups	5	1	246	0.3985
GO:0046915	IPI00709888	Molecular Function	Transition metal ion transmembrane transporter activity	10	1	246	0.1957
GO:0045182	IPI00694565	Molecular Function	Translation regulator activity	3	1	246	0.3044
GO:0035250	IPI00827121	Molecular Function	UDP-galactosyltransferase activity	7	1	246	0.3044
GO:0008194	IPI00827121	Molecular Function	UDP-glycosyltransferase activity	6	1	246	0.6640

S gene number indicated the number of significantly down-regulated proteins annotated to one certain GO term by GO analysis; TS gene number indicated the total number of significantly down-regulated proteins annotated by GO analysis.

Table S9 KEGG pathway enrichment of the differentially expressed transcripts in mammary glands between RS- and AH-fed dairy cows

KEGG pathway id	KEGG pathway description	Gene id	No. of mapping ST	No. of mapping TT	P-value of Fisher'exact Test
bta04960	Aldosterone-regulated sodium reabsorption	XLOC_007981;XLOC_012793	2	110	0.0524
bta00512	Mucin type O-Glycan biosynthesis	XLOC_005214	1	110	0.0569
bta04070	Phosphatidylinositol signaling system	XLOC_003678;XLOC_023566;XLOC_024265	3	110	0.0734
bta05145	Toxoplasmosis	XLOC_015601;XLOC_020088;XLOC_023480	3	110	0.0734
bta01100	Metabolic pathways	XLOC_003697;XLOC_005319;XLOC_010523;XLOC_011982;XLOC_012858;XLOC_017018;XLOC_020095;XLOC_020698;XLOC_023501;XLOC_023529;XLOC_023555;XLOC_023596;XLOC_023606;XLOC_023703;XLOC_023920;XLOC_023921;XLOC_023922;XLOC_024053;XLOC_024196;XLOC_024260;XLOC_024302	21	110	0.0903
bta05218	Melanoma	XLOC_023297;XLOC_023651	2	110	0.0937
bta03320	PPAR signaling pathway	XLOC_024190;XLOC_024579	2	110	0.1217
bta00190	Oxidative phosphorylation	XLOC_023495	1	110	0.1364
bta04340	Hedgehog signaling pathway	XLOC_025531	1	110	0.1613
bta04115	p53 signaling pathway	XLOC_023747;XLOC_026769	2	110	0.1720
bta05414	Dilated cardiomyopathy	XLOC_007166;XLOC_008893;XLOC_011852	3	110	0.1815
bta05142	Chagas disease	XLOC_017082	1	110	0.1856
bta04350	TGF-beta signaling pathway	XLOC_012264;XLOC_013257	2	110	0.2036
bta04530	Tight junction	XLOC_016188;XLOC_020249	2	110	0.2036
bta00620	Pyruvate metabolism	XLOC_002658	1	110	0.2092
bta04510	Focal adhesion	XLOC_024081	1	110	0.2092
bta05214	Glioma	XLOC_023465	1	110	0.2092
bta00520	Amino sugar and nucleotide sugar metabolism	XLOC_007525	1	110	0.2320
bta05410	Hypertrophic cardiomyopathy	XLOC_020881	1	110	0.2320
bta04120	Ubiquitin mediated proteolysis	XLOC_023609;XLOC_024105;XLOC_024155	3	110	0.2419
bta04210	Apoptosis	XLOC_023481	1	110	0.2543
bta04380	Osteoclast differentiation	XLOC_009240	1	110	0.2543
bta04620	Toll-like receptor signaling pathway	XLOC_024161	1	110	0.2543
bta04974	Protein digestion and absorption	XLOC_001311;XLOC_009913	2	110	0.2574

bta04010	MAPK signaling pathway	XLOC_013166;XLOC_023520	2	110	0.2682
bta04910	Insulin signaling pathway	XLOC_023531;XLOC_026730	2	110	0.2790
bta04512	ECM-receptor interaction	XLOC_012279	1	110	0.2968
bta05222	Small cell lung cancer	XLOC_005665;XLOC_023941	2	110	0.3114
bta03060	Protein export	XLOC_023666	1	110	0.3172
bta04920	Adipocytokine signaling pathway	XLOC_011157	1	110	0.3172
bta04970	Salivary secretion	XLOC_024183	1	110	0.3370
bta04972	Pancreatic secretion	XLOC_023853	1	110	0.3370
bta05323	Rheumatoid arthritis	XLOC_001733;XLOC_011014	2	110	0.3435
bta05150	Staphylococcus aureus infection	XLOC_007669	1	110	0.3562
bta04142	Lysosome	XLOC_003080;XLOC_023509;XLOC_024165	3	110	0.3771
bta04330	Notch signaling pathway	XLOC_023581	1	110	0.3930
bta04710	Circadian rhythm - mammal	XLOC_011222	1	110	0.4106
bta05221	Acute myeloid leukemia	XLOC_023653	1	110	0.4106
bta05416	Viral myocarditis	XLOC_015580;XLOC_024072	2	110	0.4163
bta00230	Purine metabolism	XLOC_023788	1	110	0.4277
bta05220	Chronic myeloid leukemia	XLOC_022857	1	110	0.4277
bta05340	Primary immunodeficiency	XLOC_005156	1	110	0.4277
bta04360	Axon guidance	XLOC_002260;XLOC_023558	2	110	0.4364
bta04130	SNARE interactions in vesicular transport	XLOC_001463	1	110	0.4913
bta04270	Vascular smooth muscle contraction	XLOC_021577	1	110	0.5061
bta05014	Amyotrophic lateral sclerosis	XLOC_023512	1	110	0.5061
bta05146	Amoebiasis	XLOC_010535	1	110	0.5205
bta04610	Complement and coagulation cascades	XLOC_019618	1	110	0.5480
bta05322	Systemic lupus erythematosus	XLOC_015255;XLOC_015261	2	110	0.5570
bta04145	Phagosome	XLOC_023801	1	110	0.6100
bta04914	Progesterone-mediated oocyte maturation	XLOC_023568	1	110	0.6100
bta05016	Huntington's disease	XLOC_023992;XLOC_024239;XLOC_025657	3	110	0.6526
bta04062	Chemokine signaling pathway	XLOC_023720	1	110	0.7012
bta03010	Ribosome	XLOC_002615;XLOC_028013	2	110	0.7462
bta03008	Ribosome biogenesis in eukaryotes	XLOC_023915	1	110	0.7499
bta04141	Protein processing in endoplasmic reticulum	XLOC_003872;XLOC_023448	2	110	0.7623
bta05160	Hepatitis C	XLOC_010800	1	110	0.7779
bta03040	Spliceosome	XLOC_012182;XLOC_023994	2	110	0.7872
bta04146	Peroxisome	XLOC_025902	1	110	0.7968
bta04080	Neuroactive ligand-receptor interaction	XLOC_021530	1	110	0.8914
bta03013	RNA transport	XLOC_003001	1	110	0.9119
bta00010	Glycolysis / Gluconeogenesis	NA	0	110	1.0000
bta00030	Pentose phosphate pathway	NA	0	110	1.0000
bta00051	Fructose and mannose metabolism	NA	0	110	1.0000
bta00071	Fatty acid metabolism	NA	0	110	1.0000
bta00100	Steroid biosynthesis	NA	0	110	1.0000
bta00120	Primary bile acid biosynthesis	NA	0	110	1.0000
bta00140	Steroid hormone biosynthesis	NA	0	110	1.0000
bta00240	Pyrimidine metabolism	NA	0	110	1.0000

bta00250	Alanine, aspartate and glutamate metabolism	NA	0	110	1.0000
bta00260	Glycine, serine and threonine metabolism	NA	0	110	1.0000
bta00270	Cysteine and methionine metabolism	NA	0	110	1.0000
bta00310	Lysine degradation	NA	0	110	1.0000
bta00340	Histidine metabolism	NA	0	110	1.0000
bta00350	Tyrosine metabolism	NA	0	110	1.0000
bta00360	Phenylalanine metabolism	NA	0	110	1.0000
bta00380	Tryptophan metabolism	NA	0	110	1.0000
bta00450	Selenocompound metabolism	NA	0	110	1.0000
bta00480	Glutathione metabolism	NA	0	110	1.0000
bta00500	Starch and sucrose metabolism	NA	0	110	1.0000
bta00510	N-Glycan biosynthesis	NA	0	110	1.0000
bta00511	Other glycan degradation	NA	0	110	1.0000
bta00514	Other types of O-glycan biosynthesis	NA	0	110	1.0000
bta00532	Glycosaminoglycan biosynthesis - chondroitin sulfate	NA	0	110	1.0000
bta00533	Glycosaminoglycan biosynthesis - keratan sulfate	NA	0	110	1.0000
bta00534	Glycosaminoglycan biosynthesis - heparan sulfate	NA	0	110	1.0000
bta00562	Inositol phosphate metabolism	NA	0	110	1.0000
bta00564	Glycerophospholipid metabolism	NA	0	110	1.0000
bta00565	Ether lipid metabolism	NA	0	110	1.0000
bta00590	Arachidonic acid metabolism	NA	0	110	1.0000
bta00600	Sphingolipid metabolism	NA	0	110	1.0000
bta00601	Glycosphingolipid biosynthesis - lacto and neolacto series	NA	0	110	1.0000
bta00650	Butanoate metabolism	NA	0	110	1.0000
bta00740	Riboflavin metabolism	NA	0	110	1.0000
bta00770	Pantothenate and CoA biosynthesis	NA	0	110	1.0000
bta00790	Folate biosynthesis	NA	0	110	1.0000
bta00830	Retinol metabolism	NA	0	110	1.0000
bta00860	Porphyrin and chlorophyll metabolism	NA	0	110	1.0000
bta00900	Terpenoid backbone biosynthesis	NA	0	110	1.0000
bta00910	Nitrogen metabolism	NA	0	110	1.0000
bta00920	Sulfur metabolism	NA	0	110	1.0000
bta00970	Aminoacyl-tRNA biosynthesis	NA	0	110	1.0000
bta00980	Metabolism of xenobiotics by cytochrome P450	NA	0	110	1.0000
bta00982	Drug metabolism - cytochrome P450	NA	0	110	1.0000
bta00983	Drug metabolism - other enzymes	NA	0	110	1.0000
bta01040	Biosynthesis of unsaturated fatty acids	NA	0	110	1.0000
bta02010	ABC transporters	NA	0	110	1.0000
bta03015	mRNA surveillance pathway	NA	0	110	1.0000
bta03018	RNA degradation	NA	0	110	1.0000
bta03020	RNA polymerase	NA	0	110	1.0000
bta03022	Basal transcription factors	NA	0	110	1.0000
bta03030	DNA replication	NA	0	110	1.0000
bta03050	Proteasome	NA	0	110	1.0000
bta03410	Base excision repair	NA	0	110	1.0000
bta03420	Nucleotide excision repair	NA	0	110	1.0000
bta03430	Mismatch repair	NA	0	110	1.0000
bta03440	Homologous recombination	NA	0	110	1.0000
bta03450	Non-homologous end-joining	NA	0	110	1.0000
bta04012	ErbB signaling pathway	NA	0	110	1.0000
bta04020	Calcium signaling pathway	NA	0	110	1.0000
bta04060	Cytokine-cytokine receptor interaction	NA	0	110	1.0000
bta04110	Cell cycle	NA	0	110	1.0000
bta04114	Oocyte meiosis	NA	0	110	1.0000

bta04122	Sulfur relay system	NA	0	110	1.0000
bta04140	Regulation of autophagy	NA	0	110	1.0000
bta04144	Endocytosis	NA	0	110	1.0000
bta04150	mTOR signaling pathway	NA	0	110	1.0000
bta04260	Cardiac muscle contraction	NA	0	110	1.0000
bta04310	Wnt signaling pathway	NA	0	110	1.0000
bta04320	Dorso-ventral axis formation	NA	0	110	1.0000
bta04370	VEGF signaling pathway	NA	0	110	1.0000
bta04514	Cell adhesion molecules	NA	0	110	1.0000
bta04520	Adherens junction	NA	0	110	1.0000
bta04540	Gap junction	NA	0	110	1.0000
bta04612	Antigen processing and presentation	NA	0	110	1.0000
bta04614	Renin-angiotensin system	NA	0	110	1.0000
bta04621	NOD-like receptor signaling pathway	NA	0	110	1.0000
bta04622	RIG-I-like receptor signaling pathway	NA	0	110	1.0000
bta04623	Cytosolic DNA-sensing pathway	NA	0	110	1.0000
bta04630	Jak-STAT signaling pathway	NA	0	110	1.0000
bta04640	Hematopoietic cell lineage	NA	0	110	1.0000
bta04650	Natural killer cell mediated cytotoxicity	NA	0	110	1.0000
bta04660	T cell receptor signaling pathway	NA	0	110	1.0000
bta04662	B cell receptor signaling pathway	NA	0	110	1.0000
bta04664	Fc epsilon RI signaling pathway	NA	0	110	1.0000
bta04666	Fc gamma R-mediated phagocytosis	NA	0	110	1.0000
bta04670	Leukocyte transendothelial migration	NA	0	110	1.0000
bta04672	Intestinal immune network for IgA production	NA	0	110	1.0000
bta04720	Long-term potentiation	NA	0	110	1.0000
bta04722	Neurotrophin signaling pathway	NA	0	110	1.0000
bta04730	Long-term depression	NA	0	110	1.0000
bta04740	Olfactory transduction	NA	0	110	1.0000
bta04742	Taste transduction	NA	0	110	1.0000
bta04744	Phototransduction	NA	0	110	1.0000
bta04810	Regulation of actin cytoskeleton	NA	0	110	1.0000
bta04912	GnRH signaling pathway	NA	0	110	1.0000
bta04916	Melanogenesis	NA	0	110	1.0000
bta04930	Type II diabetes mellitus	NA	0	110	1.0000
bta04940	Type I diabetes mellitus	NA	0	110	1.0000
bta04950	Maturity onset diabetes of the young	NA	0	110	1.0000
bta04962	Vasopressin-regulated water reabsorption	NA	0	110	1.0000
bta04964	Proximal tubule bicarbonate reclamation	NA	0	110	1.0000
bta04966	Collecting duct acid secretion	NA	0	110	1.0000
bta04971	Gastric acid secretion	NA	0	110	1.0000
bta04973	Carbohydrate digestion and absorption	NA	0	110	1.0000
bta04975	Fat digestion and absorption	NA	0	110	1.0000
bta04976	Bile secretion	NA	0	110	1.0000
bta04977	Vitamin digestion and absorption	NA	0	110	1.0000
bta05010	Alzheimer's disease	NA	0	110	1.0000
bta05012	Parkinson's disease	NA	0	110	1.0000
bta05020	Prion diseases	NA	0	110	1.0000
bta05100	Bacterial invasion of epithelial cells	NA	0	110	1.0000
bta05140	Leishmaniasis	NA	0	110	1.0000
bta05143	African trypanosomiasis	NA	0	110	1.0000
bta05144	Malaria	NA	0	110	1.0000
bta05200	Pathways in cancer	NA	0	110	1.0000
bta05210	Colorectal cancer	NA	0	110	1.0000
bta05211	Renal cell carcinoma	NA	0	110	1.0000
bta05212	Pancreatic cancer	NA	0	110	1.0000
bta05213	Endometrial cancer	NA	0	110	1.0000
bta05215	Prostate cancer	NA	0	110	1.0000
bta05216	Thyroid cancer	NA	0	110	1.0000

bta05217	Basal cell carcinoma	NA	0	110	1.0000
bta05219	Bladder cancer	NA	0	110	1.0000
bta05223	Non-small cell lung cancer	NA	0	110	1.0000
bta05310	Asthma	NA	0	110	1.0000
bta05330	Allograft rejection	NA	0	110	1.0000
bta05332	Graft-versus-host disease	NA	0	110	1.0000
bta05412	Arrhythmogenic right ventricular cardiomyopathy	NA	0	110	1.0000

No. of mapping ST indicated the number of the significantly expressed transcripts mapping to one certain kegg pathway by the KEGG analysis; No. of mapping TT indicated the number of the total significantly expressed transcripts mapping by the KEGG analysis. NA indicated no significantly expressed transcripts were mapped to some KEGG pathways.

Table S10 KEGG pathway enrichment of the differentially expressed proteins in mammary glands between RS- and AH-fed dairy cows

KEGG pathway id	KEGG pathway description	Protein accession	No. of mapping SP	No. of mapping TP	P-value of Fisher'exact test
bta04512	ECM-receptor interaction	IPI00694504;IPI00697595;IPI00707359;IPI00707857;IPI00708244;IPI00708824;IPI00710204;IPI00712524;IPI00713573;IPI00714673;IPI00715486;IPI00731432;IPI00826022;IPI00837992	14	227	0.0003
bta03010	Ribosome	IPI00687634;IPI00687662;IPI00688727;IPI00689565;IPI00694444;IPI00694565;IPI00694688;IPI00698077;IPI00699827;IPI00702948;IPI00705739;IPI00707386;IPI00707537;IPI00708210;IPI00709409;IPI00710859;IPI00712384;IPI00713536;IPI00715091;IPI00715868;IPI00716860;IPI00717048;IPI00718379	23	227	0.0005
bta05414	Dilated cardiomyopathy	IPI00688489;IPI00689750;IPI00697595;IPI00698900;IPI00703268;IPI00707359;IPI00714405;IPI00727017;IPI00905865	9	227	0.0038
bta05410	Hypertrophic cardiomyopathy （HCM)	IPI00688489;IPI00689750;IPI00697595;IPI00698900;IPI00703268;IPI00707359;IPI00714405;IPI00727017;IPI00905865	9	227	0.0049
bta04974	Protein digestion and absorption	IPI00705159;IPI00707857;IPI00708244;IPI00712524;IPI00713573;IPI00717116;IPI00731432;IPI00826022;IPI00837992	9	227	0.0079
bta04510	Focal adhesion	IPI00694504;IPI00694758;IPI00697595;IPI00698900;IPI00699798;IPI00707857;IPI00708244;IPI00709876;IPI00712524;IPI00713573;IPI00714673;IPI00715486;IPI00731432;IPI00826022;IPI00837992;IPI00906031	16	227	0.0433
bta04261	Adrenergic signaling in cardiomyocytes	IPI00688489;IPI00688651;IPI00695508;IPI00699798;IPI00703268;IPI00705159;IPI00714405;IPI00727017;IPI00906031;IPI01017588	10	227	0.0459
bta05031	Amphetamine addiction	IPI00688651;IPI00695508;IPI00698059;IPI00699798;IPI00906031	5	227	0.0540
bta05412	Arrhythmogenic right ventricular cardiomyopathy （ARVC)	IPI00689750;IPI00697595;IPI00698900;IPI00707359;IPI00708070;IPI00905865	6	227	0.0682

bta05146	Amoebiasis	IPI00693824;IPI00694198;IPI00707857;IPI00708244;IPI00712524;IPI00714673;IPI00715486;IPI00731432;IPI00826022;IPI00876781	10	227	0.0742
bta04975	Fat digestion and absorption	IPI00695965;IPI00706879;IPI00710204	3	227	0.0818
bta05206	MicroRNAs in cancer	IPI00687375;IPI00689165;IPI00689228;IPI00690309;IPI00694504;IPI00694758;IPI00713642;IPI00715059	8	227	0.0844
bta05034	Alcoholism	IPI00695508;IPI00698059;IPI00698339;IPI00699798;IPI00705000;IPI00712399;IPI00716843;IPI00906031	8	227	0.0952
bta00564	Glycerophospholipid metabolism	IPI00687661;IPI00703084;IPI00706879;IPI00839150;IPI00842934	5	227	0.0956
bta04740	Olfactory transduction	IPI00688651;IPI00695508;IPI00703776	3	227	0.1069
bta00534	Glycosami0.06glycan biosynthesis - heparan sulfate / heparin	IPI00699039;IPI00699817	2	227	0.1113
bta04744	Phototransduction	IPI00695508;IPI00703776	2	227	0.1113
bta04260	Cardiac muscle contraction	IPI00688489;IPI00694849;IPI00703268;IPI00705159;IPI00714405;IPI00727017	6	227	0.1227
bta00770	Pantothenate and CoA biosynthesis	IPI00703753;IPI00704332;IPI00715066	3	227	0.1346
bta04970	Salivary secretion	IPI00695508;IPI00696116;IPI00705159;IPI00710920;IPI00716157	5	227	0.1493
bta05205	Proteoglycans in cancer	IPI00687375;IPI00688608;IPI00688651;IPI00690309;IPI00695042;IPI00698900;IPI00699798;IPI00702948;IPI00709876;IPI00713642;IPI00714673;IPI00716158;IPI00906031	13	227	0.1497
bta00120	Primary bile acid biosynthesis	IPI00686601;IPI00702251	2	227	0.1541
bta04960	Aldosterone-regulated sodium reabsorption	IPI00705159;IPI00715059;IPI00715354	3	227	0.1644
bta04976	Bile secretion	IPI00705159;IPI00712596;IPI00714656	3	227	0.1644
bta04911	Insulin secretion	IPI00688651;IPI00696116;IPI00705159;IPI00714656	4	227	0.1835

bta04151	PI3K-Akt signaling pathway	IPI00690309;IPI00694504;IPI00697595;IPI00702948;IPI00707857;IPI00708244;IPI00712524;IPI00713573;IPI00714673;IPI00715059;IPI00715486;IPI00731432;IPI00826022;IPI00837992;IPI01017588	15	227	0.1940
bta00790	Folate biosynthesis	IPI00702768;IPI00742596	2	227	0.1995
bta00561	Glycerolipid metabolism	IPI00700920;IPI00703084;IPI00706879;IPI00842934	4	227	0.2093
bta04962	Vasopressin-regulated water reabsorption	IPI00693824;IPI00694198;IPI00696116;IPI00714660;IPI00718152	5	227	0.2131
bta05100	Bacterial invasion of epithelial cells	IPI00698900;IPI00703380;IPI00707718;IPI00708767;IPI00709876;IPI00710408;IPI00711750;IPI00714673	8	227	0.2232
bta00290	Valine, leucine and isoleucine biosynthesis	IPI00715066	1	227	0.2246
bta00430	Taurine and hypotaurine metabolism	IPI00705565	1	227	0.2246
bta04966	Collecting duct acid secretion	IPI00692186;IPI00699372;IPI00716195	3	227	0.2288
bta00130	Ubiquine and other terpeid-quine biosynthesis	IPI00685678;IPI00695952	2	227	0.2461
bta04020	Calcium signaling pathway	IPI00688651;IPI00693452;IPI00695508;IPI00703129;IPI00705378;IPI00706314	6	227	0.2539
bta00480	Glutathione metabolism	IPI00686183;IPI00702151;IPI00705565;IPI00711759;IPI01028182	5	227	0.2597
bta00980	Metabolism of xe0.06biotics by cytochrome P450	IPI00702151;IPI00712596;IPI01028182	3	227	0.2626
bta00982	Drug metabolism - cytochrome P450	IPI00698059;IPI00702151;IPI01028182	3	227	0.2626
bta05204	Chemical carci0.06genesis	IPI00702151;IPI00712596;IPI01028182	3	227	0.2626
bta05150	Staphylococcus aureus infection	IPI00692588;IPI00694204;IPI00708921;IPI00721270	4	227	0.2916
bta05416	Viral myocarditis	IPI00698900;IPI00707359;IPI00709876	3	227	0.2969
bta00460	Cyaami acid metabolism	IPI00705565	1	227	0.3173
bta00601	Glycosphingolipid biosynthesis - lacto and neolacto series	IPI00827121	1	227	0.3173
bta00590	Arachidonic acid metabolism	IPI00686183;IPI00705565;IPI00714621	3	227	0.3315
bta04310	Wnt signaling pathway	IPI00688651;IPI00690446;IPI00699355;IPI00708311;IPI00712288	5	227	0.3332

bta00040	Pentose and glucuronate interconversions	IPI00700920;IPI00712671	2	227	0.3395
bta04978	Mineral absorption	IPI00687842;IPI00705159	2	227	0.3395
bta04728	Dopaminergic synapse	IPI00688651;IPI00695508;IPI00698059;IPI00699798;IPI00716843;IPI00906031;IPI01017588	7	227	0.3468
bta04720	Long-term potentiation	IPI00688651;IPI00695508;IPI00699798;IPI00906031	4	227	0.3487
bta05166	HTLV-I infection	IPI00691963;IPI00695042;IPI00701223;IPI00703129;IPI00705378;IPI00705941;IPI00706314;IPI00714656	8	227	0.3505
bta00410	beta-Alanine metabolism	IPI00694430;IPI00702650;IPI00711759	3	227	0.3660
bta04640	Hematopoietic cell lineage	IPI00697595;IPI00710204	2	227	0.3850
bta05144	Malaria	IPI00685617;IPI00710204	2	227	0.3850
bta00740	Riboflavin metabolism	IPI00703753	1	227	0.3990
bta00780	Biotin metabolism	IPI00727591	1	227	0.3990
bta00270	Cysteine and methionine metabolism	IPI00694739;IPI00711759;IPI00883375	3	227	0.4003
bta04961	Endocrine and other factor-regulated calcium reabsorption	IPI00703547;IPI00705159;IPI00710408	3	227	0.4003
bta04721	Synaptic vesicle cycle	IPI00696116;IPI00699372;IPI00710408;IPI00716195	4	227	0.4060
bta04920	Adipocytokine signaling pathway	IPI00690309;IPI00710204;IPI00714656;IPI00715059	4	227	0.4060
bta00900	Terpe0.06id backbone biosynthesis	IPI00690789;IPI00944429	2	227	0.4292
bta00650	Buta0.06ate metabolism	IPI00691649;IPI00694312;IPI00702650	3	227	0.4340
bta04971	Gastric acid secretion	IPI00688651;IPI00695508;IPI00705159	3	227	0.4340
bta00051	Fructose and man0.06se metabolism	IPI00689323;IPI00700789;IPI00700920;IPI00706942	4	227	0.4343
bta00640	Propionate metabolism	IPI00694430;IPI00696912;IPI00702650;IPI00708438	4	227	0.4343
bta04390	Hippo signaling pathway	IPI00698900;IPI00699798;IPI00710274;IPI00906031	4	227	0.4343
bta04144	Endocytosis	IPI00688451;IPI00693824;IPI00694198;IPI00703776;IPI00705933;IPI00709876;IPI00710408;IPI00718152;IPI00718381;IPI00867339	10	227	0.4570
bta00330	Arginine and proline metabolism	IPI00698059;IPI00711759;IPI00760457;IPI00838420	4	227	0.4623
bta05322	Systemic lupus erythematosus	IPI00698339;IPI00705000;IPI00712399;IPI00876781	4	227	0.4623

bta00562	I0.06sitol phosphate metabolism	IPI00687661;IPI00692819;IPI00706942	3	227	0.4670
bta04514	Cell adhesion molecules （CAMs)	IPI00687372;IPI00713313;IPI00713979	3	227	0.4670
bta00531	Glycosami0.06glycan degradation	IPI00706203	1	227	0.4709
bta04977	Vitamin digestion and absorption	IPI00695965	1	227	0.4709
bta04145	Phagosome	IPI00690160;IPI00691963;IPI00693824;IPI00694198;IPI00698900;IPI00699372;IPI00710204;IPI00716195	8	227	0.4846
bta04110	Cell cycle	IPI00701223;IPI00715354;IPI00734257	3	227	0.4991
bta04114	Oocyte meiosis	IPI00688651;IPI00695508;IPI00699798;IPI00906031;IPI01017588	5	227	0.5063
bta05152	Tuberculosis	IPI00688651;IPI00690160;IPI00693824;IPI00694198;IPI00695508;IPI00699372;IPI00716195	7	227	0.5109
bta05164	Influenza A	IPI00698900;IPI00701747;IPI00703129;IPI00705378;IPI00706314;IPI00714269;IPI00867339	7	227	0.5109
bta04115	p53 signaling pathway	IPI00709542;IPI00715354	2	227	0.5120
bta05134	Legionellosis	IPI00712775;IPI00727050;IPI00829520;IPI00867339	4	227	0.5166
bta04916	Mela0.06genesis	IPI00688651;IPI00695508;IPI00716843	3	227	0.5302
bta05214	Glioma	IPI00688651;IPI00690309;IPI00695508	3	227	0.5302
bta00053	Ascorbate and aldarate metabolism	IPI00712671	1	227	0.5342
bta00603	Glycosphingolipid biosynthesis - globo series	IPI00706203	1	227	0.5342
bta00604	Glycosphingolipid biosynthesis - ganglio series	IPI00706203	1	227	0.5342
bta00910	Nitrogen metabolism	IPI00760457	1	227	0.5342
bta03440	Homologous recombination	IPI00695890	1	227	0.5342
bta00380	Tryptophan metabolism	IPI00698059;IPI00702650	2	227	0.5504
bta04930	Type II diabetes mellitus	IPI00690309;IPI00715059	2	227	0.5504
bta04130	SNARE interactions in vesicular transport	IPI00696116;IPI00713460;IPI00714660	3	227	0.5602
bta04150	mTOR signaling pathway	IPI00690309;IPI00702948;IPI00715059	3	227	0.5602
bta04146	Peroxisome	IPI00686601;IPI00692468;IPI00694430;IPI00702251	4	227	0.5683
bta04972	Pancreatic secretion	IPI00705159;IPI00710920	2	227	0.5866
bta05323	Rheumatoid arthritis	IPI00699372;IPI00716195	2	227	0.5866
bta03008	Ribosome biogenesis in eukaryotes	IPI00705941;IPI00708018;IPI00852474	3	227	0.5890
bta04070	Phosphatidylisitol signaling system	IPI00687661;IPI00692819;IPI00695508	3	227	0.5890

bta00072	Synthesis and degradation of ketone bodies	IPI00694312	1	227	0.5900
bta00360	Phenylalanine metabolism	IPI00698059	1	227	0.5900
bta00830	Retil metabolism	IPI00692627	1	227	0.5900
bta05340	Primary immudeficiency	IPI00693452	1	227	0.5900
bta04810	Regulation of actin cytoskeleton	IPI00694758;IPI00695042;IPI00697595;IPI00698900;IPI00699798;IPI00708767;IPI00711750;IPI00714673;IPI00906031	9	227	0.6151
bta04612	Antigen processing and presentation	IPI00689325;IPI00691963;IPI00867339	3	227	0.6166
bta05222	Small cell lung cancer	IPI00712524;IPI00714673;IPI00715486	3	227	0.6166
bta04142	Lysosome	IPI00698823;IPI00699372;IPI00706203;IPI00710408;IPI00716195;IPI00718311	6	227	0.6212
bta00511	Other glycan degradation	IPI00706203	1	227	0.6392
bta01040	Biosynthesis of unsaturated fatty acids	IPI00702650	1	227	0.6392
bta03030	DNA replication	IPI00695890	1	227	0.6392
bta03430	Mismatch repair	IPI00695890	1	227	0.6392
bta04964	Proximal tubule bicarbonate reclamation	IPI00705159	1	227	0.6392
bta03015	mRNA surveillance pathway	IPI00688446;IPI00690232;IPI00699798;IPI00906031;IPI01017588	5	227	0.6392
bta03050	Proteasome	IPI00695176;IPI00697757;IPI00701790;IPI00707875	4	227	0.6397
bta04270	Vascular smooth muscle contraction	IPI00695508;IPI00699798;IPI00906031	3	227	0.6430
bta00260	Glycine, serine and threonine metabolism	IPI00698059;IPI00698589	2	227	0.6525
bta00310	Lysine degradation	IPI00702650;IPI00871114	2	227	0.6525
bta05032	Morphine addiction	IPI00703776;IPI00716843	2	227	0.6525
bta04713	Circadian entrainment	IPI00688651;IPI00695508;IPI00716843	3	227	0.6680
bta04727	GABAergic synapse	IPI00716843;IPI00760457	2	227	0.6822
bta00340	Histidine metabolism	IPI00698059	1	227	0.6824
bta04330	0.06tch signaling pathway	IPI00690446	1	227	0.6824
bta04610	Complement and coagulation cascades	IPI00695489;IPI00701166;IPI00871133;IPI00876781	4	227	0.6828
bta03320	PPAR signaling pathway	IPI00686601;IPI00699355;IPI00710204	3	227	0.6917
bta01230	Biosynthesis of ami0.06 acids	IPI00698589;IPI00706942;IPI00715066;IPI00760457;IPI00904104	5	227	0.6967
bta00500	Starch and sucrose metabolism	IPI00703753;IPI00712671	2	227	0.7097
bta04918	Thyroid hormone synthesis	IPI00686183;IPI00705159	2	227	0.7097
bta00062	Fatty acid elongation	IPI00702650	1	227	0.7205
bta00670	One carbon pool by folate	IPI00742596	1	227	0.7205
bta00920	Sulfur metabolism	IPI00730144	1	227	0.7205

bta04973	Carbohydrate digestion and absorption	IPI00705159	1	227	0.7205
bta05216	Thyroid cancer	IPI00714405	1	227	0.7205
bta00970	Amiacyl-tRNA biosynthesis	IPI00689365;IPI00708246;IPI00867017	3	227	0.7354
bta04726	Serotonergic synapse	IPI00685510;IPI00698059;IPI00716843	3	227	0.7354
bta04910	Insulin signaling pathway	IPI00690309;IPI00695508;IPI00699798;IPI00702948;IPI00715059;IPI00906031	6	227	0.7407
bta04014	Ras signaling pathway	IPI00693824;IPI00694198;IPI00694758;IPI00695042;IPI00695508	5	227	0.7476
bta00350	Tyrosine metabolism	IPI00698059	1	227	0.7541
bta00450	Selecompound metabolism	IPI00730144	1	227	0.7541
bta00760	Nicotinate and nicotinamide metabolism	IPI00703753	1	227	0.7541
bta00983	Drug metabolism - other enzymes	IPI00686225	1	227	0.7541
bta04066	HIF-1 signaling pathway	IPI00688651;IPI00690309;IPI00702948;IPI00714656	4	227	0.7580
bta04012	ErbB signaling pathway	IPI00688651;IPI00690309;IPI00694758	3	227	0.7740
bta05221	Acute myeloid leukemia	IPI00690309;IPI00699355	2	227	0.7807
bta00860	Porphyrin and chlorophyll metabolism	IPI00687842	1	227	0.7836
bta01210	2-Oxocarboxylic acid metabolism	IPI00715066	1	227	0.7836
bta05030	Cocaine addiction	IPI00698059	1	227	0.7836
bta04015	Rap1 signaling pathway	IPI00695042;IPI00695508;IPI00698900;IPI00716843	4	227	0.7900
bta04530	Tight junction	IPI00692417;IPI00695042;IPI00698900;IPI00713313;IPI00716830	5	227	0.7920
bta00620	Pyruvate metabolism	IPI00696912;IPI00700920	2	227	0.8008
bta00520	Ami sugar and nucleotide sugar metabolism	IPI00689323;IPI00706203;IPI00712671	3	227	0.8079
bta05200	Pathways in cancer	IPI00690309;IPI00690446;IPI00699355;IPI00712524;IPI00714405;IPI00714656;IPI00714673;IPI00715486	8	227	0.8161
bta03018	RNA degradation	IPI00704902;IPI01017508	2	227	0.8192
bta04666	Fc gamma R-mediated phagocytosis	IPI00688451;IPI00708767;IPI00711750	3	227	0.8232
bta04350	TGF-beta signaling pathway	IPI00688608	1	227	0.8325
bta04724	Glutamatergic synapse	IPI00716843;IPI00760457	2	227	0.8360
bta04912	GnRH signaling pathway	IPI00688651;IPI00695508	2	227	0.8360
bta05211	Renal cell carcima	IPI00694758;IPI00714656	2	227	0.8360
bta00230	Purine metabolism	IPI00686225;IPI00703753;IPI00716555;IPI00726650;IPI00730144	5	227	0.8522

bta05012	Parkinson's disease	IPI00686966;IPI00694849;IPI00703129;IPI00705378;IPI00706314;IPI00711233;IPI00712677	7	227	0.8612
bta04915	Estrogen signaling pathway	IPI00695508;IPI00716843;IPI00867339	3	227	0.8630
bta05145	Toxoplasmosis	IPI00715486;IPI00716843;IPI00867339	3	227	0.8630
bta05014	Amyotrophic lateral sclerosis （ALS)	IPI00693824	1	227	0.8704
bta00010	Glycolysis / Gluconeogenesis	IPI00696912;IPI00698589;IPI00706942	3	227	0.8744
bta04120	Ubiquitin mediated proteolysis	IPI00697917;IPI00704041;IPI00718678	3	227	0.8744
bta05132	Salmonella infection	IPI00698900;IPI00708767;IPI00711750	3	227	0.8744
bta04141	Protein processing in endoplasmic reticulum	IPI00689325;IPI00690581;IPI00691963;IPI00692963;IPI00696616;IPI00699107;IPI00700547;IPI00702891;IPI00867339	9	227	0.8792
bta00250	Alanine, aspartate and glutamate metabolism	IPI00760457	1	227	0.8860
bta00630	Glyoxylate and dicarboxylate metabolism	IPI00760457	1	227	0.8860
bta01200	Carbon metabolism	IPI00696912;IPI00698589;IPI00706942;IPI00708438;IPI00904104	5	227	0.8893
bta04725	Cholinergic synapse	IPI00688651;IPI00716843	2	227	0.8902
bta04670	Leukocyte transendothelial migration	IPI00698900;IPI00713313;IPI00713979	3	227	0.8947
bta04722	Neurotrophin signaling pathway	IPI00688651;IPI00695508;IPI00715059	3	227	0.8947
bta01100	Metabolic pathways	IPI00686225;IPI00686601;IPI00687661;IPI00688977;IPI00689323;IPI00690789;IPI00692627;IPI00692819;IPI00694312;IPI00694430;IPI00694739;IPI00694849;IPI00695952;IPI00696912;IPI00698059;IPI00698589;IPI00699372;IPI00699817;IPI00700920;IPI00702251;IPI00702650;IPI00702768;IPI00703084;IPI00703753;IPI00704332;IPI00705565;IPI00706203;IPI00706879;IPI00706942;IPI00708246;IPI00708438;IPI00711233;IPI00711759;IPI00712671;IPI00712677;IPI00714621;IPI00715066;IPI00716195;IPI00716555;IPI00726650;IPI00727591;IPI00730144;IPI00742596;IPI00760457;IPI00827121;IPI00838420;IPI00842934;IPI00871114;IPI00883375;IPI00904104;IPI00944429	51	227	0.8971

bta03013	RNA transport	IPI00689226;IPI00690232;IPI00693324;IPI00701747;IPI00702620;IPI00705941;IPI00712775	7	227	0.8995
bta03040	Spliceosome	IPI00686595;IPI00687395;IPI00688337;IPI00690232;IPI00695600;IPI00717759;IPI00867339	7	227	0.8995
bta03060	Protein export	IPI00685804	1	227	0.8997
bta05202	Transcriptional misregulation in cancer	IPI00705463	1	227	0.8997
bta05016	Huntington's disease	IPI00692468;IPI00694849;IPI00703129;IPI00705378;IPI00706314;IPI00710408;IPI00711233;IPI00712677	8	227	0.9051
bta00030	Pentose phosphate pathway	IPI00904104	1	227	0.9118
bta00052	Galactose metabolism	IPI00700920	1	227	0.9118
bta05020	Prion diseases	IPI00876781	1	227	0.9225
bta04723	Retrograde endocannabid signaling	IPI00716843	1	227	0.9318
bta04210	Apoptosis	IPI00704835;IPI00709124	2	227	0.9347
bta05142	Chagas disease (American trypasomiasis)	IPI00691963;IPI00716843	2	227	0.9412
bta00280	Valine, leucine and isoleucine degradation	IPI00702650;IPI00715066	2	227	0.9472
bta05220	Chronic myeloid leukemia	IPI00690446	1	227	0.9473
bta00020	Citrate cycle (TCA cycle)	IPI00708438	1	227	0.9537
bta04662	B cell receptor signaling pathway	IPI00685617	1	227	0.9537
bta05168	Herpes simplex infection	IPI00699798;IPI00701698;IPI00906031	3	227	0.9542
bta04360	Axon guidance	IPI00694758	1	227	0.9593
bta04520	Adherens junction	IPI00698900	1	227	0.9593
bta04730	Long-term depression	IPI00716843	1	227	0.9593
bta05133	Pertussis	IPI00695508	1	227	0.9593
bta05215	Prostate cancer	IPI00690309	1	227	0.9593
bta00071	Fatty acid degradation	IPI00702650	1	227	0.9642
bta04380	Osteoclast differentiation	IPI00692676	1	227	0.9642
bta04660	T cell receptor signaling pathway	IPI00694758	1	227	0.9642
bta00190	Oxidative phosphorylation	IPI00694849;IPI00699372;IPI00711233;IPI00712677;IPI00716195	5	227	0.9663
bta01212	Fatty acid metabolism	IPI00702650	1	227	0.9685
bta04010	MAPK signaling pathway	IPI00695042;IPI00703776;IPI00867339	3	227	0.9829
bta05162	Measles	IPI00867339	1	227	0.9835
bta05169	Epstein-Barr virus infection	IPI00689228;IPI00701790;IPI00705941;IPI00867339	4	227	0.9849
bta04932	alcoholic fatty liver disease FLD	IPI00694849;IPI00711233;IPI00712677;IPI00715059	4	227	0.9897
bta05010	Alzheimer's disease	IPI00685510;IPI00694849;IPI00695508;IPI00711233;IPI00712677	5	227	0.9918
bta05160	Hepatitis C	IPI00685617	1	227	0.9924
bta04062	Chemokine signaling pathway	IPI00703776	1	227	0.9949
bta05203	Viral carcigenesis	IPI00705000;IPI00716195	2	227	0.9978
bta00061	Fatty acid biosynthesis	NA	0	227	1.0000
bta00100	Steroid biosynthesis	NA	0	227	1.0000
bta00140	Steroid hormone biosynthesis	NA	0	227	1.0000
bta00232	Caffeine metabolism	NA	0	227	1.0000
bta00240	Pyrimidine metabolism	NA	0	227	1.0000

bta00300	Lysine biosynthesis	NA	0	227	1.0000
bta00400	Phenylalanine, tyrosine and tryptophan biosynthesis	NA	0	227	1.0000
bta00471	D-Glutamine and D-glutamate metabolism	NA	0	227	1.0000
bta00510	N-Glycan biosynthesis	NA	0	227	1.0000
bta00512	Mucin type O-Glycan biosynthesis	NA	0	227	1.0000
bta00514	Other types of O-glycan biosynthesis	NA	0	227	1.0000
bta00524	Butirosin and neomycin biosynthesis	NA	0	227	1.0000
bta00533	Glycosamiglycan biosynthesis - keratan sulfate	NA	0	227	1.0000
bta00563	Glycosylphosphatidylisitol（GPI)-anchor biosynthesis	NA	0	227	1.0000
bta00565	Ether lipid metabolism	NA	0	227	1.0000
bta00591	Lileic acid metabolism	NA	0	227	1.0000
bta00592	alpha-Lilenic acid metabolism	NA	0	227	1.0000
bta00600	Sphingolipid metabolism	NA	0	227	1.0000
bta00730	Thiamine metabolism	NA	0	227	1.0000
bta00750	Vitamin B6 metabolism	NA	0	227	1.0000
bta00785	Lipoic acid metabolism	NA	0	227	1.0000
bta01220	Degradation of aromatic compounds	NA	0	227	1.0000
bta02010	ABC transporters	NA	0	227	1.0000
bta03020	RNA polymerase	NA	0	227	1.0000
bta03022	Basal transcription factors	NA	0	227	1.0000
bta03410	Base excision repair	NA	0	227	1.0000
bta03420	Nucleotide excision repair	NA	0	227	1.0000
bta03450	n-homologous end-joining	NA	0	227	1.0000
bta03460	Fanconi anemia pathway	NA	0	227	1.0000
bta04060	Cytokine-cytokine receptor interaction	NA	0	227	1.0000
bta04064	NF-kappa B signaling pathway	NA	0	227	1.0000
bta04080	Neuroactive ligand-receptor interaction	NA	0	227	1.0000
bta04122	Sulfur relay system	NA	0	227	1.0000
bta04140	Regulation of autophagy	NA	0	227	1.0000
bta04320	Dorso-ventral axis formation	NA	0	227	1.0000
bta04340	Hedgehog signaling pathway	NA	0	227	1.0000
bta04370	VEGF signaling pathway	NA	0	227	1.0000
bta04540	Gap junction	NA	0	227	1.0000
bta04614	Renin-angiotensin system	NA	0	227	1.0000
bta04620	Toll-like receptor signaling pathway	NA	0	227	1.0000
bta04621	D-like receptor signaling pathway	NA	0	227	1.0000
bta04622	RIG-I-like receptor signaling pathway	NA	0	227	1.0000
bta04623	Cytosolic DNA-sensing pathway	NA	0	227	1.0000
bta04630	Jak-STAT signaling pathway	NA	0	227	1.0000
bta04650	Natural killer cell mediated cytotoxicity	NA	0	227	1.0000
bta04664	Fc epsilon RI signaling pathway	NA	0	227	1.0000
bta04668	TNF signaling pathway	NA	0	227	1.0000
bta04672	Intestinal immune network for IgA production	NA	0	227	1.0000
bta04710	Circadian rhythm	NA	0	227	1.0000
bta04742	Taste transduction	NA	0	227	1.0000
bta04913	Ovarian steroidogenesis	NA	0	227	1.0000
bta04914	Progesterone-mediated oocyte maturation	NA	0	227	1.0000
bta04917	Prolactin signaling pathway	NA	0	227	1.0000
bta04940	Type I diabetes mellitus	NA	0	227	1.0000
bta04950	Maturity onset diabetes of the young	NA	0	227	1.0000

bta05140	Leishmaniasis	NA	0	227	1.0000
bta05143	African trypasomiasis	NA	0	227	1.0000
bta05161	Hepatitis B	NA	0	227	1.0000
bta05210	Colorectal cancer	NA	0	227	1.0000
bta05212	Pancreatic cancer	NA	0	227	1.0000
bta05213	Endometrial cancer	NA	0	227	1.0000
bta05217	Basal cell carcima	NA	0	227	1.0000
bta05218	Melama	NA	0	227	1.0000
bta05219	Bladder cancer	NA	0	227	1.0000
bta05223	n-small cell lung cancer	NA	0	227	1.0000
bta05320	Autoimmune thyroid disease	NA	0	227	1.0000
bta05321	Inflammatory bowel disease （IBD)	NA	0	227	1.0000
bta05330	Allograft rejection	NA	0	227	1.0000
bta05332	Graft-versus-host disease	NA	0	227	1.0000

No. of Mapping SP indicated the number of the significantly expressed proteins mapping to one certain kegg pathway by the KEGG analysis; No. of Mapping TP indicated the number of the total significantly expressed proteins mapping by the KEGG analysis. NA indicated no significantly expressed proteins were mapped to some KEGG pathways.

Table S11 The pathways mapped by differentially changed transcripts/proteins in mammary glands between RS- and AH-ffed animals

KEGG id	KEGG pathway description	No. of increased transcripts	ID of increased transcripts	No. of decreased transcrip ts	ID of decreased transcripts	No. of up-regulated proteins	ID of up-regulated proteins	No. of down-regulated proteins	ID of down-regulated proteins
bta01100	Metabolic pathways	20	XLOC_023921;<br>XLOC_023703;<br>XLOC_024260;<br>XLOC_023606;<br>XLOC_023922;<br>XLOC_023555;<br>XLOC_023596;<br>XLOC_003697;<br>XLOC_024196;<br>XLOC_024053;<br>XLOC_023529;<br>XLOC_017018;<br>XLOC_020698;<br>XLOC_012858;<br>XLOC_024302;<br>XLOC_011982;<br>XLOC_023920;<br>XLOC_020095;<br>XLOC_010523;<br>XLOC_023501.	1	XLOC_005319	29	IPI00712671;<br>IPI00716555;<br>IPI00702650;<br>IPI00686225;<br>IPI00703753;<br>IPI00711759;<br>IPI00700920;<br>IPI00742596;<br>IPI00708438;<br>IPI00716195;<br>IPI00692819;<br>IPI00694312;<br>IPI00706203;<br>IPI00706942;<br>IPI00730144;<br>IPI00694739;<br>IPI00842934;<br>IPI00689323;<br>IPI00686601;<br>IPI00904104;<br>IPI00883375;<br>IPI00694849;<br>IPI00944429;<br>IPI00726650;<br>IPI00696912;<br>IPI00692627;<br>IPI00711233;<br>IPI00698589;<br>IPI00712677.	22	IPI00871114;<br>IPI00688977;<br>IPI00698059;<br>IPI00708246;<br>IPI00706879;<br>IPI00702251;<br>IPI00705565;<br>IPI00838420;<br>IPI00702768;<br>IPI00715066;<br>IPI00704332;<br>IPI00687661;<br>IPI00760457;<br>IPI00694430;<br>IPI00695952;<br>IPI00699817;<br>IPI00699372;<br>IPI00703084;<br>IPI00690789;<br>IPI00827121;<br>IPI00714621;<br>IPI00727591.

bta03010	Ribosome	2	XLOC_002615; XLOC_028013	0	2	IPI00713536; IPI00715091	21	IPI00709409; IPI00702948; IPI00716860; IPI00708210; IPI00694688; IPI00689565; IPI00688727; IPI00694444; IPI00707386; IPI00718379; IPI00698077; IPI00694565; IPI00707537; IPI00699827; IPI00712384; IPI00705739; IPI00715868; IPI00687634; IPI00717048; IPI00710859; IPI00687662.
bta04151	PI3K-Akt signaling pathway	0		0	5	IPI00694504; IPI00715486; IPI00714673; IPI00713573; IPI00837992.	10	IPI00690309; IPI00697595; IPI00702948; IPI00712524; IPI00707857; IPI00731432; IPI01017588; IPI00708244; IPI00715059; IPI00826022.
bta04512	ECM-receptor interaction	1	XLOC_012279	0	6	IPI00694504; IPI00707359; IPI00715486; IPI00714673; IPI00713573; IPI00837992;	8	IPI00697595; IPI00708824; IPI00712524; IPI00710204; IPI00707857; IPI00731432; IPI00708244; IPI00826022.

bta04974	Protein digestion and absorption	0		0		3	IPI00717116; IPI00713573; IPI00837992.	6	IPI00705159; IPI00712524; IPI00707857; IPI00731432; IPI00708244; IPI00826022.
bta04141	Protein processing in endoplasmic reticulum	1	XLOC_023448	1	XLOC_003872	7	IPI00699107; IPI00867339; IPI00700547; IPI00691963; IPI00689325; IPI00702891; IPI00696616.	2	IPI00692963; IPI00690581.
bta03013	RNA transport	1	XLOC_003001	0		5	IPI00705941; IPI00702620; IPI00712775; IPI00690232; IPI00693324.	2	IPI00689226; IPI00701747
bta03040	Spliceosome	2	XLOC_023994; XLOC_012182	0		4	IPI00867339; IPI00690232; IPI00695600; IPI00717759.	3	IPI00686595; IPI00688337; IPI00687395.
bta04020	Calcium signaling pathway	0		0		3	IPI00688651; IPI00695508; IPI00703129.	3	IPI00706314; IPI00693452; IPI00705378.
bta04142	Lysosome	3	XLOC_024165; XLOC_003080; XLOC_023509.	0		4	IPI00716195; IPI00706203; IPI00710408; IPI00718311.	2	IPI00698823; IPI00699372.
bta04910	Insulin signaling pathway	1	XLOC_023531	1	XLOC_026730	2	IPI00695508; IPI00699798.	4	IPI00690309; IPI00906031; IPI00702948; IPI00715059.
bta04310	Wnt signaling pathway	0		0		4	IPI00688651; IPI00699355; IPI00690446; IPI00708311.	1	IPI00712288
bta01230	Biosynthesis of amino acids	0		0		3	IPI00706942; IPI00904104; IPI00698589.	2	IPI00715066; IPI00760457.

bta04530	Tight junction	0		2	XLOC_016188; XLOC_020249.	1	IPI00698900	4	IPI00713313; IPI00695042; IPI00692417; IPI00716830.
bta00230	Purine metabolism	1	XLOC_002658	0		5	IPI00716555; IPI00686225; IPI00703753; IPI00730144; IPI00726650.	0	
bta01200	Carbon metabolism	0		0		5	IPI00708438; IPI00706942; IPI00904104; IPI00696912; IPI00698589.		
bta00190	Oxidative phosphorylation	1	XLOC_023495	0		4	IPI00716195; IPI00694849; IPI00711233; IPI00712677.	1	IPI00699372
bta00330	Arginine and proline metabolism	0		0		1	IPI00711759	3	IPI00698059; IPI00838420; IPI00760457.
bta04146	Peroxisome	0		0		2	IPI00692468; IPI00686601.	2	IPI00702251; IPI00694430.
bta03050	Proteasome	0		0		4	IPI00695176; IPI00701790; IPI00707875; IPI00697757.	0	
bta00410	beta-Alanine metabolism	0		0		2	IPI00702650; IPI00711759.	1	IPI00694430
bta00270	Cysteine and methionine metabolism	0		0		3	IPI00711759; IPI00694739; IPI00883375.	0	
bta04150	mTOR signaling pathway	0		0		0		3	IPI00690309; IPI00702948; IPI00715059.
bta03008	Ribosome biogenesis in eukaryotes	1	XLOC_023915	0		2	IPI00705941; IPI00708018.	1	IPI00852474

bta03320	PPAR signaling pathway	1	XLOC_024190	1	XLOC_024579	2	IPI00699355; IPI00686601.	1	IPI00710204
bta00970	Aminoacyl-tRNA biosynthesis	0		0		0		3	IPI00708246; IPI00689365; IPI00867017.
bta00010	Glycolysis / Gluconeogenesis	0		0		3	IPI00706942; IPI00696912; IPI00698589.	0	
bta04120	Ubiquitin mediated proteolysis	3	XLOC_023609; XLOC_024105; XLOC_024155.	0		0		3	IPI00718678; IPI00697917; IPI00704041.
bta04010	MAPK signaling pathway	1	XLOC_023520	1	XLOC_013166	2	IPI00867339; IPI00703776.	1	IPI00695042
bta00380	Tryptophan metabolism	0		0		1	IPI00702650	1	IPI00698059
bta00260	Glycine, serine and threonine metabolism	0		0		1	IPI00698589	1	IPI00698059
bta00620	Pyruvate metabolism	1	XLOC_002658	0		2	IPI00700920; IPI00696912.	0	
bta00290	Valine, leucine and isoleucine biosynthesis	0		0		0		1	IPI00715066
bta00910	Nitrogen metabolism	0		0		0		1	IPI00760457
bta00072	Synthesis and degradation of ketone bodies	0		0		1	IPI00694312	0	
bta00360	Phenylalanine metabolism	0		0		0		1	IPI00698059
bta00340	Histidine metabolism	0		0		0		1	IPI00698059
bta00350	Tyrosine metabolism	0		0		0		1	IPI00698059
bta00250	Alanine, aspartate and glutamate metabolism	0		0		0		1	IPI00760457
bta03060	Protein export	1	XLOC_023666	0		0		1	IPI00685804

bta00030	Pentose phosphate pathway	0	0	0	1	IPI00685804
bta00020	Citrate cycle (TCA cycle)	0	0	0	1	IPI00685804

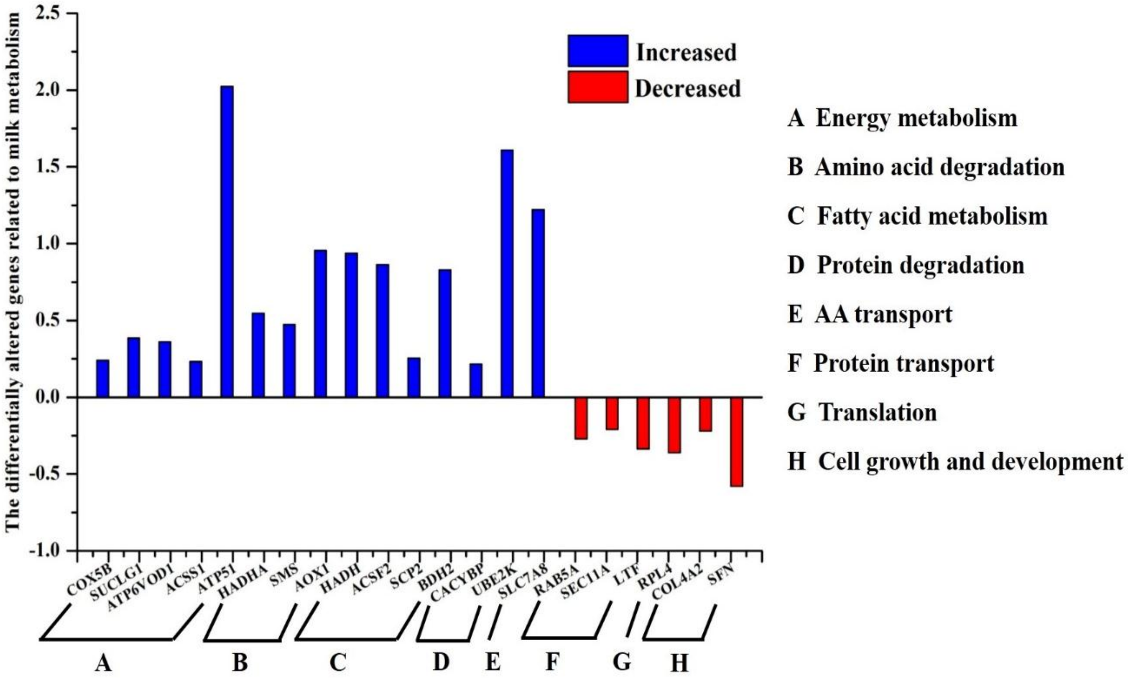

Figure S2. The differentially altered genes related to milk metabolism

Blue bar indicates the fold change of increased genes and the red bar indicates the fold change of decreased genes. A, B, C, D, E, F, G, H represent the functional classifications of these genes, respectively.

Table S12. The full name and abbreviation of genes in Figure 5 and figure 6

Gene symbol	Gene annotation	Function classification
COX5B	Cytochrome c oxidase subunit 5B, mitochondrial	Energy metabolism
SUCLG1	Succinyl-CoA ligase [GDP-forming] subunit alpha, mitochondrial	Energy metabolism
ATP6VOD1	V-type proton ATPase subunit d 1	Energy metabolism
ACSS1	Acetyl-CoA synthetase 1	Energy metabolism
ATP51	ATP synthase, H+ transporting, mitochondrial Fo complex, subunit E	Energy metabolism
HADHA	FGF-2 binding protein	Amino acid degradation
SMS	Spermine synthase	Amino acid degradation
AOX1	Aldehyde oxidase 1	Amino acid degradation
HADH	Hydroxyacyl-CoA dehydrogenase	Fatty acid metabolism
ACSF2	Acyl-CoA synthetase family member 2, mitochondrial	Fatty acid metabolism
SCP2	Isoform SCPx of Non-specific lipid-transfer protein	Fatty acid metabolism
BDH2	3-hydroxybutyrate dehydrogenase type 2	Fatty acid metabolism
CACYBP	Calcyclin-binding protein	Protein degradation
UBE2K	Ubiquitin-conjugating enzyme E2K	Protein degradation
SLC7A8	Solute carrier family 7 (amino acid transporter light chain, L system), member 8	AA transport
RAB5A	Ras-related protein Rab-5A	Protein transport
SEC11A	Signal peptidase complex catalytic subunit SEC11A	Protein transport
LTF	Lactotransferrin	Protein transport
RPL4	60S ribosomal protein L4	Translation
COL4A2	collagen, type IV, alpha 2, partial	Cell growth and development
SFN	14-3-3 protein sigma	Cell growth and development

Table S13. The full name and abbreviation of proteins in Figure 7

Gene symbol	Abbreviation	Gene annotation	Function classification
PPA2	Ppa2	Pyrophosphatase (inorganic) 2	Energy metabolism
ACYP2	Acyp2	Acylphosphatase 2, muscle type	Energy metabolism
SUCLG1	Suclg1	Succinyl-CoA ligase [GDP-forming] subunit alpha, mitochondrial	Energy metabolism
ACSS1	Acss1	Acetyl-CoA synthetase 1	Energy metabolism
COX5B	Cox5b	Cytochrome c oxidase subunit 5B, mitochondrial	Energy metabolism
NDUFB8	Ndu8	NADH dehydrogenase [ubiquinone] 1 beta subcomplex subunit 8, mitochondrial	Energy metabolism
ATP6V0D1	ATP601	V-type proton ATPase subunit d 1	Energy metabolism
NDUFB4	Ndu4	NADH dehydrogenase [ubiquinone] 1 beta subcomplex subunit 4	Energy metabolism
PGAM1	Pgam1	Phosphoglycerate mutase 1	Energy metabolism
TPI1	Tpi1	Triosephosphate isomerase	Energy metabolism
UBE2K	Ube2k	Ubiquitin-conjugating enzyme E2K	Protein degradation
UBA6	Uba6	Ubiquitin-like modifier activating enzyme 6)	Protein degradation
RCHY1	Rchy1	Ring finger and CHY zinc finger domain containing 1, E3 ubiquitin protein ligase	Protein degradation
HADHA	Hadha	FGF-2 binding protein	Protein degradation
PSMB9	Psmb9	Proteasome subunit beta type-9	Protein degradation
PSMC3	Psmc3	26S protease regulatory subunit 6A	Protein degradation
PSMA5	Psma5	Proteasome subunit alpha type-5	Protein degradation
PSMB4	Psmb4	Proteasome subunit beta type-4	Protein degradation
RPL15	Rpl15	60S ribosomal protein L15	Protein synthesis
RPS6	Rps6	40S ribosomal protein S6	Protein synthesis
RPL36	Rpl36	60S ribosomal protein L36	Protein synthesis
RPL10	Rpl10	60S ribosomal protein L10	Protein synthesis
RPS26	Rps26	40S ribosomal protein S26	Protein synthesis
RPL17	Rpl17	60S ribosomal protein L17	Protein synthesis
RPS13	Rps13	40S ribosomal protein S13	Protein synthesis
RPL27A	Rpl27A	60S ribosomal protein L27a	Protein synthesis
RPL18A	Rpl18A	60S ribosomal protein L18a	Protein synthesis
RPL18	Rpl18	60S ribosomal protein L18	Protein synthesis
RPL8	Rpl8	60S ribosomal protein L8	Protein synthesis
RPS9	Rps9	40S ribosomal protein S9	Protein synthesis
RPL28	Rpl28	60S ribosomal protein L28	Protein synthesis
RPL4	Rpl4	60S ribosomal protein L4	Protein synthesis
RPL13A	Rpl13A	60S ribosomal protein L13a	Protein synthesis
RPL13	Rpl13	60S ribosomal protein L13	Protein synthesis
RPL3	Rpl3	60S ribosomal protein L3	Protein synthesis
RPL6	Rpl6	60S ribosomal protein L6	Protein synthesis
RPL19	Rpl19	60S ribosomal protein L19	Protein synthesis
HADH	Hadha	Hydroxyacyl-CoA dehydrogenase	Fatty acid metabolism
ACSF2	Acsf2	Acyl-CoA synthetase family member 2, mitochondrial	Fatty acid metabolism
BDH2	Bdh2	3-hydroxybutyrate dehydrogenase type 2	Fatty acid metabolism
SCD5	Scd5	Stearoyl-CoA desaturase 5	Fatty acid metabolism
SCP2	Scp2	Isoform SCPx of Non-specific lipid-transfer protein	Fatty acid metabolism
BDH2	Bdh2	3-hydroxybutyrate dehydrogenase type 2	Fatty acid metabolism
HADHA	Hadha	FGF-2 binding protein	Fatty acid metabolism
SOD2	Sod2	Superoxide dismutase [Mn], mitochondrial	Fatty acid metabolism
COL4A2	Col4a2	Collagen, type IV, alpha 2, partial	Cell growth and development
COL1A1	Col1a1	Collagen alpha-1(I) chain	Cell growth and development
COL3A1	Col3a1	Collagen, type III, alpha 1	Cell growth and development
COL1A2	Col1a2	Collagen alpha-2(I) chain	Cell growth and development
IRS1	Irs1	Insulin receptor substrate 1	Cell growth and development
RAB5A	Rab5a	Ras-related protein Rab-5A	Protein transport
SEC11A	Sec11a	Signal peptidase complex catalytic subunit SEC11A	Protein transport
LTF	Ltf	Lactotransferrin	Protein transport
SLC7A8	Slc7a8	Solute carrier family 7 (amino acid transporter light chain, L system), member 8	AA transport
DNAJB11	Dnajb11	DnaJ homolog subfamily B member 11	Protein processing
HSPA2	Hspa2	Heat shock 70kDa protein 1A	Protein processing
FBXO6	Fbxo6	F-box only protein 6	Protein processing

CALR	Calr	Calreticulin	Protein processing
PDIA3	Pdia3	Protein disulfide-isomerase A3	Protein processing
ERP29	Erp29	Endoplasmic reticulum resident protein 29	Protein processing
SSR2	Ssr2	Translocon-associated protein subunit beta	Protein processing
MAGOHB	Magohb	Protein mago nashi homolog 2	Protein processing
U2AF1	U2Af1	Splicing factor U2AF 35 kDa subunit	Protein processing
TRA2B	Tra2B	Transformer-2 protein homolog beta	Protein processing
MAOA	Maoa	Amine oxidase [flavin-containing] A	AA metabolism
P4HA2	P4Ha2	prolyl 4-hydroxylase subunit alpha-2 isoform 2	AA metabolism
GLUL	Glul	Glutamine synthetase	AA metabolism
BCAT2	Bcat2	Branched-chain-amino-acid aminotransferase, mitochondrial	AA metabolism

Table S14 The details of the primers used for real-time PCR in the experiment

Gene symbol	NCBI accession	Primers	Length (bp)
COX5B	NM_001034046.2	F:GGCGTCTGGAGGTGGTGTT<br>R:TTGTTGGTGATGGA GGGGAC	165
SUCLG1	NM_001035082.1	F:ACGAAGATTATTTGCCAGGGTT<br>R:ACAGCAAAGGGAGGAGGGAC	212
ATP6VOD1	NM_174505.3	F:GTGCTCAGCCAGGCGGACTA<br>R:TGACGGAACTCCACCACCATC	173
ACSS1	NM_174746.2	F:ACACGGTGAGCGACTGCGAC<br>R:CTCGGCGGACTCCATACCTC	231
ATP5I	NM_176639.2	F:CGAGGGTCACGGACAGTATGG<br>R:TATGGTATCCTCTTGGGCTTCC	224
HADHA	NM_174335.2	F:TGCGGTCTTTGGGCTTGG<br>R:AGTGGGCGAGTGGGCAGG	215
SMS	NM_001035471.1	F:GGATGGTCGCCTGGTTGAG<br>R:CCCCCGTCTCCACCTCCC	228
AOX1	NM_176668.3	F:CTGCCGTCACCACAGTAGAAGG<br>R:TACCACCAAGAGCATCATTTAGC	189
HADH	NM_174334.3	F:CTTGGTGACCCTGCCGAGTA<br>R:TACTGGGCTTCCTTCCAAACTG	191
ACSF2	NM_001078112.1	F:TCCGTGGCGGCGAGAATA<br>R:CATTCGGTCATCCTTCACTCC	104
SCP2	NM_001033990.3	F:CGCTTCACAGTCTCCGCTCC<br>R:TGATAGATAGCCCTCTGCCCAC	219
BDH2	NM_001034488.2	F:TGCCAAAGTCATAGCCACCG<br>R:GTGATGGACGAAACCAGCAA	172
CACYBP	NM_001034809.2	F:CTTACAGAAAATGAGAAGCCAGC<br>R:AGCAGGCA CTT GAT GAACT CC	144
UBE2K	NM_174080.2	F:CAGCGAATCAAGCGGGAGT<br>R:AAACAAATAGCCCCTGTGACGG	260
SLC7A8	NM_001192889.2	F:TCTGGAATCTTCGTCTCGCC<br>R:GCCTGGTTGGTGGGGTA GATT	242
RAB5A	NM_001076186.1	F:CCAGCGTAAACTCCCGAATG<br>R:CCACCACT GCCGA GACA CG	201
SEC11A	NM_174465.2	F:GATGTGCGGCGGATGAAC<br>R:CTGCCACTGAGCACCACTACA	143
LTF	NM_180998.2	F:CTGGGCGTGACCCGAAAT<br>R:GCTCTCTCCATTCGTGTTCTCC	191
RPL4	NM_001014894.1	F:GCAAAAACAACAGACAGCCCTA<br>R:TTCACTCTGCGGT GCCAACG	214
COL4A2	XM_015465832.1	F:TCGGAGCAGACGGACAGGAG<br>R:TCGGCTCCTGAAAATCCTGG	133
SFN	NM_001075912.1	F:AAATGAAGGGCGACTACTACCG<br>R:GTGGGCGGCATCTCCTTC	130
